# Risk of Malnutrition in Digestive System Cancers: A Systematic Review and Meta-Analysis

**DOI:** 10.3390/cancers18010080

**Published:** 2025-12-26

**Authors:** Bettina Csilla Budai, Petrana Martinekova, Gefu Cai, Dalma Dobszai, Lili Fekete, Hanne Aspelund Normann, Jázmin Németh, Alíz Fazekas, Eszter Ágnes Szalai, Andrea Szentesi, Vasile Liviu Drug, Péter Hegyi, Stefania Bunduc

**Affiliations:** 1 Centre for Translational Medicine, Semmelweis University, 1085 Budapest, Hungary; budai.bettina@semmelweis.hu (B.C.B.); stfnbndc@gmail.com (S.B.); 2 Institute of Pancreatic Diseases, Semmelweis University, 1085 Budapest, Hungary; 3 Institute for Translational Medicine, Medical School, University of Pécs, 7624 Pécs, Hungary; 4 EDU. A Degree Smarter, 1320 Kalkara, Malta; 5 Department of Biophysics and Radiation Biology, Semmelweis University, 1085 Budapest, Hungary; 6 Department of Restorative Dentistry and Endodontics, Semmelweis University, 1085 Budapest, Hungary; 7 Gastroenterology Department, Grigore T. Popa University of Medicine and Pharmacy, 700115 Iași, Romania; 8 Translational Pancreatology Research Group, Interdisciplinary Centre of Excellence for Research Development and Innovation, University of Szeged, 6720 Szeged, Hungary; 9 Carol Davila University of Medicine and Pharmacy, 050474 Bucharest, Romania; 10 Digestive Diseases and Liver Transplantation Center, Fundeni Clinical Institute, 022328 Bucharest, Romania

**Keywords:** undernutrition, digestive system cancer, cachexia, malnourishment, nutrition status

## Abstract

**Background/Objectives**: Incidence of malnutrition varies greatly among gastrointestinal (GI) cancer patients and has a major impact on prognosis. We performed a meta-analysis to identify risk factors for malnutrition risk, malnutrition diagnosis, and cachexia in patients with GI cancer. **Methods**: A systematic search was performed on 31 October 2025 on the PubMed (Medline), Embase, and Cochrane Library databases. Eligible studies reported on risk factors for malnutrition risk, malnutrition diagnosis, malnutrition-related complication risk and cachexia in adult patients with GI cancer. Articles on neuroendocrine tumours, primary cancer outside the GI tract, and the paediatric population were excluded. The random-effects model yielded the pooled odds ratios (ORs) and 95% confidence intervals (CIs) for the investigated risk factors. **Results**: A total of 37,624 records were identified. Data from 262,525 patients from 578 articles were included in the analysis. Older age (≥65) was associated with higher odds for malnutrition risk across all GI cancers. In gastric cancer, males had a lower odds for malnutrition risk (OR 0.84; 95% CI 0.75–0.95); however, the sex difference across other cancer types was heterogeneous, and mostly not significant. Tumour location influenced the odds for malnutrition-related complication risk in pancreatic ductal adenocarcinoma (head vs. body/tail—OR 1.48; 95% CI 0.98–2.23) and colorectal cancer (colon vs. rectal—OR 1.39; 95% CI 1.07–1.81; right-sided vs. left-sided—OR 1.54; 95% CI 1.34–1.77). Increased C-reactive protein alone indicated higher odds for malnutrition risk at baseline. **Conclusions**: Inflammatory biomarkers and tumour characteristics may indicate malnutrition risk in GI cancer at baseline. There is a great need for standardised and harmonised approaches in nutritional status assessment in GI cancer.

## Simple Summary

Cancer cachexia accounts for up to 22% of cancer-related deaths. The highest prevalence of malnutrition is reported in pancreatic and upper gastrointestinal malignancies. Malnutrition risk, malnutrition diagnosis, and cachexia are consistently associated with adverse clinical outcomes. The aim of this systematic review and meta-analysis was to identify risk factors contributing to these nutritional conditions. For risk assessment, symptom-based screening tools were used, the biological composite score was used to assess the malnutrition-related complication risk, and the diagnosis of malnutrition was determined according to the GLIM criteria. Definitions of cachexia varied across the included articles. Our findings indicate that age, cancer stage, and systemic inflammation are significantly associated with malnutrition risk, while site-specific differences are revealed in colorectal and pancreatic cancers. In esophageal cancer, different types of assessment tools produced discordant results regarding neoadjuvant therapy as a risk factor for malnutrition. These results highlight the need for standardised and context-appropriate approaches to the assessment of malnutrition and cachexia in gastrointestinal cancers.

## 1. Introduction

According to GLOBOCAN data, digestive system cancers account for 26% of global cancer incidence and for up to 35% of cancer-related mortality [1]. Up to 22% of cancer deaths are caused by cancer cachexia [2]. Gastrointestinal (GI) and hepato-biliopancreatic (HBP) cancers are associated with various rates of malnutrition. The highest prevalence is reported in pancreatic cancer (51%) and upper gastrointestinal (UGI) tract cancers (40%), followed by colorectal cancer (30%) [3].

Malnutrition can cause a variety of adverse events, such as increased treatment toxicity, reduced physical function, delayed wound healing, increased postoperative mortality, and overall worsened clinical outcomes [4]. In addition, malnutrition increases healthcare costs [5].

A variety of tools are available for malnutrition assessment; some are biological composite scores [6,7,8,9], while others are used for screening based on clinical signs and symptoms [10,11,12,13,14,15,16,17]. Since 2019, the Global Leadership Initiative of Malnutrition (GLIM) has provided a standardised framework for diagnosing malnutrition across various clinical settings [18,19].

Although a wide range of malnutrition assessment tools are available, not all are suitable in certain clinical scenarios, and there is still no universally accepted gold standard for malnutrition assessment. Assessment tools differ in their applicability, and each carries inherent limitations, including interobserver variability, reproducibility challenges, dependence on evaluator expertise, time consumption, high costs, and, in some cases, lack of validation [20].

The aetiology of malnutrition in GI cancer patients is multifactorial. Tumour-related factors such as dysphagia, gastrointestinal obstruction, ascites, anaemia, and anorexia directly impair nutrient intake and absorption. Additionally, older age [3] or anticancer treatments, including radiotherapy and surgical interventions, play an important role [21]. Finally, disease progression itself influences nutritional status. Advanced cancer stage is associated with a heightened catabolic state and systemic inflammation, which further increases the risk of malnutrition [22]. Despite these well-established associations, malnutrition remains underdiagnosed and undertreated in clinical practice, significantly worsening prognosis and compromising patient outcomes [23].

Most of the studies investigate malnutrition as a risk factor for patients’ outcomes [24,25], but the reasons for the variation in rates and severity of malnutrition in patients with digestive system cancer are incompletely elucidated.

The purpose of our study was to evaluate any reported risk factors that can significantly influence malnutrition risk, malnutrition diagnosis, or cachexia in patients with digestive system cancers.

## 2. Materials and Methods

We followed the Cochrane Handbook and the PRISMA (Preferred Reporting Items for Systematic Reviews and Meta-Analyses) guideline recommendations (Supplementary Table S1) [26]. The protocol was registered on PROSPERO (CRD42022369200). A protocol deviation occurred by expanding the search key, as detailed in Supplementary Document S1. This work was carried out as part of the Systems Education Program at the Hungarian Pancreatic Study Group [27,28].

### 2.1. Search Strategy

We performed the original systematic search using the updated search key on PubMed, Embase, and the Cochrane Library from inception to 28 February 2023, without any restrictions or filters. We updated our search on 31 October 2025. Our search key had three main domains comprising the terms ‘cancer,’ ‘gastrointestinal tract,’ and ‘malnutrition.’ We included all the identified synonyms and variants, considering plural forms and British and American English of these terms from the literature. The three domains were connected using the “AND” Boolean operator. The complete search key is in the Supplementary Document S2. Eligible articles’ references were also screened for further eligible studies.

### 2.2. Eligibility Criteria

The eligibility criteria were defined within the PECOS framework—Population, Exposure, Control, Outcome, and Study design. Articles reporting on adult patients with confirmed digestive system cancer (P) were selected. The exposure (E) included all population or disease characteristics reported across the eligible articles in relation to malnutrition risk, malnutrition-related complication risk, diagnosis, or cachexia. Any potential risk factors we could identify were considered as they were evaluated at baseline. The comparator group (C) comprised non-exposed patients. The outcome of interest (O) was impaired nutritional status. Impaired nutritional status included the following: risk for malnutrition, measured by validated screening tools, identifying patients at increased risk of current or imminent malnutrition, but does not constitute a formal diagnosis of malnutrition; and risk of malnutrition-related complications, the diagnosis of malnutrition [18] or cachexia (definitions are detailed in Supplementary Document S3), both at baseline and during follow-up. Since there is no clear consensus on the definition of cachexia [29], all articles reporting cachexia were included (detailed definitions available in Supplementary Document S3). Regarding study design, (S) we included peer-reviewed observational cohort and cross-sectional studies and randomised controlled trials. Articles on neuroendocrine tumours, primary cancer outside the GI tract, paediatric and adolescent population, case reports, case series, reviews, and preclinical studies were excluded.

### 2.3. Selection Process

After removing duplicates, the selection was performed by three pairs of independent authors (B.C.B. with G.C., L.F. with P.M., and H.A.N. with D.D.), first according to the title and abstract using a specific selection tool (Rayyan) [30], then by full-text content. After each selection step, Cohen’s Kappa was calculated to evaluate the agreement between the selection teams. Discrepancies were solved by a third investigator (B.C.B. or P.M.). During the selection based on full-text content, reasons for exclusion were documented. The following categories were identified: ‘outcome of interest is not reported,’ ‘not only gastrointestinal cancer population,’ ‘the definition of malnutrition is not provided,’ ‘ineligible study type,’ and ‘prevalence of malnutrition is not reported.’ The selection process is detailed in Supplementary Document S4.

### 2.4. Data Collection Process and Data Items

The data was collected manually by two independent reviewers using a pre-designed, standardised data collection (Excel) sheet to reduce potential errors. The extracted data included information about the study—first author, year of publication, country of origin, study design; demographic data—sample size, sex, and age distribution; population characteristics—cancer type, cancer stage; cut-off values of the reported risk factors (exposure); and outcome-related information—definition of malnutrition, the malnutrition assessment tool (including cut-off values), and moment of measurement. We extracted the raw numbers, or available odds ratios (ORs), and the corresponding 95% confidence intervals (CIs) as reported. The two independent reviewers cross-checked each other’s data pool to ensure accuracy and to solve discrepancies. The detailed strategy for data collection is available in Supplementary Document S5.

### 2.5. Risk of Bias Assessment

For the risk of bias assessment, we used the Quality in Prognostic Studies (QUIPS) tool to assess the methodology quality of the included studies [31]. A total of 289 articles were assessed by two independent reviewers, B.C.B. and G.C.; another set of 289 articles was checked independently by L.F. and P.M. Any disagreements that arose were resolved by consensus. The assessed domains were statistical analysis reporting, study confounding, outcome measurement, prognostic factor measurement, study attrition, and study participation. The definitions were as follows: overall low risk of bias if all domains were associated with a low risk, or if for one domain the risk of bias was moderate, overall high risk of bias if one domain was associated with a high risk of bias, or if three or more domains had a moderate risk, and overall moderate risk of bias corresponding to all in between cases.

### 2.6. Synthesis Methods

As we assumed considerable between-study heterogeneity in all cases, we used random-effects models to pool effect sizes in a frequentist framework. The pooled effect size was expressed as odds ratio (OR) with the corresponding 95% confidence interval (CI). We reported the results as the odds of malnutrition in the exposed versus the comparator group. The Mantel–Haenszel method was applied to calculate the OR based on the extracted data as the number of patients with malnutrition and the total sample size for the exposed and comparator groups. A Hartung–Knapp adjustment [32,33,34,35] was applied for the CI calculation of pooled OR (also for calculating *t*-test-based *p*-value). To estimate the between-study variance measure (τ), the Paule-Mandel [36] method was applied with the Q profile method for the CI [37]. Heterogeneity was assessed by Higgins and Thompson’s I2 statistics too [38].

Small study publication bias was assessed by visual inspection of funnel plots and calculating the Harbord (modified Egger) test *p*-value [39,40]. The test has a limited diagnostic assessment, with fewer than 10 studies; we analysed a minimum of 10 studies reporting the outcome of interest. We performed a thorough sensitivity analysis through several methods to identify potential outliers as recommended by Harrer et al. [41], which are detailed in Supplementary Document S6.

All statistical analyses were calculated by R software v4.2.2 [42] using the meta [43] package for basic meta-analysis calculations and plots, and the dmetar [44] package for additional influential analysis calculations and plots.

Several moderator analyses were performed; namely, by cancer type and definition of tools using nutritional status assessment. A mixed-effects model (also known as fixed-effects “plural” model) was used (each moderator was analysed in a separate model), assuming different τ^2^ in the subgroups. A heterogeneity-based Q test was used to compare the subgroups. For the continuous moderator (ratio of females), we made a meta-regression assuming a linear relation between the moderator values and the logarithm of odds ratios. A Wald-type *p*-value and confidence interval are given. The null hypothesis was rejected at a 5% significance level. We did not perform multiplicity correction for the large number of outcomes examined in our exploratory analysis.

The publications potentially reporting on the same study were easily identified by authors’ names, study centre, and period. To avoid the risk of overlapping populations in our analyses in these cases, we prioritised the reports, including the higher number of patients. The complete strategy for avoiding overlapping populations is detailed in Supplementary Document S7.

Results were visually displayed as forest plots, and the comparisons were considered statistically significant if the CI of OR did not contain the value of one. Where applicable, such as when the study number was large enough and not too heterogeneous, we reported the prediction intervals (i.e., the expected range of true effects of future studies). Besides the classical forest plots pooling the results of individual studies, to summarise and increase the clarity of the large number of our findings, we have also created aggregated forest plots showing in one figure several pooled OR values with their corresponding CIs.

## 3. Results

### 3.1. Search and Selection

**Figure 1:**
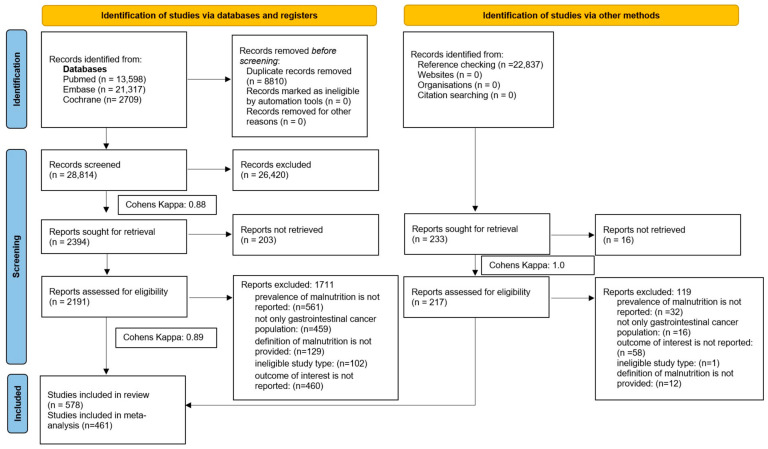
PRISMA flowchart of the selection.

The selection flowchart is summarised in Figure 1. Our search key identified a total of 37,624 records. An additional 98 articles were found by screening the references of included papers. We included 578 full texts in our systematic review, and 461 articles were eligible for quantitative analysis. The references of the included studies are found in Supplementary Document S8. The strategy for retrieval and the reasons for exclusion are detailed in Supplementary Document S9.

### 3.2. Study Characteristics

The main characteristics of the included studies are summarised in the Supplementary Table S2.

Based on the available data, we evaluated the risk factors separately for being at malnutrition risk, for malnutrition-related complication risk, for malnutrition diagnosis, and for cachexia, according to cancer type and the assessment tools, as detailed in Table 1. We distinguished between malnutrition screening tools, which focus on symptoms like weight loss or loss of appetite (symptom-based tools). We separately analysed malnutrition-related complication risk based on tools that incorporate biological composite scores such as serum levels of albumin or total lymphocyte count. This classification aligns with the existing literature [20]. Additionally, we analysed malnutrition diagnosis as per GLIM [18] and cachexia separately. All assessments were performed at baseline. The cut-off values for malnutrition risk assessment varied slightly across the studies—we considered the risk and diagnosis of malnutrition and cachexia, as defined in each article.

**Figure 2:**
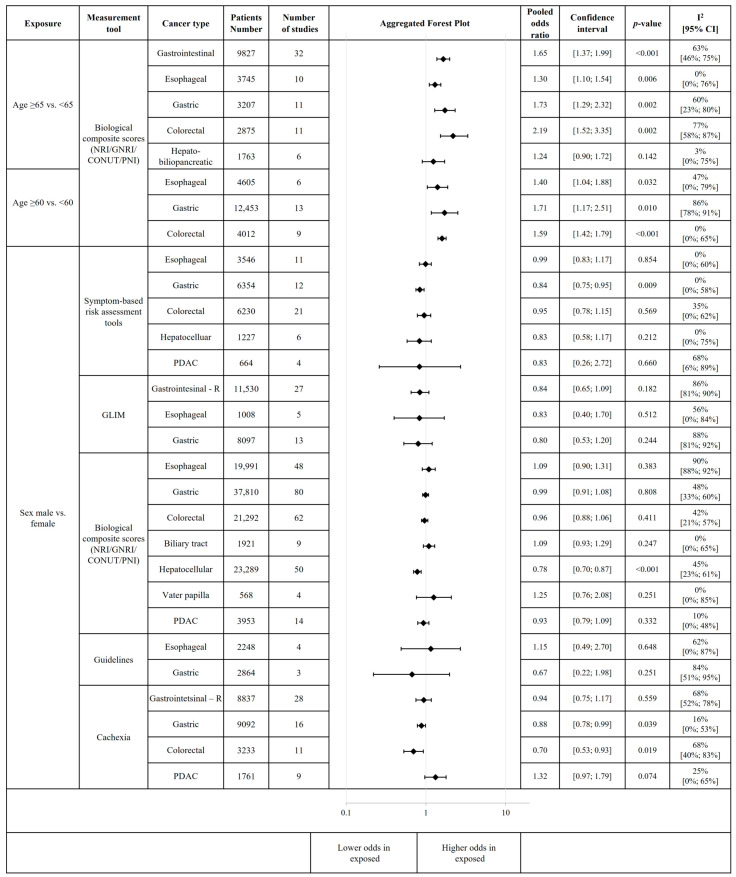
Association of population characteristics with malnutrition-related complication risk, malnutrition risk, diagnosis of malnutrition, and cachexia. This figure includes aggregated forest plots of individual meta-analyses. Each subgroup analysis includes individual, non-overlapping populations. For certain risk factors, we could not perform subgroup analysis by cancer type (gastrointestinal and hepato-biliopancreatic cancers, respectively, were analysed together). Abbreviations: R = resectable cancer stage; PDAC = pancreatic ductal adenocarcinoma; GLIM = Global Leadership Initiative on Malnutrition; GNRI = Geriatric Nutritional Risk Index, PNI = Prognostic Nutritional Index; CONUT = Controlling Nutritional Status; NRI = Nutritional Risk Index; Guidelines: Weimann et al., 2006: ESPEN guidelines on enteral nutrition: surgery including organ transplantation [45]; Cederholm et al., 2015: Diagnostic criteria for malnutrition—an ESPEN consensus statement [46]; Cachexia—definition varied across the eligible articles; however, in most cases the Fearon criteria were used (for definition see Supplementary Document S3).

**Figure 3:**
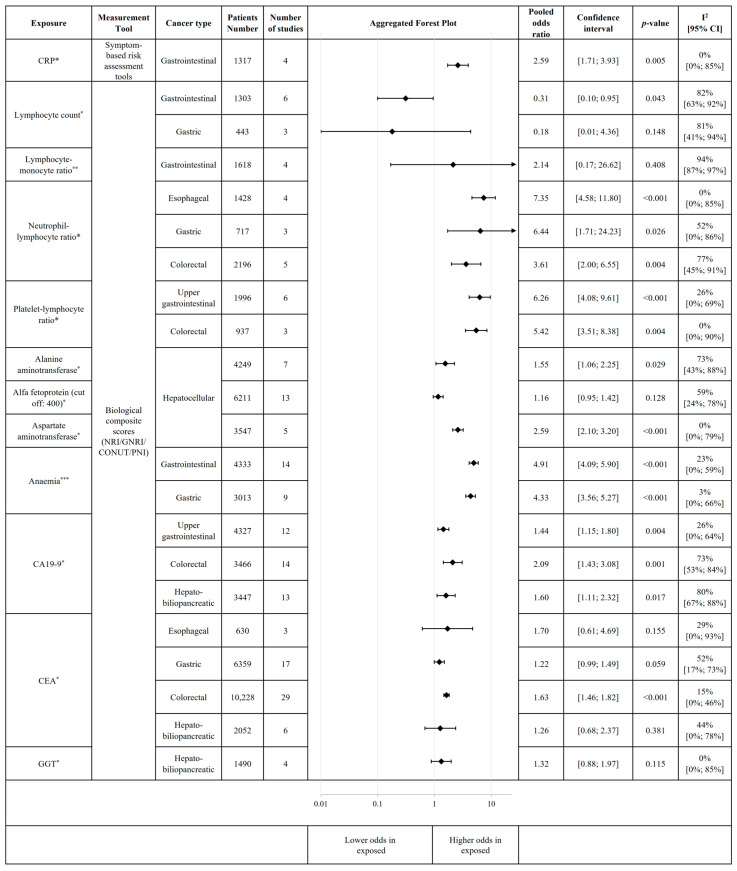
Association of inflammation and other biological parameters with the risk of malnutrition and malnutrition-related complications. * increased vs. decreased; ** decreased vs. increased; *** presence vs. absence; an arrow indicates that the upper confidence interval exceeds the value of 10. This figure includes aggregated forest plots of individual meta-analyses. Each subgroup analysis includes individual, non-overlapping populations. For certain risk factors, we could not perform subgroup analysis by cancer type (gastrointestinal and hepato-biliopancreatic cancers, respectively, were analysed together). Abbreviations: GNRI = Geriatric Nutritional Risk Index, PNI = Prognostic Nutritional Index; CONUT = Controlling Nutritional Status; NRI = Nutritional Risk Index; CRP = C-reactive protein; CA19-9 = Carbohydrate antigen 19-9; CEA = carcinoembryonic antigen; GGT = gamma-glutamyl transferase.

**Figure 4:**
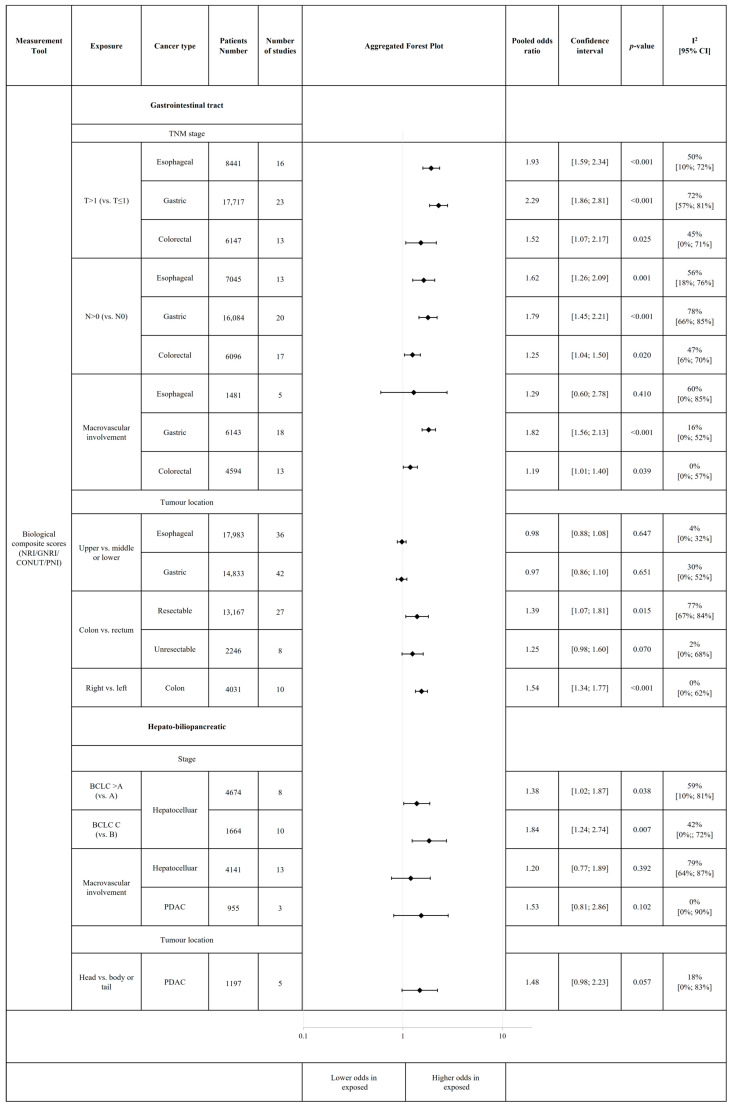
Association of tumour characteristics with malnutrition-related complications. This figure includes aggregated forest plots of individual meta-analyses. Each subgroup analysis includes individual, non-overlapping populations. For certain risk factors, we could not perform subgroup analysis by cancer type (gastrointestinal and hepato-biliopancreatic cancers, respectively, were analysed together). Abbreviations: PDAC = pancreatic ductal adenocarcinoma; GNRI = Geriatric Nutritional Risk Index, PNI = Prognostic Nutritional Index; CONUT = Controlling Nutritional Status; NRI = Nutritional Risk Index; BCLC = Barcelona Clinic Liver Cancer; T = T stage; N = N stage.

**Figure 5:**
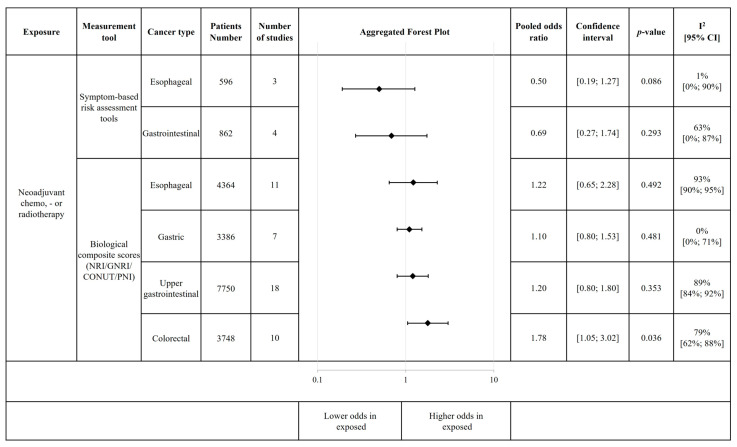
Association of neoadjuvant chemo and/or radiotherapy with risk of malnutrition and malnutrition-related complication risk. This figure includes aggregated forest plots of individual meta-analyses. Each subgroup analysis includes individual, non-overlapping populations. For certain risk factors, we could not perform subgroup analysis by cancer type (gastrointestinal and hepato-biliopancreatic cancers, respectively, were analysed together). Abbreviations: GNRI = Geriatric Nutritional Risk Index; PNI = Prognostic Nutritional Index; CONUT = Controlling Nutritional Status; NRI = Nutritional Risk Index.

Below, we present the detailed results of the evaluated subgroups. All the significant results we obtained suggest an association between the evaluated factor and malnutrition risk, malnutrition diagnosis, or cachexia, not implying causality. Besides the subgroup analyses mentioned in the protocol (CRD42022369200), we also performed moderator analysis for several of the subgrouping factors (resectability of the cancer, type of malnutrition risk screening tools, type of biological composite scores, definition of cachexia, age, and changes in TNM stage classification according to the different TNM editions). Notably, we have visualised our main results as aggregated forest plots (Figure 2, Figure 3, Figure 4 and Figure 5). Each subgroup analysis includes individual, non-overlapping populations. For certain risk factors, we could not perform subgroup analysis by cancer type (gastrointestinal and hepato-biliopancreatic cancers, respectively, were analysed together).

### 3.3. Association of Population Characteristics with Malnutrition-Related Complication Risk, Malnutrition Risk, Diagnosis of Malnutrition, and Cachexia

Age was mainly reported in association with the elevated malnutrition-related complications risk measured by biological composite scores, where older patients tended to have higher odds of it. We performed meta-analyses for several age cut-offs. The odds of positive malnutrition-related complication risk were increased in esophageal, gastric and colorectal (CRC) cancer patients both ≥60 years [esophageal—OR 1.40, 95% CI 1.04–1.88 (*p* = 0.032); gastric—OR 1.71, 95% CI 1.17–2.51 (*p* = 0.010); CRC—OR 1.59, 95% CI 1.42–1.79 (*p* < 0.001)] and ≥65 years [esophageal—OR 1.30, 95% CI 1.10–1.54 (*p* = 0.006); and gastric—OR 1.73, 95% CI 1.29–2.32 (*p* = 0.002); CRC—OR 2.19, 95% CI 1.42–3.35 (*p* = 0.002)] (Supplementary Document S10, Figures S1–S8). Moderator analysis based on age revealed increased malnutrition-related complication risk in ≥65 years in resectable and nonresectable stages and when evaluated with PNI in gastrointestinal cancer (Supplementary Document S14, Figures S127 and S128).

Regarding sex distribution, the results were mixed. In general, in GI cancers, male patients mainly had lower odds of malnutrition risk or being cachectic. However, in pancreatic ductal adenocarcinoma (PDAC), the odds of cachexia tended to be higher in males, but without a statistically significant difference [OR 1.32, 95% CI 0.97–1.79 (*p* = 0.074)]. Regarding the malnutrition-related complication risk based on biological composite scores, it revealed male sex as a protective factor in hepatocellular carcinoma (HCC) [OR 0.78, 95% CI 0.70–0.87 (*p* < 0.001)], but tended to be a risk factor for tumours of the ampulla of Vater [OR 1.25, 95% CI 0.76–2.08 (*p* = 0.251)]. In PDAC, sex did not influence the risk for malnutrition-related complication [OR 0.93, 95% CI 0.79–1.09 (*p* = 0.332)]; it also was not revealed as a risk factor being at malnutrition risk, as measured by symptom-based risk assessment tools [OR 0.83, 95% CI 0.26–2.72 (*p* = 0.660)] (Figure 2 and Supplementary Document S10, Figures S9–S29).

Meta-regression did not show a significant correlation between the proportion of females and malnutrition risk in colorectal cancer (Supplementary Document S14, Figure S130) or a significant correlation between the proportion of females and malnutrition-related complication risk in esophageal, gastric, and resectable hepatocellular carcinoma (Supplementary Document S14, Figures S131–S133). Similarly, no significant association between cachexia and sex was observed in gastrointestinal cancer (Supplementary Document S14, Figure S134), and when stratified by cancer types or definition of cachexia (Supplementary Document S14, Figures S135 and S136). Moderator analyses found increased malnutrition-related complication risk in esophageal cancer in resectable stages in males (Supplementary Document S14, Figure S137). However, no significant association was found between sex and malnutrition-related complication risk across different esophageal cancer types and assessment tool types (Supplementary Document S14, Figures S138 and S139). Similar results were found in colorectal and gastric cancer (Supplementary Document S14, Figures S140–S145). In resectable hepatocellular carcinoma, moderator analysis showed increased malnutrition-related complication risk in females, aged >55 years, and when assessed using CONUT or PNI (Supplementary Document S14, Figures S146 and S147).

Analysis of smoking and current or previous alcohol consumption found no significant association with malnutrition assessment, regardless of cancer type and assessment tool type. In hepato-biliopancreatic cancer, however, there was a slight tendency for increased odds of malnutrition-related complication risk, as evaluated by biological composite scores (Supplementary Document S10, Figures S30–S46).

The presence of comorbidities increases the odds for malnutrition-related complication risk in upper GI cancer [OR 1.23, 95% CI 1.01–1.49 (*p* = 0.039)], and especially in esophageal cancer [OR 1.37, 95% CI 1.08–1.73 (*p* = 0.024)]. We only found a significant association between chronic kidney disease (CKD) and malnutrition-related complication risk in patients with GI tract cancer [OR 2.94, 95% CI 1.82–4.77 (*p* = 0.002)—biological composite scores], while for cardiovascular disease, diabetes mellitus, and chronic respiratory diseases, the results were not significant, neither for malnutrition risk, nor malnutrition-related complication risk, or for malnutrition diagnosis or cachexia. Notably, diabetes mellitus appears to have no effect on being at risk of malnutrition [OR 0.79, 95% CI 0.49–1.27 (*p* = 0.426)—symptom-based risk assessment tools] in HBP patients; however, for PDAC patients, diabetes mellitus seems to increase the odds of cachexia [OR 1.86, 95% CI 0.81–4.27 (*p* = 0.096)] (Supplementary Document S10, Figures S47–S63).

An American Society of Anesthesiologists Physical Status (ASA) score ≥ 3 was significantly associated with malnutrition risk, measured by symptom-based risk assessment tools [OR 1.97, 95% CI 1.45–2.68 (*p* = 0.001)] in colorectal cancer, and for the diagnosis of malnutrition [OR 1.40, 95% CI 1.13–1.72 (*p* = 0.006)] in gastrointestinal cancer (Supplementary Document S10, Figures S64 and S65). Similar results were seen for malnutrition-related complication risk based on biological composite scores for esophageal [OR 2.27, 95% CI 1.33–3.85 (*p* = 0.013)], for gastric [OR 1.64, 95% CI 1.02–2.64 (*p* = 0.043), and for colorectal cancer [OR 1.82, 95% CI 1.43–2.31 (*p* < 0.001)] (Supplementary Document S10, Figures S66–S68).

ECOG (Eastern Cooperative Oncology Group) status was also reported in association with malnutrition-related complication risk, with increased odds in all cancer types starting at ECOG 2 and above compared with ECOG 0–1 (Supplementary Document S10, Figures S71–S74). Notably, in hepato-biliopancreatic cancer, the cachexia risk was increased, although it was not statistically significant [OR 1.70, 95% CI 0.51–5.68 (*p* = 0.256)], even in patients with good performance status, ECOG 1 vs. 0 (Supplementary Document S10, Figure S75).

The individual plots for population characteristics are detailed in the Supplementary Document S10, Figures S1–S75.

### 3.4. Association of Inflammation and Other Biological Parameters with the Risk of Malnutrition and Malnutrition-Related Complications

Several inflammatory biomarkers may indicate malnutrition risk if increased at baseline, such as C-reactive protein (CRP) [OR 2.59, 95% CI 1.71–3.93 (*p* = 0.005)], or indicate malnutrition-related complication risk like neutrophil-to-lymphocyte ratio [esophageal—OR 7.35, 95% CI 4.58–11.80 (*p* < 0.001); gastric—OR 6.44, 95% CI 1.71–24.23 (*p* = 0.026); and CRC—OR 3.61, 95% CI 2.00–6.55 (*p* = 0.004)]. Inflammation significantly increased the malnutrition-related complication risk, regardless of cancer type. Anaemia was another strong predictor for malnutrition-related complication risk in GI tract cancers [OR 4.91, 95% CI 4.09–5.90 (*p* < 0.001)] (Supplementary Document S11, Figure S88). Higher malnutrition-related complication risk was associated with elevated levels of gamma-glutamyl transferase (GGT) [OR 1.32, 95% CI 0.88–1.97 (*p* = 0.115)], and alanine-aminotransferase (ALT) [OR 1.55, 95% CI 1.06–2.25 (*p* = 0.029)] in hepato-biliopancreatic cancers (Supplementary Document S11, Figures S85 and S96). High tumour marker levels also indicated an increased malnutrition-related complication risk. Significant results were obtained for colorectal cancer with increased (vs. decreased) levels of carcinoembryonic antigen (CEA) [OR 1.63, 95% CI 1.46–1.82 (*p* ≤ 0.001)] and carbohydrate antigen 19-9 (CA19-9) [OR 2.09; 95% CI 1.43–3.08 (*p* = 0.001)]. Increased CA19-9 levels were associated with higher odds for malnutrition-related complication risk in upper GI cancer [OR 1.44, 95% CI 1.15–1.80 (*p* = 0.004)], and in HBP cancer [OR 1.60, 95% CI 1.11–2.32 (*p* = 0.017)] (Figure 3). The individual plots for inflammatory and other biochemical parameters are presented in Supplementary Document S11, Figures S76–S96. Moderator analysis of colorectal cancer showed that elevated serum CA19-9 levels were associated with malnutrition-related complication risk, particularly in resectable disease stages and when assessed using the PNI. However, no significant association was observed between elevated CA19-9 and malnutrition-related complication risk when stratified by colorectal cancer subtype or female proportion (Supplementary Document S14, Figures S148–S151).

### 3.5. Association of Tumour Characteristics and Treatment with Risk of Malnutrition and Malnutrition-Related Complications

Regarding tumour characteristics, there were remarkable differences between upper and lower GI tract cancers. While in esophageal and gastric cancers, the malnutrition-related complication risk is increased in early stages (T0–1, N0 vs. above), in colorectal cancer, significant differences occur mainly in later stages (T4 vs. T0–3) [OR 1.69, 95% CI 1.35–2.11 (*p* < 0.001)]. Macrovascular invasion made no difference in terms of malnutrition-related complication risk in esophageal cancer; however, it significantly increased the risk in gastric cancer [OR 1.82, 95% CI 1.56–2.13 (*p* < 0.001)] and in CRC [OR 1.19, 95% CI 1.01–1.40 (*p* = 0.039)]. On the other hand, we found no evidence that the location of the tumour in UGI tract cancers influenced the malnutrition-related complication risk (upper vs. mid or low oesophagus or stomach). Nevertheless, malnutrition-related complication risk was significantly higher in colon cancer by comparison with resectable rectal cancer [OR 1.39, 95% CI 1.07–1.81 (*p* = 0.015)], and in the right versus left colon [OR 1.54, 95% CI 1.34–1.77 (*p* < 0.001)]. In hepato-biliopancreatic cancers, macrovascular invasion was associated with a tendency for increased malnutrition-related complication risk. In HCC, the malnutrition-related complication risk also increased with Barcelona Clinic Liver Cancer criteria (BCLC) stage [BCLC C vs. B—OR 1.84, 95% CI 1.24–2.74 (*p* = 0.07)]; however, we found no evidence for it to be influenced by the aetiology of the disease [viral vs. non-viral—OR 1.16, 95% CI 1.00–1.34 (*p* = 0.047)]. For PDAC, the odds of malnutrition-related complication risk were overall higher in the head than in body or tail tumours [OR 1.48, 95% CI 0.98–2.23 (*p* = 0.057)] (Figure 4). The individual plots are available in the Supplementary Document S12, Figures S97–S121. Moderator analysis revealed further significant associations in cases of gastric and esophageal cancer between malnutrition-related complication risk and more advanced stages, when measured with PNI and according to the earlier TNM staging system edition (Supplementary Document S14, Figures S152–S169).

Regarding treatment in all cancer types, there is a clear tendency for increased malnutrition-related complication risk as measured by biological composite scores after neoadjuvant treatment. Remarkably, in esophageal cancer, if malnutrition screening was performed with symptom-based tools, the risk tends to be lower in patients receiving neoadjuvant therapy (Figure 5), although the results were not statistically significant. The individual plots are in Supplementary Document S13, Figures S122–S125. All malnutrition assessments were conducted after the neoadjuvant therapy and before surgery.

All moderator analysis results are presented in Supplementary Document S14, Figures S126–S172.

All significant results are summarised in Table 2 to better emphasise the potential risk factors for malnutrition diagnosis, being at risk of malnutrition, for malnutrition-related complications, and for cachexia based on cancer type.

Individual forest plots are found for population characteristics in Supplementary Document S10, Figures S1–S8, S10, S21, S27, S28, S47, S61, S64–S68, S70, S71, S73, and S74; for biological parameters in Supplementary Document S11, Figures S76, S78, S80–S85, S87–S91, and S94; for tumour characteristics in Supplementary Document S12, Figures S97–S103, S105, S111, S112, S118, and S119; and for therapy in Supplementary Document S13 Figure S125.

### 3.6. Risk of Bias Assessment and Sensitivity Analysis

The overall results for risk of bias assessment are available in Supplementary Table S3. The risk of bias for study participation, attrition, prognostic factor measurement, outcome measurement, study confounding, and statistical analysis reporting was generally low. Bias arising from study participation was mainly attributed to the heterogeneity of cancer stages among the included patients. Bias due to confounding was judged as moderate to high in several studies, largely because key confounders, such as age, cancer stage, comorbidities, and treatment status, were not consistently reported or appropriately adjusted for.

For some of the analyses, the amount of available articles has allowed for publication bias assessment. We could analyse the risk for publication bias in several subgroups. We found a potential risk for small study effect in case of malnutrition-related complication risk, measured by biological composite scores, for the following factors: hypertension in gastrointestinal cancer [Egger test: t = −2.06, df = 15, *p*-value = 0.0568], N stage for gastric cancer [for N0 vs. N ≥ 1—Egger test: t = −2.32, df = 18, *p*-value = 0.0320], T stage in esophageal [for T > 1 vs. T ≤ 1—Egger test: t = 4.44, df = 14, *p*-value = 0.0006], and gastric cancer [for T > 1 vs. T ≤ 1—Egger test: t = −2.51, df = 21, *p*-value = 0.0204; for T4 vs. T < 4—Egger test: t = −2.07, df = 20, *p*-value = 0.0520]. Additionally, we found a potential risk for small study effect in the case of malnutrition risk, measured by symptom-based risk assessment tools for the factor sex in esophageal cancer [Egger test: t = −2.67, df = 9, *p*-value = 0.0254]. The detailed results are found in the Supplementary Material in Supplementary Document S15, and the figures are found in Supplementary Document S16.

The detailed leave-one-out sensitivity analysis results are available in Supplementary Document S6. It revealed only four outliers—respectively, F.K. Xiao, 2021 [47] in the esophageal cancer subgroup when comparing male to female patients, C. Akgul, 2024 [48] in colorectal subgroup for macrovascular involvement exposure, A. Hiraoka, 2023 [49] in hepatocellular carcinoma subgroup for aetiology (viral vs. non-viral) exposure, and Y. Sun, 2025 [50] for esophageal cancer subgroup for the age (≥65 vs. <65) exposure. By excluding these, the decision on the null hypothesis would be changed, demonstrating the limited robustness of these results. Figures for leave-one-out analysis are found in Supplementary Document S16. The heterogeneity, in general, was high.

Although a GRADE assessment [51] was not performed, the overall certainty of evidence is likely low due to potential publication bias and the high heterogeneity across cancer types, stages, and assessment tools.

## 4. Discussion

We investigated the risk factors for malnutrition risk, malnutrition diagnosis, malnutrition-related complication risk, and cachexia in gastrointestinal cancer patients. We found age ≥ 60 to be associated with malnutrition-related complication risk and female sex to be associated with cachexia in GI cancer patients. Poor performance status (ECOG ≥ 2) indicated a higher malnutrition-related complication risk in all GI cancer patients. In HBP tumours, cachexia was increased starting at ECOG 1. Tumour location was associated with malnutrition-related complication risk in PDAC (higher in head vs. in body or tail) and CRC (higher in right vs. left-sided). Symptom-based risk assessment tools and biological composite scores results tended to be discordant after neoadjuvant therapy in esophageal cancer.

In gastrointestinal cancer, the malnutrition-related complication risk is associated with a younger age (cut-off of 60 years). Dysphagia and loss of appetite, which are among the most frequent symptoms of gastrointestinal cancer, drastically decrease oral intake, leading to a rapid deterioration of the nutritional status, also in patients who are younger and fitter. Our results underline the importance of malnutrition assessment, especially in patients with good clinical status. The odds of cachexia, malnutrition risk, malnutrition diagnosis, and malnutrition-related complication risk were increased in female sex patients with GI tract cancer. Loss of skeletal muscle mass defines the diagnosis of malnutrition based on the GLIM criteria [18,19]. Since, in most cases, the mean age at cancer diagnosis was above 50, this may be related to the post-menopausal status when the reduced levels of sex hormones, like estrone and oestrogen, are associated with the loss of skeletal muscle mass [52,53].

Except for patients with HBP cancer, in which there was a slight tendency for increased odds for malnutrition-related complication risk in association with smoking and alcohol consumption, these were not significantly associated, regardless of cancer type and stage. Alcohol may have a high energy content, and chronic consumption may be associated with elevated BMI and hyperlipidaemia [54]. These parameters are quantified in most biological composite scores, like GNRI, CONUT, or NRI. Also, symptom-based risk assessment tools showed similar results. In these patients, body composition measurement would probably more accurately reflect the nutritional status [55].

Data about comorbidities were scarce and heterogeneous. Overall, the presence of comorbidities showed statistically significant associations with malnutrition-related complication risk in upper GI cancer. However, the only significant correlation for a specific disease for malnutrition-related complication risk was found in gastrointestinal cancer patients with CKD. Chronic kidney disease may be associated with chronic inflammation, which may aggravate the cancer-induced inflammatory status and often low serum albumin levels due to urinary loss [56]. The absence of significant associations between certain comorbidities and malnutrition risk should be interpreted with caution. Many studies incompletely characterised comorbid conditions, which may have introduced confounding.

The strong association of malnutrition-related complication risk with high ASA scores (≥3) emphasises the cumulative effect of comorbidities, which will be addressed early during the disease course [57,58].

Inflammation plays an important role in cancer-induced cachexia [59], and despite substantial research effort, effective therapeutic approaches remain limited. Different management strategies emphasise approaches combining nutritional support and pharmacological interventions targeting appetite regulation; however, they have limited efficacy [60]. Different inflammatory biomarkers could predict clinical outcomes in patients with cancer [61]. The modified Glasgow Prognostic score leverages the synergy between elevated CRP level and low albumin levels to predict malnutrition and increased values are associated with prognosis in colorectal and gastric cancer; however, due to the limited amount of data, we could not include it in our analysis [62].

Anaemia was strongly associated with malnutrition-related complication risk in GI cancer patients. This association may be multifactorial, caused by chronic inflammation induced by the tumour itself, by bleeding, iron malabsorption, and the alteration of erythropoiesis [63]. Additionally, rapidly growing tumours outpace the tissue’s blood supply, resulting in hypoxia. This phenomenon leads to the decline in protection against oxidative stress, which accelerates the growth of tumours and causes malignant progression [5].

In all cases, increased levels of tumour markers were associated with an increased odds for malnutrition-related complication risk. AFP level may be increased in hepatocellular carcinoma and may deprive the proliferation of immune cells, like T-cells and Natural Killer cells, contributing to a declined immune response, tumour growth, and disease progression [64]. Elevated CA19-9 and CEA were reported in both GI and HBP cancers for higher odds for malnutrition-related complication risk, which also reflects their lack of specificity [65,66,67].

For esophageal and gastric cancers, the odds of malnutrition-related complication risk are increased starting at T, N ≥ 1, while in colorectal cancer, significant differences occurred in more advanced stages. Colorectal cancer is frequently curable if detected early; the mechanism of delayed malnutrition development, mainly in more advanced stages, shall be further investigated [68].

We explored tumour site as a risk factor for malnutrition-related complication risk, and found no association in gastric or esophageal cancer (proximal vs. distal tumour location). The heterogeneity of cancer stages and treatment may influence the overall result. Interestingly, patients with right-sided CRC had higher odds of malnutrition-related complication risk. Molecular classification of CRC affects the tumour’s microenvironment. Tumours with microsatellite instability (MSI) are more immunogenic and may be associated with higher levels of inflammatory markers than mismatched repair-proficient tumours [69]. This could explain the differences since MSI high tumours are more frequently right-sided [70]. Furthermore, in pancreatic cancer, proximal tumour location (vs. distal) increased the odds of malnutrition-related complication risk. Biliary obstruction, exocrine insufficiency, and gastric outlet obstruction may be contributing factors [71].

The discordant results regarding malnutrition after neoadjuvant therapy in patients with esophageal cancer highlight the distinct biological and clinical domains each tool captures. In patients undergoing chemo- and/or radiotherapy, changes, observed in biological composite scores, are likely influenced by treatment-induced systemic inflammation rather than reflecting nutritional risk per se. Consequently, these scores may lose some of their fidelity in predicting malnutrition-related complications in this setting. In contrast, symptom-based nutritional risk assessment tools appear to remain more reliable, which may help explain why our meta-analysis revealed discrepant results between biomarker-based and symptom-based assessment methods. Although data on malnutrition risk after chemotherapy and radiotherapy were relatively scarce, this result suggests that symptom-based scores may be more suitable in this clinical scenario and may provide a more accurate reflection of underlying nutritional status. These findings underscore the importance of context-specific tool selection.

Although CRP levels provide valuable information, our findings suggest that effective personalised malnutrition assessment requires the integration of cancer-specific factors. In esophageal cancer, advanced age (>60 years), presence of comorbidities, and tumour stage T/N ≥ 1 were associated with higher malnutrition-related complication risk, while the treatment-induced systemic inflammation effect of neoadjuvant therapy might influence the accuracy of biological composite scores for malnutrition assessment. In hepato-biliopancreatic cancers, female sex and alcohol consumption emerged as relevant risk factors. Specifically for pancreatic cancer, proximal tumour location increases malnutrition-related complication risk, while in colorectal cancer, both advanced age (>60 years), female sex, proximal tumour location, and T4 cancer stage warrant consideration.

### 4.1. Strengths and Limitations

To date, this is the most comprehensive systematic review and meta-analysis to examine risk factors for malnutrition risk, malnutrition diagnosis, malnutrition-related complication risk, and cachexia in digestive system cancers. Its novelty lies in the broad inclusion criteria, covering gastrointestinal and hepato-biliopancreatic cancer subtypes, and the structured classification of nutritional assessment, which encompasses symptom-based screening tools, biological composite scores, GLIM criteria-based malnutrition diagnosis, and cachexia. In addition, the robust methodological approach, including extensive subgroup and moderator analyses, enabled the identification of cancer-specific risk factors and the evaluation of assessment tools that may be more suitable for particular clinical scenarios, with immediate clinical applicability.

The main limitation of our meta-analysis was the high heterogeneity observed across many of the pooled analyses. We could only partially address this with subgroup analysis based on cancer type and stage, and assessment tool types. Subgroup analysis was decided on clinical rationale and performed based on the availability of the data. While we anticipated these main categories of subgroups (CRD42022369200), the decision on the specific calculations could only be made after extracting the data. Different cancer types may present distinct symptom profiles that could affect the efficacy of assessment tools. Varying stages of disease may also have an influence. Also, the different assessment tool types (biological composite scores, symptom-based) can provide distinct results. Therefore, it was essential to conduct moderator analysis to identify the source of heterogeneity. While subgroup and moderator analyses were performed to explore potential sources of heterogeneity, these methods only partially accounted for the observed variability. Therefore, the pooled estimates should be interpreted with caution and are unlikely to be directly generalizable to any single patient population. Nevertheless, it also emphasises the need for a consensus on how to select appropriate assessment tools in specific clinical settings.

Given the large number of subgroups and moderator analyses performed, the risk of Type I error is elevated. As no correction for multiple comparisons was applied, some statistically significant findings should be interpreted with caution.

Another limitation is the risk of bias we identified for some of the analyses, which results mainly from the retrospective nature of many included studies. Also, for some of the evaluated outcomes, like hypertension in gastrointestinal cancer, N stage (N0 vs. N ≥ 1) in gastric cancer, T stage (T > 1 vs. T ≤ 1 and T4 vs. T < 4) in gastric and esophageal cancer, and sex, as a risk factor in esophageal cancer, we identified potential publication bias. In gastric cancer, studies assessing N stage (N0 vs. N ≥ 1) demonstrated that larger studies tended to report higher odds ratios, while smaller studies with lower or nonsignificant effects appear to be underrepresented. For the risk factor of hypertension in gastrointestinal cancers, smaller studies reporting higher odds were absent. For sex in esophageal cancer, smaller studies reporting higher odds were missing. In the analyses of T stage (T > 1 vs. T ≤ 1 and T4 vs. T < 4) in both esophageal and gastric cancers, small-sample studies were largely absent, and those reporting lower odds were underrepresented. This likely led to overestimation of effect sizes, suggesting that the true associations may be smaller or even absent.

Furthermore, sensitivity analyses demonstrated that some findings, including sex as a risk factor in esophageal cancer, macrovascular involvement in colorectal cancer, aetiology (viral vs. non-viral) in hepatocellular carcinoma, and age ≥ 65 in esophageal cancer, were highly influenced by individual studies. These results were not robust and highlight the need for cautious interpretation, as they may reflect statistical fragility rather than definitive associations.

Although we did not perform a GRADE [51] assessment for our analysis, taking into account the potential for publication bias, the high heterogeneity related to cancer types, stages, and assessment tools, the certainty of our findings is likely low according to GRADE domains (risk of bias, inconsistency, indirectness, imprecision, and publication bias). As such, the associations reported in this review should be interpreted with caution.

### 4.2. Implications for Practice

The findings of this meta-analysis may help guide the design of future prospective studies aimed at developing and validating malnutrition assessment protocols in gastrointestinal cancers, which can be applicable in clinical practice [72,73]. Our results suggest that nutritional screening should be performed at the time of cancer diagnosis, particularly in patients aged ≥60 years, in females, and in those with preserved performance status, as these groups may still carry a substantial risk of malnutrition-related complications. Routinely measured parameters, such as C-reactive protein, could serve as an early indicator of increased malnutrition risk and should be interpreted in conjunction with symptom-based nutritional screening tools in routine clinical practice. In addition, tumour location, including right-sided colorectal cancer and pancreatic head tumours, may indicate a higher malnutrition-related complication risk even at an early stage of disease.

Although our study did not aim to evaluate the diagnostic accuracy of different screening tools for various cancer types, our findings further indicate that the choice of nutritional assessment tool should be adapted to the clinical context. Symptom-based risk assessment tools appear particularly useful in patients undergoing anticancer treatment or presenting with gastrointestinal symptoms, whereas biological composite scores may be more informative in asymptomatic patients. These findings highlight the limited applicability of certain assessment tools in specific clinical scenarios and underscore the need for a personalised approach to nutritional assessment in patients with gastrointestinal cancer.

### 4.3. Implications for Research

There is a strong need for consensus regarding the definition of cachexia and the role of different types of malnutrition assessment tools. The association between tumour location and malnutrition-related complication risk in colorectal and pancreatic cancer may be related to molecular tumour characteristics. Prospective observational clinical trials are needed to investigate associations between inflammatory parameters (e.g., TNF alpha and interleukins) and malnutrition. In addition, studies on inflammation-decreasing targeted treatments for cancer cachexia are needed.

## 5. Conclusions

Our findings confirm established risk factors for malnutrition in gastrointestinal cancer, like age, cancer stage, and inflammation, while revealing site-specific differences, notably in colorectal and pancreatic cancers. The observed divergence between symptom-based screening tools and biological composite scores following neoadjuvant therapy in esophageal cancer raises concerns regarding tool validity and comparability, underscoring the need for standardised, harmonised approaches in future research and clinical implications.

## Figures and Tables

**Table 1:** Subgroup analysis.

Subgroups		
Cancer type	Gastrointestinal tract	Esophageal
		Gastric
		Colorectal
	Upper gastrointestinal tract	Esophageal
		Gastric
	Hepato-biliopancreatic	Biliary tract
		Hepatocellular
		Pancreatic ductal adenocarcinoma
Measurement tools	Malnutrition risk screening based on symptoms and signs	NRS-2002 [10]
		MUST [11]
		MNA [12]
		MNA-SF [13]
		MST [14]
		SGA [15]
		PG-SGA [16]
		SNAQ [17]
	Tools evaluating the risk of malnutrition-related complications	GNRI [6]
		PNI [7]
		CONUT [8]
		NRI [9]
	Diagnosis of malnutrition	GLIM criteria [18]
		Previous guidelines *
	Cachexia **	

Abbreviations: GLIM = Global Leadership Initiative on Malnutrition; GNRI = Geriatric Nutritional Risk Index, PNI = Prognostic Nutritional Index; CONUT = Controlling Nutritional Status; NRI = Nutritional Risk Index; NRS-2002 = Nutritional Risk Screening 2002; MUST = Malnutrition Universal Screening Tool; MNA = Mini Nutritional Assessment; MNA-SF = Mini Nutritional Assessment ShortForm; SGA = Subjective Global Assessment; PG-SGA = Patient-Generated Subjective Global Assessment; SNAQ = Short Nutritional Assessment Questionnaire; * Guidelines: Weimann et al., 2006: ESPEN guidelines on enteral nutrition: surgery including organ transplantation [45]; Cederholm et al., 2015: Diagnostic criteria for malnutrition—an ESPEN consensus statement [46]; ** Cachexia: definition varied across the eligible articles; however, in most cases the Fearon criteria were used (for definition see Supplementary Document S3).

**Table 2:** Risk factors for malnutrition risk, malnutrition-related complication risk, malnutrition diagnosis, and cachexia based on cancer type.

Risk Factor Category	Measurement Tool	Confirmed Risk Factors for Malnutrition	Cancer Type
Population characteristics	Biological composite scores (NRI, GNRI, CONUT PNI)	Age ≥ 65	Gastrointestinal
			Esophageal
			Gastric
			Colorectal
		Age ≥ 60	Esophageal
			Gastric
			Colorectal
	Sign and symptom-based risk assessment tools	Female sex	Gastric
	Biological composite scores (NRI, GNRI, CONUT, PNI)		Hepatocellular
	Cachexia		Gastric
			Colorectal
	Biological composite scores (NRI, GNRI, CONUT, PNI)	Comorbidities	Upper gastrointestinal
	Biological composite scores (NRI, GNRI, CONUT, PNI)	Chronic kidney disease	Gastrointestinal
	Sign and symptom-based risk assessment tools	ASA score ≥ 3	Colorectal
	Malnutrition diagnosis according to GLIM criteria		Gastrointestinal
	Biological composite scores (NRI, GNRI, CONUT, PNI)		Esophageal
			Gastric
			Colorectal
	Malnutrition based on guidelines		Gastrointestinal
	Biological composite scores (NRI, GNRI, CONUT, PNI)	ECOG ≥ 2	HBP cancer
			Colorectal
			Gastric
Biological parameters	Biological composite scores (NRI, GNRI, CONUT, PNI)	Elevated CRP	Gastrointestinal
		Decreased Lymphocyte count	Gastrointestinal
		Elevated Neutrophil–Lymphocyte ratio	Esophageal
			Gastric
			Colorectal
		Elevated Platelet–Lymphocyte ratio	Upper gastrointestinal
			Colorectal
		Elevated Alanine-aminotransferase	Hepatocellular
		Elevated Aspartate-aminotransferase	Hepatocellular
		Anaemia	Gastrointestinal
			Gastric
		Elevated CA19-9	Upper gastrointestinal
			Colorectal
			HBP cancer
		Elevated CEA	Colorectal
Tumour characteristics	Biological composite scores (NRI, GNRI, CONUT, PNI)	T > 1 stage	Esophageal
			Gastric
		N > 0 stage	Esophageal
			Gastric
			Colorectal
		Macrovascular involvement	Gastric
			Colorectal
		Colon tumour location (vs. rectum)	Colorectal (resectable stage)
		Right tumour location (vs. left)	Colon cancer
		BCLC B or C (vs. A)	Hepatocellular
		BCLC C (vs. B)	
		Child–Pugh class B or C (vs. A)	
Therapy	Biological composite scores (NRI, GNRI, CONUT, PNI)	Neoadjuvant therapy	Colorectal

Abbreviations: GLIM = Global Leadership Initiative on Malnutrition; GNRI = Geriatric Nutritional Risk Index, PNI = Prognostic Nutritional Index; CONUT = Controlling Nutritional Status; NRI = Nutritional Risk Index; CRP = C-reactive protein; CA19-9 = Carbohydrate antigen 19-9; CEA = carcinoembryonic antigen T = T stage; N = N stage; BCLC = Barcelona Clinic Liver Cancer; ASA = American Society of Anesthesiologists Physical Status; ECOG = Eastern Cooperative Oncology Group; HBP = hepato-biliopancreatic, Guidelines: Weimann et al., 2006: ESPEN guidelines on enteral nutrition: surgery including organ transplantation [45]; Cederholm et al., 2015: Diagnostic criteria for malnutrition—an ESPEN consensus statement [46]; Cachexia—definition varied across the eligible articles; however, in most cases the Fearon criteria were used (for definition see Supplementary Document S3).

## Data Availability

The datasets used in this study can be found in the full-text articles that were included in the systematic review and meta-analysis.

## Supplementary Materials

The following supporting information can be downloaded at https://www.mdpi.com/article/10.3390/cancers18010080/s1, Supplementary Document S1: Detailed protocol deviation, Supplementary Document S2: Individualized search key in different databases, Supplementary Document S3: Detailed definition of the measurement tools, Supplementary Document S4: Detailed strategy for the selection process, Supplementary Document S5: Detailed strategy for data collection, Supplementary Document S6: Leave-one-out analysis, Supplementary Document S7: Detailed strategy for overlapping population, Supplementary Document S8: References of included studies, Supplementary Document S9: Detailed strategy for retrieval and reasons for excluding articles, Supplementary Document S10: Individual forest plots for population characteristics: Figures S1–S75, Supplementary Document S11: Individual forest plots for inflammation and other biological parameters: Figures S76–S96, Supplementary Document S12: Individual forest plots for tumour characteristics: Figures S97–S121, Supplementary Document S13: Individual forest plots for neoadjuvant therapy: Figures S122–S125, Supplementary Document S14: Moderator analysis: Figures S126–S172, Supplementary Document S15: Publication bias, Supplementary Document S16: Funnel plots for the publication bias and leave-one-out plots: Figures S173–S227, Supplementary Table S1: PRISMA 2020 Checklist and PRISMA 2020 for abstract Checklist, Supplementary Table S2: Study characteristics table, Supplementary Table S3: Summary plot for the risk of bias assessment.
