# Supplementary material for: Risk of Malnutrition in Digestive System Cancers: A Systematic Review and Meta-Analysis
Source: Cancers (Basel). 2025 Dec 26;18(1):80. doi: 10.3390/cancers18010080 (PMC12784894; doi:10.3390/cancers18010080)
Supplement: Supplementary file 1 [file cancers-18-00080-s001.zip › cancers-4072471-supplementary.pdf]

Supplementary Material: Risk of Malnutrition in Digestive System Cancers: a systematic review and meta-analysis

Bettina Csilla Budai[1,2,3], Petrana Martinekova[1,4], Gefu Cai[1], Dalma Dobszai[1,3], Lili Fekete[1,2], Hanne Aspelund Normann[1], Jázmin Németh[1], Alíz Fazekas[1,5], Eszter Ágnes Szalai[1,6], Andrea Szentesi[3], Vasile Liviu Drug [7], Péter Hegyi[1,2,3,8], Stefania Bunduc[1,3,9,10]

Affiliations:

1. Centre for Translational Medicine, Semmelweis University, 1085 Budapest, Hungary
2. Institute of Pancreatic Diseases, Semmelweis University,1085 Budapest, Hungary
3. Institute for Translational Medicine, Medical School, University of Pécs, 7624 Pécs, Hungary
4. Department of Hepatogastroenterology, Institute for Clinical and Experimental Medicine, 14021 Prague, Czech Republic
5. Department of Biophysics and Radiation Biology, Semmelweis University, 1085 Budapest, Hungary
6. Department of Restorative Dentistry and Endodontics, Semmelweis University, 1085 Budapest, Hungary
7. Gastroenterology Department, Grigore T. Popa University of Medicine and Pharmacy, Iași 700115, Romania
8. Translational Pancreatology Research Group, Interdisciplinary Centre of Excellence for Research Development and Innovation, University of Szeged, 6720 Szeged, Hungary
9. Carol Davila University of Medicine and Pharmacy, 050474 Bucharest, Romania
10. Digestive Diseases and Liver Transplantation Center, Fundeni Clinical Institute, 022328 Bucharest, Romania

Supplementary Document S1: Detailed protocol deviation 3
Supplementary Document S2: Individualised search key in different databases 3
Supplementary Document S3: Detailed definition of the measurement tools 4
Supplementary Document S4: Detailed strategy for the selection process 20
Supplementary Document S5: Detailed strategy for data collection 20
Supplementary Document S6: Leave-one-out analysis 20
Supplementary Document S7: Detailed strategy for overlapping population 21
Supplementary Document S8: References of the included studies 30
Supplementary Document S9: Detailed strategy for retrieval and reasons for excluding articles 74
Supplementary Document S10: Individual forest plots for population characteristics: Figure S1-S75 75
Supplementary Document S11: Individual forest plots for inflammation and other biological parameters: Figure S76-S96 127
Supplementary Document S12: Individual forest plots for tumour characteristics: Figure S97-S121 141
Supplementary Document S13: Individual forest plots for neoadjuvant therapy: Figures S122-S125 161
Supplementary Document S14: Moderator analysis: Figures S126-S172 165
Supplementary Document S15: Publication bias 210
Supplementary Document S16: Funnel plots for the publication bias and leave-one-out plots: Figures S173-S227 211
Supplementary Table S1.: PRISMA 2020 Checklist and PRISMA 2020 for Abstract Checklist 267
Supplementary Table S2.: Study characteristics table 271
Supplementary Table S3.: Summary plot of the risk of bias assessment 312

Supplementary Document S1: Detailed protocol deviation

We redo the search with a more comprehensive search key, including the following measurement tools for malnutrition: Geriatric Nutritional Risk Index (GNRI), Prognostic Nutritional Index (PNI), Controlling Nutritional Status (CONUT), Nutritional Risk Index (NRI).

Supplementary Document S2: Individualised search key in different databases

PubMed:
(("digestive system") OR ("gastrointestinal tract") OR (oesophag*) OR (esophag*) OR ("esogastric junction") OR (stomach) OR (gastric) OR (cardia) OR (antrum) OR (gut) OR (bowel) OR (duodenum) OR (ileum) OR (jejunum) OR (colon) OR (rectum) OR (appendix) OR (cecum) OR (cecal) OR (colorectal) OR (biliary) OR (gallblader) OR (cholecyst) OR (pancreas) OR (liver)) AND (("Neoplasms"[Mesh:NoExp]) OR (cancer) OR (malignancy) OR (tumor) OR (tumour) OR (carcinoma) OR (adenocarcinoma)) AND ((malnutrition) OR (malnourishment*) OR (undernutrition) OR ("fat free mass") OR ("FFM") OR ("fat mass") OR ("muscle mass") OR (cachexia) OR (cachec*) OR (non-cachectic) OR (pre-cachexia) OR ("nutritional deficiency") OR ("nutritional deficiencies") OR ("PG-SGA") OR ("skeletal muscle mass index") OR ("SMMI") OR ("Nutrition Risk Screening-2002") OR ("NRS-2002") OR ("Mini Nutritional Assessment") OR ("MNA") OR ("MNA-SF") OR ("Malnutrition Screening Tool") OR ("MST") OR ("Short Nutritional Assessment Questionnaire") OR ("SNAQ") OR ("Subjective Global Assessment") OR ("Malnutrition Universal Screening Tool") OR ("SGA") OR ("nutritional risk") OR (GLIM) OR ("geriatric nutritional risk index") OR ("gnri") OR ("prognostic nutritional index") OR ("pni") OR ("conut") OR ("controlling nutritional status") OR (nri) OR ("nutritional risk index" OR "creatinine height index"))

Embase:
('digestive system' OR 'gastrointestinal tract' OR oesophagus OR esophagus OR 'esogastric junction' OR stomach OR gastric OR cardia OR antrum OR gut OR bowel OR duodenum OR ileum OR jejunum OR colon OR rectum OR appendix OR cecum OR cecal OR colorectal OR biliary OR gallblader OR cholecyst OR pancreas OR liver) AND ('neoplasm' OR cancer OR malignancy OR tumor OR tumour OR carcinoma OR adenocarcinoma) AND (malnutrition OR malnourishment* OR undernutrition OR 'fat free mass' OR 'ffm' OR 'fat mass' OR 'muscle mass' OR cachexia OR cachec* OR 'non cachectic' OR 'pre cachexia' OR 'nutritional deficiency' OR 'nutritional deficiencies' OR 'pg-sga' OR 'skeletal muscle mass index' OR 'smmi' OR 'nutrition risk screening-2002' OR 'nrs-2002' OR 'mini nutritional assessment' OR 'mna' OR 'mna-sf' OR 'malnutrition screening tool' OR 'mst' OR 'short nutritional assessment questionnaire' OR 'snaq' OR 'subjective global assessment' OR 'sga' OR 'nutritional risk' OR 'malnutrition universal screening tool' OR 'GLIM' OR 'geriatric nutritional risk index' OR 'gnri' OR 'prognostic nutritional index' OR 'pni' OR 'conut' OR 'controlling nutritional status' OR 'nri' OR 'nutritional risk index' OR 'creatinine height index')

Central:
(('digestive system') OR ('gastrointestinal tract') OR (oesophag*) OR (esophag*) OR ('esogastric junction') OR (stomach) OR (gastric) OR (cardia) OR (antrum) OR (gut) OR (bowel) OR (duodenum) OR (ileum) OR (jejunum) OR (colon) OR (rectum) OR (appendix) OR (cecum) OR (cecal) OR (colorectal) OR (biliary) OR (gallblader) OR (cholecyst) OR (pancreas) OR (liver) AND ('Neoplasms') OR (cancer) OR (malignancy) OR (tumor) OR (tumour) OR (carcinoma) OR (adenocarcinoma)) AND ((malnutrition) OR (malnourishment*) OR (undernutrition) OR ("fat free mass") OR ("FFM") OR ("fat mass") OR ("muscle mass") OR (cachexia) OR (cachec*) OR (non-cachectic) OR (pre-cachexia) OR ("nutritional deficiency") OR ("nutritional deficiencies") OR ("PG-SGA") OR ("skeletal muscle mass index") OR ("SMMI") OR ("Nutrition Risk Screening-2002") OR ("NRS-2002") OR ("Mini Nutritional Assessment") OR ("MNA") OR ("MNA-SF") OR ("Malnutrition Screening Tool") OR ("MST") OR ("Short Nutritional Assessment Questionnaire") OR ("SNAQ") OR ("Subjective Global Assessment") OR ("SGA") OR ("Malnutrition Universal Screening Tool") OR ("nutritional risk") OR ("GLIM") OR ("geriatric nutritional risk index") OR ("gnri") OR ("prognostic nutritional index") OR ("pni") OR ("conut") ("controlling nutritional status") OR (nri) OR ("nutritional risk index") OR ("creatinine height index"))

Supplementary Document S3: Detailed definition of the measurement tools

I. Malnutrition Universal Screening Tool – MUST

BMI (kg/m^2)	Unintentional weight loss in the past 3–6 months	Acute illness with reduced food intake (estimated) for ≥5 days	
≥20 -0	≤5% -0	No = 0	Yes = 2
18.5–20.0 -1	5–10% -1		
≤18.5 -2	≥10% -2		
**Overall Risk for Malnutrition**			
0	Low		
1	Medium		
≥2	High		

II. Nutritional Risk Screening – NRS-2002

Impaired nutritional status		Severity of disease	
Absent<br>Score 0	normal nutritional status	Absent<br>Score 0	normal nutritional requirement
Mild<br>Score 1	weight loss >5% in 3 months<br>OR<br>Food intake below 50-70% of normal requirement in preceding week	Mild<br>Score 1	Hip fracture<br>Chronic patients, in particular with acute complications; cirrhosis; COPD; chronic hemodialysis, diabetes, oncology
Moderate<br>Score 2	weight loss >5% in 2 months<br>OR<br>BMI 18,5-20,5 kg/m^2 + impaired general condition<br>OR<br>Food intake 25-50% of normal requirement in preceding week	Moderate<br>Score 2	Major abdominal surgery; stroke; severe pneumonia; hematologic malignancy
Severe<br>Score 3	weight loss >5% in 1 months<br>OR<br>BMI 18,5 kg/m^2 + impaired general condition<br>OR<br>Food intake 0-25% of normal requirement in preceding week	Severe<br>Score 3	Head injury; bone marrow transplantation; intensive care patients
Total score:			
If age ≥70 years add 1 to total score:			
Score ≥3:	the patient is nutritionally at risk and a nutritional care plan is initiated		
Score <3:	weekly rescreening of the patient. If the patient e.g is scheduled for a major operation, a preventive nutritional care plan is considered to avoid the associated risk status.		

III. Mini Nutritional Assessment - MNA

Screening	
Has food intake declined over the past 3 months due to loss of appetite, digestive problems, or chewing or swallowing difficulties?	0 = severe decrease in food intake
	1 = moderate decrease in food intake
	2 = no decrease in food intake
Weight loss during the last 3 months	0 = weight loss greater than 3 kg
	1 = does not know
	2 = weight loss between 1 and 3 kg
	3 = no weight loss
Mobility	0 = bedridden or chair bound
	1 = able to get out of bed/chair but does not go out
	2 = goes out
Has the patient suffered psychological stress or acute disease in the past 3 months?	0 = yes
	2 = no
Neuropsychological problems	0 = severe dementia or depression
	1 = mild dementia
	2 = no psychological problems
Body mass index (BMI)	0 = BMI less than 19
	1 = BMI 19 to less than 21
	2 = BMI 21 to less than 23
	3 = BMI 23 or greater
**Screening Score**	
12–14 points	Normal nutritional status
8–11 points	At risk of malnutrition
0–7 points	Malnourished
**Assessment**	
Lives independently (not in nursing home or hospital)	0 = yes
	1 = no
Takes more than 3 prescription drugs per day	0 = yes
	1 = no
Pressure sores or skin ulcers	0 = yes
	1 = no
How many full meals does the patient eat daily?	0 = 1 meal
	1 = 2 meals
	2 = 3 meals
Selected consumption markers for protein intake	0.0 = if 0 or 1 yes
	0.5 = if 2 yes
	1.0 = if 3 yes
• Meat, fish or poultry every day Yes/No<br>• ≥1 serving of dairy products (milk, cheese, yoghurt) per day Yes/No<br>• ≥2 servings of legumes or eggs per week Yes/No	
Consumes ≥2 servings of fruit or vegetables per day?	0 = yes
How much fluid (water, juice, coffee, tea, milk...) is consumed per day?	1 = no
	0.0 = less than 3 cups
	1.0 = more than 5 cups
	0 = unable to eat without assistance
Mode of feeding	1 = self-fed with some difficulty
	2 = self-fed without any problem
Self-view of nutritional status	0 = views self as being malnourished
	1 = is uncertain of nutritional status
	2 = views self as having no nutritional problem

In comparison with other people of the same age, how does the patient consider his/her health status?	0.0 = not as good
	0.5 = does not know
	1.0 = as good
	2.0 = better
Mid-arm circumference (MAC) in cm	0.0 = MAC less than 21
	0.5 = MAC 21 to 22
	1.0 = MAC greater than 22
Calf circumference (CC) in cm	0 = CC less than 3
	1 = CC 31 or greater
**Malnutrition Indicator Score**	
24–30 points	Normal nutritional status
17–23.5 points	At risk of malnutrition
<17 points	Malnourished

IV. Mini Nutritional Assessment Short-Form - MNA-SFA

Has food intake declined over the past 3 months due to loss of appetite, digestive problems, or chewing or swallowing difficulties?	0 = severe decrease in food intake
	1 = moderate decrease in food intake
	2 = no decrease in food intake
Weight loss during the last 3 months	0 = weight loss greater than 3 kg
	1 = does not know
	2 = weight loss between 1 and 3 kg
	3 = no weight loss
Mobility	0 = bedridden or chair bound
	1 = able to get out of bed/chair but does not go out
	2 = goes out
Has the patient suffered psychological stress or acute disease in the past 3 months?	0 = yes
	2 = no
Neuropsychological problems	0 = severe dementia or depression
	1 = mild dementia
	2 = no psychological problems
Body mass index (BMI)	0 = BMI less than 19
	1 = BMI 19 to less than 21
	2 = BMI 21 to less than 23
	3 = BMI 23 or greater
If BMI is not available, replace with the following question	
Calf circumference (CC) in cm	0 = CC less than 31
	3 = CC 31 or greater
**Screening Score**	
12–14 points	Normal nutritional status
8–11 points	At risk of malnutrition
0–7 points	Malnourished

V. Malnutrition Screening Tool - MST

Have you lost weight recently without trying?	
No	0
Unsure	2
If yes, how much weight (kg) have you lost?	
1-2	1
6-10	2
11-15	3
>15	4
Have you been eating poorly because of a decreased appetite?	

No	0
Yes	1
**Total score:**	
Score 0-1	low risk of malnutrition
Score 2	moderate risk of malnutrition
Score 3-5	high risk of malnutrition

VI. Simplified Nutritional Appetite Questionnaire - SNAQ

Did you lose weight unintentionally?	
More than 6 kg in the last 6 month	3
More than 3 kg in the last month	2
Did you experience a decreased appetite over the last months?	1
Did you use supplemental drinks or tube feeding over last month?	1
Total scores:	
1 – no intervention	
2 – moderately malnourished, nutritional intervention	
3 – severely malnourished, nutritional intervention and treatment dietician	

VII. Subjective Global Assessment - SGA

		Ratings		
**Weight loss**	Ratings	7, 6	5,4,3	2,1
0%	7			
<3%	6			
3<5%	5			
5<7%	4			
7<10%	3			
10<15%	2			
>=15%	1			
If weight trend, add 1 point, if weight trend within 1-month, minus point				
**Dietary intake**	Ratings	Ratings		
Good (full share of usual meal)	7	7, 6	5,4,3	2,1
Good (>3/4 -<1 share of usual meal)	6			
Borderline (1/2-3/4 share usual meal)	5			
Boderline (1/2-3/4 share of usual meal, but increasing)	4			
Poor (<1/2 share of usual meal, but increasing)	3			
Poor (<1/2 share of usual meal, no increasing or decreasing)	2			
Starvation (<1/4 of usual meal)	1			
**Gastrointestinal symptoms (that persisted for >2 weeks [nausea; vomiting; diarrhea]**	Ratings	Ratings		
No symptoms	7	7, 6	5,4,3	2,1
Very few intermittent symptoms (1x per day)	6			
Some symptoms (2-3x per day) – improving	5			
Some symptoms (2-3x per day) – no change	4			
Some symptoms (2-3x per day) – getting worse	3			
Some or all symptoms 8>3x per day)	1-2			

**Functional status (nutrition related)**	Ratings	Ratings		
Full functional capacity	6-7	7, 6	5,4,3	2,1
Mild or moderate loss of stamina	3-5			
Severe loss of functional ability (bedridden)	1-2			
**Muscle wastage**		Ratings		
No deletion in all areas	6-7	7, 6	5,4,3	2,1
Mild or moderate depletion	3-5			
Severe depletion	1-2			
**Fat stores**		Ratings		
No depletion in all areas	6-7	7, 6	5,4,3	2,1
Mild to moderate depletion	3-5			
Severe depletion	1-2			
**Edema (nutrition related)**		Ratings		
No edema	6-7	7, 6	5,4,3	2,1
Mild to moderate edema	3-5			
Severe edema	1-2			
Overall SGA rating		7, 6	5,4,3	2,1
Nutritional status		well-nourished	mildly to moderately malnourished	severely malnourished

VIII. Patients Generated Subjective Global Assessment - PG-SGA

1 - 4 are designed to be completed by the patient	
1. Weight	2. Food intake:
In summary of my current and recent weight:<br>I currently weigh about _____ pounds<br>I am about _____ feet _____ inches tall<br>One month ago, I weighed about _____ pounds<br>Six months ago, I weighed about _____ pounds<br>During the past two weeks my weight has:<br>• decreased (1)<br>• not changed (0)<br>• increased (0)	As compared to my normal intake, I would rate my food intake during the past month as<br>• unchanged (0)<br>• more than usual (0)<br>• less than usual (1)<br>I am now taking normal food but less than<br>• normal amount (1)<br>• little solid food (2)<br>• only liquids (3)<br>• only nutritional supplements (3)<br>• very little of anything (4)<br>• only tube feedings or only nutrition by vein (0)
3. Symptoms	4. Activities and Functions

I have had the following problems that have kept me from eating enough during the past two weeks (check all that apply)<br>• no problems eating (0)<br>• no appetite, just did not feel like eating (3)<br>• vomiting (3)<br>• nausea (1)<br>• diarrhea (3)<br>• constipation (1)<br>• dry mouth (1)<br>• mouth sores (2)<br>• smells bother me (1)<br>• things taste funny or have no taste (1)<br>• feel full quickly (1)<br>• problems swallowing (2)<br>• fatigue (1)<br>• pain; where? (3) __________________<br>• other (1)** ______________________<br>**Examples: depression, money, or dental problems	Over the past month, I would generally rate my activity as:<br>• normal with no limitations (0)<br>• not my normal self, but able to be up and about with fairly normal activities (1)<br>• not feeling up to most things, but in bed or chair less than half the day (2)<br>• able to do little activity and spend most of the day in bed or chair (3)<br>• pretty much bed ridden, rarely out of bed (3)
**Additive Score of Boxes 1-4**	

Worksheet 1 – Scoring Weight Loss

Weight loss in 1 month	Points	Weight loss in 6 months
10% or greater	4	20% or greater
5-9.9%	3	10- 19.9%
3-4.9%	2	6- 9.9%
2-2.9%	1	2- 5.9%
0-1.9%	0	0- 1.9%
		**Numerical score from Worksheet 1**

Worksheet 2 – Disease and its relation to nutritional requirements:
Score is derived by adding 1 point for each of the following conditions:

- Cancer
- Presence of decubitus, open wound or fistula
- AIDS
- Presence of trauma
- Pulmonary or cardiac cachexia
- Age greater than 65
- Chronic renal insufficiency

Other relevant diagnoses (specify) ____
Primary disease staging (circle if known or appropriate) I II III IV Other

Numerical score from Worksheet 2

Worksheet 3 – Metabolic Demand
Score for metabolic stress is determined by a number of variables known to increase protein & caloric needs. Note: Score fever intensity or duration, whichever is greater. The score is additive so that a patient who has a fever of 38.8 °C (3 points) for < 72 hrs (1 point) and who is on 10 mg of prednisone chronically (2 points) would have an additive score for this section of 5 points.

Stress	none	low	moderate	high
Fever °F	no fever	> 99 and < 101	≥ 101 and < 102	≥ 102
Fever duration	no fever	< 72 hours	72 hours	> 72 hours
Corticosteroids	no corticosteroids	low dose<br>(< 10 mg prednisone equivalents/day)	moderate dose<br>(≥ 10 and < 30 mg prednisone equivalents/day)	high dose<br>(≥ 30 mg prednisone equivalents/day)

Numerical score from Worksheet 3

Worksheet 4 – Physical Exam
Exam includes a subjective evaluation of 3 aspects of body composition: fat, muscle, & fluid. Since this is subjective, each aspect of the exam is rated for degree. Muscle deficit/loss impacts point score more than fat deficit/loss. Definition of categories: 0 = no abnormality, 1+ = mild, 2+ = moderate, 3+ = severe. Rating in these categories is not additive but are used to clinically assess the degree of deficit (or presence of excess fluid).

<u>Muscle Status</u>	<u>Fat Stores</u>	<u>Fluid status</u>
temples (temporalis muscle)	orbital fat pads	ankle edema
clavicles (pectoralis & deltoids)	triceps skin fold	sacral edema
shoulders (deltoids)	fat overlying lower ribs	ascites
interosseous muscles		
scapula (latissimus dorsi, trapezius, deltoids)		
thigh (quadriceps)		
calf (gastrocnemius)		
Point score for the physical exam is determined by the overall subjective rating of the total body deficit		
No deficit	score = 0 points	
Mild deficit	score = 1 point	
Moderate deficit	score = 2 points	
Severe deficit	score = 3 points	
		**Numerical score from Worksheet 3**

Worksheet 5 – PG-SGA Global Assessment Categories

	**Stage A**	**Stage B**	**Stage C**
**Category**	**well-nourished**	**moderate/suspected malnourished**	**severely malnourished**
**Weight**	No weight loss OR recent non-fluid wt gain	≤ 5% loss in 1 month (≤10% in 6 months)<br>OR Progressive weight loss	> 5% loss in 1 month (>10% in 6 months)<br>OR Progressive weight loss
**Nutrient Intake**	No deficit OR Significant recent improvement	Definite decrease in intake	Severe deficit in intake

**Nutrition Impact Symptoms**	None OR significant recent improvement allowing adequate intake	Presence of NIS (Box 3 of PG-SGA)	Presence of NIS (Box 3 of PG-SGA)
**Functioning**	No deficit OR Significant recent improvement	Moderate functional deficit OR Recent deterioration	Severe functional deficit OR Recent significant deterioration
**Physical Exam**	No deficit OR chronic deficit but with recent clinical improvement	Evidence of mild to moderate loss of muscle mass &/or muscle tone on palpation &/or loss of SQ fat	Obvious signs of malnutrition (e.g., severe loss of muscle, fat, possible edema)

**Triage based on PG-SGA point score:**	
0-1	No intervention required at this time. Re-assessment on routine and regular basis during treatment
2-3	Patient & family education by dietitian, nurse, or other clinician with pharmacologic intervention as indicated by symptom survey (Box 3) and lab values as appropriate
4-8	Requires intervention by dietitian, in conjunction with nurse or physician as indicated by symptoms (Box 3)
≥9	Indicates a critical need for improved symptom management and/or nutrient intervention options

IX. Global Leadership Initiative on Malnutrition criteria -GLIM criteria

Risk screening	**At risk for malnutrition**<br>• Use validated screening tools
Diagnostic Assessment	**Assessment criteria**<br>• Phenotypic<br>○ non-volitional weight loss<br>○ low body mass index<br>○ reduced muscle mass<br>• Etiologic<br>○ reduced food intake or assimilation<br>○ disease burden/inflammatory condition
Diagnosis	**Meets criteria for malnutrition**<br>• requires at least 1 phenotypic criterion and 1 etiologic criterion
Severity Grading	**Determine severity of malnutrition**<br>• Severity determined based on phenotypic criterion

X. Geriatric Nutritional Risk Index (GNRI)

GNRI = (1.489 × serum albumin, g/l) + (41.7 × current/ideal body weight)

XI. Prognostic Nutritional Index (PNI)

PNI = serum albumin (g/L) + 5 × total lymphocyte count (10^9/L)

XII. Controlling Nutritional Status (CONUT)

CONUT = 10^* serum albumin (g/dL) + 0.005^* total lymphocyte count (number/mm^3)

XIII. Nutritional Risk Index (NRI)

NRI = (1.519 × serum albumin (g/L) +41.7× (present weight/usual weight)

XIV. Cachexia:

Defintion		Article
low cachexia index	SMI (cm2/m2) × serum albumin (g/dL)/NLR	M. Akaoka, 2022
Evans, 2008	Weight loss of at least 5%* in 12 months or less in the presence of underlying illness**, PLUS THREE of the following criteria<br>• Decreased muscle strength (lowest tertile)<br>• Fatigue<br>• Anorexia<br>• Low fat-free mass index<br>• Low fat-free mass index<br>• Abnormal biochemistry<br>o increased inflammatory markers CRP (>5.0 mg/l), IL-6 >4.0 pg/ml)<br>o Anemia (<12 g/dl)<br>o Low serum albumin (<3.2 g/dl)	M. J. Alves, 2017
	unintended loss of more than 10% of the pre-illness stable weight prior to the diagnosis of the disease	J. Bachmann, 2013
	cases of unintended weight loss greater than 10% of the pre-illness stable body weight.	J. Bachmann, 2008
	lost 10% or more of his stable pre-illness weight	J. Bachmann, 2009

Fearon, 2011	weight loss greater than 5%, or weight loss greater than 2% in individuals already showing depletion according to current bodyweight and height (body-mass index [BMI] <20 kg/m^2) or skeletal muscle mass (sarcopenia)	M. L. Batista, 2016
	unintentional weight loss of >5% of the habitual weight during the last 3 months or >10% weight loss during the last 6 months.	M. L. Batista, 2013
	had lost >10% of their pre-illness stable weight within 6 months,	S. Bo, 2010
	an unintentional 10% decrease in body weight occurring during the 6 months before surgery.	D. R. Brown, 2001
low cachexia index	SMI (cm2/m2) × serum albumin (g/dL)/NLR	L.R. Brown, 2024
Fearon, 2011	weight loss greater than 5%, or weight loss greater than 2% in individuals already showing depletion according to current bodyweight and height (body-mass index [BMI] <20 kg/m^2) or skeletal muscle mass (sarcopenia)	L. R. Brown, 2025
Fearon, 2011	weight loss greater than 5%, or weight loss greater than 2% in individuals already showing depletion according to current bodyweight and height (body-mass index [BMI] <20 kg/m^2) or skeletal muscle mass (sarcopenia)	X. Chen, 2019
Fearon, 2011	weight loss greater than 5%, or weight loss greater than 2% in individuals already showing depletion according to current bodyweight and height (body-mass index [BMI] <20 kg/m^2) or skeletal muscle mass (sarcopenia)	L. E. Daly, 2018
Evans, 2008	Weight loss of at least 5%* in 12 months or less in the presence of underlying illness**, PLUS THREE of the following criteria<br>• Decreased muscle strength (lowest tertile)<br>• Fatigue<br>• Anorexia<br>• Low fat-free mass index<br>• Low fat-free mass index<br>• Abnormal biochemistry<br>o increased inflammatory markers CRP (>5.0 mg/l), IL-6 >4.0 pg/ml)<br>o Anemia (<12 g/dl)<br>o Low serum albumin (<3.2 g/dl)	G. S. de Castro, 2021
	reported involuntary weight loss exceeding 5% of former body weight during three months period	D. Diakowska, 2010
Fearon, 2011	weight loss greater than 5%, or weight loss greater than 2% in individuals already showing depletion according to current bodyweight and height (body-mass index [BMI] <20 kg/m^2) or skeletal muscle mass (sarcopenia)	W. P. M. Dijksterhuis, 2021
Fearon, 2011	weight loss greater than 5%, or weight loss greater than 2% in individuals already showing depletion according to current bodyweight and height (body-mass index [BMI] <20 kg/m^2) or skeletal muscle mass (sarcopenia)	T. G. Dolin, 2022
Fearon, 2011	weight loss greater than 5%, or weight loss greater than 2% in individuals already showing depletion according to current bodyweight and height (body-mass index [BMI] <20 kg/m^2) or skeletal muscle mass (sarcopenia)	Y. Du, 2025
Fearon, 2011	weight loss greater than 5%, or weight loss greater than 2% in individuals already showing depletion according to current bodyweight and height (body-mass index [BMI] <20 kg/m^2) or skeletal muscle mass (sarcopenia)	H. Fujii, 2020
	Eastern Cooperative Oncology Group performance status (ECOG PS) 1 to 4, grade 1 to 4 anorexia, and weight loss greater than 10% over the past 6 months	Y. Fujiwara, 2014
Fearon, 2011	weight loss greater than 5%, or weight loss greater than 2% in individuals already showing depletion according to current	A. Fukuta, 2019

	bodyweight and height (body-mass index [BMI] <20 kg/m^2) or skeletal muscle mass (sarcopenia)	
	weight loss ≥5% if BMI ≥ 20 kg/m2 or unintentional ≥2% weight loss if BMI < 20 kg/m2	L. A. Gilmore, 2022
	skeletal muscle index x serum albumin)/neutrophil-to-lymphocyte ratio	M. J. Goh, 2022
low cachexia index	SMI (cm2/m2) × serum albumin (g/dL)/NLR	C. Gong, 2022
low cachexia index	SMI (cm2/m2) × serum albumin (g/dL)/NLR	M.C. Gül, 2025
low cachexia index	SMI (cm^2/m^2) × serum albumin (g/dL)/NLR	R. Hamura, 2022
	weight loss >5% in the recent 6 months	J. Han, 2021
	weight loss > 5% and 5 kg during radiotherapy	Y. He, 2023
	weight loss greater than 5% over the 6 months of evaluation, or weight loss greater than 2% in individuals already showing depletion according to current body weight and height (BMI <20 kg/m2 or sarcopenia)	A. E. Hendifar, 2018
Fearon, 2011	weight loss greater than 5%, or weight loss greater than 2% in individuals already showing depletion according to current bodyweight and height (body-mass index [BMI] <20 kg/m^2) or skeletal muscle mass (sarcopenia)	Y. Huang, 2025
cancer cachexia	weight loss > 10% of preillness stable weight within 6 months and serum CRP > 10 mg/L	Y. Jiang, 2014
cancer cachexia	- cancer cachexia defined as weight loss more than 5%<br>- cancer cachexia defined as weight loss more than 10%<br>- cancer cachexia defined as low muscularity (measured by CT)<br>- cancer cachexia defined as weight loss more than 2% + low muscularity	N. Johns, 2014
low cachexia index	SMI (cm^2/m^2) × serum albumin (g/dL)/NLR	T. Kamada
Fearon, 2011	weight loss greater than 5%, or weight loss greater than 2% in individuals already showing depletion according to current bodyweight and height (body-mass index [BMI] <20 kg/m^2) or skeletal muscle mass (sarcopenia)	A. E. J. Latenstein, 2020
Fearon, 2011	weight loss greater than 5%, or weight loss greater than 2% in individuals already showing depletion according to current bodyweight and height (body-mass index [BMI] <20 kg/m^2) or skeletal muscle mass (sarcopenia)	H. Li, 2024
Fearon, 2011	weight loss greater than 5%, or weight loss greater than 2% in individuals already showing depletion according to current bodyweight and height (body-mass index [BMI] <20 kg/m^2) or skeletal muscle mass (sarcopenia)	X.-X. Li, 2024
Fearon, 2011	weight loss greater than 5%, or weight loss greater than 2% in individuals already showing depletion according to current bodyweight and height (body-mass index [BMI] <20 kg/m^2) or skeletal muscle mass (sarcopenia)	W. Ch. Liao, 2020
low cachexia index	SMI (cm^2/m^2) × serum albumin (g/dL)/NLR	T. Matsunaga, 2024
Fearon, 2011	weight loss greater than 5%, or weight loss greater than 2% in individuals already showing depletion according to current bodyweight and height (body-mass index [BMI] <20 kg/m^2) or skeletal muscle mass (sarcopenia)	J. Miller, 2020
Fearon, 2011	weight loss greater than 5%, or weight loss greater than 2% in individuals already showing depletion according to current bodyweight and height (body-mass index [BMI] <20 kg/m^2) or skeletal muscle mass (sarcopenia)	A. Molfino, 2021
	unintentional weight loss >=5% during the previous 6 months	T. Mracek, 2011
low cachexia index	SMI (cm^2/m^2) × serum albumin (g/dL)/NLR	K. Nakashima, 2022
Fearon, 2011	weight loss greater than 5%, or weight loss greater than 2% in individuals already showing depletion according to current bodyweight and height (body-mass index [BMI] <20 kg/m^2) or skeletal muscle mass (sarcopenia)	A. Narasimhan, 2018
	weight loss >5% in the past 6 months or any degree of weight	N. I. P. Neto, 2018

	loss >2% in the last 6 months plus BMI <20 kg/m2	
low cachexia index	SMI (cm^2/m^2) × serum albumin (g/dL)/NLR	T. Nishhiyama, 2025
low cachexia index	SMI (cm^2/m^2) × serum albumin (g/dL)/NLR	T. Ohara, 2025
Fearon, 2011	weight loss greater than 5%, or weight loss greater than 2% in individuals already showing depletion according to current bodyweight and height (body-mass index [BMI] <20 kg/m^2) or skeletal muscle mass (sarcopenia)	S. Olaechea, 2021
Fearon, 2011	weight loss greater than 5%, or weight loss greater than 2% in individuals already showing depletion according to current bodyweight and height (body-mass index [BMI] <20 kg/m^2) or skeletal muscle mass (sarcopenia)	S. Olaechea, 2023
Fearon, 2011	weight loss greater than 5%, or weight loss greater than 2% in individuals already showing depletion according to current bodyweight and height (body-mass index [BMI] <20 kg/m^2) or skeletal muscle mass (sarcopenia)	J. Ose, 2019
	weight loss >5 kg in the 6 months before diagnosis	M. Piciucchi, 2013
low cachexia index	SMI (cm^2/m^2) × serum albumin (g/dL)/NLR	Y. Qin, 2024
Fearon, 2011	weight loss greater than 5%, or weight loss greater than 2% in individuals already showing depletion according to current bodyweight and height (body-mass index [BMI] <20 kg/m^2) or skeletal muscle mass (sarcopenia)	N. E. Rich, 2022
Fearon, 2011	weight loss greater than 5%, or weight loss greater than 2% in individuals already showing depletion according to current bodyweight and height (body-mass index [BMI] <20 kg/m^2) or skeletal muscle mass (sarcopenia)	G.-T. Ruan, 2023
low cachexia index	SMI (cm^2/m^2) × serum albumin (g/dL)/NLR	K. Sakashita, 2025
low cachexia index	SMI (cm^2/m^2) × serum albumin (g/dL)/NLR	T. Shimagaki, 2025
	at least 5% of involuntary weight loss in the previous 6months or BMI<20kg/m^2 and three out of five of the following criteria: decreased muscle strength, fatigue, anorexia, low fat-free mass index or abnormal biochemistry characterised by increased inflammatory biomarkers (PCR>5mg/L; IL-6>4pg/ mL), anaemia (Hb<12 g/dL) or hypoalbuminemia (< 3.2 g/dL)	E. Simoes, 2025
Fearon, 2011	weight loss greater than 5%, or weight loss greater than 2% in individuals already showing depletion according to current bodyweight and height (body-mass index [BMI] <20 kg/m^2) or skeletal muscle mass (sarcopenia)	J. D. P. Soares, 2020
	met all 3 factors of the multifactor cachexia profile: weight loss (≥10% of their pre-illness stable weight within 6 months), low food intake (≤1500 kcal/d), systemic inflammation (CRP ≥10 mg/L)	B. Song, 2009
Fearon, 2011	weight loss greater than 5%, or weight loss greater than 2% in individuals already showing depletion according to current bodyweight and height (body-mass index [BMI] <20 kg/m^2) or skeletal muscle mass (sarcopenia)	N. A. Stephens, 2015
	weight loss >5% in the past 6 months or any degree of weight loss >2% in the last 6 months plus BMI <20 kg/m2	N. I. P. Stephens, 2018
	body weight loss of more than 5% within 6 months; (2) body mass index (BMI) is less than 20kg/m^2, body weight loss of more than 2% within 6 months; (3) combined with sarcopenia, Body weight decreased by more than 2% within 6 months.	Y. Sun, 2014
	>5% weight loss in the past six months or body mass index (BMI) <20 or >2% weight loss in the past six months with sarcopenia.	D. Sun, 2024
	weight loss was >10% of their pre-illness stable weight within 6 months and if their serum CRP level was >10 mg/L	F. Sun, 2010
Fearon, 2011	weight loss greater than 5%, or weight loss greater than 2% in individuals already showing depletion according to current	H. Suzuki, 2021

	bodyweight and height (body-mass index [BMI] <20 kg/m^2) or skeletal muscle mass (sarcopenia)	
low cachexia index	SMI (cm^2/m^2) × serum albumin (g/dL)/NLR	Y. Takano, 2023
	weight loss > 5 % or > 2% with either a BMI of less than 20 kg/m2 or the presence of sarcopenia	T. Takeda, 2021
Fearon, 2011	weight loss greater than 5%, or weight loss greater than 2% in individuals already showing depletion according to current bodyweight and height (body-mass index [BMI] <20 kg/m^2) or skeletal muscle mass (sarcopenia)	E. E. Talbert, 2018
Fearon, 2011	weight loss greater than 5%, or weight loss greater than 2% in individuals already showing depletion according to current bodyweight and height (body-mass index [BMI] <20 kg/m^2) or skeletal muscle mass (sarcopenia)	F. Tambaro, 2024
low cachexia index	SMI (cm^2/m^2) × serum albumin (g/dL)/NLR	H. Tanda, 2025
low cachexia index	SMI (cm^2/m^2) × serum albumin (g/dL)/NLR	Y. Tanji, 2022
Fearon, 2011	weight loss greater than 5%, or weight loss greater than 2% in individuals already showing depletion according to current bodyweight and height (body-mass index [BMI] <20 kg/m^2) or skeletal muscle mass (sarcopenia)	Z. Tao, 2024
Asian Working Group for Cachexia (AWGC) criteria	body mass index (BMI) < 21 kg/m^2 or a weight loss >2% in the past 3-6 months as mandatory, combined with one or more of the following: anorexia, decreased grip strength (<28 kg for men and <18 kg for women), or C-reactive protein (CRP) > 0.5 mg/dl	S. Unome, 2025
Fearon, 2011	weight loss greater than 5%, or weight loss greater than 2% in individuals already showing depletion according to current bodyweight and height (body-mass index [BMI] <20 kg/m^2) or skeletal muscle mass (sarcopenia)	A. van der Werf, 2018
low cachexia index	SMI (cm^2/m^2) × serum albumin (g/dL)/NLR	Q. Wan, 2022
Fearon, 2011	weight loss greater than 5%, or weight loss greater than 2% in individuals already showing depletion according to current bodyweight and height (body-mass index [BMI] <20 kg/m^2) or skeletal muscle mass (sarcopenia)	S. Yasui-Yamada, 2020
	weight loss >10% plus CRP >10 mg/dl	D. Zhang, 2008
	weight loss >10 %;	D. Zhang, 2007
Fearon, 2011	weight loss greater than 5%, or weight loss greater than 2% in individuals already showing depletion according to current bodyweight and height (body-mass index [BMI] <20 kg/m^2) or skeletal muscle mass (sarcopenia)	X. Zhang, 2025
Fearon, 2011	weight loss greater than 5%, or weight loss greater than 2% in individuals already showing depletion according to current bodyweight and height (body-mass index [BMI] <20 kg/m^2) or skeletal muscle mass (sarcopenia)	C. L. Zhuang, 2022

XV. Guidelines

Name of the guideline		Articles
**current guidelines of the French health authority (HAS)**	Classify malnutrition as absent, moderate, or severe based on body weight variations within the last 6 month or body mass index (BMI) or serum albumin levels	A. Attar, 2012
**ESPEN 2015**	• Alternative 1: BMI <18,5 kg/m^2<br>• Alternative 2: Weight loss (unintentional) >10% indefinite of time, or >5% over the last 3 months combined with either	J. Cao, 2021

	○ BMI <20 kg/m^2 if <70 years of age, or <22 kg/m^2 if ≥70 years of age or<br>○ FFMI <15 and 17 kg/m^2 in women and men, respectively.	
**ESPEN 2006**	Presence of at least one of the following criteria: weight loss >10-15% within 6 months, BMI<18.5 kg/m^2, Subjective Global Assessment Grade C, serum albumin <30 g/l (with no evidence of hepatic or renal dysfunction)	Y. Fukuda, 2015
**Chang's protocol**	Nutritional assessments were based on the following five parameters: percentage ideal body weight, triceps skin fold thickness (TSF), arm muscle circumference (AMC), serum albumin and lymphocyte count	J. M. Gallardo-Valverd 2005
**Chonnam National University Hwasun Hospital-Nutritional Risk Screening Tool (CNUHH-NRST)**	consisted of the following data: body mass index (BMI), weight loss, recent food intake, metabolic stress, and age;	S. Y. Lee, 2020
**Chonnam National University Hwasun Hospital-Nutritional Risk Screening Tool (CNUHH-NRST)**	consisted of the following data: body mass index (BMI), weight loss, recent food intake, metabolic stress, and age;	S. Y. Lee, 2018
**Chonnam National University Hwasun Hospital-Nutritional Risk Screening Tool (CNUHH-NRST)**	consisted of the following data: body mass index (BMI), weight loss, recent food intake, metabolic stress, and age;	S. Y. Lee, 2019
**ESPEN 2015**	• Alternative 1: BMI $<18,5$ kg/m^2<br>• Alternative 2: Weight loss (unintentional) >10% indefinite of time, or >5% over the last 3 months combined with either<br>○ BMI <20 kg/m^2 if <70 years of age, or <22 kg/m^2 if ≥70 years of age or<br>○ FFMI <15 and 17 kg/m^2 in women and men, respectively.	J. H. Park, 2021
**ESPEN 2015**	• Alternative 1: BMI $<18,5$ kg/m^2<br>• Alternative 2: Weight loss (unintentional) >10% indefinite of time, or >5% over the last 3 months combined with either<br>○ BMI <20 kg/m^2 if <70 years of age, or <22 kg/m^2 if ≥70 years of age or<br>○ FFMI <15 and 17 kg/m^2 in women and men, respectively.	P. Y. Wang, 2021
**ASPEN 2012**	Malnutrition was defined as having any of the following characteristics:<br>• body mass index (BMI) <18.5 kg/m^2<br>• weight loss >10% during previous 6-month, preoperative albumin level of <3.5 mg/dL	J. H. Wolf, 2020
**ESPEN 2015**	• Alternative 1: BMI $<18,5$ kg/m^2<br>• Alternative 2: Weight loss (unintentional) >10% indefinite of time, or >5% over the last 3 months combined with either<br>○ BMI <20 kg/m^2 if <70 years of age, or <22 kg/m^2 if ≥70 years of age or<br>○ FFMI <15 and 17 kg/m^2 in women and men, respectively.	X. J. Ye, 2018
**ESPEN 2015**	• Alternative 1: BMI $<18,5$ kg/m^2<br>• Alternative 2: Weight loss (unintentional) >10% indefinite of time, or >5% over the last 3 months combined with either<br>○ BMI <20 kg/m^2 if <70 years of age, or <22 kg/m^2 if ≥70 years of age or<br>○ FFMI <15 and 17 kg/m^2 in women and men, respectively.	L. Yin, 2021

**ASPEN 2012**	Malnutrition was defined as having any of the following characteristics:<br>• body mass index (BMI) <18.5 kg/m^2<br>• weight loss >10% during previous 6-month,<br>• preoperative albumin level of <3.5 mg/dL	Y. X. Zhang, 2022
**ESPEN 2015**	• Alternative 1: BMI <18,5 kg/m^2<br>• Alternative 2: Weight loss (unintentional) >10% indefinite of time, or >5% over the last 3 months combined with either<br>○ BMI <20 kg/m^2 if <70 years of age, or <22 kg/m^2 if ≥70 years of age or<br>○ FFMI <15 and 17 kg/m^2 in women and men, respectively.	X. Zhao, 2022
**ESPEN 2015**	• Alternative 1: BMI <18,5 kg/m^2<br>• Alternative 2: Weight loss (unintentional) >10% indefinite of time, or >5% over the last 3 months combined with either<br>○ BMI <20 kg/m^2 if <70 years of age, or <22 kg/m^2 if ≥70 years of age or<br>○ FFMI <15 and 17 kg/m^2 in women and men, respectively.	X. H. Hua, 2022

Supplementary Document S4: Detailed strategy for the selection process

The selection of full-text content was performed by three groups which included two independent reviewers (B.Cs.B. with G.C., L.F. with P.M., and H.N. with D.D). Cohen's kappa coefficient was calculated after every selection step to assess the agreement. In the case of two groups (L.F. with P.M., and H.N. with D.D.) B.Cs.B as a third person solved the disagreements, while in the group which included B.Cs.B and G.C., P.M. as a third person solved the disagreements.

Supplementary Document S5: Detailed strategy for data collection

The extracted data on the standardized collection sheet included: information about the study – first author, year of publication, country of origin, and study design; demographic data – sample size, sex, and age distribution; population characteristics – cancer type and cancer stage; cut-off values of the reported risk factors (exposure); and outcome-related information – definition of impaired nutritional status e.g.: malnutrition risk, malnutrition diagnosis, (including cut-off values), and moment of measurement. For the meta-analyses, the number of patients with malnutrition and the total number of patients in the exposed (e.g., male gender, age ≥ 65 years) and comparator group (e.g., female gender, age < 65 years) were extracted from each article. In the case, if the mentioned count data is not available, we did not use that article in the meta-analysis. The data extraction process was done independently by reviewers (G.C., P.M., L.F., D.D., and B.Cs.B.) in each article manually and crosschecked each other's data pool.

Supplementary Document S6: Leave-one-out analysis

We did leave-one-out analysis to assess the possible influence of articles on the overall results. Cases that are considered as possibly influential with respect to any of the shown measures are marked with a 'star' in column 'Influential.' Note that the chosen cut-offs are (somewhat) arbitrary based on dmetar package.
The provided column names are: 'Influental', 'effect size', '95% CI', 'I2', 'Std residual', 'Dffits', 'Cook's dist.', 'Covariance ratio', 'Hat value'. Effect size is the pooled effect size without the given study. The 95% confidence interval of the pooled effect size without the given study. I^2 means the Higgins&Thomson I^2 heterogeneity value without the given study. Std residual is the studentized residuals. It shows the deleted residual divided by its estimated standard deviation. Dffits is the difference in fits. It quantifies the number of standard deviations that the fitted value changes without the given study. (Typical threshold is $3*\sqrt{\frac{P}{k-P}}$). Cook's dist means Cook's distance. It depends on both the residual and leverage of the omitted study. (Typical threshold value is 2). Covariance ratio is the covariance ratio. It shows the

change in the determinant of the covariance matrix of the effect size. (Typical threshold value is 1) Hat value means the value of the hat matrix without the given study. (Typical threshold is $3*\frac{P}{k}$)

Based on the leave-one--out analyses (LOO) we found relevant change in the effect (> 1 range in OR) or relevant change in the decision on the null-hypothesis in the following cases (**see figures in Supplementary Documents S10):**

In cases of "Association between malnutrition-related complication risk and sex in esophageal cancer" (Fig. 16.1); "Association between malnutrition-related complication risk and macrovascular involvement in colorectal cancer" (Fig. 54.1); "Association between malnutrition-related complication risk and etiology in hepatocellular carcinoma" (Fig.51.1); and "Association between malnutrition-related complication risk and age ≥65 in esophageal cancer" (Fig.31.1) we found an article, that leaving out would change the decision on the null-hypothesis:

- In the case of "Association between malnutrition-related complication risk and sex in esophageal cancer" (Fig. 16.1), if we omit F.K. Xiao, 2021, the OR would be 1.20 (95%Cl 1.07-1.34). In this case, the overall results would be statistically significant, meaning a statistically significant higher odds for males, compared to females.
- In the case of "Association between malnutrition-related complication risk and macrovascular involvement in colorectal cancer" (Fig. 54.1), if we omit C. Akgul, 2024, the OR would be 1.12 (95%Cl 0.99-1.26). In this case, the overall results would not be statistically significant, meaning not a statistically significant higher odds for macrovascular involvement.
- In the case of "Association between malnutrition-related complication risk and etiology in hepatocellular carcinoma" (Fig. 51.1), if we omit A. Hiraoka, 2023 the OR would be 1.24 (95%Cl 1.10-1.39). In this case, the overall results would be statistically significant, meaning a statistically significant higher odds for viral etiology, compared to non-viral.
- In the case of "Association between malnutrition-related complication risk and age ≥65 in esophageal cancer" (Fig.31.1), if we omit Y. Sun, 2025, the OR would be 1.24 (95%Cl 0.99-1.55). In this case, the overall results would not be statistically significant, meaning not a statistically significant higher odds for age ≥65 in esophageal.

Although there were additional influential studies in some cases, but their effect was not relevant.

Supplementary Document S7: Detailed strategy for overlapping population

In the case of more than one article reported on the same population across the eligible articles, those reporting on the higher sample size were analyzed. When different screening tools were used to measure the same risk factor in one article, the data for each screening tool were retrieved. However, we always preferred the screening tool that was used by more articles for this risk factor, as well as the screening tool that had more criteria to assess malnutrition. Therefore, we have no overlapping populations nor multiple uses of the same article in meta-analyses. Data collected prior to anticancer treatment (e.g., neoadjuvant therapy or surgery) were included in the analysis when post-treatment data were also provided to avoid an unintentional influence of therapy on malnutrition, which would distort the objective effect of the risk factor.

Author	DOI	Risk factor	Strategy
A. Fujio, 2022	10.1620/tjem.256.43	sex	use the whole population data, not the subgroups according age groups
A. Daniele, 2017	10.21873/anticanres.11447	sex	use the data in group MNA <17
A. S. Senger, 2022	NA	CA19-9	exclude, because normal CA19-9 level was not reported
A. van der Werf, 2018	10.1080/01635581.2018.1504092	sex	use the data where cachexia was defined according Fearon criteria, not clinical assessment
B. Akagunduz, 2022	10.1007/s12029-021-00664-4	N stage	exclude from the analysis, because patients got treatment
D. D. Huang, 2022	10.1038/s41430-022-01109-2	sex<br>ASA performance status	overlapping population with Huang, 2022 (10.1016/j.ejso.2021.02.032), chose the bigger population number
D. Huang, 2022	10.1016/j.ejso.2021.02.032		
D. Huang, 2022	10.1016/j.ejso.2021.02.032	sex<br>ASA performance status	by GLIM assessment, chose the data with sceletal muscle mass, not handgrip strength
E. Paillaud, 2014	10.1016/j.clnu.2013.05.014	sex	use the data in group MNA <17
F. Sun, 2010	NA	sex	overlapping population, chose F. Sun, 2010 (NA), because of bigger population number
F. Sun, 2010	10.1007/bf03256358		
F. Terasaki, 2021	10.1007/s13304-020-00792-9	CA19-9	exclude from the analysis, because cut off value 200 (only one with this cut off)
H. Jin, 2021	10.5230/jgc.2021.21.e14	T stage<br>N stage	exclude from the analysis, because the population got preoperative treatment, in other articles only surgery
H. Xie, 2022	10.3389/fnut.2022.902080	tumour site<br>N stage<br>T stage<br>sex	overlapping population, chose H. Xie, 2022 (10.3389/fnut.2022.794489), because of the bigger population
H. Xie, 2020	10.7150/JCA.49383		
H. Xie, 2022	10.3389/fnut.2022.794489		

J. Bachmann, 2008	10.1007/s11605-008-0505-z	sex	overlapping population, chose J. Bachmann, 2008 (10.1007/s11605-008-0505-z)
J. Bachmann, 2009	10.1186/1471-2407-9-255		
J. Bachmann, 2008	10.1007/s11605-008-0505-z	sex	use the whole population, not only the resectable cases
J. Bachmann, 2013	10.1080/01635581.2013.804580	diabetes mellitus	overlapping population, chose J. Bachmann, 2013 (10.1080/01635581.2013.804580)
J. Bachmann, 2008	10.1007/s11605-008-0505-z		
J. Cao, 2021	10.1016/j.currproblcancer.2020.100638	sex	chose the data measured by NRS-2002
J. Kang, 2022	10.1155/2022/3889588	sex<br>tumour site	cohort and testing group analyzed together
J. Ose, 2019	10.3390/metabo9090178	sex	analyzed data about cachexia, not pre-cachexia
J. Sun, 2016	10.1016/j.jss.2016.09.050	T stage	exclude from the analysis, because the data was about T0
J. F. Feng, 2013	10.2147/tcrm.s56159	alcohol<br>active smoking<br>T stage<br>N stage<br>tumour site	analyzed the PNI <42 and PNI 42-52 together
K. Fujiya, 2018	10.1245/s10434-018-6342-8	sex<br>T stage<br>comorbidities	used the preoperative data
K. Sugawara, 2020	10.1016/j.jgo.2020.02.007	sex<br>ASA performance status<br>tumour site	used the preoperative data
K. Sugawara, 2020	10.1016/j.jgo.2020.02.007	sex<br>tumour site	overlapping population, chose K. Sugawara, 2020 (10.1016/j.jgo.2020.02.007) because of the used measurement was PNI, instead of GNRI
K. Sugawara, 2021	10.1002/jpen.1978		
K. Takao, 2020	10.21873/invivo.12184	sex	used the pretreatment data
K. Fu, 2017	NA	tumour site	medium and high-risk malnutrition (NRS 2002 3-5 and NRS 2022 >5) analyzed together
K. Ge, 2021	10.1080/01635581.2020.1841252	T stage	cancer type is an esophageal junction, we included in the analysis of gastric cancer
K. Kouzu, 2020	10.1007/s10388-020-00795-w	T stage	chose the data about pathological T stage, not clinical
K. Mimatsu, 2017	10.21873/anticanres.11812	Ca19-9<br>CEA level<br>sex	analyzed the data measured by CONUT, not PNI

		T stage	
K. Sakurai, 2015	10.1245/s10434-015-4814-7	CVD<br>sex<br>T stage<br>chronic kidney disease<br>neoadjuvant chemotherapy	overlapping population, chose K. Sakurai, 2015, because of the bigger population
K. Sakurai, 2016	10.1245/s10434-016-5272-6		
K. Sakurai, 2015	10.1245/s10434-015-4814-7	CVD	put the ischaemic heart disease into the analysis and not the cerebrovascular heart disease
K. Takao, 2020	10.21873/invivo.12184	sex<br>N stage	used the pre-treatment data
K. Tanaka, 2021	10.1016/j.clnesp.2020.11.009	sex	use the data in group MNA <17<br>analyzed the data measured by CONUT, not GNRI
L. B. Xu, 2022	10.3389/fonc.2022.851091	sex	overlapping population, chose L.B. Xu,2022 (10.3389/fonc.2022.851091) because of the bigger population
L. B. Xu, 2022	10.1002/jpen.2127		
L. B. Xu, 2022	10.3389/fonc.2022.851091	sex	chose the weight loss criteria for the definition of malnutrition by the GLIM criteria
L. P. Zhou, 2021	10.1016/j.nut.2020.111044	ASA performance status	by GLIM assessment, chose the data with sceletal muscle mass, not handgrip strength
M. Shibutani, 2015	10.1186/s12885-015-1537-x	sex	analyzed the preoperative data
M. Tamai, 2021	NA	sex	analyzed the preoperative data
M. F. Chen, 2021	10.3390/nu13092997	neoadjuvant therapy	analyzed the pretreatment data
M. L. Batista, 2016	10.1002/jcsm.12037	sex	overlapping population, chose the L. M. Batista, 2013 (10.1016/j.cyto.2012.10.023) because of the bigger population
M. L. Batista, 2013	10.1016/j.cyto.2012.10.023		
M. Piciucchi, 2013	NA	sex	excluded from the analysis, because of the study type (abstract)
M. Sasaki, 2020	10.1038/s41598-020-67285-y	sex<br>CEA<br>tumour site	overlapping population, chose M. Sasaki, 2019 (10.23922/jarc.2018-041), because of the used measurement was PNI, instead of GNRI
M. Sasaki, 2019	10.23922/jarc.2018-041		

M. Shibutani, 2015	10.1186/s12885-015-1537-x	sex<br>CA19-9<br>CEA<br>tumour site<br>T stage	analyzed the whole population's preoperative data (surgery in 2005 and 2006-2011)
M. Tamai, 2021	NA	sex<br>CA19-9<br>CEA<br>tumour site<br>T stage	analyzed the pretreatment data
M. Wobith, 2022	10.1016/j.clnesp.2022.06.004	sex<br>ASA performance status	by GLIM assessment, chose the data with skeletal muscle mass measured by CT, not BIA
N. Kubo, 2018	10.1007/s10388-018-0644-6	sex	overlapping population, chose N. Kubo, 2017 (10.1007/s10388-016-0548-2), because of the used measurement was PNI, instead of GNRI
N. Kubo, 2017	10.1007/s10388-016-0548-2		
N. E. Rich, 2022	10.1016/j.cgh.2021.09.022	sex	analyzed data about cachexia, not pre-cachexia
N. Harimoto, 2018	10.1245/s10434-018-6672-6	sex<br>Child-Pugh class	overlapping population, chose N. Harimoto, 2018, because of the bigger population
N. Harimoto, 2017	10.1007/s00268-017-4097-1		
N. Hirahara, 2020	10.18632/oncotarget.27670	sex<br>T stage<br>tumour site	overlapping population, chose N. Hirahara, 2018 (10.1097/MJT.0000000000000414) because of the used measurement was CONUT
N. Hirahara, 2018	10.1097/MJT.0000000000000414		
N. Hirahara, 2018	10.18632/oncotarget.27635		
N. Hirahara, 2020	10.18632/oncotarget.27670	ASA performance status	chose the GNRI <94,8 (calculated for overall survival), instead of 90,9 (calculated for cancer specific survival)
N. Johns, 2014	10.1371/journal.pone.0083618	sex	four different cachectic groups were created, we analyzed data measured by weight loss more 5%
R. Sato, 2020	10.1007/s00595-020-02007-5	sex<br>CA19-9<br>CEA<br>T stage	overlapping population, chose the L. R.Sato, 2021 (10.1007/s00595-020-02066-8) because of the bigger population
R. Sato, 2021	10.1007/s00595-020-02066-8		

R. Tokunaga, 2015	10.1097/dcr.0000000000000458	CA19-9<br>neoadjuvant chemotherapy	overlapping population, chose the L. Tokunaga, 2015 (10.1007/s00384-016-2668-5) because of the bigger population
R. Tokunaga, 2016	10.1007/s00384-016-2668-5		
R. Tokunaga, 2015	10.1097/dcr.0000000000000458	sex<br>CEA<br>tumour site	overlapping population, choose the L. Tokunaga,2016(10.1097/dcr.0000000000000458) analyzed the data measured by CONUT, not PNI
R. Tokunaga, 2016	10.1007/s00384-016-2668-5		
S. B. Chen, 2021	10.3389/fsurg.2021.752792	sex<br>T stage<br>tumour site	analyzed the data measured by PNI, not GNRI
S. Ide, 2021	10.1186/s12957-021-02139-z	N stage	exclude from the analysis, because the population got preoperative treatment and metastasis
S. Y. Lee, 2020	10.1016/j.amjsurg.2020.02.015	ASA performance status	overlapping population, chose the L. Lee, 2018 (10.1038/s41430-018-0112-3) because of the bigger population
S. Y. Lee, 2018	10.1038/s41430-018-0112-3		
T. Mracek, 2011	10.1038/s41430-018-0112-3	sex	analyzed the two cohorts together
T. Hatanaka, 2019	10.2169/internalmedicine.1594-18	BCLC stage	excluded because the population is not well-described
T. Hayama, 2022	10.1038/s41598-022-07540-6	sex	overlapping population, chose T. Hayama, 2020 (10.1038/s41598-020-70252-2) because the used measurement was CONUT
T. Hayama, 2020	10.1038/s41598-020-70252-2		
T. Matsunaga, 2022	10.1186/s12885-022-09638-6	sex<br>diabetes<br>CVD<br>T stage<br>ASA performance status	chose the GNRI <95,8 (calculated for overall survival), instead of 97 (calculated for cancer specific survival)
T. Tominaga, 2019	10.1007/s00595-019-01910-w	sex	overlapping population, chose the L. Tominaga, 2020 (10.1007/s00595-020-02050-2) because of the bigger population
T. Tominaga, 2020	10.1007/s00595-020-02050-2		
T. Tominaga, 2019	10.1007/s00595-019-01910-w	comorbidities<br>ASA performance status	use the whole population data, not the subgroups according age groups
W. Peng, 2021	10.1186/s12893-021-01334-18	alfa-fetoprotein	analyzed the data before propensity score matching
X. H. Hua, 2022	10.1371/journal.pone.0083618	sex	analyzed the data measured by NRS-2002

		diabetes mellitus<br>chronic kidney disease<br>neoadjuvant therapy	
X. Wang, 2020	10.1007/s10147-020-01743-5	anemia	analyzed together the mild and moderate anemia
X. Zhang, 2021	10.2147/cmar.s316437	CVD	analyzed the coronary artery disease, instead of cerebrovascular
Y. Bo, 2015	10.6133/apjcn.2015.24.4.26	alfa fetoprotein	analyzed NRI 83,5-97,5 and <83,5 together
Y. Chen, 2020	10.1007/s00270-020-02519-0	aspartate aminotransferase<br>alfa fetoprotein	overlapping population, chose the Y. Chen, 2020 (10.1007/s00270-020-02519-0) because of the bigger population
Y. Chen, 2022	10.1017/s000711452100492x		
Y. Geng, 2015	10.1016/j.ejso.2015.07.022	sex<br>CA19-9<br>tumour site	analyzed together validation and test group together
Y. Iseki, 2015	10.1371/journal.pone.0132488	sex<br>T stage<br>tumour site	analyzed the data measured by CONUT, not PNI
Y. S. Mao, 2020	10.1017/S0007114520002299	sex	analyzed the data measured by CONUT, not PNI
Y. Saito, 2017	10.1016/j.jamcollsurg.2017.07.865.	sex	overlapping population, chose the Y. Saito, 2017 (10.1016/j.jamcollsurg.2017.07.865.) because population did not get treatment
Y. Saito, 2021	10.3892/ol.2020.12414		
Y. Takamizawa, 2020	10.1186/s12885-020-07560-3	sex<br>CEA<br>tumour site<br>age	analyzed the data measured by CONUT, not PNI
Y. X. Zhang, 2022	10.1007/s13304-022-01293-7	CVD	analyzed the data of arrhythmia, not the history of stroke, because of the bigger patient's number
Y. Zhao, 2022	10.1080/01635581.2021.1982997	tumour site	analyzed the data before propensity score matching
F. Akad, 2024	10.3390/nu16101485	sex	analyzed together PG-SGA B+C
P. Fang, 2024	10.21037/jtd-24-187	age<br>sex<br>tumor site	analyzed the article with doi:10.3389/fnut.2022.983039, because of bigger population
P. Fang, 2002	10.3389/fnut.2022.983039		
Zuo, 2024	10.1016/j.nut.2024.112363	sex	choose the article with doi: 10.1016/j.nut.2024.112363, because bigger population
Zuo, 2023	10.1016/j.clnesp.2023.12.004		
Feng, 2022	10.3389/fimmu.2022.1015366	sex<br>alcohol<br>hypertension<br>tumor site<br>macrovascular invasion	choose article with doi: 10.3389/fimmu.2022.1015366
Feng, 2024	10.1016/j.ejso.2024.108323		
Fujiwara, 2021	10.1159/000513961	sex	choose article with doi: 10.1159/000513961

Fujiwara, 2022	10.1007/s10388-022-00961-16	N-stage<br>T-stage<br>tumor site	
X. Ma, 2025	10.1186/s12876-025-03766-6	sex	choose article with doi: 10.20960/nh.05079, and analyze data measured by PNI, not CONUT
X. Ma, 2024	10.20960/nh.05079		
Tekin, 2023	10.37047/jos.2023-96450	sex<br>comorbidities<br>CVD<br>hypertension<br>serum CEA<br>tumor site	analyzed data measured by PNI, not GNRI
Matsunaga, 2022	10.1186/s12885-022-09638-6	sex<br>ASA performance status<br>T-stage<br>macrovascular invasion	choose article with doi: 10.1186/s12885-022-09638-6
Matsunaga, 2024	10.1007/s00595-024-02850-w		
Takashi, 2020	10.1007/s13304-020-00745-2	sex<br>ASA performance status<br>tumor site	choose article with doi: 10.1007/s13304-020-00745-2
Takashi, 2024	NA		
Zhang, 2023	10.4240/wjgs.v15.i2.211	sex	analyzed data measured by PNI, not others
L.J. Wang, 2025	10.5306/wjco.v16.i4.102294	sex<br>anaemia<br>serum CA19-9<br>serum CEA	analyzed data measured by PNI, not others
Shibutani, 2023	10.21873/invivo.13265	sex<br>serum CEA<br>T-stage<br>tumor site	analyzed data measured by PNI, not others
Nakamaru, 2023	10.1002/jso.27488	sex<br>serum CEA	choose article with doi: 10.1016/j.clnesp.2022.03.011
Nakamaru, 2025	10.7759/cureus.86268		
Nakamaru, 2022	10.1016/j.clnesp.2022.03.011		
Sato, 2022	10.23922/jarc.2022-053	sex	choose article with doi: 10.1007/s00595-020-02066-8
Sato, 2021	10.1007/s00595-020-02066-8		
Hiraoka, 2023	10.1111/hepr.13934	sex<br>ECOG status<br>BCLC stage<br>Child-Pugh class<br>etiology of HCC	analyzed GNRI data together, as GNRI <98
Ch. K. Yang, 2024	10.1016/j.clnesp.2023.12.148	sex<br>BCLC stage<br>Child-Pugh class	analyzed data measured by PNI, not others
Chen, 2024	10.5582/bst.2024.01108	sex	analyzed data measured by PNI, not others
Tsukagoshi, 2024	10.3390/nu16070940	sex<br>Child-Pugh class<br>etiology of HCC<br>macrovascular invasion	analyzed data measured by PNI, not others

N. Funamizzu, 2025	10.3390/cancers17091448	sex	choose article with doi: 10.3390/cancers17091448
N. Funamizu, 2022	10.1038/s41598-022-18077-z		
Takano, 2023	10.1007/s00423-023-02883-8	sex	moderate and severe cachexia
D.D. Huang, 2022	10.1038/s41430-022-01109-2	sex<br>ASA performance status	choose article with doi: 10.1038/s41430-022-01109-2
D. D. Huang, 2021	10.1016/j.clnu.2021.01.038		
T. Wu, 2022	10.3390/nu14235166	sex	moderate and severe malnutrition (according GLIM) analyzed together
L. Chang, 2022	10.1097/CJI.0000000000000438	sex<br>smoking	choose article with doi: 10.1097/CJI.0000000000000438
L. Chang, 2023	10.21037/tcr-23-1193		
Ma, 2025	10.1186/s12876-025-03766-6	smoking<br>alcohol<br>hypertension<br>chronic respiratory diseases<br>serum CA19-9<br>serum CEA<br>tumor site<br>macrovascular invasion	choose article, with doi: 10.1186/s12876-025-03766-6
Ma, 2024	10.20960/nh.05079		
Ch. K. Yang	10.1016/j.clnesp.2023.12.148	smoking<br>alcohol<br>serum alanine aminotransferase<br>serum alfa-fetoprotein<br>etiology of HCC	analyzed data measured by PNI, not others
Yoshida, 2017	10.1007/s00423-017-1553-1	sex<br>chronic respiratory diseases<br>ASA performance status<br>neoadjuvant chemotherapy	mild and moderate malnutrition analyzed together
Nakmaru, 2023	10.1002/jso.27488	ASA performance status<br>N-stage<br>tumor site	choose article, with doi: 10.1002/jso.27488
Nakamaru, 2025	10.7759/cureus.86268		
R. Sato, 2022	10.23922/jarc.2022-053	serum CA19-9<br>serum CEA<br>T-stage	choose article, with doi: 10.1007/s00595-020-02066-8
R. Sato, 2021	10.1007/s00595-020-02066-8		
T. Kosuga, 2019	10.21873/anticanres.13901	N-stage	choose clinical N-stage, instead of the pathological N-stage
Hashimoto, 2024	10.21873/invivo.13514	T-stage	choose clinical T-stage, instead of pathological N stage
M. Yamamoto, 2025	10.1002/ags3.70057	T-stage	choose clinical T-stage, instead of pathological N stage
Sakurai, 2015	10.1245/s10434-015-4814-7	neoadjuvant therapy	choose article, with doi: 10.1245/s10434-015-4814-7
Sakurai 2016	10.1245/s10434-016-5272-6		

Supplementary Document S8: References of the included studies

Abe, A., Ito, Y., Hayashi, H., Nakayama, A., Furuta, H., Momokita, M., Hasegawa, H., & Tsunoda, A. (2022). Relationship between nutritional biomarkers and occlusal status in gastric cancer patients using the Eichner index: Observational study. *Medicine (Baltimore)*, *101*(11). https://doi.org/10.1097/md.0000000000029094

Abe, A., Nakayama, A., Otsuka, Y., Shibata, K., Matsui, Y., Ito, Y., Hayashi, H., Momokita, M., & Taniguchi, S. (2023). Relationship of preoperative oral hypofunction with prognostic nutritional index in gastric cancer: A case-control retrospective study. *Plos one*, *18*(6), e0283911.

Abe, S., Nozawa, H., Sasaki, K., Murono, K., Emoto, S., Yokoyama, Y., Matsuzaki, H., Nagai, Y., Shinagawa, T., Sonoda, H., & Ishihara, S. (2024). Nutritional Status Indicators Predict Tolerability to Adjuvant Chemotherapy in Patients with Stage II/III Rectal Cancer Undergoing Neoadjuvant Chemoradiotherapy. *Digestion*, *105*(5), 345-358. https://doi.org/10.1159/000539211

Abe, T., Nakata, K., Kibe, S., Mori, Y., Miyasaka, Y., Ohuchida, K., Ohtsuka, T., Oda, Y., & Nakamura, M. (2018). Prognostic Value of Preoperative Nutritional and Immunological Factors in Patients with Pancreatic Ductal Adenocarcinoma. *Annals of Surgical Oncology*, *25*(13), 3996-4003. https://doi.org/10.1245/s10434-018-6761-6

Ahiko, Y., Shida, D., Horie, T., Tanabe, T., Takamizawa, Y., Sakamoto, R., Moritani, K., Tsukamoto, S., & Kanemitsu, Y. (2019). Controlling nutritional status (CONUT) score as a preoperative risk assessment index for older patients with colorectal cancer. *BMC Cancer*, *19*(1), 946. https://doi.org/10.1186/s12885-019-6218-8

Akad, F., Filip, B., Preda, C., Zugun-Eloae, F., Peiu, S. N., Akad, N., Crauciuc, D. V., Vatavu, R., Gavril, L. C., Sufaru, R. F., & Mocanu, V. (2024). Assessing Nutritional Status in Gastric Cancer Patients after Total versus Subtotal Gastrectomy: Cross-Sectional Study. *Nutrients*, *16*(10). https://doi.org/10.3390/nu16101485 10.3390/nu16101485.

Akagunduz, B., Demir, M., & Atcı, M. M. (2022). Controlling Nutritional Status (CONUT) Score Is a Prognostic Factor for Patients with Gastric Cancer Treated by Perioperative FLOT [Article]. *Journal of Gastrointestinal Cancer*, *53*(3), 571-580. https://doi.org/10.1007/s12029-021-00664-4

Akaoka, M., Haruki, K., Taniai, T., Yanagaki, M., Igarashi, Y., Furukawa, K., Onda, S., Tsunematsu, M., Shirai, Y., Okui, N., Gocho, T., & Ikegami, T. (2022). Clinical significance of cachexia index in patients with hepatocellular carcinoma after hepatic resection. *Surgical oncology*, *45*, 101881-101881. https://doi.org/10.1016/j.suronc.2022.101881

Akgul, C., Colapkulu-Akgul, N., & Gunes, A. (2024). Prognostic Value of Systemic Inflammatory Markers and Scoring Systems in Predicting Postoperative 30-Day Complications and Mortality in Colorectal Cancer Surgery: A Retrospective Cross-Sectional Analysis. *Ann Ital Chir*, *95*(4), 636-647. https://doi.org/10.62713/aic.3287 10.62713/aic.3287.

Akgül, O., Bagante, F., Weiss, M., Merath, K., Alexandrescu, S., Marques, H. P., Aldrighetti, L., Maithel, S. K., Pulitano, C., Bauer, T. W., Shen, F., Poultsides, G., Soubrane, O., Martel, G., Koerkamp, B. G., Guglielmi, A., Itaru, E., & Pawlik, T. M. (2018). Preoperative prognostic nutritional index predicts survival of patients with intrahepatic cholangiocarcinoma after curative resection [Conference Abstract]. *Annals of Surgical Oncology*, *25*(1), S135. https://doi.org/10.1245/s10434-018-6349-1

Akgül, Ö., Bagante, F., Olsen, G., Cloyd, J. M., Weiss, M., Merath, K., Alexandrescu, S., Marques, H. P., Aldrighetti, L., Maithel, S. K., Pulitano, C., Bauer, T. W., Shen, F., Poultsides, G. A., Soubrane, O., Martel, G., Koerkamp, B. G., Guglielmi, A., Itaru, E., & Pawlik, T. M. (2018). Preoperative prognostic nutritional index predicts survival of patients with intrahepatic cholangiocarcinoma after curative resection. *J Surg Oncol*, *118*(3), 422-430. https://doi.org/10.1002/jso.25140

A-Lai, G. H., Deng, H. Y., Song, T. N., Luo, J., Zhuo, Z. G., Shen, X., & Lin, Y. D. (2019). Preoperative prognostic nutritional index shows no significant prognostic value for short-term outcomes of anastomosis-leakage patients

after cancerous esophagectomy [Article]. *Annals of palliative medicine*, *8*(5), 698-707. https://doi.org/10.21037/apm.2019.11.08

Alkan, Ş. B., Artaç, M., & Rakıcıoğlu, N. (2018). The relationship between nutritional status and handgrip strength in adult cancer patients: a cross-sectional study [Article]. *Supportive Care in Cancer*, *26*(7), 2441-2451. https://doi.org/10.1007/s00520-018-4082-8

Almasaudi, A. S., Dolan, R. D., Edwards, C. A., & McMillan, D. C. (2020). Hypoalbuminemia reflects nutritional risk, body composition and systemic inflammation and is independently associated with survival in patients with colorectal cancer [Article]. *Cancers*, *12*(7), 1-14. https://doi.org/10.3390/cancers12071986

Almasaudi, A. S., McSorley, S. T., Dolan, R. D., Edwards, C. A., & McMillan, D. C. (2019). The relation between Malnutrition Universal Screening Tool (MUST), computed tomography-derived body composition, systemic inflammation, and clinical outcomes in patients undergoing surgery for colorectal cancer [Article]. *American Journal of Clinical Nutrition*, *110*(6), 1327-1334. https://doi.org/10.1093/ajcn/nqz230

Alves, M. J., Figuerêdo, R. G., Azevedo, F. F., Cavallaro, D. A., Neto, N. I. P., Lima, J. D. C., Matos-Neto, E., Radloff, K., Riccardi, D. M., Camargo, R. G., De Alcântara, P. S. M., Otoch, J. P., Junior, M. L. B., & Seelaender, M. (2017). Adipose tissue fibrosis in human cancer cachexia: The role of TGFβ pathway [Article]. *BMC Cancer*, *17*(1). https://doi.org/10.1186/s12885-017-3178-8

Amano, T., Akiyoshi, T., Furuta, M., Saino, Y., Mukai, T., Hiyoshi, Y., Nagasaki, T., Yamaguchi, T., Kawachi, H., & Fukunaga, Y. (2023). Geriatric nutritional risk index after neoadjuvant chemoradiotherapy and survival in older patients with advanced rectal cancer. *Int J Colorectal Dis*, *38*(1), 119. https://doi.org/10.1007/s00384-023-04425-6 10.1007/s00384-023-04425-6.

Attar, A., Malka, D., Sabaté, J. M., Bonnetain, F., Lecomte, T., Aparicio, T., Locher, C., Laharie, D., Ezenfis, J., & Taieb, J. (2012). Malnutrition is high and underestimated during chemotherapy in gastrointestinal cancer: an AGEO prospective cross-sectional multicenter study. *Nutr Cancer*, *64*(4), 535-542. https://doi.org/10.1080/01635581.2012.670743

Bachmann, J., Büchler, M. W., Friess, H., & Martignoni, M. E. (2013). Cachexia in patients with chronic pancreatitis and pancreatic cancer: impact on survival and outcome. *Nutr Cancer*, *65*(6), 827-833. https://doi.org/10.1080/01635581.2013.804580

Bachmann, J., Heiligensetzer, M., Krakowski-Roosen, H., Büchler, M. W., Friess, H., & Martignoni, M. E. (2008). Cachexia worsens prognosis in patients with resectable pancreatic cancer. *J Gastrointest Surg*, *12*(7), 1193-1201. https://doi.org/10.1007/s11605-008-0505-z

Bachmann, J., Ketterer, K., Marsch, C., Fechtner, K., Krakowski-Roosen, H., Büchler, M. W., Friess, H., & Martignoni, M. E. (2009). Pancreatic cancer related cachexia: influence on metabolism and correlation to weight loss and pulmonary function. *BMC Cancer*, *9*, 255. https://doi.org/10.1186/1471-2407-9-255

Bai, X., & Feng, L. (2020). Correlation between Prognostic Nutritional Index, Glasgow Prognostic Score, Systemic Inflammatory Response, and TNM Staging in Colorectal Cancer Patients. *Nutr Cancer*, *72*(7), 1170-1177. https://doi.org/10.1080/01635581.2019.1675725

Bailón-Cuadrado, M., Pérez-Saborido, B., Sánchez-González, J., Rodríguez-López, M., Velasco-López, R., C Sarmentero-Prieto, J., I Blanco-Álvarez, J., & Pacheco-Sánchez, D. (2019). Prognostic Nutritional Index predicts morbidity after curative surgery for colorectal cancer [Article]. *Cirugia espanola*, *97*(2), 71-80. https://doi.org/10.1016/j.ciresp.2018.08.015

Barbosa, L. R. L. S., Lacerda-Filho, A., & Barbosa, L. C. L. S. (2014). Immediate preoperative nutritional status of patients with colorectal cancer: A warning [Article]. *Arquivos de Gastroenterologia*, *51*(4), 331-336. https://doi.org/10.1590/S0004-28032014000400012

Bardakci, M., Hafizoglu, E., Kos, F. T., & Uncu, D. (2021). Does Prognostic Nutritional Index Predict Survival in Operated Papilla Vateri Tumors? A Single-centre Experience. *J Coll Physicians Surg Pak*, *31*(12), 1428-1432.

https://doi.org/10.29271/jcpsp.2021.12.1428

Barret, M., Malka, D., Aparicio, T., Dalban, C., Locher, C., Sabate, J. M., Louafi, S., Mansourbakht, T., Bonnetain, F., Attar, A., & Taieb, J. (2011). Nutritional status affects treatment tolerability and survival in metastatic colorectal cancer patients: results of an AGEO prospective multicenter study. *Oncology*, *81*(5-6), 395-402. https://doi.org/10.1159/000335478

Batista, M. L., Henriques, F. S., Neves, R. X., Olivan, M. R., Matos-Neto, E. M., Alcântara, P. S. M., Maximiano, L. F., Otoch, J. P., Alves, M. J., & Seelaender, M. (2016). Cachexia-associated adipose tissue morphological rearrangement in gastrointestinal cancer patients [Article]. *Journal of Cachexia, Sarcopenia and Muscle*, *7*(1), 37-47. https://doi.org/10.1002/jcsm.12037

Batista, M. L., Jr., Olivan, M., Alcantara, P. S., Sandoval, R., Peres, S. B., Neves, R. X., Silverio, R., Maximiano, L. F., Otoch, J. P., & Seelaender, M. (2013). Adipose tissue-derived factors as potential biomarkers in cachectic cancer patients. *Cytokine*, *61*(2), 532-539. https://doi.org/10.1016/j.cyto.2012.10.023

Bicakli, D. H., Uslu, R., Güney, S. C., & Coker, A. (2020). The Relationship Between Nutritional Status, Performance Status, and Survival Among Pancreatic Cancer Patients. *Nutrition and Cancer*, *72*(2), 202-208. https://doi.org/10.1080/01635581.2019.1634217

Bo, S., Dianliang, Z., Hongmei, Z., Xinxiang, W., Yanbing, Z., & Xiaobo, L. (2010). Association of interleukin-8 gene polymorphism with cachexia from patients with gastric cancer. *J Interferon Cytokine Res*, *30*(1), 9-14. https://doi.org/10.1089/jir.2009.0007

Bo, Y., Wang, K., Liu, Y., You, J., Cui, H., Zhu, Y., Lu, Q., & Yuan, L. (2016). The Geriatric Nutritional Risk Index Predicts Survival in Elderly Esophageal Squamous Cell Carcinoma Patients with Radiotherapy. *PLoS One*, *11*(5), e0155903. https://doi.org/10.1371/journal.pone.0155903

Bo, Y., Yao, M., Zhang, L., Bekalo, W., Lu, W., & Lu, Q. (2015). Preoperative Nutritional Risk Index to predict postoperative survival time in primary liver cancer patients. *Asia Pac J Clin Nutr*, *24*(4), 591-597. https://doi.org/10.6133/apjcn.2015.24.4.26

Borda, F., Borda, A., Zozaya, J. M., Urman, J., Jiménez, J., & Ibáñez, B. (2014). [Prognostic value of Onodera's index in colorectal cancer survival]. *An Sist Sanit Navar*, *37*(2), 213-221. https://doi.org/10.4321/s1137-66272014000200004

Borda, F., Miranda, C., Borda, A., Echeverría, E., Guerra, A., Iñigo, J. J., & Zozaya, J. M. (2017). [Relation between preoperative prognostic Onodera's Index and postsurgery complications in the R0 gastric carcinoma resection]. *An Sist Sanit Navar*, *40*(1), 67-75. https://doi.org/10.23938/assn.0007

Brown, D. R., Berkowitz, D. E., & Breslow, M. J. (2001). Weight Loss Is Not Associated with Hyperleptinemia in Humans with Pancreatic Cancer. *The Journal of Clinical Endocrinology & Metabolism*, *86*(1), 162-166. https://doi.org/10.1210/jcem.86.1.7104

Brown, L., Thomson, G., Gardner, E., Chien, S., Crumley, A., & Skipworth, R. (2024). 'Cachexia Index' for Prognostication in Surgical Patients with Locally Advanced Oesophageal or Gastric Cancer: A Multicentre Cohort Study [Conference Abstract]. *British Journal of Surgery*, *111*, viii1. https://doi.org/10.1093/bjs/znae197.002

Brown, L. R., Soupashi, M., Yule, M. S., Grossart, C. M., McMillan, D. C., Laird, B. J. A., Wigmore, S. J., & Skipworth, R. J. E. (2025). A Comparison of Established Diagnostic Criteria for Cachexia and Their Impacts on Prognostication in Patients with Oesophagogastric Cancer. *Cancers (Basel)*, *17*(3). https://doi.org/10.3390/cancers17030448 10.3390/cancers17030448.

Brown, L. R., Thomson, G. G., Gardner, E., Chien, S., McGovern, J., Dolan, R. D., McSorley, S. T., Forshaw, M. J., McMillan, D. C., Wigmore, S. J., Crumley, A. B., & Skipworth, R. J. E. (2024). Cachexia index for prognostication in surgical patients with locally advanced oesophageal or gastric cancer: multicentre cohort study. *Br J Surg*, *111*(4). https://doi.org/10.1093/bjs/znae098

Cai, W., Yang, H., Zheng, J., Huang, J., Ji, W., Lu, Y., Yang, X., Zhang, W., Shen, X., & Chen, X. (2022). Global

leaders malnutrition initiative-defined malnutrition affects long-term survival of different subgroups of patients with gastric cancer: A propensity score-matched analysis. *Frontiers in nutrition*, *9*, 995295-NA. https://doi.org/10.3389/fnut.2022.995295

Campos, S. B. G., de Azevedo, S. C. L., Gomes, T., Bueno, N. B., Goulart, M. O. F., & Moura, F. A. (2022). Lack of Concordance among Nutritional Diagnostic Methods in Newly Diagnosed Colorectal Cancer Patients. *Nutr Cancer*, *74*(6), 2067-2074. https://doi.org/10.1080/01635581.2021.2001546

Cao, J., Xu, H., Li, W., Guo, Z., Lin, Y., Shi, Y., Hu, W., Ba, Y., Li, S., Li, Z., Wang, K., Wu, J., He, Y., Yang, J., Xie, C., Zhou, F., Song, X., Chen, G., Ma, W., Luo, S., Chen, Z., Cong, M., Ma, H., Zhou, C., Wang, W., Qi, L., Shi, Y., Qi, Y., Jiang, H., Guan, W., Chen, J., Chen, J., Fang, Y., Zhou, L., Feng, Y., Tan, R., Ou, J., Zhao, Q., Wu, J., Xin, L., Yang, L., Fu, Z., Wang, C., Deng, L., Li, T., Song, C., & Shi, H. (2021). Nutritional assessment and risk factors associated to malnutrition in patients with esophageal cancer. *Curr Probl Cancer*, *45*(1), 100638. https://doi.org/10.1016/j.currproblcancer.2020.100638

Cao, X., Zhao, G., Yu, T., An, Q., Yang, H., & Xiao, G. (2017). Preoperative Prognostic Nutritional Index Correlates with Severe Complications and Poor Survival in Patients with Colorectal Cancer Undergoing Curative Laparoscopic Surgery: A Retrospective Study in a Single Chinese Institution. *Nutr Cancer*, *69*(3), 454-463. https://doi.org/10.1080/01635581.2017.1285038

Casalone, V., Bellomo, S. E., Berrino, E., Bo, S., Favaro, E., Mellano, A., Fenocchio, E., Marchiò, C., & Sapino, A. (2025). Clinical significance of preoperative nutrition and inflammation assessment tools in gastrointestinal cancer patients undergoing surgery: a retrospective cohort study. *Front Nutr*, *12*, 1551048. https://doi.org/10.3389/fnut.2025.1551048 10.3389/fnut.2025.1551048. eCollection 2025.

Chai, V. W., Chia, M., Cocco, A., Bhamidipaty, M., & D'Souza, B. (2021). Sarcopenia is a strong predictive factor of clinical and oncological outcomes following curative colorectal cancer resection. *ANZ J Surg*, *91*(5), E292-e297. https://doi.org/10.1111/ans.16706

Chan, A. W., Chan, S. L., Wong, G. L., Wong, V. W., Chong, C. C., Lai, P. B., Chan, H. L., & To, K. F. (2015). Prognostic Nutritional Index (PNI) Predicts Tumor Recurrence of Very Early/Early Stage Hepatocellular Carcinoma After Surgical Resection. *Ann Surg Oncol*, *22*(13), 4138-4148. https://doi.org/10.1245/s10434-015-4516-1

Chang, J. S., Cheng, H. H., Huang, S. C., Lin, H. H., Chang, S. C., & Lin, C. C. (2023). The impact of inflammatory markers on prognosis of stage II colon cancers depends on tumour sidedness. *ANZ J Surg*, *93*(1-2), 182-195. https://doi.org/10.1111/ans.18014 10.1111/ans.18014. Epub 2022 Sep 12.

Chang, L., Cheng, Q., Ma, Y., Wu, C., Zhang, X., Ma, Q., He, L., Li, Q., & Tao, J. (2022). Prognostic Effect of the Controlling Nutritional Status Score in Patients With Esophageal Cancer Treated With Immune Checkpoint Inhibitor. *J Immunother*, *45*(9), 415-422. https://doi.org/10.1097/cji.0000000000000438

Chang, L., Zhang, X., & Li, Q. (2023). The prognostic value of the controlling nutritional status (CONUT) score in predicting outcomes of esophageal cancer patients receiving radiotherapy with or without chemotherapy. *Transl Cancer Res*, *12*(12), 3618-3628. https://doi.org/10.21037/tcr-23-1193

Chen, F. F., Zhang, F. Y., Zhou, X. Y., Shen, X., Yu, Z., & Zhuang, C. L. (2016). Role of frailty and nutritional status in predicting complications following total gastrectomy with D2 lymphadenectomy in patients with gastric cancer: a prospective study. *Langenbecks Arch Surg*, *401*(6), 813-822. https://doi.org/10.1007/s00423-016-1490-4

Chen, K., Li, G., Qiu, Y., Yang, M., Wang, T., Yang, Y., Qiu, H., Sun, T., & Wang, W. (2024). The role of cholesterol-modified prognostic nutritional index in nutritional status assessment and predicting survival after liver resection for hepatocellular carcinoma. *Biosci Trends*, *18*(4), 388-397. https://doi.org/10.5582/bst.2024.01108 10.5582/bst.2024.01108. Epub 2024 Jul 27.

Chen, L., Sun, H., Zhao, R., Huang, R., Pan, H., Zuo, Y., Zhang, L., Xue, Y., Li, X., & Song, H. (2022). Controlling Nutritional Status (CONUT) Predicts Survival in Gastric Cancer Patients With Immune Checkpoint Inhibitor (PD-1/PD-L1) Outcomes [Article]. *Frontiers in Pharmacology*, *13*. https://doi.org/10.3389/fphar.2022.836958

Chen, L., Zhao, M., Tan, L., & Zhang, Y. (2023). Effects of Five-Step Nutritional Interventions Conducted by a Multidisciplinary Care Team on Gastroenteric Cancer Patients Undergoing Chemotherapy: a Randomized Clinical Trial [Journal article]. *Nutrition and cancer*, *75*(1), 197-206. https://doi.org/10.1080/01635581.2022.2104329

Chen, L. R., Li, Z. J., Cui, H. Y., Cheng, B., Tang, D. N., Zhang, A. Q., Ding, L. L., & Zhu, M. W. (2024). [Comparison of prognosis of elderly patients with gastric and colorectal tumors receiving different nutritional support treatments]. *Zhonghua Yi Xue Za Zhi*, *104*(33), 3130-3135. https://doi.org/10.3760/cma.j.cn112137-20240423-00962 10.3760/cma.j.cn112137-20240423-00962.

Chen, M. F., Hsieh, C. C., Chen, P. T., & Lu, M. S. (2021). Role of Nutritional Status in the Treatment Outcome for Esophageal Squamous Cell Carcinoma. *Nutrients*, *13*(9). https://doi.org/10.3390/nu13092997

Chen, N., Yu, Y., Shen, W., Xu, X., & Fan, Y. (2024). Nutritional status as prognostic factor of advanced oesophageal cancer patients treated with immune checkpoint inhibitors. *Clin Nutr*, *43*(1), 142-153. https://doi.org/10.1016/j.clnu.2023.11.030 10.1016/j.clnu.2023.11.030. Epub 2023 Nov 27.

Chen, S. B., Liu, D. T., & Chen, Y. P. (2021). The Impact of Preoperative Nutritional Status on the Survival of Patients With Esophageal Squamous Cell Carcinoma. *Front Surg*, *8*, 752792. https://doi.org/10.3389/fsurg.2021.752792

Chen, W., Yu, D., Ren, Q., Shen, Z., Huang, G., Chen, X., Dong, Q., & Yu, Z. (2024). Predictive value of Global Leadership Initiative on Malnutrition criteria combined with handgrip strength for postoperative outcomes in overweight colorectal cancer patients. *J Gastroenterol Hepatol*, *39*(4), 716-724. https://doi.org/10.1111/jgh.16481 10.1111/jgh.16481. Epub 2024 Jan 11.

Chen, W., Zhang, M., Chen, C., & Pang, X. (2022). Prognostic Nutritional Index and Neutrophil/Lymphocyte Ratio Can Serve as Independent Predictors of the Prognosis of Hepatocellular Carcinoma Patients Receiving Targeted Therapy [Article]. *Journal of Oncology*, *2022*. https://doi.org/10.1155/2022/1389049

Chen, W. Z., Zhang, X. Z., Zhang, F. M., Yu, D. Y., Chen, W. H., Lin, F., Dong, Q. T., Zhuang, C. L., & Yu, Z. (2022). Coexistence of GLIM-defined malnutrition and sarcopenia have negative effect on the clinical outcomes in the elderly gastric cancer patients after radical gastrectomy. *Front Nutr*, *9*, 960670. https://doi.org/10.3389/fnut.2022.960670

Chen, X. (2022). A novel nomogram based on the nutritional risk screening 2002 score to predict survival in hepatocellular carcinoma treated with transarterial chemoembolization. *Nutr Hosp*, *39*(4), 835-842. https://doi.org/10.20960/nh.03983

Chen, X., Zeng, Y., Huang, Y., Xu, J., Meng, W., Wang, X., Zhu, C., Zhu, G., Mao, C., & Shen, X. (2019). Preoperative Cachexia predicts poor outcomes in young rather than elderly gastric cancer patients: a prospective study. *Cancer Manag Res*, *11*, 8101-8110. https://doi.org/10.2147/cmar.S213237

Chen, X. Y., Li, B., Ma, B. W., Zhang, X. Z., Chen, W. Z., Lu, L. S., Shen, X., Zhuang, C. L., & Yu, Z. (2019). Sarcopenia is an effective prognostic indicator of postoperative outcomes in laparoscopic-assisted gastrectomy. *Eur J Surg Oncol*, *45*(6), 1092-1098. https://doi.org/10.1016/j.ejso.2018.09.030 10.1016/j.ejso.2018.09.030. Epub 2019 Feb 6.

Chen, Y., Xu, W. J., Yang, Y., Xin, Y. J., Zhang, X. Y., Li, X., & Zhou, X. (2022). Nomograms including the controlling nutritional status score in patients with hepatocellular carcinoma undergoing transarterial chemoembolisation for prediction survival: a retrospective analysis. *Br J Nutr*, *128*(10), 1966-1974. https://doi.org/10.1017/s000711452100492x

Chen, Y., Zhao, C., Yang, Y., Xin, Y. J., Wang, Y. N., Li, X., Zhou, X., & Feng, D. P. (2020). Using the Controlling Nutritional Status (CONUT) Score for Evaluating Patients with Early-Stage Hepatocellular Carcinoma After Radiofrequency Ablation: A Two-Center Retrospective Study. *Cardiovasc Intervent Radiol*, *43*(9), 1294-1304. https://doi.org/10.1007/s00270-020-02519-0

Cho, J. W., Youn, J., Kim, E. M., Choi, M. G., & Lee, J. E. (2022). Associations of patient-generated subjective

global assessment (PG-SGA) and NUTRISCORE with survival in gastric cancer patients: timing matters, a retrospective cohort study. *BMC Gastroenterol*, *22*(1), 468. https://doi.org/10.1186/s12876-022-02515-3

Choi, Y., Kim, J. W., Nam, K. H., Han, S. H., Kim, J. W., Ahn, S. H., Park, D. J., Lee, K. W., Lee, H. S., & Kim, H. H. (2017). Systemic inflammation is associated with the density of immune cells in the tumor microenvironment of gastric cancer. *Gastric Cancer*, *20*(4), 602-611. https://doi.org/10.1007/s10120-016-0642-0

Chu, M. O., Shen, C. H., Chang, T. S., Xu, H. W., Yen, C. W., Lu, S. N., & Hung, C. H. (2018). Pretreatment Inflammation-Based Markers Predict Survival Outcomes in Patients with Early Stage Hepatocellular Carcinoma After Radiofrequency Ablation. *Sci Rep*, *8*(1), 16611. https://doi.org/10.1038/s41598-018-34543-z

da Silva Couto, A., Gonzalez, M. C., Martucci, R. B., Feijó, P. M., Rodrigues, V. D., de Pinho, N. B., & Souza, N. C. (2023). Predictive validity of GLIM malnutrition diagnosis in patients with colorectal cancer. *JPEN J Parenter Enteral Nutr*. https://doi.org/10.1002/jpen.2475

Dai, T., Wu, D., Tang, J., Liu, Z., & Zhang, M. (2023). Construction and validation of a predictive model for the risk of three-month-postoperative malnutrition in patients with gastric cancer: a retrospective case-control study. *J Gastrointest Oncol*, *14*(1), 128-145. https://doi.org/10.21037/jgo-22-1307 10.21037/jgo-22-1307. Epub 2023 Feb 15.

Dai, Y., Fu, X., Li, T., Yao, Q., Su, L., Su, H., & Li, J. (2019). Long-term impact of prognostic nutritional index in cervical esophageal squamous cell carcinoma patients undergoing definitive radiotherapy. *Ann Transl Med*, *7*(8), 175. https://doi.org/10.21037/atm.2019.03.60

Daitoku, N., Miyamoto, Y., Tokunaga, R., Sakamoto, Y., Hiyoshi, Y., Iwatsuki, M., Baba, Y., Iwagami, S., Yoshida, N., & Baba, H. (2018). Controlling Nutritional Status (CONUT) Score Is a Prognostic Marker in Metastatic Colorectal Cancer Patients Receiving First-line Chemotherapy. *Anticancer Res*, *38*(8), 4883-4888. https://doi.org/10.21873/anticanres.12802

D'Almeida, C. A., Pinho, N. B., Martucci, R. B., & Peres, W. A. F. (2013). Weight loss and postoperative complications in abdominal cancer patients [Conference Abstract]. *Clinical Nutrition*, *32*, S182-S183.

Daly, L. E., Ní Bhuachalla, É. B., Power, D. G., Cushen, S. J., James, K., & Ryan, A. M. (2018). Loss of skeletal muscle during systemic chemotherapy is prognostic of poor survival in patients with foregut cancer [Article]. *Journal of Cachexia, Sarcopenia and Muscle*, *9*(2), 315-325. https://doi.org/10.1002/jcsm.12267

Dang, C., Wang, M., Qin, T., & Qin, R. (2022). Clinical importance of preoperative red-cell volume distribution width as a prognostic marker in patients undergoing radical surgery for pancreatic cancer. *Surg Today*, *52*(3), 465-474. https://doi.org/10.1007/s00595-021-02374-7

Dang, C., Wang, M., Zhu, F., Qin, T., & Qin, R. (2022). Controlling nutritional status (CONUT) score-based nomogram to predict overall survival of patients with pancreatic cancer undergoing radical surgery. *Asian J Surg*, *45*(6), 1237-1245. https://doi.org/10.1016/j.asjsur.2021.08.011

Daniele, A., Divella, R., Abbate, I., Casamassima, A., Garrisi, V. M., Savino, E., Casamassima, P., Ruggieri, E., & De Luca, R. (2017). Assessment of nutritional and inflammatory status to determine the prevalence of malnutrition in patients undergoing surgery for colorectal carcinoma [Article]. *Anticancer Research*, *37*(3), 1281-1287. https://doi.org/10.21873/anticanres.11445

de Castro, G. S., Correia-Lima, J., Simoes, E., Orsso, C. E., Xiao, J., Gama, L. R., Gomes, S. P., Gonçalves, D. C., Costa, R. G. F., Radloff, K., Lenz, U., Taranko, A. E., Bin, F. C., Formiga, F. B., de Godoy, L. G. L., de Souza, R. P., Nucci, L. H. A., Feitoza, M., de Castro, C. C., Tokeshi, F., Alcantara, P. S. M., Otoch, J. P., Ramos, A. F., Laviano, A., Coletti, D., Mazurak, V. C., Prado, C. M., & Seelaender, M. (2021). Myokines in treatment-naïve patients with cancer-associated cachexia [Article]. *Clinical Nutrition*, *40*(4), 2443-2455. https://doi.org/10.1016/j.clnu.2020.10.050

de Luis Roman, D., López Gómez, J. J., Muñoz, M., Primo, D., Izaola, O., & Sánchez, I. (2024). Evaluation of Muscle Mass and Malnutrition in Patients with Colorectal Cancer Using the Global Leadership Initiative on Malnutrition Criteria and Comparing Bioelectrical Impedance Analysis and Computed Tomography Measurements.

Nutrients, *16*(17). https://doi.org/10.3390/nu16173035 10.3390/nu16173035.

Deliktaş Onur, İ., Fırat, H. G., Sertesen Çamöz, E., & Yildiz, F. (2024). Is the Prognostic Nutritional Index a Novel Prognostic Factor in Patients With Unresectable/Metastatic Gallbladder and Cholangiocarcinoma Receiving Chemotherapy? *Cureus*, *16*(7), e65003. https://doi.org/10.7759/cureus.65003 10.7759/cureus.65003. eCollection 2024 Jul.

Demir, M., & Demircan, N. C. (2023). The CONUT score is prognostic in esophageal cancer treated with chemoradiotherapy. *Saudi J Gastroenterol*, *29*(2), 119-126. https://doi.org/10.4103/sjg.sjg_384_22 10.4103/sjg.sjg_384_22.

Diakowska, D., Krzystek-Korpacka, M., Markocka-Maczka, K., Diakowski, W., Matusiewicz, M., & Grabowski, K. (2010). Circulating leptin and inflammatory response in esophageal cancer, esophageal cancer-related cachexia-anorexia syndrome (CAS) and non-malignant CAS of the alimentary tract. *Cytokine*, *51*(2), 132-137. https://doi.org/10.1016/j.cyto.2010.05.006

Dijksterhuis, W. P. M., Latenstein, A. E. J., van Kleef, J. J., Verhoeven, R. H. A., de Vries, J. H. M., Slingerland, M., Steenhagen, E., Heisterkamp, J., Timmermans, L. M., de van der Schueren, M. A. E., van Oijen, M. G. H., Beijer, S., & van Laarhoven, H. W. M. (2021). Cachexia and Dietetic Interventions in Patients With Esophagogastric Cancer: A Multicenter Cohort Study. *J Natl Compr Canc Netw*, *19*(2), 144-152. https://doi.org/10.6004/jnccn.2020.7615

Dolin, T. G., Mikkelsen, M. K., Jakobsen, H. L., Vinther, A., Zerahn, B., Nielsen, D. L., Johansen, J. S., Lund, C. M., & Suetta, C. (2022). The prevalence of sarcopenia and cachexia in older patients with localized colorectal cancer [Article in Press]. *Journal of Geriatric Oncology*. https://doi.org/10.1016/j.jgo.2022.11.001

Dongdong, H., Wu, G.-F., Luo, X., Song, H.-N., Wang, W.-B., Liu, N.-X., Zhen, Y., Dong, Q.-T., Chen, X.-L., & Yan, J.-Y. (2021). Value of muscle quality, strength and gait speed in supporting the predictive power of GLIM-defined malnutrition for postoperative outcomes in overweight patients with gastric cancer. *Clinical nutrition (Edinburgh, Scotland)*, *40*(6), 4201-4208. https://doi.org/10.1016/j.clnu.2021.01.038

Dou, L., Wang, X., Cao, Y., Hu, A., & Li, L. (2020). Relationship between Postoperative Recovery and Nutrition Risk Screened by NRS 2002 and Nutrition Support Status in Patients with Gastrointestinal Cancer. *Nutr Cancer*, *72*(1), 33-40. https://doi.org/10.1080/01635581.2019.1612927

Du, Y., Li, Y., Tan, Z., Song, J., Jiang, Y., Liu, S., Guo, Y., Qiao, Y., Zhu, J., Li, S., & Li, J. (2025). Prognostic value of combining preoperative immune-inflammatory-nutritional index and tumor biomarkers in gastric cancer patients undergoing radical resection. *Front Nutr*, *12*, 1562202. https://doi.org/10.3389/fnut.2025.1562202 10.3389/fnut.2025.1562202. eCollection 2025.

Duan, H., Zhang, J., Wang, P., Zhang, J., & Jiang, J. (2023). Association between nutritional status and platelet-to-lymphocyte ratio in patients with hepatocellular carcinoma undergoing transcatheter arterial chemoembolization. *Nutr Hosp*, *40*(5), 1009-1016. https://doi.org/10.20960/nh.04447 10.20960/nh.04447. (Asociación entre el estado nutricional y el índice plaquetas-linfocitos en pacientes con carcinoma hepatocelular sometidos a quimioembolización transarterial.)

Eo, W. K., Chang, H. J., Suh, J., Ahn, J., Shin, J., Hur, J. Y., Kim, G. Y., Lee, S., Park, S., & Lee, S. (2015). The Prognostic Nutritional Index Predicts Survival and Identifies Aggressiveness of Gastric Cancer. *Nutr Cancer*, *67*(8), 1260-1267. https://doi.org/10.1080/01635581.2015.1082112

Erdogan, B., Ozcan, E., Gokmen, I., Gokyer, A., Kucukarda, A., Kostek, O., Hacioglu, M., Uzunoglu, S., & Cicin, I. (2023). Relationship between prognostic nutritional index and neutrophil lymphocyte ratio with overall survival in patients with metastatic colorectal cancer receiving regorafenib [Article]. *Journal of Cancer Research and Therapeutics*, *19*(3), 762-767. https://doi.org/10.4103/jcrt.jcrt_1620_21

Ericson, J., Klevebro, F., Sunde, B., Szabo, E., Halldestam, I., Smedh, U., Wallner, B., Johansson, J., Johnsen, G., Aahlin, E. K., & et al. (2025). Nutritional outcomes and impact of malnutrition in a randomised comparison between standard and prolonged time to surgery after neoadjuvant chemoradiotherapy for oesophageal cancer

[Journal article]. *European journal of surgical oncology*, *51*(9), 110228. https://doi.org/10.1016/j.ejso.2025.110228

Esfahani, A., Makhdami, N., Faramarzi, E., Asghari Jafarabadi, M., Ostadrahimi, A., Ghayour Nahand, M., & Ghoreishi, Z. (2016). Prealbumin/CRP Based Prognostic Score, a New Tool for Predicting Metastasis in Patients with Inoperable Gastric Cancer. *Gastroenterol Res Pract*, *2016*, 4686189. https://doi.org/10.1155/2016/4686189

Fan, Z., Wu, M., Tang, Z., He, A., Liu, F., Liang, W., Wang, Z., & Yang, D. (2022). Predictive Value of Platelet-Related Measures in Patients with Hepatocellular Carcinoma. *Technol Cancer Res Treat*, *21*, 15330338211064414. https://doi.org/10.1177/15330338211064414

Fang, P., Yang, Q., Zhou, J., Yang, Y., Luan, S., Xiao, X., Li, X., Gu, Y., Shang, Q., Zhang, H., Chen, L., Zeng, X., & Yuan, Y. (2022). The impact of geriatric nutritional risk index on esophageal squamous cell carcinoma patients with neoadjuvant therapy followed by esophagectomy. *Front Nutr*, *9*, 983038. https://doi.org/10.3389/fnut.2022.983038

Fang, P., Zhou, J., Liang, Z., Yang, Y., Luan, S., Xiao, X., Li, X., Shang, Q., Zhang, H., Zeng, X., & Yuan, Y. (2024). The prognostic value of controlling nutritional status score on esophageal squamous cell carcinoma patients with neoadjuvant therapy followed by esophagectomy-a retrospective research. *J Thorac Dis*, *16*(7), 4460-4473. https://doi.org/10.21037/jtd-24-187

Feng, J., Wang, L., Yang, X., Chen, Q., & Cheng, X. (2022). The usefulness of pretreatment controlling nutritional status score for predicting recurrence in patients with esophageal squamous cell carcinoma undergoing neoadjuvant immunochemotherapy: A real-world study. *Front Immunol*, *13*, 1015365. https://doi.org/10.3389/fimmu.2022.1015365

Feng, J., Wang, L., Yang, X., Chen, Q., & Cheng, X. (2024). Clinical significance of geriatric nutritional risk index in esophageal squamous cell carcinoma receiving neoadjuvant immunotherapy. *Eur J Surg Oncol*, *50*(6), 108323. https://doi.org/10.1016/j.ejso.2024.108323

Feng, J. F., & Chen, Q. X. (2014). Significance of the prognostic nutritional index in patients with esophageal squamous cell carcinoma. *Ther Clin Risk Manag*, *10*, 1-7. https://doi.org/10.2147/tcrm.S56159

Findlay, M., Purvis, M., Venman, R., Luong, R., & Carey, S. (2020). Nutritional management of patients with oesophageal cancer throughout the treatment trajectory: benchmarking against best practice. *Support Care Cancer*, *28*(12), 5963-5971. https://doi.org/10.1007/s00520-020-05416-x

Fruchtenicht, A. V. G., Poziomyck, A. K., Reis, A. M. D., Galia, C. R., Kabke, G. B., & Moreira, L. F. (2018). Inflammatory and nutritional statuses of patients submitted to resection of gastrointestinal tumors. *Rev Col Bras Cir*, *45*(2), e1614. https://doi.org/10.1590/0100-6991e-20181614

Fu, K., & Pan, H. (2017). Nutritional status and risk factors for malnutrition in CRC patients undergoing neoadjuvant therapy [Article]. *Biomedical Research (India)*, *28*(10), 4406-4412.

Fu, L., Song, L., Zhou, X., Chen, L., Zheng, L., Hu, D., Zhu, S., Hu, Y., Gong, D., Chen, C. L., Ye, X., & Yu, S. (2024). Serum metabolomics analysis of malnutrition in patients with gastric cancer: a cross sectional study. *BMC Cancer*, *24*(1), 1195. https://doi.org/10.1186/s12885-024-12964-6

Fugane, Y., Tanaka, S., Mizuno, Y., Nakajima, H., Yamamoto, H., Inoue, T., Nagaya, M., Nishida, Y., Onoe, S., Yamaguchi, J., Mizuno, T., Yokoyama, Y., & Ebata, T. (2025). Prognostic impact of preoperative cachexia in patients undergoing major hepatopancreatobiliary surgery for malignancy. *Clin Nutr*, *47*, 112-118. https://doi.org/10.1016/j.clnu.2025.02.014

Fujii, H., Makiyama, A., Iihara, H., Okumura, N., Yamamoto, S., Imai, T., Arakawa, S., Kobayashi, R., Tanaka, Y., Yoshida, K., & Suzuki, A. (2020). Cancer Cachexia Reduces the Efficacy of Nivolumab Treatment in Patients With Advanced Gastric Cancer. *Anticancer Res*, *40*(12), 7067-7075. https://doi.org/10.21873/anticanres.14734

Fujio, A., Usuda, M., Hara, Y., Kakizaki, Y., Okada, K., Miyata, G., Unno, M., & Kamei, T. (2022). Usefulness of Preoperative Controlling Nutritional Status in Predicting Prolonged Hospitalization and Incidence of Postoperative Delirium for Elderly Hepatectomy with Hepatocellular Carcinoma. *The Tohoku Journal of Experimental Medicine*,

256(1), 43-52.

Fujiwara, Y., Endo, S., Higashida, M., Kubota, H., Yoshimatsu, K., & Ueno, T. (2023). The prognostic significance of preoperative nutritional/inflammatory markers and clinicopathological features in resectable esophagectomy patients: possibility of nutritional intervention. *Esophagus*, *20*(2), 234-245. https://doi.org/10.1007/s10388-022-00961-2

Fujiwara, Y., Higashida, M., Kubota, H., Okamoto, Y., Mineta, S., Endo, S., & Ueno, T. (2021). Perioperative Predictive Markers for Recurrence of Esophageal Cancer after Esophagectomy. *Gastrointestinal Tumors*, *8*(2), 87-95. https://doi.org/10.1159/000513961

Fujiwara, Y., Kobayashi, T., Chayahara, N., Imamura, Y., Toyoda, M., Kiyota, N., Mukohara, T., Nishiumi, S., Azuma, T., Yoshida, M., & Minami, H. (2014). Metabolomics Evaluation of Serum Markers for Cachexia and Their Intra-Day Variation in Patients with Advanced Pancreatic Cancer. *PLOS ONE*, *9*(11), e113259. https://doi.org/10.1371/journal.pone.0113259

Fujiya, K., Kawamura, T., Omae, K., Makuuchi, R., Irino, T., Tokunaga, M., Tanizawa, Y., Bando, E., & Terashima, M. (2018). Impact of Malnutrition After Gastrectomy for Gastric Cancer on Long-Term Survival [Article]. *Annals of Surgical Oncology*, *25*(4), 974-983. https://doi.org/10.1245/s10434-018-6342-8

Fukuda, Y., Yamamoto, K., Hirao, M., Nishikawa, K., Maeda, S., Haraguchi, N., Miyake, M., Hama, N., Miyamoto, A., Ikeda, M., Nakamori, S., Sekimoto, M., Fujitani, K., & Tsujinaka, T. (2015). Prevalence of Malnutrition Among Gastric Cancer Patients Undergoing Gastrectomy and Optimal Preoperative Nutritional Support for Preventing Surgical Site Infections [Article]. *Annals of Surgical Oncology*, *22*, 778-785. https://doi.org/10.1245/s10434-015-4820-9

Fukuta, A., Saito, T., Murata, S., Makiura, D., Inoue, J., Okumura, M., Sakai, Y., & Ono, R. (2019). Impact of preoperative cachexia on postoperative length of stay in elderly patients with gastrointestinal cancer [Article]. *Nutrition*, *58*, 65-68. https://doi.org/10.1016/j.nut.2018.06.022

Funamizu, N., Sakamoto, A., Mori, S., Iwata, M., Shine, M., Ito, C., Uraoka, M., Ueno, Y., Tamura, K., Kamei, Y., Takada, Y., Aoki, T., & Umeda, Y. (2025). Postoperative Geriatric Nutritional Risk Index as a Determinant of Tolerance to S-1 Adjuvant Chemotherapy After Curative Surgery for Pancreatic Ductal Adenocarcinoma: A Cohort Study with External Validation. *Cancers (Basel)*, *17*(9). https://doi.org/10.3390/cancers17091448 10.3390/cancers17091448.

Funamizu, N., Sakamoto, A., Utsunomiya, T., Uraoka, M., Nagaoka, T., Iwata, M., Ito, C., Tamura, K., Sakamoto, K., Ogawa, K., & Takada, Y. (2022). Geriatric nutritional risk index as a potential prognostic marker for patients with resectable pancreatic cancer: a single-center, retrospective cohort study. *Scientific reports*, *12*(1), 13644-NA. https://doi.org/10.1038/s41598-022-18077-z

Furuke, H., Matsubara, D., Kubota, T., Kiuchi, J., Kubo, H., Ohashi, T., Shimizu, H., Arita, T., Yamamoto, Y., Konishi, H., Morimura, R., Shiozaki, A., Kuriu, Y., Ikoma, H., Fujiwara, H., Okamoto, K., & Otsuji, E. (2021). Geriatric Nutritional Risk Index Predicts Poor Prognosis of Patients After Curative Surgery for Gastric Cancer. *Cancer Diagn Progn*, *1*(2), 43-52. https://doi.org/10.21873/cdp.10007

Gabrielson, D. K., Brezden-Masley, C., Keith, M., Bazinet, R. P., Sykes, J., & Darling, P. B. (2021). Evaluation of Nutritional, Inflammatory, and Fatty Acid Status in Patients with Gastric and Colorectal Cancer Receiving Chemotherapy. *Nutr Cancer*, *73*(3), 420-432. https://doi.org/10.1080/01635581.2020.1756351

Gallardo-Valverde, J. M., Calañas-Continente, A., Baena-Delgado, E., Zurera-Tendero, L., Vázquez-Martínez, C., Membrives-Obrero, A., Muntané, J., & Arévalo-Jiménez, E. (2005). Obstruction in patients with colorectal cancer increases morbidity and mortality in association with altered nutritional status. *Nutr Cancer*, *53*(2), 169-176. https://doi.org/10.1207/s15327914nc5302_6

Gao, J., Wang, Y., Li, F., Zhu, Z., Han, B., Wang, R., Xie, R., & Xue, Y. (2019). Prognostic Nutritional Index and Neutrophil-to-Lymphocyte Ratio Are Respectively Associated with Prognosis of Gastric Cancer with Liver Metatasis

Undergoing and without Hepatectomy. *Biomed Res Int*, *2019*, 4213623. https://doi.org/10.1155/2019/4213623

Garth, A. K., Newsome, C. M., Simmance, N., & Crowe, T. C. (2010). Nutritional status, nutrition practices and post-operative complications in patients with gastrointestinal cancer. *J Hum Nutr Diet*, *23*(4), 393-401. https://doi.org/10.1111/j.1365-277X.2010.01058.x

Gavazzi, C., Colatruglio, S., Sironi, A., Mazzaferro, V., & Miceli, R. (2011). Importance of early nutritional screening in patients with gastric cancer. *Br J Nutr*, *106*(12), 1773-1778. https://doi.org/10.1017/s0007114511002509

Ge, K., Fang, C., Zhu, D., Yan, H., Wang, Q., Chen, W., & Wu, J. (2021). The Prognostic Value of the Prognostic Nutritional Index (PNI) in Radically Resected Esophagogastric Junction Adenocarcinoma. *Nutr Cancer*, *73*(11-12), 2589-2596. https://doi.org/10.1080/01635581.2020.1841252

Geng, Y., Qi, Q., Sun, M., Chen, H., Wang, P., & Chen, Z. (2015). Prognostic nutritional index predicts survival and correlates with systemic inflammatory response in advanced pancreatic cancer. *European Journal of Surgical Oncology (EJSO)*, *41*(11), 1508-1514. https://doi.org/https://doi.org/10.1016/j.ejso.2015.07.022

Gillis, C., Richer, L., Fenton, T. R., Gramlich, L., Keller, H., Culos-Reed, S. N., Sajobi, T. T., Awasthi, R., & Carli, F. (2021). Colorectal cancer patients with malnutrition suffer poor physical and mental health before surgery. *Surgery*, *170*(3), 841-847. https://doi.org/10.1016/j.surg.2021.04.003

Gilmore, L. A., Olaechea, S., Gilmore, B. W., Gannavarapu, B. S., Alvarez, C. M., Ahn, C., Iyengar, P., & Infante, R. E. (2022). A preponderance of gastrointestinal cancer patients transition into cachexia syndrome. *J Cachexia Sarcopenia Muscle*, *13*(6), 2920-2931. https://doi.org/10.1002/jcsm.13086

Goh, B. K., Kam, J. H., Lee, S. Y., Chan, C. Y., Allen, J. C., Jeyaraj, P., Cheow, P. C., Chow, P. K., Ooi, L. L., & Chung, A. Y. (2016). Significance of neutrophil-to-lymphocyte ratio, platelet-to-lymphocyte ratio and prognostic nutrition index as preoperative predictors of early mortality after liver resection for huge (≥10 cm) hepatocellular carcinoma. *J Surg Oncol*, *113*(6), 621-627. https://doi.org/10.1002/jso.24197

Goh, M. J., Kang, W., Jeong, W. K., Sinn, D. H., Gwak, G. Y., Paik, Y. H., Choi, M. S., Lee, J. H., Koh, K. C., & Paik, S. W. (2022). Prognostic significance of cachexia index in patients with advanced hepatocellular carcinoma treated with systemic chemotherapy. *Sci Rep*, *12*(1), 7647. https://doi.org/10.1038/s41598-022-11736-1

Gómez Sánchez, M. B., García-Talavera Espín, N. V., Sánchez Álvarez, C., Zomeño Ros, A. I., Hernández, M. N., Gómez Ramos, M. J., Parra Baños, P., & González Valverde, F. M. (2010). Perioperative nutritional support in patients with colorectal neoplasms [Journal article]. *Nutricion hospitalaria*, *25*(5), 797-805. https://www.cochranelibrary.com/central/doi/10.1002/central/CN-00786125/full

Gong, C., Wan, Q., Zhao, R., Zuo, X., Chen, Y., & Li, T. (2022). Cachexia Index as a Prognostic Indicator in Patients with Gastric Cancer: A Retrospective Study [Article]. *Cancers*, *14*(18). https://doi.org/10.3390/cancers14184400

Grinstead, C., & Yoon, S. L. (2025). Geriatric Nutritional Risk Index (GNRI) and Survival in Pancreatic Cancer: A Retrospective Study. *Nutrients*, *17*(3), 509-509. https://doi.org/10.3390/nu17030509

Guo, H., Wang, T., Jiang, X., Wang, J., & Ma, X. (2025). Prognostic nomogram for overall survival in resectable gastric cancer: incorporating prognostic nutritional index and fibrinogen. *Nutr Hosp*, *42*(4), 728-737. https://doi.org/10.20960/nh.05679 10.20960/nh.05679. (Nomograma pronóstico de supervivencia global en cáncer gástrico resecable: incorporación del índice nutricional pronóstico y el fibrinógeno.)

Guo, X. W., Liu, Y. C., Gao, F., Ji, S. J., Zhou, J. Y., Ji, L., & Zhou, S. B. (2018). Pretreatment NRS-2002 scores combined with hematologic inflammation markers are independent prognostic factors in patients with resectable thoracic esophageal squamous cell carcinoma. *Cancer Manag Res*, *10*, 2409-2418. https://doi.org/10.2147/cmar.S167179

Guo, Z. Q., Yu, J. M., Li, W., Fu, Z. M., Lin, Y., Shi, Y. Y., Hu, W., Ba, Y., Li, S. Y., Li, Z. N., Wang, K. H., Wu, J., He, Y., Yang, J. J., Xie, C. H., Song, X. X., Chen, G. Y., Ma, W. J., Luo, S. X., Chen, Z. H., Cong, M. H., Ma, H., Zhou, C. L.,

Wang, W., Luo, Q., Shi, Y. M., Qi, Y. M., Jiang, H. P., Guan, W. X., Chen, J. Q., Chen, J. X., Fang, Y., Zhou, L., Feng, Y. D., Tan, R. S., Li, T., Ou, J. W., Zhao, Q. C., Wu, J. X., Deng, L., Lin, X., Yang, L. Q., Yang, M., Wang, C., Song, C. H., Xu, H. X., & Shi, H. P. (2020). Survey and analysis of the nutritional status in hospitalized patients with malignant gastric tumors and its influence on the quality of life. *Support Care Cancer*, *28*(1), 373-380. https://doi.org/10.1007/s00520-019-04803-3

Gül, M. C., Çolakoğlu, M. K., Öter, V., Karaca, N., Eroğlu, S. E., Ökten, R. S., & Bostancı, E. B. (2025). Low Preoperative Cachexia Index Is Associated with Severe Postoperative Morbidity in Patients Undergoing Gastrectomy for Gastric Cancer. *Diagnostics (Basel)*, *15*(18). https://doi.org/10.3390/diagnostics15182284

Hamura, R., Haruki, K., Shirai, Y., Tanji, Y., Taniai, T., Okui, N., Furukawa, K., Shiozaki, H., Onda, S., & Ikegami, T. (2022). Preoperative cachexia index can predict the prognosis of extrahepatic biliary tract cancer after resection. *Surg Oncol*, *44*, 101825. https://doi.org/10.1016/j.suronc.2022.101825

Han, J., Tang, M., Lu, C., Shen, L., She, J., & Wu, G. (2021). Subcutaneous, but not visceral, adipose tissue as a marker for prognosis in gastric cancer patients with cachexia. *Clin Nutr*, *40*(9), 5156-5161. https://doi.org/10.1016/j.clnu.2021.08.003

Han, L., Song, Q., Jia, Y., Chen, X., Wang, C., Chen, P., Min, R., & Cheng, Y. (2016). The clinical significance of systemic inflammation score in esophageal squamous cell carcinoma. *Tumour Biol*, *37*(3), 3081-3090. https://doi.org/10.1007/s13277-015-4152-1

Han, Y., Wu, J., Ji, R., Tan, H., Tian, S., Yin, J., Xu, J., Chen, X., Liu, W., & Cui, H. (2023). Preoperative sarcopenia combined with prognostic nutritional index predicts long-term prognosis of radical gastrectomy with advanced gastric cancer: a comprehensive analysis of two-center study. *BMC Cancer*, *23*(1), 751. https://doi.org/10.1186/s12885-023-11251-0

Haneda, R., Hiramatsu, Y., Kawata, S., Honke, J., Soneda, W., Matsumoto, T., Morita, Y., Kikuchi, H., Kamiya, K., & Takeuchi, H. (2022). Survival impact of perioperative changes in prognostic nutritional index levels after esophagectomy. *Esophagus*, *19*(2), 250-259. https://doi.org/10.1007/s10388-021-00883-5

Harimoto, N., Tsukagoshi, M., Okuyama, T., Hoshino, K., Hagiwara, K., Kawai, S., Ishii, N., Igarashi, T., Araki, K., & Shirabe, K. (2023). Significance of malnutrition defined with Global Leadership Initiative on Malnutrition criteria in patients with hepatocellular carcinoma after hepatic resection. *Hepatol Res*, *53*(12), 1235-1248. https://doi.org/10.1111/hepr.13959

Harimoto, N., Yoshizumi, T., Inokuchi, S., Itoh, S., Adachi, E., Ikeda, Y., Uchiyama, H., Utsunomiya, T., Kajiyama, K., Kimura, K., Kishihara, F., Sugimachi, K., Tsujita, E., Ninomiya, M., Fukuzawa, K., Maeda, T., Shirabe, K., & Maehara, Y. (2018). Prognostic Significance of Preoperative Controlling Nutritional Status (CONUT) Score in Patients Undergoing Hepatic Resection for Hepatocellular Carcinoma: A Multi-institutional Study. *Ann Surg Oncol*, *25*(11), 3316-3323. https://doi.org/10.1245/s10434-018-6672-6

Harimoto, N., Yoshizumi, T., Sakata, K., Nagatsu, A., Motomura, T., Itoh, S., Harada, N., Ikegami, T., Uchiyama, H., Soejima, Y., & Maehara, Y. (2017). Prognostic Significance of Preoperative Controlling Nutritional Status (CONUT) Score in Patients Undergoing Hepatic Resection for Hepatocellular Carcinoma. *World J Surg*, *41*(11), 2805-2812. https://doi.org/10.1007/s00268-017-4097-1

Hashimoto, I., Kano, K., Suematsu, H., Yamada, T., Watanabe, H., Kanematsu, K., Nagasawa, S., Aoyama, T., Ogata, T., Rino, Y., Saito, A., & Oshima, T. (2024). Survival Predictors Before Preoperative Adjuvant Chemotherapy in Patients With Locally Advanced Esophageal Squamous Cell Carcinoma. *In Vivo*, *38*(2), 881-889. https://doi.org/10.21873/invivo.13514 10.21873/invivo.13514.

Hatada, T., & Miki, C. (2000). Nutritional status and postoperative cytokine response in colorectal cancer patients. *Cytokine*, *12*(9), 1331-1336. https://doi.org/10.1006/cyto.2000.0726

Hatanaka, T., Kakizaki, S., Uehara, D., Nagashima, T., Ueno, T., Namikawa, M., Saito, S., Hosonuma, K., Suzuki, H., Naganuma, A., Takagi, H., Sato, K., & Uraoka, T. (2019). Impact of the Prognostic Nutritional Index on the

Survival of Japanese Patients with Hepatocellular Carcinoma Treated with Sorafenib: A Multicenter Retrospective Study. *Intern Med*, *58*(13), 1835-1844. https://doi.org/10.2169/internalmedicine.1594-18

Hauner, H., Kocsis, A., Jaeckel, B., Martignoni, M., Hauner, D., & Holzapfel, C. (2020). Prevalence of malnutrition risk in patients of cancer outpatient clinics - A cross-sectional survey [Article]. *Deutsche Medizinische Wochenschrift*, *145*(1), E1-E9. https://doi.org/10.1055/a-1008-5702

Hayama, T., Hashiguchi, Y., Ozawa, T., Watanabe, M., Fukushima, Y., Shimada, R., Nozawa, K., Matsuda, K., Fujii, S., & Fukagawa, T. (2022). The preoperative geriatric nutritional risk index (GNRI) is an independent prognostic factor in elderly patients underwent curative resection for colorectal cancer [Article]. *Scientific reports*, *12*(1), 3682. https://doi.org/10.1038/s41598-022-07540-6

Hayama, T., Ozawa, T., Okada, Y., Tsukamoto, M., Fukushima, Y., Shimada, R., Nozawa, K., Matsuda, K., Fujii, S., & Hashiguchi, Y. (2020). The pretreatment Controlling Nutritional Status (CONUT) score is an independent prognostic factor in patients undergoing resection for colorectal cancer [Article]. *Scientific reports*, *10*(1), 13239. https://doi.org/10.1038/s41598-020-70252-2

Hayashi, H., Shimizu, A., Kubota, K., Notake, T., Masuo, H., Yoshizawa, T., Hosoda, K., Sakai, H., Yasukawa, K., & Soejima, Y. (2023). Combination of sarcopenia and prognostic nutritional index to predict long-term outcomes in patients undergoing initial hepatectomy for hepatocellular carcinoma [Article]. *Asian journal of surgery*, *46*(2), 816-823. https://doi.org/10.1016/j.asjsur.2022.07.122

He, Y., Liao, W. J., Hu, A. Q., Li, X. Y., Wang, J. G., & Qian, D. (2024). A nomogram based on clinical characteristics and nutritional indicators for relative and absolute weight loss during radiotherapy in initially inoperable patients with locally advanced esophageal squamous cell carcinoma. *Nutrition*, *117*, 112227. https://doi.org/10.1016/j.nut.2023.112227

He, Y., Liu, H., Ma, Y., Li, J., Zhang, J., Ren, Y., Dong, C., Bai, B., Zhang, Y., Lin, Y., Yue, P., & Meng, W. (2023). Preoperative prognostic nutritional index predicts short-term complications after radical resection of distal cholangiocarcinoma. *Frontiers in surgery*, *9*, 1091534-NA. https://doi.org/10.3389/fsurg.2022.1091534

Hendifar, A. E., Chang, J. I., Huang, B. Z., Tuli, R., & Wu, B. U. (2017). Cachexia, and not obesity, prior to pancreatic cancer diagnosis worsens survival and is negated by chemotherapy. *Journal of Gastrointestinal Oncology*, *9*(1), 17-23. https://jgo.amegroups.org/article/view/17616

Hirahara, N., Matsubara, T., Fujii, Y., Kaji, S., Hyakudomi, R., Yamamoto, T., Uchida, Y., Miyazaki, Y., Ishitobi, K., Kawabata, Y., & Tajima, Y. (2020). Geriatric nutritional risk index as a prognostic marker of pTNM-stage I and II esophageal squamous cell carcinoma after curative resection. *Oncotarget*, *11*(29), 2834-2846. https://doi.org/10.18632/oncotarget.27670

Hirahara, N., Matsubara, T., Fujii, Y., Kaji, S., Hyakudomi, R., Yamamoto, T., Uchida, Y., Miyazaki, Y., Ishitobi, K., Kawabata, Y., & Tajima, Y. (2020). Preoperative geriatric nutritional risk index is a useful prognostic indicator in elderly patients with gastric cancer. *Oncotarget*, *11*(24), 2345-2356. https://doi.org/10.18632/oncotarget.27635

Hirahara, N., Matsubara, T., Hayashi, H., Takai, K., Nakada, S., & Tajima, Y. (2018). Prognostic Importance of Controlling Nutritional Status in Patients Undergoing Curative Thoracoscopic Esophagectomy for Esophageal Cancer. *Am J Ther*, *25*(5), e524-e532. https://doi.org/10.1097/mjt.0000000000000414

Hirahara, N., Tajima, Y., Fujii, Y., Kaji, S., Kawabata, Y., Hyakudomi, R., Yamamoto, T., & Taniura, T. (2019). Controlling Nutritional Status (CONUT) as a prognostic immunonutritional biomarker for gastric cancer after curative gastrectomy: a propensity score-matched analysis. *Surg Endosc*, *33*(12), 4143-4152. https://doi.org/10.1007/s00464-019-06723-z

Hirahara, N., Tajima, Y., Fujii, Y., Kaji, S., Yamamoto, T., Hyakudomi, R., Taniura, T., Miyazaki, Y., Kishi, T., & Kawabata, Y. (2018). Preoperative Prognostic Nutritional Index Predicts Long-Term Surgical Outcomes in Patients with Esophageal Squamous Cell Carcinoma. *World J Surg*, *42*(7), 2199-2208. https://doi.org/10.1007/s00268-017-4437-1

Hiramatsu, K., Shindoh, J., Hanaoka, Y., Toda, S., Ueno, M., Matoba, S., & Kuroyanagi, H. (2021). Postoperative Nutritional Status is Predictive of the Survival Outcomes in Patients Undergoing Resection of Stage III Colorectal Cancer. *World J Surg*, *45*(10), 3198-3205. https://doi.org/10.1007/s00268-021-06202-4

Hiraoka, A., Kumada, T., Tada, T., Hirooka, M., Kariyama, K., Tani, J., Atsukawa, M., Takaguchi, K., Itobayashi, E., Fukunishi, S., Tsuji, K., Ishikawa, T., Tajiri, K., Ochi, H., Yasuda, S., Toyoda, H., Ogawa, C., Nishimura, T., Hatanaka, T., Kakizaki, S., Shimada, N., Kawata, K., Naganuma, A., Kosaka, H., Matono, T., Kuroda, H., Yata, Y., Ohama, H., Tada, F., Nouso, K., Morishita, A., Tsutsui, A., Nagano, T., Itokawa, N., Okubo, T., Arai, T., Imai, M., Koizumi, Y., Nakamura, S., Iijima, H., Kaibori, M., & Hiasa, Y. (2023). Geriatric nutritional risk index as an easy-to-use assessment tool for nutritional status in hepatocellular carcinoma treated with atezolizumab plus bevacizumab. *Hepatol Res*, *53*(10), 1031-1042. https://doi.org/10.1111/hepr.13934

Ho, C. T., Chia-Hui Tan, E., Lee, P. C., Chu, C. J., Huang, Y. H., Huo, T. I., Hou, M. C., Wu, J. C., & Su, C. W. (2024). Prognostic Nutritional Index as a Prognostic Factor for Very Early-Stage Hepatocellular Carcinoma. *Clin Transl Gastroenterol*, *15*(4), e00678. https://doi.org/10.14309/ctg.0000000000000678

Hoc, T. H., Hieu, N. D., Phu, P. V., Huong, T. T., & Son, T. Q. (2021). Nutritional status of patients undergoing upper gastrointestinal cancer surgery: A cross-sectional study at a single centre. *Tạp chí Nghiên cứu Y học*, *148*(12), 158-164. https://doi.org/10.52852/tcncyh.v148i12.565

Hu, S.-P., Chen, L., Lin, C.-Y., Lin, W.-H., Fang, F.-Q., & Tu, M.-Y. (2020). The prognostic value of preoperative geriatric nutritional risk index in patients with pancreatic ductal adenocarcinoma. *Cancer management and research*, 385-395.

Hu, Y., Cai, Y., Ma, W., Hu, H., Gu, H., Jin, Y., & Li, F. (2023). The prognostic nutritional index and tumor pathological characteristics predict the prognosis of elderly patients with early-stage hepatocellular carcinoma after surgery. *Biosci Trends*, *17*(5), 369-380. https://doi.org/10.5582/bst.2023.01212 1

Hu, Z., Li, Y., Mao, W., Chen, B., Yang, L., & Meng, X. (2020). Impact of Nutritional Indices on the Survival Outcomes of Patients with Colorectal Cancer. *Cancer management and research*, *12*, 2279-2289. https://doi.org/10.2147/cmar.s243172

Hua, X. H., Shi, K. F., Yu, Y. K., Li, H. M., Ma, F., Sun, H. B., Qian, R. L., & Li, Y. (2022). Nutritional assessment in esophageal fast-track surgery: comparisons of 4 objective malnutrition screening tools. *Ann Transl Med*, *10*(1), 20. https://doi.org/10.21037/atm-21-6383

Huang, D. D., Yu, D. Y., Song, H. N., Wang, W. B., Luo, X., Wu, G. F., Yu, Z., Liu, N. X., Dong, Q. T., Chen, X. L., & Yan, J. Y. (2021). The relationship between the GLIM-defined malnutrition, body composition and functional parameters, and clinical outcomes in elderly patients undergoing radical gastrectomy for gastric cancer [Article]. *European Journal of Surgical Oncology*, *47*(9), 2323-2331. https://doi.org/10.1016/j.ejso.2021.02.032

Huang, D. D., Yu, D. Y., Wang, W. B., Song, H. N., Luo, X., Wu, G. F., Chen, X. L., Yu, Z., & Yan, J. Y. (2022). Global leadership initiative in malnutrition (GLIM) criteria using hand-grip strength adequately predicts postoperative complications and long-term survival in patients underwent radical gastrectomy for gastric cancer. *Eur J Clin Nutr*, *76*(9), 1323-1331. https://doi.org/10.1038/s41430-022-01109-2

Huang, P. Y., Wang, C. C., Lin, C. C., Lu, S. N., Wang, J. H., Hung, C. H., Kee, K. M., Chen, C. H., Chen, K. D., Hu, T. H., & Tsai, M. C. (2019). Predictive Effects of Inflammatory Scores in Patients with BCLC 0-A Hepatocellular Carcinoma after Hepatectomy. *J Clin Med*, *8*(10). https://doi.org/10.3390/jcm8101676

Huang, S., Wang, S., Xie, Y., He, X., Yi, X., Zhang, J., Deng, Z., & Yin, L. (2021). Application of NRS2002 in Preoperative Nutritional Screening for Patients with Liver Cancer. *J Oncol*, *2021*, 8943353. https://doi.org/10.1155/2021/8943353

Huang, T. H., Hsieh, C. C., Kuo, L. M., Chang, C. C., Chen, C. H., Chi, C. C., & Liu, C. H. (2019). Malnutrition associated with an increased risk of postoperative complications following hepatectomy in patients with hepatocellular carcinoma. *HPB (Oxford)*, *21*(9), 1150-1155. https://doi.org/10.1016/j.hpb.2019.01.003

Huang, W., Wang, C., Wang, Y., Yu, Z., Wang, S., Yang, J., Lu, S., Zhou, C., Wu, E., & Chen, J. (2024). Predicting malnutrition in gastric cancer patients using computed tomography(CT) deep learning features and clinical data. *Clin Nutr*, *43*(3), 881-891. https://doi.org/10.1016/j.clnu.2024.02.005

Huang, Y., Huang, Y., Lu, M., Sun, W., Sun, X., Chen, X., Li, L., Chandoo, A., & Li, L. (2019). Controlling Nutritional Status (CONUT) Score Is A Predictor Of Post-Operative Outcomes In Elderly Gastric Cancer Patients Undergoing Curative Gastrectomy: A Prospective Study. *Cancer Manag Res*, *11*, 9793-9800. https://doi.org/10.2147/cmar.S233872

Huang, Y., Huang, Z., Hou, W., Wang, C., Wang, X., & Zuo, J. (2025). Cachexia index as a biomarker for cancer cachexia and quality of life in patients with gastric cancer. *BMC Cancer*, *25*(1), 1293. https://doi.org/10.1186/s12885-025-14752-2

Ide, S., Okugawa, Y., Omura, Y., Yamamoto, A., Ichikawa, T., Kitajima, T., Shimura, T., Imaoka, H., Fujikawa, H., Yasuda, H., Yokoe, T., Okita, Y., Ohi, M., & Toiyama, Y. (2021). Geriatric nutritional risk index predicts cancer prognosis in patients with local advanced rectal cancer undergoing chemoradiotherapy followed by curative surgery [Article]. *World Journal of Surgical Oncology*, *19*(1). https://doi.org/10.1186/s12957-021-02139-z

Igarashi, T., Harimoto, N., Fukushima, R., Hagiwara, K., Hoshino, K., Kawai, S., Ishii, N., Tsukagoshi, M., Araki, K., & Shirabe, K. (2025). Significance of the modified global leadership initiative on malnutrition (GLIM) criteria malcondition for patients with biliary tract cancer. *Surg Today*, *55*(6), 830-838. https://doi.org/10.1007/s00595-024-02970-3

Iguchi, T., Sugimachi, K., Mano, Y., Motomura, T., Sugiyama, M., Ota, M., Ikebe, M., Esaki, T., Yoshizumi, T., Morita, M., Mori, M., & Toh, Y. (2020). Prognostic Impact of Geriatric Nutritional Risk Index in Patients With Synchronous Colorectal Liver Metastasis. *Anticancer research*, *40*(7), 4165-4171. https://doi.org/10.21873/anticanres.14416

Ikeya, T., Shibutani, M., Maeda, K., Sugano, K., Nagahara, H., Ohtani, H., & Hirakawa, K. (2015). Maintenance of the nutritional prognostic index predicts survival in patients with unresectable metastatic colorectal cancer. *J Cancer Res Clin Oncol*, *141*(2), 307-313. https://doi.org/10.1007/s00432-014-1799-8

Ikuta, S., Nakajima, T., Fujikawa, M., Aihara, T., & Yamanaka, N. (2023). Prognostic value of geriatric nutritional risk index for patients with biliary tract cancer undergoing surgical resection – a single-institution retrospective cohort study [Article]. *Wspolczesna Onkologia*, *27*(2), 65-70. https://doi.org/10.5114/wo.2023.127436

Imaoka, Y., Ohira, M., Kobayashi, T., Honmyo, N., Hamaoka, M., Onoe, T., Abe, T., Oishi, K., Inoue, M., & Ohdan, H. (2023). Impact of Geriatric Nutritional Risk Index After Initial Hepatectomy for Hepatocellular Carcinoma: a Retrospective Cohort Study with the Hiroshima Surgical Study Group of Clinical Oncology (HiSCO). *J Gastrointest Surg*, *27*(6), 1152-1158. https://doi.org/10.1007/s11605-

Iseki, Y., Shibutani, M., Maeda, K., Nagahara, H., Ohtani, H., Sugano, K., Ikeya, T., Muguruma, K., Tanaka, H., Toyokawa, T., Sakurai, K., & Hirakawa, K. (2015). Impact of the Preoperative Controlling Nutritional Status (CONUT) Score on the Survival after Curative Surgery for Colorectal Cancer. *PLoS One*, *10*(7), e0132488. https://doi.org/10.1371/journal.pone.0132488

Ishizuka, M., Oyama, Y., Abe, A., Tago, K., Tanaka, G., & Kubota, K. (2014). Prognostic nutritional index is associated with survival after total gastrectomy for patients with gastric cancer. *Anticancer Res*, *34*(8), 4223-4229.

Itoh, S., Tsujita, E., Fukuzawa, K., Sugimachi, K., Iguchi, T., Ninomiya, M., Maeda, T., Kajiyama, K., Adachi, E., Uchiyama, H., Utsunomiya, T., Ikeda, Y., Maekawa, S., Toshima, T., Harada, N., Yoshizumi, T., & Mori, M. (2021). Prognostic significance of preoperative PNI and CA19-9 for pancreatic ductal adenocarcinoma: A multi-institutional retrospective study. *Pancreatology : official journal of the International Association of Pancreatology (IAP) ... [et al.]*, *21*(7), 1356-1363. https://doi.org/10.1016/j.pan.2021.08.003

Jeon, C. H., Park, K. B., Jung, Y. J., Seo, H. S., Park, C. H., Song, K. Y., & Lee, H. H. (2020). Modified controlling

nutritional status score: A refined prognostic indicator depending on the stage of gastric cancer [Review]. *Surgical Oncology*, *34*, 261-269. https://doi.org/10.1016/j.suronc.2020.05.008

Jeong, H., Kim, K. H., Jo, S., & Song, S. (2021). Impact of prognostic nutritional index on the recurrence of hepatocellular carcinoma after a curative resection. *Ann Hepatobiliary Pancreat Surg*, *25*(4), 456-461. https://doi.org/10.14701/ahbps.2021.25.4.456

Ji, F., Liang, Y., Fu, S., Chen, D., Cai, X., Li, S., Peng, B., Liang, L., & Hua, Y. (2017). Prognostic value of combined preoperative prognostic nutritional index and body mass index in HCC after hepatectomy [Article]. *HPB*, *19*(8), 695-705. https://doi.org/10.1016/j.hpb.2017.04.008

Jia, P., Wu, X., Shen, F., Sun, K., Wang, X., Xu, G., Xu, H., Cong, M., Song, C., & Shi, H. (2024). The combination of handgrip strength and CONUT predicts overall survival in patients with gastrointestinal cancer: A multicenter cohort study. *Clin Nutr*, *43*(9), 2057-2068. https://doi.org/10.1016/j.clnu.2024.07.026

Jiang, N., Deng, J. Y., Ding, X. W., Ke, B., Liu, N., Zhang, R. P., & Liang, H. (2014). Prognostic nutritional index predicts postoperative complications and long-term outcomes of gastric cancer [Article]. *World Journal of Gastroenterology*, *20*(30), 10537-10544. https://doi.org/10.3748/wjg.v20.i30.10537

Jiang, Y., Guo, C., Zhang, D., Zhang, J., Wang, X., & Geng, C. (2014). The altered tight junctions: An important gateway of bacterial translocation in cachexia patients with advanced gastric cancer [Article]. *Journal of Interferon and Cytokine Research*, *34*(7), 518-525. https://doi.org/10.1089/jir.2013.0020

Jian-hui, C., Iskandar, E. A., Cai, S. I., Chen, C. Q., Wu, H., Xu, J. B., & He, Y. L. (2016). Significance of Onodera's prognostic nutritional index in patients with colorectal cancer: a large cohort study in a single Chinese institution [Article]. *Tumor Biology*, *37*(3), 3277-3283. https://doi.org/10.1007/s13277-015-4008-8

Jin, H., Zhu, K., & Wang, W. (2021). The Predictive Values of Pretreatment Controlling Nutritional Status (CONUT) Score in Estimating Short- and Long-term Outcomes for Patients with Gastric Cancer Treated with Neoadjuvant Chemotherapy and Curative Gastrectomy. *J Gastric Cancer*, *21*(2), 155-168. https://doi.org/10.5230/jgc.2021.21.e14

Jin, J., Xiong, G., Wang, X., Peng, F., Zhu, F., Wang, M., & Qin, R. (2021). The Impact of Preoperative and Postoperative Malnutrition on Outcomes for Ampullary Carcinoma After Pancreaticoduodenectomy. *Front Oncol*, *11*, 748341. https://doi.org/10.3389/fonc.2021.748341

Jing, Y., Ren, M., Li, X., Sun, X., Xiao, Y., Xue, J., & Liu, Z. (2024). The Effect of Systemic Immune-Inflammatory Index (SII) and Prognostic Nutritional Index (PNI) in Early Gastric Cancer. *J Inflamm Res*, *17*, 10273-10287. https://doi.org/10.2147/jir.S499094

Johns, N., Hatakeyama, S., Stephens, N. A., Degen, M., Degen, S., Frieauff, W., Lambert, C., Ross, J. A., Roubenoff, R., Glass, D. J., Jacobi, C., & Fearon, K. C. H. (2014). Clinical classification of cancer cachexia: Phenotypic correlates in human skeletal muscle [Article]. *PLoS ONE*, *9*(1). https://doi.org/10.1371/journal.pone.0083618

Kamada, T., Haruki, K., Nakashima, K., Takahashi, J., Nakaseko, Y., Suzuki, N., Ohdaira, H., Eto, K., Ikegami, T., & Suzuki, Y. (2023). Prognostic significance of the cachexia index in patients with stage I–III colorectal cancer who underwent laparoscopic surgery [Article in Press]. *Surgery Today*. https://doi.org/10.1007/s00595-023-02646-4

Kanda, M., Mizuno, A., Tanaka, C., Kobayashi, D., Fujiwara, M., Iwata, N., Hayashi, M., Yamada, S., Nakayama, G., Fujii, T., Sugimoto, H., Koike, M., Takami, H., Niwa, Y., Murotani, K., & Kodera, Y. (2016). Nutritional predictors for postoperative short-term and long-term outcomes of patients with gastric cancer. *Medicine (Baltimore)*, *95*(24), e3781. https://doi.org/10.1097/md.0000000000003781

Kaner, G., Yılmaz, A. Z., Inanc, N., Yıldırım, M. N. S., Kurklu, N. S., & Koyu, E. B. (2018). Assessment of nutritional status and quality of life among patients with cancer who underwent major upper gastrointestinal system cancer surgery [Article]. *Progress in Nutrition*, *20*(4), 592-601. https://doi.org/10.23751/pn.v20i4.6117

Kang, J., Yang, G., Wang, D., Lin, Y., Wang, Q., & Luo, H. (2022). The Clinical Application Value of the

Prognostic Nutritional Index for the Overall Survival Prognosis of Patients with Esophageal Cancer: a Robust Real-World Observational Study in China [Journal article]. *Computational and mathematical methods in medicine*, *2022*, 3889588. https://doi.org/10.1155/2022/3889588

Kanno, H., Goto, Y., Sasaki, S., Fukutomi, S., Hisaka, T., Fujita, F., Akagi, Y., & Okuda, K. (2021). Geriatric nutritional risk index predicts prognosis in hepatocellular carcinoma after hepatectomy: a propensity score matching analysis. *Sci Rep*, *11*(1), 9038. https://doi.org/10.1038/s41598-021-88254-z

Kapagan, T., Bulut, N., Arslansoy, B., Ozcalimli, Z., Turkmencalikoglu, M., Yontan, E., Kılıc, C. E., Kardes, B. C. O., & Erdem, G. U. (2025). Nutritional status and prognosis in metastatic colorectal cancer: a cohort study. *Rev Assoc Med Bras (1992)*, *71*(6), e20250238. https://doi.org/10.1590/1806-9282.20250238 10

Kataoka, M., Hirano, Y., Ishii, T., Ishikawa, S., Kataoka, A., Fujii, T., & Shimamura, S. (2021). Prognostic Utility of Geriatric Nutritional Risk Index After Curative Resection of Colorectal Cancer: A Propensity Score-matched Study. *Cancer diagnosis & prognosis*, *1*(5), 479-484. https://doi.org/10.21873/cdp.10064

Kato, Y., Yamada, S., Suenaga, M., Takami, H., Niwa, Y., Hayashi, M., Iwata, N., Kanda, M., Tanaka, C., Nakayama, G., Koike, M., Fujiwara, M., & Kodera, Y. (2018). Impact of the Controlling Nutritional Status Score on the Prognosis After Curative Resection of Pancreatic Ductal Adenocarcinoma. *Pancreas*, *47*(7), 823-829. https://doi.org/10.1097/mpa.0000000000001105

Kawahara, S., Aoyama, T., Murakawa, M., Kanemoto, R., Takahashi, D., Kamioka, Y., Hashimoto, I., Maezawa, Y., Kobayashi, S., Ueno, M., Yamamoto, N., Oshima, T., Yukawa, N., Rino, Y., Saito, A., & Morinaga, S. (2024). Prognostic nutritional index is an independent risk factor for continuing S-1 adjuvant chemotherapy in patients with pancreatic cancer who received neoadjuvant chemotherapy and surgical resection. *BMC cancer*, *24*(1), 1469-NA. https://doi.org/10.1186/s12885-024-13244-z

Kaya, A. Ş., & Pekcan, G. (2020). Evaluation of preoperative nutritional status of patients with gastrointestinal cancer using different nutritional screening tests [Article]. *Progress in Nutrition*, *22*(4). https://doi.org/10.23751/pn.v22i4.9339

Kazlow, E., Rinawi, E., Gerszman, E., Mattar, S., Essami, N., Nasir, M., Abu Shtaya, A., Assaf, W., Haddad, R., & Mahamid, A. (2025). Higher Neutrophil-to-Lymphocyte Ratio and Systemic Immune–Inflammation Index Is Associated with Better Prognosis Following Pancreaticoduodenectomy for Pancreatic Adenocarcinoma [Article]. *Journal of Clinical Medicine*, *14*(11). https://doi.org/10.3390/jcm14113762

Ke, M., Xu, T., Li, N., Ren, Y., Shi, A., Lv, Y., & He, H. (2016). Prognostic nutritional index predicts short-term outcomes after liver resection for hepatocellular carcinoma within the Milan criteria. *Oncotarget*, *7*(49), 81611-81620. https://doi.org/10.18632/oncotarget.13151

Keskinkilic, M., Semiz, H. S., Ataca, E., & Yavuzsen, T. (2024). The prognostic value of immune-nutritional status in metastatic colorectal cancer: Prognostic Nutritional Index (PNI). *Support Care Cancer*, *32*(6), 374. https://doi.org/10.1007/s00520-024-08572-6 10.1007/s00520-024-08572-6.

Kim, E., Jang, J. Y., Han, Y., Kim, H., Lee, H. E., Kwon, W., Kim, J., & Kim, S. W. (2017). Influence of preoperative nutritional status on clinical outcomes after pancreatoduodenectomy [Conference Abstract]. *Journal of Hepato-Biliary-Pancreatic Sciences*, *24*, A123.

Kim, E., Kang, J. S., Han, Y., Kim, H., Kwon, W., Kim, J. R., Kim, S. W., & Jang, J. Y. (2018). Influence of preoperative nutritional status on clinical outcomes after pancreatoduodenectomy [Article]. *HPB*, *20*(11), 1051-1061. https://doi.org/10.1016/j.hpb.2018.05.004

Kim, E., Lee, D. H., & Jang, J. Y. (2019). Effects of Preoperative Malnutrition on Postoperative Surgical Outcomes and Quality of Life of Elderly Patients with Periampullary Neoplasms: A Single-Center Prospective Cohort Study. *Gut Liver*, *13*(6), 690-697. https://doi.org/10.5009/gnl18469

Kim, Y., Kim, S. R., Kim, K., & Yu, S. J. (2024). Demographic, clinical and psychological predictors of malnutrition among people with liver cancer. *Eur J Oncol Nurs*, *68*, 102497.

https://doi.org/10.1016/j.ejon.2023.102497

Kim, Y. J., Sung, M. K., Kim, C. S., Lee, J., Kim, J. H., Sim, J. H., & Jeong, S. M. (2025). Impact of prognostic nutritional index on survival in periampullary/pancreatic cancer patients undergoing pylorus-preserving pancreaticoduodenectomy: A propensity score-matched analysis. *Hepatobiliary Pancreat Dis Int*, *24*(5), 550-557. https://doi.org/10.1016/j.hbpd.2025.06.007

Kinoshita, A., Hagiwara, N., Osawa, A., Akasu, T., Matsumoto, Y., Ueda, K., Saeki, C., Oikawa, T., Koike, K., & Saruta, M. (2022). The Geriatric Nutritional Risk Index Predicts Tolerability of Lenvatinib in Patients With Hepatocellular Carcinoma [Article]. *In Vivo*, *36*(2), 865-873. https://doi.org/10.21873/INVIVO.12775

Konaka, R., Yanagimoto, H., Tsugawa, D., Akita, M., Mizumoto, T., Yoshida, T., Sou, S., Ishida, J., Nanno, Y., Urade, T., Fukushima, K., Gon, H., Komatsu, S., Asari, S., Kido, M., Toyama, H., & Fukumoto, T. (2025). GLIM Criteria as Prognostic Factor in Patients Undergoing Adjuvant S-1 Chemotherapy for Biliary Tract Cancer. *Cancer Diagn Progn*, *5*(5), 557-565. https://doi.org/10.21873/cdp.10470

Kong, X., Liu, P., Wang, G., Sun, S., & Li, L. (2025). Methods for diagnosing malnutrition in patients with esophageal cancer, and the association with nutritional and inflammatory indices: A cross-sectional study. *Oncol Lett*, *29*(5), 223. https://doi.org/10.3892/ol.2025.14969

Korkmaz, M., Eryılmaz, M. K., Er, M. M., Koçak, M. Z., Demirkıran, A., Karaağaç, M., Araz, M., Artaç, M., & Koçak, Z. M. (2023). Is the Prognostic Nutritional Index a Prognostic Marker for the Survival of Patients with Lymph-Node Positive Stage II-III Gastric Cancer Who Receive Adjuvant Chemotherapy? *J Gastrointest Cancer*, *54*(3), 962-969. https://doi.org/10.1007/s12029-023-00972-x

Kosuga, T., Konishi, T., Kubota, T., Shoda, K., Konishi, H., Shiozaki, A., Okamoto, K., Fujiwara, H., Kudou, M., Arita, T., Morimura, R., Murayama, Y., Kuriu, Y., Ikoma, H., Nakanishi, M., & Otsuji, E. (2019). Value of Prognostic Nutritional Index as a Predictor of Lymph Node Metastasis in Gastric Cancer. *Anticancer research*, *39*(12), 6843-6849. https://doi.org/10.21873/anticanres.13901

Kouzu, K., Tsujimoto, H., Sugasawa, H., Ishibashi, Y., Hase, K., Kishi, Y., & Ueno, H. (2021). Modified geriatric nutrition risk index as a prognostic predictor of esophageal cancer [Article]. *Esophagus*, *18*(2), 278-287. https://doi.org/10.1007/s10388-020-00795-w

Kubo, N., Ohira, M., Tamura, T., Sakurai, K., Toyokawa, T., Tanaka, H., Yashiro, M., Yamashita, Y., & Hirakawa, K. (2017). Prognostic significance of baseline nutritional index for patients with esophageal squamous cell carcinoma after radical esophagectomy [Article]. *Esophagus*, *14*(1), 84-90. https://doi.org/10.1007/s10388-016-0548-2

Kubo, N., Sakurai, K., Tamura, T., Toyokawa, T., Tanaka, H., Muguruma, K., Yashiro, M., & Ohira, M. (2019). The impact of geriatric nutritional risk index on surgical outcomes after esophagectomy in patients with esophageal cancer. *Esophagus*, *16*(2), 147-154. https://doi.org/10.1007/s10388-018-0644-6

Kuroda, D., Sawayama, H., Kurashige, J., Iwatsuki, M., Eto, T., Tokunaga, R., Kitano, Y., Yamamura, K., Ouchi, M., Nakamura, K., Baba, Y., Sakamoto, Y., Yamashita, Y., Yoshida, N., Chikamoto, A., & Baba, H. (2018). Controlling Nutritional Status (CONUT) score is a prognostic marker for gastric cancer patients after curative resection. *Gastric Cancer*, *21*(2), 204-212. https://doi.org/10.1007/s10120-017-0744-3

Kurtul, N., & Eroğlu, C. (2017). Low prognostic nutritional index before radiotherapy is a poor prognostic factor for rectum cancer [Article]. *Journal of Clinical and Analytical Medicine*, *8*(3), 185-189. https://doi.org/10.4328/JCAM.4796

Latenstein, A. E. J., Dijksterhuis, W. P. M., Mackay, T. M., Beijer, S., van Eijck, C. H. J., de Hingh, I., Molenaar, I. Q., van Oijen, M. G. H., van Santvoort, H. C., de van der Schueren, M. A. E., de Vos-Geelen, J., de Vries, J. H. M., Wilmink, J. W., Besselink, M. G., & van Laarhoven, H. W. M. (2020). Cachexia, dietetic consultation, and survival in patients with pancreatic and periampullary cancer: A multicenter cohort study. *Cancer Med*, *9*(24), 9385-9395. https://doi.org/10.1002/cam4.3556

Lee, J. Y., Kim, H. I., Kim, Y. N., Hong, J. H., Alshomimi, S., An, J. Y., Cheong, J. H., Hyung, W. J., Noh, S. H., & Kim, C. B. (2016). Clinical Significance of the Prognostic Nutritional Index for Predicting Short- and Long-Term Surgical Outcomes After Gastrectomy: A Retrospective Analysis of 7781 Gastric Cancer Patients. *Medicine (Baltimore)*, *95*(18), e3539. https://doi.org/10.1097/md.0000000000003539

Lee, S., Kang, D. H., Ahn, T. S., Jo, D. H., Kim, E., & Baek, M. J. (2023). Clinical influence of neoadjuvant chemoradiotherapy on immunonutritional status in locally advanced rectal cancer. *Korean J Clin Oncol*, *19*(1), 3-10. https://doi.org/10.14216/kjco.23002

Lee, S. H., Chung, M. J., Kim, B., Lee, H. S., Lee, H. J., Heo, J. Y., Kim, Y. J., Park, J. Y., Bang, S., Park, S. W., Song, S. Y., & Chung, J. B. (2017). The Significance of the Prognostic Nutritional Index for All Stages of Pancreatic Cancer. *Nutrition and Cancer*, *69*(3), 512-519. https://doi.org/10.1080/01635581.2016.1250921

Lee, S. Y., Jung, M. R., Kim, C. H., Kim, Y. J., & Kim, H. R. (2018). Nutritional risk screening score is an independent predictive factor of anastomotic leakage after rectal cancer surgery. *Eur J Clin Nutr*, *72*(4), 489-495. https://doi.org/10.1038/s41430-018-0112-3

Lee, S. Y., & Kim, H. R. (2019). NUTRITIONAL RISK SCREENING SCORE IS ASSOCIATED WITH OMISSION OF ADJUVANT CHEMOTHERAPY FOR STAGE III COLON CANCER [Conference Abstract]. *Gastroenterology*, *156*(6), S-1448. https://doi.org/10.1016/S0016-5085(19)40678-1

Lee, S. Y., Yeom, S. S., Kim, C. H., & Kim, H. R. (2020). Nutritional risk screening score is associated with omission of adjuvant chemotherapy for stage III colon cancer. *Am J Surg*, *220*(4), 993-998. https://doi.org/10.1016/j.amjsurg.2020.02.015

Li, G., He, L., & Sun, H. (2023). Nutritional risk index predicts the prognosis of gastric cancer patients with pyloric stenosis who received preoperative parenteral nutrition. *Oncol Lett*, *26*(3), 401. https://doi.org/10.3892/ol.2023.13988

Li, H., Li, B., Wang, X., Zhang, H., Wang, C., Fan, B., & Wang, L. (2024). Effect of longitudinal changes of cachexia on the efficacy and toxicity of immune checkpoint inhibitors in esophageal squamous cell cancer (ESCC) patients. *Nutrition*, *124*, 112462. https://doi.org/10.1016/j.nut.2024.112462

Li, J., Jiang, M., Hua, X., Xu, H., Wu, M., Wu, J., Liu, S., Shi, H., & Meng, Q. (2024). Reduced muscle mass is an important part of Global Leadership Initiative on Malnutrition criteria in nutritional diagnosis of hepatocellular carcinoma. *BMC Gastroenterol*, *24*(1), 358. https://doi.org/10.1186/s12876-024-03438-x

Li, J., Shi, H. Y., & Zhou, M. (2023). Correlation between preoperative systemic immune inflammation index, nutritional risk index, and prognosis of radical resection of liver cancer. *World J Gastrointest Surg*, *15*(11), 2445-2455. https://doi.org/10.4240/wjgs.v15.i11.2445

Li, J., Zhu, N., Wang, C., You, L., Guo, W., Yuan, Z., Qi, S., Zhao, H., Yu, J., & Huang, Y. (2023). Preoperative albumin-to-globulin ratio and prognostic nutritional index predict the prognosis of colorectal cancer: a retrospective study. *Sci Rep*, *13*(1), 17272. https://doi.org/10.1038/s41598-023-43391-5

Li, K. J., Zhang, Z. Y., Sulayman, S., Shu, Y., Wang, K., Ababaike, S., Zeng, X. Y., & Zhao, Z. L. (2024). Prognostic value of combined systemic inflammation response index and prognostic nutritional index in colorectal cancer patients. *World J Gastrointest Surg*, *16*(12), 3794-3805. https://doi.org/10.4240/wjgs.v16.i12.3794

Li, L., Liu, C., Yang, J., Wu, H., Wen, T., Wang, W., Li, B., & Yan, L. (2018). Early postoperative controlling nutritional status (CONUT) score is associated with complication III-V after hepatectomy in hepatocellular carcinoma: A retrospective cohort study of 1,334 patients. *Sci Rep*, *8*(1), 13406. https://doi.org/10.1038/s41598-018-31714-w

Li, S., Guo, J. H., Lu, J., Wang, C., & Wang, H. (2021). Prognostic value of preoperative prognostic nutritional index and body mass index combination in patients with unresectable hepatocellular carcinoma after transarterial chemoembolization [Article]. *Cancer Management and Research*, *13*, 1637-1650. https://doi.org/10.2147/CMAR.S290983

Li, X. X., Liu, B., Zhao, Y. F., Jiang, Y., Cui, Y., & Peng, X. G. (2024). Functional Liver Imaging Score Derived from Gadoxetic Acid-enhanced MRI Predicts Cachexia and Prognosis in Hepatocellular Carcinoma Patients. *Curr Med Sci*, *44*(5), 1018-1025. https://doi.org/10.1007/s11596-024-2930-y

Li, Y., Nie, C., Li, N., Liang, J., Su, N., & Yang, C. (2024). The association between controlling nutritional status and postoperative pulmonary complications in patients with colorectal cancer. *Front Nutr*, *11*, 1425956. https://doi.org/10.3389/fnut.2024.1425956

Liang, X., Liangliang, X., Peng, W., Tao, Y., Jinfu, Z., Ming, Z., & Mingqing, X. (2021). Combined prognostic nutritional index and albumin-bilirubin grade to predict the postoperative prognosis of HBV-associated hepatocellular carcinoma patients. *Scientific reports*, *11*(1), 14624-NA. https://doi.org/10.1038/s41598-021-94035-5

Liao, C. K., Chern, Y. J., Hsu, Y. J., Lin, Y. C., Yu, Y. L., Chiang, J. M., Yeh, C. Y., & You, J. F. (2021). The Clinical Utility of the Geriatric Nutritional Risk Index in Predicting Postoperative Complications and Long-Term Survival in Elderly Patients with Colorectal Cancer after Curative Surgery. *Cancers (Basel)*, *13*(22). https://doi.org/10.3390/cancers13225852

Liao, W.-C., Chen, P.-R., Huang, C.-C., Chang, Y. T., Huang, B.-S., Chang, C. C., Wu, M.-S., & Chow, L.-P. (2020). Relationship between pancreatic cancer-associated diabetes and cachexia. *Journal of cachexia, sarcopenia and muscle*, *11*(4), 899-908. https://doi.org/10.1002/jcsm.12553

Lin, S., Zheng, C., Chen, Z., Chen, Y., & Lin, W. (2025). Prognostic value of the combined fibrinogen and prognostic nutritional index score in resectable gastric cancer. *Front Oncol*, *15*, 1596774. https://doi.org/10.3389/fonc.2025.1596774

Lin, Z. X., Ruan, D. Y., Jia, C. C., Wang, T. T., Cheng, J. T., Huang, H. Q., & Wu, X. Y. (2020). Controlling nutritional status (CONUT) score-based nomogram to predict overall survival of patients with HBV-associated hepatocellular carcinoma after curative hepatectomy. *Clin Transl Oncol*, *22*(3), 370-380. https://doi.org/10.1007/s12094-019-02137-4

Liu, B., & Zhang, L. (2024). Geriatric nutritional risk index predicts the prognosis of gastric cancer patients treated with immune checkpoint inhibitors. *Medicine (Baltimore)*, *103*(17), e37863. https://doi.org/10.1097/md.0000000000037863

Liu, J., Fang, K., Pei, S., Gao, Y., Liu, L., Tang, M., Pan, X., Sun, M., & Zhang, C. (2025). The role of prognostic nutrition index in the prognosis of patients with advanced hepatocellular carcinoma who received first-line therapy. *BMC Cancer*, *25*(1), 1258. https://doi.org/10.1186/s12885-025-14672-1

Liu, J., Hu, G., Zhai, C., Wang, J., Xu, W., Xie, J., Zhu, W., Jiang, P., & Liu, D. (2023). Predictive value of nutritional indicators with regard to the survival outcomes in patients with metastatic esophageal squamous cell carcinoma treated with camrelizumab. *Oncol Lett*, *25*(5), 198. https://doi.org/10.3892/ol.2023.13784

Liu, J., Sun, R., Cai, K., Xu, Y., & Yuan, W. (2024). A nomogram combining neutrophil to lymphocyte ratio (NLR) and prognostic nutritional index (PNI) to predict distant metastasis in gastric cancer. *Sci Rep*, *14*(1), 15391. https://doi.org/10.1038/s41598-024-65307-7

Liu, J. Y., Dong, H. M., Wang, W. L., Wang, G., Pan, H., Chen, W. W., Wang, Q., & Wang, Z. J. (2021). The Effect of the Prognostic Nutritional Index on the Toxic Side Effects of Radiochemotherapy and Prognosis After Radical Surgery for Gastric Cancer. *Cancer Manag Res*, *13*, 3385-3392. https://doi.org/10.2147/cmar.S301140

Liu, L. X., Wang, H., Gao, B., Xu, T. T., Yuan, Q. G., Zhou, S. Z., Ding, C., Miao, J., & Guan, W. X. (2024). Preoperative controlling nutritional status as an optimal prognostic nutritional index to predict the outcome for colorectal cancer. *World J Gastrointest Oncol*, *16*(2), 343-353. https://doi.org/10.4251/wjgo.v16.i2.343

Liu, X., Zhang, D., Lin, E., Chen, Y., Li, W., Chen, Y., Sun, X., & Zhou, Z. (2018). Preoperative controlling nutritional status (CONUT) score as a predictor of long-term outcome after curative resection followed by adjuvant chemotherapy in stage II-III gastric Cancer. *BMC Cancer*, *18*(1), 699. https://doi.org/10.1186/s12885-018-4616-y

Liu, Y., Kang, J., Qi, Z., Yang, Y., Bai, M., & Yi, H. (2023). Comparison of GLIM and PG-SGA for predicting clinical outcomes of patients with esophageal squamous carcinoma resection. *Nutr Hosp*, *40*(3), 574-582. https://doi.org/10.20960/nh.04401 10.20960/nh.04401. (Comparación de GLIM y PG-SGA para predecir los desenlaces clínicos de pacientes con resección de carcinoma escamoso de esófago.)

LiYa, L., XinSheng, Z., Xiang, H., Zhao, L., Lu, L., XiuMing, L., Ye, L., Jing, C., KeMing, Z., HongChi, W., Jing, X., Yang, C., Xiu, C., HongBo, L., ShuQin, Y., Fang, L., & YingHua, L. (2024). A cross-sectional survey study on the correlation analysis of nutritional status and intestinal flora in patients with esophageal cancer. *Front Nutr*, *11*, 1424039. https://doi.org/10.3389/fnut.2024.1424039

Loh, K. W., Vriens, M. R., Gerritsen, A., Borel Rinkes, I. H., van Hillegersberg, R., Schippers, C., Steenhagen, E., Ong, T. A., Moy, F. M., & Molenaar, I. Q. (2012). Unintentional weight loss is the most important indicator of malnutrition among surgical cancer patients. *Neth J Med*, *70*(8), 365-369.

Lohsiriwat, V. (2014). The influence of preoperative nutritional status on the outcomes of an enhanced recovery after surgery (ERAS) programme for colorectal cancer surgery [Article]. *Techniques in Coloproctology*, *18*(11), 1075-1080. https://doi.org/10.1007/s10151-014-1210-4

Lu, S., Li, X., Li, X., Zhang, Q., Wang, Y., Peng, R., Fu, W., & Wang, H. (2023). The preoperative geriatric nutritional risk index predicts long-term prognosis in elderly locally advanced rectal cancer patients: a two-center retrospective cohort study [Article]. *Aging Clinical and Experimental Research*, *35*(2), 311-321. https://doi.org/10.1007/s40520-022-02297-4

Lu, Y., Xin, D., & Wang, F. (2019). Predictive Significance Of Preoperative Systemic Immune-Inflammation Index Determination In Postoperative Liver Metastasis Of Colorectal Cancer. *Onco Targets Ther*, *12*, 7791-7799. https://doi.org/10.2147/ott.S223419

Luo, X., Cai, B., & Jin, W. (2024). A modified GLIM criteria-based nomogram for the survival prediction of gastric cancer patients undergoing surgical resection. *BMC Gastroenterol*, *24*(1), 307. https://doi.org/10.1186/s12876-024-03395-5

Luo, Z., Zhou, L., Balde, A. I., Li, Z., He, L., ZhenWei, C., Zou, Z., Huang, S., Han, S., Wei Zhou, M., Zhang, G. Q., & Cai, Z. (2019). Prognostic impact of preoperative prognostic nutritional index in resected advanced gastric cancer: A multicenter propensity score analysis. *Eur J Surg Oncol*, *45*(3), 425-431. https://doi.org/10.1016/j.ejso.2018.09.004

Ma, H., Liu, Y., Ye, H., Gao, F., Li, Z., & Qin, S. (2024). The prognostic value of preoperative laboratory data indicators in patients with esophageal carcinoma: An observational study. *Medicine (Baltimore)*, *103*(24), e38477. https://doi.org/10.1097/md.0000000000038477

Ma, H., Zhou, J., Yang, S., Gao, X., Wei, H., Wu, D., Zuo, Z., Wang, Y., Li, S., & He, Y. (2025). Precise nutritional diagnosis in digestive system malignancies based on blood nutrients, inflammatory makers, intestinal function indicators and body composition. *Clin Nutr*, *54*, 251-264. https://doi.org/10.1016/j.clnu.2025.09.024

Ma, X., Jiang, X., Guo, H., Wang, J., Wang, T., Yao, J., Liang, S., Lu, X., Wang, C., & Wang, C. (2025). Using a nomogram based on the controlling nutritional status score to predict prognosis after surgery in patients with resectable gastric cancer. *BMC Gastroenterol*, *25*(1), 180. https://doi.org/10.1186/s12876-025-03766-6

Ma, X., Lu, X., Jiang, X., Wang, J., Wang, T., & Zhang, L. (2024). A nomogram combining prognostic nutritional index and platelet lymphocyte ratio predicts postoperative pulmonary infection following D2 radical gastrectomy for gastric cancer. *Nutr Hosp*, *41*(3), 602-611. https://doi.org/10.20960/nh.05079 10.20960/nh.05079. (Un nomograma que combina el índice nutricional pronóstico y el cociente plaquetario linfocitario predice la infección pulmonar postoperatoria tras la gastrectomía radical D2 por cáncer gástrico.)

Maeda, K., Shibutani, M., Otani, H., Nagahara, H., Sugano, K., Ikeya, T., Kubo, N., Amano, R., Kimura, K., Muguruma, K., Tanaka, H., & Hirakawa, K. (2014). Low nutritional prognostic index correlates with poor survival in patients with stage IV colorectal cancer following palliative resection of the primary tumor. *World J Surg*, *38*(5),

1217-1222. https://doi.org/10.1007/s00268-013-2386-x

Mao, Y. S., Hao, S. J., Zou, C. F., Xie, Z. B., & Fu, D. L. (2020). Controlling Nutritional Status score is superior to Prognostic Nutritional Index score in predicting survival and complications in pancreatic ductal adenocarcinoma: A Chinese propensity score matching study [Article]. *British Journal of Nutrition*, *124*(11), 1190-1197. https://doi.org/10.1017/S0007114520002299

Martínez-Escribano, C., Arteaga Moreno, F., Pérez-López, M., Cunha-Pérez, C., Belenguer-Varea, Á., Cuesta Peredo, D., Blanco González, F. J., & Tarazona-Santabalbina, F. J. (2022). Malnutrition and Increased Risk of Adverse Outcomes in Elderly Patients Undergoing Elective Colorectal Cancer Surgery: A Case-Control Study Nested in a Cohort. *Nutrients*, *14*(1). https://doi.org/10.3390/nu14010207

Matsuda, T., Umeda, Y., Matsuda, T., Endo, Y., Sato, D., Kojima, T., Sui, K., Inagaki, M., Ota, T., Hioki, M., Oishi, M., Kimura, M., Murata, T., Ishido, N., Yagi, T., & Fujiwara, T. (2021). Preoperative prognostic nutritional index predicts postoperative infectious complications and oncological outcomes after hepatectomy in intrahepatic cholangiocarcinoma. *BMC cancer*, *21*(1), 708-708. https://doi.org/10.1186/s12885-021-08424-0

Matsui, R., Inaki, N., & Tsuji, T. (2022). Effect of malnutrition as defined by the Global Leadership Initiative on Malnutrition criteria on compliance of adjuvant chemotherapy and relapse-free survival for advanced gastric cancer. *Nutrition*, *109*, 111958. https://doi.org/10.1016/j.nut.2022.111958

Matsui, R., Inaki, N., & Tsuji, T. (2022). Impact of GLIM Defined Malnutrition on Long Term Prognosis in Patients With Gastric Cancer After Gastrectomy. *Anticancer Res*, *42*(9), 4611-4618. https://doi.org/10.21873/anticanres.15965

Matsui, R., Inaki, N., & Tsuji, T. (2022). Impact of malnutrition as defined by the global leadership initiative on malnutrition criteria on the long-term prognosis in older patients with gastric cancer after gastrectomy. *Surg Today*. https://doi.org/10.1007/s00595-022-02594-5

Matsui, R., Inaki, N., Tsuji, T., & Fukunaga, T. (2023). Association of GLIM Defined Malnutrition According to Preoperative Chronic Inflammation with Long-Term Prognosis after Gastrectomy in Patients with Advanced Gastric Cancer. *J Clin Med*, *12*(4). https://doi.org/10.3390/jcm12041579

Matsumoto, Y., Zhou, Q., Kamimura, K., Moriyama, M., & Saijo, Y. (2018). The Prognostic Nutrition Index Predicts the Development of Hematological Toxicities in and the Prognosis of Esophageal Cancer Patients Treated with Cisplatin Plus 5-Fluorouracil Chemotherapy. *Nutr Cancer*, *70*(3), 447-452. https://doi.org/10.1080/01635581.2018.1445765

Matsunaga, T., Saito, H., Osaki, T., Fukuda, K., Fukumoto, Y., Takahashi, S., Taniguchi, K., Iwamoto, A., Kuroda, H., Katano, K., Shimizu, S., Shishido, Y., Miyatani, K., Sakamoto, T., & Fujiwara, Y. (2024). Using the geriatric nutritional risk index to predict outcomes in older patients with remnant gastric cancer after gastrectomy: a retrospective multicenter study in Japan. *Surg Today*, *54*(11), 1360-1368. https://doi.org/10.1007/s00595-024-02850-w

Matsunaga, T., Saito, H., Osaki, T., Takahashi, S., Iwamoto, A., Fukuda, K., Taniguchi, K., Kuroda, H., Takeuchi, T., Sugamura, K., Sumi, K., Katano, K., Shishido, Y., Miyatani, K., & Fujiwara, Y. (2022). Impact of geriatric nutritional risk index on outcomes after gastrectomy in elderly patients with gastric cancer: a retrospective multicenter study in Japan. *BMC Cancer*, *22*(1), 540. https://doi.org/10.1186/s12885-022-09638-6

Matsunaga, T., Satio, H., Sakano, Y., Makinoya, M., Shimizu, S., Shishido, Y., Miyatani, K., Hanaki, T., Kihara, K., Yamamoto, M., Tokuyasu, N., Takano, S., Sakamoto, T., Hasegawa, T., & Fujiwara, Y. (2024). Prognostic significance of the cachexia index in patients with unresectable advanced gastric cancer receiving palliative chemotherapy: a retrospective single-center study. *Surg Today*, *54*(3), 231-239. https://doi.org/10.1007/s00595-023-02721-w

Maurício, S. F., da Silva, J. B., Bering, T., & Correia, M. I. (2013). Relationship between nutritional status and the Glasgow Prognostic Score in patients with colorectal cancer. *Nutrition*, *29*(4), 625-629.

https://doi.org/10.1016/j.nut.2012.09.016

Migita, K., Matsumoto, S., Wakatsuki, K., Ito, M., Kunishige, T., Nakade, H., Kitano, M., Nakatani, M., & Sho, M. (2018). The prognostic significance of inflammation-based markers in patients with recurrent gastric cancer. *Surg Today*, *48*(3), 282-291. https://doi.org/10.1007/s00595-017-1582-y

Migita, K., Matsumoto, S., Wakatsuki, K., Ito, M., Kunishige, T., Nakade, H., & Sho, M. (2018). The Prognostic Significance of the Geriatric Nutritional Risk Index in Patients with Esophageal Squamous Cell Carcinoma. *Nutr Cancer*, *70*(8), 1237-1245. https://doi.org/10.1080/01635581.2018.1512640

Migita, K., Takayama, T., Saeki, K., Matsumoto, S., Wakatsuki, K., Enomoto, K., Tanaka, T., Ito, M., Kurumatani, N., & Nakajima, Y. (2013). The prognostic nutritional index predicts long-term outcomes of gastric cancer patients independent of tumor stage. *Ann Surg Oncol*, *20*(8), 2647-2654. https://doi.org/10.1245/s10434-013-2926-5

Miller, J., Dreczkowski, G., Ramage, M. I., Wigmore, S. J., Gallagher, I. J., & Skipworth, R. J. E. (2020). Adipose depot gene expression and intelectin-1 in the metabolic response to cancer and cachexia. *J Cachexia Sarcopenia Muscle*, *11*(4), 1141-1153. https://doi.org/10.1002/jcsm.12568

Mimatsu, K., Fukino, N., Ogasawara, Y., Saino, Y., & Oida, T. (2017). Utility of Inflammatory Marker- and Nutritional Status-based Prognostic Factors for Predicting the Prognosis of Stage IV Gastric Cancer Patients Undergoing Non-curative Surgery. *Anticancer Res*, *37*(8), 4215-4222. https://doi.org/10.21873/anticanres.11812

Mito, M., Sakata, J., Hirose, Y., Abe, S., Saito, S., Miura, Y., Ishikawa, H., Miura, K., Takizawa, K., Ichikawa, H., Shimada, Y., Kobayashi, T., & Wakai, T. (2022). Preoperative controlling nutritional status score predicts systemic disease recurrence in patients with resectable biliary tract cancer [Article in Press]. *European Journal of Surgical Oncology*. https://doi.org/10.1016/j.ejso.2022.11.003

Miyamoto, H., Toyokawa, T., Ishidate, T., Kuroda, K., Miki, Y., Yoshii, M., Tamura, T., Lee, S., & Maeda, K. (2024). Significance of the geriatric nutritional risk index and neutrophil-to-lymphocyte ratio as prognostic indicators in older patients with gastric cancer: a retrospective cohort study. *BMC Cancer*, *24*(1), 1396. https://doi.org/10.1186/s12885-024-13158-w

Miyazaki, T., Sakai, M., Sohda, M., Tanaka, N., Yokobori, T., Motegi, Y., Nakajima, M., Fukuchi, M., Kato, H., & Kuwano, H. (2016). Prognostic Significance of Inflammatory and Nutritional Parameters in Patients with Esophageal Cancer. *Anticancer Res*, *36*(12), 6557-6562. https://doi.org/10.21873/anticanres.11259

Mohri, Y., Inoue, Y., Tanaka, K., Hiro, J., Uchida, K., & Kusunoki, M. (2013). Prognostic nutritional index predicts postoperative outcome in colorectal cancer. *World J Surg*, *37*(11), 2688-2692. https://doi.org/10.1007/s00268-013-2156-9

Molfino, A., Carletti, R., Imbimbo, G., Amabile, M. I., Belli, R., di Gioia, C. R. T., Belloni, E., Spinelli, F., Rizzo, V., Catalano, C., Nigri, G., & Muscaritoli, M. (2022). Histomorphological and inflammatory changes of white adipose tissue in gastrointestinal cancer patients with and without cachexia [Article]. *Journal of Cachexia, Sarcopenia and Muscle*, *13*(1), 333-342. https://doi.org/10.1002/jcsm.12893

Momokita, M., Abe, A., Shibata, K., Hayashi, H., Ishihama, T., Inoue, H., Achiwa, M., Taniguchi, S., & Nakayama, A. (2022). Association between oral function and nutrition in gastric cancer patients undergoing chemotherapy. *Oral Dis*. https://doi.org/10.1111/odi.14310

Mracek, T., Stephens, N. A., Gao, D., Bao, Y., Ross, J. A., Rydén, M., Arner, P., Trayhurn, P., Fearon, K. C. H., & Bing, C. (2011). Enhanced ZAG production by subcutaneous adipose tissue is linked to weight loss in gastrointestinal cancer patients [Article]. *British Journal of Cancer*, *104*(3), 441-447. https://doi.org/10.1038/sj.bjc.6606083

Mülküt, F., Ofluoğlu, C. B., Başdoğan, M. K., Aydın İ, C., Akdoğan, O., Gündoğdu, A., & Subaşı İ, E. (2025). Prognostic value of prognostic nutritional index in patients undergoing surgery for gastric cancer. *Front Surg*, *12*, 1618111. https://doi.org/10.3389/fsurg.2025.1618111

Nagata, S., Maeda, S., Nagamatsu, S., Kai, S., Fukuyama, Y., Korematsu, S., Orita, H., Anai, H., Kuwano, H., & Korenaga, D. (2021). Prognostic Nutritional Index Considering Resection Range Is Useful for Predicting Postoperative Morbidity of Hepatectomy. *J Gastrointest Surg*, *25*(11), 2788-2795. https://doi.org/10.1007/s11605-020-04893-z

Naghashi, S., Somi, M. H., & Nikniaz, Z. (2022). Pretreatment nutritional status is associated with quality of life in patients with gastric cancer: a cross-sectional study from Iran. *Support Care Cancer*, *30*(4), 3313-3319. https://doi.org/10.1007/s00520-021-06792-8

Nakagawa, S., Yamashita, Y. I., Umezaki, N., Yamao, T., Okabe, H., Imai, K., Nitta, H., Hashimoto, D., Chikamoto, A., & Baba, H. (2018). Serum Marker Score Based on Prognostic Nutrition Index, Carcinoembryonic Antigen, and Carbohydrate Antigen 19-9 Is Associated With Recurrence for Patients Undergoing Surgery for Pancreatic Ductal Adenocarcinoma. *Pancreas*, *47*(9), 1130-1134. https://doi.org/10.1097/mpa.0000000000001146

Nakagoe, T., Tsuji, T., Sawai, T., Tanaka, K., Hidaka, S., Shibasaki, S., Nanashima, A., Ohbatake, M., Yamaguchi, H., Yasutake, T., Sugawara, K., Inokuchi, N., & Kamihira, S. (2003). Increased serum levels of interleukin-6 in malnourished patients with colorectal cancer. *Cancer Lett*, *202*(1), 109-115. https://doi.org/10.1016/j.canlet.2003.09.008

Nakamura, Y., Imada, A., Fukugaki, A., Kanto, S., Yamaura, T., Kinjo, Y., & Kuroda, N. (2022). Association of nutritional risk and systemic inflammation with survival in patients with colorectal cancer who underwent curative surgery. *Clin Nutr ESPEN*, *49*, 417-424. https://doi.org/10.1016/j.clnesp.2022.03.011

Nakamura, Y., Kawase, M., Kawabata, Y., Kanto, S., Yamaura, T., Kinjo, Y., Ogo, Y., & Kuroda, N. (2024). Impact of malnutrition on cancer recurrence, colorectal cancer-specific death, and non-colorectal cancer-related death in patients with colorectal cancer who underwent curative surgery. *J Surg Oncol*, *129*(2), 317-330. https://doi.org/10.1002/jso.27488

Nakamura, Y., Nishimura, T., Kanemitsu, E., Nagata, H., Komori, J., & Takada, Y. (2025). Usefulness of the Geriatric Nutritional Risk Index (GNRI) as a Predictor of Postoperative Complications After Colorectal Cancer Surgery. *Cureus*, *17*(6), e86268. https://doi.org/10.7759/cureus.86268 10.7759/cureus.86268. eCollection 2025 Jun.

Nakashima, K., Haruki, K., Kamada, T., Takahashi, J., Nakaseko, Y., Ohdaira, H., Furukawa, K., Suzuki, Y., & Ikegami, T. (2023). Usefulness of the cachexia index as a prognostic indicator for patients with gastric cancer. *Ann Gastroenterol Surg*, *7*(5), 733-740. https://doi.org/10.1002/ags3.12669 10.1002/ags3.12669. eCollection 2023 Sep.

Nakatani, M., Migita, K., Matsumoto, S., Wakatsuki, K., Ito, M., Nakade, H., Kunishige, T., Kitano, M., & Sho, M. (2018). Prognostic Significance of the Prognostic Nutritional Index in Patients with Recurrent Esophageal Squamous Cell Carcinoma. *Nutr Cancer*, *70*(3), 467-473. https://doi.org/10.1080/01635581.2018.1445771

Narasimhan, A., Greiner, R., Bathe, O. F., Baracos, V., & Damaraju, S. (2018). Differentially expressed alternatively spliced genes in skeletal muscle from cancer patients with cachexia [Article]. *Journal of Cachexia, Sarcopenia and Muscle*, *9*(1), 60-70. https://doi.org/10.1002/jcsm.12235

Negrichi, S., & Taleb, S. (2020). Evaluation of Nutritional Status of Colorectal Cancer Patients from Algerian East Using Anthropometric Measurements and Laboratory Assessment. *Iran J Public Health*, *49*(7), 1242-1251. https://doi.org/10.18502/ijph.v49i7.3577

Neto, N. I. P., Murari, A. S. D. P., Oyama, L. M., Otoch, J. P., Alcântara, P. S. M., Tokeshi, F., Figuerêdo, R. G., Alves, M. J., Lima, J. D. C. C., Matos-Neto, E. M. D., Seelaender, M., & Oller do Nascimento, C. M. (2018). Peritumoural adipose tissue pro-inflammatory cytokines are associated with tumoural growth factors in cancer cachexia patients [Article]. *Journal of Cachexia, Sarcopenia and Muscle*, *9*(6), 1101-1108. https://doi.org/10.1002/jcsm.12345

Ngoc Anh, L. T., Kien, T. G., Tuan, N. V., Tuong, T. T. A., Ko, J., Dan, P. T., Cho, J., & Tap, N. V. (2025). Malnutrition in Colorectal Cancer Patients: Association with the Lack of Eating Motivation and Inappropriate Diet.

Asian Pac J Cancer Prev, *26*(5), 1661-1670. https://doi.org/10.31557/apjcp.2025.26.5.1661

Nie, R., Yuan, S., Chen, S., Chen, X., Chen, Y., Zhu, B., Qiu, H., Zhou, Z., Peng, J., & Chen, Y. (2016). Prognostic nutritional index is an independent prognostic factor for gastric cancer patients with peritoneal dissemination. *Chin J Cancer Res*, *28*(6), 570-578. https://doi.org/10.21147/j.issn.1000-9604.2016.06.03

Nie, X., Nie, X., Xie, Q., Li, C., He, W., Ni, K., Lu, S., Wang, C., Miao, Y., Jiang, L., Wang, K., Liu, G., Fang, Q., Peng, L., Shi, Q., Kanamori, J., Aiolfi, A., Deana, C., Han, Y., & Leng, X. (2025). Patient-reported outcomes of the prognostic nutritional index for resectable esophageal squamous cell carcinoma. *J Thorac Dis*, *17*(7), 5133-5145. https://doi.org/10.21037/jtd-2025-1229

Nishibeppu, K., Kubota, T., Yubakami, M., Ohashi, T., Kiuchi, J., Shimizu, H., Arita, T., Yamamoto, Y., Konishi, H., Morimura, R., Shiozaki, A., Ikoma, H., Kuriu, Y., Fujiwara, H., & Otsuji, E. (2024). Impact of hypoglycemia after gastrectomy on Global Leader Initiative on Malnutrition-defined malnutrition: a retrospective study. *Surg Today*, *54*(7), 743-750. https://doi.org/10.1007/s00595-024-02799-w

Nishiyama, T., Shibutani, M., Tanda, H., Seki, Y., Kashiwagi, S., Kasashima, H., Fukuoka, T., & Maeda, K. (2025). Prognostic Value of the Modified Cachexia Index in Colorectal Cancer Patients Undergoing Curative Surgery. *Cancer Diagn Progn*, *5*(1), 89-94. https://doi.org/10.21873/cdp.10416

Nogueiro, J., Santos-Sousa, H., Pereira, A., Devezas, V., Fernandes, C., Sousa, F., Fonseca, T., Barbosa, E., & Barbosa, J. A. (2022). The impact of the prognostic nutritional index (PNI) in gastric cancer. *Langenbecks Arch Surg*, *407*(7), 2703-2714. https://doi.org/10.1007/s00423-022-02627-0

Nonogaki, I., Kanda, M., Shimizu, D., Inokawa, Y., Hattori, N., Hayashi, M., Tanaka, C., Koike, M., Nakayama, G., & Kodera, Y. (2023). Controlling Nutritional Status Score Serves as a Prognosticator in Esophageal Squamous Cell Carcinoma: Optimal Timing of Evaluation of Patients Undergoing Neoadjuvant Treatment. *World J Surg*, *47*(1), 217-226. https://doi.org/10.1007/s00268-022-06773-w

Nozoe, T., Kohno, M., Iguchi, T., Mori, E., Maeda, T., Matsukuma, A., & Ezaki, T. (2012). The prognostic nutritional index can be a prognostic indicator in colorectal carcinoma. *Surg Today*, *42*(6), 532-535. https://doi.org/10.1007/s00595-011-0061-0

Nozoe, T., Ninomiya, M., Maeda, T., Matsukuma, A., Nakashima, H., & Ezaki, T. (2010). Prognostic nutritional index: a tool to predict the biological aggressiveness of gastric carcinoma. *Surg Today*, *40*(5), 440-443. https://doi.org/10.1007/s00595-009-4065-y

Obaid, R., & Alkazemi, D. (2025). Malnutrition and Nutrition Impact Symptoms in Kuwaiti Colorectal Cancer Patients: Validation of PG-SGA Short Form. *Nutrients*, *17*(17). https://doi.org/10.3390/nu17172770 10.3390/nu17172770.

Ogata, T., Yoshida, N., Sadakari, Y., Iwanaga, A., Nakane, H., Okawara, K., Endo, K., Kaneshiro, K., Hirokata, G., Aoyagi, T., Shima, H., & Taniguchi, M. (2022). Colorectal cancer surgery in elderly patients 80 years and older: a comparison with younger age groups. *J Gastrointest Oncol*, *13*(1), 137-148. https://doi.org/10.21037/jgo-21-627

Ogawa, D., Miyata, T., Yumoto, S., Shiraishi, Y., Matsumoto, T., Takematsu, T., Tsukamoto, M., Nakagawa, S., Mima, K., Nitta, H., Hayashi, H., & Baba, H. (2024). Prognostic value of preoperative geriatric nutritional risk index in intrahepatic cholangiocarcinoma after hepatectomy: a single-center retrospective cohort study. *Langenbecks Arch Surg*, *409*(1), 47. https://doi.org/10.1007/s00423-023-03221-8

Oh, C. A., Kim, D. H., Oh, S. J., Choi, M. G., Noh, J. H., Sohn, T. S., Bae, J. M., & Kim, S. (2012). Nutritional risk index as a predictor of postoperative wound complications after gastrectomy. *World J Gastroenterol*, *18*(7), 673-678. https://doi.org/10.3748/wjg.v18.i7.673

Oh, S. E., Park, J. S., & Jeung, H. C. (2022). Pre-treatment Nutritional Risk Assessment by NRS-2002 Predicts Prognosis in Patients With Advanced Biliary Tract Cancer: A Single Center Retrospective Study. *Clin Nutr Res*, *11*(3), 183-193. https://doi.org/10.7762/cnr.2022.11.3.183

Ohama, H., Hiraoka, A., Tada, T., Hirooka, M., Kariyama, K., Tani, J., Atsukawa, M., Takaguchi, K., Itobayashi,

E., Fukunishi, S., Tsuji, K., Ishikawa, T., Tajiri, K., Ochi, H., Yasuda, S., Toyoda, H., Ogawa, C., Nishimura, T., Hatanaka, T., Kakizaki, S., Shimada, N., Kawata, K., Naganuma, A., Kosaka, H., Matono, T., Kuroda, H., Yata, Y., Tanaka, H., Nishikawa, H., Shibata, H., Tada, F., Nouso, K., Morishita, A., Tsutsui, A., Nagano, T., Itokawa, N., Okubo, T., Arai, T., Imai, M., Koizumi, Y., Nakamura, S., Iijima, H., Kaibori, M., Hiasa, Y., & Kumada, T. (2025). Geriatric nutritional risk index and newly developed scoring system as prognosis prediction for unresectable hepatocellular carcinoma patients treated with lenvatinib. *Sci Rep*, *15*(1), 72. https://doi.org/10.1038/s41598-024-78539-4

Ohara, T., Iwai, N., Oka, K., Okabe, K., Sakai, H., Tsuji, T., Okuda, T., Sakagami, J., Kagawa, K., Doi, T., Inoue, K., Dohi, O., Yoshida, N., Yamaguchi, K., Moriguchi, M., Uchiyama, K., Ishikawa, T., Takagi, T., Konishi, H., & Itoh, Y. (2025). Clinical significance of cachexia index determined by bioelectrical impedance analysis in patients with gastrointestinal cancer. *Oncol Lett*, *29*(3), 114. https://doi.org/10.3892/ol.2024.14860

Okada, G., Matsumoto, Y., Habu, D., Matsuda, Y., Lee, S., & Osugi, H. (2021). Relationship between GLIM criteria and disease-specific symptoms and its impact on 5-year survival of esophageal cancer patients. *Clin Nutr*, *40*(9), 5072-5078. https://doi.org/10.1016/j.clnu.2021.08.008

Okadome, K., Baba, Y., Yagi, T., Kiyozumi, Y., Ishimoto, T., Iwatsuki, M., Miyamoto, Y., Yoshida, N., Watanabe, M., & Baba, H. (2020). Prognostic Nutritional Index, Tumor-infiltrating Lymphocytes, and Prognosis in Patients with Esophageal Cancer. *Ann Surg*, *271*(4), 693-700. https://doi.org/10.1097/sla.0000000000002985

Okamoto, A., Furukawa, K., Ohkuma, M., Nakano, T., Yoshioka, S., Imaizumi, Y., Sugano, H., Takeda, Y., Kosuge, M., & Eto, K. (2023). Clinical Significance of Controlling Nutritional Status (CONUT) Score in Patients With Colorectal Liver Metastases After Hepatectomy. *In Vivo*, *37*(6), 2678-2686. https://doi.org/10.21873/invivo.13377

Okamura, Y., Sugiura, T., Ito, T., Yamamoto, Y., Ashida, R., & Uesaka, K. (2017). The optimal cut-off value of the preoperative prognostic nutritional index for the survival differs according to the TNM stage in hepatocellular carcinoma. *Surg Today*, *47*(8), 986-993. https://doi.org/10.1007/s00595-017-1491-0

Okubo, K., Arigami, T., Matsushita, D., Tanaka, T., Tsuruda, Y., Noda, M., Sasaki, K., Mori, S., Kurahara, H., & Ohtsuka, T. (2021). Clinical Impact of the Prognostic Nutritional Index as a Predictor of Outcomes in Patients with Stage II/III Gastric Cancer: A Retrospective Cohort Study. *Oncology*, *99*(6), 380-388. https://doi.org/10.1159/000514572

Olaechea, S., Gannavarapu, B. S., Gilmore, A., Alvarez, C., Iyengar, P., & Infante, R. (2021). The influence of tumour fluorodeoxyglucose avidity and cachexia development on patient survival in oesophageal or gastroesophageal junction cancer. *JCSM Clin Rep*, *6*(4), 128-136. https://doi.org/10.1002/crt2.42

Olaechea, S., Sarver, B., Liu, A., Gilmore, L. A., Alvarez, C., Iyengar, P., & Infante, R. (2023). Race, Ethnicity, and Socioeconomic Factors as Determinants of Cachexia Incidence and Outcomes in a Retrospective Cohort of Patients With Gastrointestinal Tract Cancer. *JCO Oncol Pract*, *19*(7), 493-500. https://doi.org/10.1200/op.22.00674

Omiya, S., Urade, T., Komatsu, S., Kido, M., Kuramitsu, K., Yanagimoto, H., Toyama, H., & Fukumoto, T. (2023). Impact of GLIM criteria-based malnutrition diagnosis on outcomes following liver resection for hepatocellular carcinoma. *HPB (Oxford)*, *25*(12), 1555-1565. https://doi.org/10.1016/j.hpb.2023.08.012

Onishi, S., Tajika, M., Tanaka, T., Yamada, K., Abe, T., Higaki, E., Hosoi, T., Inaba, Y., Muro, K., Shimizu, M., & Niwa, Y. (2020). Prognostic Impact of Sarcopenic Obesity after Neoadjuvant Chemotherapy Followed by Surgery in Elderly Patients with Esophageal Squamous Cell Carcinoma. *J Clin Med*, *9*(9). https://doi.org/10.3390/jcm9092974

Onuma, S., Hashimoto, I., Suematsu, H., Nagasawa, S., Kanematsu, K., Aoyama, T., Yamada, T., Rino, Y., Ogata, T., & Oshima, T. (2023). Clinical Effects of the Neutrophil-to-Lymphocyte Ratio/Serum Albumin Ratio in Patients with Gastric Cancer after Gastrectomy. *J Pers Med*, *13*(3). https://doi.org/10.3390/jpm13030432

Ose, J., Gigic, B., Lin, T., Liesenfeld, D. B., Böhm, J., Nattenmüller, J., Scherer, D., Zielske, L., Schrotz-King, P., Habermann, N., Ochs-Balcom, H. M., Peoples, A. R., Hardikar, S., Li, C. I., Shibata, D., Figueiredo, J., Toriola, A. T., Siegel, E. M., Schmit, S., Schneider, M., Ulrich, A., Kauczor, H. U., & Ulrich, C. M. (2019). Multiplatform Urinary Metabolomics Profiling to Discriminate Cachectic from Non-Cachectic Colorectal Cancer Patients: Pilot Results from

the ColoCare Study. *Metabolites*, *9*(9). https://doi.org/10.3390/metabo9090178

Özcan, P., & Çarkman, M. S. (2024). The relationship between the Prognostic Nutritional Index and lymphovascular and perineural invasion of the tumor in patients diagnosed with gastric cancer, and its effect on overall survival. *Medicine (Baltimore)*, *103*(42), e40087. https://doi.org/10.1097/md.0000000000040087

Paillaud, E., Liuu, E., Laurent, M., Le Thuaut, A., Vincent, H., Raynaud-Simon, A., Bastuji-Garin, S., Tournigand, C., Caillet, P., & Canoui-Poitrine, F. (2014). Geriatric syndromes increased the nutritional risk in elderly cancer patients independently from tumour site and metastatic status. The ELCAPA-05 cohort study. *Clin Nutr*, *33*(2), 330-335. https://doi.org/10.1016/j.clnu.2013.05.014

Pan, P., Tao, G., & Sun, X. (2015). Subjective global assessment and prealbumin levels of esophageal cancer patients undergoing concurrent chemoradiotherapy. *Nutr Hosp*, *31*(5), 2167-2173. https://doi.org/10.3305/nh.2015.31.5.8596

Pan, Y., Ma, Y., & Dai, G. (2023). The Prognostic Value of the Prognostic Nutritional Index in Patients with Advanced or Metastatic Gastric Cancer Treated with Immunotherapy. *Nutrients*, *15*(19). https://doi.org/10.3390/nu15194290

Park, J. H., Kim, E., Seol, E. M., Kong, S. H., Park, D. J., Yang, H. K., Choi, J. H., Park, S. H., Choe, H. N., Kweon, M., Park, J., Choi, Y., & Lee, H. J. (2021). Prediction Model for Screening Patients at Risk of Malnutrition After Gastric Cancer Surgery. *Ann Surg Oncol*, *28*(8), 4471-4481. https://doi.org/10.1245/s10434-020-09559-3

Park, J. S., Kim, H.-m., Jeung, H.-C., & Kang, S. A. (2019). Association between early nutritional risk and overall survival in patients with advanced pancreatic cancer: A single-center retrospective study. *Clinical Nutrition ESPEN*, *30*, 94-99. https://doi.org/https://doi.org/10.1016/j.clnesp.2019.01.012

Park, S. H., Lee, S., Song, J. H., Choi, S., Cho, M., Kwon, I. G., Son, T., Kim, H. I., Cheong, J. H., Hyung, W. J., Choi, S. H., Noh, S. H., & Choi, Y. Y. (2020). Prognostic significance of body mass index and prognostic nutritional index in stage II/III gastric cancer [Article]. *European Journal of Surgical Oncology*, *46*(4), 620-625. https://doi.org/10.1016/j.ejso.2019.10.024

Patil, P. S., Dhingra, J. S., & Mehta, S. (2017). Profile of gastric adenocarcinoma in patients younger than 40 years-a single center experience from India [Conference Abstract]. *Annals of Oncology*, *28*, x64-x65. https://doi.org/10.1093/annonc/mdx660.025

Patil, P. S., Dhingra, J. S., & Mehta, S. (2017). Profile of signet ring cell carcinoma histology gastric cancer in a tertiary cancer center in India [Conference Abstract]. *Journal of Gastroenterology and Hepatology*, *32*, 91. https://doi.org/10.1111/jgh.13875

Peng, J., Zhang, R., Zhao, Y., Wu, X., Chen, G., Wan, D., Lu, Z., & Pan, Z. (2017). Prognostic value of preoperative prognostic nutritional index and its associations with systemic inflammatory response markers in patients with stage III colon cancer. *Chin J Cancer*, *36*(1), 96. https://doi.org/10.1186/s40880-017-0260-1

Peng, W., Yao, M., Zou, K., Li, C., Wen, T., & Sun, X. (2021). Postoperative controlling nutritional status score is an independent risk factor of survival for patients with small hepatocellular carcinoma: a retrospective study. *BMC Surg*, *21*(1), 338. https://doi.org/10.1186/s12893-021-01334-9

Pérez-Cruz, E., & Camacho-Limas, C. P. (2017). Association of nutritional status and functional capacity in gastrointestinal cancer patients [Article]. *Gaceta Medica de Mexico*, *153*(5), 575-580. https://doi.org/10.24875/GMM.17002776

Permuth, J. B., Park, M. A., Chen, D. T., Basinski, T., Powers, B. D., Gwede, C. K., Dezsi, K. B., Gomez, M., Vyas, S. L., Biachi, T., Cortizas, E. M., Crowder, S., Genilo-Delgado, M., Green, B., Greene, A., Gregg, C., Hoffe, S. E., Jiang, K., Kim, B., Vasudevan, V., Garcialopez De Llano, J., Menon, A. A., Mo, Q., MorenoUrazan, L. M., Mok, S., Parker, N., Rajasekhara, S., Rasool, G., Sinnamon, A., Sparks, L., Stewart, P. A., Tardif, K., Tassielli, A. F., Teer, J. K., Tran, D. V., Turner, K. L., Vadaparampil, S. T., Whelan, C. J., Douglas, W. G., Velanovich, V., Karachristos, A., Legaspi, A., Meredith, K., Molina-Vega, M. A., Huguet, K. L., Arnoletti, J. P., Bloomston, M., Trevino, J., Merchant, N. B.,

Pimiento, J. M., Hodul, P. J., Malafa, M., Fleming, J., Judge, S. M., Jeong, D. K., & Judge, A. (2024). Leveraging real-world data to predict cancer cachexia stage, quality of life, and survival in a racially and ethnically diverse multi-institutional cohort of treatment-naïve patients with pancreatic ductal adenocarcinoma [Article]. *Frontiers in Oncology*, *14*. https://doi.org/10.3389/fonc.2024.1362244

Persano, M., Rimini, M., Tada, T., Suda, G., Shimose, S., Kudo, M., Cheon, J., Finkelmeier, F., Lim, H. Y., Presa, J., Masi, G., Yoo, C., Lonardi, S., Stefanini, B., Kumada, T., Sakamoto, N., Iwamoto, H., Aoki, T., Chon, H. J., Himmelsbach, V., Montes, M., Vivaldi, C., Soldà, C., Hiraoka, A., Sho, T., Niizeki, T., Nishida, N., Steup, C., Hirooka, M., Kariyama, K., Tani, J., Atsukawa, M., Takaguchi, K., Itobayashi, E., Fukunishi, S., Tsuji, K., Ishikawa, T., Tajiri, K., Ochi, H., Yasuda, S., Toyoda, H., Ogawa, C., Nishimura, T., Hatanaka, T., Kakizaki, S., Shimada, N., Kawata, K., Tada, F., Ohama, H., Nouso, K., Morishita, A., Tsutsui, A., Nagano, T., Itokawa, N., Okubo, T., Arai, T., Imai, M., Kosaka, H., Naganuma, A., Koizumi, Y., Nakamura, S., Kaibori, M., Iijima, H., Hiasa, Y., Burgio, V., Della Corte, A., Ratti, F., De Cobelli, F., Aldrighetti, L., Scartozzi, M., Cascinu, S., & Casadei-Gardini, A. (2023). Role of the Prognostic Nutritional Index in Predicting Survival in Advanced Hepatocellular Carcinoma Treated with Atezolizumab Plus Bevacizumab. *Oncology*, *101*(5), 283-291. https://doi.org/10.1159/000528818

Pian, G., & Oh, S. Y. (2022). Comparison of nutritional and immunological scoring systems predicting prognosis in T1-2N0 colorectal cancer. *Int J Colorectal Dis*, *37*(1), 179-188. https://doi.org/10.1007/s00384-021-04043-0

Piciucchi, M., Larghi, A., Valente, R., Archibugi, L., Delle Fave, G., & Capurso, G. (2013). Clinical features and outcome of pancreatic adenocarcinoma presenting with cachexia [Conference Abstract]. *Pancreatology*, *13*(3), S78-S79.

Piciucchi, M., Valente, R., Larghi, A., Stigliano, S., Archibugi, L., Signoretti, M., Cavallini, M., Costamagna, G., Ziparo, V., Delle Fave, G., & Capurso, G. (2013). Clinical features and outcome of pancreatic adenocarcinoma patients presentingwith cachexia [Conference Abstract]. *Digestive and Liver Disease*, *45*, S161-S162.

Piciucchi, M., Valente, R., Stigliano, S., Archibugi, L., Zerboni, G., Signoretti, M., Larghi, A., Costamagna, G., Fave, G. D., & Capurso, G. (2013). Cachexia is an Insidious Symptom of Pancreatic Ductal Adenocarcinoma Associated with Delayed Diagnosis and Advanced Stage of Disease [Conference Abstract]. *Journal of the Pancreas*, *14*, 589.

Qi, Q., Song, Q., Cheng, Y., & Wang, N. (2021). Prognostic significance of preoperative prognostic nutritional index for overall survival and postoperative complications in esophageal cancer patients [Article]. *Cancer Management and Research*, *13*, 8585-8597. https://doi.org/10.2147/CMAR.S333190

Qian, Y., Liu, H., Pan, J., Yu, W., Lv, J., Yan, J., Gao, J., Wang, X., Ge, X., & Zhou, W. (2021). Preoperative Controlling Nutritional Status (CONUT) score predicts short-term outcomes of patients with gastric cancer after laparoscopy-assisted radical gastrectomy [Article]. *World Journal of Surgical Oncology*, *19*(1). https://doi.org/10.1186/s12957-021-02132-6

Qin, L., Tian, Q., Zhu, W., & Wu, B. (2021). The Validity of the GLIM Criteria for Malnutrition in Hospitalized Patients with Gastric Cancer [Article]. *Nutrition and Cancer*, *73*(11-12), 2732-2739. https://doi.org/10.1080/01635581.2020.1856894

Qin, Y., Xie, H., Liu, T., Zhang, H., Liu, C., Li, X., Bu, Z., Liu, X., Lin, S., Chen, Y., Zheng, X., Zhao, H., Shi, J., & Shi, H. (2024). Prognostic value of the fat-free mass index-based cachexia index in patients with colorectal cancer. *Scientific reports*, *14*(1), 24390-NA. https://doi.org/10.1038/s41598-024-75485-z

Qiu, M., Zhou, Y. X., Jin, Y., Wang, Z. X., Wei, X. L., Han, H. Y., Ye, W. F., Zhou, Z. W., Zhang, D. S., Wang, F. H., Li, Y. H., Yang, D. J., & Xu, R. H. (2015). Nutrition support can bring survival benefit to high nutrition risk gastric cancer patients who received chemotherapy [Article]. *Supportive Care in Cancer*, *23*(7), 1933-1939. https://doi.org/10.1007/s00520-014-2523-6

Qu, Z., Lu, Y.-J., Feng, J.-W., Chen, Y.-X., Shi, L.-Q., Chen, J., Rambaran, N., Duan, Y.-F., & He, X.-Z. (2022).

Preoperative Prognostic Nutritional Index and Neutrophil-to-Lymphocyte Ratio Predict Survival Outcomes of Patients With Hepatocellular Carcinoma After Curative Resection. *Frontiers in oncology*, *11*, 823054-NA. https://doi.org/10.3389/fonc.2021.823054

Ræder, H., Henriksen, C., Bøhn, S. K., O'de Fey Vilbo, A. R., Henriksen, H. B., Kværner, A. S., Rolid, K., Paur, I., Smeland, S., & Blomhoff, R. (2018). Agreement between PG-SGA category and fat-free mass in colorectal cancer patients [Article]. *Clinical Nutrition ESPEN*, *27*, 24-31. https://doi.org/10.1016/j.clnesp.2018.07.005

Ren, W., Wang, H., Xiang, T., & Liu, G. (2023). Role of preoperative peripheral blood inflammatory parameters and postoperative lymph-node ratio in prognosis of patients with gastric cancer undergoing chemotherapy [Article]. *Cancer Research on Prevention and Treatment*, *50*(5), 490-497. https://doi.org/10.3971/j.issn.1000-8578.2023.22.1218

Riad, A., Knight, S. R., Ghosh, D., Kingsley, P. A., Lapitan, M. C., Parreno-Sacdalan, M. D., Sundar, S., Qureshi, A. U., Valparaiso, A. P., Pius, R., Shaw, C. A., Drake, T. M., Norman, L., Ademuyiwa, A. O., Adisa, A. O., Aguilera, M. L., Al-Saqqa, S. W., Al-Slaibi, I., Bhangu, A., Biccard, B. M., Brocklehurst, P., Burden, S., Chu, K., Costas-Chavarri, A., Dare, A. J., Elhadi, M., Fairfield, C. J., Fitzgerald, J. E., Glasbey, J., van Berge Henegouwen, M. I., Ingabire, J. C. A., Kingham, T. P., Lawani, I., Lieske, B., Lilford, R., Magill, L., Maimbo, M., Martin, J., Mathai, S., McLean, K. A., Moore, R., Morton, D., Nepogodiev, D., Norrie, J., Ntirenganya, F., Pata, F., Pinkney, T., Kottayasamy Seenivasagam, R., Ramos-De la Medina, A., Roberts, T. E., Salem, H. K., Simões, J., Skipworth, R. J., Spence, R. T., Smart, N., Tabiri, S., Theodoratou, E., Thomas, H., Weiser, T. G., West, M., Whitaker, J., Yenli, E., & Harrison, E. M. (2023). Impact of malnutrition on early outcomes after cancer surgery: an international, multicentre, prospective cohort study [Article]. *The Lancet Global Health*, *11*(3), e341-e349. https://doi.org/10.1016/S2214-109X(22)00550-2

Rich, N. E., Phen, S., Desai, N., Mittal, S., Yopp, A. C., Yang, J. D., Marrero, J. A., Iyengar, P., Infante, R. E., & Singal, A. G. (2022). Cachexia is Prevalent in Patients With Hepatocellular Carcinoma and Associated With Worse Prognosis [Article]. *Clinical Gastroenterology and Hepatology*, *20*(5), e1157-e1169. https://doi.org/10.1016/j.cgh.2021.09.022

Rivelsrud, M., Paur, I., Sygnestveit, K., Nilsen, R. M., & Tangvik, R. J. (2021). Nutritional treatment is associated with longer survival in patients with pancreatic disease and concomitant risk of malnutrition. *Clin Nutr*, *40*(4), 2128-2137. https://doi.org/10.1016/j.clnu.2020.09.037

Ruan, G. T., Xie, H. L., Yuan, K. T., Lin, S. Q., Zhang, H. Y., Liu, C. A., Shi, J. Y., Ge, Y. Z., Song, M. M., Hu, C. L., Zhang, X. W., Liu, X. Y., Yang, M., Wang, K. H., Zheng, X., Chen, Y., Hu, W., Cong, M. H., Zhu, L. C., Deng, L., & Shi, H. P. (2023). Prognostic value of systemic inflammation and for patients with colorectal cancer cachexia. *J Cachexia Sarcopenia Muscle*, *14*(6), 2813-2823. https://doi.org/10.1002/jcsm.13358

Ryo, S., Kanda, M., Ito, S., Mochizuki, Y., Teramoto, H., Ishigure, K., Murai, T., Asada, T., Ishiyama, A., Matsushita, H., Tanaka, C., Kobayashi, D., Fujiwara, M., Murotani, K., & Kodera, Y. (2019). The Controlling Nutritional Status Score Serves as a Predictor of Short- and Long-Term Outcomes for Patients with Stage 2 or 3 Gastric Cancer: Analysis of a Multi-institutional Data Set. *Ann Surg Oncol*, *26*(2), 456-464. https://doi.org/10.1245/s10434-018-07121-w

Ryu, T., Takami, Y., Wada, Y., Sasaki, S., & Saitsu, H. (2022). Predictive impact of the prognostic nutritional index in early-staged hepatocellular carcinoma after operative microwave ablation. *Asian J Surg*, *45*(1), 202-207. https://doi.org/10.1016/j.asjsur.2021.04.043

Sachlova, M., Majek, O., & Tucek, S. (2014). Prognostic value of scores based on malnutrition or systemic inflammatory response in patients with metastatic or recurrent gastric cancer. *Nutr Cancer*, *66*(8), 1362-1370. https://doi.org/10.1080/01635581.2014.956261

Saito, H., Kono, Y., Murakami, Y., Kuroda, H., Matsunaga, T., Fukumoto, Y., & Osaki, T. (2017). Influence of prognostic nutritional index and tumor markers on survival in gastric cancer surgery patients. *Langenbecks Arch Surg*, *402*(3), 501-507. https://doi.org/10.1007/s00423-017-1572-y

Saito, Y., Imura, S., Morine, Y., Ikemoto, T., Iwahashi, S., Yoshikawa, M., Yoshimoto, T., & Shimada, M. (2017). Preoperative prognostic nutritional index predicts both short and long-term outcomes after liver resection for hepatocellular carcinoma [Conference Abstract]. *Journal of the American College of Surgeons*, *225*(4), e126.

Saito, Y., Imura, S., Morine, Y., Ikemoto, T., Yamada, S., & Shimada, M. (2021). Preoperative prognostic nutritional index predicts short- and long-term outcomes after liver resection in patients with hepatocellular carcinoma [Article]. *Oncology Letters*, *21*(2). https://doi.org/10.3892/ol.2020.12414

Sakamoto, T., Yagyu, T., Uchinaka, E., Miyatani, K., Hanaki, T., Kihara, K., Matsunaga, T., Yamamoto, M., Tokuyasu, N., Honjo, S., & Fujiwara, Y. (2021). The prognostic significance of combined geriatric nutritional risk index and psoas muscle volume in older patients with pancreatic cancer. *BMC Cancer*, *21*(1), 342. https://doi.org/10.1186/s12885-021-08094-y

Sakanaka, K., Fujii, K., Ishida, Y., Miyamoto, S., Horimatsu, T., Muto, M., & Mizowaki, T. (2019). Nutritional and clinical outcomes of chemoradiotherapy for clinical T1N0M0 esophageal carcinoma. *Cancer Manag Res*, *11*, 3623-3630. https://doi.org/10.2147/cmar.S189518

Sakashita, K., Otsuka, S., Ashida, R., Ohgi, K., Kato, Y., Dei, H., Notsu, A., Uesaka, K., & Sugiura, T. (2025). Prognostic significance of the cachexia index for patients with perihilar cholangiocarcinoma. *Surgery*, *182*, 109344. https://doi.org/10.1016/j.surg.2025.109344

Sakurai, K., Ohira, M., Tamura, T., Toyokawa, T., Amano, R., Kubo, N., Tanaka, H., Muguruma, K., Yashiro, M., Maeda, K., & Hirakawa, K. (2016). Predictive Potential of Preoperative Nutritional Status in Long-Term Outcome Projections for Patients with Gastric Cancer. *Ann Surg Oncol*, *23*(2), 525-533. https://doi.org/10.1245/s10434-015-4814-7

Sakurai, K., Tamura, T., Toyokawa, T., Amano, R., Kubo, N., Tanaka, H., Muguruma, K., Yashiro, M., Maeda, K., Ohira, M., & Hirakawa, K. (2016). Low Preoperative Prognostic Nutritional Index Predicts Poor Survival Post-gastrectomy in Elderly Patients with Gastric Cancer. *Ann Surg Oncol*, *23*(11), 3669-3676. https://doi.org/10.1245/s10434-016-5272-6

Santos, C. A., Santos, I. M., Mendes, L., & Mansinho, H. (2021). Gastric cancer: nutritional and functional status & survival time/mortality [Conference Abstract]. *Clinical Nutrition ESPEN*, *46*, S720-S721. https://doi.org/10.1016/j.clnesp.2021.09.501

Santos, I., Mendes, L., Mansinho, H., & Santos, C. A. (2021). Nutritional status and functional status of the pancreatic cancer patients and the impact of adjacent symptoms. *Clin Nutr*, *40*(11), 5486-5493. https://doi.org/10.1016/j.clnu.2021.09.019

Sasahara, M., Kanda, M., Ito, S., Mochizuki, Y., Teramoto, H., Ishigure, K., Murai, T., Asada, T., Ishiyama, A., Matsushita, H., Tanaka, C., Kobayashi, D., Fujiwara, M., Murotani, K., & Kodera, Y. (2020). The Preoperative Prognostic Nutritional Index Predicts Short-Term and Long-Term Outcomes of Patients with Stage II/III Gastric Cancer: Analysis of a Multi-Institution Dataset. *Dig Surg*, *37*(2), 135-144. https://doi.org/10.1159/000497454

Sasaki, M., Miyoshi, N., Fujino, S., Ishikawa, S., Saso, K., Takahashi, H., Haraguchi, N., Hata, T., Matsuda, C., Mizushima, T., Doki, Y., & Mori, M. (2019). Development of Novel Prognostic Prediction Models including the Prognostic Nutritional Index for Patients with Colorectal Cancer after Curative Resection. *J Anus Rectum Colon*, *3*(3), 106-115. https://doi.org/10.23922/jarc.2018-041

Sasaki, M., Miyoshi, N., Fujino, S., Ogino, T., Takahashi, H., Uemura, M., Matsuda, C., Yamamoto, H., Mizushima, T., Mori, M., & Doki, Y. (2020). The Geriatric Nutritional Risk Index predicts postoperative complications and prognosis in elderly patients with colorectal cancer after curative surgery. *Sci Rep*, *10*(1), 10744. https://doi.org/10.1038/s41598-020-67285-y

Sato, R., Oikawa, M., Kakita, T., Okada, T., Abe, T., Tsuchiya, H., Akazawa, N., Ohira, T., Harada, Y., Okano, H., Ito, K., & Tsuchiya, T. (2023). Low Geriatric Nutritional Risk Index (GNRI) Predicts Poorer Survival in Patients with Obstructive Colorectal Cancer Who Had a Self-Expandable Metallic Stent (SEMS) Inserted as a Bridge to Curative

Surgery. *J Anus Rectum Colon*, *7*(2), 63-73. https://doi.org/10.23922/jarc.2022-053

Sato, R., Oikawa, M., Kakita, T., Okada, T., Abe, T., Yazawa, T., Tsuchiya, H., Akazawa, N., Sato, M., Ohira, T., Harada, Y., Okano, H., Ito, K., & Tsuchiya, T. (2020). The prognostic value of the prognostic nutritional index and inflammation-based markers in obstructive colorectal cancer. *Surg Today*, *50*(10), 1272-1281. https://doi.org/10.1007/s00595-020-02007-5

Sato, R., Oikawa, M., Kakita, T., Okada, T., Abe, T., Yazawa, T., Tsuchiya, H., Akazawa, N., Sato, M., Ohira, T., Harada, Y., Okano, H., Ito, K., & Tsuchiya, T. (2021). The Controlling Nutritional Status (CONUT) Score as a prognostic factor for obstructive colorectal cancer patients received stenting as a bridge to curative surgery. *Surg Today*, *51*(1), 144-152. https://doi.org/10.1007/s00595-020-02066-8

Schütte, K., Tippelt, B., Schulz, C., Röhl, F. W., Feneberg, A., Seidensticker, R., Arend, J., & Malfertheiner, P. (2015). Malnutrition is a prognostic factor in patients with hepatocellular carcinoma (HCC). *Clin Nutr*, *34*(6), 1122-1127. https://doi.org/10.1016/j.clnu.2014.11.007

Senger, A. S., Dincer, M., Uzun, O., Gulmez, S., Avan, D., Ofluoglu, C. B., Polat, E., & Duman, M. (2022). Impact of preoperative prognostic nutritional index levels on morbidity in colorectal cancer surgery [Article]. *Annali italiani di chirurgia*, *92*, 97-101.

Serna Thomé, M. G., Leal Gonzalez, G. D., Álvarez del Castillo, H., López Basave, H. N., Padilla Rosciano, A. E., Miranda Devora, G., & Meneses García, A. A. (2020). C-Reactive protein and nutrition in oncology patients [Conference Abstract]. *Clinical Nutrition ESPEN*, *40*, 552. https://doi.org/10.1016/j.clnesp.2020.09.439

Shen, N., Wen, J., Chen, C., Chen, X., Zhang, W., Garijo, P. D., Wei, M. Y., Chen, W., Xue, X., & Sun, X. (2023). The relationship between GLIM-malnutrition, post-operative complications and long-term prognosis in elderly patients undergoing colorectal cancer surgery. *J Gastrointest Oncol*, *14*(5), 2134-2145. https://doi.org/10.21037/jgo-23-543

Shibutani, M., Kashiwagi, S., Fukuoka, T., Iseki, Y., Kasashima, H., & Maeda, K. (2023). Impact of Preoperative Nutritional Status on Long-term Survival in Patients With Stage I-III Colorectal Cancer. *In Vivo*, *37*(4), 1765-1774. https://doi.org/10.21873/invivo.13265

Shibutani, M., Maeda, K., Nagahara, H., Ohtani, H., Iseki, Y., Ikeya, T., Sugano, K., & Hirakawa, K. (2015). The prognostic significance of the postoperative prognostic nutritional index in patients with colorectal cancer. *BMC Cancer*, *15*, 521. https://doi.org/10.1186/s12885-015-1537-x

Shim, H., Cheong, J. H., Lee, K. Y., Lee, H., Lee, J. G., & Noh, S. H. (2013). Perioperative nutritional status changes in gastrointestinal cancer patients. *Yonsei Med J*, *54*(6), 1370-1376. https://doi.org/10.3349/ymj.2013.54.6.1370

Shimagaki, T., Sugimachi, K., Tomino, T., Onishi, E., Koga, N., Kasagi, Y., Sugiyama, M., Kimura, Y., & Morita, M. (2025). Cachexia index as a prognostic marker in patients undergoing biliary tract cancer resection. *Surg Today*, *55*(11), 1644-1654. https://doi.org/10.1007/s00595-025-03073-3

Shimizu, A., Fukasawa, M., Endo, R., Nakamura, T., Yamada, S., Fujibayashi, R., Kikuchi, K., Tanaka, M., Hasegawa, I., & Sato, H. (2024). Association of Geriatric Nutritional Risk Index With Adverse Event and Treatment Duration in Adjuvant Chemotherapy for Patients With Colorectal Cancer. *In Vivo*, *38*(1), 453-459. https://doi.org/10.21873/invivo.13459

Shimizu, Y., Ashida, R., Sugiura, T., Okamura, Y., Ito, T., Yamamoto, Y., Ohgi, K., Otsuka, S., Notsu, A., & Uesaka, K. (2022). Prognostic Impact of Indicators of Systemic Inflammation and the Nutritional Status of Patients with Resected Carcinoma of the Ampulla of Vater: A Single-Center Retrospective Study. *World J Surg*, *46*(1), 246-258. https://doi.org/10.1007/s00268-021-06346-3

Si, Y., Xu, P., Xu, A., Wang, P., & Zhao, K. (2022). Geriatric nutritional risk index as a prognostic factor in patients with hepatocellular carcinoma following transarterial chemoembolization: A retrospective study. *Medicine (Baltimore)*, *101*(51), e32322. https://doi.org/10.1097/md.0000000000032322

Sim, J. H., Kim, S.-H., Jun, I.-G., Kang, S.-J., Kim, B., Kim, S.-O., & Song, J.-G. (2021). The Association between Prognostic Nutritional Index (PNI) and Intraoperative Transfusion in Patients Undergoing Hepatectomy for Hepatocellular Carcinoma: A Retrospective Cohort Study. *Cancers*, *13*(11), 2508-NA. https://doi.org/10.3390/cancers13112508

Simoes, E., Uchida, R., Nucci, M., Duran, F., Lima, J., Gama, L., Costa, N., Otaduy, M., Bin, F., Otoch, J., Alcantara, P., Ramos, A., Laviano, A., Diaz, M., Esiri, M., DeLuca, G., Herzig, S., Filho, G., & Seelaender, M. (2025). Cachexia Alters Central Nervous System Morphology and Functionality in Cancer Patients [Article]. *Journal of Cachexia, Sarcopenia and Muscle*, *16*(1). https://doi.org/10.1002/jcsm.13742

Soares, J. D. P., Siqueira, J. M., Oliveira, I. C. L., Laviano, A., & Pimentel, G. D. (2020). A high-protein diet, not isolated BCAA, is associated with skeletal muscle mass index in patients with gastrointestinal cancer. *Nutrition*, *72*, 110698. https://doi.org/10.1016/j.nut.2019.110698

Song, B., Zhang, D., Wang, S., Zheng, H., & Wang, X. (2009). Association of interleukin-8 with cachexia from patients with low-third gastric cancer. *Comp Funct Genomics*, *2009*, 212345. https://doi.org/10.1155/2009/212345

Song, H., Sun, H., Yang, L., Gao, H., Cui, Y., Yu, C., Xu, H., & Li, L. (2022). Nutritional Risk Index as a Prognostic Factor Predicts the Clinical Outcomes in Patients With Stage III Gastric Cancer. *Front Oncol*, *12*, 880419. https://doi.org/10.3389/fonc.2022.880419

Song, H. N., Wang, W. B., Luo, X., Huang, D. D., Ruan, X. J., Xing, C. G., Chen, W. Z., Dong, Q. T., & Chen, X. L. (2022). Effect of GLIM-defined malnutrition on postoperative clinical outcomes in patients with colorectal cancer. *Jpn J Clin Oncol*, *52*(5), 466-474. https://doi.org/10.1093/jjco/hyab215

Sonoi, M., Shirakawa, Y., Sonoi, N., Noma, K., Tanabe, S., Maeda, N., & Morimatsu, H. (2025). Preoperative Nutritional Status Influences Enteral Nutrition Weaning 6 Months Post-Surgery in Patients with Esophageal Cancer. *Asian Pac J Cancer Prev*, *26*(1), 263-267. https://doi.org/10.31557/apjcp.2025.26.1.263

Soria-Utrilla, V., Sánchez-Torralvo, F. J., Palmas-Candia, F. X., Fernández-Jiménez, R., Mucarzel-Suarez-Arana, F., Guirado-Peláez, P., Olveira, G., García-Almeida, J. M., & Burgos-Peláez, R. (2024). AI-Assisted Body Composition Assessment Using CT Imaging in Colorectal Cancer Patients: Predictive Capacity for Sarcopenia and Malnutrition Diagnosis. *Nutrients*, *16*(12). https://doi.org/10.3390/nu16121869

Souza, B. U., Souza, N. C. S., Martucci, R. B., Rodrigues, V. D., Pinho, N. B., Gonzalez, M. C., & Avesani, C. M. (2018). Factors Associated with Sarcopenia in Patients with Colorectal Cancer. *Nutr Cancer*, *70*(2), 176-183. https://doi.org/10.1080/01635581.2018.1412480

Souza, N. C., Avesani, C. M., Prado, C. M., Martucci, R. B., Rodrigues, V. D., de Pinho, N. B., Heymsfield, S. B., & Gonzalez, M. C. (2021). Phase angle as a marker for muscle abnormalities and function in patients with colorectal cancer. *Clin Nutr*, *40*(7), 4799-4806. https://doi.org/10.1016/j.clnu.2021.06.013

Souza, N. C., Gonzalez, M. C., Martucci, R. B., Rodrigues, V. D., de Pinho, N. B., Qureshi, A. R., & Avesani, C. M. (2020). Comparative Analysis Between Computed Tomography and Surrogate Methods to Detect Low Muscle Mass Among Colorectal Cancer Patients. *JPEN J Parenter Enteral Nutr*, *44*(7), 1328-1337. https://doi.org/10.1002/jpen.1741

Stephens, N. A., Skipworth, R. J. E., Gallagher, I. J., Greig, C. A., Guttridge, D. C., Ross, J. A., & Fearon, K. C. H. (2015). Evaluating potential biomarkers of cachexia and survival in skeletal muscle of upper gastrointestinal cancer patients [Article]. *Journal of Cachexia, Sarcopenia and Muscle*, 53-61. https://doi.org/10.1002/jcsm.12005

Sugawara, K., Aikou, S., Yajima, S., Uemura, Y., Okumura, Y., Nishida, M., Yagi, K., Yamashita, H., & Seto, Y. (2020). Pre- and post-operative low prognostic nutritional index influences survival in older patients with gastric carcinoma. *J Geriatr Oncol*, *11*(6), 989-996. https://doi.org/10.1016/j.jgo.2020.02.007

Sugawara, K., Yamashita, H., Urabe, M., Okumura, Y., Yagi, K., Aikou, S., & Seto, Y. (2021). Geriatric Nutrition Index Influences Survival Outcomes in Gastric Carcinoma Patients Undergoing Radical Surgery. *JPEN J Parenter Enteral Nutr*, *45*(5), 1042-1051. https://doi.org/10.1002/jpen.1978

Sun, C. Y., Zhang, X. J., Li, Z., Fei, H., Li, Z. F., & Zhao, D. B. (2024). Preoperative prognostic nutritional index predicts long-term outcomes of patients with ampullary adenocarcinoma after curative pancreatoduodenectomy. *World J Gastrointest Surg*, *16*(5), 1291-1300. https://doi.org/10.4240/wjgs.v16.i5.1291

Sun, D., Yin, H., Liu, X., Ding, Z., Shen, L., Sah, S., Han, J., & Wu, G. (2024). Elevated 18F-FDG uptake in subcutaneous adipose tissue correlates negatively with nutritional status and prognostic survival in cachexia patients with gastric cancer. *Clin Nutr*, *43*(2), 567-574. https://doi.org/10.1016/j.clnu.2024.01.006

Sun, F., Sun, Y., Yu, Z., Zhang, D., Zhang, J., Song, B., & Zheng, H. (2010). Interleukin-10 gene polymorphisms influence susceptibility to cachexia in patients with low-third gastric cancer in a Chinese population. *Mol Diagn Ther*, *14*(2), 95-100. https://doi.org/10.1007/bf03256358

Sun, F., Sun, Y., Zhang, D., Zhang, J., Song, B., & Zheng, H. (2010). Association of interleukin-10 gene polymorphism with cachexia in Chinese patients with gastric cancer [Article]. *Annals of Clinical and Laboratory Science*, *40*(2), 149-155.

Sun, F., Zhang, C., Liu, Z., Ai, S., Guan, W., & Liu, S. (2021). Controlling Nutritional Status (CONUT) score as a predictive marker for short-term complications following gastrectomy of gastric cancer: a retrospective study. *BMC Gastroenterol*, *21*(1), 107. https://doi.org/10.1186/s12876-021-01682-z

Sun, H., Chen, L., Huang, R., Pan, H., Zuo, Y., Zhao, R., Xue, Y., & Song, H. (2022). Prognostic nutritional index for predicting the clinical outcomes of patients with gastric cancer who received immune checkpoint inhibitors. *Front Nutr*, *9*, 1038118. https://doi.org/10.3389/fnut.2022.1038118

Sun, J., Wang, D., Mei, Y., Jin, H., Zhu, K., Liu, X., Zhang, Q., & Yu, J. (2017). Value of the prognostic nutritional index in advanced gastric cancer treated with preoperative chemotherapy. *J Surg Res*, *209*, 37-44. https://doi.org/10.1016/j.jss.2016.09.050

Sun, K. Y., Xu, J. B., Chen, S. L., Yuan, Y. J., Wu, H., Peng, J. J., Chen, C. Q., Guo, P., Hao, Y. T., & He, Y. L. (2015). Novel immunological and nutritional-based prognostic index for gastric cancer. *World J Gastroenterol*, *21*(19), 5961-5971. https://doi.org/10.3748/wjg.v21.i19.5961

Sun, L., Su, S., Xiong, J., Hu, W., Liu, L., Xu, H., Du, S., Zhao, H., Lu, X., Sang, X., Zhong, S., Yang, H., & Mao, Y. (2021). Controlling nutritional status score as a prognostic marker to predict overall survival in resected biliary tract cancers. *Ann Transl Med*, *9*(8), 644. https://doi.org/10.21037/atm-20-6770

Sun, S., Li, W., Guo, X., & Chen, J. (2024). Prognostic value of the neutrophil-to-lymphocyte ratio and prognostic nutritional index in unresectable hepatocellular carcinoma patients treated with tyrosine kinase inhibitors and immune checkpoint inhibitors. *Hum Vaccin Immunother*, *20*(1), 2394268. https://doi.org/10.1080/21645515.2024.2394268

Sun, Y., Wu, X., Yu, X., Liu, J., Zhou, H., Wu, H., Cui, X., Geng, S., & Li, Z. (2025). The investigation of factors influencing the prognostic nutritional index in patients with esophageal cancer-a cross-sectional study. *J Thorac Dis*, *17*(7), 5065-5077. https://doi.org/10.21037/jtd-2025-120

Sun, Y., Zhang, B., Han, Y., Jiang, Y., Zhuang, Q., Gong, Y., & Wu, G. (2014). Survey of cachexia in digestive system cancer patients and its impact on clinical outcomes [Article]. *Zhonghua wei chang wai ke za zhi = Chinese journal of gastrointestinal surgery*, *17*(10), 968-971.

Suzuki, H., Mitsunaga, S., Ikeda, M., Aoyama, T., Yoshizawa, K., Yoshimatsu, H., Kawai, N., Masuda, M., Miura, T., & Ochiai, A. (2021). Clinical and tumor characteristics of patients with high serum levels of growth differentiation factor 15 in advanced pancreatic cancer [Article]. *Cancers*, *13*(19). https://doi.org/10.3390/cancers13194842

Suzuki, S., Kanaji, S., Yamamoto, M., Oshikiri, T., Nakamura, T., & Kakeji, Y. (2019). Controlling Nutritional Status (CONUT) Score Predicts Outcomes of Curative Resection for Gastric Cancer in the Elderly [Article]. *World journal of surgery*, *43*(4), 1076-1084. https://doi.org/10.1007/s00268-018-04889-6

Szefel, J., Kruszewski, W. J., Szajewski, M., Ciesielski, M., & Danielak, A. (2020). Bioelectrical Impedance

Analysis to Increase the Sensitivity of Screening Methods for Diagnosing Cancer Cachexia in Patients with Colorectal Cancer [Article]. *Journal of Nutrition and Metabolism*, *2020*. https://doi.org/10.1155/2020/3874956

Takagi, K., Yagi, T., Umeda, Y., Shinoura, S., Yoshida, R., Nobuoka, D., Kuise, T., Araki, H., & Fujiwara, T. (2017). Preoperative Controlling Nutritional Status (CONUT) Score for Assessment of Prognosis Following Hepatectomy for Hepatocellular Carcinoma [Article]. *World journal of surgery*, *41*(9), 2353-2360. https://doi.org/10.1007/s00268-017-3985-8

Takahashi, T., Kaneoka, Y., Maeda, A., Takayama, Y., Aoyama, H., Hosoi, T., & Seita, K. (2024). Low prognostic nutrition index as a prognostic biomarker in elderly patients with early gastric cancer after gastrectomy. *J Med Invest*, *71*(1.2), 113-120. https://doi.org/10.2152/jmi.71.113

Takahashi, T., Kaneoka, Y., Maeda, A., Takayama, Y., Fukami, Y., & Uji, M. (2020). The preoperative prognostic nutrition index is a prognostic indicator for survival in elderly gastric cancer patients after gastrectomy: a propensity score-matched analysis [Article]. *Updates in surgery*, *72*(2), 483-491. https://doi.org/10.1007/s13304-020-00745-2

Takamizawa, Y., Shida, D., Boku, N., Nakamura, Y., Ahiko, Y., Yoshida, T., Tanabe, T., Takashima, A., & Kanemitsu, Y. (2020). Nutritional and inflammatory measures predict survival of patients with stage IV colorectal cancer [Article]. *BMC Cancer*, *20*(1). https://doi.org/10.1186/s12885-020-07560-3

Takano, Y., Kodera, K., Tsukihara, S., Takahashi, S., Yasunobu, K., Kanno, H., Ishiyama, S., Saito, R., Hanyu, N., & Eto, K. (2023). Association of a newly developed Cancer Cachexia Score with survival in Stage I–III colorectal cancer [Article]. *Langenbeck's Archives of Surgery*, *408*(1). https://doi.org/10.1007/s00423-023-02883-8

Takao, K., Konishi, H., Fujiwara, H., Shiozaki, A., Shoda, K., Kosuga, T., Kubota, T., Arita, T., Morimura, R., Murayama, Y., Kuriu, Y., Ikoma, H., Nakanishi, M., Okamoto, K., & Otsuji, E. (2020). Clinical Significance of Prognostic Nutritional Index in the Treatment of Esophageal Squamous Cell Carcinoma. *In Vivo*, *34*(6), 3451-3457. https://doi.org/10.21873/invivo.12184

Takechi, H., Fujikuni, N., Tanabe, K., Hattori, M., Amano, H., Noriyuki, T., & Nakahara, M. (2020). Using the preoperative prognostic nutritional index as a predictive factor for non-cancer-related death in post-curative resection gastric cancer patients: a retrospective cohort study. *BMC Gastroenterol*, *20*(1), 256. https://doi.org/10.1186/s12876-020-01402-z

Takeda, T., Sasaki, T., Suzumori, C., Mie, T., Furukawa, T., Yamada, Y., Kasuga, A., Matsuyama, M., Ozaka, M., & Sasahira, N. (2021). The impact of cachexia and sarcopenia in elderly pancreatic cancer patients receiving palliative chemotherapy. *Int J Clin Oncol*, *26*(7), 1293-1303. https://doi.org/10.1007/s10147-021-01912-0

Talbert, E. E., Lewis, H. L., Farren, M. R., Ramsey, M. L., Chakedis, J. M., Rajasekera, P., Haverick, E., Sarna, A., Bloomston, M., & Pawlik, T. M. (2018). Circulating monocyte chemoattractant protein-1 (MCP-1) is associated with cachexia in treatment-naïve pancreatic cancer patients. *Journal of cachexia, sarcopenia and muscle*, *9*(2), 358-368.

Tamai, M., Kiuchi, J., Kuriu, Y., Arita, T., Shimizu, H., Ohashi, T., Konishi, H., Yamamoto, Y., Morimura, R., Shiozaki, A., Ikoma, H., Kubota, T., Fujiwara, H., Okamoto, K., & Otsuji, E. (2021). Clinical impact of postoperative prognostic nutritional index in colorectal cancer patients undergoing adjuvant chemotherapy. *Am J Cancer Res*, *11*(10), 4947-4955.

Tamai, Y., Iwasa, M., Eguchi, A., Shigefuku, R., Sugimoto, R., Tanaka, H., Kobayashi, Y., Mizuno, S., & Nakagawa, H. (2022). The prognostic role of controlling nutritional status and skeletal muscle mass in patients with hepatocellular carcinoma after curative treatment [Article]. *European Journal of Gastroenterology and Hepatology*, *34*(12), 1269-1276. https://doi.org/10.1097/MEG.0000000000002459

Tambaro, F., Imbimbo, G., Ferraro, E., Andreini, M., Belli, R., Amabile, M. I., Ramaccini, C., Lauteri, G., Nigri, G., Muscaritoli, M., & Molfino, A. (2024). Assessment of lipolysis biomarkers in adipose tissue of patients with gastrointestinal cancer. *Cancer Metab*, *12*(1), 1. https://doi.org/10.1186/s40170-023-00329-9

Tan, S., Jiang, J., Qiu, L., Liang, Y., Meng, J., Tan, N., & Xiang, B. (2024). Prevalence of Malnutrition in Patients with Hepatocellular Carcinoma: A Comparative Study of GLIM Criteria, NRS2002, and PG-SGA, and Identification of Independent Risk Factors. *Nutr Cancer*, *76*(4), 335-344. https://doi.org/10.1080/01635581.2024.2314317

Tan, S., Wang, J., Zhou, F., Tang, M., Xu, J., Zhang, Y., Yan, M., Li, S., Zhang, Z., & Wu, G. (2022). Validation of GLIM malnutrition criteria in cancer patients undergoing major abdominal surgery: A large-scale prospective study [Article]. *Clinical Nutrition*, *41*(3), 599-609. https://doi.org/10.1016/j.clnu.2022.01.010

Tanabe, M., Aoyama, T., Hashimoto, I., Nakayama, Y., Morita, J., Kanematsu, K., Nagasawa, S., Maezawa, Y., Yamada, T., Ogata, T., Saito, A., & Oshima, T. (2025). Clinical Significance of the Geriatric Nutritional Risk Index in the Evaluation of Outcomes of Patients After Radical Gastrectomy. *In Vivo*, *39*(4), 2277-2285. https://doi.org/10.21873/invivo.14023

Tanaka, K., Taoda, A., & Kashiwagi, H. (2021). The associations between nutritional status, physical function and skeletal muscle mass of geriatric patients with colorectal cancer. *Clin Nutr ESPEN*, *41*, 318-324. https://doi.org/10.1016/j.clnesp.2020.11.009

Tanda, H., Shibutani, M., Seki, Y., Nishiyama, T., Kasashima, H., Fukuoka, T., & Maeda, K. (2025). Prognostic Impact of Cachexia in Patients Undergoing Radical Resection for Colorectal Cancer: A Retrospective Study. *J Gastrointest Cancer*, *56*(1), 195. https://doi.org/10.1007/s12029-025-01320-x

Tanemura, A., Mizuno, S., Hayasaki, A., Gyoten, K., Fujii, T., Iizawa, Y., Kato, H., Murata, Y., Kuriyama, N., Kishiwada, M., Sakurai, H., & Isaji, S. (2020). Onodera's prognostic nutritional index is a strong prognostic indicator for patients with hepatocellular carcinoma after initial hepatectomy, especially patients with preserved liver function. *BMC surgery*, *20*(1), 261-261. https://doi.org/10.1186/s12893-020-00917-2

Tang, J., Wong, G., Naffouje, S., Felder, S., Sanchez, J., Dineen, S., Powers, B. D., Dessureault, S., Gurd, E., Castillo, D., & Hodul, P. (2021). A Novel Nomogram for Early Identification and Intervention in Colorectal Cancer Patients at Risk for Malnutrition. *Am Surg*, 31348211058620. https://doi.org/10.1177/00031348211058620

Tang, M., Ge, Y., Zhang, Q., Zhang, X., Xiao, C., Li, Q., Zhang, X., Zhang, K., Song, M., Wang, X., Yang, M., Ruan, G., Mu, Y., Huang, H., Cong, M., Zhou, F., & Shi, H. (2021). Near-term prognostic impact of integrated muscle mass and function in upper gastrointestinal cancer [Article]. *Clinical Nutrition*, *40*(9), 5169-5179. https://doi.org/10.1016/j.clnu.2021.07.028

Tang, M., Liu, S., Li, W., Peng, X., Wang, Y., Chen, Y., Yang, D., Xiang, T., & Wu, Z. (2025). Prognostic value of albumin-to-alkaline phosphatase ratio and CONUT score in rectal cancer patients undergoing XELOX-based chemotherapy: development of a nomogram-based predictive model. *Am J Cancer Res*, *15*(4), 1578-1596. https://doi.org/10.62347/hsde2538

Tanji, Y., Furukawa, K., Haruki, K., Taniai, T., Onda, S., Tsunematsu, M., Shirai, Y., Yanagaki, M., Igarashi, Y., & Ikegami, T. (2022). Significant impact of cachexia index on the outcomes after hepatic resection for colorectal liver metastases. *Annals of gastroenterological surgery*, *6*(6), 804-812. https://doi.org/10.1002/ags3.12578

Tao, Z., Chen, Z., Gao, Y., & Quan, M. (2024). Influence of cachexia on immunotherapy efficacy and prognosis for malignant tumors of the digestive system. *Cancer Rep (Hoboken)*, *7*(5), e2100. https://doi.org/10.1002/cnr2.2100

Tekin, S., Tatli, A. M., Sezgin Göksu, S., & Coşkun, H. Ş. (2023). Prognostic Nutritional Index Predicts Overall Survival Better than Geriatric Nutritional Risk Index in Patients with Metastatic Gastric Cancer [Article]. *Journal of Oncological Science*, *9*(2), 79-88. https://doi.org/10.37047/jos.2023-96450

Teraishi, F., Utsumi, M., Yoshida, Y., Shoji, R., Kanaya, N., Matsumi, Y., Shigeyasu, K., Kondo, Y., Itagaki, S., Tamura, R., Matsuoka, Y., Fujiwara, T., & Inagaki, M. (2025). The Geriatric Nutritional Risk Index: A Key Indicator of Perioperative Outcome in Oldest-old Patients With Colorectal Cancer. *In Vivo*, *39*(5), 2810-2817. https://doi.org/10.21873/invivo.14080

Teraishi, F., Yoshida, Y., Shoji, R., Kanaya, N., Matsumi, Y., Shigeyasu, K., Kondo, Y., Kagawa, S., Tamura, R., Matsuoka, Y., Morimatsu, H., Mitsuhashi, T., & Fujiwara, T. (2024). Subjective global assessment for nutritional screening and its impact on surgical outcomes: A prospective study in older patients with colorectal cancer. *Langenbecks Arch Surg*, *409*(1), 356. https://doi.org/10.1007/s00423-024-03548-w

Terasaki, F., Sugiura, T., Okamura, Y., Ito, T., Yamamoto, Y., Ashida, R., Ohgi, K., & Uesaka, K. (2021). The preoperative controlling nutritional status (CONUT) score is an independent prognostic marker for pancreatic ductal adenocarcinoma. *Updates Surg*, *73*(1), 251-259. https://doi.org/10.1007/s13304-020-00792-9

Terasaki, F., Sugiura, T., Okamura, Y., Ito, T., Yamamoto, Y., Ashida, R., Ohgi, K., & Uesaka, K. (2021). Use of preoperative controlling nutritional status (CONUT) score as a better prognostic marker for distal cholangiocarcinoma after pancreatoduodenectomy. *Surg Today*, *51*(3), 358-365. https://doi.org/10.1007/s00595-020-02098-0

Tırnova, İ., & Karaca, A. S. (2025). Preoperative CONUT score predicts postoperative complications in stage I-III gastric cancer patients undergoing curative gastric resections. *Turk J Surg*, *41*(3), 261-269. https://doi.org/10.47717/turkjsurg.2025.2025-7-36

Tohme, S., Chidi, A. P., Sud, V., & Tsung, A. (2017). Prognostic Nutritional Index Is Associated with Survival in Patients with Unresectable Hepatocellular Carcinoma Treated with Radioembolization. *J Vasc Interv Radiol*, *28*(3), 470-472. https://doi.org/10.1016/j.jvir.2016.10.016

Tokunaga, R., Sakamoto, Y., Nakagawa, S., Miyamoto, Y., Yoshida, N., Oki, E., Watanabe, M., & Baba, H. (2015). Prognostic Nutritional Index Predicts Severe Complications, Recurrence, and Poor Prognosis in Patients With Colorectal Cancer Undergoing Primary Tumor Resection. *Dis Colon Rectum*, *58*(11), 1048-1057. https://doi.org/10.1097/dcr.0000000000000458

Tokunaga, R., Sakamoto, Y., Nakagawa, S., Ohuchi, M., Izumi, D., Kosumi, K., Taki, K., Higashi, T., Miyamoto, Y., Yoshida, N., Oki, E., Watanabe, M., & Baba, H. (2017). CONUT: a novel independent predictive score for colorectal cancer patients undergoing potentially curative resection. *Int J Colorectal Dis*, *32*(1), 99-106. https://doi.org/10.1007/s00384-016-2668-5

Tominaga, T., Nagasaki, T., Akiyoshi, T., Fukunaga, Y., Honma, S., Nagaoka, T., Matsui, S., Minami, H., Miyanari, S., Yamaguchi, T., & Ueno, M. (2020). Prognostic nutritional index and postoperative outcomes in patients with colon cancer after laparoscopic surgery. *Surg Today*, *50*(12), 1633-1643. https://doi.org/10.1007/s00595-020-02050-2

Tominaga, T., Nonaka, T., Hisanaga, M., Fukuda, A., Tanoue, Y., Yoshimoto, T., Hidaka, S., Sawai, T., & Nagayasu, T. (2020). Prognostic value of the preoperative prognostic nutritional index in oldest-old patients with colorectal cancer. *Surg Today*, *50*(5), 449-459. https://doi.org/10.1007/s00595-019-01910-w

Topçu, A., & Yasin, A. İ. (2022). Impact of Malnutrition on Prognosis in Patients with HER2-negative Metastatic Gastric Cancer [Article]. *Medical Journal of Bakirkoy*, *18*(2), 258-265. https://doi.org/10.4274/BMJ.galenos.2022.2022.4-7

Toyokawa, T., Kubo, N., Tamura, T., Sakurai, K., Amano, R., Tanaka, H., Muguruma, K., Yashiro, M., Hirakawa, K., & Ohira, M. (2016). The pretreatment Controlling Nutritional Status (CONUT) score is an independent prognostic factor in patients with resectable thoracic esophageal squamous cell carcinoma: results from a retrospective study. *BMC Cancer*, *16*(1), 722. https://doi.org/10.1186/s12885-016-2696-0

Trestini, I., Paiella, S., Sandini, M., Sperduti, I., Elio, G., Pollini, T., Melisi, D., Auriemma, A., Soldà, C., Bonaiuto, C., Tregnago, D., Avancini, A., Secchettin, E., Bonamini, D., Lanza, M., Pilotto, S., Malleo, G., Salvia, R., Bovo, C., Gianotti, L., Bassi, C., & Milella, M. (2020). Prognostic Impact of Preoperative Nutritional Risk in Patients Who Undergo Surgery for Pancreatic Adenocarcinoma. *Ann Surg Oncol*, *27*(13), 5325-5334. https://doi.org/10.1245/s10434-020-08515-5

Tsukagoshi, M., Araki, K., Igarashi, T., Ishii, N., Kawai, S., Hagiwara, K., Hoshino, K., Seki, T., Okuyama, T.,

Fukushima, R., Harimoto, N., & Shirabe, K. (2024). Lower Geriatric Nutritional Risk Index and Prognostic Nutritional Index Predict Postoperative Prognosis in Patients with Hepatocellular Carcinoma. *Nutrients*, *16*(7). https://doi.org/10.3390/nu16070940

Tsunematsu, M., Haruki, K., Fujiwara, Y., Furukawa, K., Onda, S., Matsumoto, M., Gocho, T., Shiba, H., & Yanaga, K. (2021). Preoperative controlling nutritional status (CONUT) score predicts long-term outcomes in patients with non-B non-C hepatocellular carcinoma after curative hepatic resection. *Langenbecks Arch Surg*, *406*(1), 99-107. https://doi.org/10.1007/s00423-020-01987-9

Tu, M. Y., Chien, T. W., & Chou, M. T. (2012). Using a nutritional screening tool to evaluate the nutritional status of patients with colorectal cancer. *Nutr Cancer*, *64*(2), 323-330. https://doi.org/10.1080/01635581.2012.650778

Tustumi, F., Pereira, M. A., Lisak, A. S., Ramos, M., Ribeiro Junior, U., & Dias, A. R. (2024). THE VALUE OF PREOPERATIVE PROGNOSTIC NUTRITIONAL INDEX IN GASTRIC CANCER AFTER CURATIVE RESECTION. *Arq Bras Cir Dig*, *37*, e1805. https://doi.org/10.1590/0102-6720202400012e1805

Ucar, G., Ergun, Y., Acikgoz, Y., & Uncu, D. (2020). The prognostic value of the prognostic nutritional index in patients with metastatic colorectal cancer. *Asia Pac J Clin Oncol*, *16*(5), e179-e184. https://doi.org/10.1111/ajco.13328

Uemura, S., Iwashita, T., Ichikawa, H., Iwasa, Y., Mita, N., Shiraki, M., & Shimizu, M. (2022). Impact of Controlling nutritional status (CONUT) in patients with unresectable advanced pancreatic cancer receiving multi-agent chemotherapy: A single center, retrospective cohort study [Article]. *Pancreatology*, *22*(2), 304-310. https://doi.org/10.1016/j.pan.2022.01.010

Umino, R., Kobayashi, Y., Akabane, M., Kojima, K., Okubo, S., Hashimoto, M., & Shindoh, J. (2022). Preoperative nutritional score predicts underlying liver status and surgical risk of hepatocellular carcinoma. *Scand J Surg*, *111*(1), 14574969211061953. https://doi.org/10.1177/14574969211061953

Unome, S., Imai, K., Aiba, M., Miwa, T., Hanai, T., Suetsugu, A., Takai, K., & Shimizu, M. (2025). Cachexia is an independent predictor of mortality in patients with hepatocellular carcinoma on systemic targeted therapy. *Clin Nutr ESPEN*, *66*, 454-459. https://doi.org/10.1016/j.clnesp.2025.02.018

Urabe, M., Ueno, M., Ogawa, Y., Yago, A., Shimoyama, H., Honda, A., Ohkura, Y., Haruta, S., & Udagawa, H. (2021). Comparative analysis of the prognostic utility of preoperative nutritional parameters in patients with resectable esophageal carcinoma. *Gen Thorac Cardiovasc Surg*, *69*(2), 326-335. https://doi.org/10.1007/s11748-020-01555-4

van der Kroft, G., Bours, D., Janssen-Heijnen, D. M., van Berlo, D., & Konsten, D. (2018). Value of sarcopenia assessed by computed tomography for the prediction of postoperative morbidity following oncological colorectal resection: A comparison with the malnutrition screening tool. *Clin Nutr ESPEN*, *24*, 114-119. https://doi.org/10.1016/j.clnesp.2018.01.003

van der Werf, A., van Bokhorst, Q. N. E., de van der Schueren, M. A. E., Verheul, H. M. W., & Langius, J. A. E. (2018). Cancer Cachexia: identification by Clinical Assessment versus International Consensus Criteria in Patients with Metastatic Colorectal Cancer [Journal article; Multimedia]. *Nutrition and cancer*, *70*(8), 1322-1329. https://doi.org/10.1080/01635581.2018.1504092

Vashi, P., DaSilva, T., Nader, A., & Gupta, D. (2017). Prevalence of sarcopenia assessed using CT imaging and its correlation with subjective global assessment in advanced gastrointestinal cancer [Conference Abstract]. *Journal of Parenteral and Enteral Nutrition*, *41*(2), 276. https://doi.org/10.1177/0148607116686023

Vashi, P. G., Gorsuch, K., Wan, L., Hill, D., Block, C., & Gupta, D. (2019). Sarcopenia supersedes subjective global assessment as a predictor of survival in colorectal cancer [Article]. *PLoS ONE*, *14*(6). https://doi.org/10.1371/journal.pone.0218761

Velasco, R. N., Catedral, L. I. G., Chua, A. V., Hernandez, A. R. B., King, R. E. C., Leones, L. M. B., Mondragon,

K. A. M., Ting, F. I. L., Callueng, J. M. C., Tampo, M. M. T., & Sacdalan, D. L. (2022). The Impact of Malnutrition on the Quality of Life of Colorectal Cancer Patients in a Tertiary Hospital [Article]. *Nutrition and Cancer*, *74*(8), 2937-2945. https://doi.org/10.1080/01635581.2022.2044061

Vieira Maroun, E., Argente Pla, M., Pedraza Serrano, M. J., Muresan, B. T., Ramos Prol, A., Gascó Santana, E., Martín Sanchis, S., Durá De Miguel, Á., Micó García, A., Cebrián Vázquez, A., Durbá Lacruz, A., & Merino-Torres, J. F. (2024). Phase Angle and Ultrasound Assessment of the Rectus Femoris for Predicting Malnutrition and Sarcopenia in Patients with Esophagogastric Cancer: A Cross-Sectional Pilot Study. *Nutrients*, *17*(1). https://doi.org/10.3390/nu17010091 10.3390/nu17010091.

Wan, Q., Yuan, Q., Zhao, R., Shen, X., Chen, Y., Li, T., & Song, Y. (2022). Prognostic value of cachexia index in patients with colorectal cancer: A retrospective study [Article]. *Frontiers in Oncology*, *12*. https://doi.org/10.3389/fonc.2022.984459

Wang, A., He, Z., Cong, P., Qu, Y., Hu, T., Cai, Y., Sun, B., Chen, H., Fu, W., & Peng, Y. (2021). Controlling Nutritional Status (CONUT) Score as a New Indicator of Prognosis in Patients With Hilar Cholangiocarcinoma Is Superior to NLR and PNI: A Single-Center Retrospective Study. *Frontiers in oncology*, *10*, 593452-NA. https://doi.org/10.3389/fonc.2020.593452

Wang, C., Lu, M., Zhou, T., Zhao, S., & Guan, S. (2019). Intensity-modulated radiotherapy does not decrease the risk of malnutrition in esophageal cancer patients during radiotherapy compared to three-dimensional conformal radiation therapy [Article]. *Journal of Thoracic Disease*, *11*(9), 3721-3731. https://doi.org/10.21037/jtd.2019.09.33

Wang, H. X., Wang, C. C., Yang, W., Gao, L. L., & Yu, S. Q. (2018). Prognostic value of preoperative prognostic nutritional index in stage III gastric cancer after curative resection: a retrospective cohort study [Article]. *Asia Pacific journal of clinical nutrition*, *27*(3), 540-545. https://doi.org/10.6133/apjcn.072017.03

Wang, J., Bo, X., Li, M., Nan, L., Wang, C., Gao, Z., Suo, T., Ni, X., Liu, H., Han, J., Lu, P., Liu, H., & Wang, Y. (2021). Prediction Efficacy for Clinical Outcome of Prognostic Nutritional Index in Patients with Resectable Biliary Tract Cancer Depends on Sex and Obstructive Jaundice Status [Article]. *Annals of Surgical Oncology*, *28*(1), 430-438. https://doi.org/10.1245/s10434-020-08728-8

Wang, J., Yu, B., Ye, Y., Shen, J., Ding, N., Tang, H., Xu, Y., Song, L., Zhu, Z., Chen, Y., Xie, S., & Chen, M. (2018). Predictive Value of Nutritional Risk Screening 2002 and Prognostic Nutritional Index for Esophageal Cancer Patients Undergoing Definitive Radiochemotherapy [Article]. *Nutrition and Cancer*, *70*(6), 879-885. https://doi.org/10.1080/01635581.2018.1470656

Wang, J., Zhuang, Q., Tan, S., Xu, J., Zhang, Y., Yan, M., Li, S., Zhang, Z., & Wu, G. (2023). Loss of body weight and skeletal muscle negatively affect postoperative outcomes after major abdominal surgery in geriatric patients with cancer [Article]. *Nutrition*, *106*. https://doi.org/10.1016/j.nut.2022.111907

Wang, L., Miao, Y., Chen, T., Sun, D., Ge, S., Zuo, L., & Liu, M. (2020). Value of the preoperative prognostic nutritional index for the evaluation of patient prognosis after radical gastrectomy. *Mol Clin Oncol*, *12*(3), 196-201. https://doi.org/10.3892/mco.2020.1980

Wang, L. J., Lei, C. L., Wang, T. A., Lin, Z. F., Feng, S. J., Wei, T., Li, Y. Q., Shen, M. R., Li, Y., & Liao, L. F. (2025). Prognostic value of the preoperative systemic immune-inflammation nutritional index in patients with gastric cancer. *World J Clin Oncol*, *16*(4), 102294. https://doi.org/10.5306/wjco.v16.i4.102294

Wang, P., Chen, X., Liu, Q., Liu, X., & Li, Y. (2021). Good performance of the Global Leadership Initiative on Malnutrition criteria for diagnosing and classifying malnutrition in people with esophageal cancer undergoing esophagectomy [Article]. *Nutrition*, *91-92*. https://doi.org/10.1016/j.nut.2021.111420

Wang, P. Y., Chen, X. K., Liu, Q., Xu, L., Zhang, R. X., Liu, X. B., & Li, Y. (2021). Application of four nutritional risk indexes in perioperative management for esophageal cancer patients [Article]. *Journal of Cancer Research and Clinical Oncology*, *147*(10), 3099-3111. https://doi.org/10.1007/s00432-021-03585-8

Wang, S., Cheng, L., Dou, L., Kuang, Y., Huang, Y., Wen, T., Xiang, L., Xie, W., Zhang, C., Li, D., & Li, H. (2025). Geriatric nutritional risk index and body composition dictate the prognosis of elderly patients with intrahepatic cholangiocarcinoma. *Front Nutr*, *12*, 1565317. https://doi.org/10.3389/fnut.2025.1565317

Wang, W. J., Li, T. T., Wang, X., Li, W., & Cui, J. W. (2020). Combining the Patient-Generated Subjective Global Assessment (PG-SGA) and Objective Nutrition Assessment Parameters Better Predicts Malnutrition in Elderly Patients with Colorectal Cancer. *Journal of Nutritional Oncology*, *5*(1).

Wang, X., Cheng, G., Tao, R., Qu, Z., Tang, W., Deng, Y., & Liu, W. (2020). Clinical characteristics and predictors of permanent stoma in rectal cancer patients underwent anterior resections: the value of preoperative prognostic nutritional index [Article]. *International Journal of Clinical Oncology*, *25*(11), 1960-1968. https://doi.org/10.1007/s10147-020-01743-5

Wang, X., Liu, X., Dai, H., & Jia, J. (2023). Peripheral blood nutrient indices as biomarkers for anti-PD-1 therapy efficacy and prognosis in patients with advanced gastric cancer. *Oncol Lett*, *26*(3), 397. https://doi.org/10.3892/ol.2023.13983

Wang, X., Tang, X., Xu, J., Zhang, R., Chu, J., Chen, C., & Wei, C. (2024). Investigating the clinical predictive utility of inflammatory markers and nomogram development in colorectal cancer patients with malnutrition. *Front Nutr*, *11*, 1442094. https://doi.org/10.3389/fnut.2024.1442094

Wang, X., Wu, J., Lei, S., Tian, F., Cao, C., & Shi, G. (2020). Effect of Preoperative Nutritional Risk Screening on Postoperative Recovery in Patients with Laparoscopic-Assisted Radical Resection for Colorectal Cancer. *Gastroenterol Res Pract*, *2020*, 2046253. https://doi.org/10.1155/2020/2046253

Wang, X. B., Chen, J., Xiang, B. D., Wu, F. X., & Li, L. Q. (2019). High CONUT score predicts poor survival and postoperative HBV reactivation in HBV-related hepatocellular carcinoma patients with low HBV-DNA levels. *Eur J Surg Oncol*, *45*(5), 782-787. https://doi.org/10.1016/j.ejso.2018.11.007

Wang, Y., Wang, L., Fang, M., Li, J., Song, T., Zhan, W., & Xu, H. (2020). Prognostic Value of the Geriatric Nutritional Risk Index in Patients Exceeding 70 Years Old with Esophageal Squamous Cell Carcinoma. *Nutr Cancer*, *72*(4), 620-626. https://doi.org/10.1080/01635581.2019.1650189

Wang, Y., Wang, Y., Li, G., Zhang, H., Yu, H., Xiang, J., Wang, Z., Jiang, X., Yan, G., Liu, Y., Wang, C., Xiong, H., Wang, G., Shi, H., & Liu, M. (2022). Associations of intermuscular adipose tissue and total muscle wasting score in PG-SGA with low muscle radiodensity and mass in nonmetastatic colorectal cancer: A two-center cohort study. *Front Nutr*, *9*, 967902. https://doi.org/10.3389/fnut.2022.967902

Wang, Y., Zheng, J., Gao, Z., Han, X., & Qiu, F. (2018). Investigation on nutritional risk assessment and nutritional support status of surgical patients with colorectal cancer [Article]. *Journal of B.U.ON.*, *23*(1), 62-67.

Watanabe, H., Yamada, T., Komori, K., Hara, K., Kano, K., Takahashi, K., Kumazu, Y., Fujikawa, H., Numata, M., Aoyama, T., Tamagawa, H., Inokuchi, Y., Machida, N., Shiozawa, M., Yukawa, N., Morinaga, S., Rino, Y., Masuda, M., Ogata, T., & Oshima, T. (2021). Effect of Prognostic Nutrition Index in Gastric or Gastro-oesophageal Junction Cancer Patients Undergoing Nivolumab Monotherapy. *In Vivo*, *35*(1), 563-569. https://doi.org/10.21873/invivo.12292

Watanabe, M., Iwatsuki, M., Iwagami, S., Ishimoto, T., Baba, Y., & Baba, H. (2012). Prognostic nutritional index predicts outcomes of gastrectomy in the elderly. *World J Surg*, *36*(7), 1632-1639. https://doi.org/10.1007/s00268-012-1526-z

Wijma, A. G., Hogenbirk, R. N. M., Driessens, H., Kluifhooft, D. A., Jellema-Betten, E. S., Tjalsma-de Vries, M., Liem, M. S. L., Nieuwenhuijs, V. B., Manusama, E. M., Hoogwater, F. J. H., Nijkamp, M. W., Beijer, S., & Klaase, J. M. (2024). Nutritional support in pancreatic cancer patients and its effect on nutritional status: an observational regional HPB network study investigating current practice. *Support Care Cancer*, *32*(7), 487. https://doi.org/10.1007/s00520-024-08683-0

Wobith, M., Herbst, C., Lurz, M., Haberzettl, D., Fischer, M., & Weimann, A. (2022). Evaluation of

malnutrition in patients undergoing major abdominal surgery using GLIM criteria and comparing CT and BIA for muscle mass measurement [Article]. *Clinical Nutrition ESPEN*, *50*, 148-154. https://doi.org/10.1016/j.clnesp.2022.06.004

Wolf, J. H., Ahuja, V., D'Adamo, C. R., Coleman, J., Katlic, M., & Blumberg, D. (2020). Preoperative Nutritional Status Predicts Major Morbidity After Primary Rectal Cancer Resection. *J Surg Res*, *255*, 325-331. https://doi.org/10.1016/j.jss.2020.05.081

Wong, G., Tang, J., Standlick, A., Russo, N., Rennie, A., Gurd, E., Powers, B., & Hodul, P. (2021). Colorectal cancer stage is associated with increased risk of malnutrition [Conference Abstract]. *Journal of Parenteral and Enteral Nutrition*, *45*(SUPPL 1), S131-S132. https://doi.org/10.1002/jpen.2095

Wu, Q., Zeng, J., & Zeng, J. (2025). Prognostic nutritional index is a better inflammation based prognostic marker in elderly patients with hepatocellular carcinoma after surgery. *Sci Rep*, *15*(1), 30510. https://doi.org/10.1038/s41598-025-12158-5

Wu, S. J., Lin, Y. X., Ye, H., Li, F. Y., Xiong, X. Z., & Cheng, N. S. (2016). Lymphocyte to monocyte ratio and prognostic nutritional index predict survival outcomes of hepatitis B virus-associated hepatocellular carcinoma patients after curative hepatectomy [Article]. *Journal of Surgical Oncology*, *114*(2), 202-210. https://doi.org/10.1002/jso.24297

Wu, T., Xu, H., Li, W., Zhou, F., Guo, Z., Wang, K., Weng, M., Zhou, C., Liu, M., Lin, Y., Li, S., He, Y., Yao, Q., Shi, H., & Song, C. (2024). The potential of machine learning models to identify malnutrition diagnosed by GLIM combined with NRS-2002 in colorectal cancer patients without weight loss information. *Clin Nutr*, *43*(5), 1151-1161. https://doi.org/10.1016/j.clnu.2024.04.001

Wu, T., Xu, H., Zou, Y., Cui, J., Xu, K., Zhou, M., Guo, P., Cheng, H., Shi, H., Song, C., The Investigation On Nutrition, S., & Its Clinical Outcome Of Common Cancers Inscoc Group, N. A. (2022). Mid-Arm Muscle Circumference or Body Weight-Standardized Hand Grip Strength in the GLIM Superiorly Predicts Survival in Chinese Colorectal Cancer Patients. *Nutrients*, *14*(23), 5166-5166. https://doi.org/10.3390/nu14235166

Xi, X., Yang, M. X., Wang, X. Y., & Shen, D. J. (2022). Predictive value of prognostic nutritional index on infection after radical gastrectomy: a retrospective study [Article]. *Journal of Gastrointestinal Oncology*, *13*(2), 569-580. https://doi.org/10.21037/jgo-22-192

Xia, L. J., Li, W., Zhai, J. C., Yan, C. W., Chen, J. B., & Yang, H. (2020). Significance of neutrophil-to-lymphocyte ratio, platelet-to-lymphocyte ratio, lymphocyte-to-monocyte ratio and prognostic nutritional index for predicting clinical outcomes in T1-2 rectal cancer [Article]. *BMC Cancer*, *20*(1). https://doi.org/10.1186/s12885-020-6698-6

Xiang, S., Yang, Y. X., Pan, W. J., Li, Y., Zhang, J. H., Gao, Y., & Liu, S. (2023). Prognostic value of systemic immune inflammation index and geriatric nutrition risk index in early-onset colorectal cancer. *Front Nutr*, *10*, 1134300. https://doi.org/10.3389/fnut.2023.1134300

Xiao, A. T., Tong, Y. X., Xu, X. S., Zhou, Y., & Zhang, S. (2020). Preoperative Nutritional Status Contributes to the Development of Neutropenia Event in Patients With Gastric Cancer Receiving CAPEOX Adjuvant Chemotherapy [Article]. *Frontiers in Oncology*, *10*. https://doi.org/10.3389/fonc.2020.00692

Xiao, F. K., Wang, L., Zhang, W. C., Wang, L. D., & Zhao, L. S. (2021). Preoperative Prognostic Nutritional Index is a Significant Predictor of Survival in Esophageal Squamous Cell Carcinoma Patients. *Nutr Cancer*, *73*(2), 215-220. https://doi.org/10.1080/01635581.2020.1757129

Xiao, L., Lyu, J., Liu, X., Li, K., Wang, Y., Zhang, R., Chen, T., & Li, T. (2021). Clinical Application Value of the Prognostic Nutritional Index for Predicting Survival in Patients with Esophageal Squamous Cell Carcinoma Undergoing Chemoradiotherapy or Radiotherapy. *Nutr Cancer*, *73*(10), 1933-1940. https://doi.org/10.1080/01635581.2020.1817511

Xiao, Q., Li, X., Duan, B., Li, X., Liu, S., Xu, B., Shi, S., Zhang, J., Qin, H., Duan, X., & Pu, Y. (2022). Clinical

significance of controlling nutritional status score (CONUT) in evaluating outcome of postoperative patients with gastric cancer. *Sci Rep*, *12*(1), 93. https://doi.org/10.1038/s41598-021-04128-4

Xiao, Y., Wei, G., Ma, M., Liu, D., Chen, P., Quan, H., Luo, J., & Xiao, H. (2022). Association among prognostic nutritional index, post-operative infection and prognosis of stage II/III gastric cancer patients following radical gastrectomy. *European journal of clinical nutrition*, *76*(10), 1449-1456. https://doi.org/10.1038/s41430-022-01120-7

Xie, H., Nong, C., Yuan, G., Huang, S., Kuang, J., Yan, L., Ruan, G., Tang, S., & Gan, J. (2020). The value of preoperative controlling nutritional status score in evaluating short-term and long-term outcomes of patients with colorectal cancer following surgical resection. *J Cancer*, *11*(23), 7045-7056. https://doi.org/10.7150/jca.49383

Xie, H., Wei, L., Yuan, G., Liu, M., Liang, Y., Gao, S., Wang, Q., Lin, X., Tang, S., & Gan, J. (2022). Combination of Geriatric Nutritional Risk Index and Carcinoembryonic Antigen to Predict the Survival of Patients With Colorectal Cancer. *Front Nutr*, *9*, 902080. https://doi.org/10.3389/fnut.2022.902080

Xie, H., Wei, L., Yuan, G., Liu, M., Tang, S., & Gan, J. (2022). Prognostic Value of Prognostic Nutritional Index in Patients With Colorectal Cancer Undergoing Surgical Treatment. *Front Nutr*, *9*, 794489. https://doi.org/10.3389/fnut.2022.794489

Xishan, Z., Ye, Z., Feiyan, M., Liang, X., & Shikai, W. (2020). The role of prognostic nutritional index for clinical outcomes of gastric cancer after total gastrectomy. *Sci Rep*, *10*(1), 17373. https://doi.org/10.1038/s41598-020-74525-8

Xu, L. B., Mei, T. T., Cai, Y. Q., Chen, W. J., Zheng, S. X., Wang, L., Chen, X. D., & Huang, Y. S. (2022). Correlation Between Components of Malnutrition Diagnosed by Global Leadership Initiative on Malnutrition Criteria and the Clinical Outcomes in Gastric Cancer Patients: A Propensity Score Matching Analysis. *Front Oncol*, *12*, 851091. https://doi.org/10.3389/fonc.2022.851091

Xu, L. B., Shi, M. M., Huang, Z. X., Zhang, W. T., Zhang, H. H., Shen, X., & Chen, X. D. (2022). Impact of malnutrition diagnosed using Global Leadership Initiative on Malnutrition criteria on clinical outcomes of patients with gastric cancer. *JPEN J Parenter Enteral Nutr*, *46*(2), 385-394. https://doi.org/10.1002/jpen.2127

Xu, S., Xu, M., Sun, X., Deng, J., Wu, H., Xiong, R., & Xie, M. (2018). The correlation of prognostic nutritional index with postoperative complications and prognosis in patients with esophageal cancer [Article]. *Chinese Journal of Clinical Oncology*, *45*(15), 789-794. https://doi.org/10.3969/j.issn.1000-8179.2018.15.451

Xu, S., Zhu, H., & Zheng, Z. (2023). Preoperative Prognostic Nutritional Index Predict Survival in Patients with Resectable Adenocarcinoma of the Gastroesophageal Junction: A Retrospective Study Based on Propensity Score Matching Analyses. *Cancer Manag Res*, *15*, 591-599. https://doi.org/10.2147/cmar.S415618

Xu, X., & Jing, J. (2022). Inflammation-related parameter serve as prognostic biomarker in esophageal squamous cell carcinoma. *Front Oncol*, *12*, 900305. https://doi.org/10.3389/fonc.2022.900305

Xu, Y., Zhang, L., Huang, Q., Yin, Z., & Zhang, W. (2025). Nutritional Risk Index (NRI) predicts the clinical outcomes of patients with gastric cancer who received immune checkpoint inhibitors (PD-1/PD-L1). *Medicine (Baltimore)*, *104*(1), e40898. https://doi.org/10.1097/md.0000000000040898

Xu, Y. S., Liu, G., Zhao, C., Lu, S. L., Long, C. Y., Zhong, H. G., Chen, Y., Huang, L. X., & Liang, Z. (2021). Prognostic Value of Combined Preoperative Carcinoembryonic Antigen and Prognostic Nutritional Index in Patients With Stage II-III Colon Cancer. *Front Surg*, *8*, 667154. https://doi.org/10.3389/fsurg.2021.667154

Xu, Z., Chen, X., Yuan, J., Wang, C., An, J., & Ma, X. (2022). Correlations of preoperative systematic immuno-inflammatory index and prognostic nutrition index with a prognosis of patients after radical gastric cancer surgery. *Surgery*, *172*(1), 150-159. https://doi.org/10.1016/j.surg.2022.01.006

Yagyu, T., Yamamoto, M., Tanio, A., Hara, K., Sugezawa, K., Uejima, C., Kihara, K., Tatebe, S., Kurisu, Y., Shibata, S., Yamamoto, T., Nishie, H., Shiota, S., Saito, H., Naka, T., Sugamura, K., Katano, K., & Fujiwara, Y. (2022). Risk factors for recurrence in elderly patients with stage II colorectal cancer: a multicenter retrospective study.

BMC Cancer, *22*(1), 390. https://doi.org/10.1186/s12885-022-09501-8

Yamamoto, M., Omori, T., Masuike, Y., Shinno, N., Hara, H., Sugase, T., Kanemura, T., Takeno, A., Hirao, M., & Miyata, H. (2025). Postoperative Prognostic Nutritional Index as a Useful Prognostic Factor in Patients With Gastric Cancer [Article in Press]. *Annals of Gastroenterological Surgery*. https://doi.org/10.1002/ags3.70057

Yamamoto, M., Saito, H., Uejima, C., Tanio, A., Tada, Y., Matsunaga, T., Sakamoto, T., Honjo, S., Ashida, K., & Fujiwara, Y. (2019). Prognostic Value of Combined Tumor Marker and Controlling Nutritional Status (CONUT) Score in Colorectal Cancer Patients. *Yonago Acta Med*, *62*(1), 124-130. https://doi.org/10.33160/yam.2019.03.017

Yamana, I., Takeno, S., Shibata, R., Shiwaku, H., Maki, K., Hashimoto, T., Shiraishi, T., Iwasaki, A., & Yamashita, Y. (2015). Is the Geriatric Nutritional Risk Index a Significant Predictor of Postoperative Complications in Patients with Esophageal Cancer Undergoing Esophagectomy? *Eur Surg Res*, *55*(1-2), 35-42. https://doi.org/10.1159/000376610

Yamanaka-Kohno, R., Shirakawa, Y., Inoue-Minakuchi, M., Yokoi, A., Muro, M., Kosaki, H., Tanabe, S., Fujiwara, T., & Morita, M. (2021). Association of dental occlusal support with the Prognostic Nutritional Index in patients with esophageal cancer who underwent esophagectomy. *Esophagus*, *18*(1), 49-55. https://doi.org/10.1007/s10388-020-00751-8

Yan, K., Wei, W., Shen, W., Du, X., Zhu, S., Zhao, H., Wang, X., Yang, J., Zhang, X., & Deng, W. (2022). Combining the systemic inflammation response index and prognostic nutritional index to predict the prognosis of locally advanced elderly esophageal squamous cell carcinoma patients undergoing definitive radiotherapy. *J Gastrointest Oncol*, *13*(1), 13-25. https://doi.org/10.21037/jgo-21-784

Yan, X., Zhu, J., Wang, J., Lu, Y., Ye, X., Sun, X., Jiang, H., Li, Z., He, C., Zhai, W., Dong, Q., Chen, W., Yu, Z., Pan, Y., & Huang, D. (2024). Development and validation of a novel prognostic prediction system based on GLIM-defined malnutrition for colorectal cancer patients post-radical surgery. *Front Nutr*, *11*, 1425317. https://doi.org/10.3389/fnut.2024.1425317

Yang, C. K., Huang, K. T., Qin, W., Wu, Q. Y., Huang, X. L., Peng, K., Lao, Q., Ye, X. P., Zhu, G. Z., Li, T. M., & Peng, T. (2024). Prognostic value of geriatric nutritional risk index and prognostic nutritional index in hepatocellular carcinoma. *Clin Nutr ESPEN*, *59*, 355-364. https://doi.org/10.1016/j.clnesp.2023.12.148

Yang, J. Y., Zhu, J. Y., Cao, P. L., Haq, I. U., Shao, J. H., Huang, S. P., & Wang, S. A. (2024). Applicability of Global Leadership Initiative on Malnutrition (GLIM) criteria in nutrition risk screening for patients over 60 years old with digestive system tumors – A retrospective study [Article]. *Nutrition Clinique et Metabolisme*, *38*(3), 194-202. https://doi.org/10.1016/j.nupar.2024.06.002

Yang, Y., Yang, J., & Xu, L. (2025). Predictive values of body mass index, prognostic nutritional index and C-reactive protein to prealbumin ratio for prognosis of patients receiving radical gastrectomy. *Nutr Hosp*, *42*(2), 275-284. https://doi.org/10.20960/nh.05445 10.20960/nh.05445. (Valores predictivos del índice de masa corporal, el índice pronóstico nutricional y el cociente proteína C-reactiva/prealbúmina para el pronóstico de los pacientes sometidos a gastrectomía radical.)

Yang, Y., Ye, F., Xin, Y., Wang, Y., Li, X., Feng, D., Chen, Y., & Zhou, X. (2020). Prognostic significance of controlling nutritional status score-based nomogram for hepatocellular carcinoma within Milan criteria after radiofrequency ablation [Article]. *Journal of Gastrointestinal Oncology*, *11*(5), 1024-1039. https://doi.org/10.21037/jgo-20-225

Yasui-Yamada, S., Oiwa, Y., Saito, Y., Aotani, N., Matsubara, A., Matsuura, S., Tanimura, M., Tani-Suzuki, Y., Kashihara, H., Nishi, M., Shimada, M., & Hamada, Y. (2020). Impact of phase angle on postoperative prognosis in patients with gastrointestinal and hepatobiliary–pancreatic cancer [Article]. *Nutrition*, *79-80*. https://doi.org/10.1016/j.nut.2020.110891

Ye, X. J., Ji, Y. B., Ma, B. W., Huang, D. D., Chen, W. Z., Pan, Z. Y., Shen, X., Zhuang, C. L., & Yu, Z. (2018). Comparison of three common nutritional screening tools with the new European Society for Clinical Nutrition and

Metabolism (ESPEN) criteria for malnutrition among patients with geriatric gastrointestinal cancer: A prospective study in China [Article]. *BMJ Open*, *8*(4). https://doi.org/10.1136/bmjopen-2017-019750

Yıldırım, İ., Kaya, T., İşsever, K., Genç, A. C., Karacan, A., Önmez, A., & Hacıbekiroğlu, İ. (2021). Psoas muscle mass, nutritional status, inflammation, and their relationship with prognosis in patients with pancreatic adenocarcinoma. *Nutr Hosp*, *38*(5), 1009-1015. https://doi.org/10.20960/nh.03573 10.20960/nh.03573. (Masa del músculo psoas, estado nutricional, inflamación y su relación con el pronóstico en pacientes con adenocarcinoma de páncreas.)

Yıldız Kopuz, T. N., Yildiz, H. F., Er, S., & Fisunoglu, M. (2024). Preoperative nutritional factors as predictors of postoperative early outcomes in colorectal cancer - A prospective cohort study. *Nutr Hosp*, *41*(5), 1032-1043. https://doi.org/10.20960/nh.05331 10.20960/nh.05331. (Factores nutricionales preoperatorios como predictores de resultados postoperatorios precoces en cáncer colorrectal: un estudio prospectivo de cohortes.)

Yin, L., Cheng, N., Chen, P., Zhang, M., Li, N., Lin, X., He, X., Wang, Y., Xu, H., Guo, W., & Liu, J. (2021). Association of Malnutrition, as Defined by the PG-SGA, ESPEN 2015, and GLIM Criteria, With Complications in Esophageal Cancer Patients After Esophagectomy. *Front Nutr*, *8*, 632546. https://doi.org/10.3389/fnut.2021.632546

Yoo, Y. J., Kang, C. M., Choi, M., Rho, S. Y., Hwang, H. K., Lee, W. J., Kim, E. W., & Lee, J. A. (2020). Preoperative prognostic nutritional index as an independent prognostic factor for resected ampulla of Vater cancer. *PLoS One*, *15*(3), e0229597. https://doi.org/10.1371/journal.pone.0229597

Yoshida, N., Harada, K., Baba, Y., Kosumi, K., Iwatsuki, M., Kinoshita, K., Nakamura, K., Sakamoto, Y., Miyamoto, Y., Karashima, R., Mima, K., Sawayama, H., Ohuchi, M., Chikamoto, A., Imamura, Y., Watanabe, M., & Baba, H. (2017). Preoperative controlling nutritional status (CONUT) is useful to estimate the prognosis after esophagectomy for esophageal cancer. *Langenbeck's archives of surgery*, *402*(2), 333-341. https://doi.org/10.1007/s00423-017-1553-1

Yoshimatsu, K., Sagawa, M., Yokomizo, H., Yano, Y., Okayama, S., Satake, M., Sakuma, A., Matsumoto, A., Fujimoto, T., & Asaka, S. (2017). Clinical significance of controlling nutritional status (CONUT) in patients with colorectal cancer. *Japan J Surg Metab Nutri*, *51*, 183-190.

Yu, Y., Wu, H., Qiu, J., Ke, D., Wu, Y., Lin, M., Liu, T., Zheng, Q., Zheng, H., Yang, J., Wang, Z., Li, H., Liu, L., Yao, Q., Li, J., Cheng, W., & Chen, X. (2022). A Nutrition-Related Factor-Based Risk Stratification for Exploring the Clinical Benefits in the Treatment of Patients With Locally Advanced Esophageal Squamous Cell Carcinoma Receiving Definitive Chemoradiotherapy: A Retrospective Cohort Study. *Frontiers in nutrition*, *9*, 896847-NA. https://doi.org/10.3389/fnut.2022.896847

Yun, J. H., Song, G. J., Son, M. W., & Lee, M. S. (2024). Global Leadership Initiative on Malnutrition Criteria and Immunonutritional Status Predict Chemoadherence and Survival in Stage II/III Gastric Cancer Treated with XELOX Chemotherapy. *Nutrients*, *16*(20). https://doi.org/10.3390/nu16203468 10.3390/nu16203468.

Zhang, C., Wang, H., Ning, Z., Xu, L., Zhuang, L., Wang, P., & Meng, Z. (2016). Prognostic nutritional index serves as a predictive marker of survival and associates with systemic inflammatory response in metastatic intrahepatic cholangiocarcinoma. *OncoTargets and therapy*, 6417-6423.

Zhang, C., & Zhao, J. (2021). Analysis of influencing factors of malnutrition in elderly patients with gastrointestinal malignant tumors and the intervention effects of enteral and parenteral nutrition support [Article]. *Anti-Tumor Pharmacy*, *11*(6), 769-774. https://doi.org/10.3969/j.issn.2095-1264.2021.06.19

Zhang, D., Zheng, H., Zhou, Y., Tang, X., Yu, B., & Li, J. (2007). Association of IL-1beta gene polymorphism with cachexia from locally advanced gastric cancer. *BMC Cancer*, *7*, 45. https://doi.org/10.1186/1471-2407-7-45

Zhang, D., Zhou, Y., Wu, L., Wang, S., Zheng, H., Yu, B., & Li, J. (2008). Association of IL-6 gene polymorphisms with cachexia susceptibility and survival time of patients with pancreatic cancer [Article]. *Annals of Clinical and Laboratory Science*, *38*(2), 113-119.

Zhang, F. M., Chen, X. L., Wu, Q., Dong, W. X., Dong, Q. T., Shen, X., Shi, H. P., Yu, Z., & Zhuang, C. L. (2021). Development and validation of nomograms for the prediction of low muscle mass and radiodensity in gastric cancer patients [Article]. *American Journal of Clinical Nutrition*, *113*(2), 348-358. https://doi.org/10.1093/ajcn/nqaa305

Zhang, H., Shang, X., Ren, P., Gong, L., Ahmed, A., Ma, Z., Ma, R., Wu, X., Xiao, X., Jiang, H., Tang, P., & Yu, Z. (2019). The predictive value of a preoperative systemic immune-inflammation index and prognostic nutritional index in patients with esophageal squamous cell carcinoma. *J Cell Physiol*, *234*(2), 1794-1802. https://doi.org/10.1002/jcp.27052

Zhang, J., Duan, H., Zhang, J., Qiao, H., & Jiang, J. (2024). Symptom clusters and nutritional status in primary liver cancer patients receiving transcatheter arterial chemoembolization. *Nutr Hosp*, *41*(4), 815-823. https://doi.org/10.20960/nh.04936 10.20960/nh.04936. (Grupos de síntomas y estado nutricional en pacientes con cáncer hepático primario que reciben quimioembolización arterial transcatéter.)

Zhang, L., Hu, C., Li, R., Zhang, Z., Wang, Y., Zhao, J., Liu, R., Li, Z., She, J., & Shi, F. (2023). The clinical predictive value of geriatric nutritional risk index in elderly rectal cancer patients received surgical treatment after neoadjuvant therapy. *Front Nutr*, *10*, 1237047. https://doi.org/10.3389/fnut.2023.1237047

Zhang, Q., Yu, S., Li, Q., Zhang, M., Meng, L., & Hu, S. (2022). Preoperative Nutritional Status in Elderly Inpatients with Gastrointestinal Cancer and Its Linear Association with Frailty [Article]. *Nutrition and Cancer*, *74*(4), 1376-1387. https://doi.org/10.1080/01635581.2021.1955284

Zhang, X., Dai, X. T., Wang, C., Huang, J. X., Jia, P. P., Tang, M., Song, C. H., Li, W., Shi, H. P., & Cong, M. H. (2025). A Comprehensive Analysis of the Association Between the EORTC QLQ-C30 Questionnaire and Cachexia in Patients With Gastric Cancer. *J Cachexia Sarcopenia Muscle*, *16*(3), e13859. https://doi.org/10.1002/jcsm.13859

Zhang, X., Fang, H., Zeng, Z., Zhang, K., Lin, Z., Deng, G., Deng, W., Guan, L., Wei, X., Li, X., Jiang, L., & Xu, L. (2021). Preoperative Prognostic Nutrition Index as a Prognostic Indicator of Survival in Elderly Patients Undergoing Gastric Cancer Surgery. *Cancer Manag Res*, *13*, 5263-5273. https://doi.org/10.2147/cmar.S316437

Zhang, X., Zhao, W., Chen, X., Zhao, M., Qi, X., Li, G., Shen, A., & Yang, L. (2020). Combining the Fibrinogen-to-Pre-Albumin Ratio and Prognostic Nutritional Index (FPR-PNI) Predicts the Survival in Elderly Gastric Cancer Patients After Gastrectomy. *Onco Targets Ther*, *13*, 8845-8859. https://doi.org/10.2147/ott.S264199

Zhang, Y., Wang, L. J., Li, Q. Y., Yuan, Z., Zhang, D. C., Xu, H., Yang, L., Gu, X. H., & Xu, Z. K. (2023). Prognostic value of preoperative immune-nutritional scoring systems in remnant gastric cancer patients undergoing surgery. *World J Gastrointest Surg*, *15*(2), 211-221. https://doi.org/10.4240/wjgs.v15.i2.211

Zhang, Y., Zhu, J. Y., Zhou, L. N., Tang, M., Chen, M. B., & Tao, M. (2020). Predicting the Prognosis of Gastric Cancer by Albumin/Globulin Ratio and the Prognostic Nutritional Index. *Nutr Cancer*, *72*(4), 635-644. https://doi.org/10.1080/01635581.2019.1651347

Zhang, Y. X., Yang, Y. F., Han, P., Ye, P. C., & Kong, H. (2022). Protein-energy malnutrition worsens hospitalization outcomes of patients with pancreatic cancer undergoing open pancreaticoduodenectomy. *Updates Surg*, *74*(5), 1627-1636. https://doi.org/10.1007/s13304-022-01293-7

Zhao, X., Liu, J., Wang, Y., Yang, Y., Pan, Y., & Ge, S. (2022). Preoperative Nutritional Status and Risk Factors Associated with Delayed Discharge in Geriatric Patients Undergoing Gastrectomy: A Single-Center Retrospective Study. *Appl Bionics Biomech*, *2022*, 8263986. https://doi.org/10.1155/2022/8263986

Zhao, X. H., Shen, W. B., Wang, D., Wang, H. S., Song, C. Y., & Deng, W. Z. (2023). The prognosis value of CONUT and SIS score for recurrent or metastatic esophageal squamous cell carcinoma patients treated with second-line immunotherapy. *Front Oncol*, *13*, 1167625. https://doi.org/10.3389/fonc.2023.1167625

Zhao, X. N., Lu, J., He, H. Y., & Ge, S. J. (2024). Clinical significance of preoperative nutritional status in elderly gastric cancer patients undergoing radical gastrectomy: A single-center retrospective study. *World J Gastrointest Surg*, *16*(7), 2211-2220. https://doi.org/10.4240/wjgs.v16.i7.2211

Zhao, Y., Deng, Y., Peng, J., Sui, Q., Lin, J., Qiu, M., & Pan, Z. (2018). Does the Preoperative Prognostic Nutritional Index Predict Survival in Patients with Liver Metastases from Colorectal Cancer Who Underwent Curative Resection? *J Cancer*, *9*(12), 2167-2174. https://doi.org/10.7150/jca.25346

Zhao, Y., Shen, W., Song, C., Su, J., Wu, P., Wang, X., Yan, K., Xu, J., & Zhu, S. (2022). Prognostic Significance of Prognostic Nutritional Index in Esophageal Squamous Cell Carcinoma Patients Undergoing Radical Radiotherapy: A Propensity Score Matching Analysis. *Nutr Cancer*, *74*(6), 2095-2104. https://doi.org/10.1080/01635581.2021.1982997

Zheng, H. L., Lin, J., Shen, L. L., Yang, H. B., Xu, B. B., Xue, Z., Wu, D., Huang, J. B., Lin, G. S., Zheng, C. H., Li, P., Xie, J. W., Wang, J. B., Lin, J. X., Chen, Q. Y., Cao, L. L., Lu, J., & Huang, C. M. (2023). The GLIM criteria as an effective tool for survival prediction in gastric cancer patients. *Eur J Surg Oncol*, *49*(5), 964-973. https://doi.org/10.1016/j.ejso.2023.01.009

Zheng, J., Pan, S., Li, Q., Yu, W., Shi, C., & Yu, X. (2020). Geriatric nutritional risk index predicts clinical outcomes in hepatocellular carcinoma after transarterial chemoembolization: A retrospective cohort study [Article]. *International Journal of Clinical and Experimental Medicine*, *13*(10), 7566-7576.

Zheng, J., Wang, X., Yu, J., Hu, Q., Zhan, Z., Zhou, S., Xu, J., Li, Q., Song, C., Wang, C., Zhao, Q., Xu, H., Shi, H., & Guo, Z. (2025). Global Leadership Initiative on Malnutrition criteria: Clinical benefits for patients with gastric cancer. *Nutr Clin Pract*, *40*(1), 239-251. https://doi.org/10.1002/ncp.11224

Zheng, Z., Zhu, H., & Cai, H. (2022). Preoperative Prognostic Nutritional Index Predict Survival in Patients With Resectable Esophageal Squamous Cell Carcinoma. *Front Nutr*, *9*, 824839. https://doi.org/10.3389/fnut.2022.824839

Zheng, Z. F., Lu, J., Xie, J. W., Wang, J. B., Lin, J. X., Chen, Q. Y., Cao, L. L., Lin, M., Tu, R. H., Zheng, C. H., Huang, C. M., & Li, P. (2018). Preoperative skeletal muscle index vs the controlling nutritional status score: Which is a better objective predictor of long-term survival for gastric cancer patients after radical gastrectomy? *Cancer Med*, *7*(8), 3537-3547. https://doi.org/10.1002/cam4.1548

Zhou, C. J., Cheng, Y. F., Xie, L. Z., Hu, W. L., Chen, B., Xu, L., Huang, C. J., Cai, M., Shen, X., & Liu, C. B. (2020). Metabolic Syndrome, as Defined Based on Parameters Including Visceral Fat Area, Predicts Complications After Surgery for Rectal Cancer. *Obes Surg*, *30*(1), 319-326. https://doi.org/10.1007/s11695-019-04163-1

Zhou, H., Wang, W., Zhang, R., Dong, H., Wang, G., Chen, W., & Li, G.-D. (2022). Comparison of the prognostic value of prognostic nutritional index in colorectal cancer deaths:a retrospective cohort study. *Food Science and Technology*, *42*, NA-NA. https://doi.org/10.1590/fst.36320

Zhou, J., & Xie, F. (2022). Predictive significance of modified Glasgow prognostic score and serum carcinoembryonic antigen level in liver metastasis from colorectal cancer [Article]. *Journal of Practical Oncology*, *37*(5), 424-432. https://doi.org/10.13267/j.cnki.syzlzz.2022.072

Zhou, L. P., Yu, D. Y., Ma, B. W., Shen, Z. L., Zou, H. B., Zhang, X. Z., Yan, X. L., Zhuang, C. L., & Yu, Z. (2021). Feasibility of substituting handgrip strength for muscle mass as a constituent standard in the Global Leadership Initiative on Malnutrition for diagnosing malnutrition in patients with gastrointestinal cancers. *Nutrition*, *84*, 111044. https://doi.org/10.1016/j.nut.2020.111044

Zhou, X., Qiu, G. Q., Bao, W. A., & Zhang, D. H. (2017). The prognostic role of nutrition risk score (NRS) in patients with metastatic or recurrent esophageal squamous cell carcinoma (ESCC). *Oncotarget*, *8*(44), 77465-77473. https://doi.org/10.18632/oncotarget.20530

Zhu, C., Wang, X., Chen, S., Yang, X., Sun, J., Pan, B., Zhang, W., Chen, X., & Huang, Y. (2020). Efficacy of the Preoperative Albumin-Bilirubin Grade for Predicting Survival and Outcomes of Postoperative Chemotherapy for Advanced Gastric Cancer. *Cancer Manag Res*, *12*, 11921-11932. https://doi.org/10.2147/cmar.S279782

Zhu, X., Xu, Q., Zhou, Y., Zhu, C., & Zeng, L. (2025). The prognostic value of the neutrophil-to-lymphocyte ratio, platelet-to-lymphocyte ratio, and prognostic nutritional index for survival in patients with colorectal cancer.

Open Med (Wars), *20*(1), 20251214. https://doi.org/10.1515/med-2025-1214 10.1515/med-2025-1214. eCollection 2025.

Zhu, X., Zhao, Y., Ma, F., & Wu, S. (2021). Controlling Nutritional Status score predict the individualized survival of patients with gastric cancer. *Asia Pac J Clin Nutr*, *30*(1), 51-59. https://doi.org/10.6133/apjcn.202103_30(1).0007

Zhu, Y., Fan, L., Geng, X., & Li, J. (2021). The predictive value of the prognostic nutritional index to postoperative prognosis and nursing intervention measures for colorectal cancer [Article]. *American Journal of Translational Research*, *13*(12), 14096-14101.

Zhuang, C. L., Dong, Q. T., Shi, H. P., Zhang, F. M., Luo, X., Wang, W. B., Yu, Z., Chen, X. L., & Wang, S. L. (2022). Cachexia Versus Sarcopenia in Clinical Characteristics and Prognostic Value After Radical Gastrectomy for Gastric Cancer: A Large-Scale Prospective Study [Article]. *Annals of Surgical Oncology*, *29*(4), 2348-2358. https://doi.org/10.1245/s10434-021-11084-w

Zou, W., Kuang, W., Cai, C., & Qian, Y. (2024). Prognostic Nutritional Index as a Prognostic Indicator for the Occurrence of Postoperative Complications in Patients with Esophageal Squamous Cell Carcinoma Following Neoadjuvant Immunochemotherapy. *Cancer Manag Res*, *16*, 643-650. https://doi.org/10.2147/cmar.S465501

Zou, Y., Li, L., Jia, K., Tian, L., He, M., & Huang, D. (2025). Effect of preoperative nutritional risk index on 30-day postoperative complications in patients with gastric cancer: a retrospective cohort study. *Front Oncol*, *15*, 1475381. https://doi.org/10.3389/fonc.2025.1475381

Zuo, J., Huang, Y., Huang, Z., Zhang, J., Hou, W., Wang, C., Wang, X., & Bu, X. (2025). Comparison of three objective nutritional screening tools for identifying GLIM-defined malnutrition in patients with gastric cancer. *Eur J Clin Nutr*, *79*(1), 64-70. https://doi.org/10.1038/s41430-024-01514-9

Zuo, J., Huang, Z., Ge, Y., Ding, X., Wang, X., & Zhou, X. (2024). Geriatric Nutrition Risk Index is closely associated with sarcopenia and quality of life in gastric cancer patients: a cross-sectional study. *Sci Rep*, *14*(1), 31545. https://doi.org/10.1038/s41598-024-83380-w

Zuo, J., Zhou, D., Zhang, L., Zhou, X., Gao, X., Hou, W., Wang, C., Jiang, P., & Wang, X. (2024). Comparison of bioelectrical impedance analysis and computed tomography for the assessment of muscle mass in patients with gastric cancer. *Nutrition*, *121*, 112363. https://doi.org/10.1016/j.nut.2024.112363

Zuo, J., Zhou, D., Zhang, L., Zhou, X., Gao, X., Zhang, J., Ding, X., Hou, W., Wang, C., Jiang, P., & Wang, X. (2024). Phase angle - A screening tool for malnutrition, sarcopenia, and complications in gastric cancer. *Clin Nutr ESPEN*, *59*, 334-342. https://doi.org/10.1016/j.clnesp.2023.12.004

Supplementary Document S9: Detailed strategy for retrieval and reasons for excluding articles

Strategy for retrieval: We have emailed the corresponding authors.

Reasons for excluding articles: During the full-text selection, we excluded 1305 articles because of the following reasons: 248 - outcome of interest is not reported, 382 not only gastrointestinal cancer patients, 472 – prevalence of malnutrition is not reported, 93 - ineligible study type, and 110 – the definition of malnutrition is not provided.

During the manual data retrieval, if odds (OR) with the corresponding 95% confidence interval (Cl) were not reported, the available raw data were used (which is the number of patients in each group of interest-exposed patients experiencing the outcome, exposed patients not experiencing the outcome, non-exposed patients experiencing the outcomes, and non-exposed patients not experiencing the outcome, respectively)

Supplementary Document S10: Individual forest plots for population characteristics: Figure S1-S75.

Figure S1.: Association between malnutrition-related complication risk and age (cut off 65) in gastrointestinal cancer (Biological composite scores)

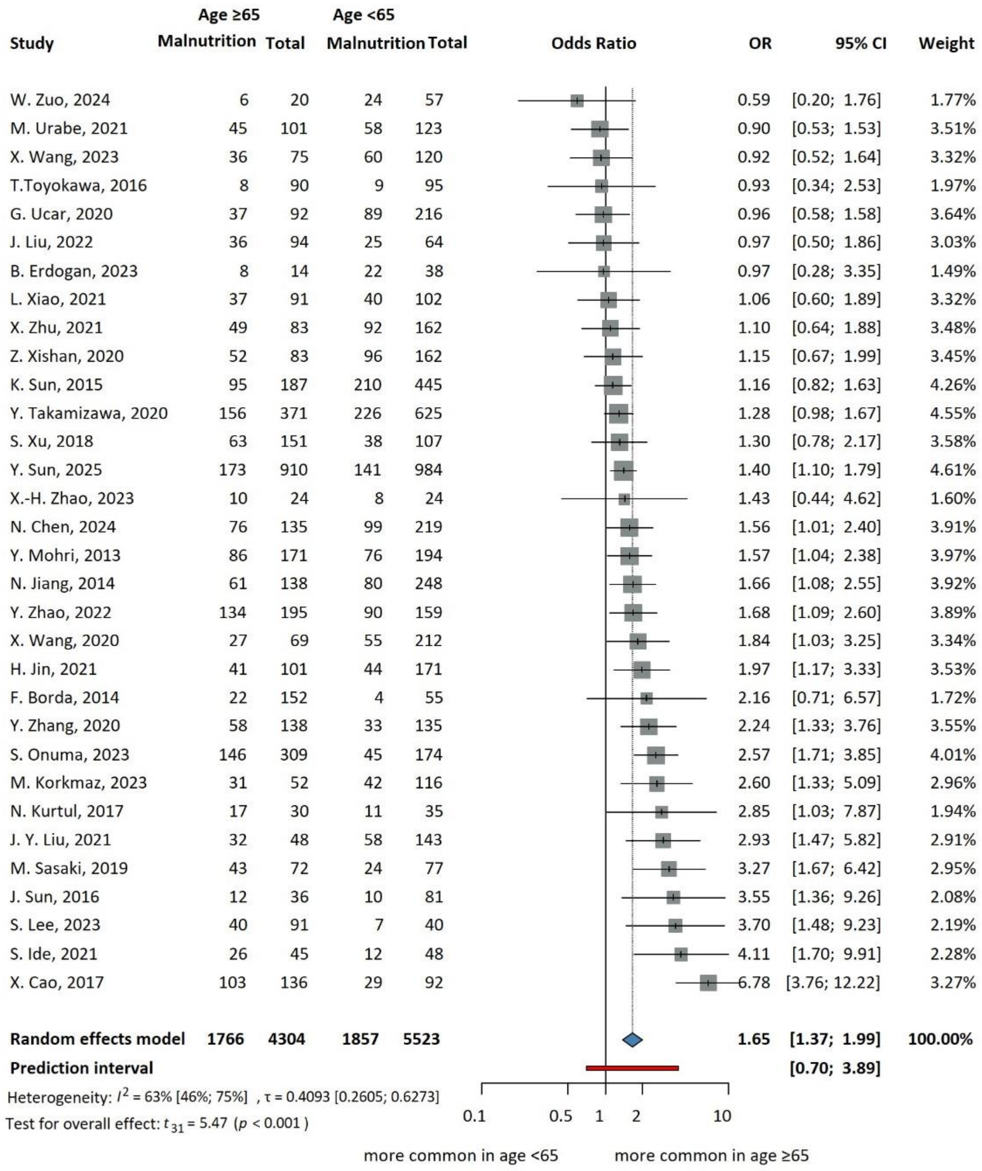

Figure S2.: Association between malnutrition-related complication risk and age (cut off 65) in esophageal cancer (Biological composite scores)

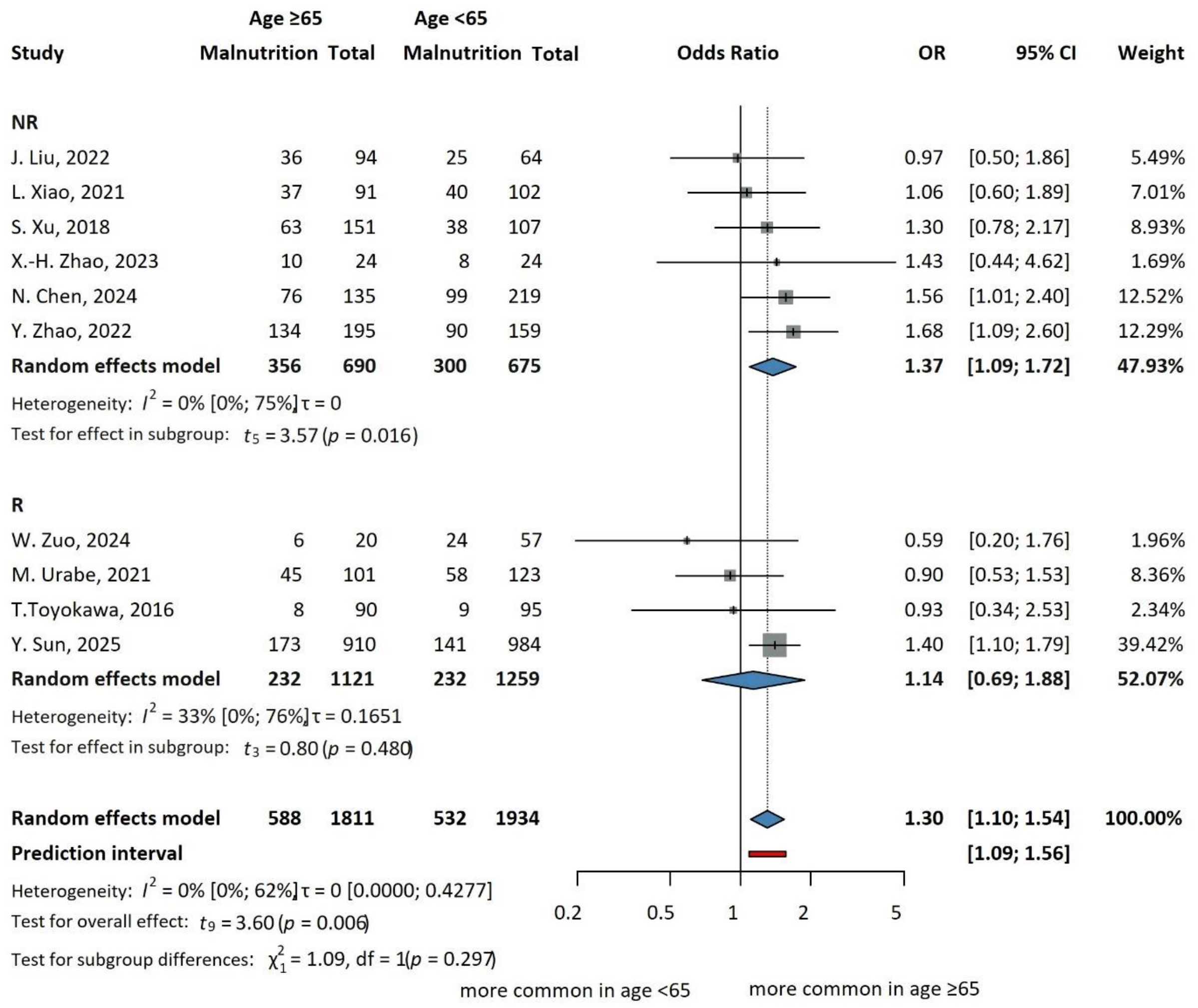

Abbreviation: R=secetable; NR= non-resectable

Figure S3.: Association between malnutrition-related complication risk and age (cut off 65) in gastric cancer (Biological composite scores)

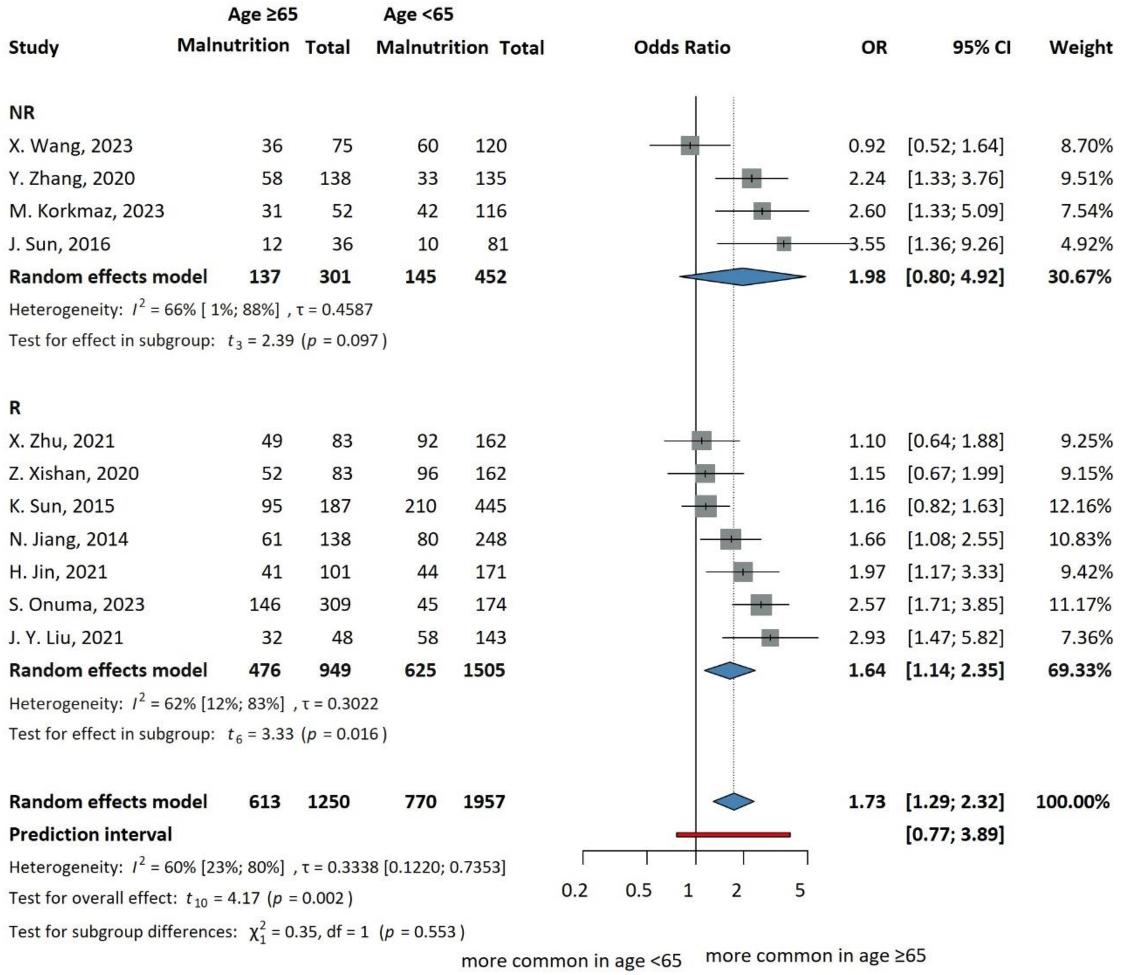

Abbreviation: R=secetable; NR= non-resectable

Figure S4.: Association between malnutrition-related complication risk and age (cut off 65) in colorectal cancer (Biological composite scores)

Study	Age ≥65 Malnutrition	Age ≥65 Total	Age <65 Malnutrition	Age <65 Total	OR	95% CI	Weight
G. Ucar, 2020	37	92	89	216	0.96	[0.58; 1.58]	11.08%
B. Erdogan, 2023	8	14	22	38	0.97	[0.28; 3.35]	5.52%
Y. Takamizawa, 2020	156	371	226	625	1.28	[0.98; 1.67]	12.88%
Y. Mohri, 2013	86	171	76	194	1.57	[1.04; 2.38]	11.77%
X. Wang, 2020	27	69	55	212	1.84	[1.03; 3.25]	10.42%
F. Borda, 2014	22	152	4	55	2.16	[0.71; 6.57]	6.24%
N. Kurtul, 2017	17	30	11	35	2.85	[1.03; 7.87]	6.87%
M. Sasaki, 2019	43	72	24	77	3.27	[1.67; 6.42]	9.54%
S. Lee, 2023	40	91	7	40	3.70	[1.48; 9.23]	7.58%
S. Ide, 2021	26	45	12	48	4.11	[1.70; 9.91]	7.83%
X. Cao, 2017	103	136	29	92	6.78	[3.76; 12.22]	10.28%
**Random effects model**	**565**	**1243**	**555**	**1632**	**2.19**	**[1.42; 3.35]**	**100.00%**
**Prediction interval**						**[0.63; 7.62]**	

Heterogeneity: I^2 = 77% [58%; 87%] τ = 0.5179 [0.2867; 1.0351]

Test for overall effect: t_{10} = 4.07 (p = 0.002)

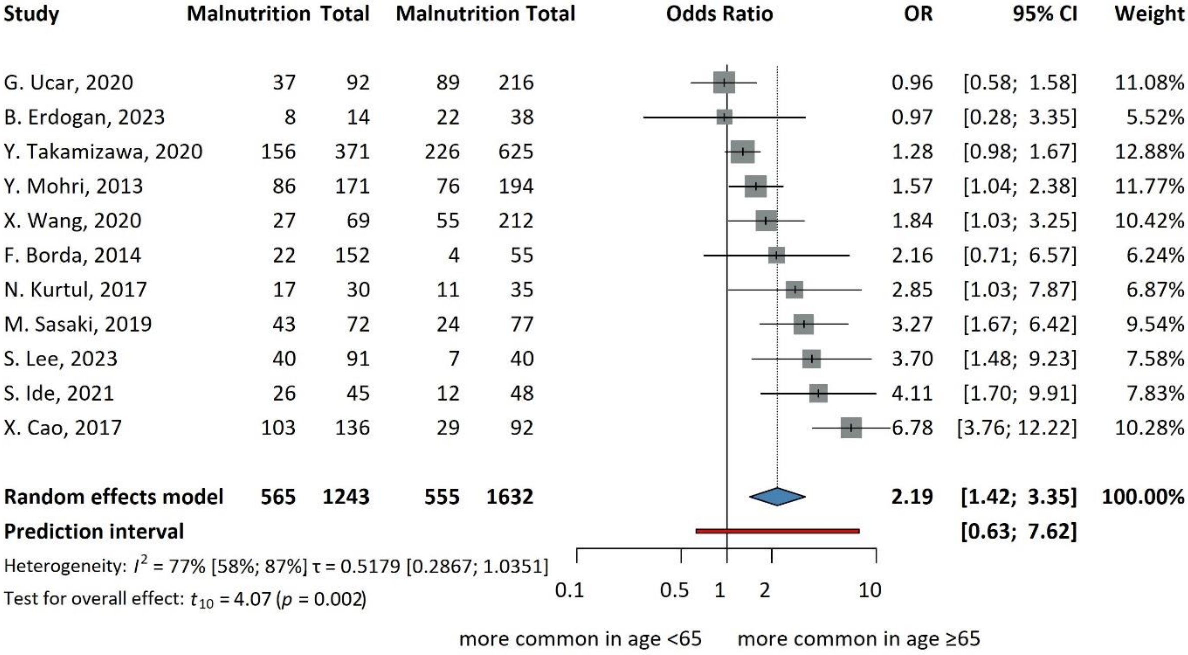

Figure 5: Association between malnutrition-related complication risk and age (cut off 65) in hepato-biliopancreatic cancer (Biological composite scores)

Study	Age ≥65 Malnutrition	Age ≥65 Total	Age <65 Malnutrition	Age <65 Total	OR	95% CI	Weight
Z. X. Lin, 2020	19	44	168	336	0.76	[0.40; 1.43]	14.56%
L. Sun, 2021	71	150	99	221	1.11	[0.73; 1.68]	32.48%
Y. Chen, 2020	13	58	49	267	1.29	[0.64; 2.56]	12.32%
Y. Si, 2022	42	78	69	157	1.49	[0.86; 2.57]	19.45%
Y. Yang, 2020	22	85	57	318	1.60	[0.91; 2.81]	18.26%
S. Sun, 2024	5	10	10	39	2.90	[0.69; 12.15]	2.92%
**Random effects model**	**172**	**425**	**452**	**1338**	**1.24**	**[0.90; 1.72]**	**100.00%**

Heterogeneity: I^2 = 3% [0%; 75%] τ = 0.0577 [0.0000; 0.9393]

Test for overall effect: t_5 = 1.74 (p = 0.142)

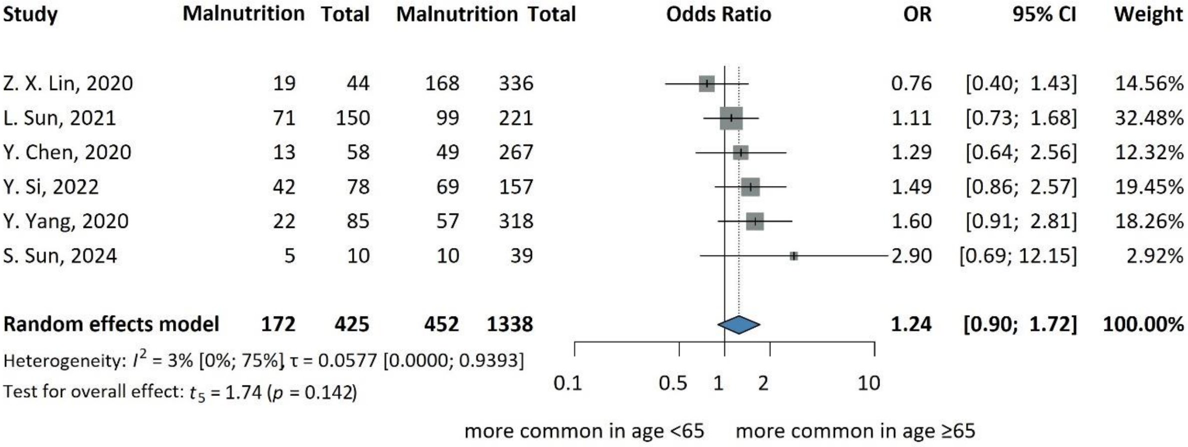

Figure S6.: Association between malnutrition-related complication risk and age (cut off 60) in esophageal cancer (Biological composite scores)

Study	Age ≥ 60 Malnutrition	Age ≥ 60 Total	Age <60 Malnutrition	Age <60 Total	OR	95% CI	Weight
J. F. Feng, 2013	113	161	157	214	0.85	[0.54; 1.35]	14.72%
L. Han, 2016	19	112	15	94	1.08	[0.51; 2.26]	7.37%
X. Xu, 2022	58	212	37	158	1.23	[0.77; 1.98]	13.87%
J. Kang, 2022	652	1567	340	1094	1.58	[1.34; 1.86]	30.78%
P. Fang, 2022	55	224	25	149	1.61	[0.95; 2.73]	12.19%
S. B. Chen, 2021	170	317	117	303	1.84	[1.34; 2.53]	21.07%
**Random effects model**	**1067**	**2593**	**691**	**2012**	**1.40**	**[1.04; 1.88]**	**100.00%**
**Prediction interval**						**[0.76; 2.60]**	

Heterogeneity: I^2 = 47% [0%; 79%], τ^2 = 0.04 p = 0.096

Test for overall effect: t_5 = 2.95 (p = 0.032)

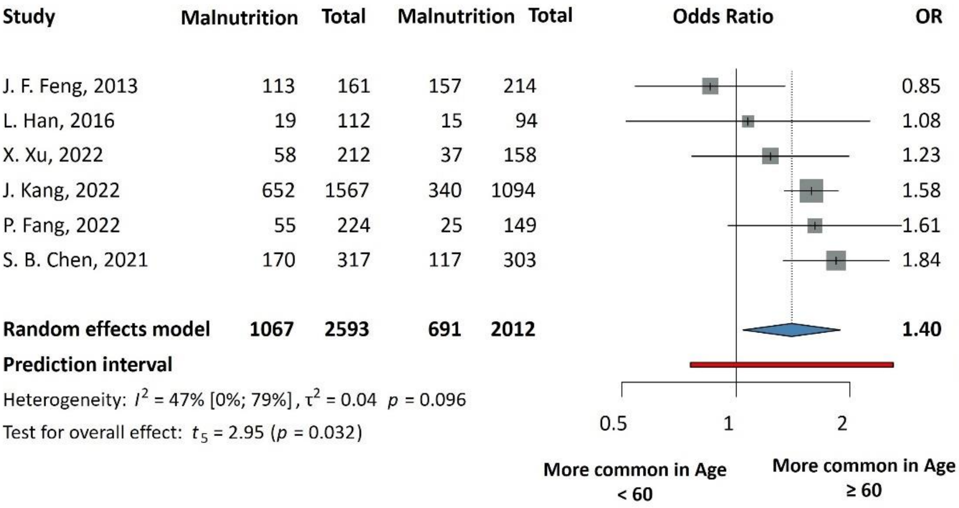

Figure S7.: Association between malnutrition-related complication risk and age (cut off 60) in gastric cancer (Biological composite scores)

Study	Age ≥60 Malnutrition	Age ≥60 Total	Age <60 Malnutrition	Age <60 Total	OR	95% CI	Weight
Y. Jing, 2024	222	243	233	247	0.64	[0.32; 1.28]	6.90%
Y. Xu, 2024	18	71	20	75	0.93	[0.45; 1.96]	6.68%
Y. Han, 2023	157	366	125	281	0.94	[0.69; 1.28]	8.95%
Q. Xiao, 2022	51	84	12	22	1.29	[0.50; 3.32]	5.58%
L. Wang, 2020	67	107	35	63	1.34	[0.71; 2.52]	7.29%
X. Liu, 2018	99	283	118	414	1.35	[0.98; 1.87]	8.91%
Z. Xu, 2022	208	319	251	452	1.50	[1.12; 2.02]	9.03%
X. Zhang, 2020	72	166	33	108	1.74	[1.04; 2.90]	7.97%
H. Song, 2022	46	103	29	99	1.95	[1.09; 3.48]	7.58%
S. Lin, 2025	214	287	122	204	1.97	[1.34; 2.90]	8.62%
J. Y. Lee, 2016	534	3624	245	4157	2.76	[2.36; 3.23]	9.48%
X. Ma, 2025	63	321	4	83	4.82	[1.70; 13.67]	5.13%
H. X. Wang, 2018	97	133	40	141	6.80	[4.01; 11.55]	7.87%
**Random effects model**	**1848**	**6107**	**1267**	**6346**	**1.71**	**[1.17; 2.51]**	**100.00%**
**Prediction interval**						**[0.47; 6.29]**	

Heterogeneity: I^2 = 86% [78%; 91%] τ = 0.5644 [0.3471; 1.0258]

Test for overall effect: t_{12} = 3.07 (p = 0.010)

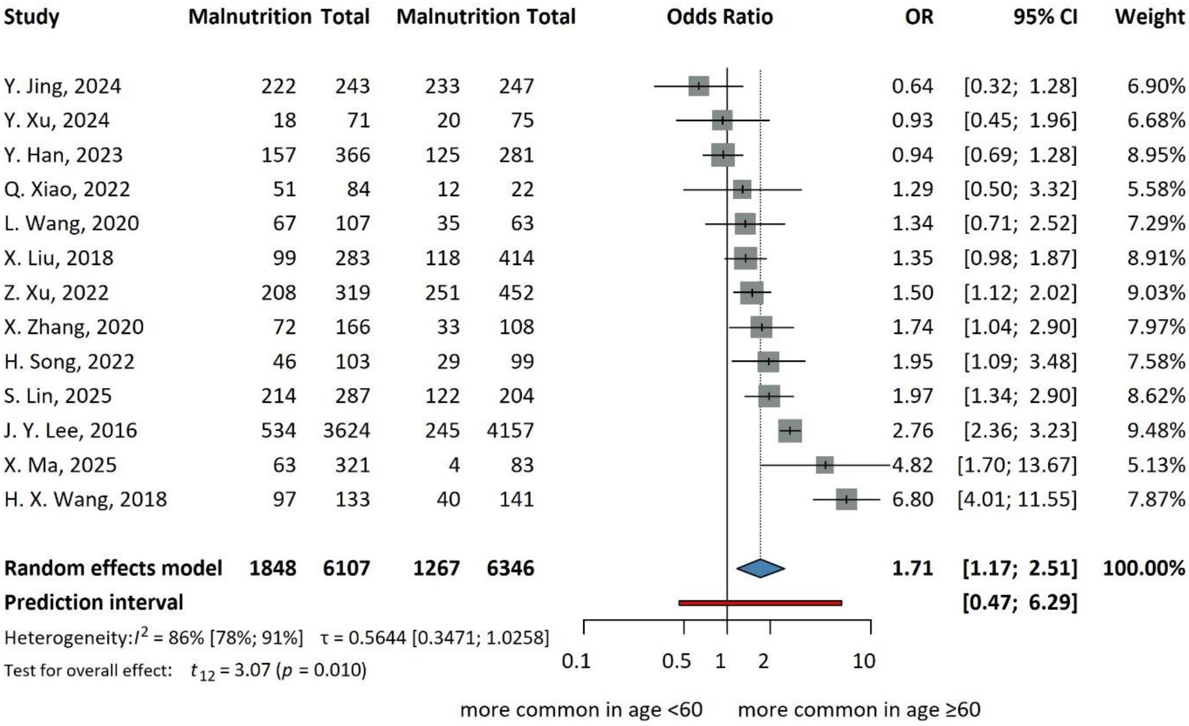

Figure S8.: Association between malnutrition-related complication risk and age (cut off 60) in colorectal cancer (Biological composite scores)

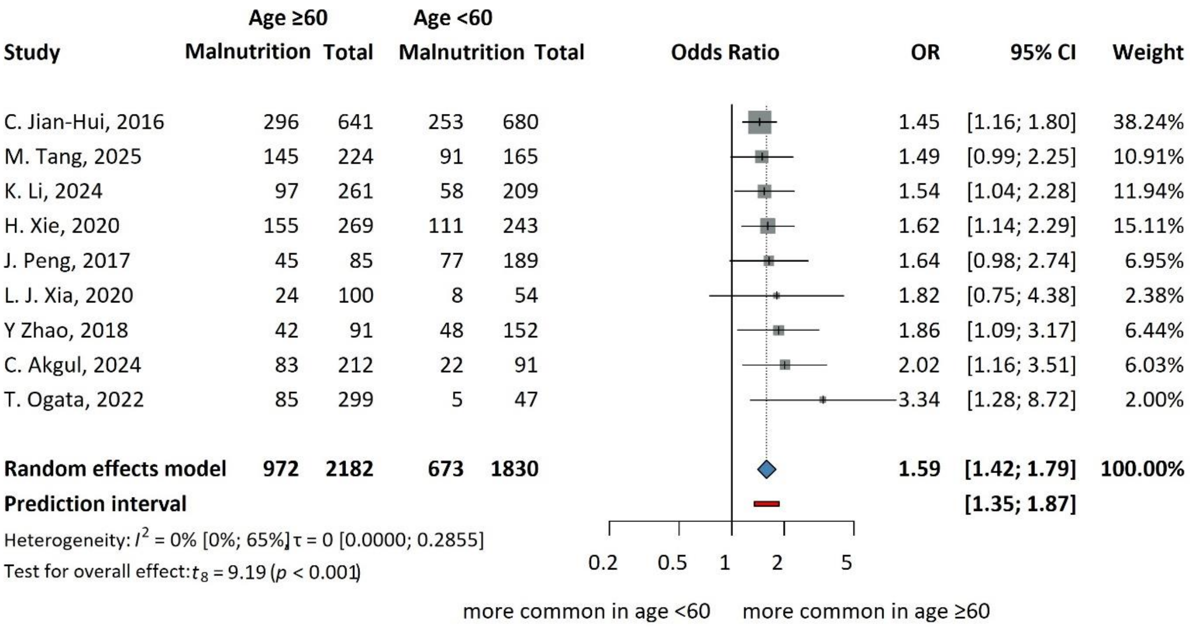

Figure S9.: Association between malnutrition risk and sex in esophageal cancer (Symptom-based risk assessment tools)

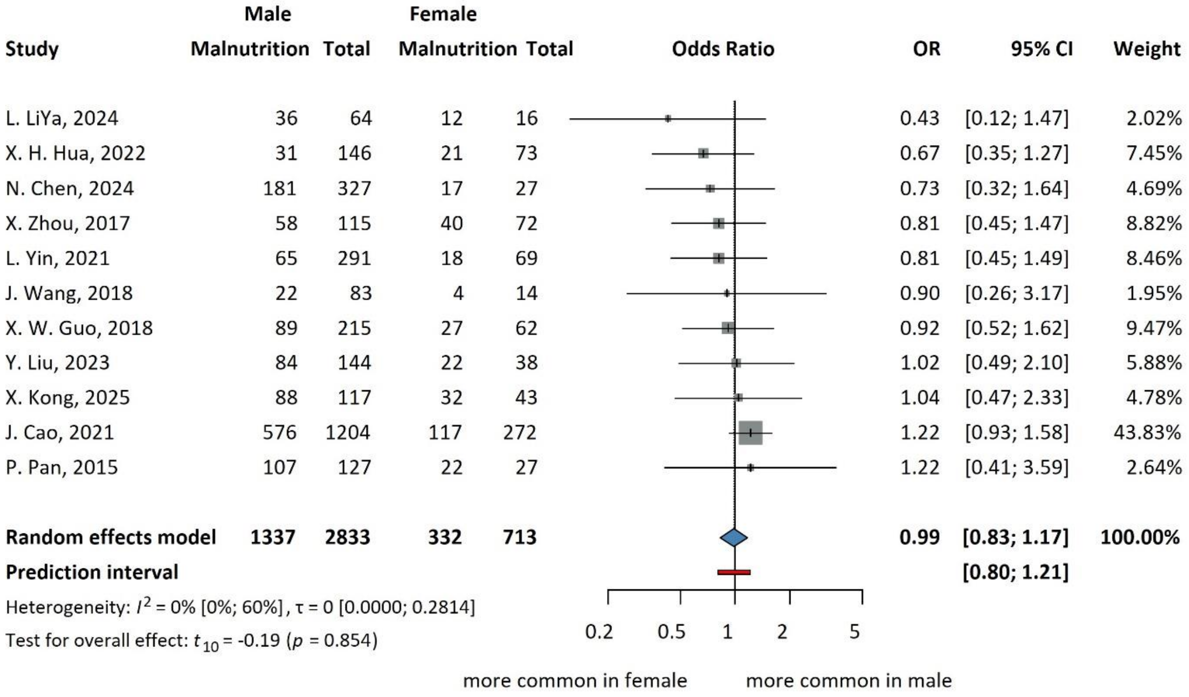

Figure S10.: Association between malnutrition risk and sex in gastric cancer (Symptom-based risk assessment tools)

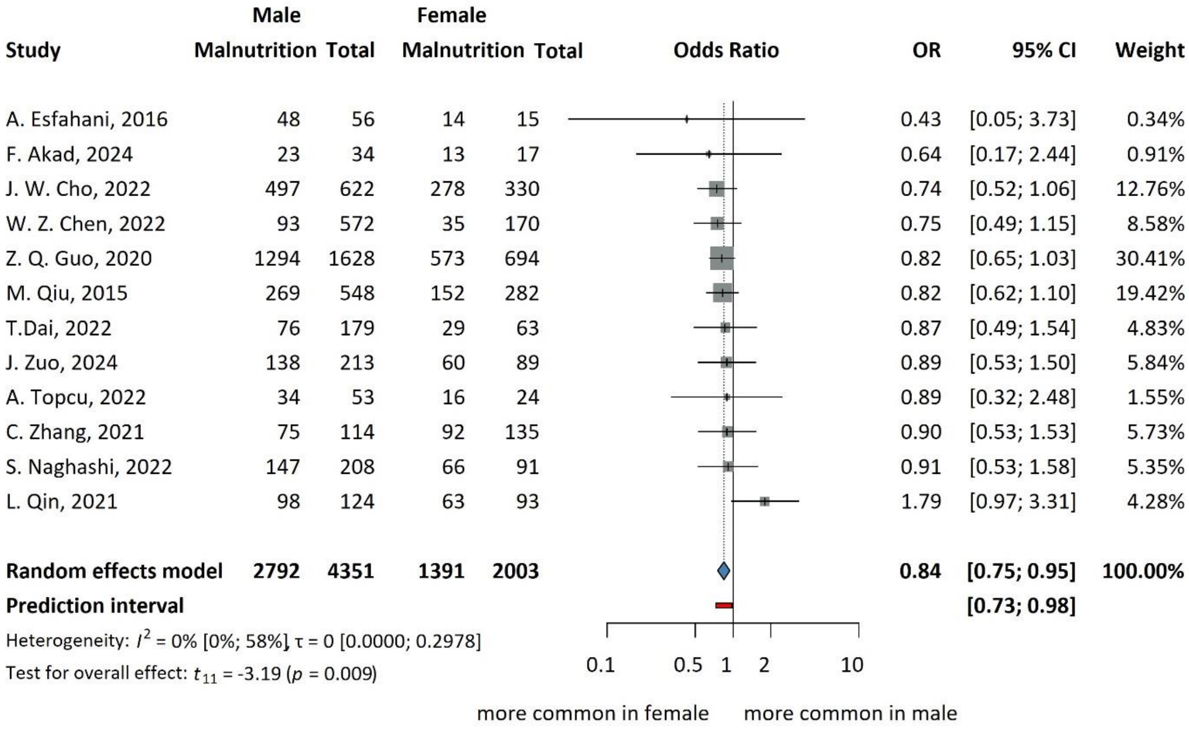

Figure S11.: Association between malnutrition risk and sex in colorectal cancer (Symptom-based risk assessment tools)

Study	Male Malnutrition	Male Total	Female Malnutrition	Female Total	OR	95% CI	Weight
**NR**							
W. J. Wang, 2020	58	77	48	54	0.38	[0.14; 1.03]	2.62%
R. Obaid, 2025	22	37	18	28	0.81	[0.30; 2.25]	2.54%
K. Tanaka, 2021	23	68	20	59	1.00	[0.48; 2.08]	4.02%
L. T. Ngoc Anh, 2024	197	224	144	164	1.01	[0.55; 1.88]	4.99%
J. Szefel, 2020	1	34	1	36	1.06	[0.06; 17.66]	0.41%
R. N. Velasco, 2022	117	152	106	140	1.07	[0.62; 1.84]	5.74%
B. U. Souza, 2018	37	112	26	85	1.12	[0.61; 2.05]	5.08%
N. C. Souza, 2021	35	109	24	81	1.12	[0.60; 2.10]	4.92%
G. Wong, 2021	53	176	42	157	1.18	[0.73; 1.90]	6.44%
A. da Silva Couto, 2023	37	110	24	81	1.20	[0.65; 2.24]	4.96%
T. Kapagan, 2025	56	91	31	59	1.45	[0.74; 2.80]	4.59%
K. Fu, 2017	76	187	39	123	1.47	[0.91; 2.38]	6.43%
**Random effects model**	**712**	**1377**	**523**	**1067**	**1.13**	**[0.95; 1.33]**	**52.74%**
**Prediction interval**						**[0.91; 1.39]**	
Heterogeneity: $I^2 = 0\%$ [0%; 58%] $\tau = 0$							
Test for effect in subgroup: $t_{11} = 1.57$ ($p = 0.145$)							
**R**							
L. R. Barbosa, 2014	4	30	20	36	0.12	[0.04; 0.43]	1.83%
V. Lohsiriwat, 2014	23	76	30	73	0.62	[0.32; 1.22]	4.48%
A. S. Almasaudi, 2019	36	199	39	164	0.71	[0.43; 1.18]	6.08%
W. Chen, 2023	62	558	43	292	0.72	[0.48; 1.10]	7.18%
C. Gillis, 2021	89	154	73	112	0.73	[0.44; 1.21]	6.15%
X. Wang, 2020	40	70	32	50	0.75	[0.36; 1.58]	3.95%
Y. Wang, 2022	278	932	208	705	1.02	[0.82; 1.26]	9.90%
F. Teraishi, 2024	21	34	22	37	1.10	[0.42; 2.86]	2.80%
Y. Wang, 2018	155	188	55	76	1.79	[0.96; 3.36]	4.89%
**Random effects model**	**708**	**2241**	**522**	**1545**	**0.77**	**[0.49; 1.22]**	**47.26%**
**Prediction interval**						**[0.22; 2.73]**	
Heterogeneity: $I^2 = 60\%$ [16%; 81%] $\tau = 0.4948$							
Test for effect in subgroup: $t_8 = -1.30$ ($p = 0.230$)							
**Random effects model**	**1420**	**3618**	**1045**	**2612**	**0.95**	**[0.78; 1.15]**	**100.00%**
**Prediction interval**						**[0.51; 1.74]**	

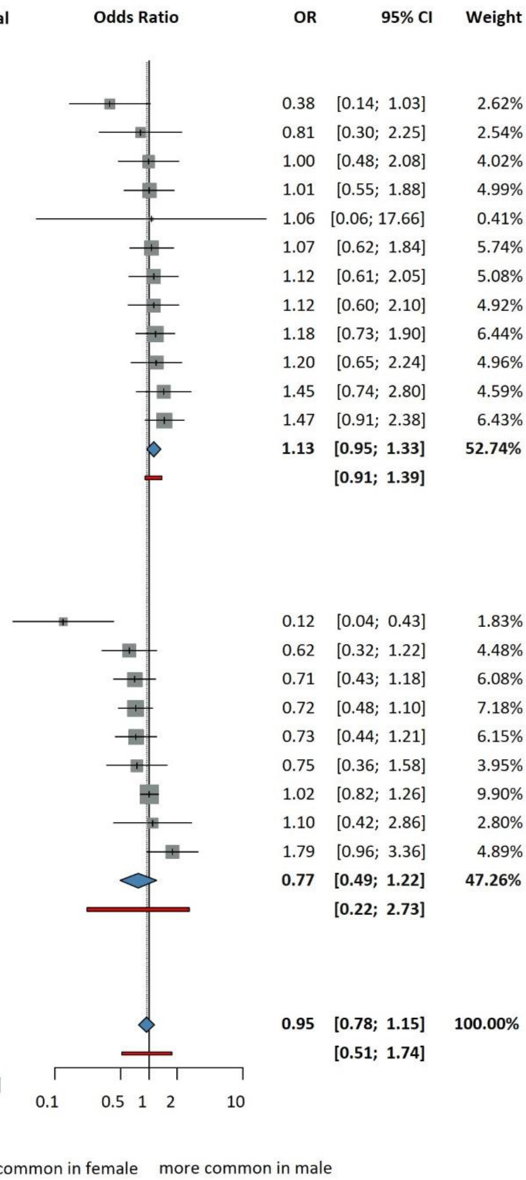

Heterogeneity: $I^2 = 35\%$ [0%; 62%] $\tau = 0.2764$ [0.0000; 0.6209]
Test for overall effect: $t_{20} = -0.58$ ($p = 0.569$)
Test for subgroup differences: $\chi^2_1 = 3.15$, df = 1 ($p = 0.076$)

Abbreviation: R= resectable; NR=non-resectable

Figure S12.: Association between malnutrition risk and sex in hepatocellular carcinoma cancer (Symptom-based risk assessment tools)

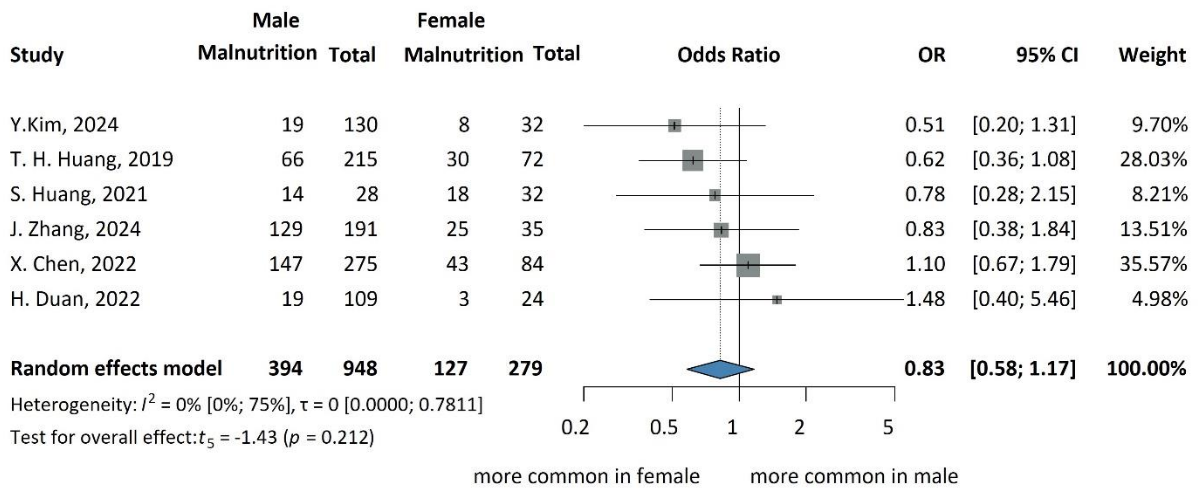

Figure S13.: Association between malnutrition risk and sex in pancreatic ductal adenocarcinoma (Symptom-based risk assessment tools)

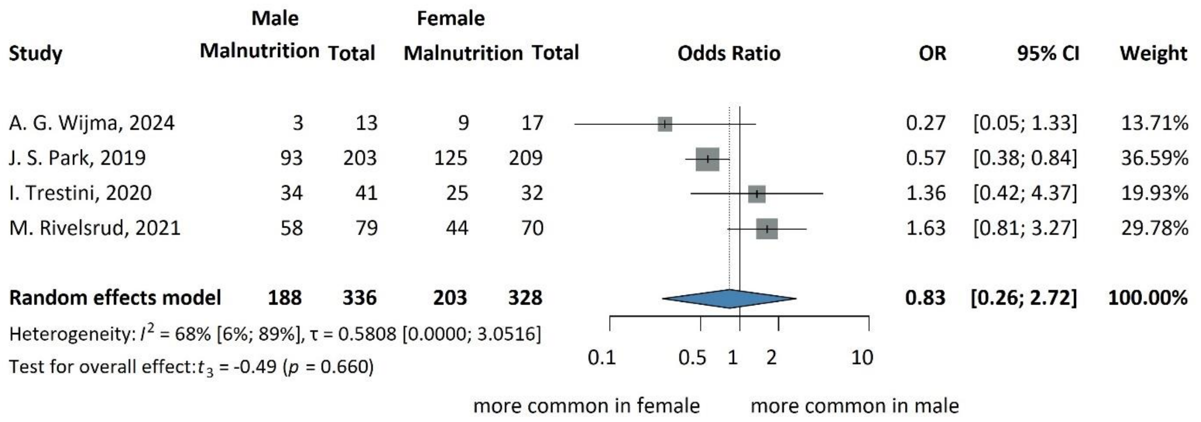

Figure S14.: Association between malnutrition diagnosis and sex in resectable gastrointestinal cancer (GLIM criteria)

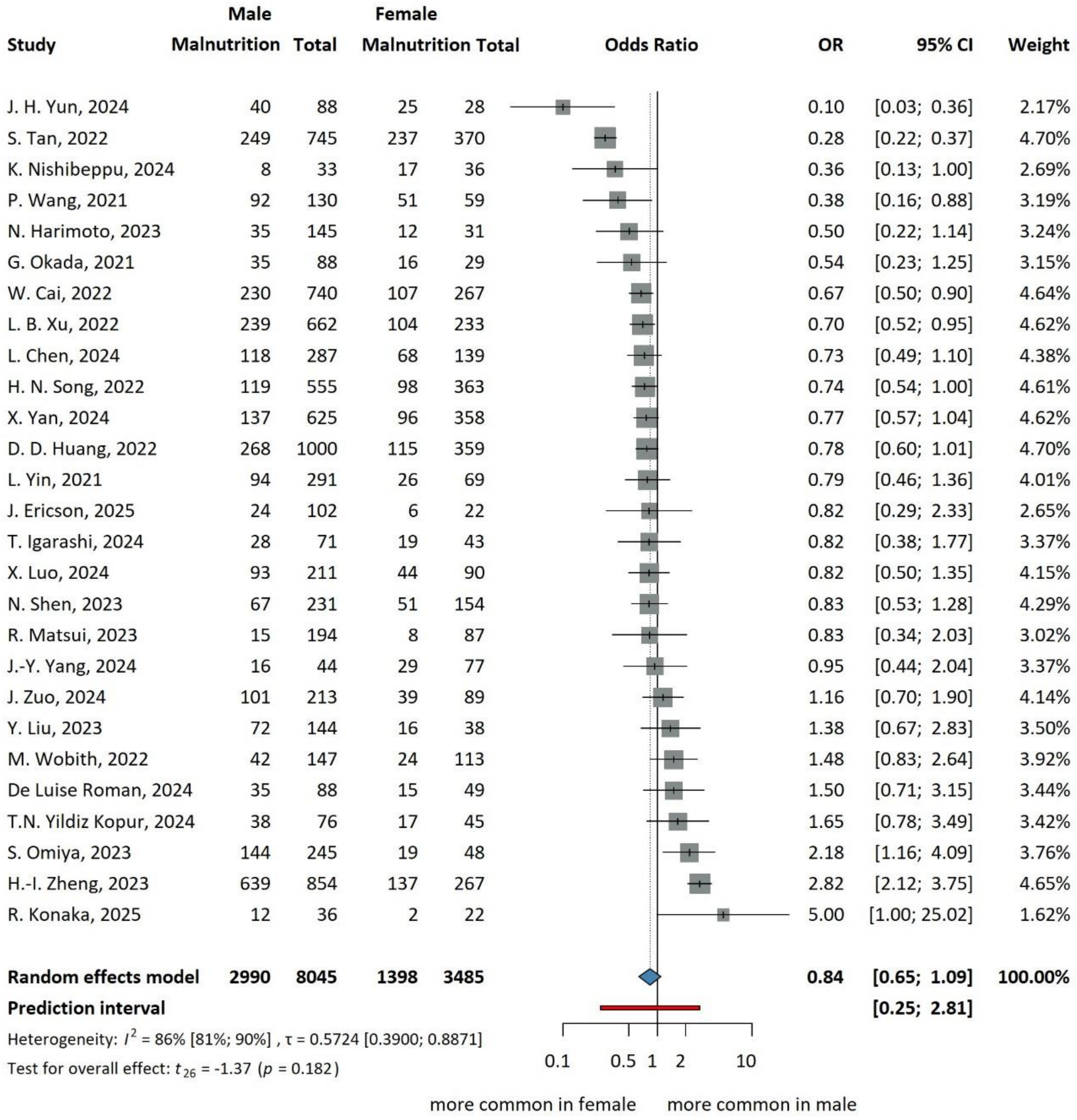

Figure S15.: Association between malnutrition diagnosis and sex in esophageal cancer (GLIM criteria)

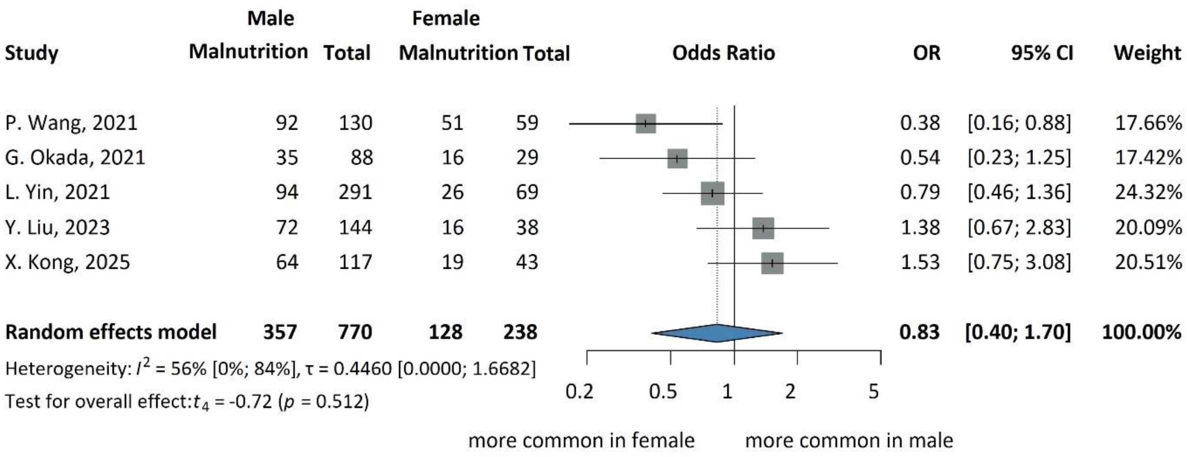

Figure S16.: Association between malnutrition diagnosis and sex in gastric cancer (GLIM criteria)

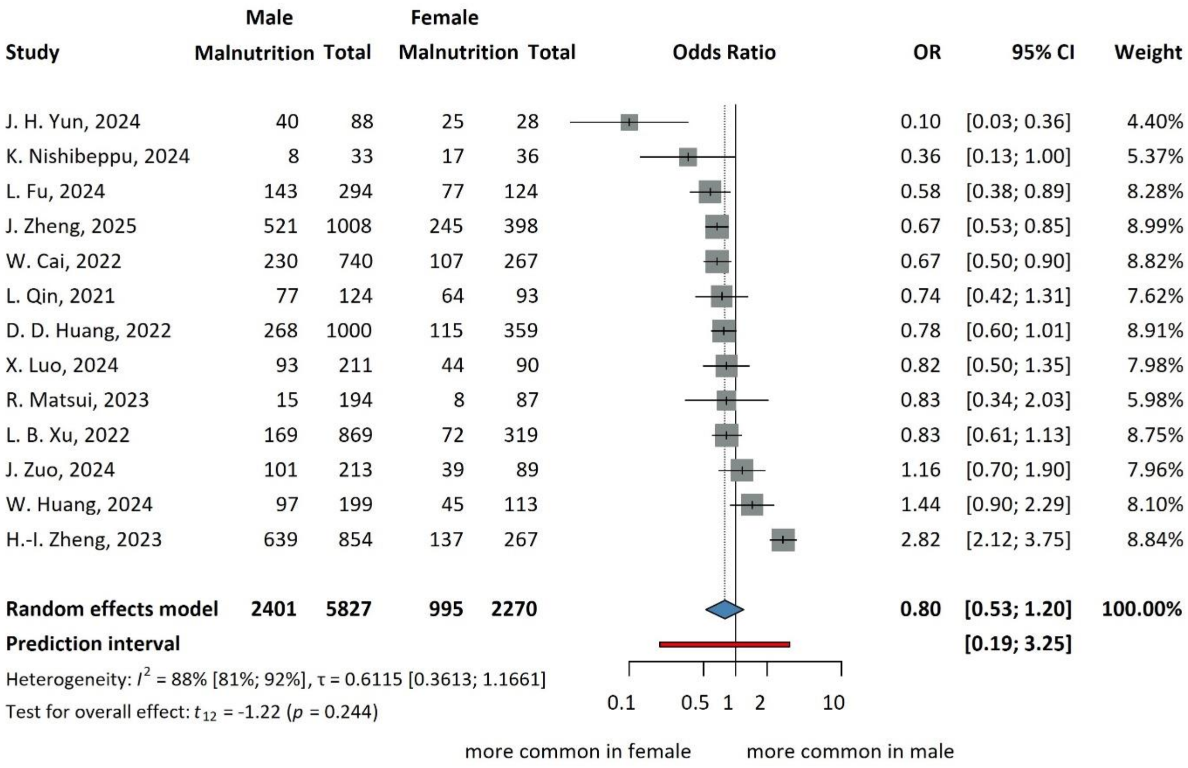

Abbreviation: R= resectable; NR= non-resectable

Figure S17.: Association between malnutrition-related complication risk and sex in esophageal cancer (Biological composite scores)

Study	Male Malnutrition	Male Total	Female Malnutrition	Female Total	OR	95% CI	Weight
**NR**							
F. K. Xiao, 2021	90	2623	450	1523	0.08	[0.07; 0.11]	3.30%
Y. Matsumoto, 2018	49	158	13	33	0.69	[0.32; 1.50]	2.16%
Y. Zhao, 2022	125	206	99	148	0.76	[0.49; 1.19]	2.91%
S. Xu, 2018	69	182	32	76	0.84	[0.49; 1.45]	2.68%
N. Chen, 2024	161	327	14	27	0.90	[0.41; 1.98]	2.14%
K. Yan, 2022	59	111	44	81	0.95	[0.54; 1.69]	2.61%
L. Xiao, 2021	60	149	17	44	1.07	[0.54; 2.13]	2.35%
Y. Yu, 2022	111	769	39	299	1.12	[0.76; 1.66]	3.02%
J. Liu, 2022	42	101	19	57	1.42	[0.72; 2.80]	2.37%
M. Demir, 2022	26	44	25	57	1.85	[0.83; 4.10]	2.11%
X.-H. Zhao, 2023	14	32	4	16	2.33	[0.62; 8.82]	1.24%
L. Chang, 2022	27	67	0	2	3.40	[0.16; 73.48]	0.33%
**Random effects model**	**833**	**4769**	**756**	**2363**	**0.85**	**[0.48; 1.51]**	**27.21%**
**Prediction interval**						**[0.13; 5.56]**	
Heterogeneity: $I^2 = 96\%$ [94%; 97%], $\tau = 0.8019$							
Test for effect in subgroup: $t_{11} = -0.62$ ($p = 0.546$)							
**R**							
Y. Fujiwara, 2021	14	93	7	18	0.28	[0.09; 0.84]	1.55%
J. Feng, 2022	139	203	11	13	0.39	[0.09; 1.83]	1.02%
R. Yamanaka-Kohno, 2021	17	68	2	5	0.50	[0.08; 3.25]	0.76%
M. Sonoi, 2025	15	69	4	12	0.56	[0.15; 2.10]	1.24%
W. Zuo, 2024	23	62	7	15	0.67	[0.22; 2.10]	1.50%
I. Hashimoto, 2024	30	166	9	37	0.69	[0.29; 1.60]	2.01%
N. Kubo, 2017	22	162	7	40	0.74	[0.29; 1.88]	1.85%
M. Nakatani, 2018	36	66	6	10	0.80	[0.21; 3.10]	1.21%
I. Nonogaki, 2023	156	394	30	70	0.87	[0.52; 1.46]	2.75%
H. Ma, 2024	47	181	18	65	0.92	[0.48; 1.73]	2.47%
M. Urabe, 2021	84	183	19	41	0.98	[0.50; 1.94]	2.37%
K. Takao, 2020	52	130	14	35	1.00	[0.47; 2.14]	2.19%
X. Xu, 2022	63	245	32	125	1.01	[0.61; 1.65]	2.80%
J. F. Feng, 2013	235	326	35	49	1.03	[0.53; 2.01]	2.40%
S. B. Chen, 2021	223	477	64	143	1.08	[0.74; 1.58]	3.05%
N. Hirahara, 2018	70	132	8	16	1.13	[0.40; 3.19]	1.66%
K. Ge, 2021	128	308	42	112	1.19	[0.76; 1.85]	2.90%
P. Fang, 2022	67	305	13	68	1.19	[0.61; 2.31]	2.41%
Y. Sun, 2025	273	1599	41	295	1.28	[0.89; 1.82]	3.09%
Y. Bo, 2016	37	150	18	89	1.29	[0.68; 2.44]	2.47%
H. Zhang, 2018	217	537	37	118	1.48	[0.97; 2.27]	2.95%
T. Miyazaki, 2016	60	173	5	19	1.49	[0.51; 4.33]	1.61%
G. H. A-Lai, 2019	37	71	8	19	1.50	[0.54; 4.16]	1.68%
L. Han, 2016	29	165	5	41	1.54	[0.55; 4.25]	1.69%
J. Kang, 2022	852	2173	140	488	1.60	[1.29; 1.99]	3.34%
K. Migita, 2018	40	116	5	21	1.68	[0.57; 4.93]	1.60%
K. Okadome, 2020	108	300	9	37	1.75	[0.80; 3.84]	2.13%
X. Nie, 2025	115	232	15	42	1.77	[0.89; 3.50]	2.36%
Z. Zheng, 2022	61	131	11	34	1.82	[0.82; 4.04]	2.11%
N. Yoshida, 2017	153	332	13	41	1.84	[0.92; 3.68]	2.34%
K. Kouzu, 2020	46	113	4	15	1.89	[0.57; 6.30]	1.40%
P. Y. Wang, 2021	51	131	15	61	1.96	[0.99; 3.86]	2.37%
R. Haneda, 2022	64	140	5	18	2.19	[0.74; 6.47]	1.58%
Q. Qi, 2021	113	324	15	83	2.43	[1.33; 4.44]	2.54%
I. Yamana, 2015	26	101	2	21	3.29	[0.72; 15.11]	1.03%
T. Toyokawa, 2016	17	152	0	33	8.65	[0.51; 147.58]	0.38%
**Random effects model**	**3720**	**10510**	**676**	**2349**	**1.26**	**[1.11; 1.44]**	**72.79%**
**Prediction interval**						**[0.90; 1.77]**	
Heterogeneity: $I^2 = 17\%$ [0%; 46%], $\tau = 0.1533$							
Test for effect in subgroup: $t_{35} = 3.60$ ($p < 0.001$)							
**Random effects model**	**4553**	**15279**	**1432**	**4712**	**1.09**	**[0.90; 1.31]**	**100.00%**
**Prediction interval**						**[0.39; 3.04]**	
Heterogeneity: $I^2 = 90\%$ [88%; 92%], $\tau = 0.5027$ [0.3884; 0.6810]							
Test for overall effect: $t_{47} = 0.88$ ($p = 0.383$)							
Test for subgroup differences: $\chi^2_1 = 2.17$, df = 1 ($p = 0.141$)							

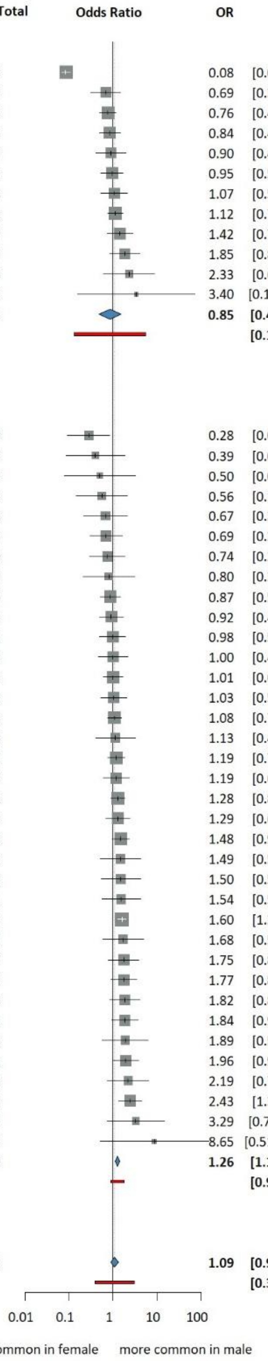

Abbreviation: R= resectable; NR= non-resectable

Figure S18.: Association between malnutrition-related complication risk and sex in gastric cancer (Biological composite scores)

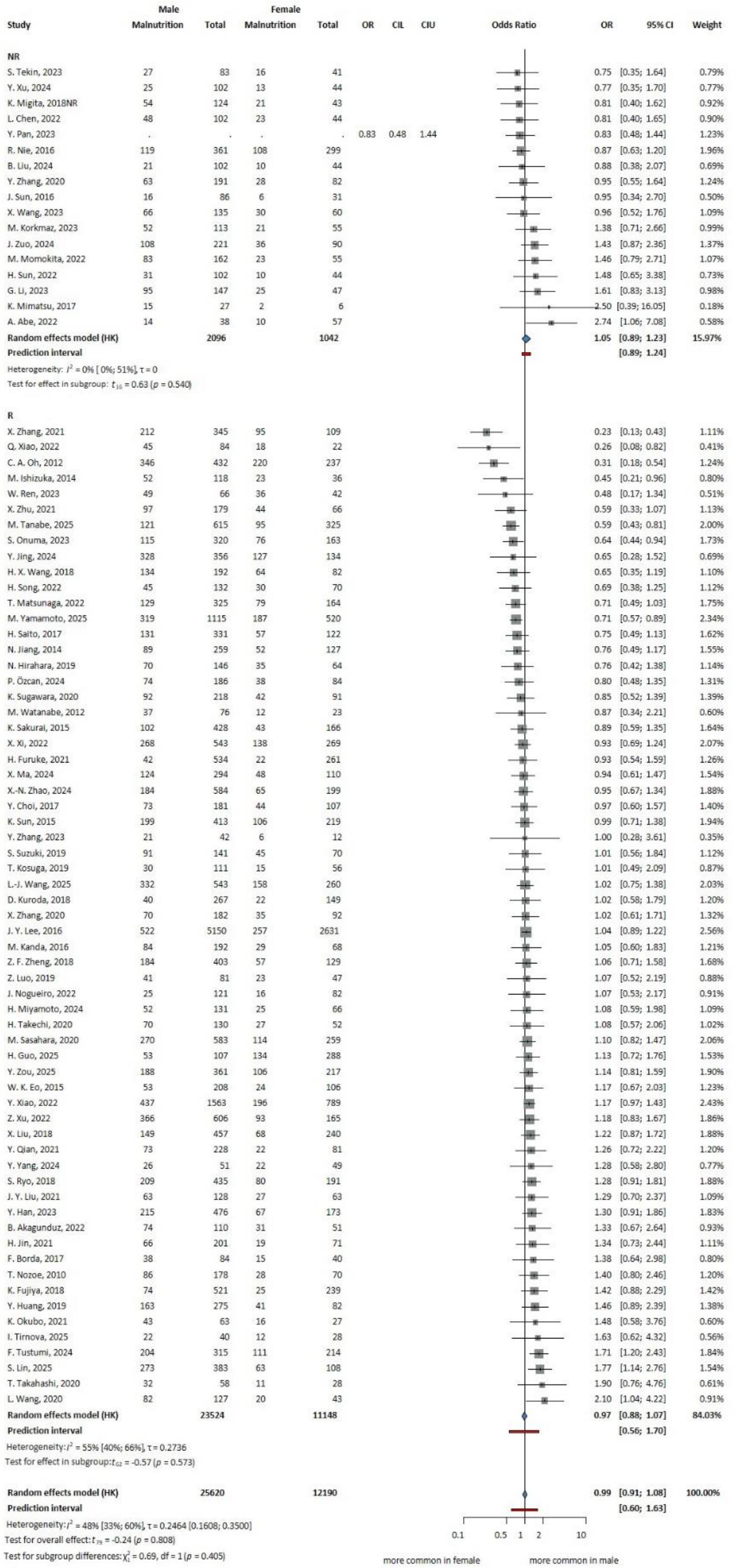

Abbreviation: R= resectable; NR= non-resectable

Figure S19.: Association between malnutrition-related complication risk and sex in colorectal cancer (Biological composite scores)

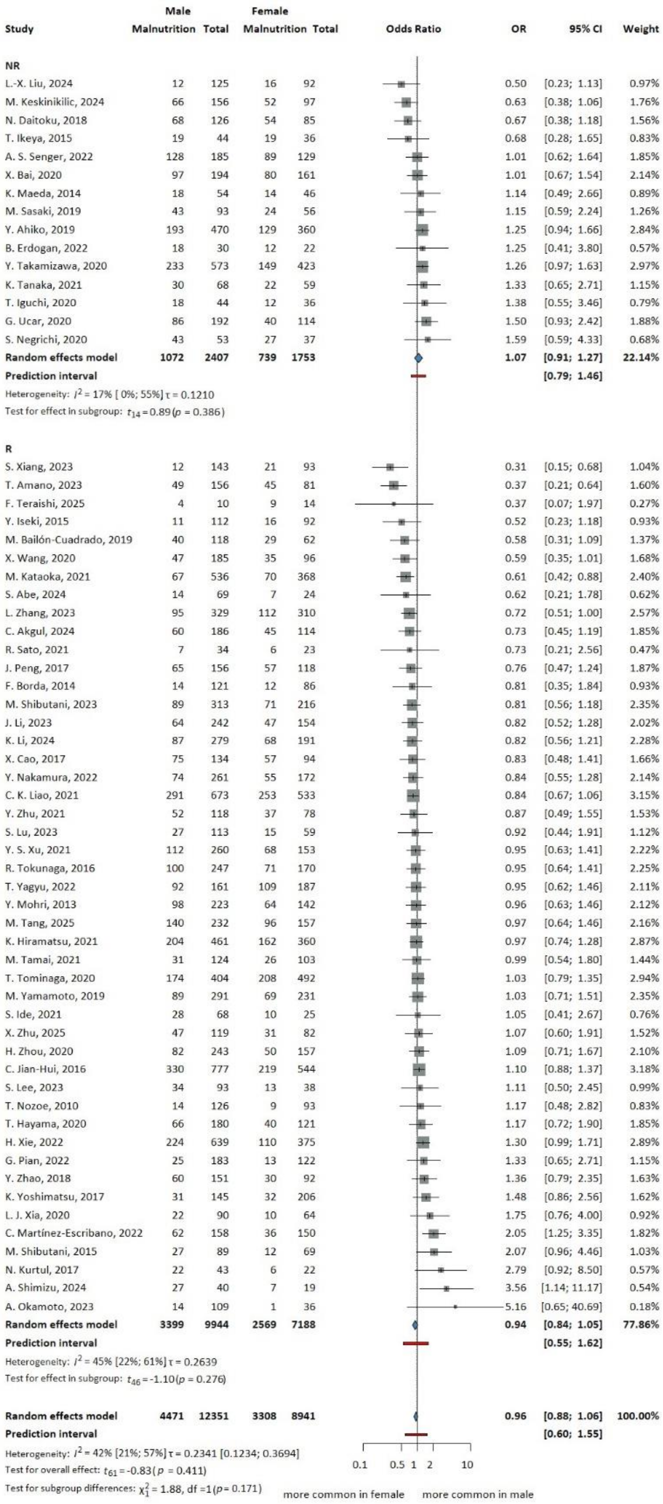

Abbreviation: R= resectable; NR= non-resectable

Figure S20.: Association between malnutrition-related complication risk and sex in biliary tract cancer (Biological composite scores)

Study	Male Malnutrition	Male Total	Female Malnutrition	Female Total	OR	95% CI	Weight
S. Wang, 2025	57	74	67	83	0.80	[0.37; 1.73]	6.14%
T. Matsuda, 2021	100	187	74	129	0.85	[0.54; 1.34]	17.75%
Í.D. Onur, 2024	10	27	16	41	0.92	[0.34; 2.50]	3.61%
S. Ikuta, 2023	75	118	60	95	1.02	[0.58; 1.78]	11.54%
L. Sun, 2021	93	199	77	172	1.08	[0.72; 1.63]	21.61%
D. Ogawa, 2023	40	82	19	41	1.10	[0.52; 2.34]	6.43%
F. Terasaki, 2020	29	115	7	34	1.30	[0.51; 3.30]	4.18%
J. Wang, 2021	86	201	82	229	1.34	[0.91; 1.98]	24.01%
A. Wang, 2021	19	49	12	45	1.74	[0.73; 4.18]	4.73%
**Random effects model**	**509**	**1052**	**414**	**869**	**1.09**	**[0.93; 1.29]**	**100.00%**
**Prediction interval**						**[0.87; 1.37]**	

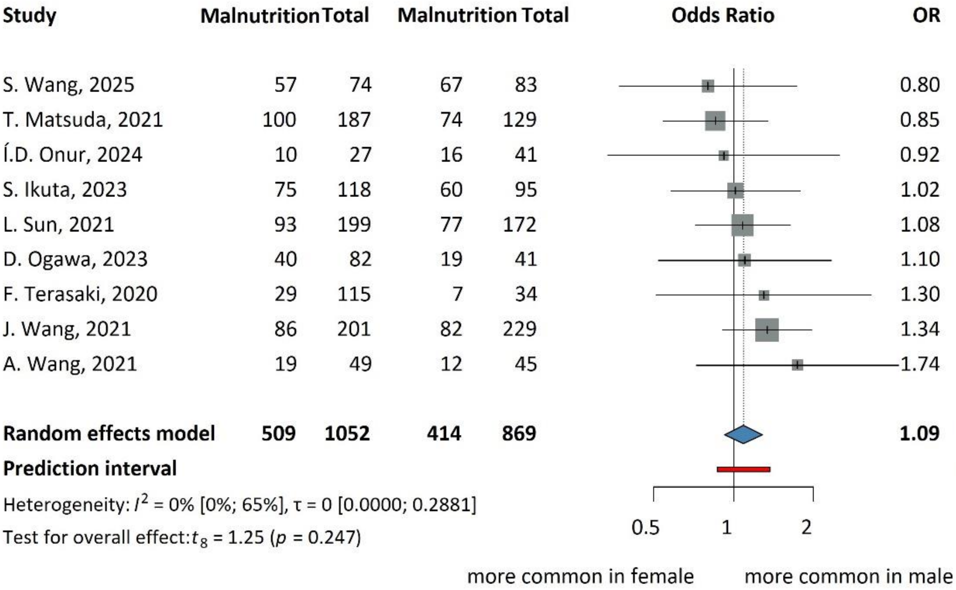

Heterogeneity: $I^2 = 0\%$ [0%; 65%], $\tau = 0$ [0.0000; 0.2881]

Test for overall effect: $t_8 = 1.25$ ($p = 0.247$)

Figure S21.: Association between malnutrition-related complication risk and sex in hepatocellular carcinoma (Biological composite scores)

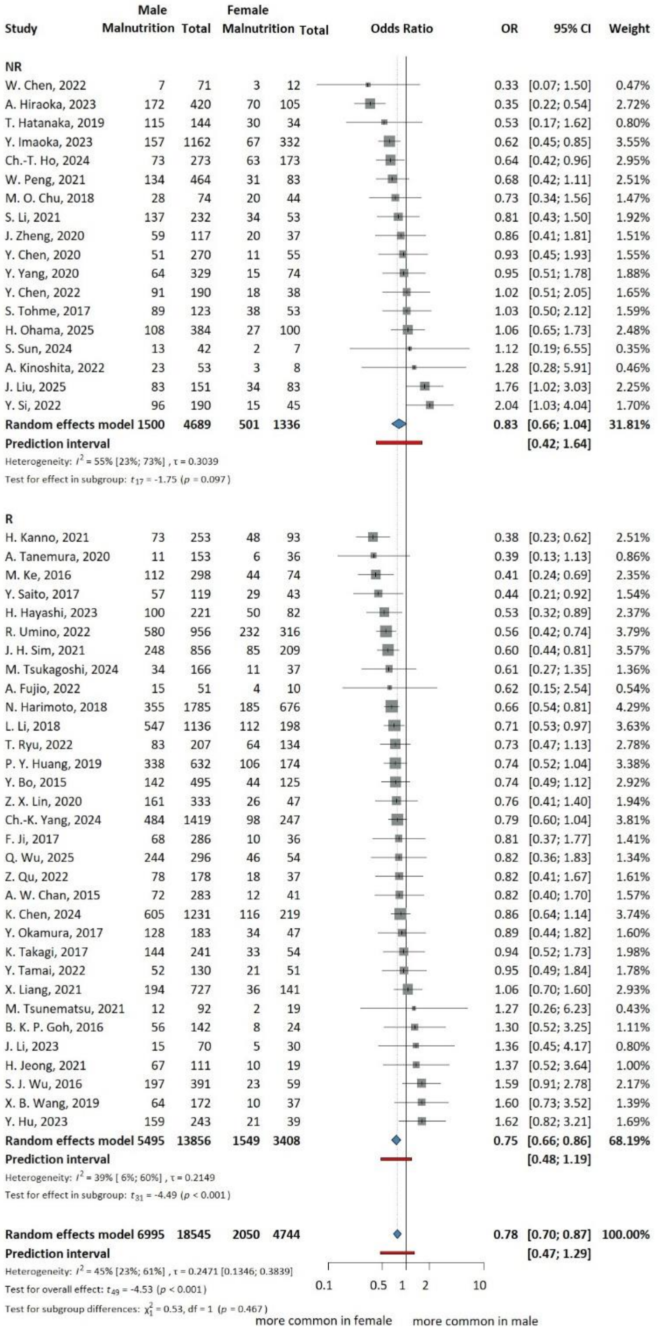

Abbreviation: R= resectable; NR= non-resectable

Figure S22.: Association between malnutrition-related complication risk and sex in Vater papilla cancer (Biological composite scores)

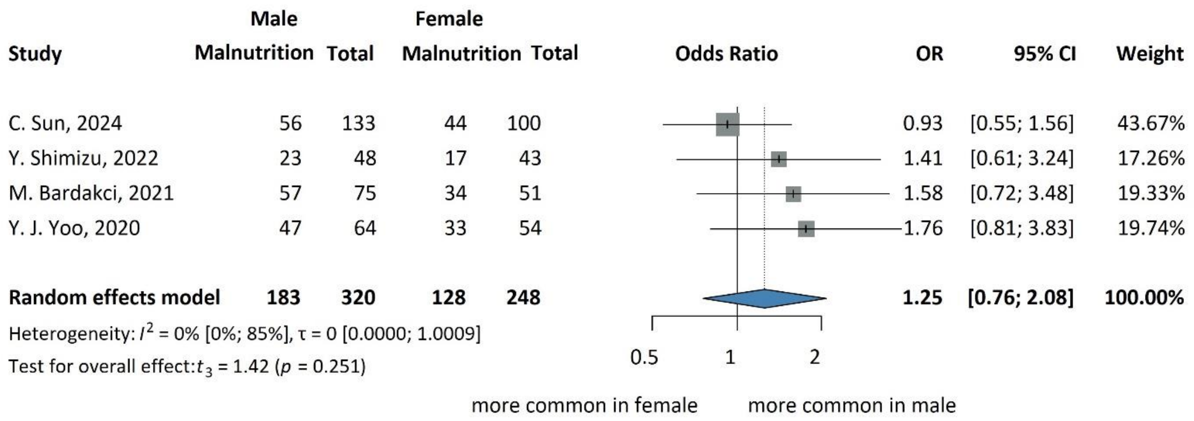

Figure S23.: Association between malnutrition-related complication risk and sex in pancreatic ductal adenocarcinoma (Biological composite scores)

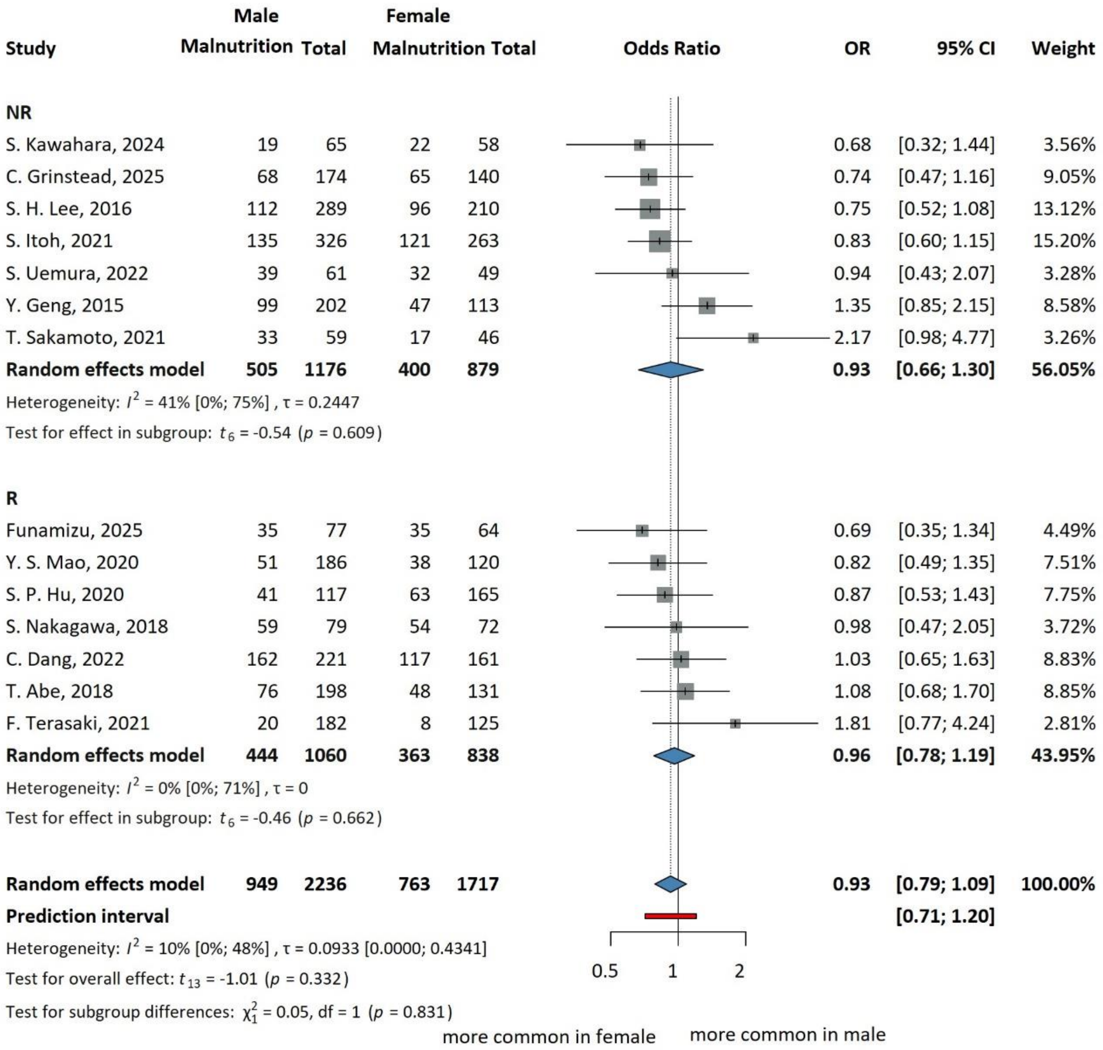

Abbreviation: R= resectable; NR= non-resectable

Figure S24.: Association between malnutrition diagnosis and sex in esophageal cancer (guidelines)

Study	Male Malnutrition	Male Total	Female Malnutrition	Female Total	OR	95% CI	Weight
L. Yin, 2021	32	291	12	69	0.59	[0.28; 1.21]	22.13%
J. Cao, 2021	311	1205	73	272	0.95	[0.70; 1.28]	33.74%
P. Y. Wang, 2021	38	131	12	61	1.67	[0.80; 3.48]	21.81%
X. H. Hua, 2022	42	146	12	73	2.05	[1.00; 4.20]	22.32%
**Random effects model**	**423**	**1773**	**109**	**475**	**1.15**	**[0.49; 2.70]**	**100.00%**
**Prediction interval**						**[0.13; 10.49]**	

Heterogeneity: I^2 = 62% [0%; 87%], τ^2 = 0.19 p = 0.050

Test for overall effect: t_3 = 0.51 (p = 0.648)

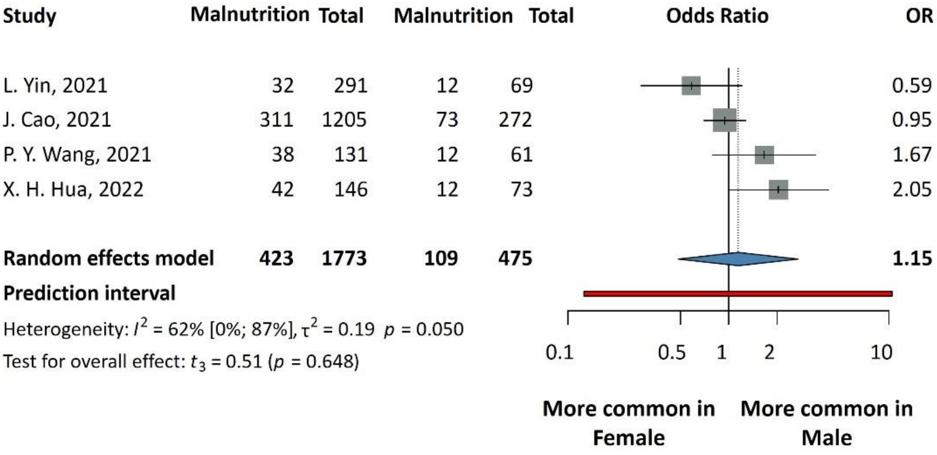

Figure S25.: Association between malnutrition diagnosis and sex in gastric cancer (guidelines)

Study	Male Malnutrition	Male Total	Female Malnutrition	Female Total	OR	95% CI	Weight
J. H. Park, 2021	65	794	87	487	0.41	[0.29; 0.58]	33.68%
Y. Fukuda, 2015	100	563	52	237	0.77	[0.53; 1.12]	32.64%
X. Zhao, 2022	184	584	65	199	0.95	[0.67; 1.34]	33.68%
**Random effects model**	**349**	**1941**	**204**	**923**	**0.67**	**[0.22; 1.98]**	**100.00%**
**Prediction interval**						**[0.00; 270.62]**	

Heterogeneity: I^2 = 84% [51%; 95%], τ^2 = 0.16 p = 0.002

Test for overall effect: t_2 = -1.60(p= 0.251)

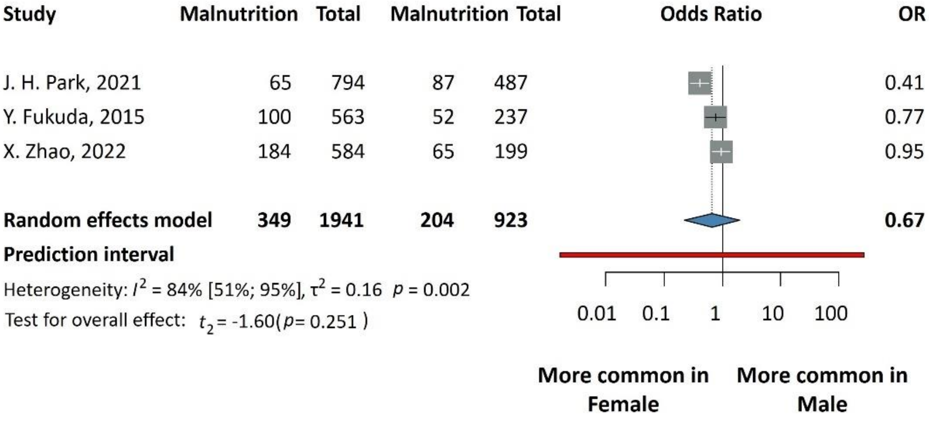

Figure S26.: Association between cachexia and sex in resectable gastrointestinal cancer

Study	Male Malnutrition	Male Total	Female Malnutrition	Female Total	OR	95% CI	Weight
J. Miller, 2020	3	11	3	5	0.25	[0.03; 2.32]	0.79%
N. Johns, 2014	11	30	7	11	0.33	[0.08; 1.39]	1.60%
N. A. Stephens, 2015	32	66	19	26	0.35	[0.13; 0.94]	2.59%
T. Nishhiyama, 2025	97	167	95	124	0.42	[0.25; 0.71]	4.41%
H. Tanda, 2025	54	177	59	121	0.46	[0.29; 0.74]	4.58%
N. I. P. Neto, 2018	5	10	4	6	0.50	[0.06; 4.09]	0.87%
T. Mracek, 2011	21	42	11	17	0.55	[0.17; 1.75]	2.14%
Q. Wan, 2022	131	234	100	145	0.57	[0.37; 0.89]	4.77%
C. L. Zhuang, 2022	210	886	112	329	0.60	[0.46; 0.79]	5.43%
L. R. Brown, 2024	78	277	40	108	0.67	[0.42; 1.07]	4.62%
M. C. Gül, 2025	102	214	48	87	0.74	[0.45; 1.22]	4.49%
L. R. Brown, 2025	178	311	97	154	0.79	[0.53; 1.17]	4.95%
K. Nakashima, 2022	67	119	33	56	0.90	[0.47; 1.71]	3.86%
X. Chen, 2019	153	433	53	142	0.92	[0.62; 1.36]	4.96%
Y. Jiang, 2014	19	61	20	65	1.02	[0.48; 2.17]	3.40%
Y. Du, 2025	575	826	186	275	1.10	[0.82; 1.47]	5.37%
T. Kamada, 2023	61	192	33	114	1.14	[0.69; 1.90]	4.46%
G.-T. Ruan, 2023	208	528	136	377	1.15	[0.88; 1.51]	5.44%
D. Sun, 2024	174	513	78	257	1.18	[0.85; 1.63]	5.25%
M. Akaoka, 2022	72	171	15	42	1.31	[0.65; 2.64]	3.62%
K. Sakashita, 2025	69	162	26	74	1.37	[0.77; 2.42]	4.18%
M. J. Alves, 2017	13	21	8	16	1.62	[0.44; 6.07]	1.81%
F. Tambaro, 2024	5	11	4	12	1.67	[0.31; 9.01]	1.25%
T. Shimagaki, 2025	47	80	22	48	1.68	[0.82; 3.46]	3.54%
A. Molfino, 2021	8	16	5	15	2.00	[0.47; 8.56]	1.57%
Y. Takano, 2023	146	232	75	164	2.01	[1.34; 3.03]	4.90%
R. Hamura, 2022	48	94	9	30	2.43	[1.01; 5.87]	2.95%
A. Fukuta, 2019	18	70	4	63	5.11	[1.62; 16.06]	2.18%
**Random effects model**	**2605**	**5954**	**1302**	**2883**	**0.94**	**[0.75; 1.17]**	**100.00%**
**Prediction interval**						**[0.37; 2.39]**	

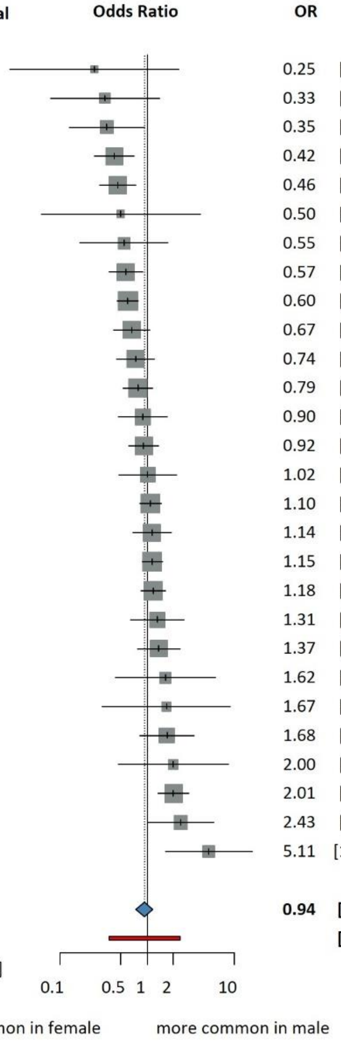

Heterogeneity: I^2 = 68% [52%; 78%], τ = 0.4429 [0.2673; 0.7329]
Test for overall effect: t_{27} = -0.59 (p = 0.559)

Figure S27.: Association between cachexia and sex in gastric cancer

Study	Male Malnutrition	Male Total	Female Malnutrition	Female Total	OR	95% CI	Weight
T. Matsunaga, 2024	25	71	17	31	0.45	[0.19; 1.06]	1.55%
C. L. Zhuang, 2022	210	886	112	329	0.60	[0.46; 0.79]	12.07%
B. Song, 2009	38	83	23	42	0.70	[0.33; 1.47]	2.04%
M. C. Gül, 2025	102	214	48	87	0.74	[0.45; 1.22]	4.32%
X. Zhang, 2025	1151	2178	560	980	0.84	[0.72; 0.98]	26.44%
F. Sun, 2010	70	150	37	73	0.85	[0.49; 1.49]	3.51%
K. Nakashima, 2022	67	119	33	56	0.90	[0.47; 1.71]	2.69%
X. Chen, 2019	153	433	53	142	0.92	[0.62; 1.36]	6.68%
Y. Huang, 2025	118	309	48	122	0.95	[0.62; 1.46]	5.70%
H. Fujii, 2020	13	23	12	21	0.97	[0.30; 3.22]	0.81%
C. Gong, 2022	109	217	54	107	0.99	[0.62; 1.57]	4.98%
Y. Jiang, 2014	19	61	20	65	1.02	[0.48; 2.17]	1.98%
D. Zhang, 2007	55	127	36	87	1.08	[0.62; 1.88]	3.60%
Y. Du, 2025	575	826	186	275	1.10	[0.82; 1.47]	10.96%
S. Bo, 2010	65	138	31	70	1.12	[0.63; 2.00]	3.30%
D. Sun, 2024	174	513	78	257	1.18	[0.85; 1.63]	9.36%
**Random effects model**	**2944**	**6348**	**1348**	**2744**	**0.88**	**[0.78; 0.99]**	**100.00%**
**Prediction interval**						**[0.72; 1.08]**	

Heterogeneity: I^2 = 16% [0%; 53%] , τ = 0.0745 [0.0000; 0.2623]

Test for overall effect: t_{15} = -2.27 (p = 0.039)

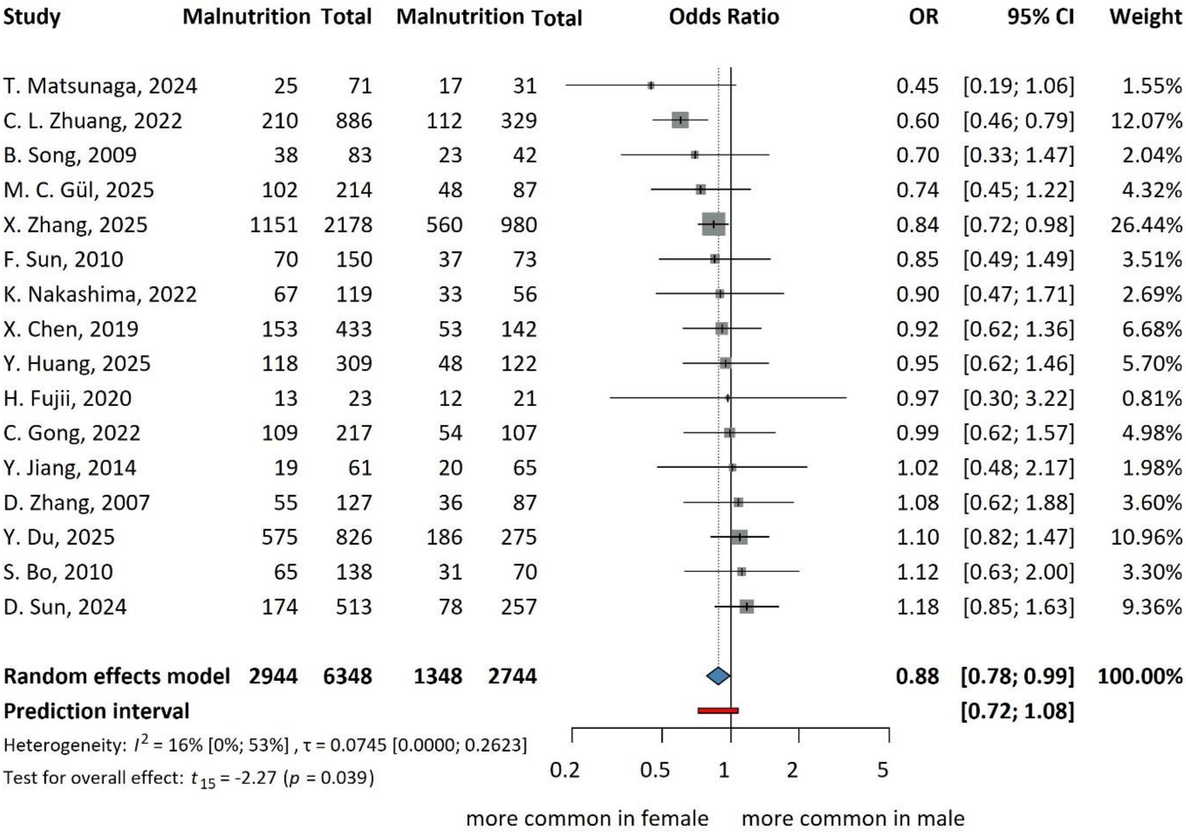

Figure S28.: Association between cachexia and sex in colorectal cancer

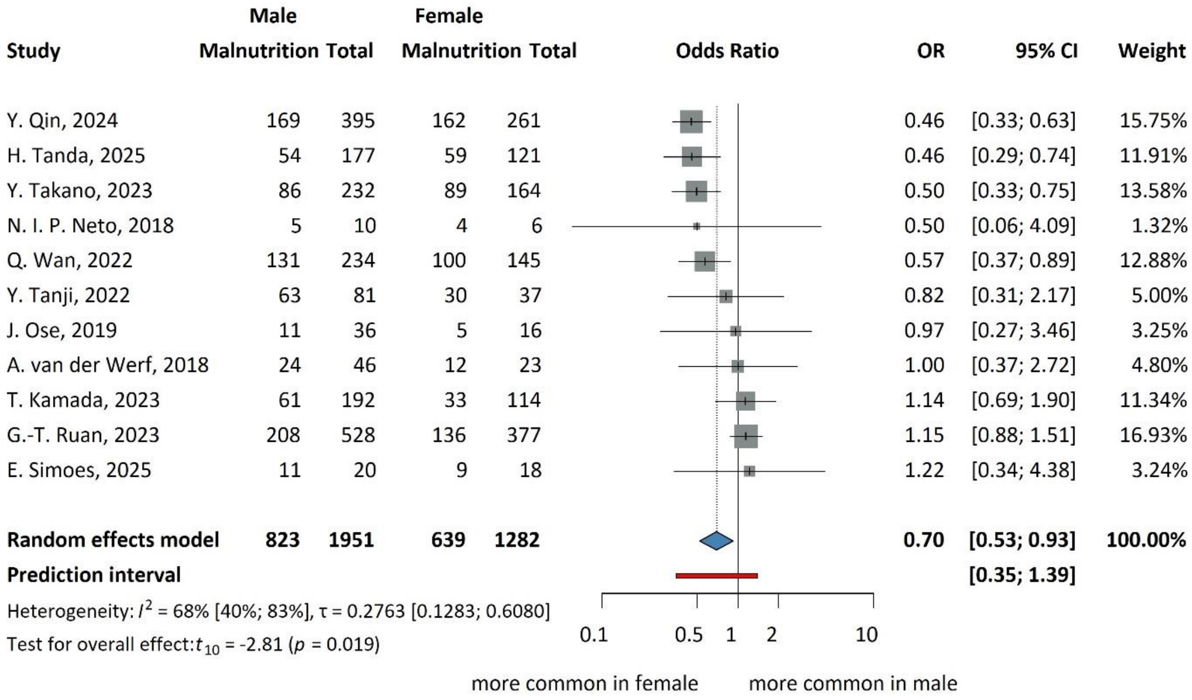

Figure S29.: Association between cachexia and sex in pancreatic ductal adenocarcinoma

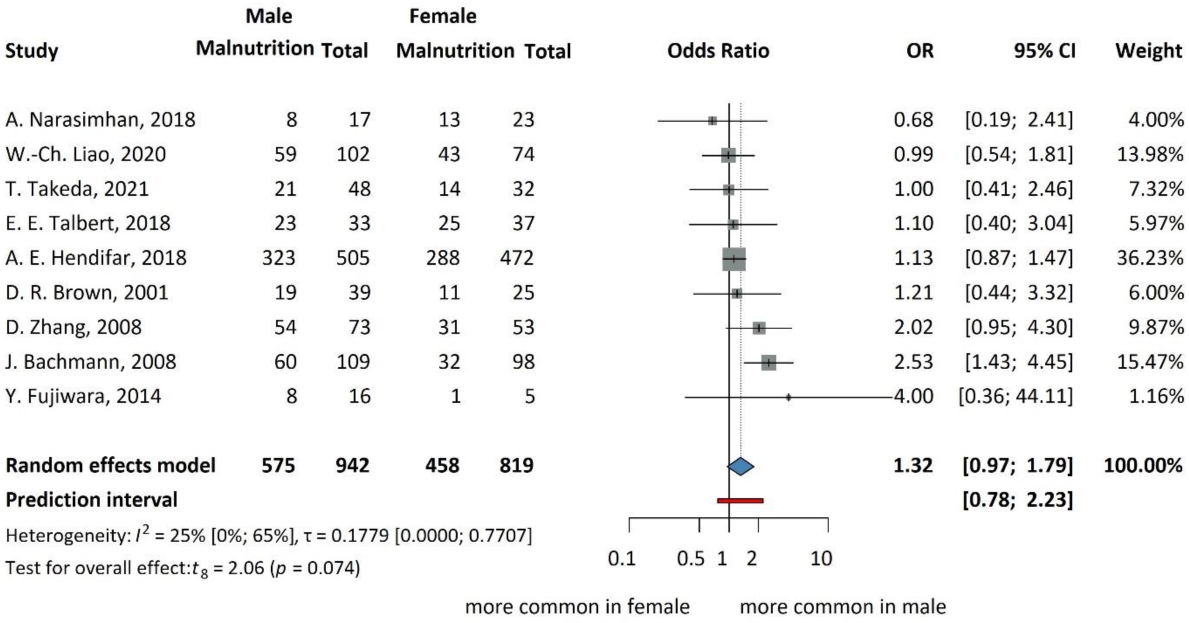

Figure S30.: Association between malnutrition risk and active smoking in gastrointestinal cancer (Symptom-based risk assessment tools)

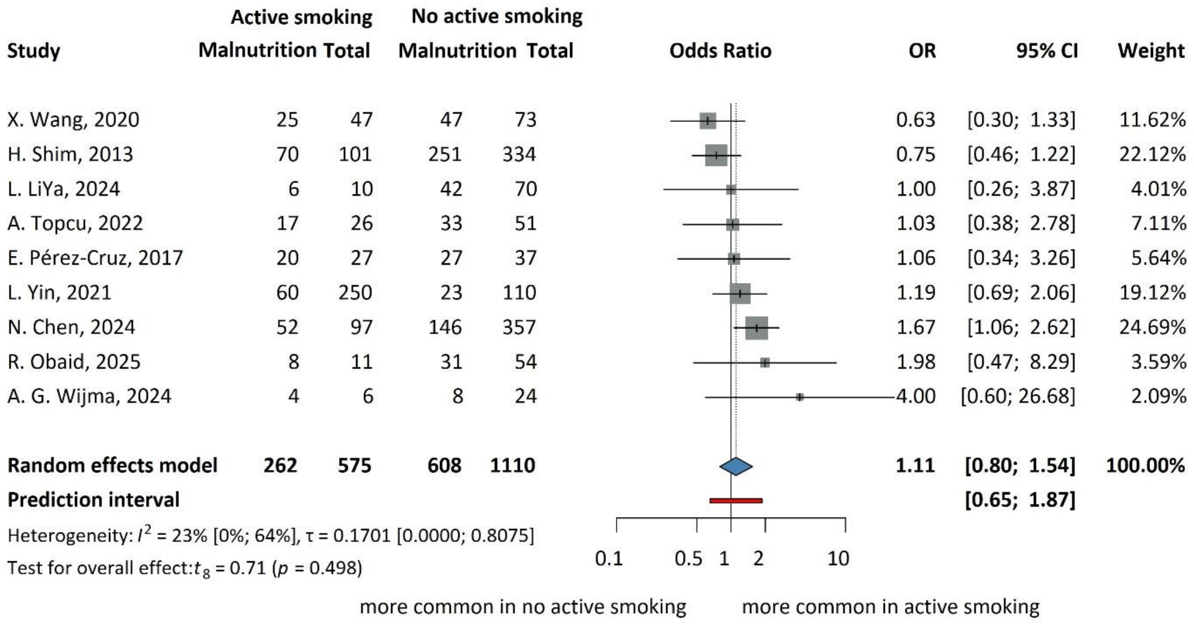

Figure S31.: Association between malnutrition-related complication risk and active smoking in esophageal cancer (Biological composite scores)

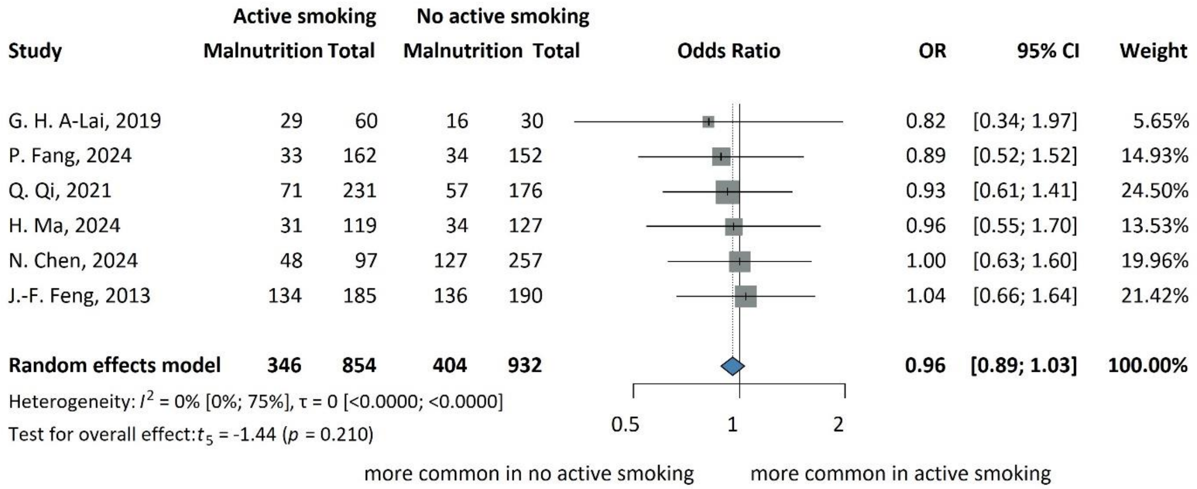

Figure S32.: Association between malnutrition-related complication risk and active smoking in gastric cancer (Biological composite scores)

Study	Active smoking Malnutrition	Active smoking Total	No active smoking Malnutrition	No active smoking Total	OR	95% CI	Weight
T. Kosuga, 2019	18	73	27	94	0.81	[0.41; 1.63]	3.79%
Y. Han, 2023	98	229	184	420	0.96	[0.69; 1.33]	17.33%
X. Ma, 2025	11	68	56	336	0.96	[0.48; 1.96]	3.67%
Z. Xu, 2022	221	373	238	398	0.98	[0.73; 1.30]	22.11%
X. Zhang, 2021	37	54	270	400	1.05	[0.57; 1.93]	4.90%
M. Momokita, 2022	61	106	61	111	1.11	[0.65; 1.90]	6.35%
A. Abe, 2022	14	53	10	42	1.15	[0.45; 2.93]	2.09%
X. Xi, 2022	188	360	218	452	1.17	[0.89; 1.55]	23.84%
Y. Zou, 2025	124	217	170	361	1.50	[1.07; 2.10]	15.92%
**Random effects model**	**772**	**1533**	**1234**	**2614**	**1.10**	**[0.96; 1.25]**	**100.00%**
**Prediction interval**						**[0.93; 1.29]**	

Heterogeneity: $I^2 = 0\%$ [0%; 65%], $\tau = 0$ [0.0000; 0.2359]

Test for overall effect: $t_8 = 1.61$ ($p = 0.147$)

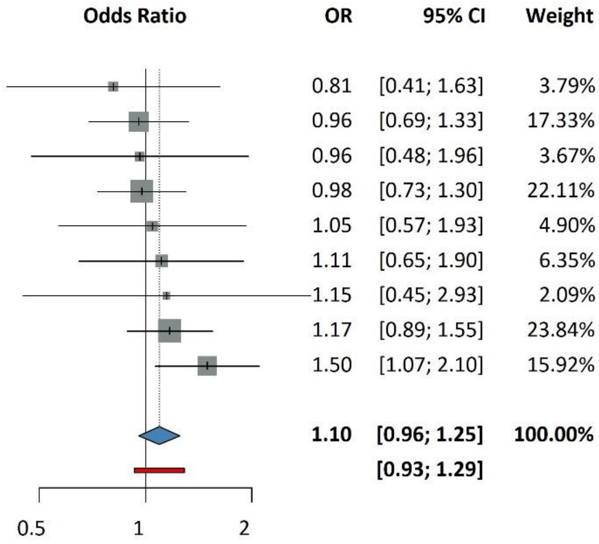

Figure S33.: Association between malnutrition-related complication risk and smoking (active or history) in esophageal cancer (Biological composite scores)

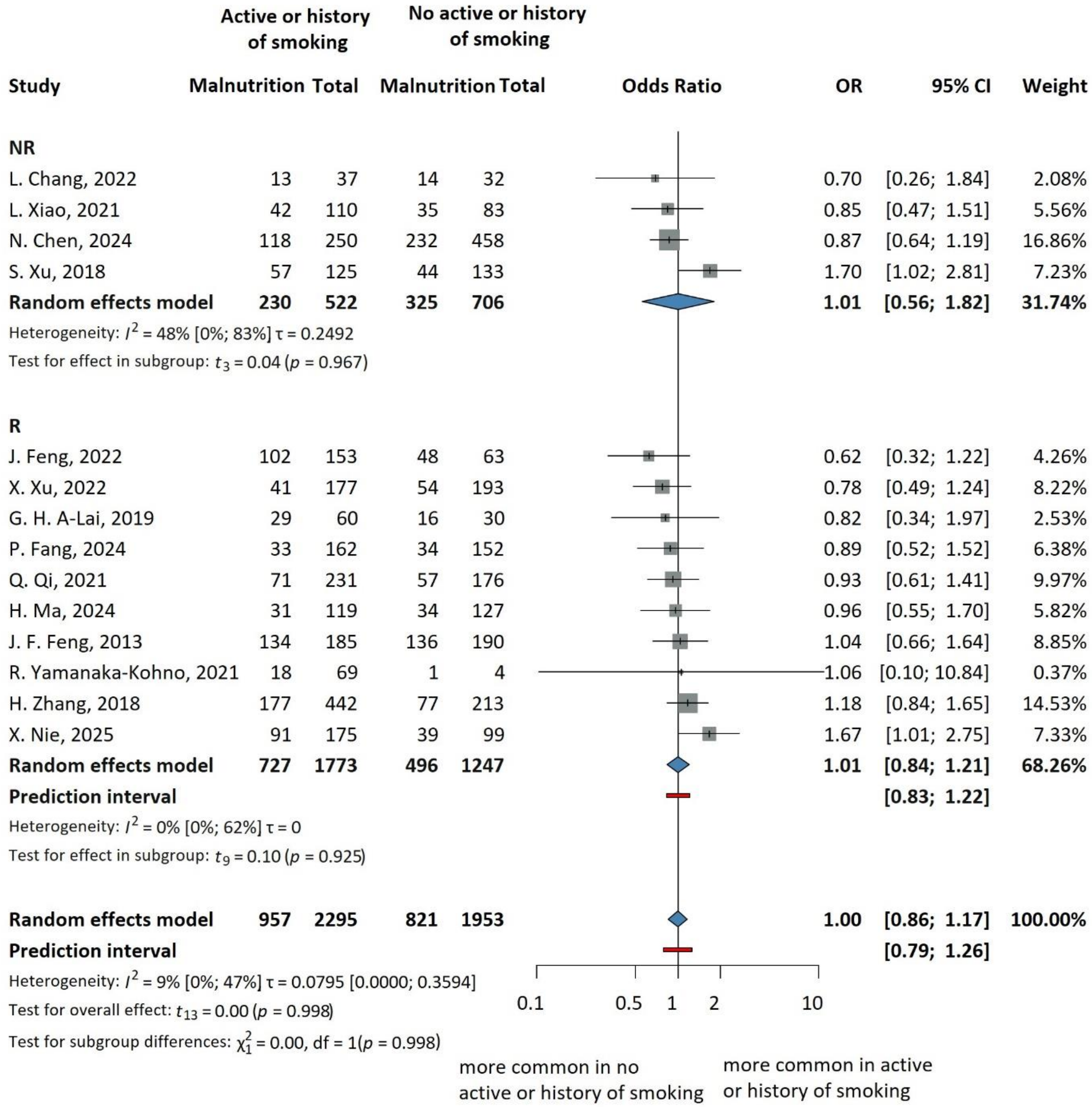

Abbreviation: R= resectable; NR= non-resectable

Figure S34.: Association between malnutrition-related complication risk and smoking (active or history) in colorectal cancer (Biological composite scores)

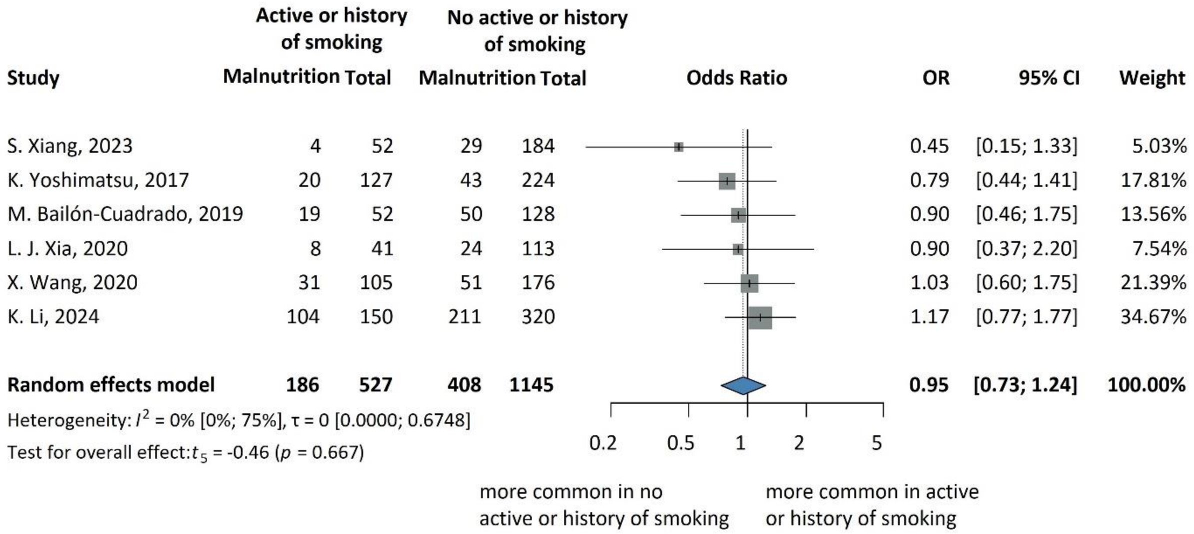

Figure S35.: Association between malnutrition-related complication risk and smoking (active or history) in hepato-biliopancreatic cancer (Biological composite scores)

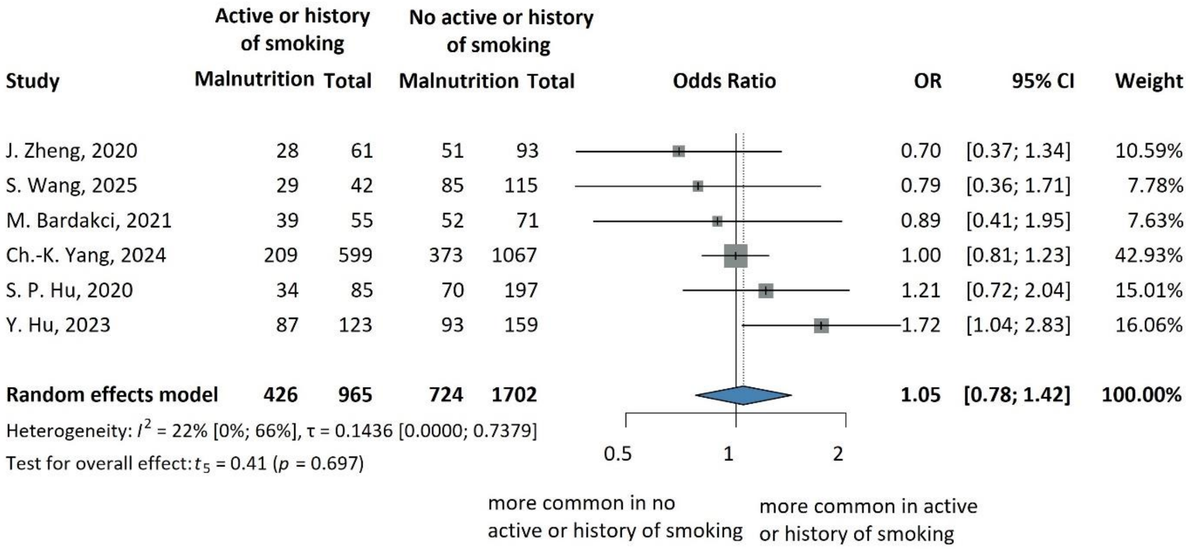

Figure S36.: Association between malnutrition diagnosis and smoking (active or history) in gastrointestinal cancer (guideline)

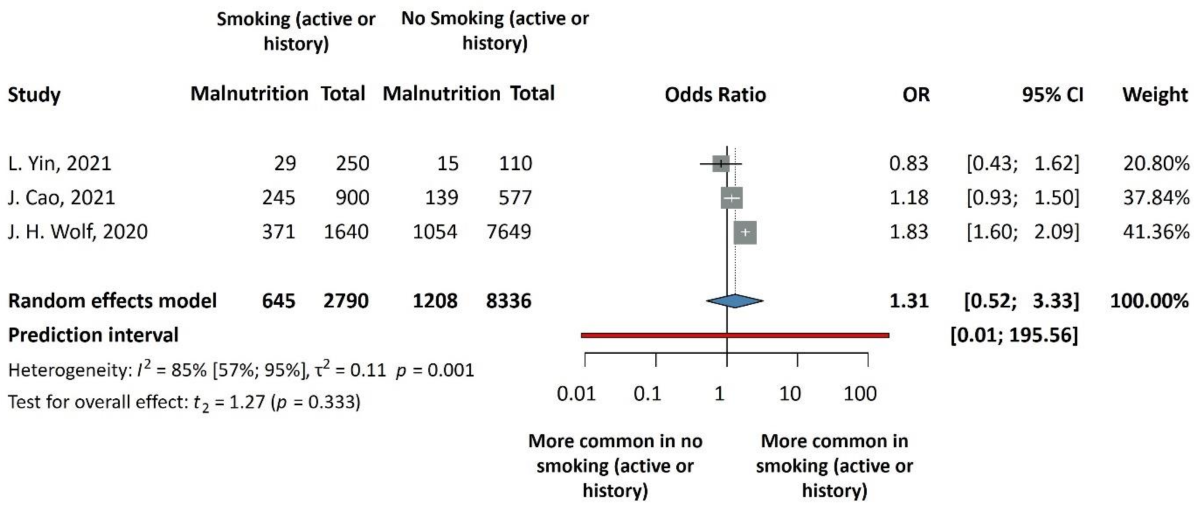

Figure S37.: Association between malnutrition-related complication risk and smoking history in esophageal and colorectal cancer (Biological composite scores)

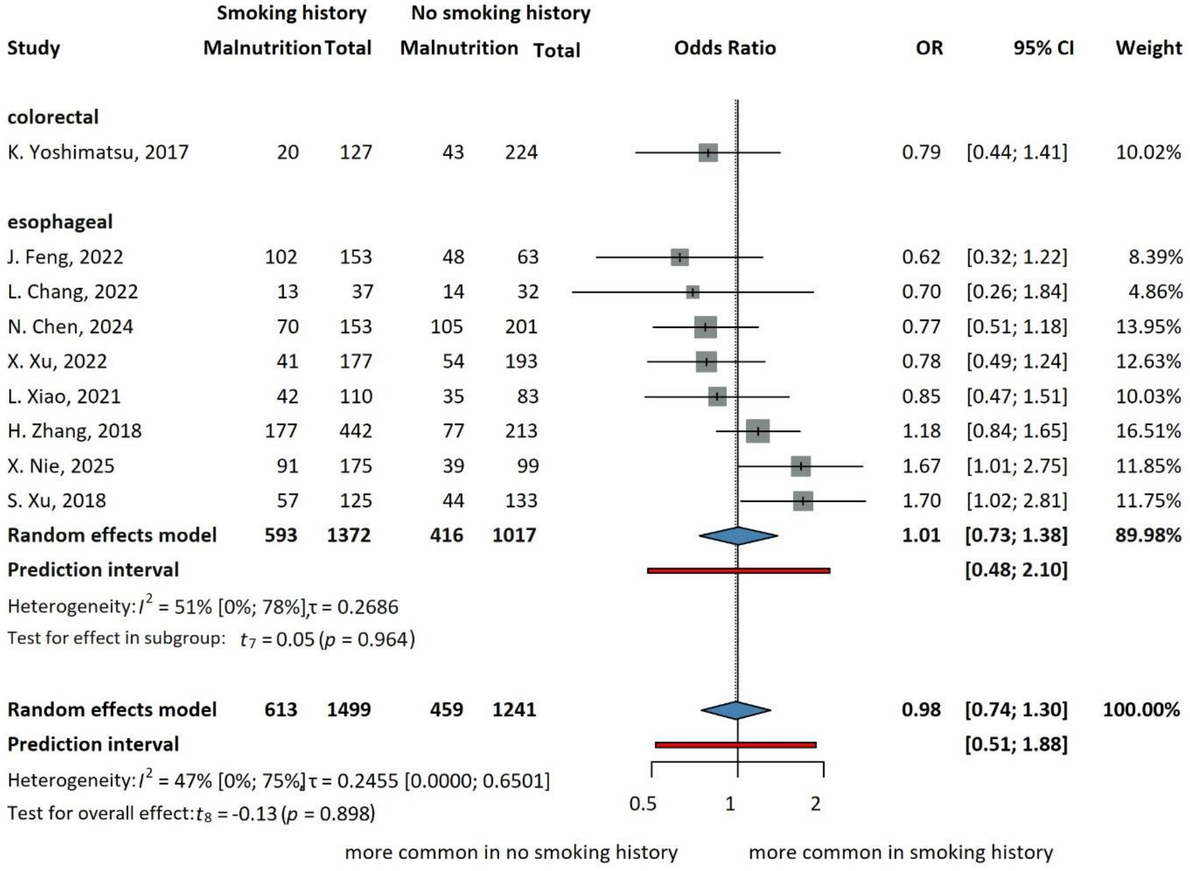

Figure S38.: Association between malnutrition risk and alcohol consumption (active) in gastrointestinal cancer (Symptom-based risk assessment tool)

Study	Active alcohol consumption: Malnutrition	Active alcohol consumption: Total	No active alcohol consumption: Malnutrition	No active alcohol consumption: Total	OR	95% CI	Weight
N. Chen, 2024	47	94	151	260	0.72	[0.45; 1.16]	24.97%
H. Shim, 2013	146	206	175	229	0.75	[0.49; 1.15]	30.52%
C. Zhang, 2021	98	147	69	102	0.96	[0.56; 1.64]	19.32%
Y.Kim, 2024	2	10	25	152	1.27	[0.25; 6.34]	2.17%
L. Yin, 2021	39	152	44	208	1.29	[0.79; 2.11]	23.02%
**Random effects model**	**332**	**609**	**464**	**951**	**0.89**	**[0.64; 1.23]**	**100.00%**

Heterogeneity: I^2 = 0% [0%; 79%], τ = 0 [0.0000; 0.6934]

Test for overall effect: t_4 = -0.98 (p = 0.384)

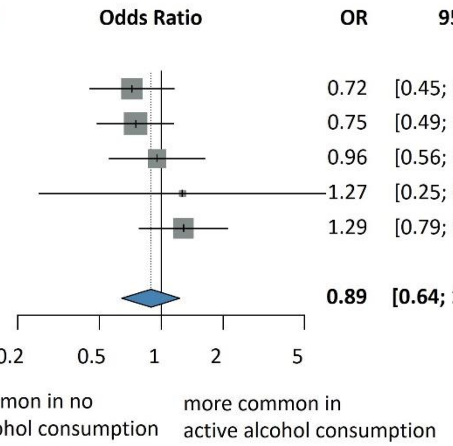

Figure S39.: Association between malnutrition-related complication risk and alcohol consumption (active) in gastrointestinal cancer (Biological composite scores)

Study	Active alcohol consumption: Malnutrition	Active alcohol consumption: Total	No active alcohol consumption: Malnutrition	No active alcohol consumption: Total	OR	95% CI	Weight
A. Abe, 2022	10	51	14	44	0.52	[0.20; 1.34]	1.87%
S. Xiang, 2023	4	46	29	190	0.53	[0.18; 1.59]	1.38%
R. Yamanaka-Kohno, 2021	16	65	3	8	0.54	[0.12; 2.53]	0.71%
J. F. Feng, 2013	95	141	175	234	0.70	[0.44; 1.10]	7.06%
K. Li, 2024	24	89	131	381	0.70	[0.42; 1.18]	5.79%
L. Chang, 2022	17	46	10	23	0.76	[0.28; 2.11]	1.60%
P. Y. Wang, 2021	15	48	51	144	0.83	[0.41; 1.67]	3.28%
Z. Xu, 2022	185	315	274	456	0.95	[0.71; 1.27]	14.73%
N. Chen, 2024	46	94	129	260	0.97	[0.61; 1.56]	6.74%
L. J. Xia, 2020	8	39	24	115	0.98	[0.40; 2.40]	2.04%
Y. Han, 2023	77	176	205	473	1.02	[0.72; 1.44]	11.21%
G. H. A-Lai, 2019	21	41	24	49	1.09	[0.48; 2.51]	2.37%
Q. Qi, 2021	74	229	54	178	1.10	[0.72; 1.67]	8.15%
K. Okadome, 2020	101	288	16	49	1.11	[0.58; 2.12]	3.83%
J. Liu, 2022	38	95	23	63	1.16	[0.60; 2.24]	3.69%
X. Ma, 2025	12	64	55	340	1.20	[0.60; 2.39]	3.36%
H. Ma, 2024	36	124	29	122	1.31	[0.74; 2.32]	4.81%
Y. Zou, 2025	120	210	174	368	1.49	[1.06; 2.09]	11.61%
S. Xu, 2018	47	98	54	160	1.81	[1.08; 3.03]	5.78%
**Random effects model**	**946**	**2259**	**1474**	**3657**	**1.03**	**[0.89; 1.18]**	**100.00%**
**Prediction interval**						**[0.81; 1.30]**	

Heterogeneity: I^2 = 11% [0%; 47%] τ = 0.0888 [0.0000; 0.3381]

Test for overall effect: t_{18} = 0.42 (p = 0.676)

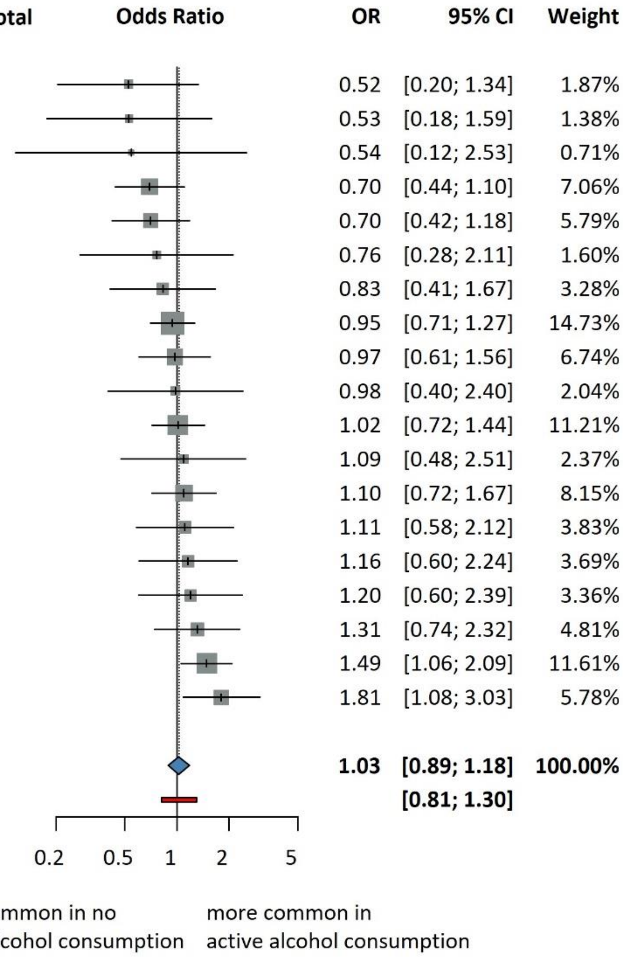

Figure S40.: Association between malnutrition-related complication risk and alcohol consumption (active) in esophageal and other gastrointestinal tract cancer (Biological composite scores)

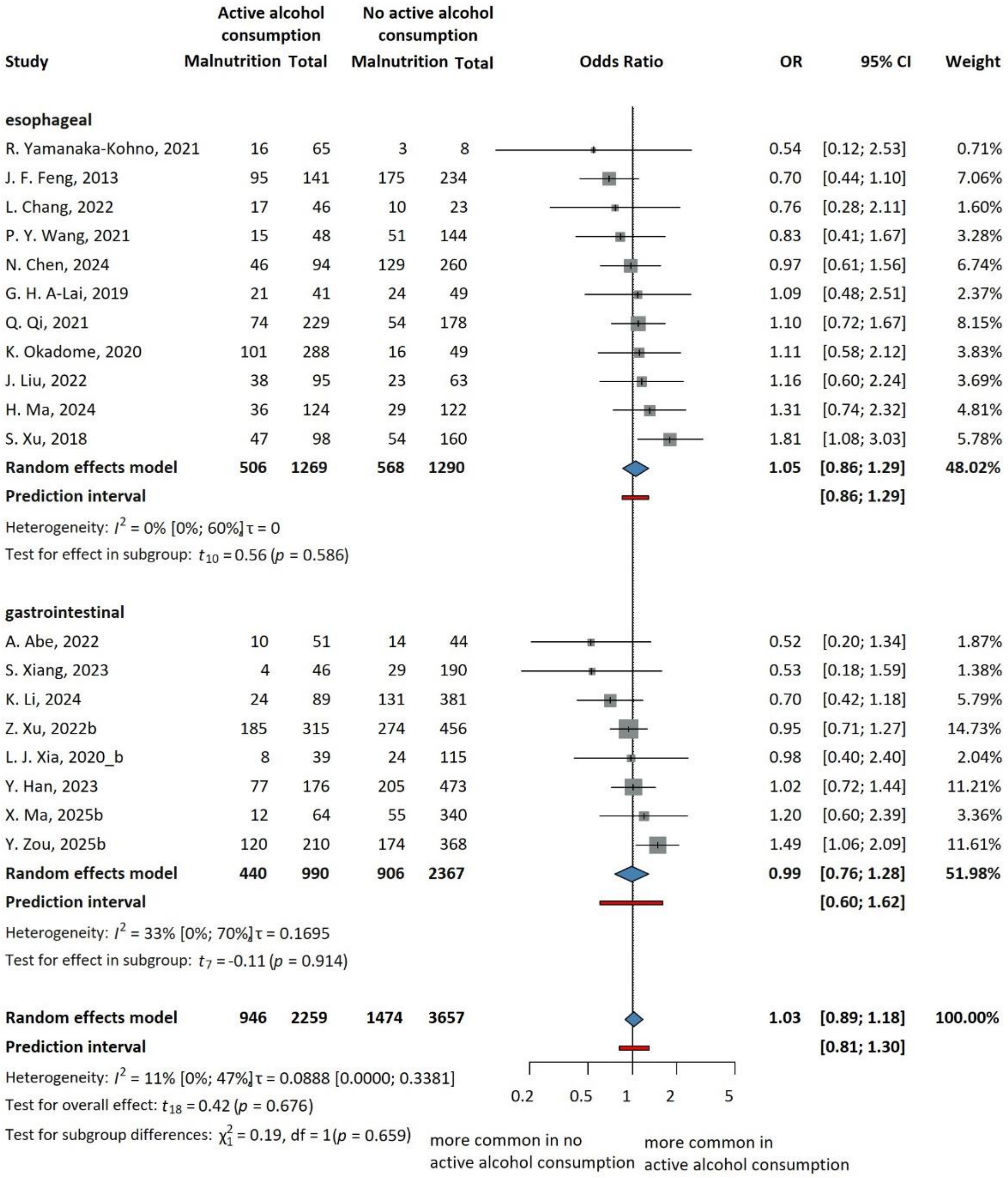

Figure S41.: Association between malnutrition-related complication risk and alcohol consumption (active) in hepatobiliopancreatic cancer (Biological composite scores)

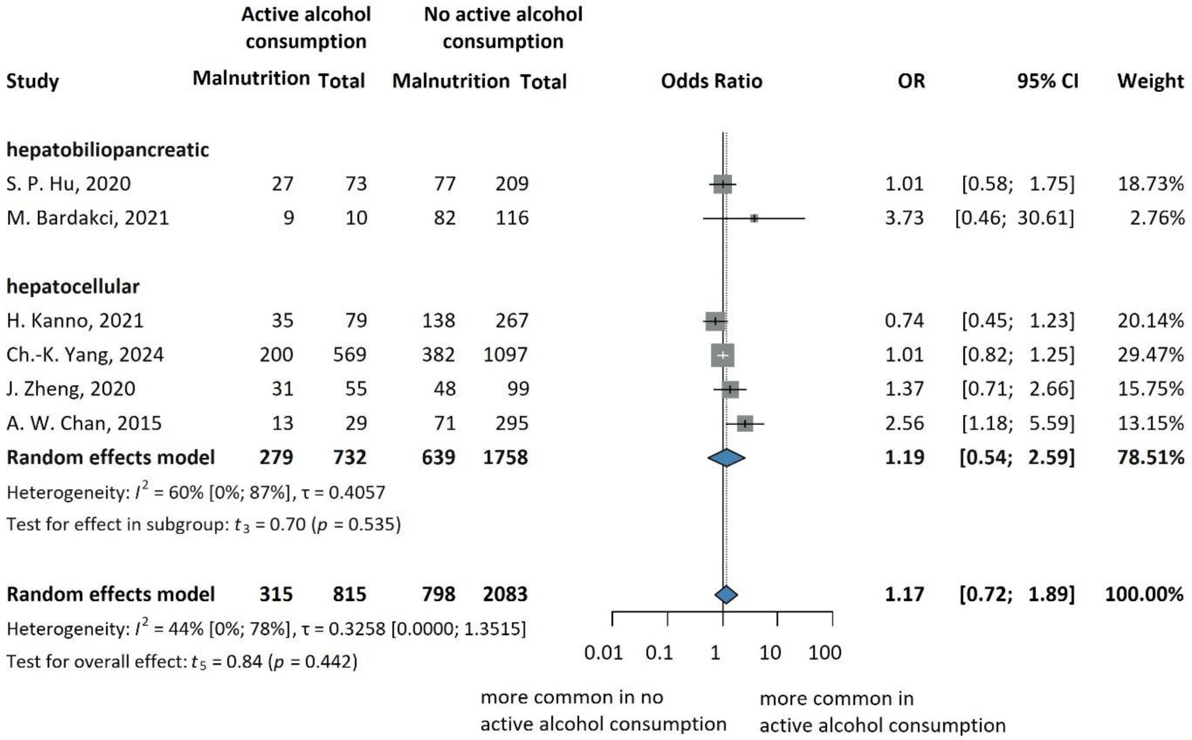

Figure S42.: Association between malnutrition-related complication risk and alcohol consumption (active) in hepato-biliopancreatic cancer [subgroup analysis for hepatocellular carcinoma] (Biological composite scores)

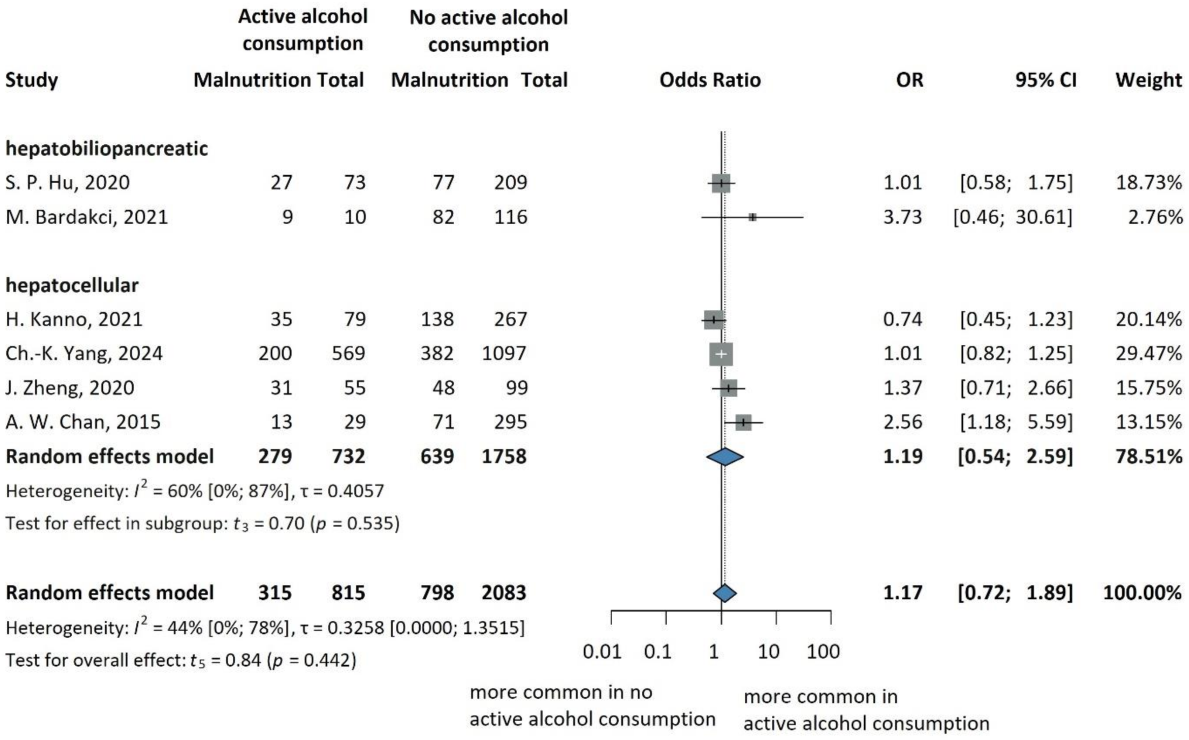

Figure S43.: Association between malnutrition diagnosis and alcohol consumption (active) in esophageal cancer (guideline)

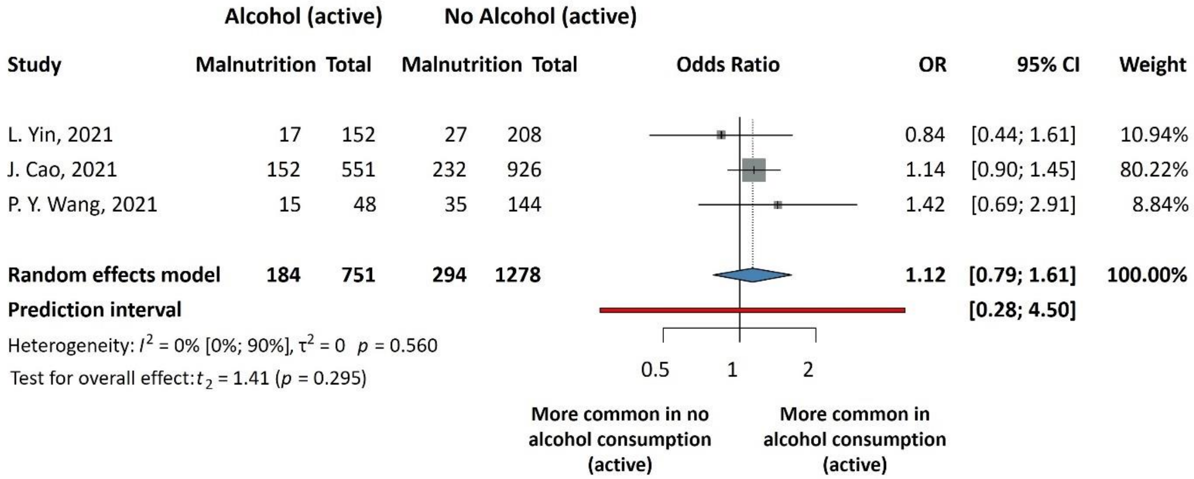

Figure S44.: Association between cachexia and alcohol consumption (active) in gastrointestinal cancer

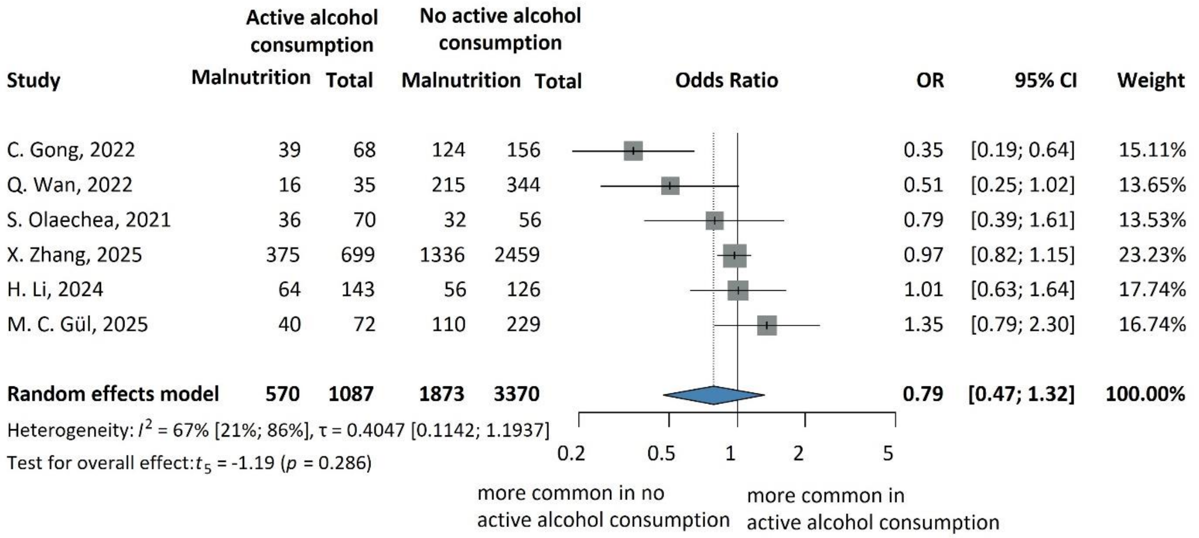

Figure S45.: Association between malnutrition-related complication risk and alcohol consumption (active and history) in hepatobilio-pancreatic cancer [subgroup analysis for hepatocellular cancer] (Biological composite scores)

	History or active alcohol consumption		No history or active alcohol consumption				
**Study**	**Malnutrition**	**Total**	**Malnutrition**	**Total**	**OR**	**95% CI**	**Weight**
**hepatobiliopancreatic**							
S. P. Hu, 2020	27	73	77	209	1.01	[0.58; 1.75]	15.68%
M. Bardakci, 2021	9	10	82	116	3.73	[0.46; 30.61]	3.64%
**hepatocellular**							
H. Kanno, 2021	35	79	138	267	0.74	[0.45; 1.23]	16.33%
Y. Hu, 2023	45	72	135	210	0.93	[0.53; 1.61]	15.65%
Ch.-K. Yang, 2024	200	569	382	1097	1.01	[0.82; 1.25]	19.79%
J. Zheng, 2020	31	55	48	99	1.37	[0.71; 2.66]	14.15%
A. W. Chan, 2015	13	29	71	295	2.56	[1.18; 5.59]	12.62%
W. Chen, 2022	10	46	0	37	21.58	[1.22; 381.82]	2.13%
**Random effects model**	**334**	**850**	**774**	**2005**	**1.29**	**[0.59; 2.81]**	**80.68%**
Heterogeneity: I^2 = 58% [0%; 83%], τ = 0.6301							
Test for effect in subgroup: t_5 = 0.84 (p = 0.441)							
**Random effects model**	**370**	**933**	**933**	**2330**	**1.25**	**[0.73; 2.13]**	**100.00%**
**Prediction interval**						**[0.33; 4.73]**	
Heterogeneity: I^2 = 48% [0%; 77%], τ = 0.4957 [0.0000; 1.9395]							
Test for overall effect: t_7 = 0.98 (p = 0.362)							

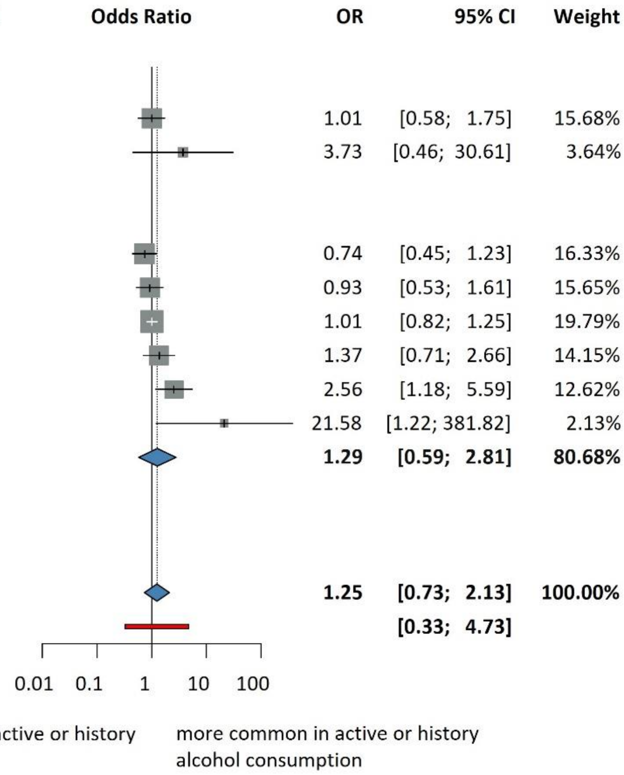

Figure S46.: Association between malnutrition-related complication risk and alcohol consumption (history) in esophageal cancer (Biological composite scores)

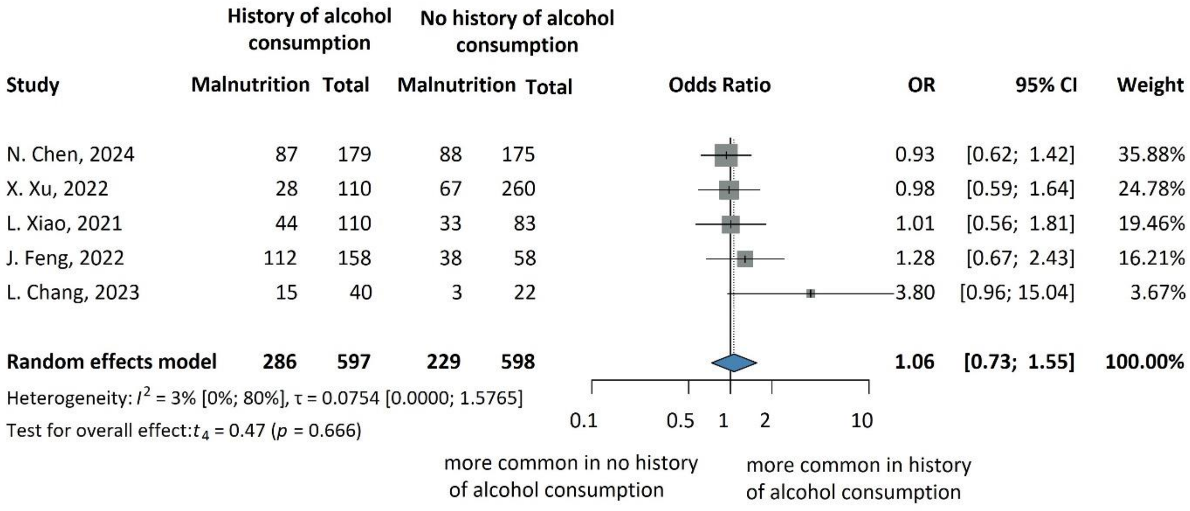

Figure S47.: Association between malnutrition-related complication risk and comorbidities in esophageal and gastric cancer (Biological composite scores)

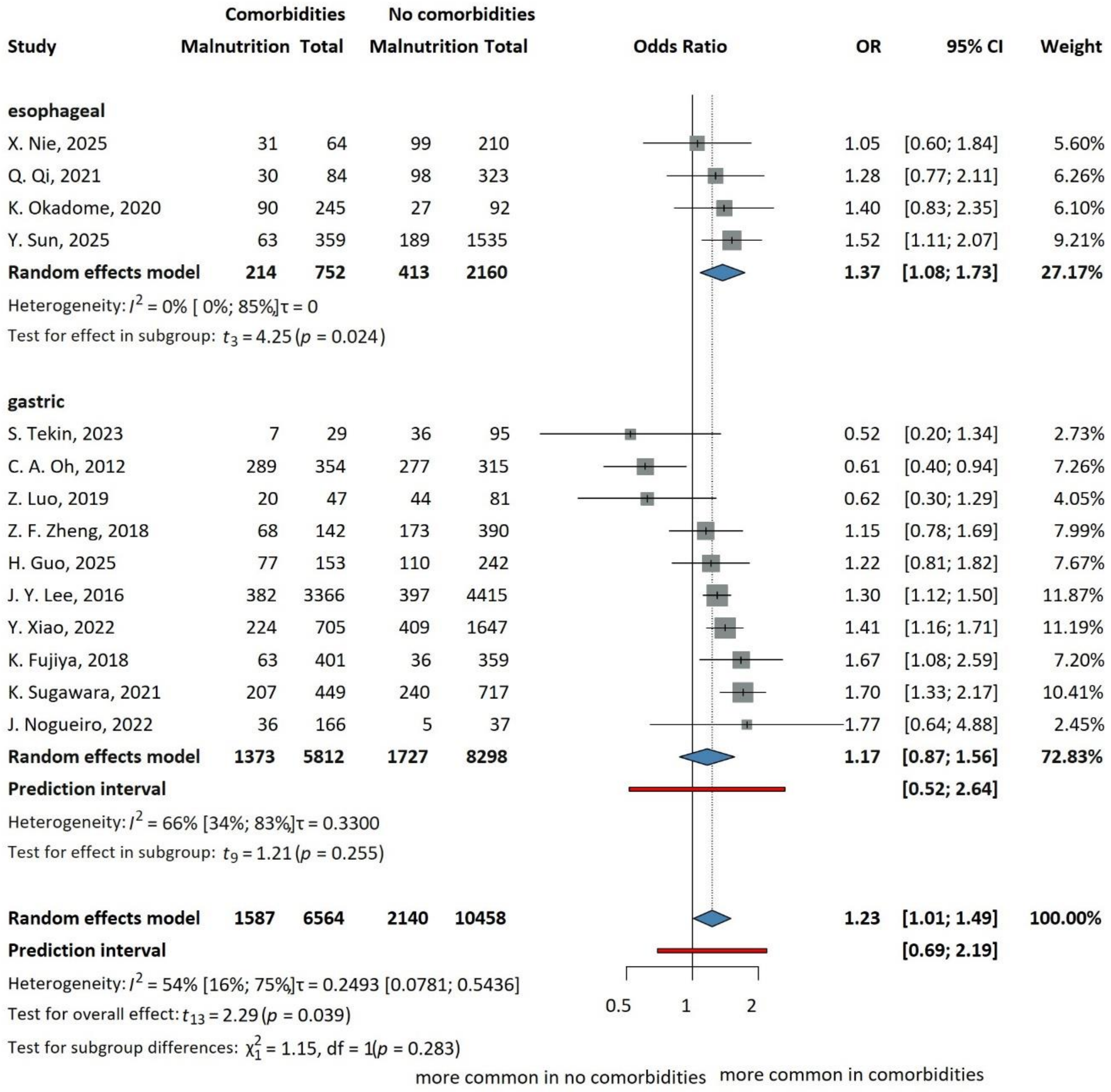

Figure S48.: Association between malnutrition-related complication risk and cardiovascular disease in gastric cancer (Biological composite scores)

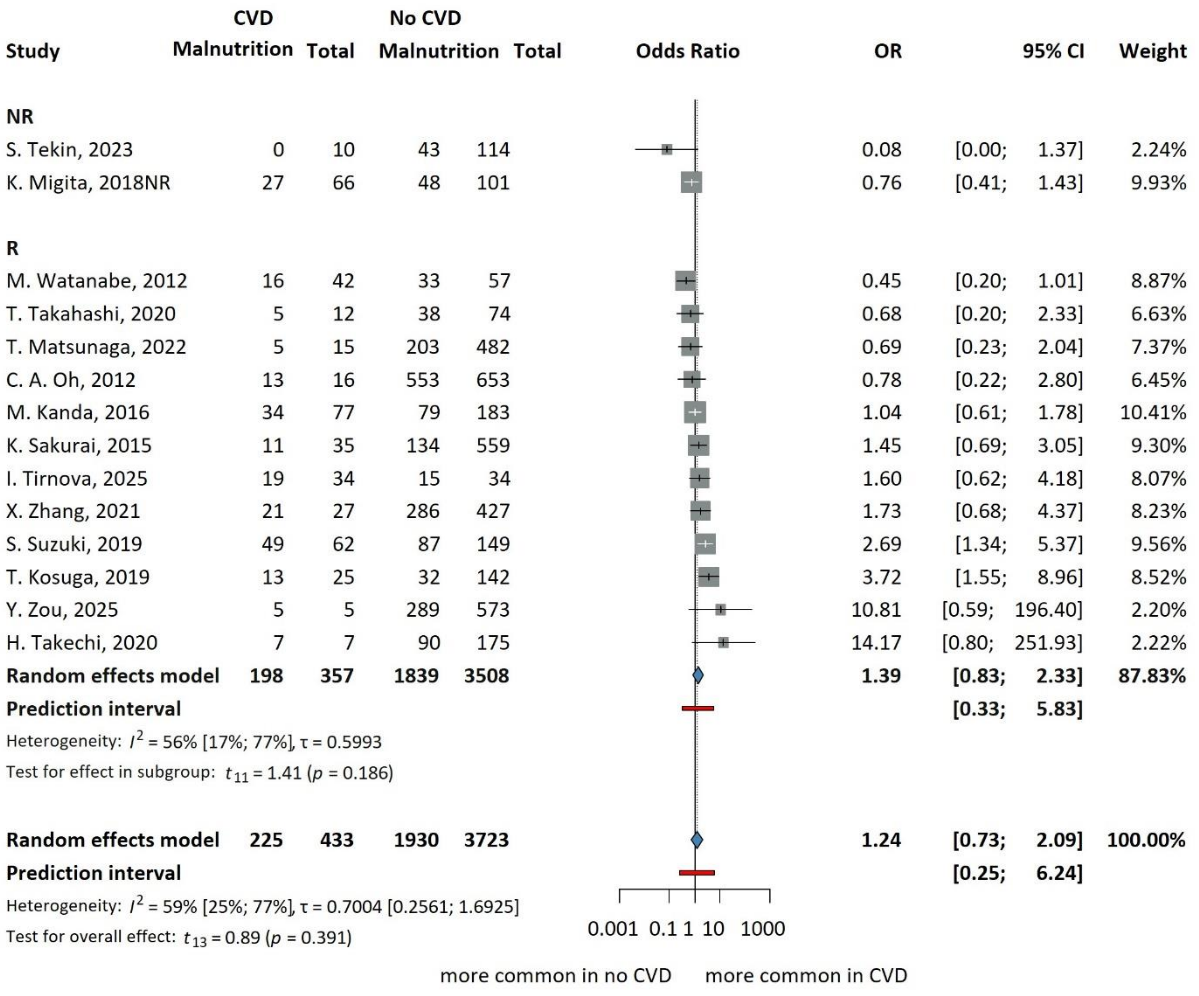

Abbreviation: R= resectable; NR= non-resectable; CVD= cardiovascular diseases

Figure S49.: Association between malnutrition-related complication risk and cardiovascular disease in hepatobilio-pancreatic cancer (Biological composite scores)

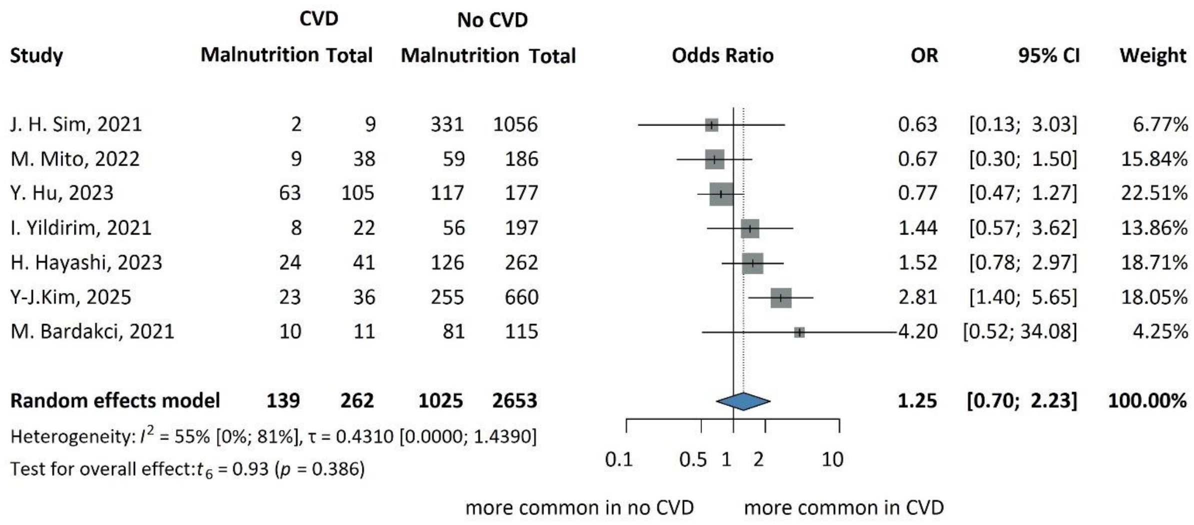

Abbreviation: CVD= cardiovascular diseases

Figure S50.: Association between malnutrition-related complication risk and hypertension in gastrointestinal cancer [subgroup analysis for gastric and colorectal cancer] (Biological composite scores)

Study	Hypertension Malnutrition	Hypertension Total	No hypertension Malnutrition	No hypertension Total	OR	95% CI	Weight
**colorectal**							
X. Wang, 2020a	12	52	70	229	0.68	[0.34; 1.38]	3.07%
L. J. Xia, 2020a	8	45	24	109	0.77	[0.31; 1.86]	1.96%
M. Bailón-Cuadrado, 2019	41	106	28	74	1.04	[0.56; 1.91]	4.00%
Y. Li, 2025	211	463	798	2090	1.36	[1.11; 1.66]	23.67%
S. Lee, 2023	25	63	22	68	1.38	[0.67; 2.82]	2.97%
H. Xie, 2022	64	153	270	861	1.57	[1.11; 2.24]	10.65%
**Random effects model**	**361**	**882**	**1212**	**3431**	**1.24**	**[0.92; 1.67]**	**46.33%**
Heterogeneity: I^2 = 22% [0%; 67%]τ = 0.1502							
Test for effect in subgroup: t_5 = 1.87 (p = 0.121)							
**esophageal**							
P. Fang, 2024	11	60	56	254	0.79	[0.39; 1.63]	2.95%
J. Feng, 2022	45	63	105	153	1.14	[0.60; 2.18]	3.62%
**gastric**							
S. Tekin, 2023	3	13	40	111	0.53	[0.14; 2.05]	0.87%
C. A. Oh, 2012	130	159	436	510	0.76	[0.47; 1.22]	6.43%
Y. Zou, 2025a	55	115	239	463	0.86	[0.57; 1.29]	8.27%
X. Zhang, 2021	91	139	216	315	0.87	[0.57; 1.33]	7.80%
T. Takahashi, 2020	20	41	23	45	0.91	[0.39; 2.12]	2.15%
K. Sakurai, 2015	41	158	104	436	1.12	[0.74; 1.70]	7.95%
T. Kosuga, 2019	14	48	31	119	1.17	[0.55; 2.46]	2.75%
X. Ma, 2025a	25	129	42	275	1.33	[0.77; 2.30]	4.93%
Y. Qian, 2021	41	117	54	192	1.38	[0.84; 2.26]	5.94%
**Random effects model**	**420**	**919**	**1185**	**2466**	**0.99**	**[0.83; 1.19]**	**47.09%**
**Prediction interval**						**[0.81; 1.23]**	
Heterogeneity: I^2 = 0% [0%; 65%]τ = 0							
Test for effect in subgroup: t_8 = -0.07 (p = 0.943)							
**Random effects model**	**837**	**1924**	**2558**	**6304**	**1.12**	**[0.97; 1.28]**	**100.00%**
**Prediction interval**						**[0.89; 1.40]**	
Heterogeneity: I^2 = 15% [0%; 51%]τ = 0.0829 [0.0000; 0.3017]							
Test for overall effect: t_{16} = 1.71 (p = 0.107)							

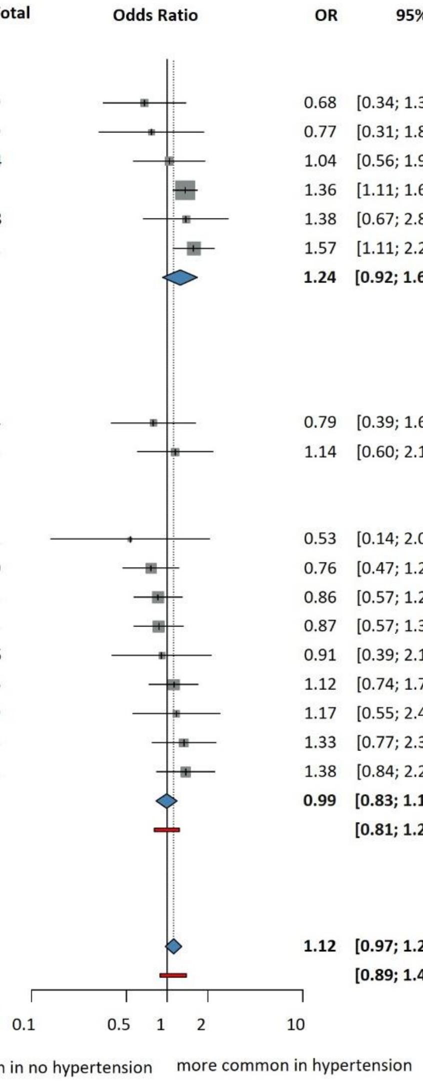

Figure S51.: Association between cachexia and hypertension in gastrointestinal cancer

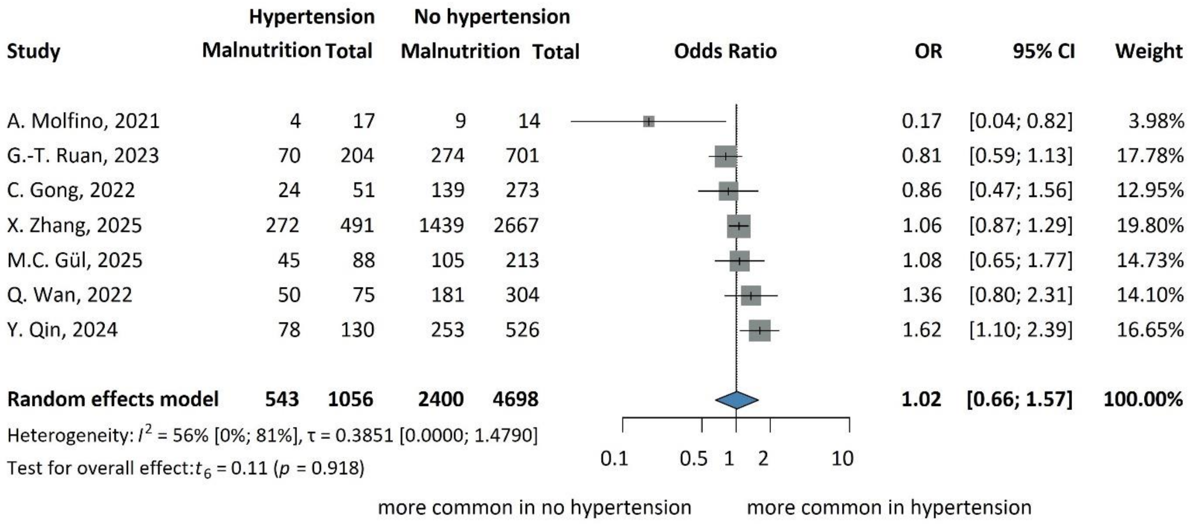

Figure S52.: Association between malnutrition risk and diabetes mellitus in gastrointestinal cancer [subgroup analysis for colorectal cancer] (Symptom-based risk assessment tool)

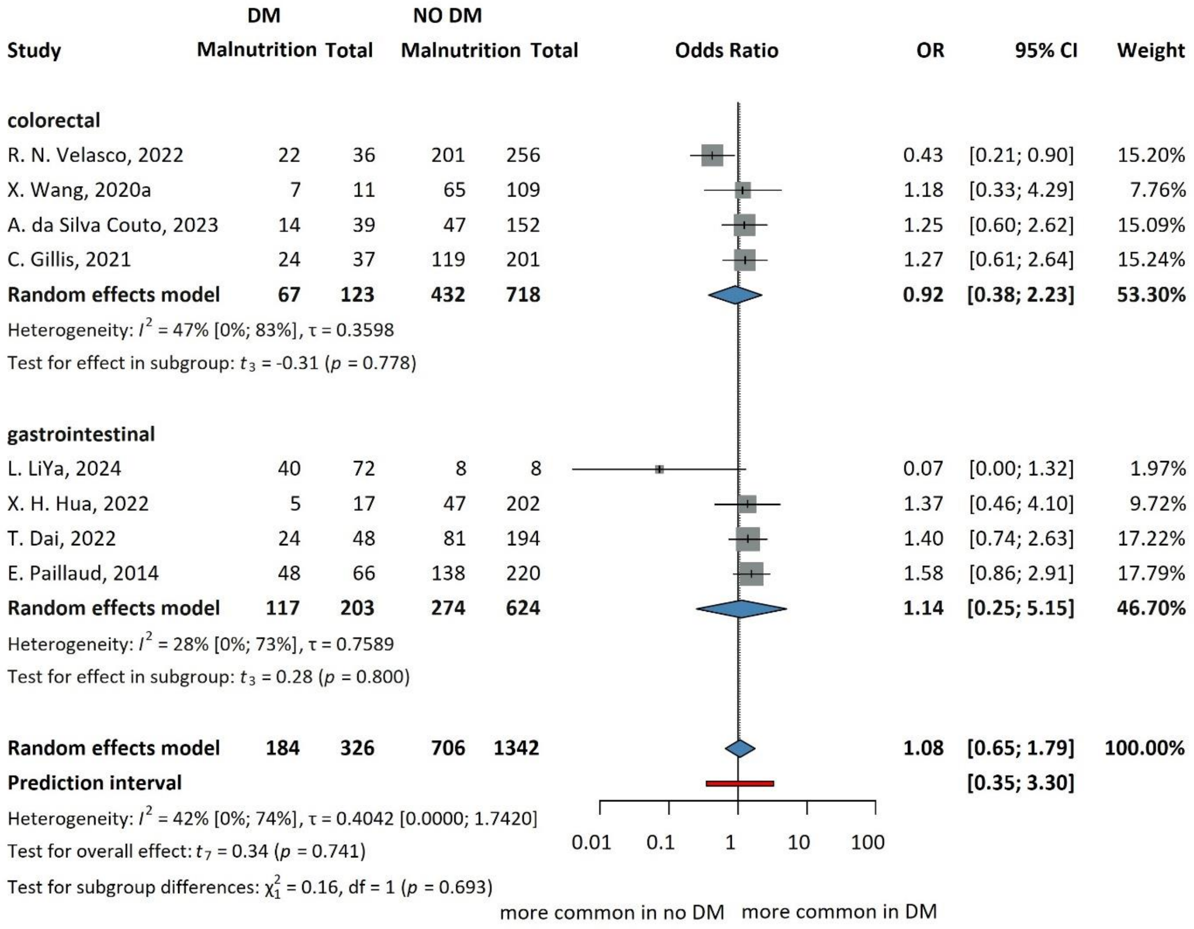

Abbreviation: DM= diabetes mellitus

Figure S53.: Association between malnutrition risk and diabetes mellitus in hepato-biliopancreatic cancer (Symptom-based risk assessment tool)

Study	DM Malnutrition	DM Total	No DM Malnutrition	No DM Total	OR	95% CI	Weight
M. Rivelsrud, 2021	6	15	96	134	0.26	[0.09; 0.79]	9.19%
X. Chen, 2022	20	44	170	315	0.71	[0.38; 1.34]	21.28%
J. Zhang, 2024	31	48	123	178	0.82	[0.42; 1.60]	19.68%
S. E. Oh, 2022	62	150	228	514	0.88	[0.61; 1.28]	37.48%
A. G. Wijma, 2024	3	7	9	23	1.17	[0.21; 6.48]	4.14%
S. Huang, 2021	10	16	22	44	1.67	[0.52; 5.38]	8.23%
**Random effects model**	**132**	**280**	**648**	**1208**	**0.79**	**[0.49; 1.27]**	**100.00%**

Heterogeneity: I^2 = 17% [0%; 62%], τ = 0.2352 [0.0000; 1.4058]

Test for overall effect: t_5 = -1.26 (p = 0.262)

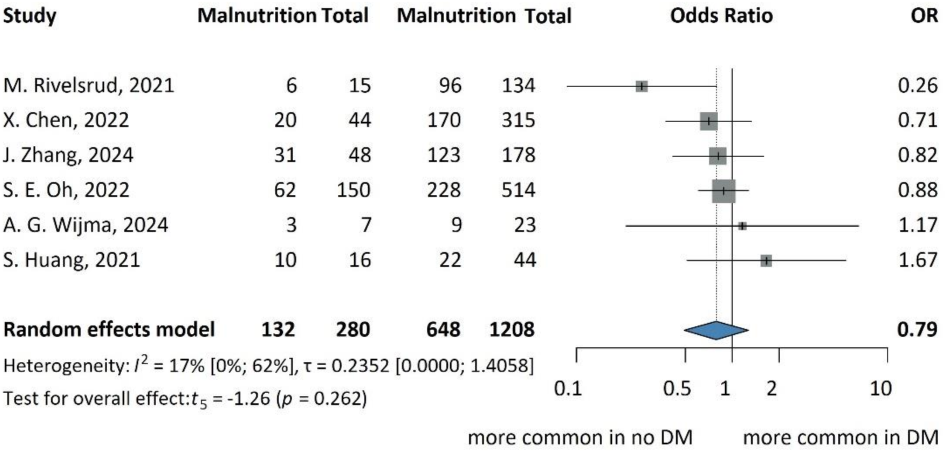

Abbreviation: DM= diabetes mellitus

Figure S54.: Association between mild severity of malnutrition-related complication risk and diabetes mellitus in gastrointestinal cancer (Biological composite scores)

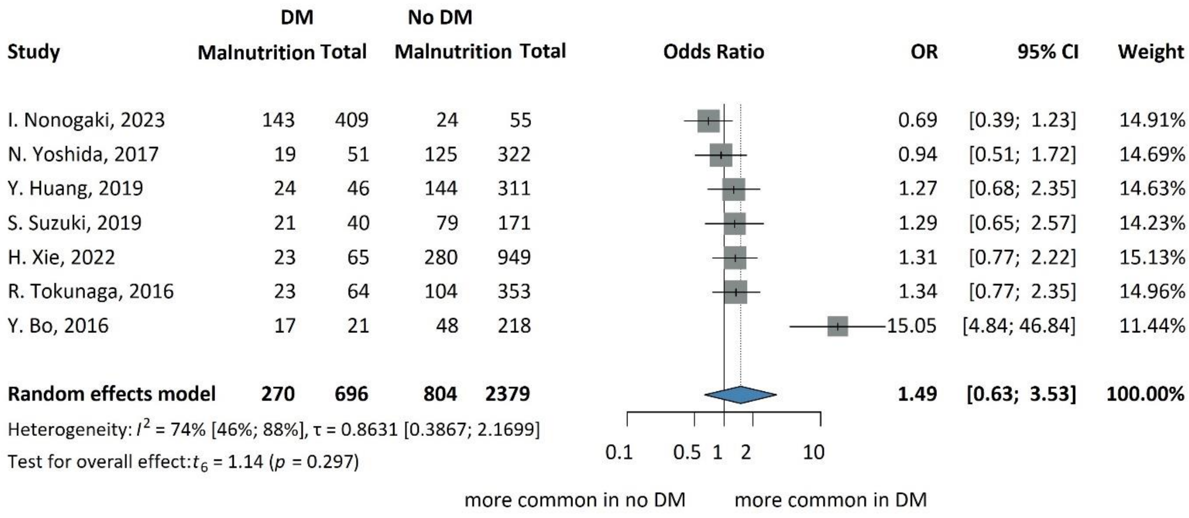

Abbreviation: DM= diabetes mellitus

Figure S55.: Association between moderate or severe severity of malnutrition-related complication risk and diabetes mellitus in gastrointestinal cancer (Biological composite scores)

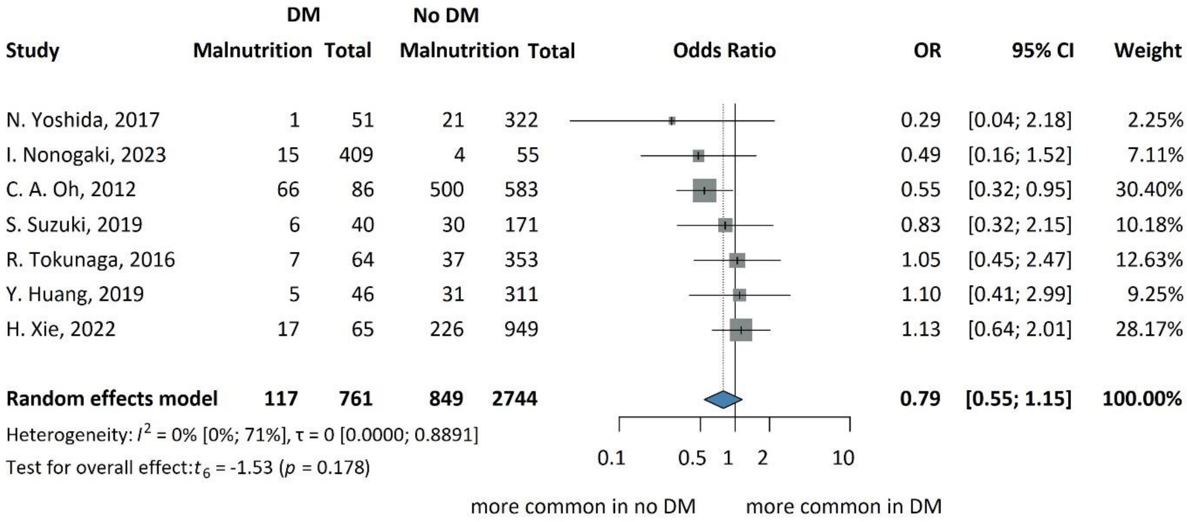

Abbreviation: DM= diabetes mellitus

Figure S56.: Association between malnutrition diagnosis and diabetes mellitus in gastrointestinal cancer (guidelines)

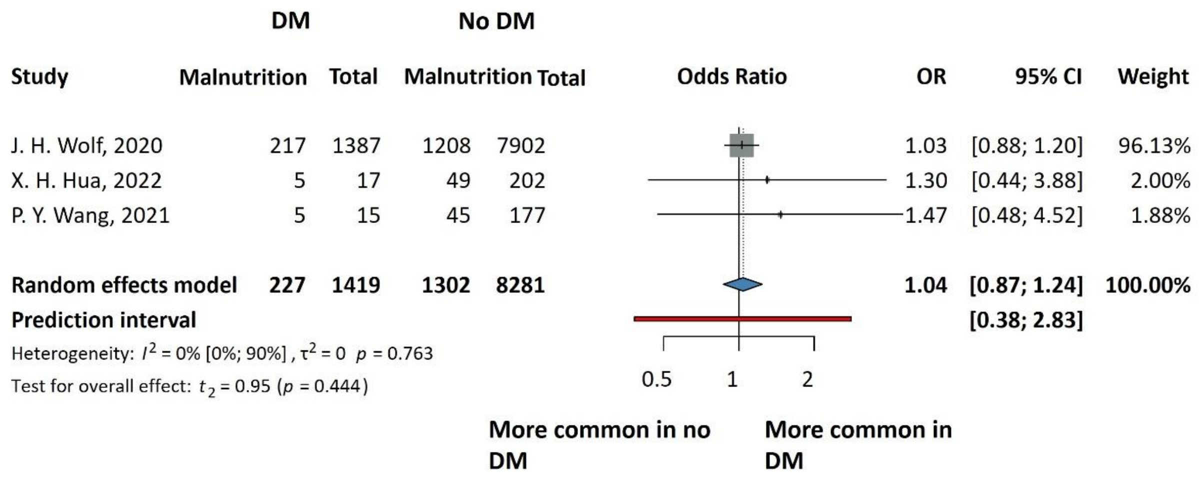

Abbreviation: DM= diabetes mellitus

Figure S57.: Association between cachexia and diabetes mellitus in gastrointestinal cancer

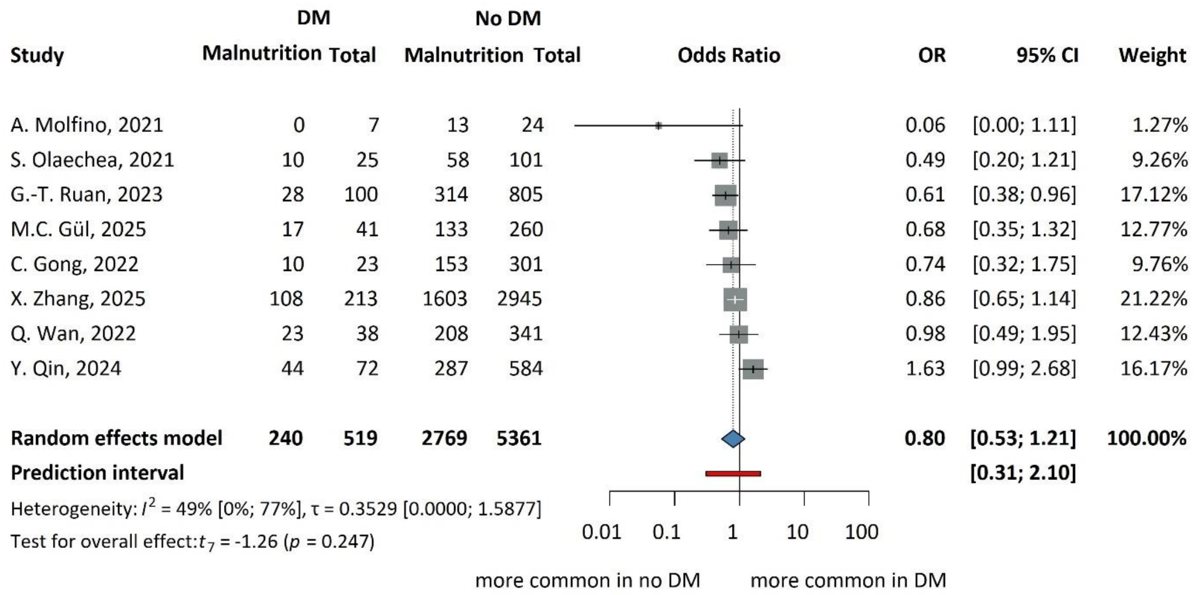

Abbreviation: DM= diabetes mellitus

Figure S58.: Association between cachexia and diabetes mellitus in hepato-biliopancreatic cancer

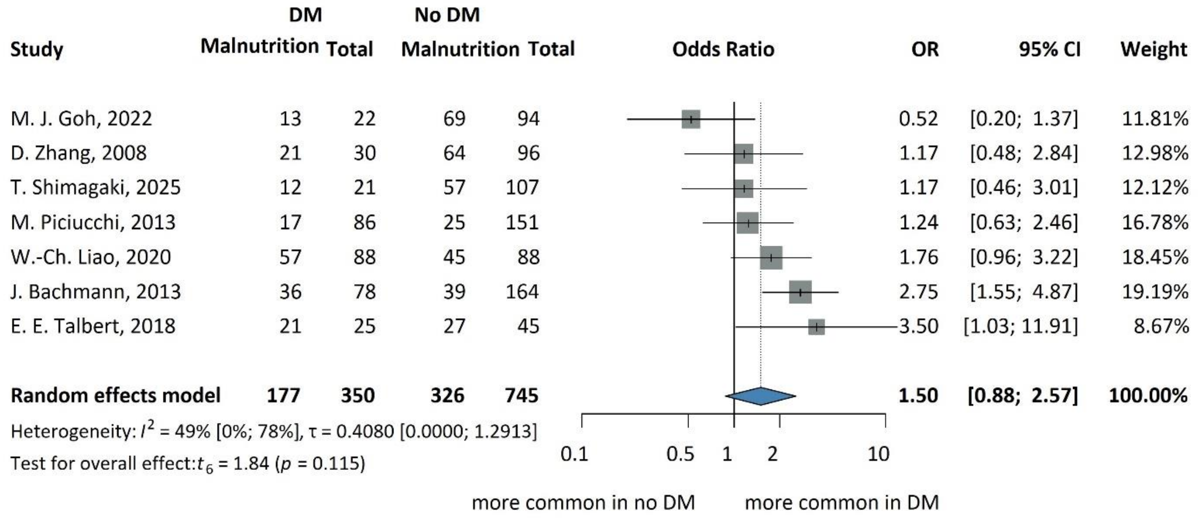

Abbreviation: DM= diabetes mellitus

Figure S59.: Association between cachexia and diabetes mellitus in pancreatic ductal adenocarcinoma

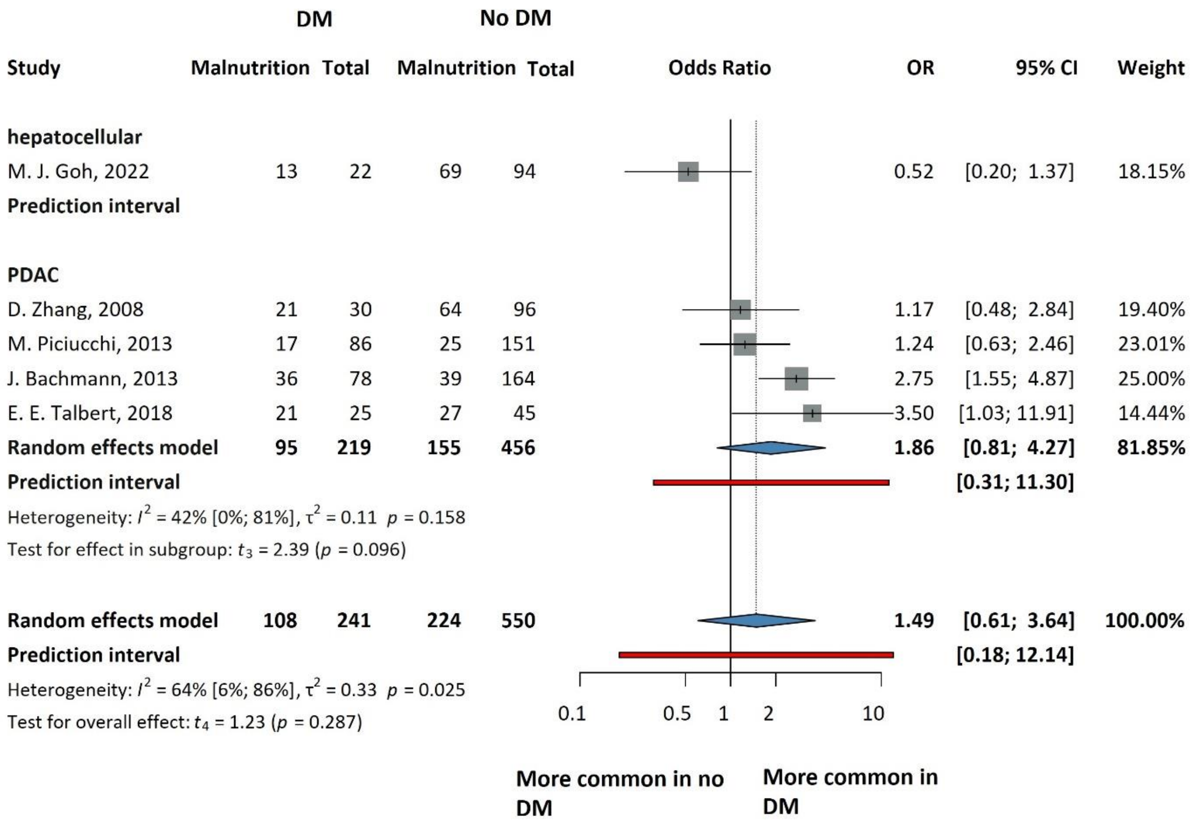

Abbreviation: DM= diabetes mellitus; PDAC= pancreatic ductal adenocarcinoma

Figure S60.: Association between malnutrition risk and chronic kidney disease in gastrointestinal cancer (Symptom-based risk assessment tool)

	Chronic kidney disease		No Chronic kidney disease					
**Study**	**Malnutrition**	**Total**	**Malnutrition**	**Total**	**Odds Ratio**	**OR**	**95% CI**	**Weight**
R. N. Velasco, 2022	6	8	217	284		0.93	[0.18; 4.70]	30.88%
E. Paillaud, 2014	17	21	169	239		1.76	[0.57; 5.42]	37.99%
X. H. Hua, 2022	7	9	45	210		12.83	[2.58; 63.92]	31.13%
**Random effects model**	**30**	**38**	**431**	**733**		**2.68**	**[0.10; 72.93]**	**100.00%**
**Prediction interval**							**[0.00; 72064902.67]**	

Heterogeneity: $I^2 = 65\%$ [0%; 90%], $\tau^2 = 1.22$ $p = 0.055$

Test for overall effect: $t_2 = 1.28$ ($p = 0.328$)

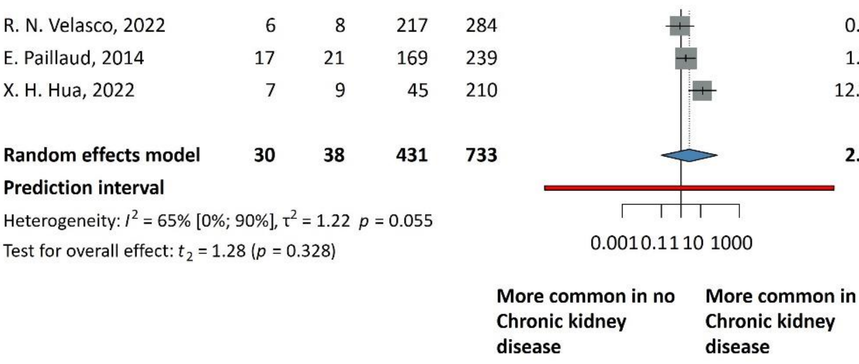

Figure S61.: Association between malnutrition-related complication risk and chronic kidney disease in gastrointestinal cancer (Biological composite scores)

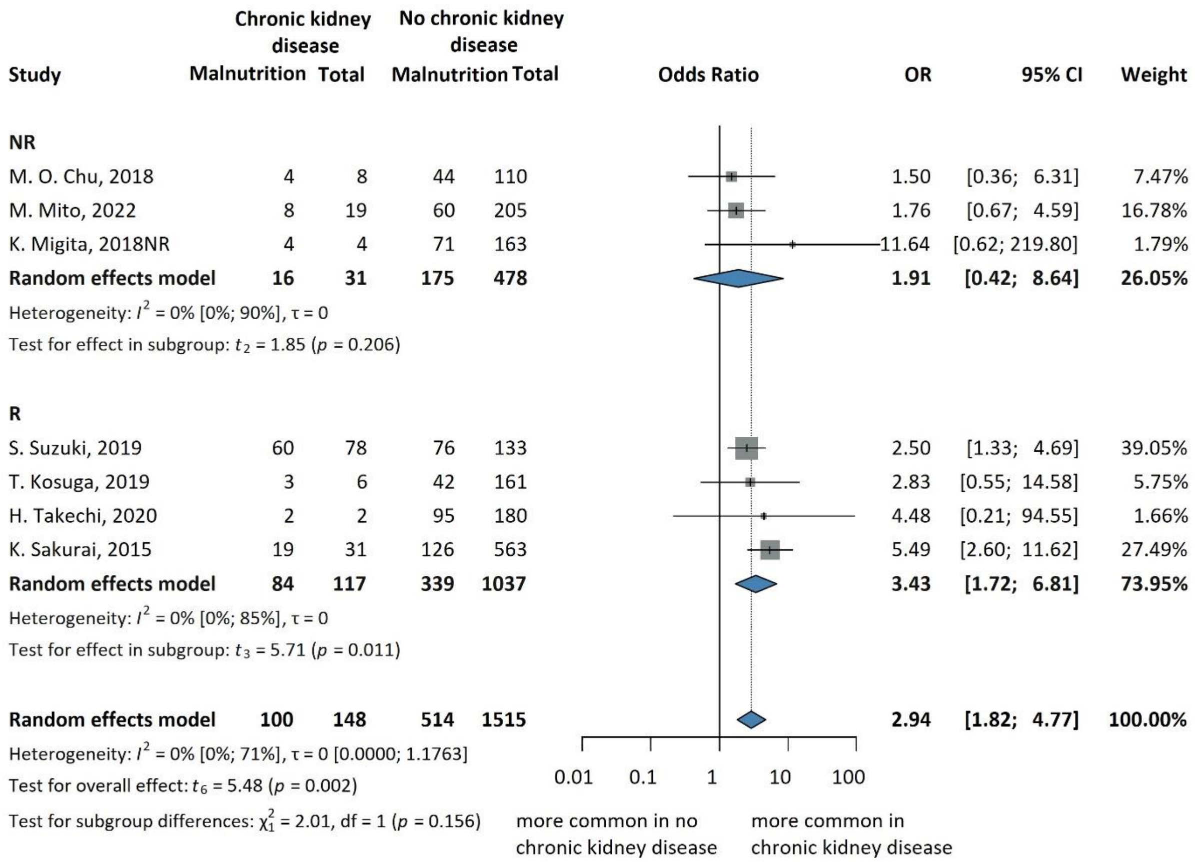

Abbreviation: R= resectable; NR= non-resectable

Figure S62.: Association between malnutrition-related complication risk and chronic respiratory disease in gastrointestinal cancer (Biological composite scores)

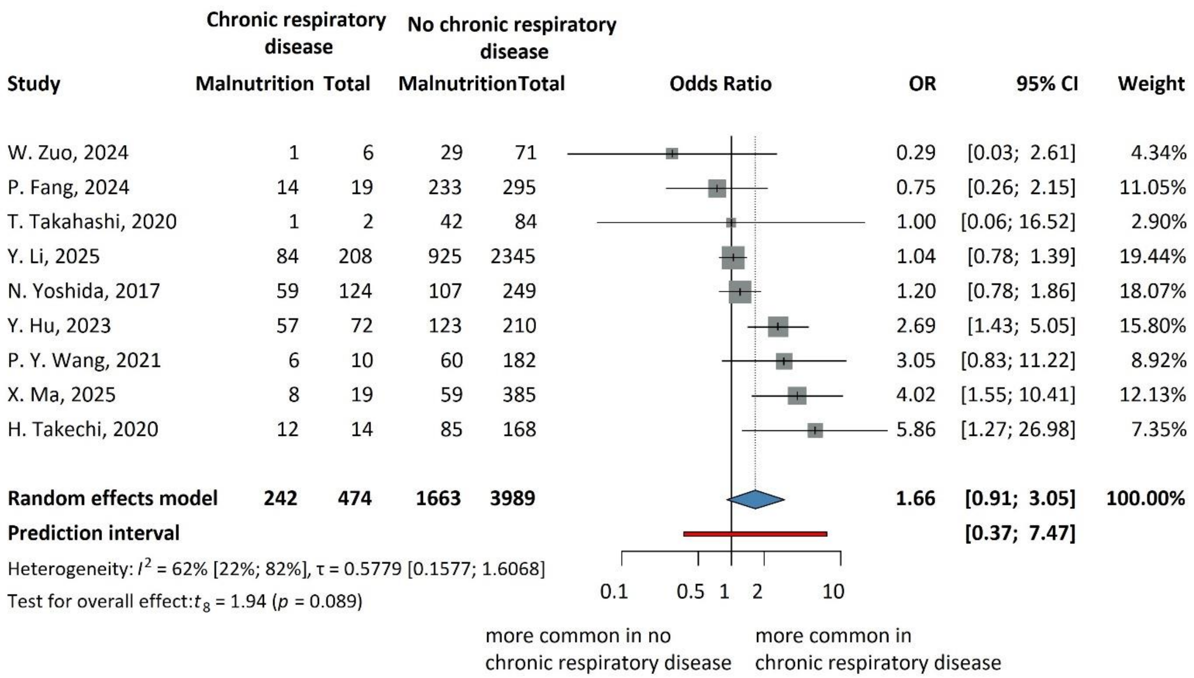

Figure S63.: Association between malnutrition diagnosis and chronic respiratory disease in gastrointestinal cancer (guidelines)

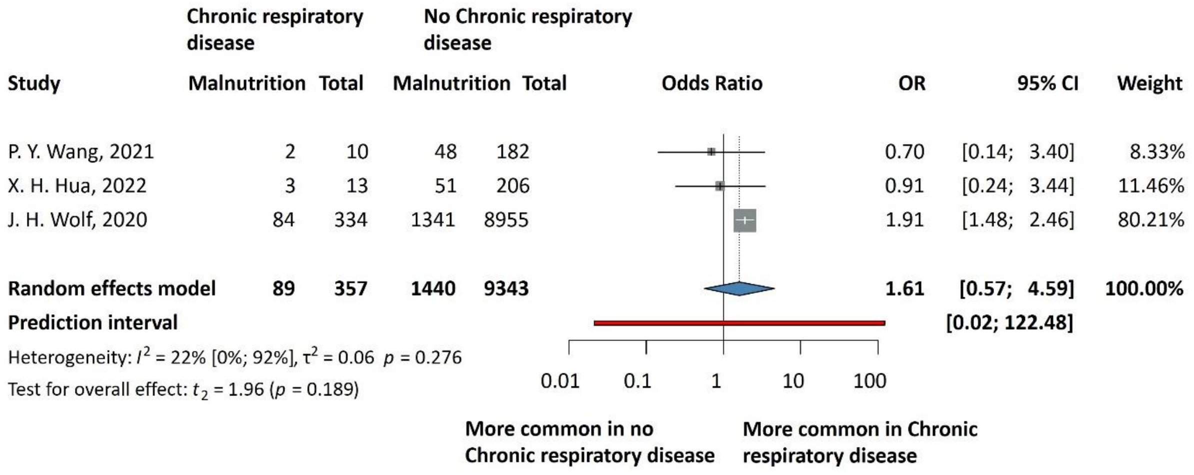

Figure S64.: Association between malnutrition risk and ASA score in gastrointestinal cancer [subgroup analysis for colorectal cancer] (Symptom-based risk assessment tool)

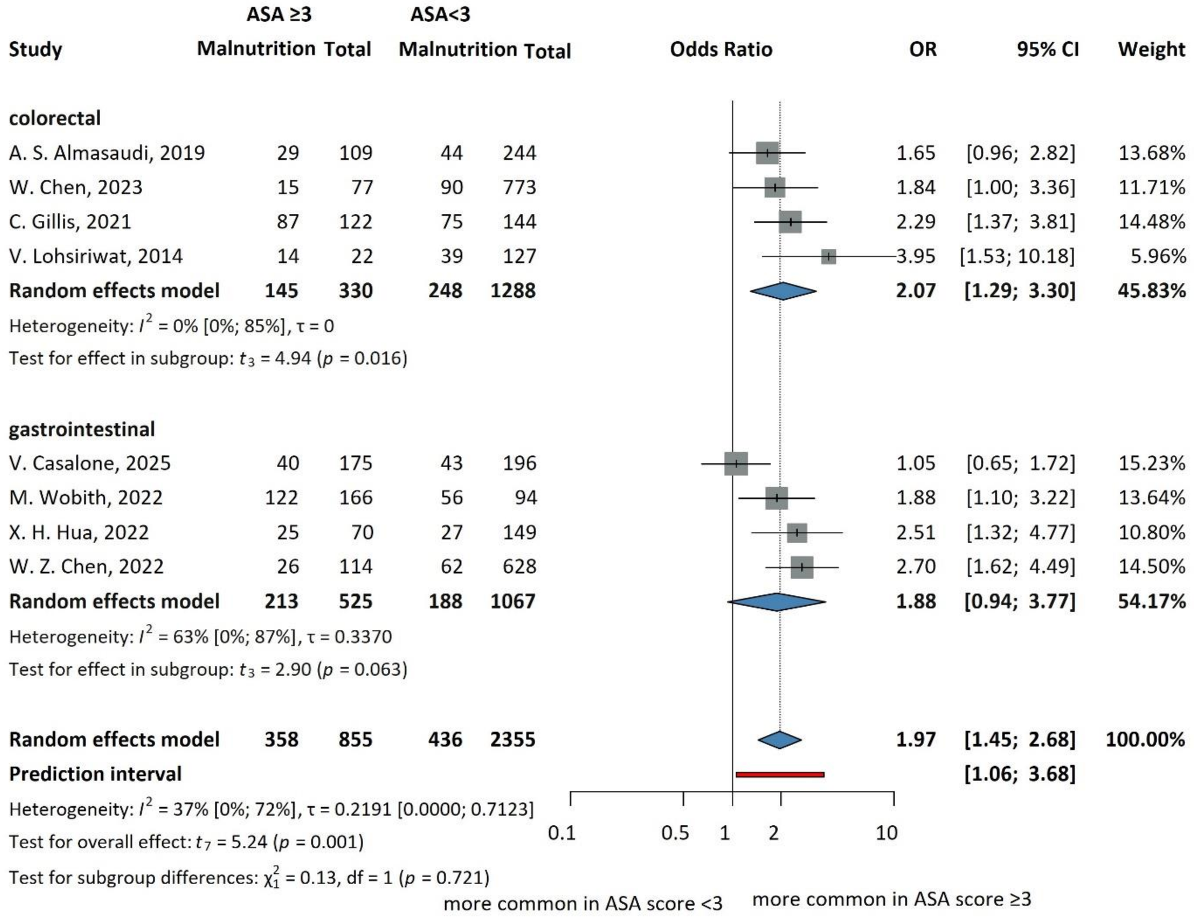

Abbreviation: ASA= American Society of Anesthesiologists Physical Status

Figure S65.: Association between malnutrition diagnosis and ASA score in gastrointestinal cancer (GLIM criteria)

Study	ASA ≥3 Malnutrition	ASA ≥3 Total	ASA<3 Malnutrition	ASA<3 Total	OR	95% CI	Weight
H.-I. Zheng, 2023	773	1118	3	3	0.32	[0.02; 6.21]	0.37%
A. Riad, 2023	1816	5496	3809	11724	1.03	[0.96; 1.10]	29.29%
X. Yan, 2024	30	104	203	879	1.35	[0.86; 2.12]	10.63%
D. D. Huang, 2022	53	156	330	1203	1.36	[0.95; 1.94]	14.19%
N. Shen, 2024	23	62	95	323	1.42	[0.80; 2.50]	7.72%
L. P. Zhou, 2021	81	106	448	654	1.49	[0.92; 2.40]	9.88%
H. N. Song, 2022	29	88	188	830	1.68	[1.05; 2.69]	10.01%
S. Omiya, 2023	26	39	137	254	1.71	[0.84; 3.47]	5.44%
M. Wobith, 2022	125	166	54	94	2.26	[1.32; 3.88]	8.32%
P. Wang, 2021	51	59	92	130	2.63	[1.14; 6.07]	4.13%
**Random effects model**	**3007**	**7394**	**5359**	**16094**	**1.40**	**[1.13; 1.72]**	**100.00%**
**Prediction interval**						**[0.90; 2.18]**	

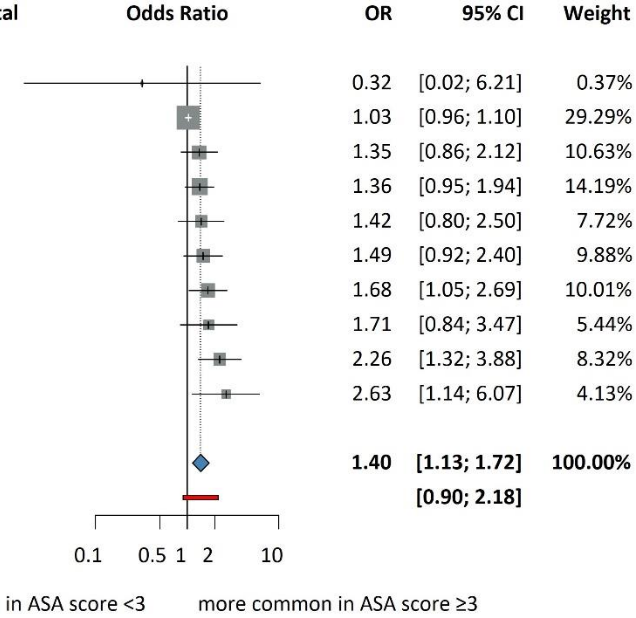

Heterogeneity: I^2 = 63% [27%; 81%], τ = 0.1686 [0.0579; 0.5377]
Test for overall effect: t_9 = 3.58 (p = 0.006)

Abbreviation: ASA= American Society of Anesthesiologists Physical Status

Figure S66.: Association between malnutrition-related complication risk and ASA score in esophageal cancer (Biological composite scores)

Study	ASA ≥3 Malnutrition	ASA ≥3 Total	ASA<3 Malnutrition	ASA<3 Total	OR	95% CI	Weight
I. Yamana, 2015	1	5	27	117	0.83	[0.09; 7.77]	4.54%
T. Toyokawa, 2016	1	11	16	174	0.99	[0.12; 8.22]	5.04%
P. Y. Wang, 2021	28	61	38	131	2.08	[1.11; 3.90]	57.13%
N. Yoshida, 2017	17	24	149	349	3.26	[1.32; 8.06]	27.60%
I. Hashimoto, 2024	2	4	37	199	4.38	[0.60; 32.10]	5.70%
**Random effects model**	**49**	**105**	**267**	**970**	**2.27**	**[1.33; 3.85]**	**100.00%**

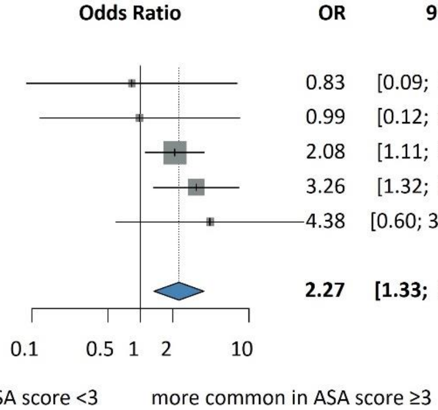

Heterogeneity: I^2 = 0% [0%; 79%], τ = 0 [0.0000; 1.8251]
Test for overall effect: t_4 = 4.29 (p = 0.013)

Abbreviation: ASA= American Society of Anesthesiologists Physical Status

Figure S67.: Association between malnutrition-related complication risk and ASA score in gastric cancer (Biological composite scores)

Study	ASA ≥3 Malnutrition	ASA ≥3 Total	ASA<3 Malnutrition	ASA<3 Total	OR	95% CI	Weight
J. Y. Lee, 2016	5	273	705	7405	0.18	[0.07; 0.43]	5.83%
Z. F. Zheng, 2018	8	15	233	317	0.41	[0.14; 1.17]	5.34%
Y. Zou, 2025	58	118	236	460	0.92	[0.61; 1.38]	7.19%
Z. Luo, 2019	11	22	53	106	1.00	[0.40; 2.51]	5.73%
K. Sugawara, 2020	23	46	111	263	1.37	[0.73; 2.57]	6.62%
X. Xi, 2022	79	138	327	674	1.42	[0.98; 2.06]	7.27%
T. Takahashi, 2020	18	32	25	54	1.49	[0.62; 3.59]	5.85%
Y. Huang, 2019	57	88	147	269	1.53	[0.93; 2.51]	6.97%
S. Suzuki, 2019	33	46	103	165	1.53	[0.75; 3.12]	6.36%
T. Matsunaga, 2022	52	89	156	408	2.27	[1.42; 3.62]	7.05%
N. Hirahara, 2020	17	33	80	264	2.44	[1.18; 5.08]	6.31%
F. Borda, 2017	33	60	20	64	2.69	[1.29; 5.60]	6.31%
Y. Xiao, 2022	123	241	510	2111	3.27	[2.49; 4.29]	7.44%
J. Nogueiro, 2022	22	64	19	139	3.31	[1.63; 6.71]	6.39%
I. Tirnova, 2025	25	37	9	30	4.86	[1.72; 13.77]	5.35%
K. Sakurai, 2016	14	16	49	131	11.71	[2.55; 53.74]	3.98%
**Random effects model**	**578**	**1318**	**2783**	**12860**	**1.64**	**[1.02; 2.64]**	**100.00%**
**Prediction interval**						**[0.27; 9.91]**	

Heterogeneity: $I^2 = 82\%$ [72%; 88%], $\tau = 0.8080$ [0.5006; 1.3928]

Test for overall effect: $t_{15} = 2.22$ ($p = 0.043$)

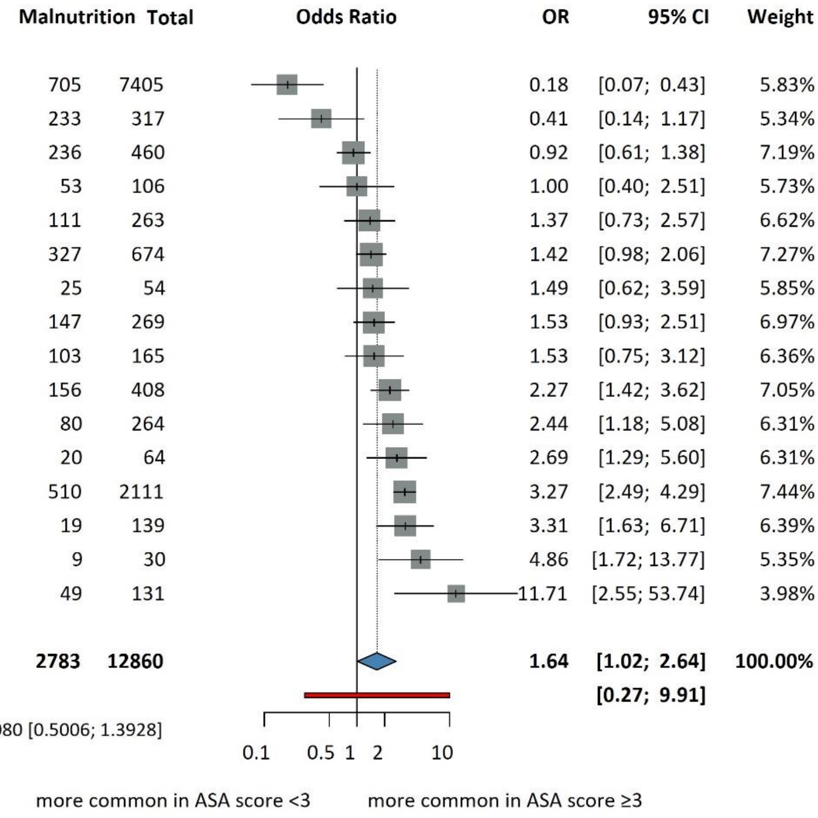

Abbreviation: ASA= American Society of Anesthesiologists Physical Status

Figure S68.: Association between malnutrition-related complication risk and ASA score in colorectal cancer (Biological composite scores)

Study	ASA ≥3 Malnutrition	ASA ≥3 Total	ASA<3 Malnutrition	ASA<3 Total	OR	95% CI	Weight
**NR**							
Y. Ahiko, 2019	84	161	238	669	1.98	[1.40; 2.80]	12.71%
M. Sasaki, 2019	9	12	58	137	4.09	[1.06; 15.76]	2.32%
**R**							
C. Martínez-Escribano, 2022	3	11	95	314	0.86	[0.22; 3.33]	2.33%
X. Wang, 2020	43	152	39	129	0.91	[0.54; 1.52]	9.20%
H. Xie, 2020	127	230	139	282	1.27	[0.89; 1.80]	12.66%
M. Bailón-Cuadrado, 2019	28	63	41	117	1.48	[0.79; 2.77]	7.43%
L. Zhang, 2023	103	266	104	373	1.63	[1.17; 2.28]	13.00%
K. Hiramatsu, 2021	22	40	332	781	1.65	[0.87; 3.13]	7.24%
Y. Nakamura, 2023	33	74	147	527	2.08	[1.27; 3.42]	9.55%
R. Sato, 2022	3	6	17	55	2.24	[0.41; 12.23]	1.54%
T. Tominaga, 2019	11	17	91	208	2.36	[0.84; 6.61]	3.65%
M. Kataoka, 2021	25	88	112	816	2.49	[1.51; 4.13]	9.40%
S. Abe, 2024	1	2	20	91	3.55	[0.21; 59.31]	0.59%
S. Lee, 2023	9	14	38	117	3.74	[1.17; 11.93]	3.01%
K. Yoshimatsu, 2017	13	28	50	323	4.73	[2.12; 10.55]	5.36%
**Random effects model**	**421**	**991**	**1225**	**4133**	**1.76**	**[1.34; 2.32]**	**84.97%**
**Prediction interval**						**[0.90; 3.45]**	
Heterogeneity: I^2 = 43% [0%; 70%] , τ = 0.2787							
Test for effect in subgroup: t_{12} = 4.50 (p < 0.001)							
**Random effects model**	**514**	**1164**	**1521**	**4939**	**1.82**	**[1.43; 2.31]**	**100.00%**
**Prediction interval**						**[0.99; 3.35]**	
Heterogeneity: I^2 = 40% [0%; 68%] , τ = 0.2601 [0.0000; 0.6325]							
Test for overall effect: t_{14} = 5.32 (p < 0.001)							

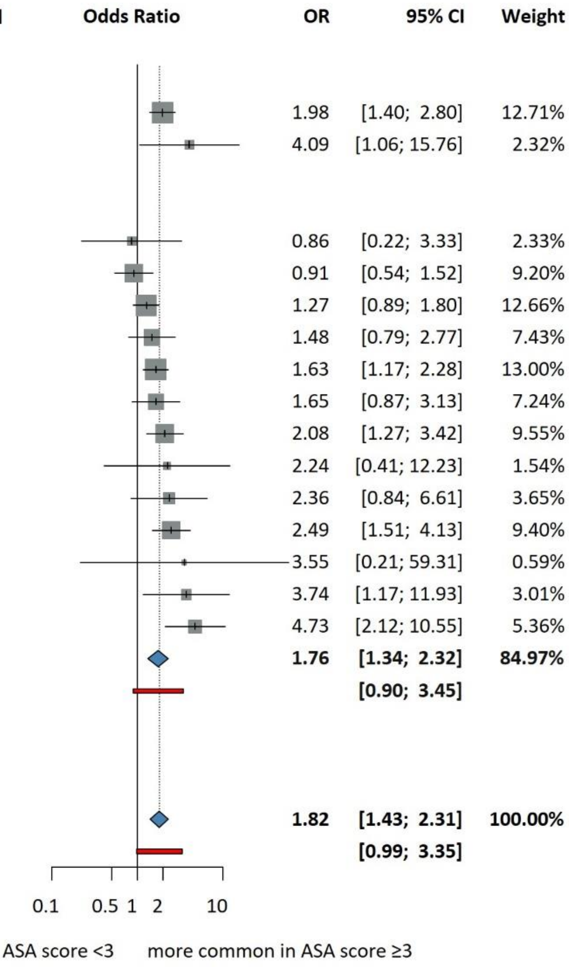

Abbreviation: ASA= American Society of Anesthesiologists Physical Status; R= resectable; NR= non-resectable

Figure S69.: Association between malnutrition-related complication risk and ASA score in hepatocellular carcinoma (Biological composite scores)

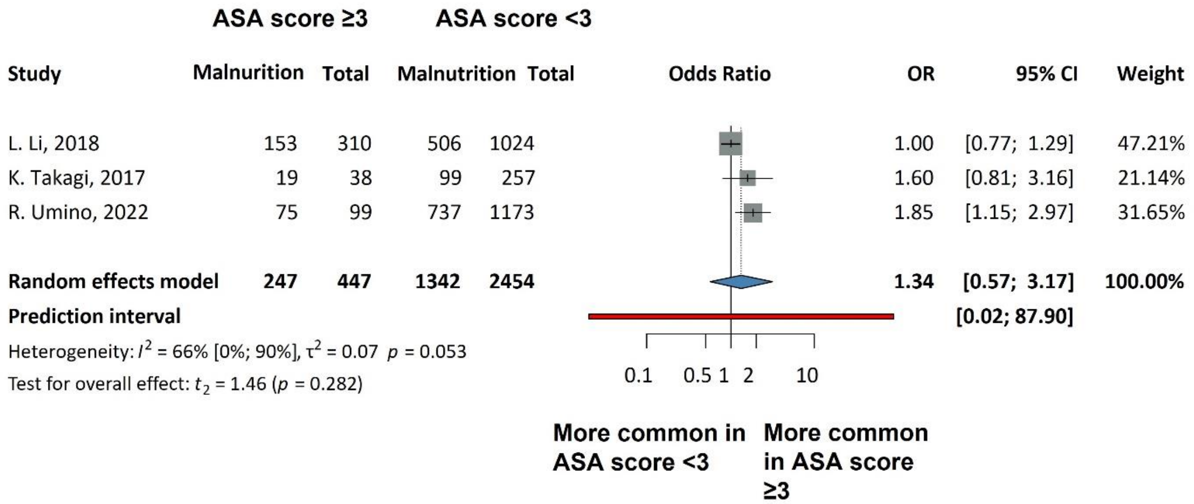

Abbreviation: ASA= American Society of Anesthesiologists Physical Status

Figure S70.: Association between malnutrition diagnosis and ASA score in gastrointestinal cancer (guidelines)

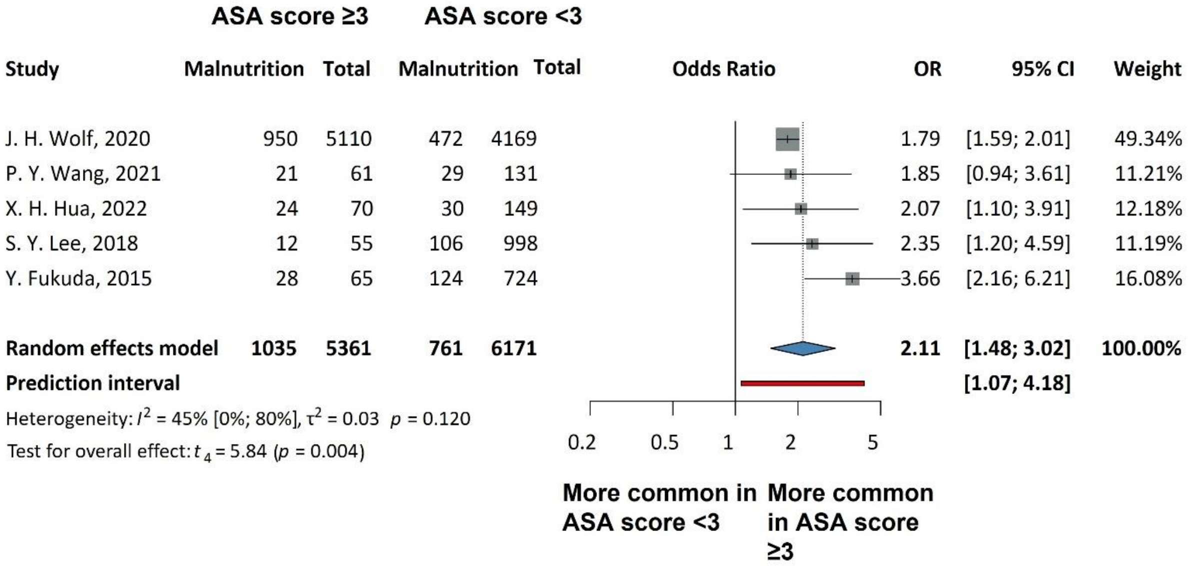

Abbreviation: ASA= American Society of Anesthesiologists Physical Status

Figure S71.: Association between ECOG >=2 and malnutrition-related complication risk in hepato-biliopancreatic cancer patients (Biological composite scores)

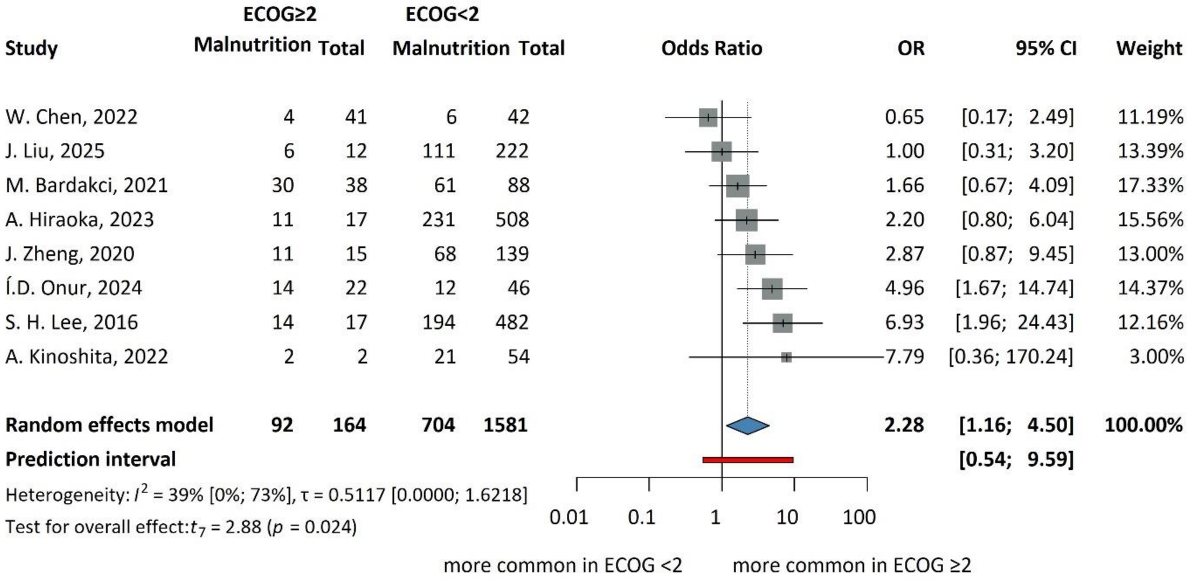

Abbreviations: ECOG= Eastern Cooperative Oncology Group

Figure S72.: Association between ECOG >=2 and malnutrition diagnosis in gastrointestinal cancer (guideline)

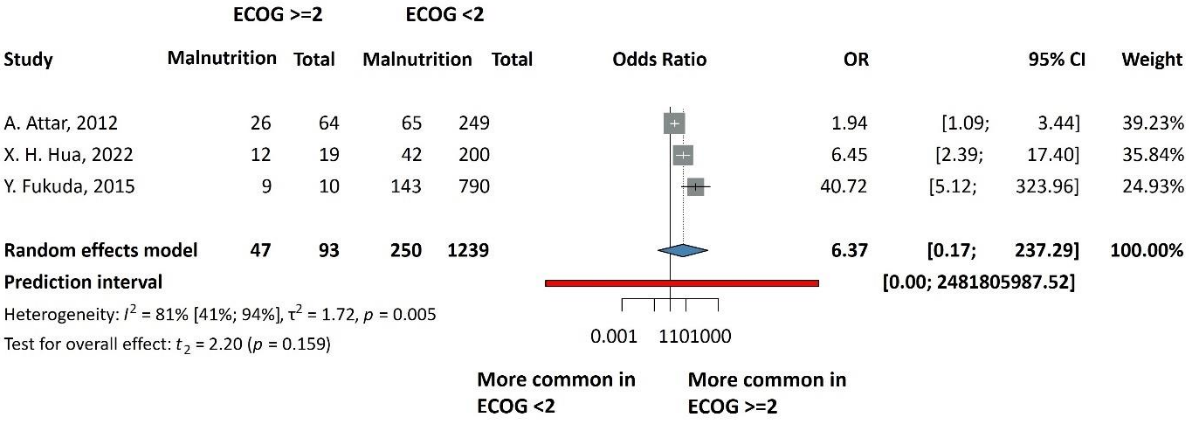

Abbreviations: ECOG= Eastern Cooperative Oncology Group

Figure S73.: Association between ECOG >=2 and malnutrition-related complication risk in colorectal patients (Biological composite scores)

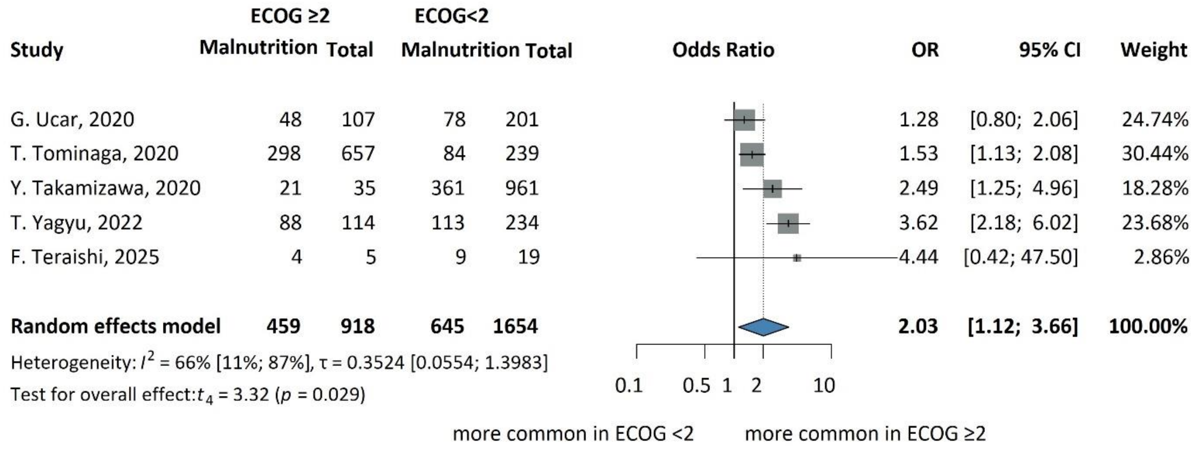

Abbreviations: ECOG= Eastern Cooperative Oncology Group

Figure S74.: Association between ECOG >=2 and malnutrition-related complication risk in gastric patients (Biological composite scores)

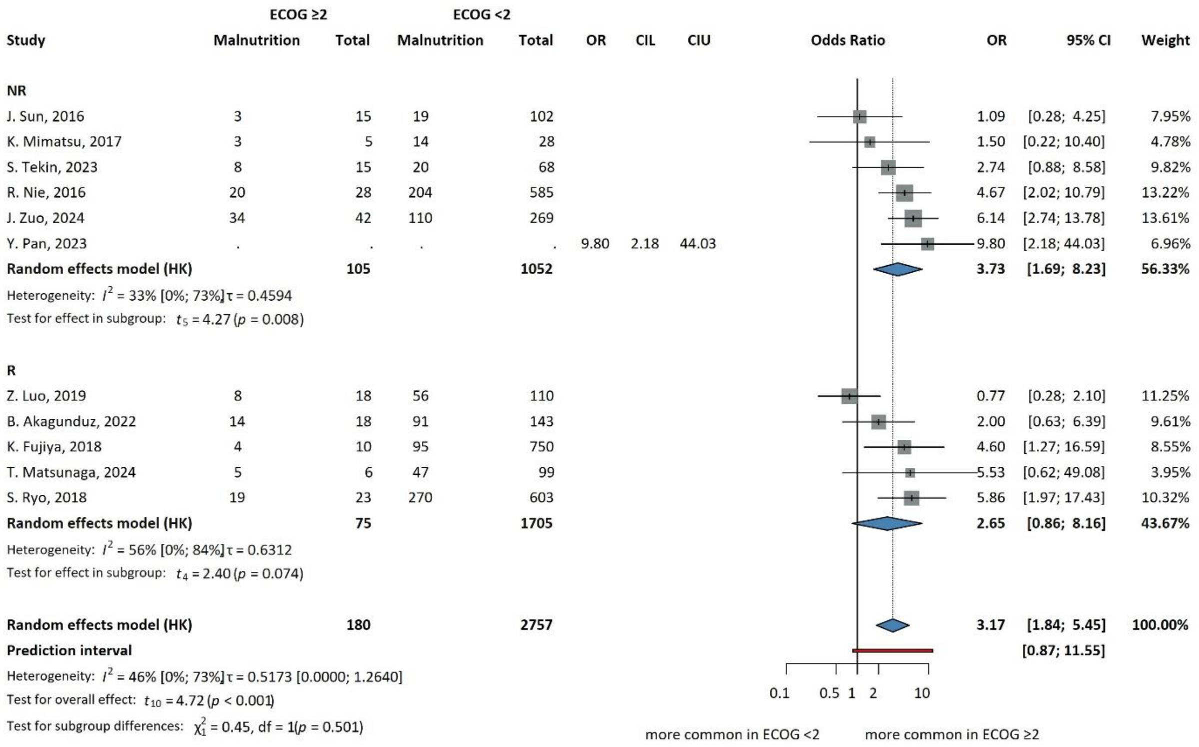

Abbreviations: ECOG= Eastern Cooperative Oncology Group; R= resectable; NR= non-resectable

Figure S75.: Association between ECOG >=1 and cachexia in HBP patients

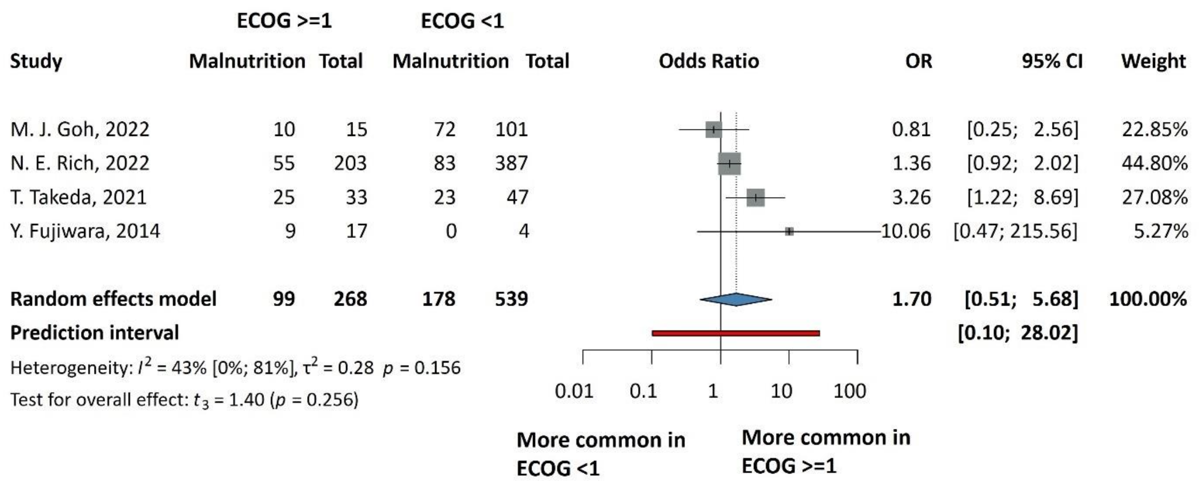

Abbreviations: ECOG= Eastern Cooperative Oncology Group

Supplementary Document S11: Individual forest plots for inflammation and other biological parameters: Figure S76-S96

Figure S76.: Association between malnutrition risk and serum CRP level in gastrointestinal cancer (Symptom-based risk assessment tool)

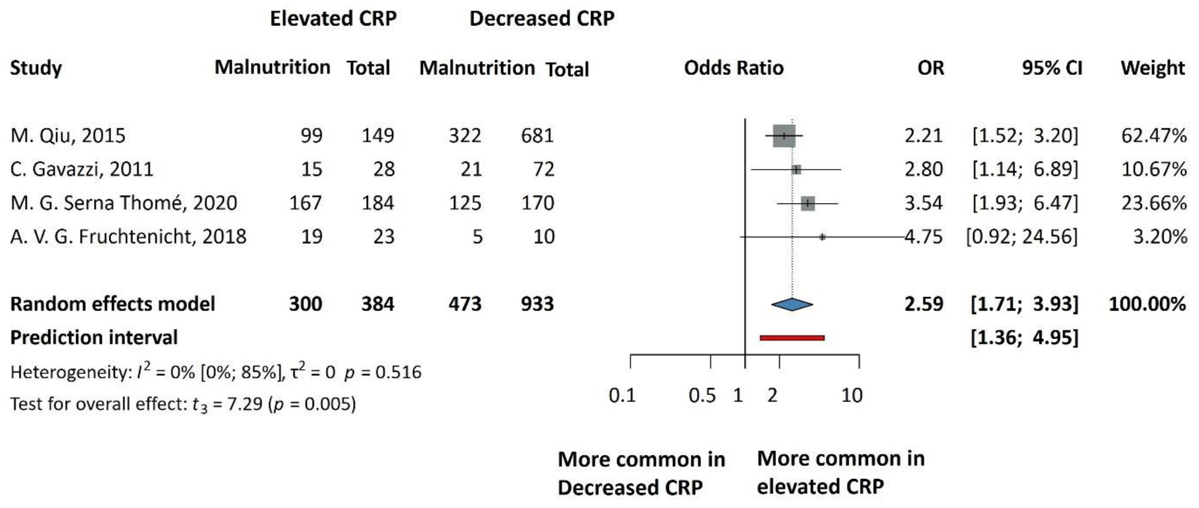

Abbreviations: CRP= C-reactive protein

Figure S77.: Association between malnutrition-related complication risk and serum lymphocyte count in gastrointestinal/gastric cancer (Biological composite scores)

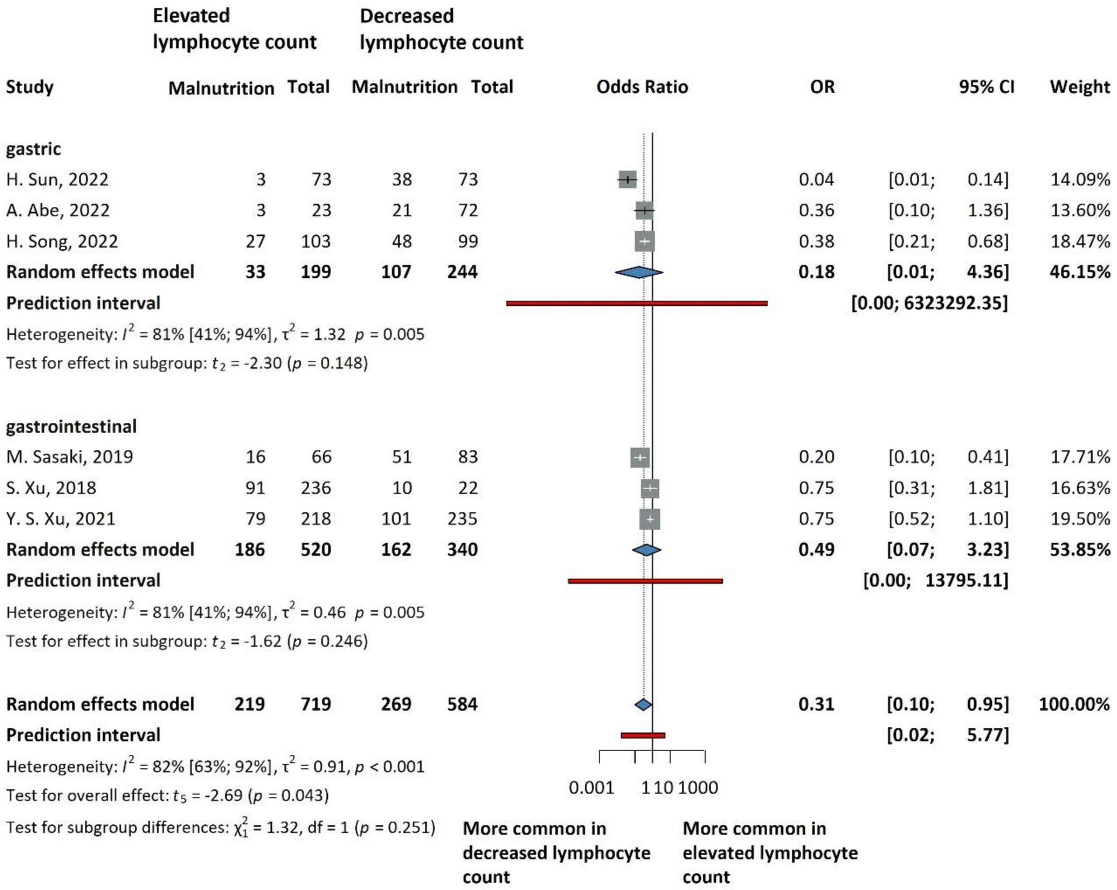

Figure S78.: Association between malnutrition-related complication risk and serum lymphocyte count in gastrointestinal cancer (Biological composite scores)

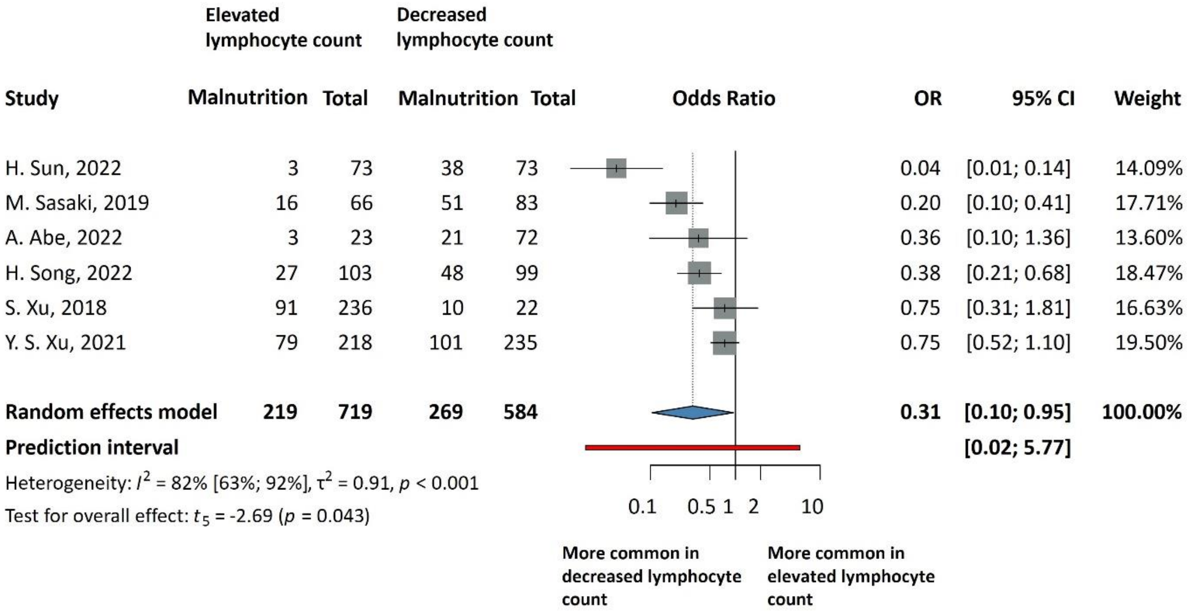

Figure S79.: Association between malnutrition-related complication risk and serum lymphocyte-monocyte ratio in gastrointestinal cancer (Biological composite scores)

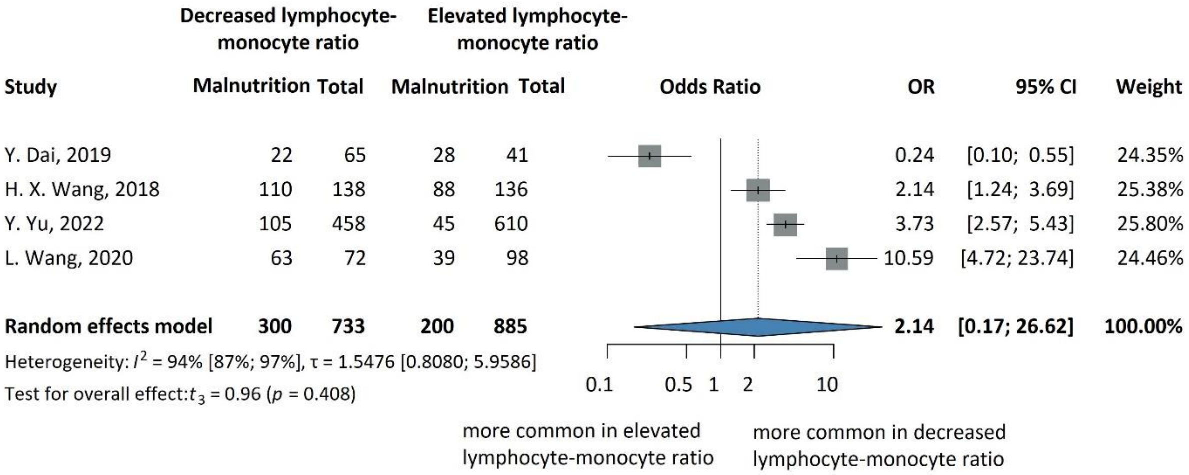

Figure S80.: Association between malnutrition-related complication risk and serum neutrophil-lymphocyte ratio in esophageal cancer (Biological composite scores)

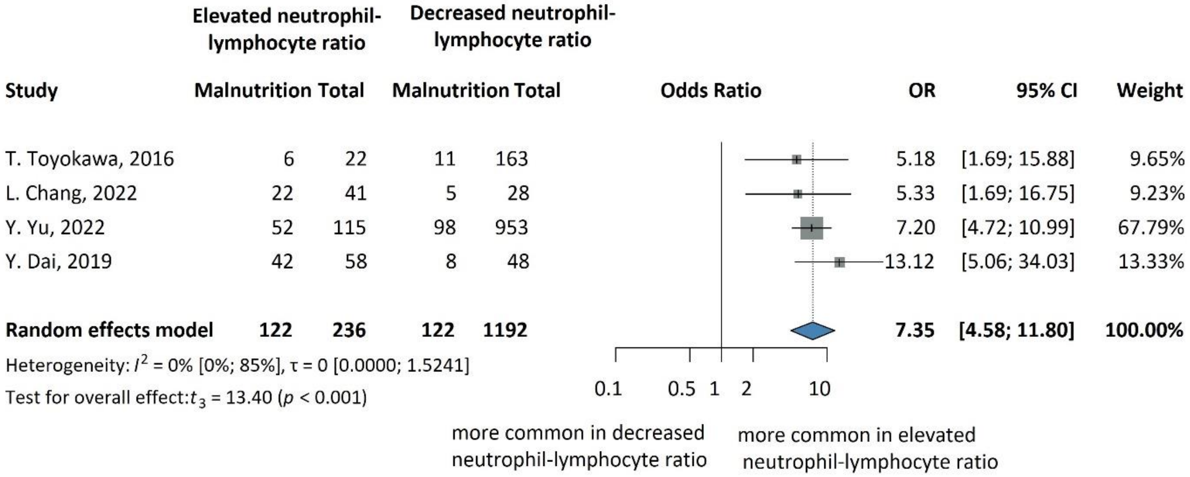

Figure S81.: Association between malnutrition-related complication risk and serum neutrophil-lymphocyte ratio in gastric cancer (Biological composite scores)

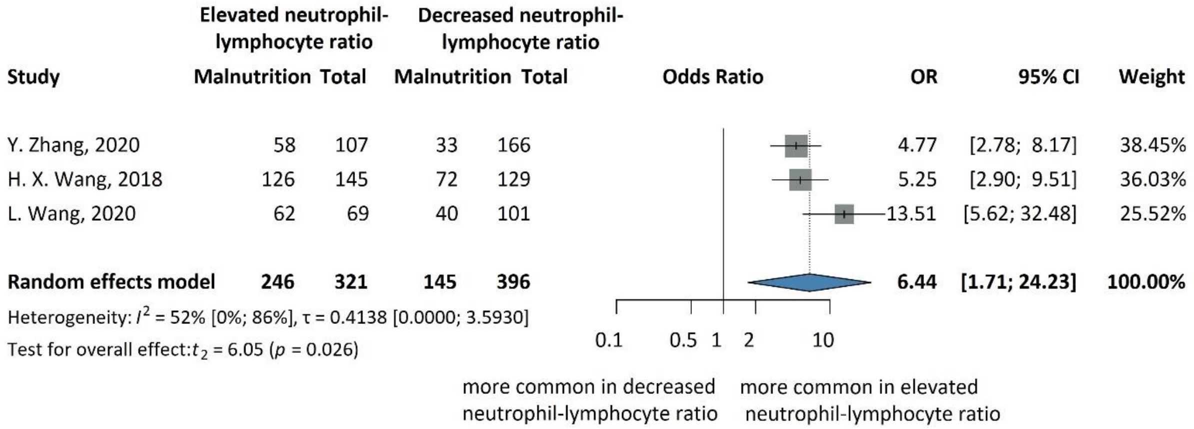

Figure S82.: Association between malnutrition-related complication risk and serum neutrophil-lymphocyte ratio in colorectal cancer (Biological composite scores)

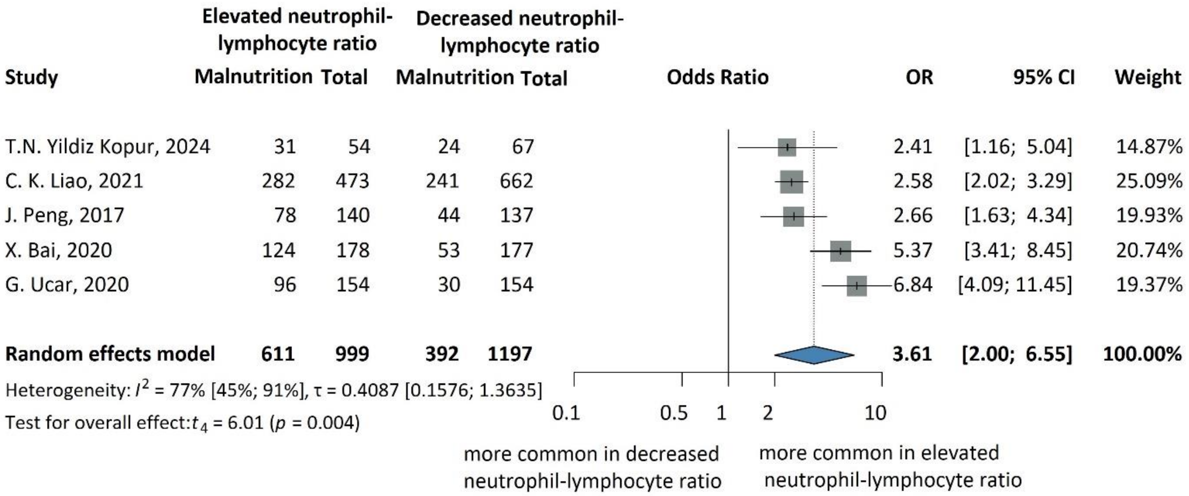

Figure S83.: Association between malnutrition-related complication risk and serum platelet-lymphocyte ratio in upper gastrointestinal cancer (Biological composite scores)

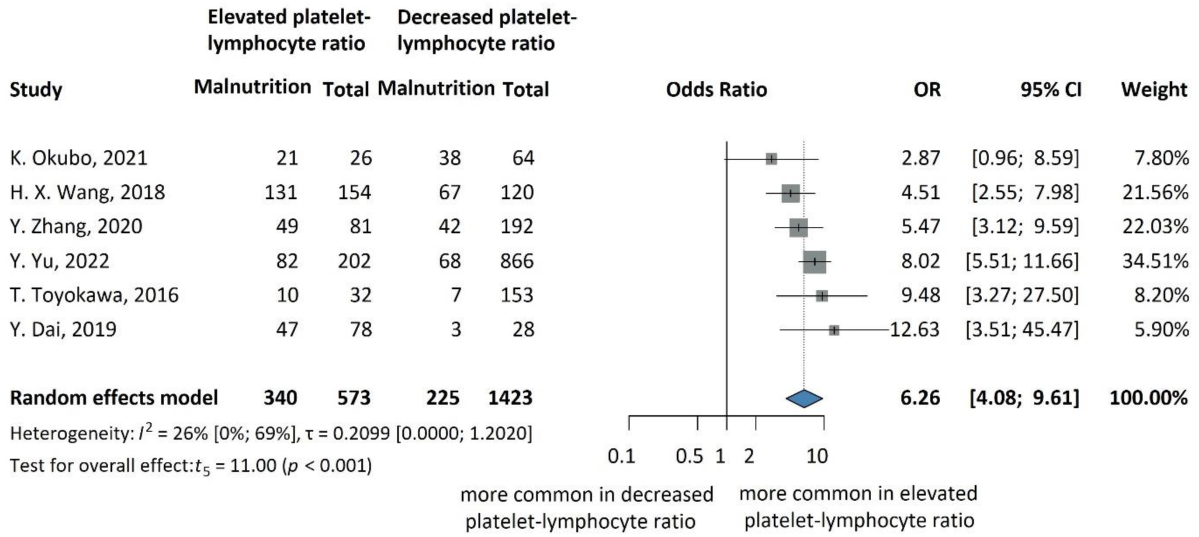

Figure S84.: Association between malnutrition-related complication risk and serum platelet-lymphocyte ratio in colorectal cancer (Biological composite scores)

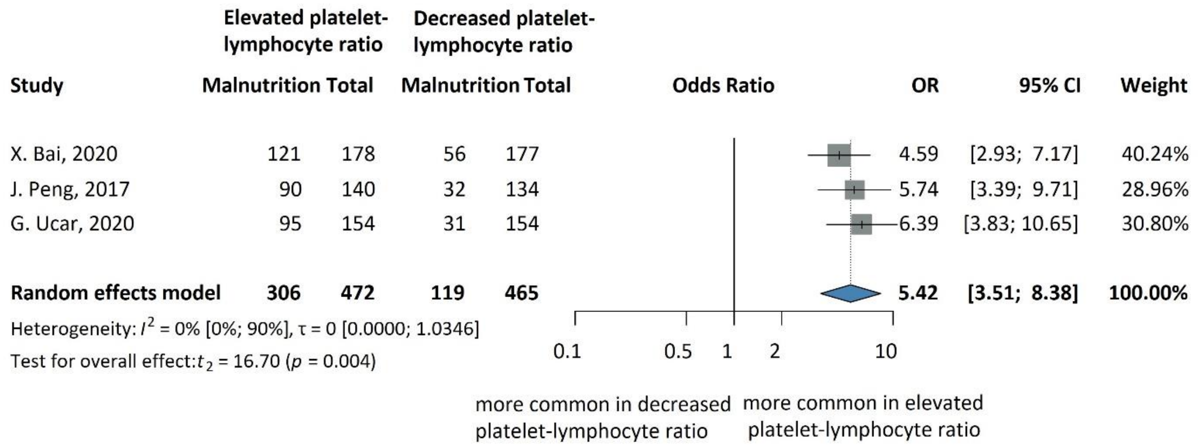

Figure S85.: Association between malnutrition-related complication risk and serum alanine aminotransferase (ALT) level in hepatocellular carcinoma (Biological composite scores)

Study	Elevated ALT Malnutrition	Elevated ALT Total	Decreased ALT Malnutrition	Decreased ALT Total	OR	95% CI	Weight
**NR**							
Y. Yang, 2020	27	99	52	304	1.82	[1.07; 3.10]	12.62%
Y. Chen, 2020	22	79	40	246	1.99	[1.09; 3.61]	11.46%
S. Li, 2021	74	101	97	184	2.46	[1.45; 4.16]	12.74%
**Random effects model**	**123**	**279**	**189**	**734**	**2.08**	**[1.40; 3.10]**	**36.83%**
Heterogeneity: I^2 = 0% [0%; 90%], τ = 0							
Test for effect in subgroup: t_2 = 7.90 (p = 0.016)							
**R**							
Z. X. Lin, 2020	80	170	107	210	0.86	[0.57; 1.28]	15.20%
F. Ji, 2017	41	165	37	157	1.07	[0.64; 1.79]	13.07%
X. Liang, 2021	89	303	141	565	1.25	[0.92; 1.71]	17.13%
Ch.-K. Yang, 2024	120	233	462	1433	2.23	[1.69; 2.95]	17.78%
**Random effects model**	**330**	**871**	**747**	**2365**	**1.30**	**[0.67; 2.52]**	**63.17%**
Heterogeneity: I^2 = 83% [57%; 93%], τ = 0.3716							
Test for effect in subgroup: t_3 = 1.24 (p = 0.304)							
**Random effects model**	**453**	**1150**	**936**	**3099**	**1.55**	**[1.06; 2.25]**	**100.00%**
Heterogeneity: I^2 = 73% [43%; 88%], τ = 0.3334 [0.1437; 0.8541]							
Test for overall effect: t_6 = 2.85 (p = 0.029)							
Test for subgroup differences: χ^2_1 = 4.27, df = 1 (p = 0.039)							

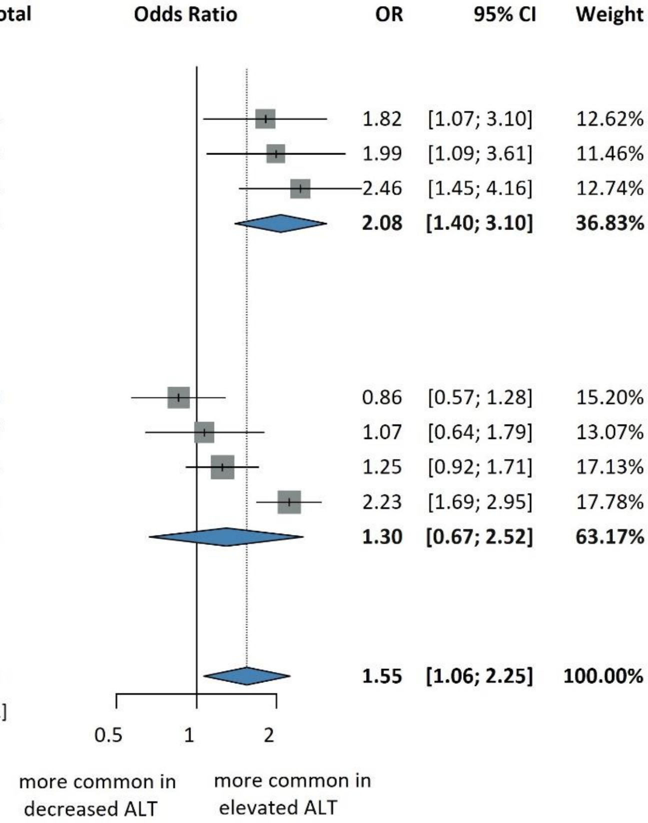

Abbreviations: R= resectable; NR= non-resectable; ALT= alanine aminotransferase

Figure S86.: Association between malnutrition-related complication risk and serum alpha-fetoprotein (AFP) (cut off 400 ng/ml) level in hepatocellular carcinoma (Biological composite scores)

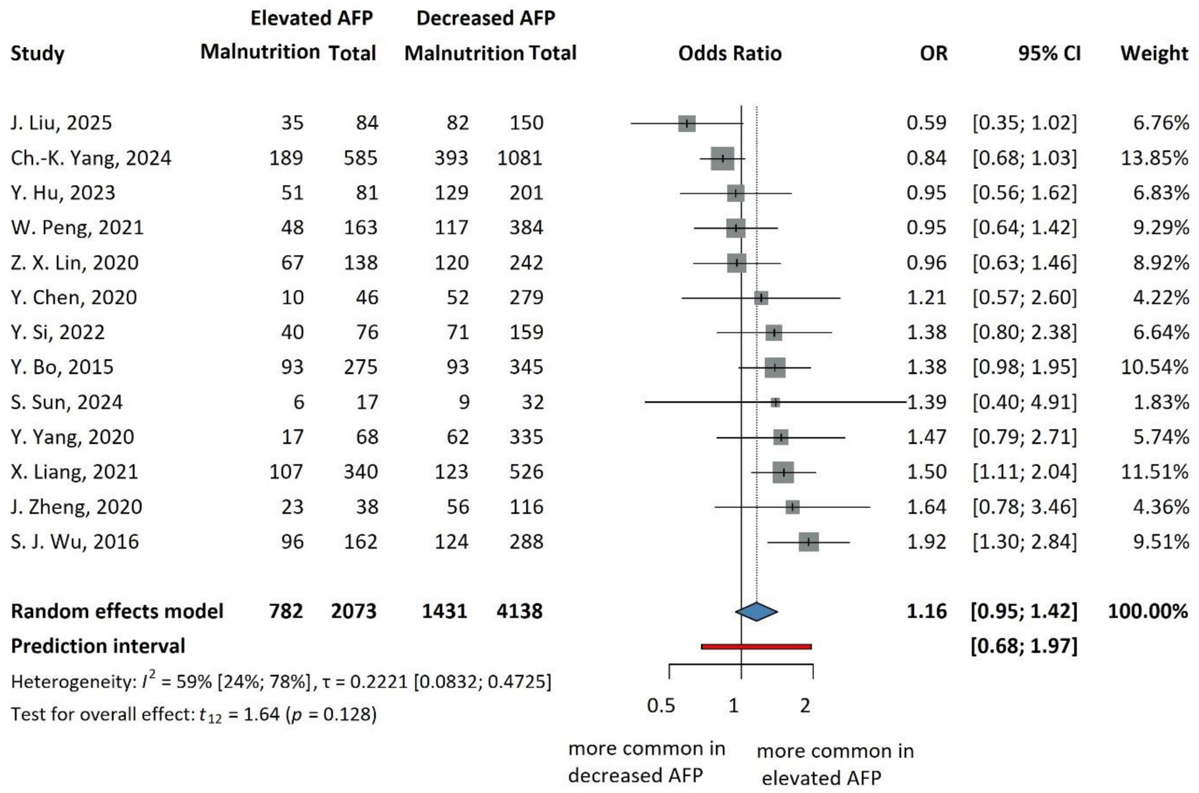

Abbreviations: AFP= alfa fetoprotein

Figure S87.: Association between malnutrition-related complication risk and serum aspartate aminotransferase (AST) level in hepatocellular carcinoma (Biological composite scores)

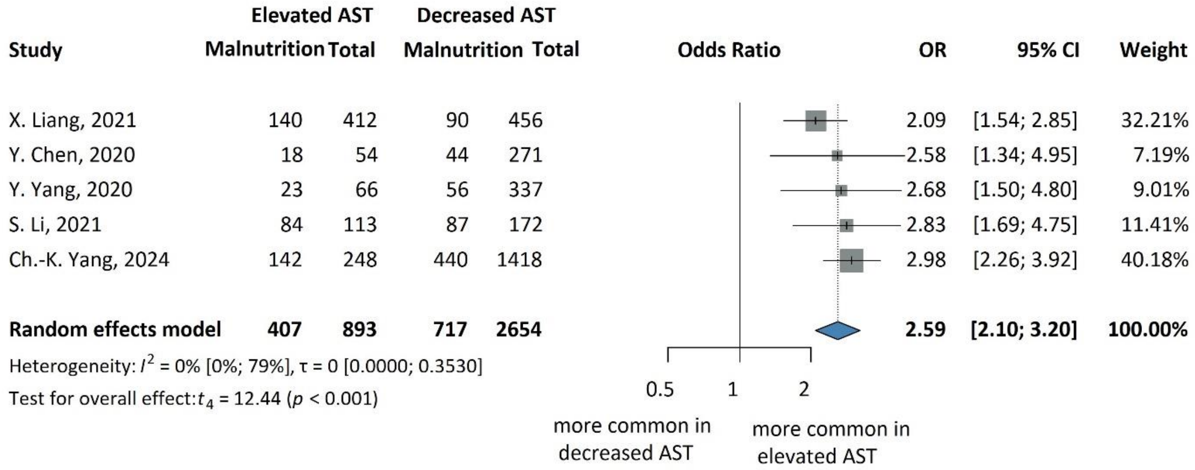

Abbreviations: AST= aspartate aminotransferase

Figure S88.: Association between malnutrition-related complication risk and anaemia in gastrointestinal cancer [subgroup analysis for gastric cancer] (Biological composite scores)

	Anaemia		No anaemia				
**Study**	**Malnutrition**	**Total**	**Malnutrition**	**Total**	**OR**	**95% CI**	**Weight**
**gastric**							
L. Chen, 2022	46	73	25	73	3.27	[1.66; 6.44]	5.28%
H. Song, 2022	51	100	24	102	3.38	[1.85; 6.18]	6.46%
X. Liu, 2018	107	209	110	488	3.60	[2.55; 5.09]	14.98%
B. Liu, 2024	23	73	8	73	3.74	[1.54; 9.06]	3.27%
H. Sun, 2022	30	73	11	73	3.93	[1.78; 8.69]	3.99%
L.-J. Wang, 2025	274	346	215	441	4.00	[2.91; 5.51]	16.44%
F. Borda, 2017	38	60	15	64	5.64	[2.58; 12.32]	4.10%
X. Zhang, 2021	221	264	86	190	6.22	[4.03; 9.59]	10.89%
J. Zuo, 2024	94	132	50	179	6.38	[3.88; 10.51]	8.81%
**Random effects model**	**884**	**1330**	**544**	**1683**	**4.33**	**[3.56; 5.27]**	**74.21%**
**Prediction interval**						**[3.47; 5.41]**	
**gastrointestinal**							
L. J. Xia, 2020_b	6	11	26	143	5.40	[1.53; 19.05]	1.68%
X. Wang, 2020b	58	114	24	167	6.17	[3.50; 10.88]	7.15%
K. Ge, 2021	110	161	60	259	7.15	[4.61; 11.11]	10.67%
X. Ma, 2025b	53	165	14	239	7.61	[4.05; 14.29]	5.97%
R. Sato, 2022	20	47	0	14	21.62	[1.22; 383.81]	0.33%
**Random effects model**	**247**	**498**	**124**	**822**	**6.94**	**[5.63; 8.56]**	**25.79%**
**Random effects model**	**1131**	**1828**	**668**	**2505**	**4.91**	**[4.09; 5.90]**	**100.00%**
**Prediction interval**						**[3.49; 6.91]**	

Gastric subgroup — Heterogeneity: I^2 = 3% [0%; 66%] , τ = 0.0390

Test for effect in subgroup: t_8 = 17.16 (p < 0.001)

Gastrointestinal subgroup — Heterogeneity: I^2 = 0% [0%; 79%] , τ = 0

Test for effect in subgroup: t_4 = 25.60 (p < 0.001)

Overall — Heterogeneity: I^2 = 23% [0%; 59%] , τ = 0.1317 [0.0000; 0.4280]

Test for overall effect: t_{13} = 18.73 (p < 0.001)

Test for subgroup differences: χ^2_1 = 17.10, df = 1 (p < 0.001)

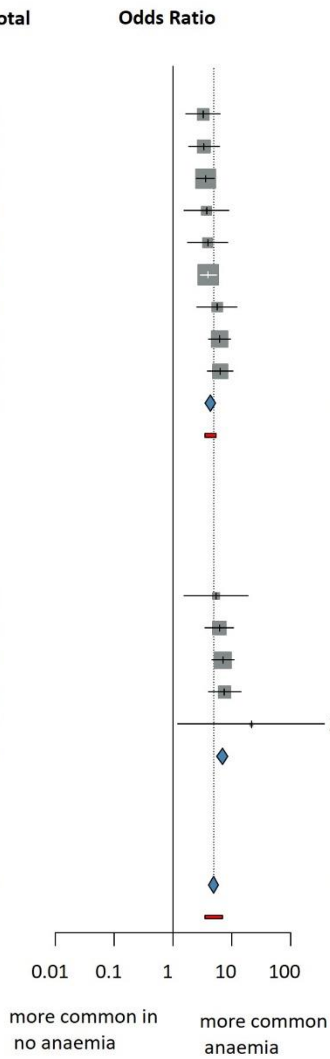

Figure S89.: Association between malnutrition-related complication risk and serum CA 19-9 level in upper-gastrointestinal cancer (Biological composite scores)

Study	Elevated CA19-9 Malnutrition	Elevated CA19-9 Total	Decreased CA19-9 Malnutrition	Decreased CA19-9 Total	OR	95% CI	Weight
K. Ge, 2021	25	70	145	350	0.79	[0.46; 1.34]	9.37%
X. Liu, 2018	51	156	166	541	1.10	[0.75; 1.61]	14.01%
L. Chen, 2022	38	74	33	72	1.25	[0.65; 2.39]	7.04%
K. Migita, 2018	29	62	39	95	1.26	[0.66; 2.40]	7.13%
Z. Xu, 2022	115	180	344	591	1.27	[0.90; 1.79]	15.45%
X. Ma, 2025	12	55	54	346	1.51	[0.75; 3.05]	6.24%
H. Sun, 2022	24	74	17	72	1.55	[0.75; 3.22]	5.88%
L.-J. Wang, 2025	87	121	403	682	1.77	[1.16; 2.71]	12.45%
X. Wang, 2023	43	74	53	121	1.78	[0.99; 3.19]	8.23%
B. Liu, 2024	20	74	11	72	2.05	[0.90; 4.67]	4.84%
D. Kuroda, 2018	19	69	43	343	2.65	[1.43; 4.92]	7.61%
K. Mimatsu, 2017	11	16	6	17	4.03	[0.94; 17.22]	1.75%
**Random effects model**	**474**	**1025**	**1314**	**3302**	**1.44**	**[1.15; 1.80]**	**100.00%**
**Prediction interval**						**[0.89; 2.31]**	

Heterogeneity: I^2 = 29% [0%; 64%], τ = 0.1874 [0.0000; 0.5650]

Test for overall effect: t_{11} = 3.59 (p = 0.004)

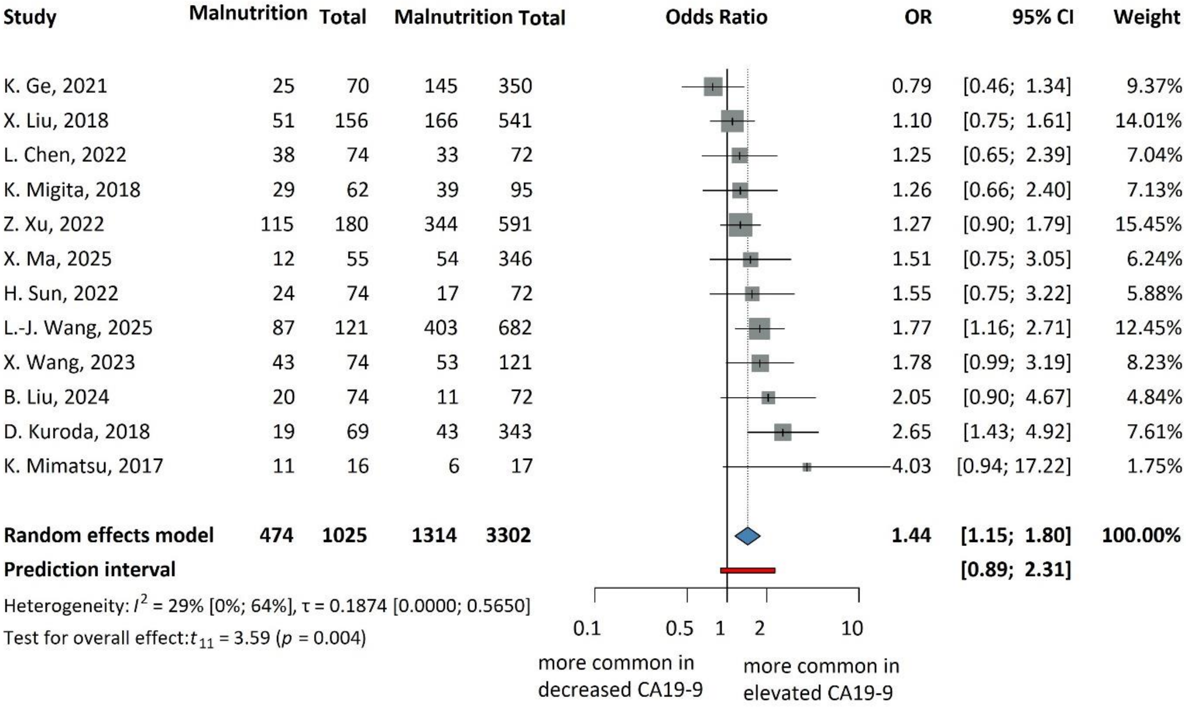

Abbreviations: CA19-9= cancer antigen 19-9

Figure S90.: Association between malnutrition-related complication risk and serum CA 19-9 level in colorectal cancer (Biological composite scores)

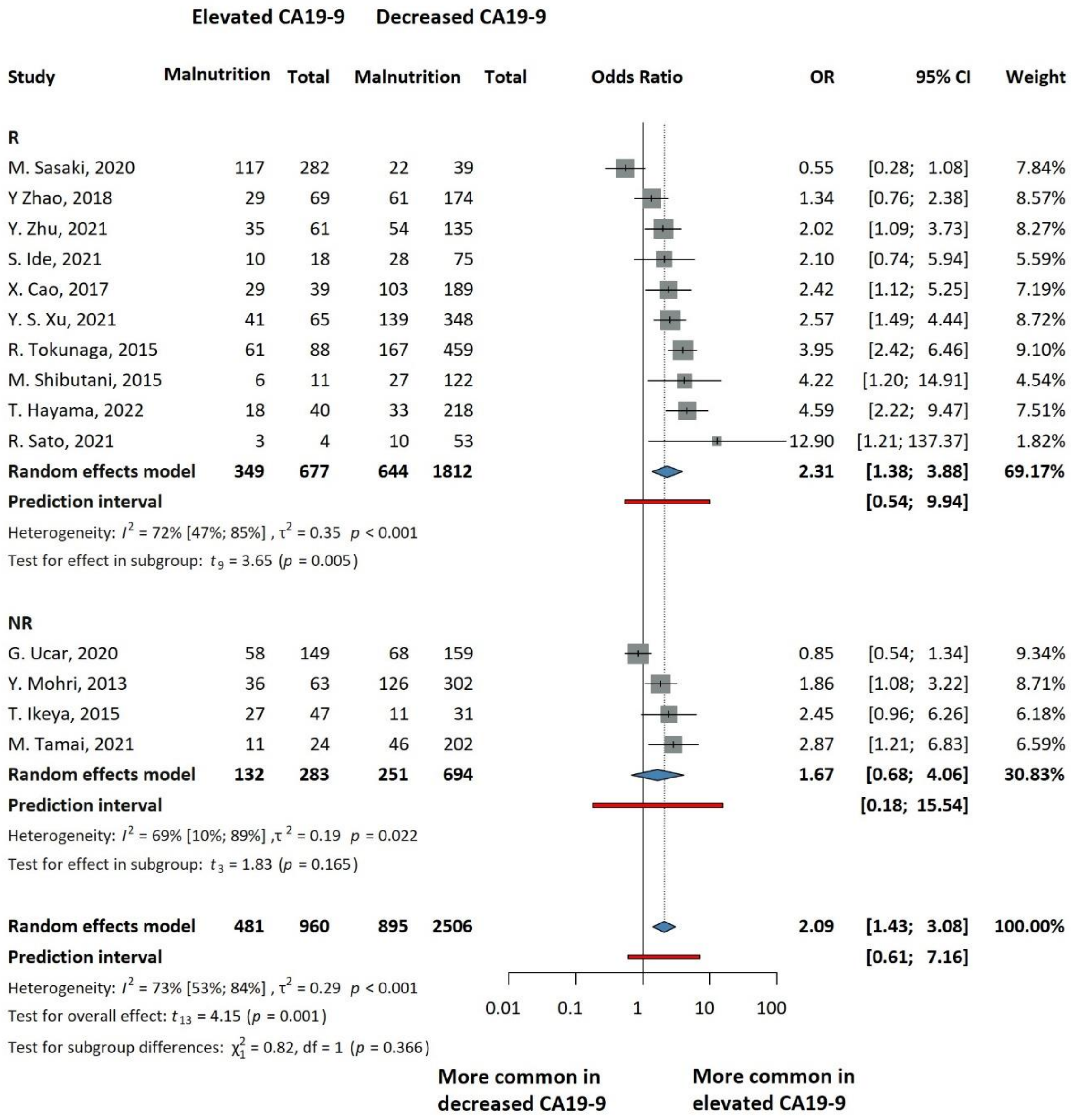

Abbreviations: CA19-9= cancer antigen 19-9; R= resectable; NR= non-resectable

Figure S91.: Association between malnutrition-related complication risk and serum CA 19-9 level in hepato-biliopancreatic cancer (Biological composite scores)

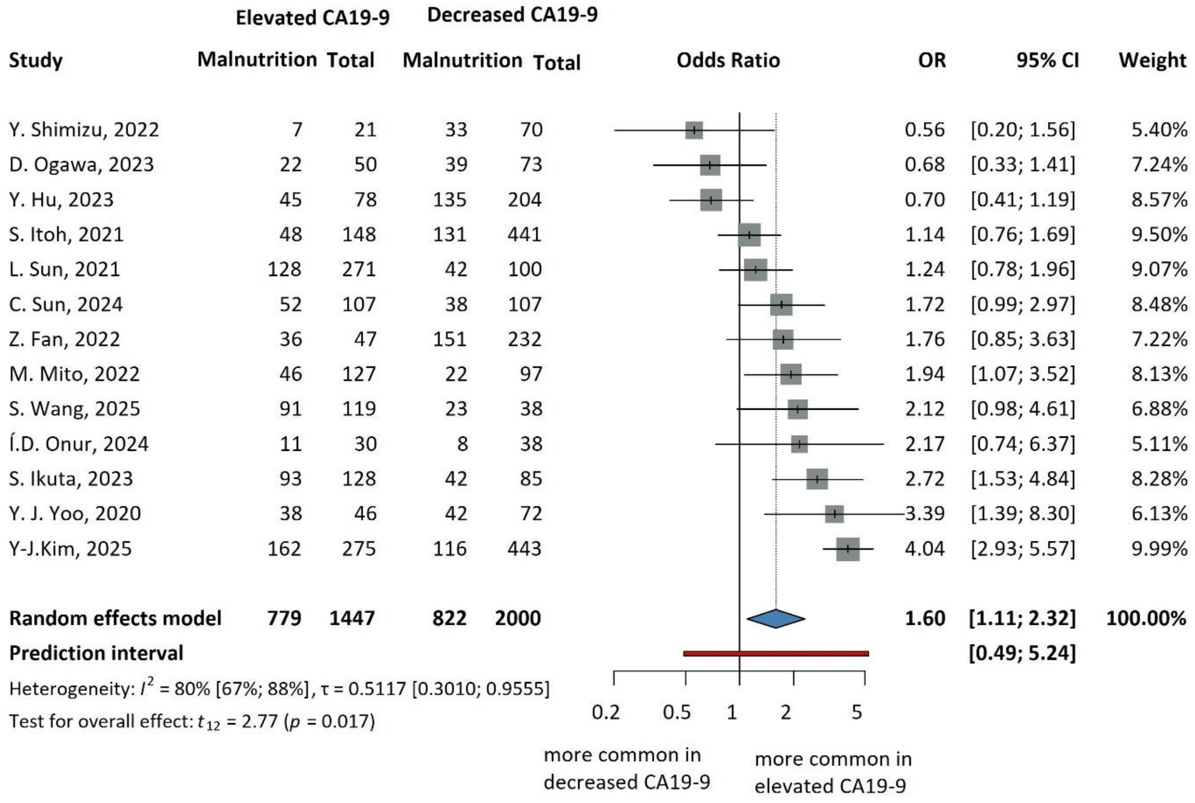

Abbreviations: CA19-9= cancer antigen 19-9

Figure S92.: Association between malnutrition-related complication risk and serum CEA level in esophageal cancer (Biological composite scores)

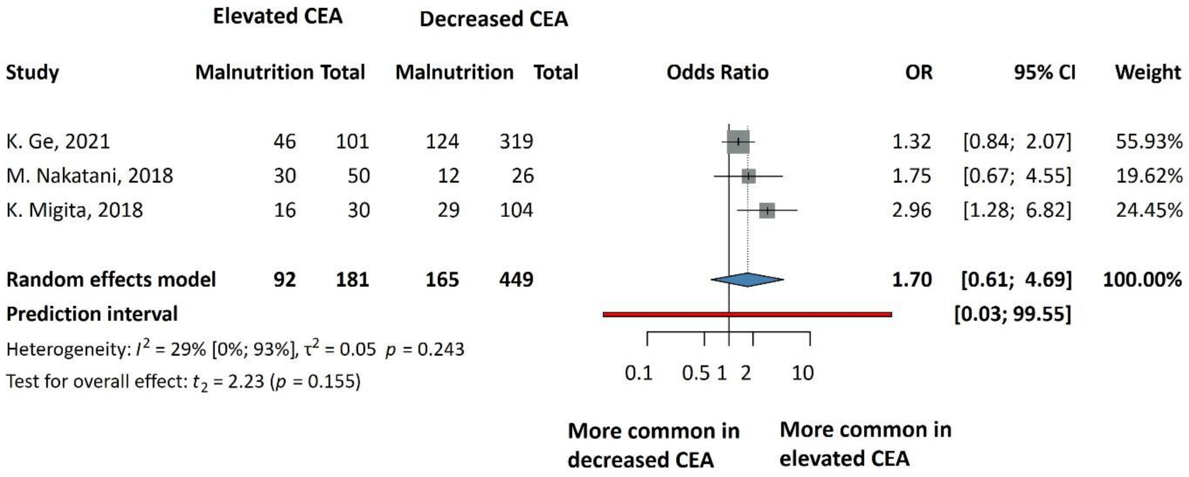

Abbreviations: CEA= carcinoembryonic antigen

Figure S93.: Association between malnutrition-related complication risk and serum CEA level in gastric cancer (Biological composite scores)

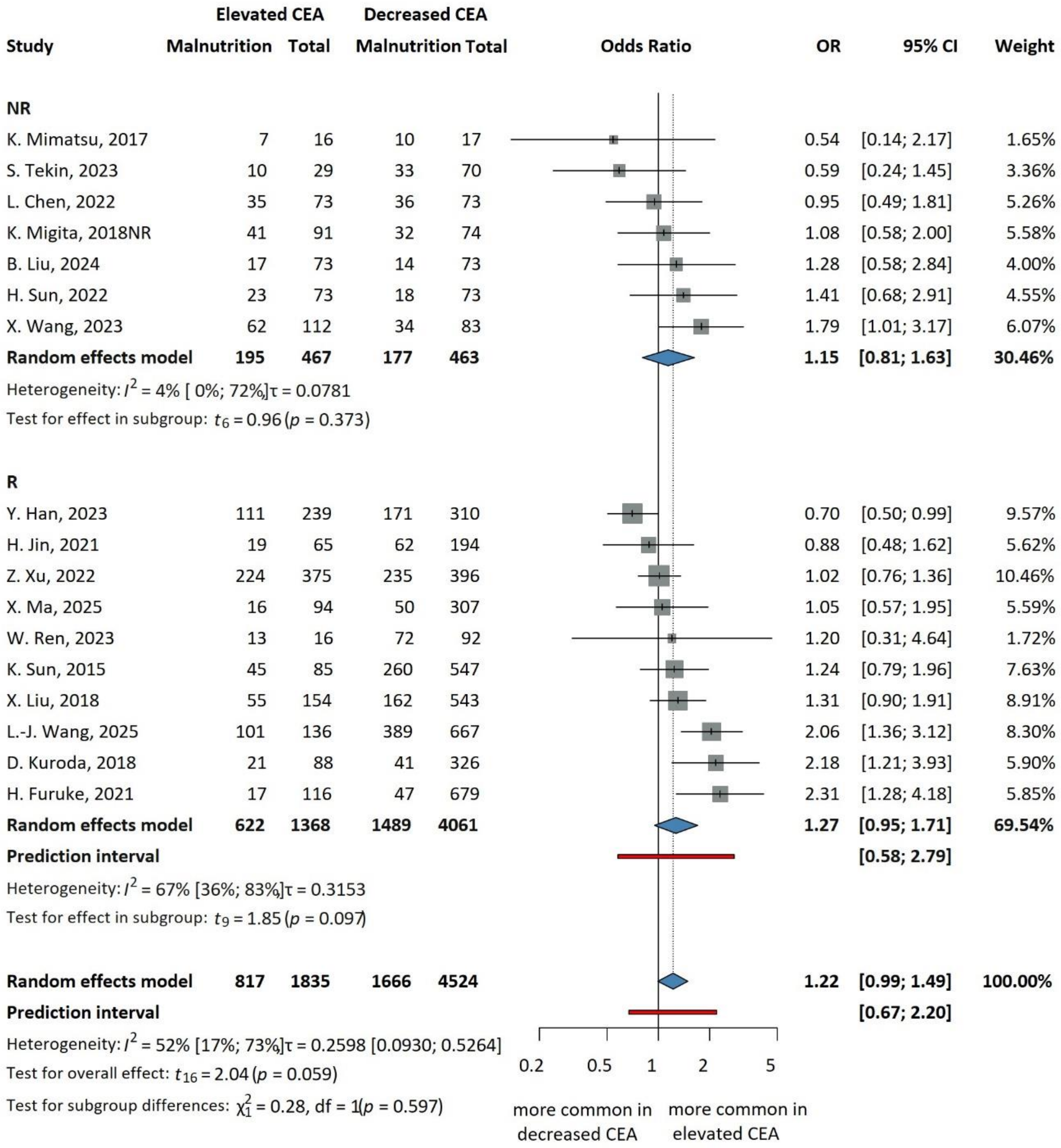

Abbreviations: CEA= carcinoembryonic antigen; R= resectable; NR= non-resectable

Figure S94.: Association between malnutrition-related complication risk and serum CEA level in colorectal cancer (Biological composite scores)

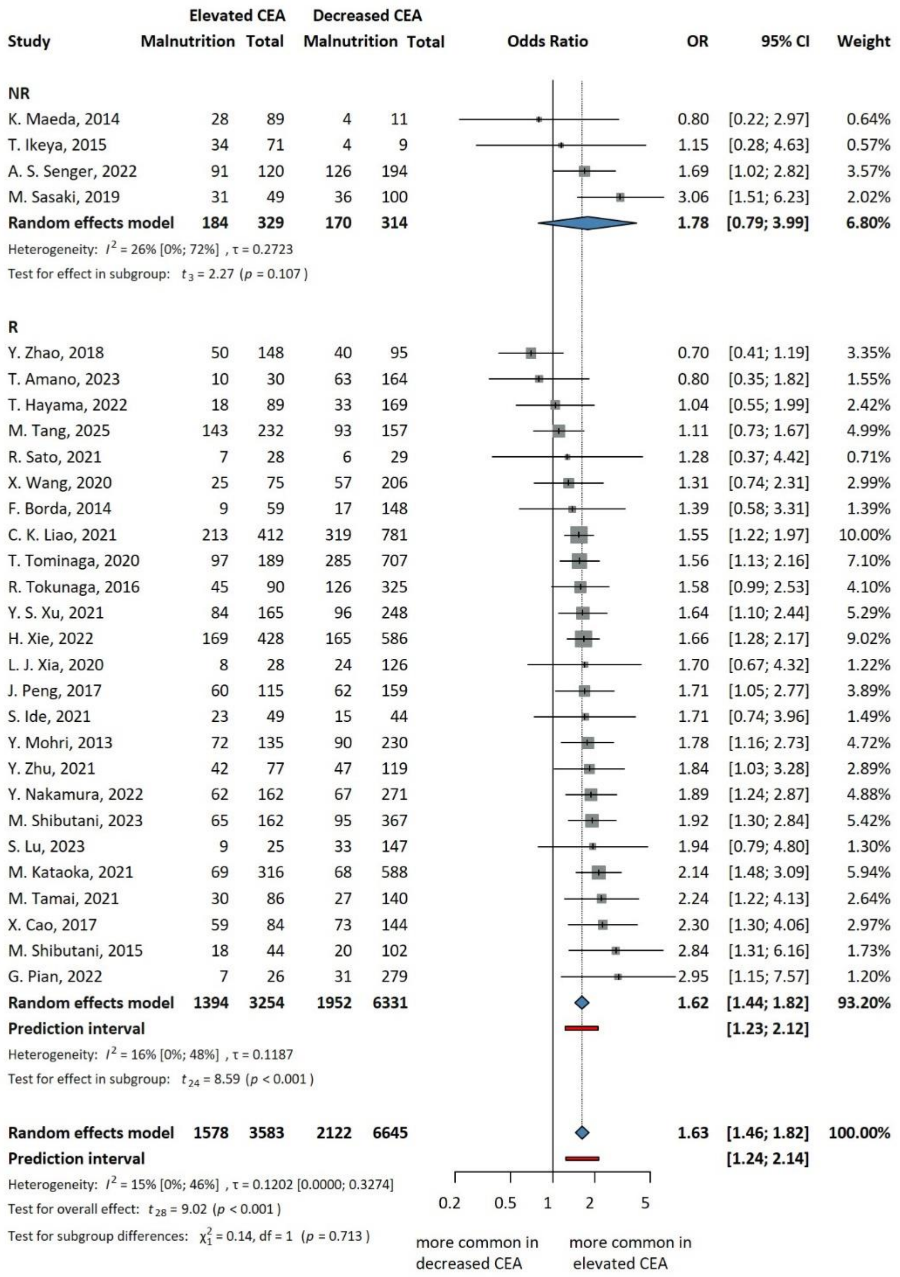

Abbreviations: CEA= carcinoembryonic antigen; R= resectable; NR= non-resectable

Figure S95.: Association between malnutrition-related complication risk and serum CEA level in hepato-biliopancreatic cancer (Biological composite scores)

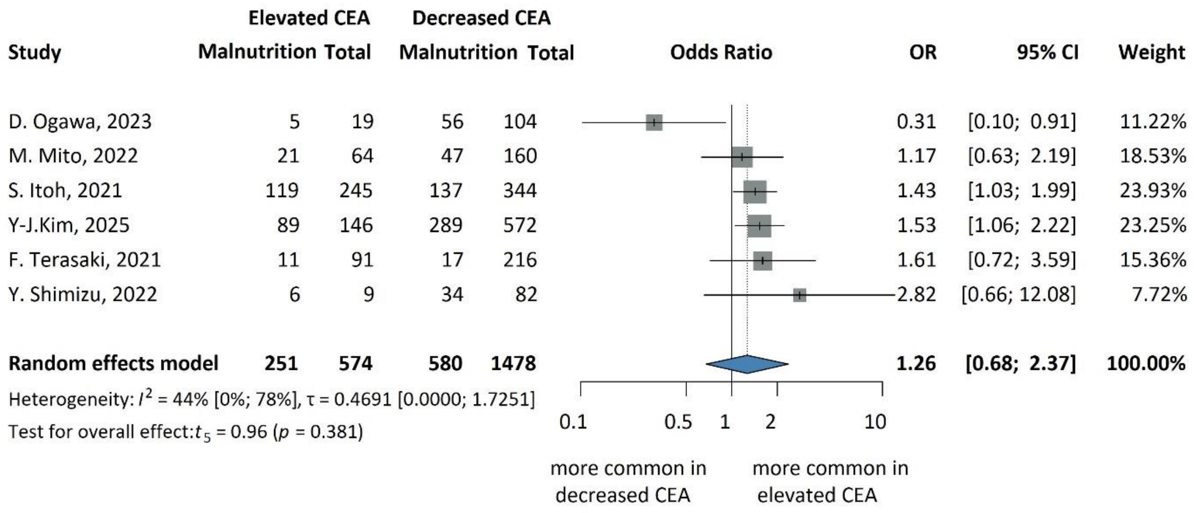

Abbreviations: CEA= carcinoembryonic antigen

Figure S96.: Association between malnutrition-related complication risk and serum GGT level in hepato-biliopancreatic cancer [subgroup analysis for hepatocellular carcinoma] (Biological composite scores)

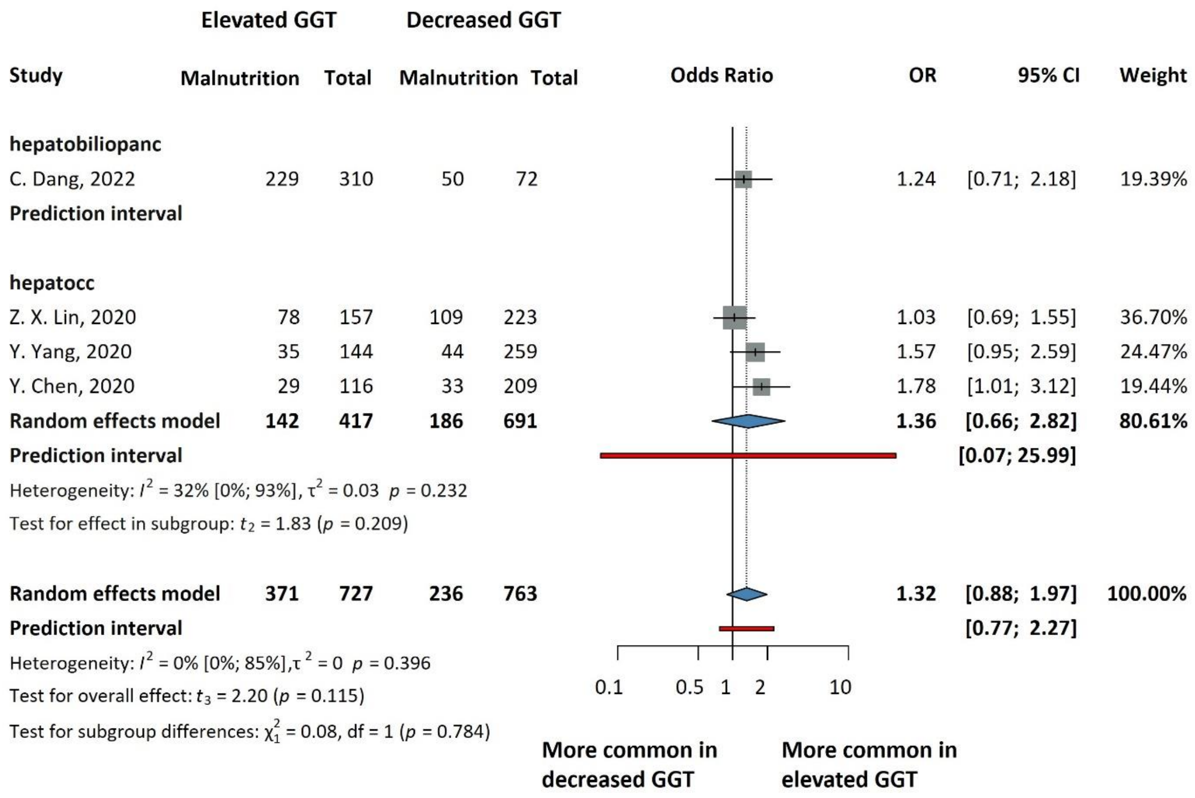

Abbreviations: GGT= gamma-glutamyl transferase

Supplementary Document S12: Individual forest plots for tumour characteristics: Figure S97-S121

Figure S97.: Association between malnutrition-related complication risk and BCLC stage in hepatocellular carcinoma (Biological composite scores)

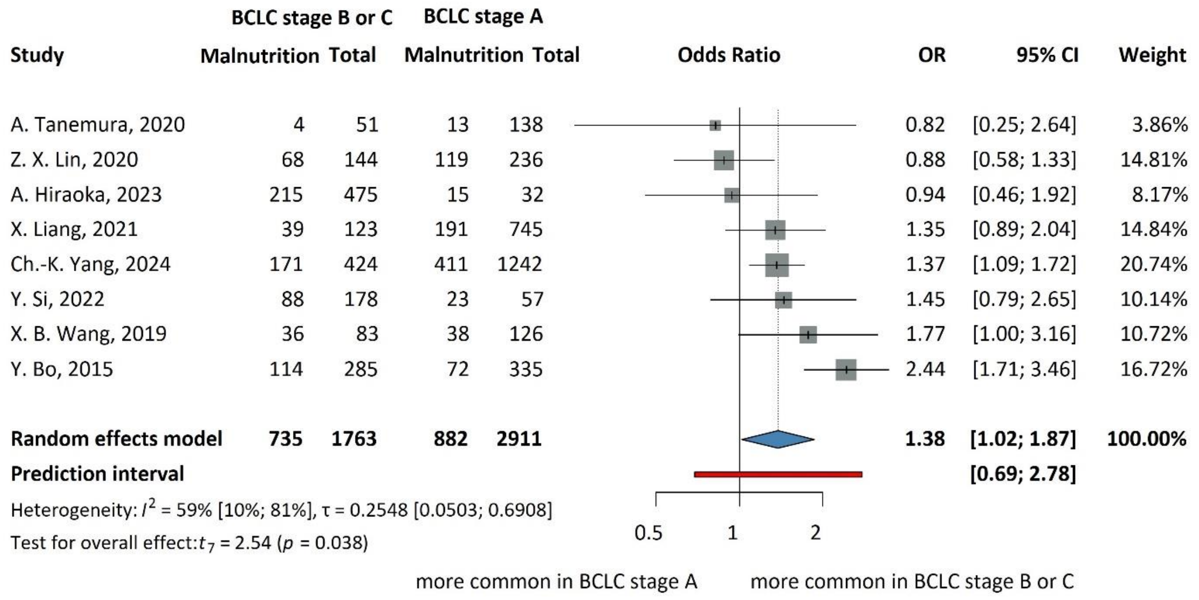

Abbreviations: BCLC= Barcelona Clinic Liver Cancer

Figure S98.: Association between malnutrition-related complication risk and BCLC stage in hepatocellular carcinoma (Biological composite scores)

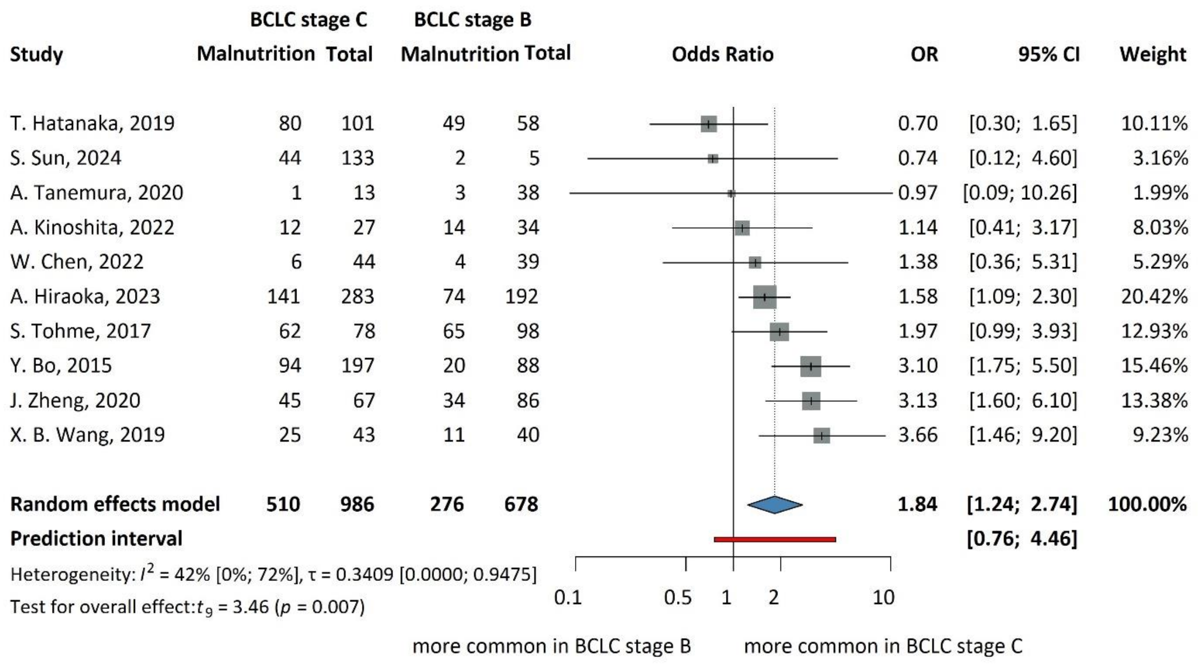

Abbreviations: BCLC= Barcelona Clinic Liver Cancer

Figure S99.: Association between malnutrition-related complication risk and Child-Pugh class in hepatocellular carcinoma (Biological composite scores)

Study	Child-Pugh class B or C Malnutrition	Child-Pugh class B or C Total	Child-Pugh class A Malnutrition	Child-Pugh class A Total	OR	95% CI	Weight
**NR**							
W. Chen, 2022	3	41	7	42	0.39	[0.09; 1.65]	2.89%
J. Zheng, 2020	16	29	63	125	1.21	[0.54; 2.73]	4.01%
Y. Chen, 2020	1	4	61	321	1.42	[0.15; 13.89]	1.76%
A. Kinoshita, 2022	5	9	21	52	1.85	[0.44; 7.69]	2.89%
Y. Yang, 2020	2	5	77	398	2.78	[0.46; 16.92]	2.31%
S. Sun, 2024	4	7	11	42	3.76	[0.72; 19.52]	2.54%
J. Liu, 2025	10	12	107	222	5.37	[1.15; 25.09]	2.70%
A. Hiraoka, 2023	33	41	209	484	5.43	[2.46; 12.00]	4.04%
S. Li, 2021	87	104	84	181	5.91	[3.26; 10.73]	4.38%
S. Tohme, 2017	19	20	62	82	6.13	[0.77; 48.72]	1.98%
Y. Si, 2022	60	79	51	156	6.50	[3.52; 12.02]	4.35%
T. Hatanaka, 2019	25	26	120	152	6.67	[0.87; 51.09]	2.02%
Y. Imaoka, 2023	76	108	148	1386	19.87	[12.71; 31.06]	4.60%
M. O. Chu, 2018	28	31	20	87	31.27	[8.60; 113.72]	3.12%
**Random effects model**	**369**	**516**	**1041**	**3730**	**4.45**	**[2.30; 8.60]**	**43.60%**
**Prediction interval**						**[0.53; 37.25]**	
Heterogeneity: I^2 = 81% [68%; 88%] τ = 0.9263							
Test for effect in subgroup: t_{13} = 4.89 (p < 0.001)							
**R**							
J. Li, 2023	8	40	12	60	1.00	[0.37; 2.72]	3.66%
K. Takagi, 2017	7	14	288	576	1.00	[0.35; 2.89]	3.54%
L. Li, 2018	22	39	637	1295	1.34	[0.70; 2.54]	4.30%
H. Hayashi, 2023	6	8	144	295	3.15	[0.62; 15.84]	2.59%
H. Jeong, 2021	10	12	67	118	3.81	[0.80; 18.13]	2.67%
Y. Tamai, 2022	18	24	55	157	5.56	[2.09; 14.83]	3.69%
Y. Bo, 2015	47	66	139	554	7.39	[4.19; 13.01]	4.43%
M. Tsukagoshi, 2024	4	6	41	197	7.61	[1.35; 43.00]	2.42%
Ch.-K. Yang, 2024	53	64	529	1602	9.77	[5.06; 18.86]	4.28%
X. B. Wang, 2019	29	37	45	172	10.23	[4.36; 24.02]	3.93%
H. Kanno, 2021	10	11	163	335	10.55	[1.34; 83.35]	1.99%
T. Ryu, 2022	38	44	109	297	10.92	[4.47; 26.67]	3.86%
N. Harimoto, 2018	119	161	421	2300	12.65	[8.76; 18.26]	4.70%
A. Fujio, 2022	5	6	14	55	14.64	[1.57; 136.33]	1.81%
A. Tanemura, 2020	6	11	11	178	18.22	[4.80; 69.19]	3.05%
Z. Qu, 2022	25	27	71	188	20.60	[4.74; 89.60]	2.82%
M. Tsunematsu, 2021	6	9	8	102	23.50	[4.93; 112.13]	2.67%
**Random effects model**	**413**	**579**	**2754**	**8481**	**6.22**	**[3.67; 10.55]**	**56.40%**
**Prediction interval**						**[0.98; 39.72]**	
Heterogeneity: I^2 = 78% [66%; 86%] τ = 0.8331							
Test for effect in subgroup: t_{16} = 7.34 (p < 0.001)							
**Random effects model**	**782**	**1095**	**3795**	**12211**	**5.40**	**[3.65; 8.01]**	**100.00%**
**Prediction interval**						**[0.87; 33.37]**	

Heterogeneity: I^2 = 79% [70%; 85%] τ = 0.8690 [0.6080; 1.2784]

Test for overall effect: t_{30} = 8.75 (p < 0.001)

Test for subgroup differences: χ^2_1 = 0.73, df = 1 (p = 0.394)

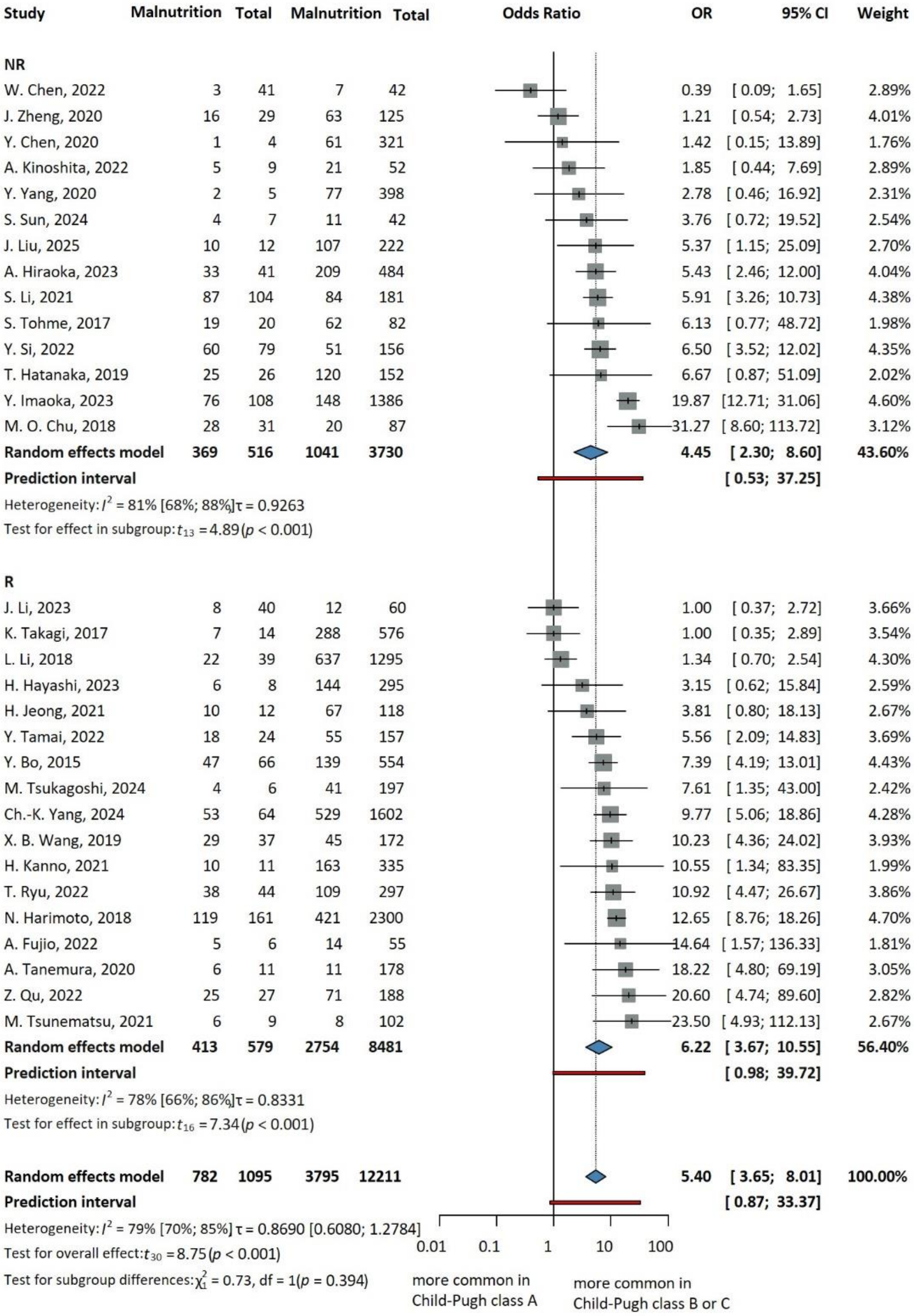

Abbreviations: R= resectable; NR= non-resectable

Figure S100.: Association between malnutrition-related complication risk and N stage in esophageal squamous cell carcinoma (Biological composite scores)

Study	N≥1 stage Malnutrition	N≥1 stage Total	N0 stage Malnutrition	N0 stage Total	OR	95% CI	Weight
L. Xiao, 2021	76	192	1	1	0.22	[0.01; 5.44]	0.49%
Y. Zhao, 2022	155	247	69	107	0.93	[0.58; 1.49]	9.93%
J. Feng, 2022	59	85	91	131	1.00	[0.55; 1.80]	7.97%
K. Yan, 2022	64	119	39	73	1.01	[0.57; 1.82]	8.09%
Y. Dai, 2019	32	66	18	40	1.15	[0.52; 2.53]	5.63%
P. Fang, 2024	31	117	36	197	1.61	[0.93; 2.79]	8.67%
J. F. Feng, 2013	135	174	135	201	1.69	[1.07; 2.69]	10.12%
Y. Fujiwara, 2021	15	66	6	45	1.91	[0.68; 5.38]	3.78%
K. Takao, 2020	54	123	12	42	1.96	[0.92; 4.18]	5.92%
I. Hashimoto, 2024	29	125	10	78	2.05	[0.94; 4.49]	5.68%
F. K. Xiao, 2021	744	1763	616	2383	2.09	[1.84; 2.39]	16.44%
I. Nonogaki, 2023	123	253	63	211	2.22	[1.51; 3.26]	11.62%
L. Han, 2016	23	85	11	121	3.71	[1.70; 8.12]	5.68%
**Random effects model**	**1540**	**3415**	**1107**	**3630**	**1.62**	**[1.26; 2.09]**	**100.00%**
**Prediction interval**						**[0.83; 3.14]**	

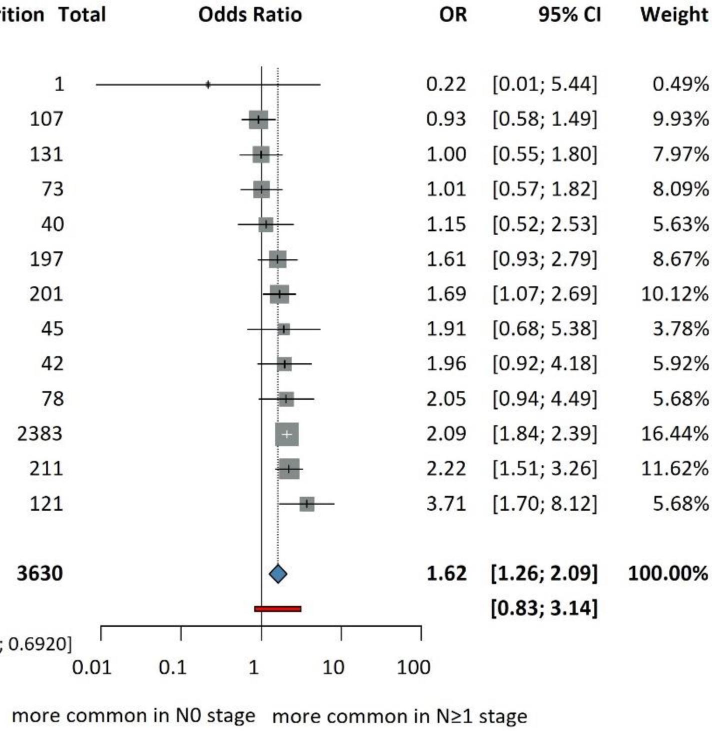

Heterogeneity: I^2 = 56% [18%; 76%], τ = 0.2780 [0.0784; 0.6920]

Test for overall effect: t_{12} = 4.16 (p = 0.001)

Figure S101.: Association between malnutrition-related complication risk and N stage in gastric cancer (Biological composite scores)

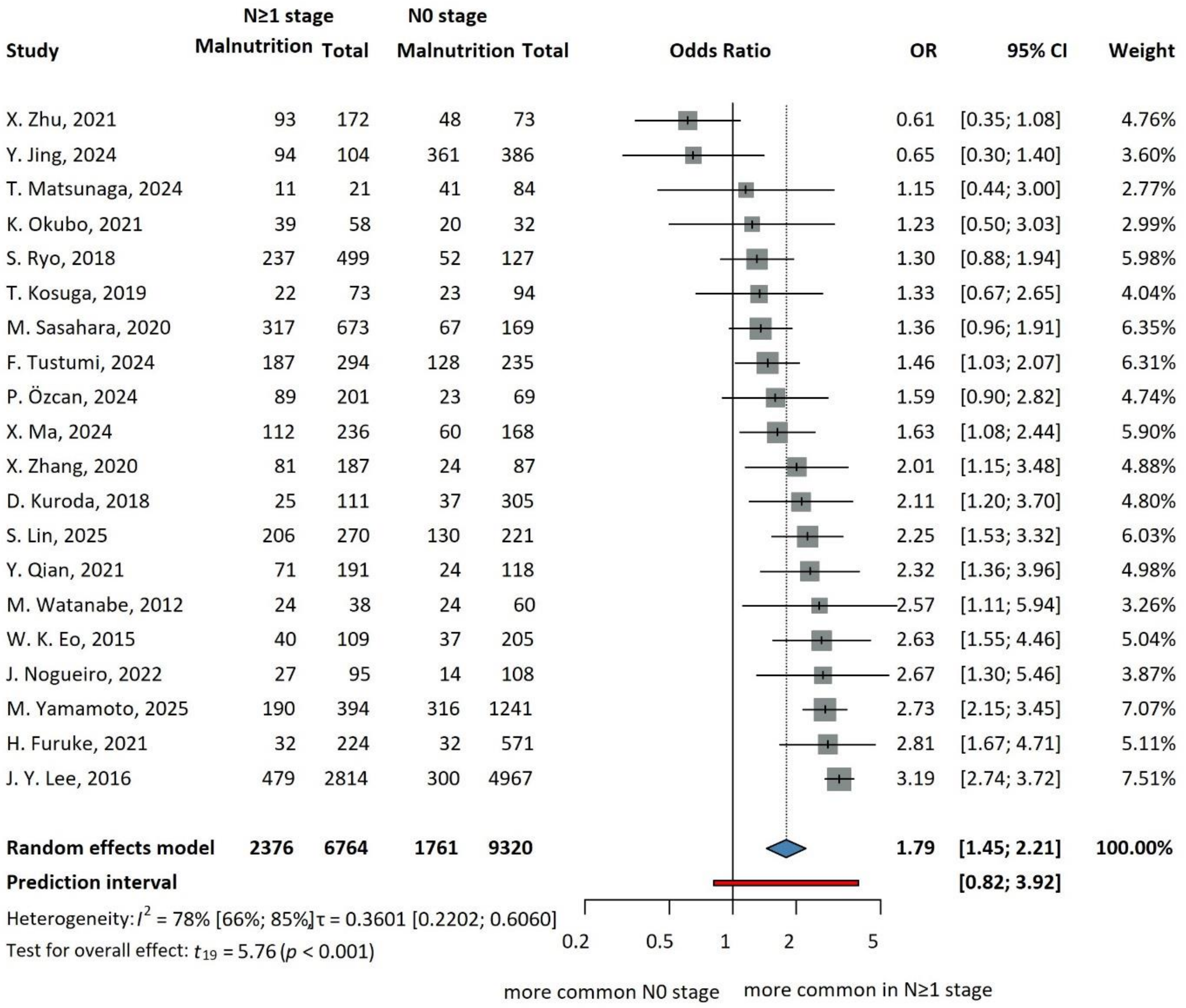

Figure S102.: Association between malnutrition-related complication risk and N stage in colorectal cancer (Biological composite scores)

Study	N≥1 stage Events	N≥1 stage Total	N0 stage Events	N0 stage Total	OR	95% CI	Weight
H. Xie, 2022	143	465	191	551	0.84	[0.64; 1.09]	10.60%
N. Kurtul, 2017	11	25	17	37	0.92	[0.33; 2.57]	2.27%
S. Lee, 2023	34	96	13	35	0.93	[0.42; 2.07]	3.34%
T. Hayama, 2020	66	189	40	112	0.97	[0.59; 1.57]	6.47%
Y. Zhao, 2018	51	138	39	105	0.99	[0.59; 1.68]	5.96%
X. Zhu, 2025	33	85	45	116	1.00	[0.56; 1.78]	5.35%
X. Wang, 2020	36	123	46	158	1.01	[0.60; 1.69]	6.05%
K. Li, 2024	66	188	89	282	1.17	[0.79; 1.73]	8.07%
C. Akgul, 2024	47	124	58	176	1.24	[0.77; 2.01]	6.60%
T. Iguchi, 2020	19	48	11	32	1.25	[0.49; 3.17]	2.64%
T. Tominaga, 2019	40	82	72	168	1.27	[0.75; 2.16]	5.90%
C. Jian-Hui, 2016	373	843	176	478	1.36	[1.08; 1.71]	11.30%
Y. Nakamura, 2023	77	226	103	375	1.36	[0.96; 1.95]	8.71%
J. Li, 2023	45	132	66	264	1.55	[0.98; 2.45]	6.98%
R. Sato, 2022	14	34	6	27	2.45	[0.79; 7.63]	1.89%
L. J. Xia, 2020	8	22	24	132	2.57	[0.97; 6.82]	2.45%
X. Cao, 2017	74	100	58	128	3.44	[1.95; 6.05]	5.44%
**Random effects model**	**1137**	**2920**	**1054**	**3176**	**1.25**	**[1.04; 1.50]**	**100.00%**
**Prediction interval**						**[0.75; 2.09]**	

Heterogeneity: I^2 = 47% [6%; 70%], τ = 0.2256 [0.0401; 0.4911]

Test for overall effect: t_{16} = 2.59 (p = 0.020)

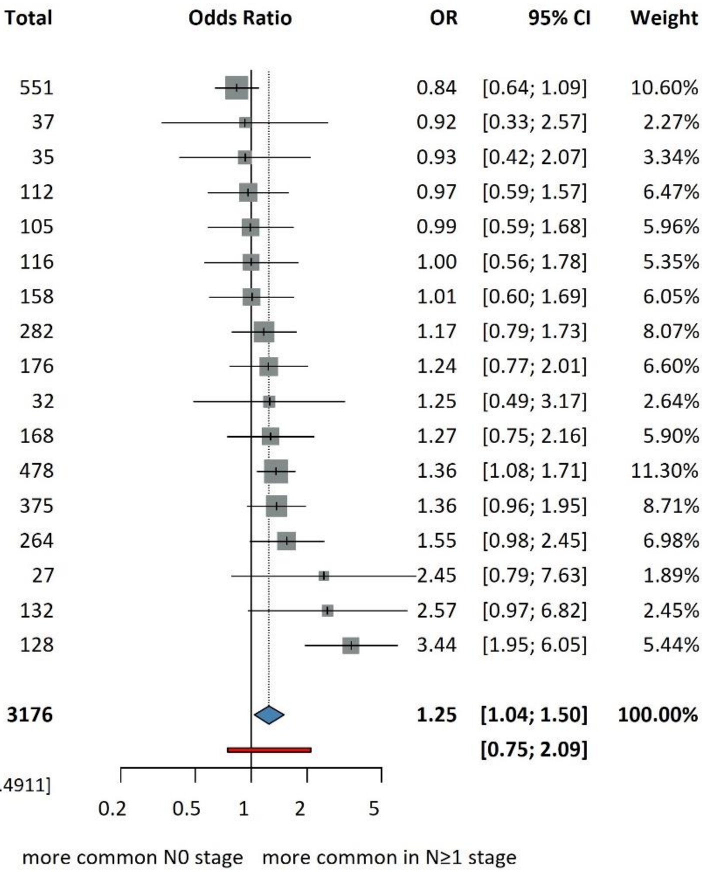

Figure S103.: Association between malnutrition-related complication risk and T stage in esophageal cancer (Biological composite scores)

	T>1 stage		T≤1 stage				
**Study**	**Malnutrition**	**Total**	**Malnutrition**	**Total**	**OR**	**95% CI**	**Weight**
F. K. Xiao, 2021	1171	3500	189	646	1.22	[1.01; 1.46]	23.97%
M. Urabe, 2021	55	106	48	118	1.57	[0.93; 2.67]	8.36%
H. Zhang, 2018	245	622	9	33	1.73	[0.79; 3.79]	4.45%
Q. Qi, 2021	118	354	9	42	1.83	[0.85; 3.96]	4.58%
S. B. Chen, 2021	263	547	24	73	1.89	[1.13; 3.17]	8.67%
I. Hashimoto, 2024	38	194	1	9	1.95	[0.24; 16.06]	0.70%
G. H. A-Lai, 2019	38	71	7	19	1.97	[0.70; 5.60]	2.67%
L. Han, 2016	32	185	2	21	1.99	[0.44; 8.96]	1.34%
J. F. Feng, 2013	233	312	37	63	2.07	[1.18; 3.64]	7.63%
N. Kubo, 2018	30	127	14	113	2.19	[1.09; 4.38]	5.47%
T.Toyokawa, 2016	13	112	4	73	2.27	[0.71; 7.24]	2.19%
Y. Fujiwara, 2021	14	56	7	55	2.29	[0.84; 6.20]	2.90%
I. Nonogaki, 2023	149	322	37	142	2.44	[1.58; 3.77]	11.04%
T. Miyazaki, 2016	46	108	19	84	2.54	[1.34; 4.80]	6.27%
Z. Zheng, 2022	64	130	8	35	3.27	[1.38; 7.74]	3.77%
N. Hirahara, 2018	71	97	27	72	4.55	[2.36; 8.77]	6.00%
**Random effects model**	**2580**	**6843**	**442**	**1598**	**1.93**	**[1.59; 2.34]**	**100.00%**
**Prediction interval**						**[1.30; 2.86]**	

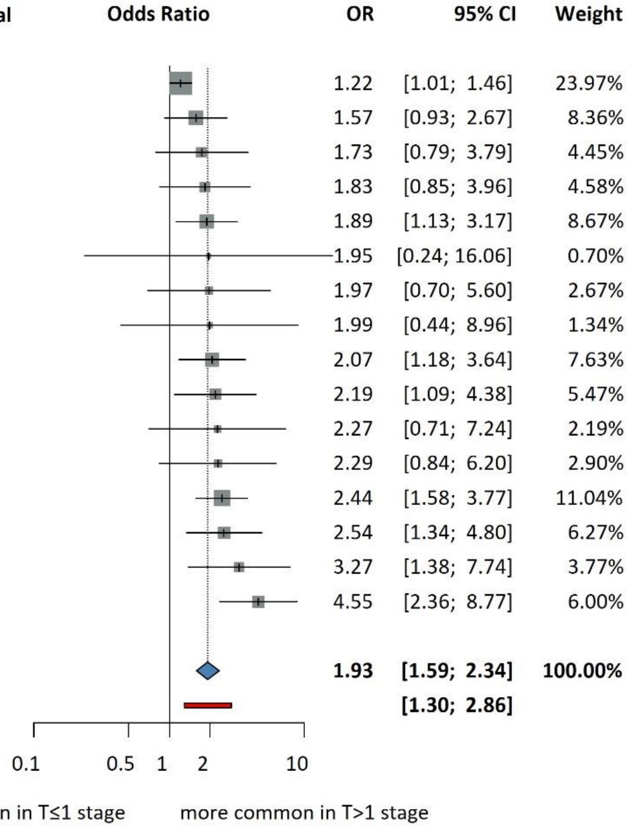

Heterogeneity: I^2 = 50% [10%; 72%], τ = 0.1601 [0.0414; 0.3590]

Test for overall effect: t_{15} = 7.24 (p < 0.001)

Figure S104.: Association between malnutrition-related complication risk and T stage in esophageal cancer (Biological composite scores)

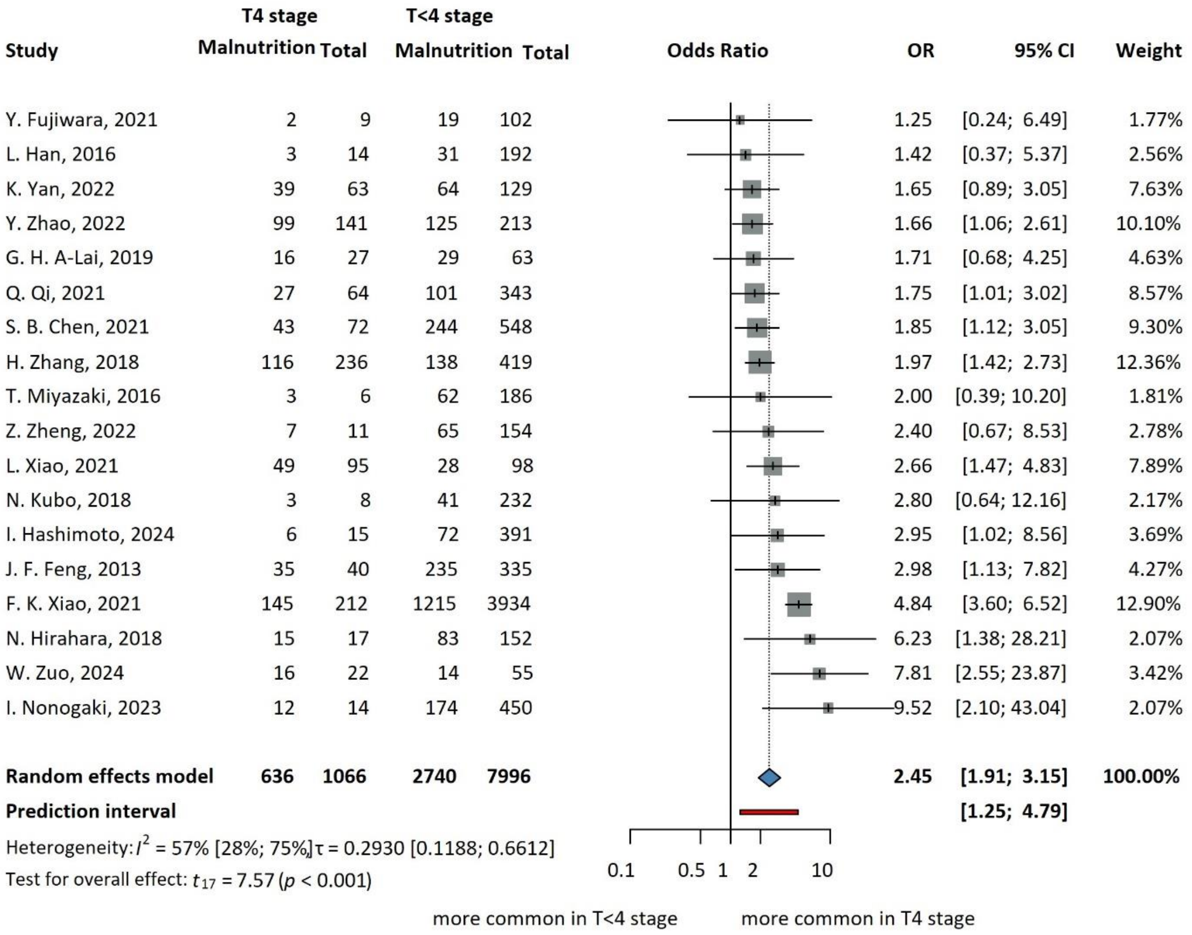

Figure S105.: Association between malnutrition-related complication risk and T stage in gastric cancer (Biological composite scores)

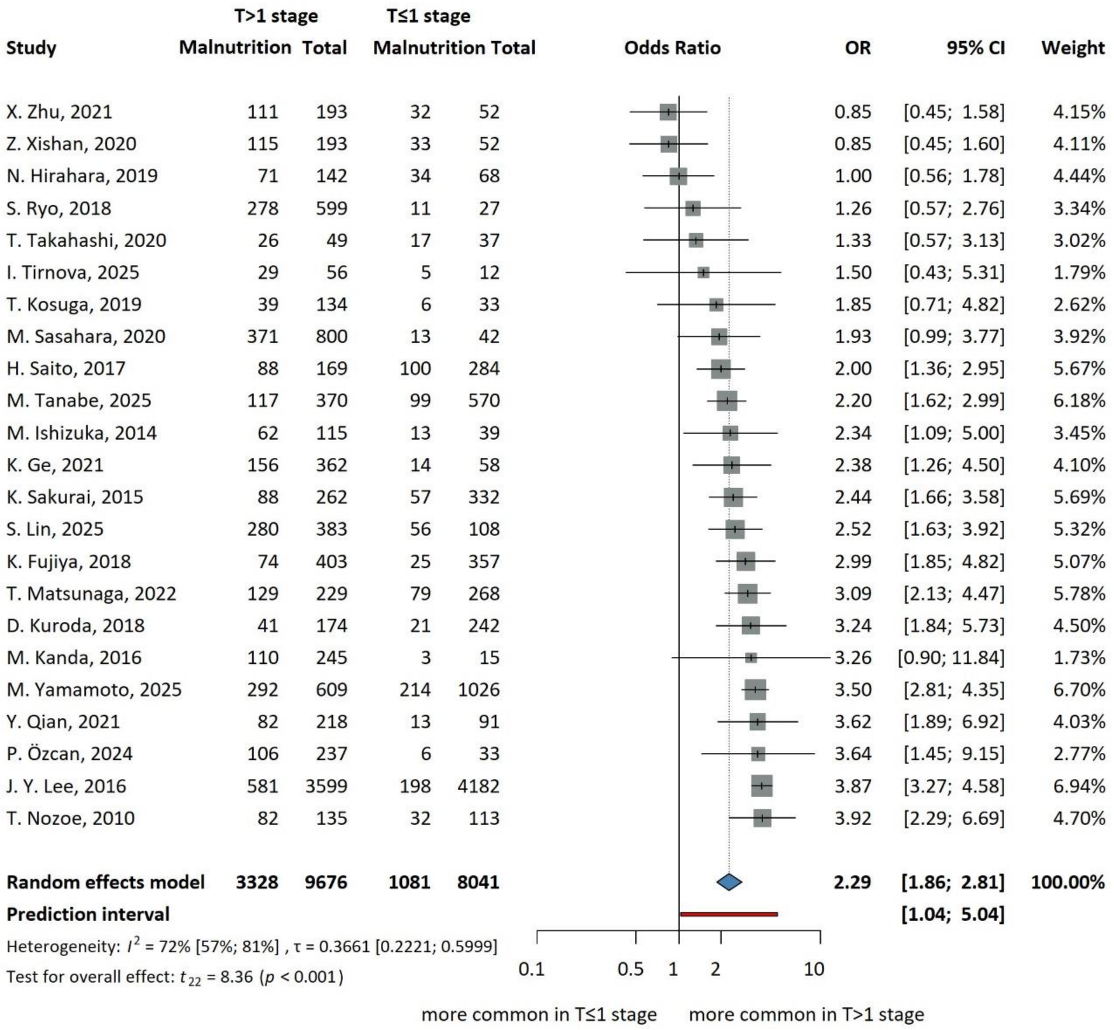

Figure S106.: Association between malnutrition-related complication risk and T stage in gastric cancer (Biological composite scores)

Study	T4 stage Malnutrition	T4 stage Total	T<4 stage Malnutrition	T<4 stage Total	Odds Ratio	OR	95% CI	Weight
Z. Xishan, 2020	42	72	106	173		0.88	[0.51; 1.55]	4.65%
X. Zhu, 2021	41	72	102	173		0.92	[0.53; 1.61]	4.67%
Z. Luo, 2019	45	87	19	41		1.24	[0.59; 2.61]	3.56%
S. Ryo, 2018	134	260	155	366		1.45	[1.05; 1.99]	6.35%
M. Kanda, 2016	53	108	60	152		1.48	[0.90; 2.43]	5.07%
Y. Qian, 2021	50	141	45	168		1.50	[0.92; 2.44]	5.16%
H. Jin, 2021	65	186	20	83		1.69	[0.94; 3.04]	4.47%
M. Sasahara, 2020	194	363	190	479		1.75	[1.33; 2.30]	6.65%
T. Matsunaga, 2022	29	52	179	445		1.87	[1.05; 3.34]	4.52%
T. Takahashi, 2020	7	11	36	75		1.90	[0.51; 7.02]	1.67%
T. Kosuga, 2019	17	46	28	121		1.95	[0.94; 4.05]	3.62%
P. Özcan, 2024	85	179	27	91		2.14	[1.25; 3.67]	4.80%
N. Hirahara, 2020	30	64	67	233		2.19	[1.24; 3.85]	4.60%
K. Sakurai, 2015	45	116	100	478		2.40	[1.55; 3.70]	5.53%
S. Lin, 2025	232	299	104	192		2.93	[1.98; 4.34]	5.82%
D. Kuroda, 2018	12	38	50	378		3.03	[1.44; 6.38]	3.55%
J. Y. Lee, 2016	357	1742	422	6039		3.43	[2.95; 4.00]	7.35%
B. Akagunduz, 2022	41	49	64	109		3.60	[1.54; 8.41]	3.07%
K. Fujiya, 2018	38	135	61	625		3.62	[2.29; 5.73]	5.34%
I. Tirnova, 2025	18	26	16	42		3.66	[1.29; 10.34]	2.35%
M. Ishizuka, 2014	7	9	68	145		3.96	[0.80; 19.73]	1.20%
M. Yamamoto, 2025	84	134	422	1501		4.30	[2.97; 6.20]	6.00%
**Random effects model**	**1626**	**4189**	**2341**	**12109**		**2.10**	**[1.71; 2.57]**	**100.00%**
**Prediction interval**							**[0.98; 4.50]**	

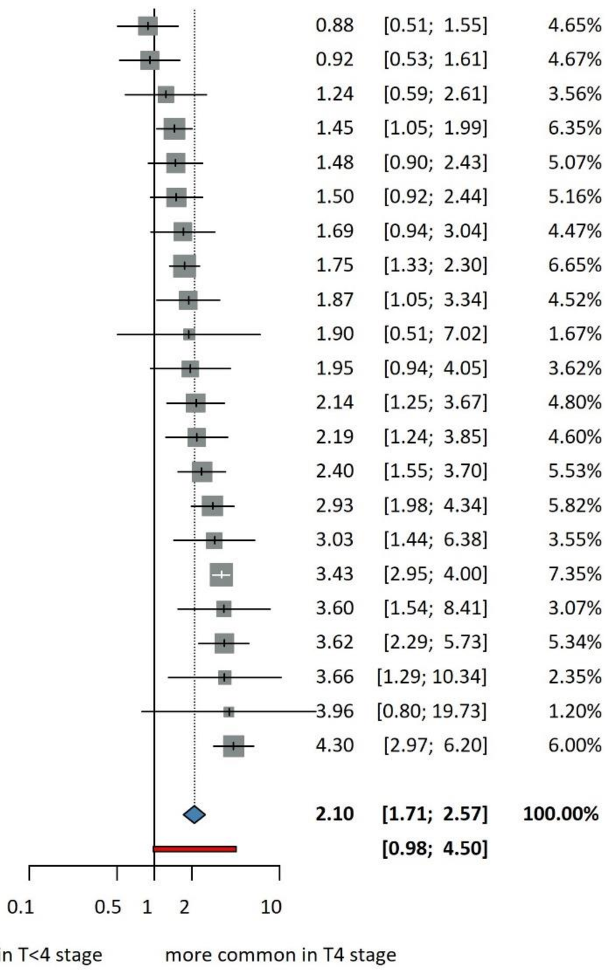

Heterogeneity: I^2 = 76% [64%; 84%], τ = 0.3515 [0.2213; 0.5752]

Test for overall effect: t_{21} = 7.60 (p < 0.001)

Figure S107.: Association between malnutrition-related complication risk and T stage in colorectal cancer (Biological composite scores)

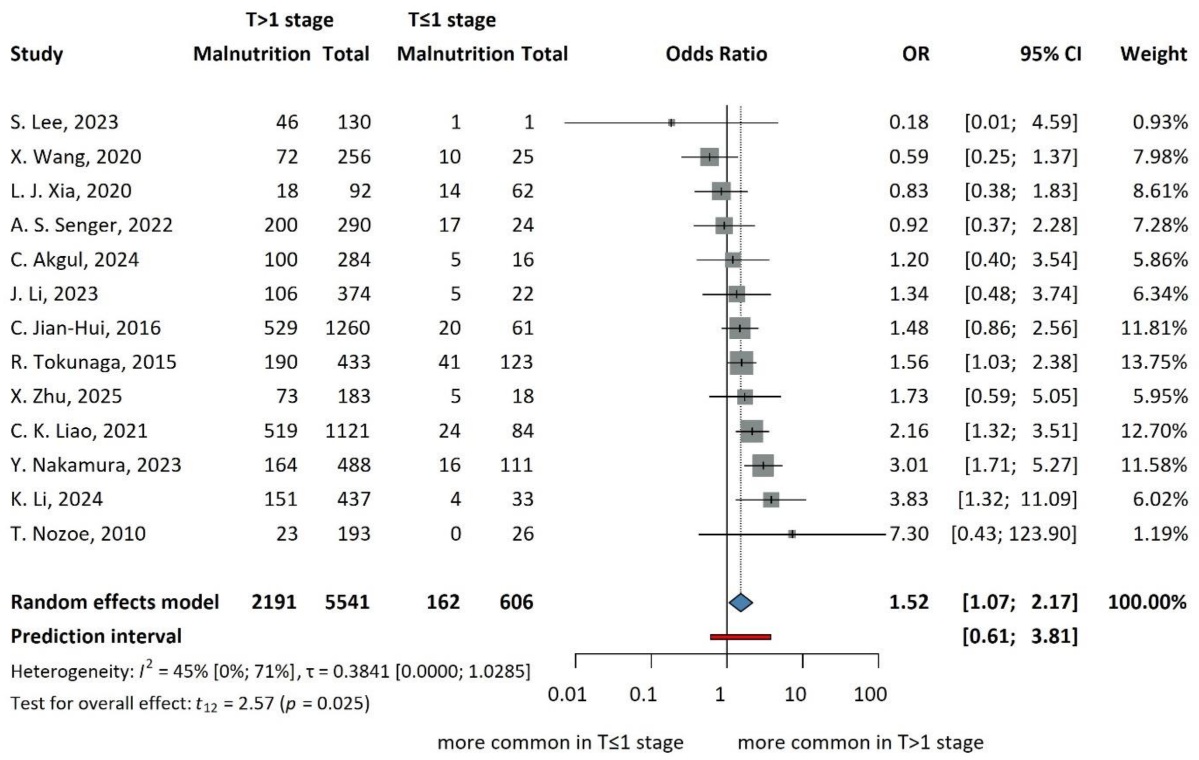

Figure S108.: Association between malnutrition-related complication risk and T stage in colorectal cancer (Biological composite scores)

Study	T4 stage Malnutrition	T4 stage Total	T<4 stage Malnutrition	T<4 stage Total	OR	95% CI	Weight
R. Sato, 2021	2	13	11	44	0.55	[0.10; 2.85]	1.38%
J. Li, 2023	44	181	67	215	0.71	[0.45; 1.11]	6.27%
X. Wang, 2020	8	34	74	247	0.72	[0.31; 1.66]	3.68%
Y. Zhao, 2018	28	84	62	159	0.78	[0.45; 1.36]	5.44%
X. Zhu, 2025	5	12	73	189	1.14	[0.35; 3.71]	2.34%
J. Peng, 2017	65	141	57	133	1.14	[0.71; 1.84]	6.03%
S. Lee, 2023	28	75	19	56	1.16	[0.56; 2.39]	4.31%
K. Maeda, 2014	24	72	8	28	1.25	[0.48; 3.25]	3.14%
T. Amano, 2023	19	39	75	198	1.56	[0.78; 3.11]	4.52%
Y. Iseki, 2015	22	67	32	137	1.60	[0.84; 3.06]	4.81%
M. Shibutani, 2023	21	52	139	477	1.65	[0.91; 2.97]	5.20%
N. Kurtul, 2017	4	7	24	56	1.78	[0.36; 8.70]	1.48%
C. Jian-Hui, 2016	163	299	386	1022	1.97	[1.52; 2.56]	7.67%
A. S. Senger, 2022	38	47	179	267	2.08	[0.96; 4.48]	4.04%
C. Akgul, 2024	28	55	77	245	2.26	[1.25; 4.10]	5.16%
M. Shibutani, 2015	18	50	21	108	2.33	[1.10; 4.93]	4.17%
M. Tamai, 2021	19	47	38	176	2.46	[1.24; 4.89]	4.56%
Y. Nakamura, 2023	51	105	129	494	2.67	[1.73; 4.12]	6.38%
C. K. Liao, 2021	107	161	436	1044	2.76	[1.95; 3.92]	7.02%
T. Yagyu, 2022	49	63	153	285	3.02	[1.60; 5.71]	4.86%
K. Li, 2024	18	31	137	439	3.05	[1.45; 6.41]	4.21%
T. Tominaga, 2019	18	25	94	225	3.58	[1.44; 8.92]	3.33%
**Random effects model**	**779**	**1660**	**2291**	**6244**	**1.69**	**[1.35; 2.11]**	**100.00%**
**Prediction interval**						**[0.76; 3.75]**	

Heterogeneity: I^2 = 63% [41%; 77%], τ = 0.3671 [0.2055; 0.6329]

Test for overall effect: t_{21} = 4.84 (p < 0.001)

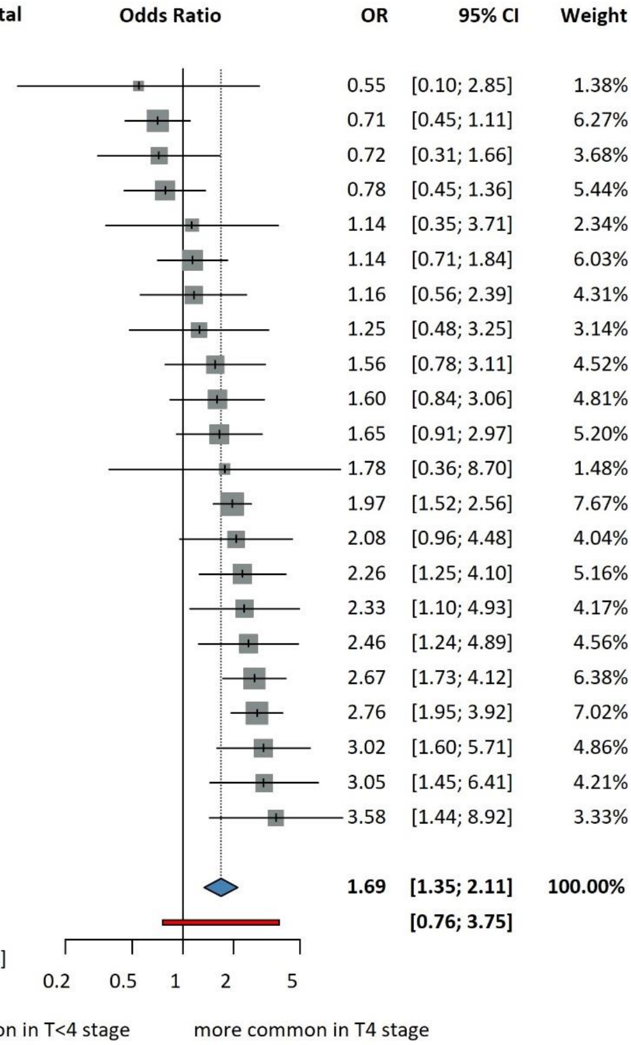

Figure S109.: Association between malnutrition-related complication risk and gastric tumor location (Biological composite scores)

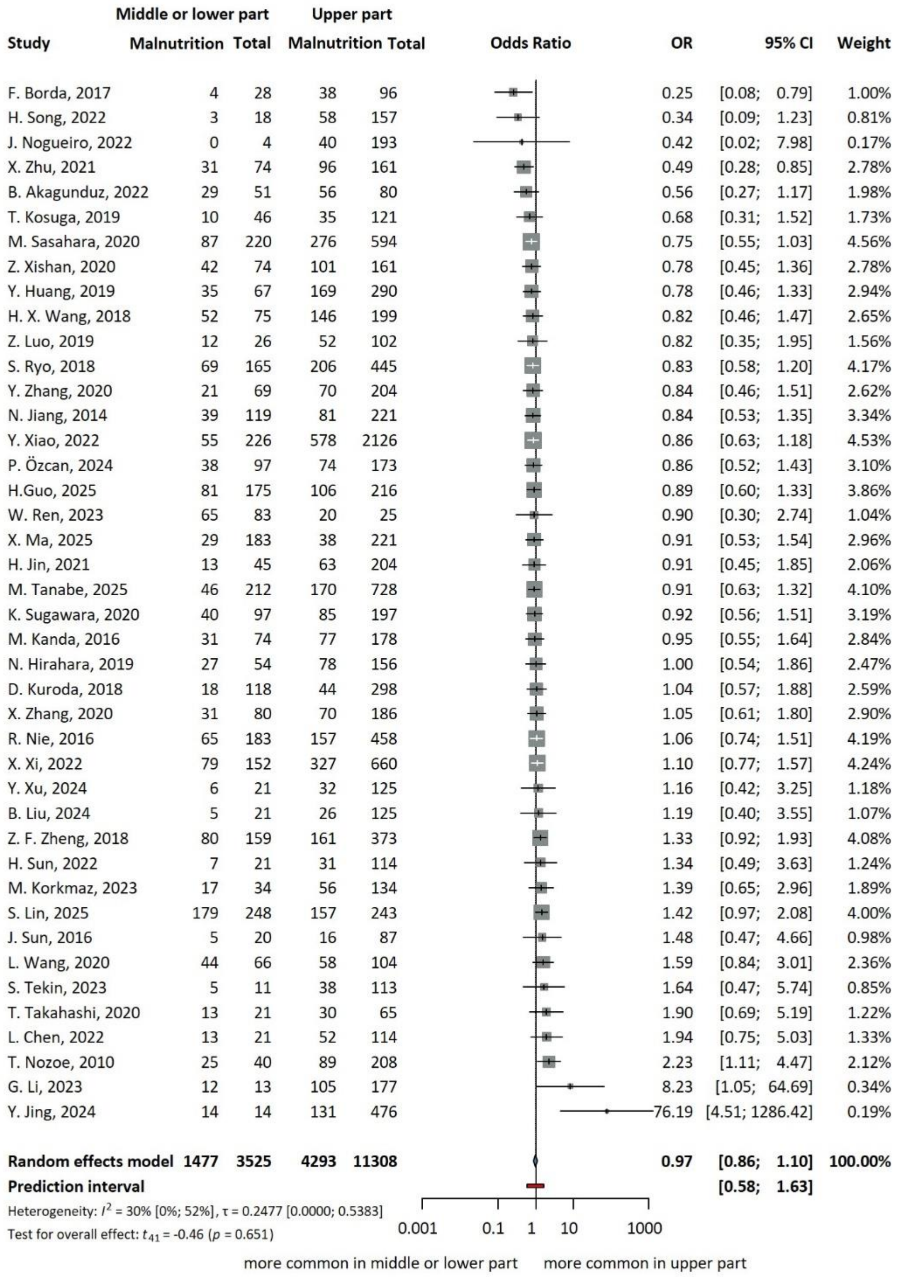

Figure S110.: Association between malnutrition-related complication risk and esophagus tumour location (Biological composite scores)

Study	Upper part Malnutrition	Upper part Total	Middle or lower part Malnutritions	Middle or lower part Total	OR	95% CI	Weight
W. Zuo, 2024	1	6	29	71	0.29	[0.03; 2.61]	0.21%
L. Han, 2016	1	17	33	189	0.30	[0.04; 2.31]	0.24%
N. Kubo, 2017	2	27	27	175	0.44	[0.10; 1.96]	0.45%
I. Hashimoto, 2024	3	27	36	176	0.49	[0.14; 1.70]	0.63%
K. Yan, 2022	28	64	75	128	0.55	[0.30; 1.01]	2.58%
T. Miyazaki, 2016	4	17	61	175	0.58	[0.18; 1.84]	0.73%
Y. Bo, 2016	12	68	43	171	0.64	[0.31; 1.30]	1.90%
Y. Zhao, 2022	66	117	158	237	0.65	[0.41; 1.02]	4.38%
N. Chen, 2024	14	34	161	320	0.69	[0.34; 1.42]	1.88%
J. F. Feng, 2013	13	20	257	355	0.71	[0.27; 1.83]	1.09%
H. Zhang, 2018	12	37	242	618	0.75	[0.37; 1.51]	1.93%
J. Feng, 2022	13	20	137	196	0.80	[0.30; 2.11]	1.05%
G. H. A-Lai, 2019	6	13	39	77	0.84	[0.26; 2.71]	0.71%
S. Xu, 2018	11	31	90	227	0.84	[0.38; 1.83]	1.59%
I. Nonogaki, 2023	25	66	161	398	0.90	[0.53; 1.53]	3.24%
Y. Yu, 2022	54	404	96	664	0.91	[0.64; 1.31]	6.60%
X. Nie, 2025	12	26	118	248	0.94	[0.42; 2.12]	1.48%
Y. Sun, 2025	41	251	273	1643	0.98	[0.68; 1.40]	6.61%
J. Kang, 2022	248	670	744	1991	0.98	[0.82; 1.18]	17.77%
R. Haneda, 2022	7	16	62	142	1.00	[0.35; 2.84]	0.91%
S. B. Chen, 2021	51	110	236	510	1.00	[0.66; 1.52]	5.19%
F. K. Xiao, 2021	296	895	1064	3251	1.02	[0.87; 1.19]	20.71%
K. Okadome, 2020	18	51	99	286	1.03	[0.55; 1.92]	2.44%
I. Yamana, 2015	3	12	25	110	1.13	[0.28; 4.51]	0.52%
L. Xiao, 2021	40	96	37	97	1.16	[0.65; 2.06]	2.83%
P. Fang, 2022	11	45	69	328	1.21	[0.59; 2.52]	1.81%
T. Toyokawa, 2016	3	27	14	158	1.29	[0.34; 4.81]	0.57%
Q. Qi, 2021	10	27	118	380	1.31	[0.58; 2.94]	1.48%
J. Liu, 2022	13	29	48	129	1.37	[0.61; 3.10]	1.47%
X. Xu, 2022	5	15	90	355	1.47	[0.49; 4.42]	0.82%
Y. Fujiwara, 2021	6	23	15	88	1.72	[0.58; 5.08]	0.84%
N. Hirahara, 2018	13	20	65	128	1.80	[0.67; 4.81]	1.02%
Z. Zheng, 2022	13	23	59	142	1.83	[0.75; 4.45]	1.24%
M. Urabe, 2021	27	43	76	181	2.33	[1.18; 4.63]	2.05%
M. Demir, 2022	9	12	42	88	3.29	[0.83; 12.96]	0.53%
P. Y. Wang, 2021	6	9	60	183	4.10	[0.99; 16.96]	0.49%
**Random effects model**	**1097**	**3368**	**4959**	**14615**	**0.98**	**[0.88; 1.08]**	**100.00%**
**Prediction interval**						**[0.81; 1.18]**	

Heterogeneity: I^2 = 4% [0%; 32%] , τ = 0.0790 [0.0000; 0.3948]

Test for overall effect: t_{35} = -0.46 (p = 0.647)

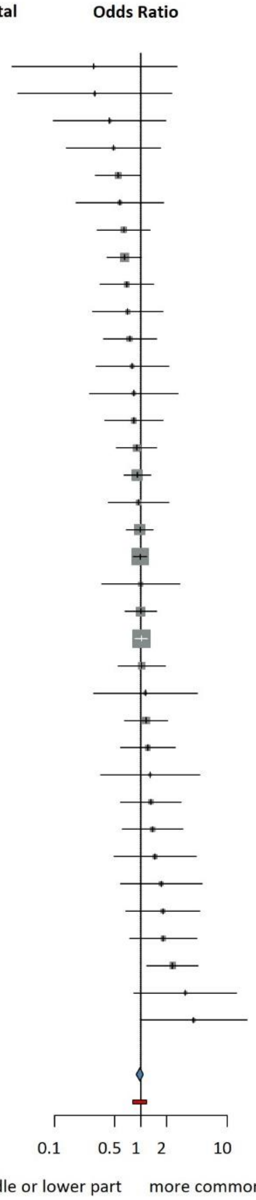

Figure S111.: Association between malnutrition-related complication risk and colorectal tumor location (Biological composite scores)

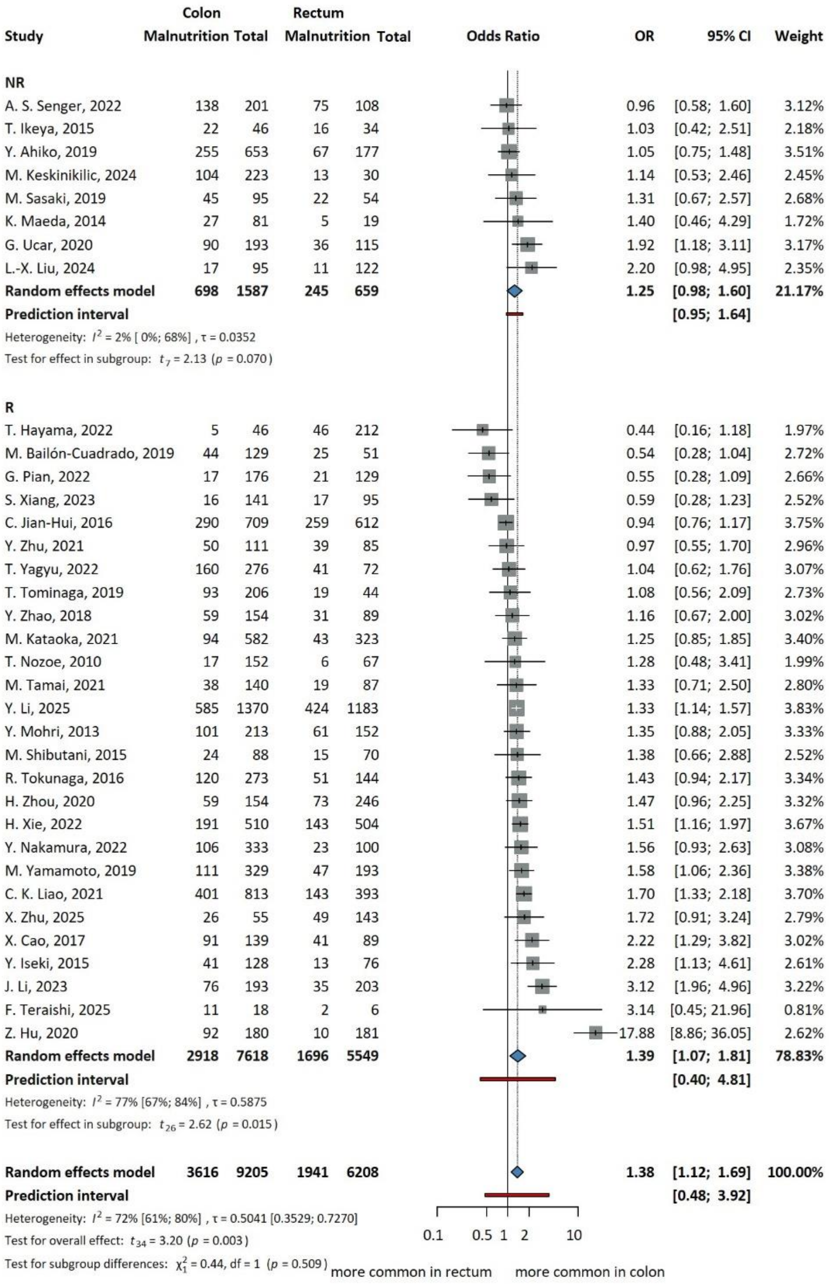

Abbreviations: R= resectable; NR= non-resectable

Figure S112.: Association between malnutrition-related complication risk and colon tumor location (Biological composite scores)

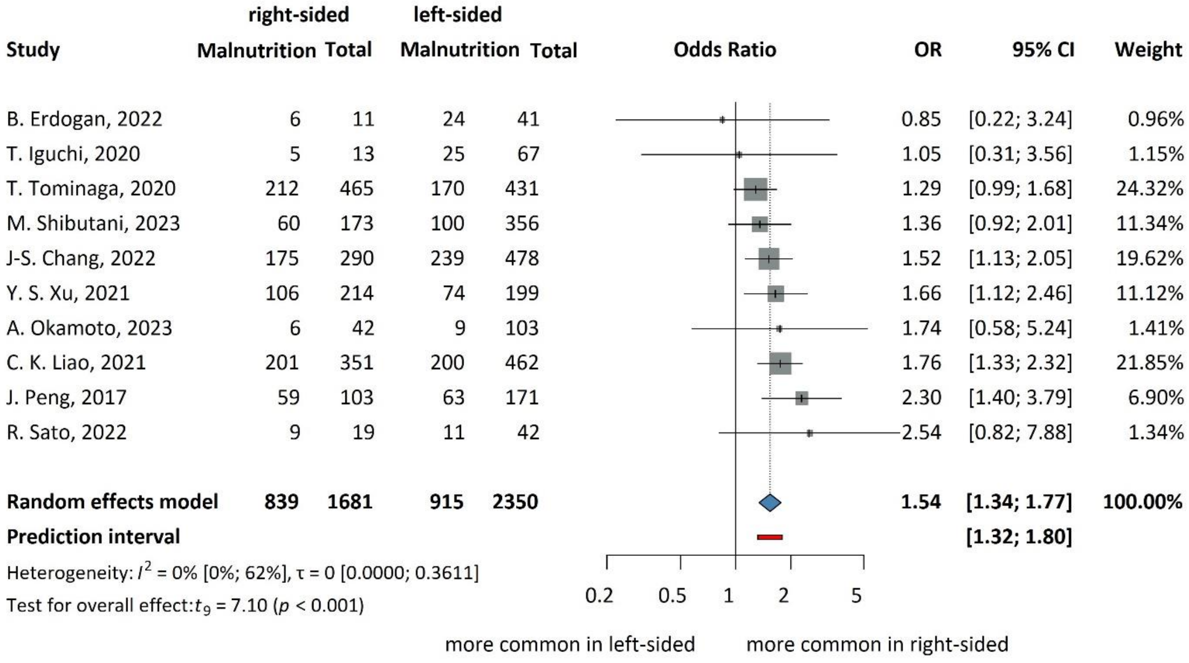

Figure S113.: Association between malnutrition-related complication risk and pancreatic tumour location (Biological composite scores)

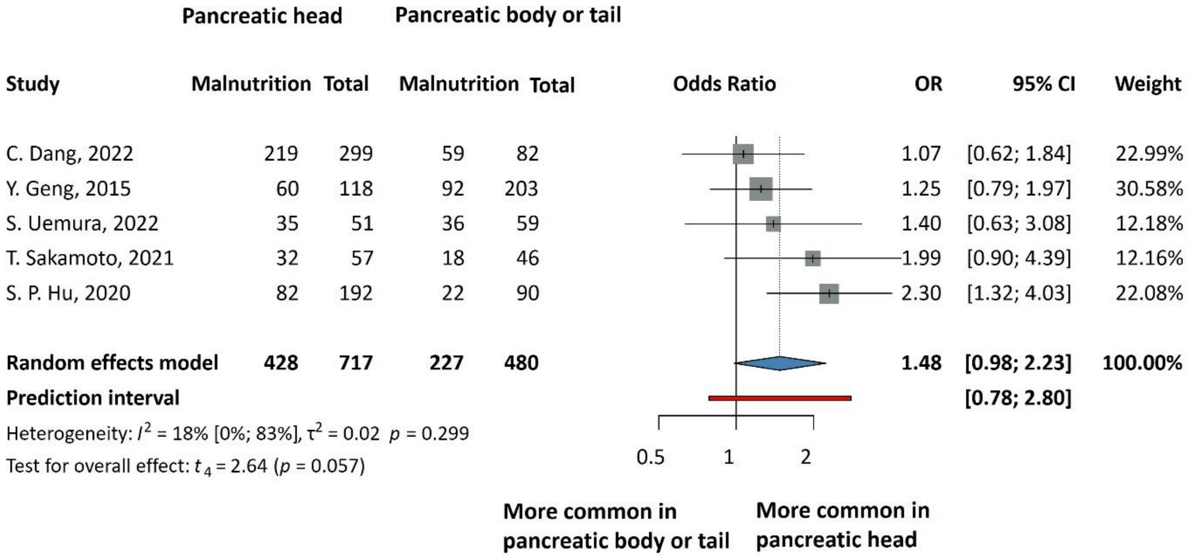

Figure S114.: Association between cachexia and gastric tumor location

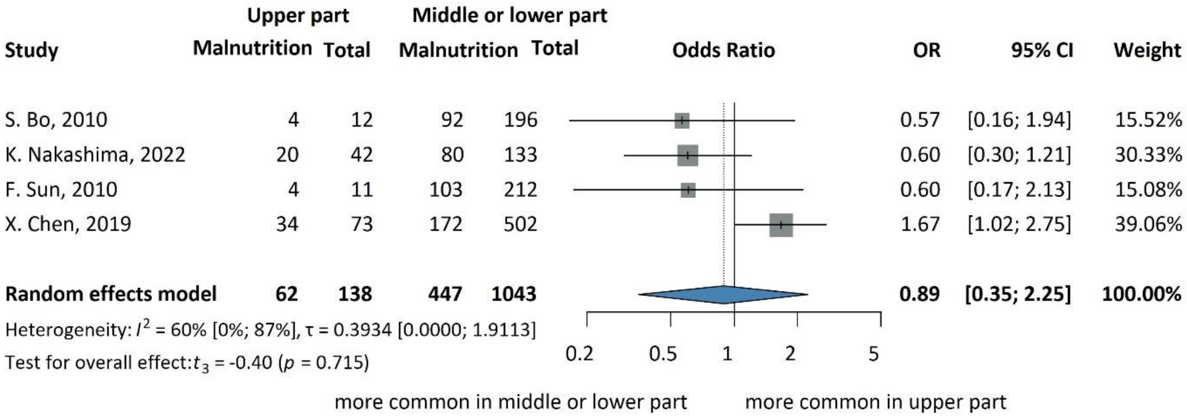

Figure S115.: Association between cachexia and pancreatic tumor location

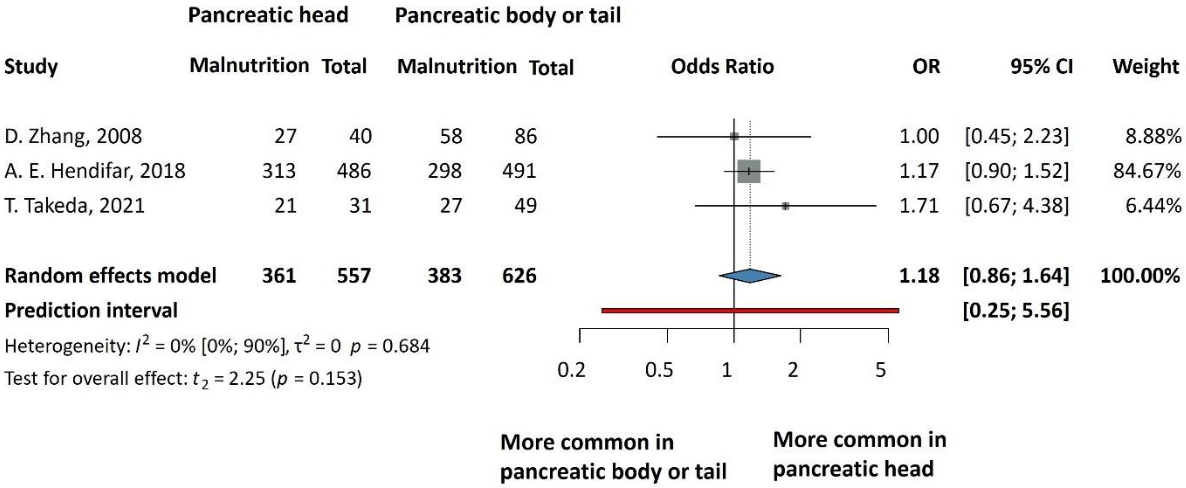

Figure S116.: Association between malnutrition-related complication risk and etiology in hepatocellular carcinoma (Biological composite scores)

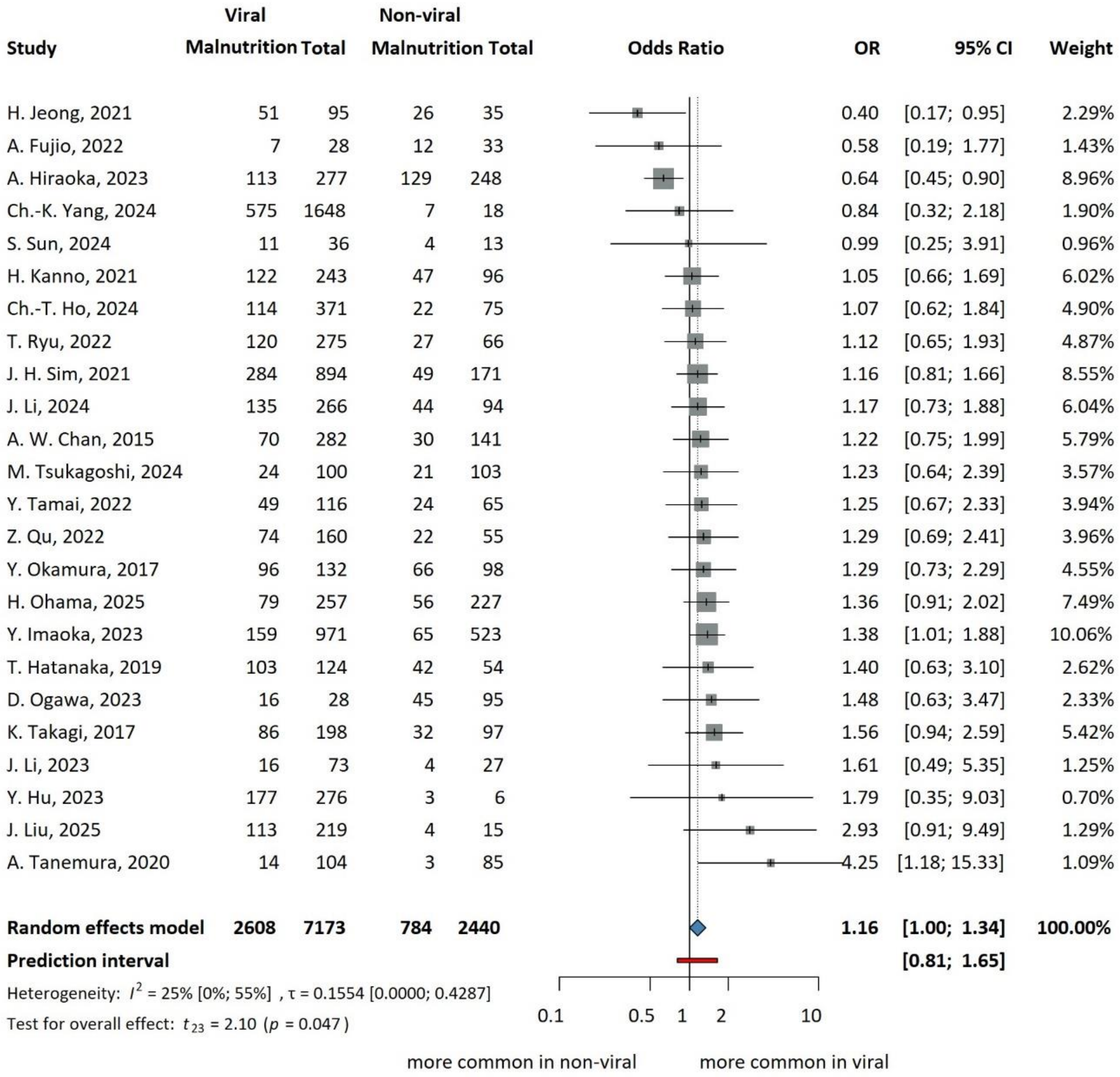

Figure S117.: Association between malnutrition-related complication risk and macrovascular invasion in esophageal cancer (Biological composite scores)

	Macrovascular invasion		No macrovascular invasion				
**Study**	**Malnutrition**	**Total**	**Malnutrition**	**Total**	**OR**	**95% CI**	**Weight**
J. Feng, 2022	11	22	139	194	0.40	[0.16; 0.97]	16.12%
M. Urabe, 2021	59	122	44	102	1.23	[0.73; 2.09]	22.46%
K. Ge, 2021	44	92	126	328	1.47	[0.92; 2.34]	23.59%
H. Ma, 2024	13	34	52	212	1.90	[0.89; 4.07]	18.32%
J. F. Feng, 2013	54	65	216	310	2.14	[1.07; 4.27]	19.50%
**Random effects model**	**181**	**335**	**577**	**1146**	**1.29**	**[0.60; 2.78]**	**100.00%**

Heterogeneity: I^2 = 60% [0%; 85%], τ = 0.5186 [0.0000; 1.8865]

Test for overall effect: t_4 = 0.92 (p = 0.410)

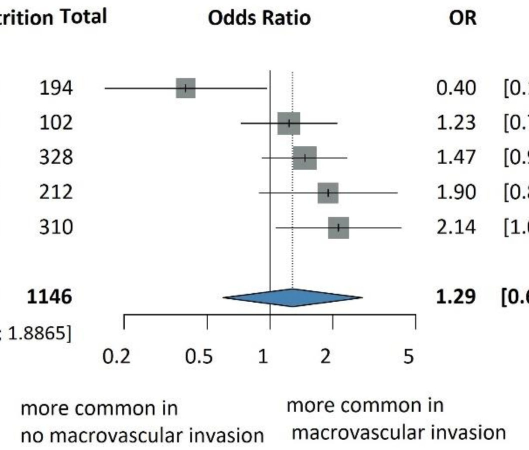

Figure S118.: Association between malnutrition-related complication risk and macrovascular invasion in gastric cancer (Biological composite scores)

	Macrovascular invasion		No macrovascular invasion				
**Study**	**Malnutrition**	**Total**	**Malnutrition**	**Total**	**OR**	**95% CI**	**Weight**
W. Ren, 2023	50	68	35	40	0.40	[0.13; 1.17]	1.67%
T. Matsunaga, 2022	109	236	99	261	1.40	[0.98; 2.01]	9.86%
X. Zhang, 2020	40	89	65	185	1.51	[0.90; 2.52]	5.98%
X. Zhu, 2021	75	119	66	126	1.55	[0.93; 2.58]	6.06%
H. Guo, 2025	100	187	87	208	1.60	[1.07; 2.38]	8.62%
X. Ma, 2025	38	184	29	220	1.71	[1.01; 2.91]	5.74%
S. Onuma, 2023	96	203	95	280	1.75	[1.21; 2.53]	9.46%
H. Saito, 2017	116	221	72	186	1.75	[1.18; 2.60]	8.68%
Z. Xishan, 2020	80	119	68	126	1.75	[1.04; 2.94]	5.92%
I. Tirnova, 2025	8	13	26	55	1.78	[0.52; 6.14]	1.30%
H. Furuke, 2021	32	284	32	511	1.90	[1.14; 3.18]	6.02%
S. Lin, 2025	73	91	263	400	2.11	[1.21; 3.68]	5.32%
F. Tustumi, 2024	126	177	189	352	2.13	[1.45; 3.14]	8.95%
Y. Choi, 2017	19	32	98	256	2.36	[1.11; 4.98]	3.24%
J. Nogueiro, 2022	20	67	20	132	2.38	[1.17; 4.83]	3.58%
M. Ishizuka, 2014	59	106	15	47	2.68	[1.30; 5.52]	3.44%
W. K. Eo, 2015	7	14	70	300	3.29	[1.11; 9.69]	1.67%
T. Nozoe, 2010	43	62	71	186	3.67	[1.98; 6.78]	4.51%
**Random effects model**	**1091**	**2272**	**1400**	**3871**	**1.82**	**[1.56; 2.13]**	**100.00%**
**Prediction interval**						**[1.28; 2.58]**	

Heterogeneity: I^2 = 16% [0%; 52%], τ = 0.1476 [0.0000; 0.5040]

Test for overall effect: t_{17} = 8.13 (p < 0.001)

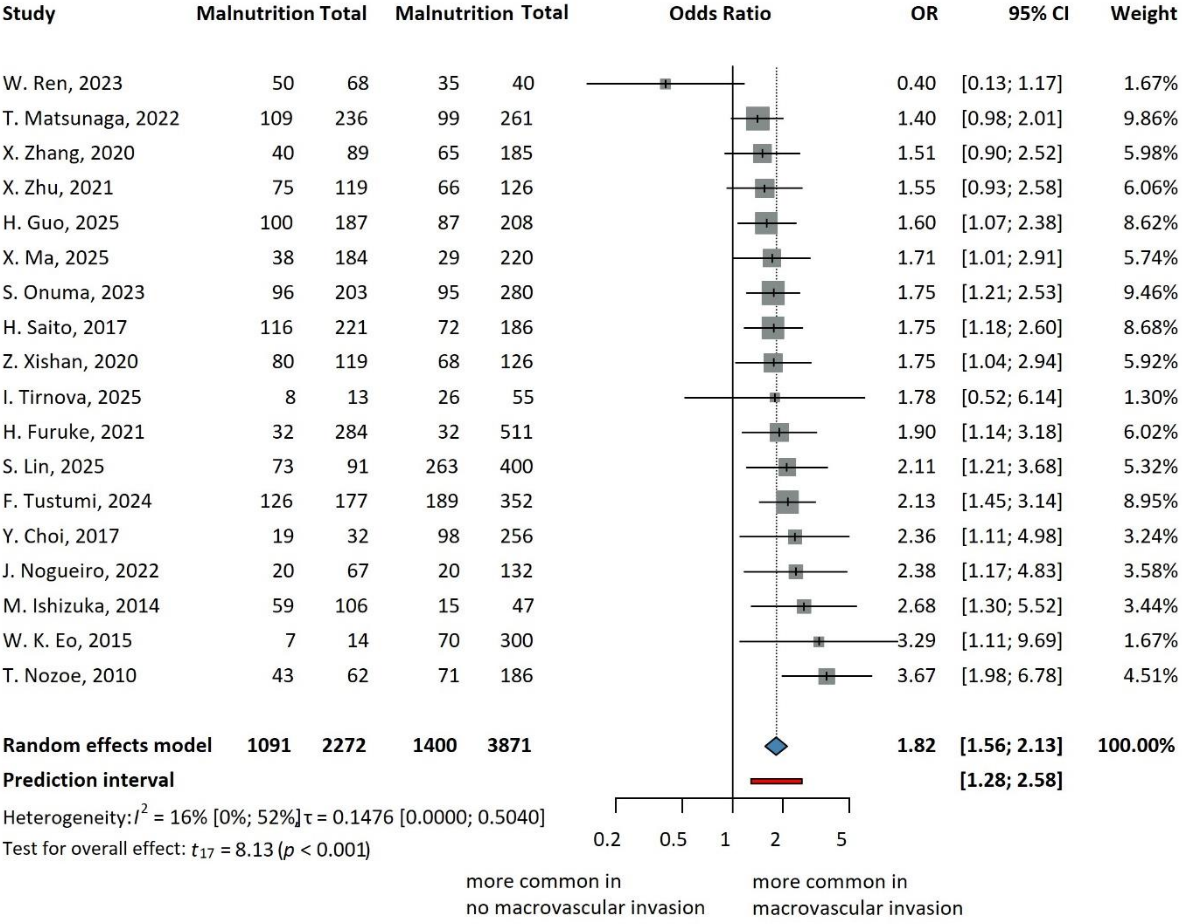

Figure S119.: Association between malnutrition-related complication risk and macrovascular invasion in colorectal carcinoma (Biological composite scores)

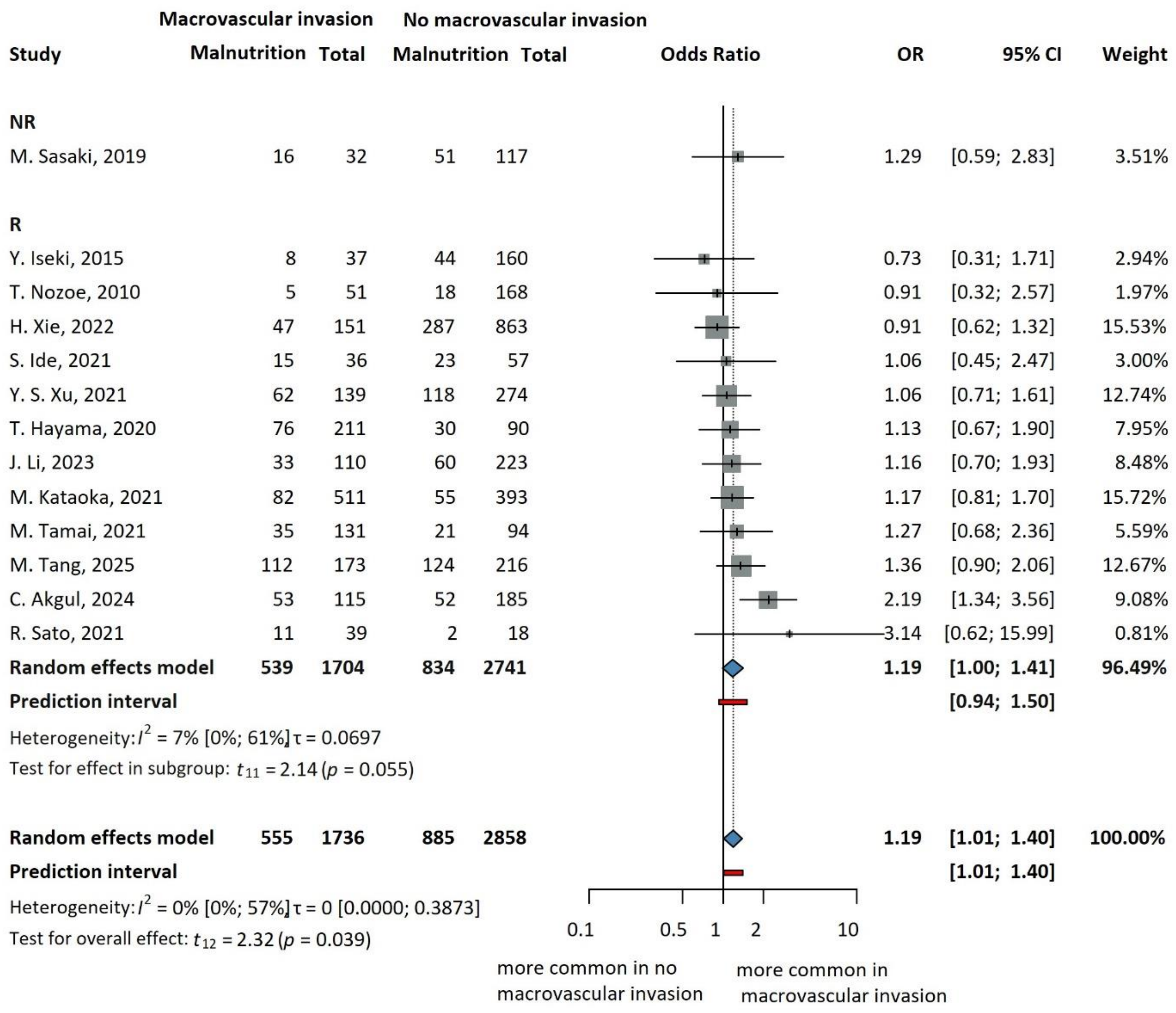

Abbreviations: R= resectable; NR= non-resectable

Figure S120.: Association between malnutrition-related complication risk and macrovascular invasion in hepatocellular carcinoma (Biological composite scores)

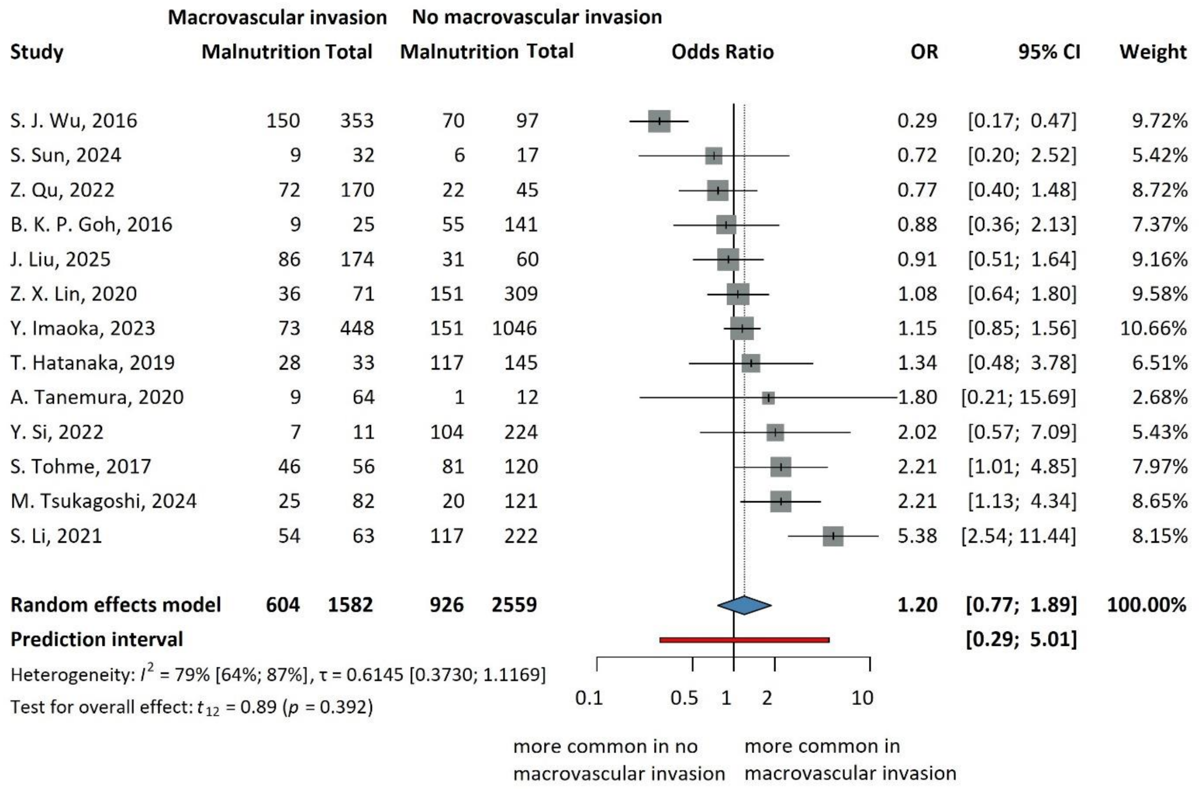

Figure S121.: Association between malnutrition-related complication risk and macrovascular invasion in pancreatic ductal adenocarcinoma (Biological composite scores)

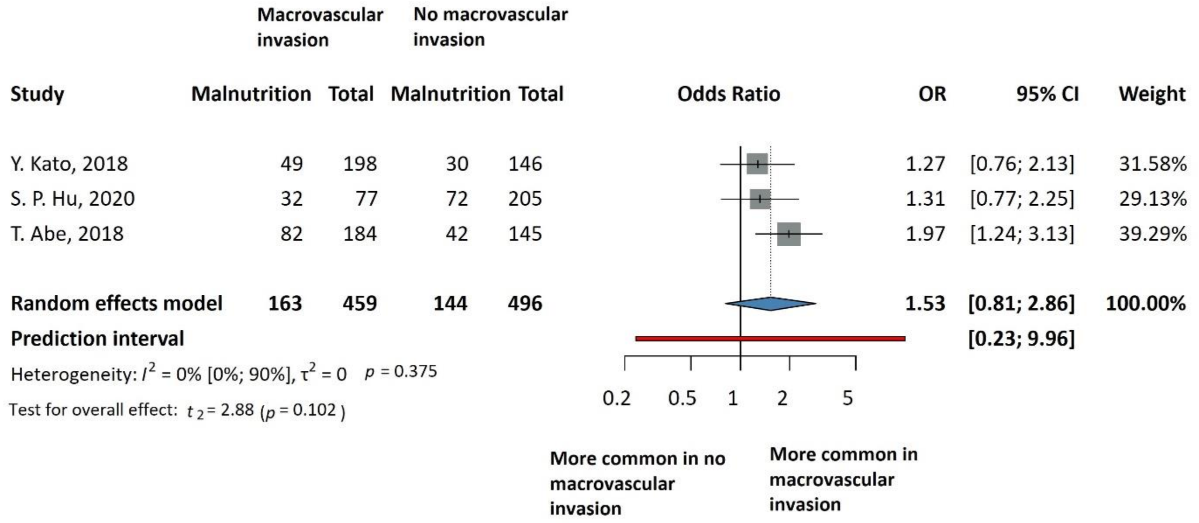

Supplementary Document S13: Individual forest plots for neoadjuvant therapy: Figures S122-S125.

Figure S122.: Association between malnutrition-related complication risk and neoadjuvant chemotherapy in upper gastrointestinal cancer (Biological composite scores)

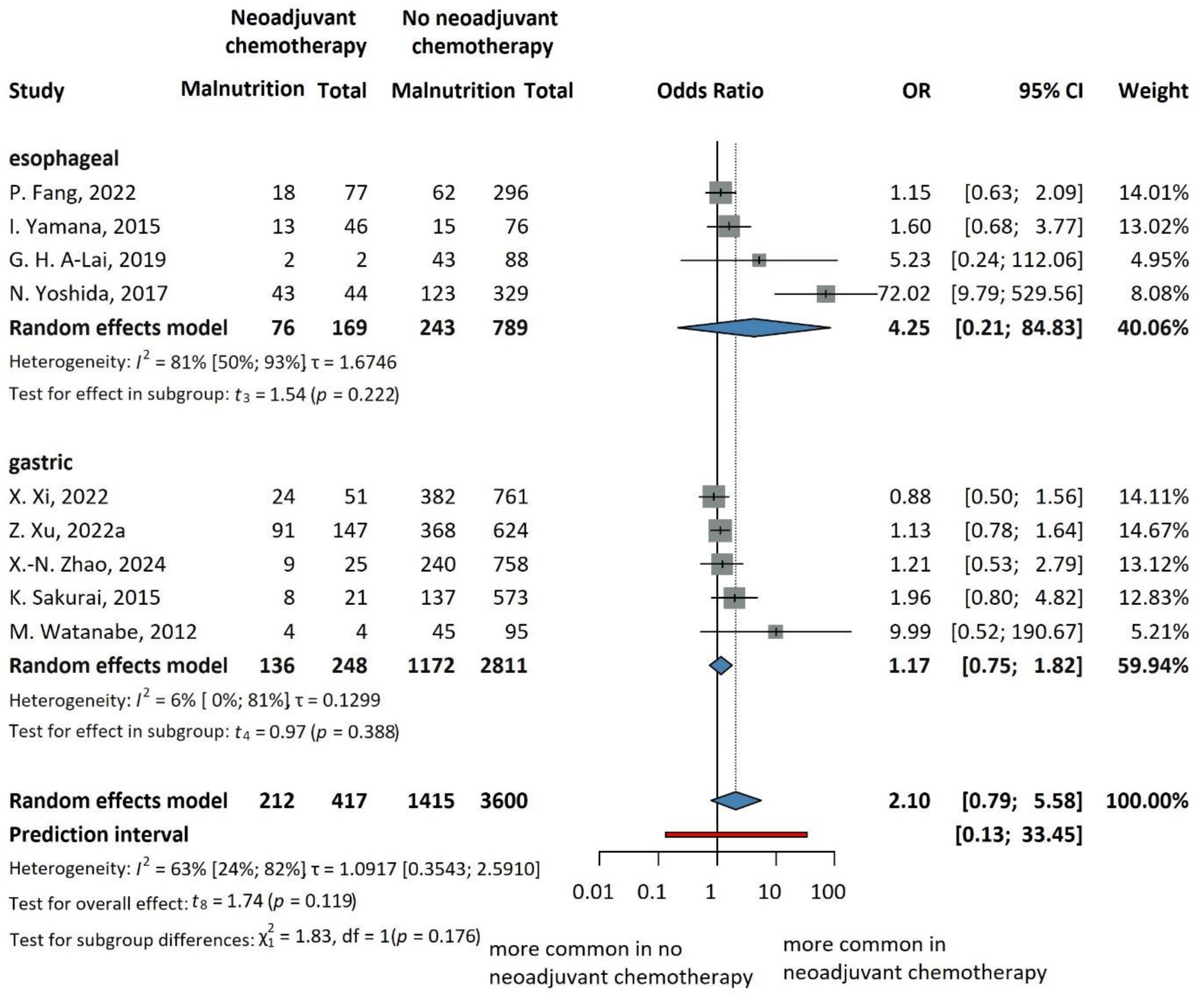

Figure S123.: Association between malnutrition risk and neoadjuvant chemo or radiotherapy in esophageal and in colorectal carcinoma (Symptom-based risk assessment tool)

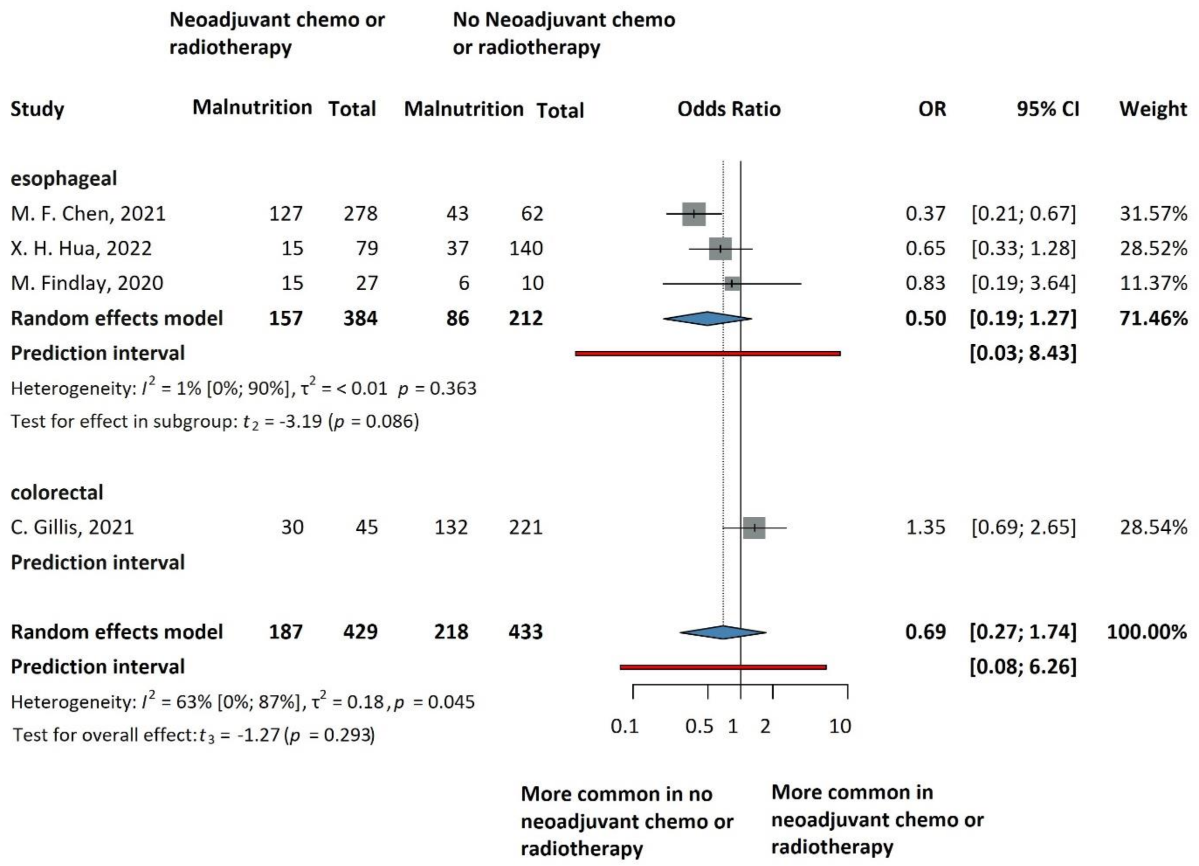

Figure S124.: Association between malnutrition-related complication risk and neoadjuvant chemo or radiotherapy in upper gastrointestinal cancer (Biological composite scores)

Study	Neoadjuvant chemo or radiotherapy: Malnutrition	Neoadjuvant chemo or radiotherapy: Total	No neoadjuvant chemo or radiotherapy: Malnutrition	No neoadjuvant chemo or radiotherapy: Total	OR	95% CI	Weight
**esophageal**							
X. Nie, 2025	57	171	73	103	0.21	[0.12; 0.35]	6.70%
R. Haneda, 2022	25	80	44	78	0.35	[0.18; 0.67]	6.27%
P. Fang, 2022	80	373	80	373	1.00	[0.70; 1.42]	7.25%
N. Kubo, 2018	12	40	19	65	1.04	[0.44; 2.46]	5.49%
R. Yamanaka-Kohno, 2021	10	35	9	38	1.29	[0.45; 3.67]	4.82%
P. Y. Wang, 2021	31	77	35	115	1.54	[0.84; 2.82]	6.44%
I. Nonogaki, 2023	125	276	61	188	1.72	[1.17; 2.54]	7.15%
I. Yamana, 2015	23	75	33	169	1.82	[0.98; 3.39]	6.38%
K. Kouzu, 2020	27	55	21	69	2.20	[1.06; 4.60]	5.96%
Y. Sun, 2025	125	327	189	1567	4.51	[3.44; 5.91]	7.44%
G. H. A-Lai, 2019	2	2	43	88	5.23	[0.24; 112.06]	1.25%
**Random effects model**	**517**	**1511**	**607**	**2853**	**1.22**	**[0.65; 2.28]**	**65.17%**
**Prediction interval**						**[0.17; 8.99]**	
Heterogeneity: $I^2 = 93\%$ [90%; 95%], $\tau = 0.8368$							
Test for effect in subgroup: $t_{10} = 0.71$ ($p = 0.492$)							
**gastric**							
J. Nogueiro, 2022	4	31	37	172	0.54	[0.18; 1.64]	4.60%
X. Xi, 2022	24	51	382	761	0.88	[0.50; 1.56]	6.57%
F. Borda, 2017	5	11	48	113	1.13	[0.33; 3.92]	4.17%
Z. Xu, 2022a	91	147	368	624	1.13	[0.78; 1.64]	7.20%
X.-N. Zhao, 2024	9	25	240	758	1.21	[0.53; 2.79]	5.60%
K. Sakurai, 2015	8	21	137	573	1.96	[0.80; 4.82]	5.34%
M. Watanabe, 2012	4	4	45	95	9.99	[0.52; 190.67]	1.33%
**Random effects model**	**145**	**290**	**1257**	**3096**	**1.10**	**[0.80; 1.53]**	**34.83%**
Heterogeneity: $I^2 = 0\%$ [0%; 71%], $\tau = 0$							
Test for effect in subgroup: $t_6 = 0.75$ ($p = 0.481$)							
**Random effects model**	**662**	**1801**	**1864**	**5949**	**1.20**	**[0.80; 1.80]**	**100.00%**
**Prediction interval**						**[0.27; 5.43]**	
Heterogeneity: $I^2 = 89\%$ [84%; 92%], $\tau = 0.6863$ [0.4633; 1.1516]							
Test for overall effect: $t_{17} = 0.96$ ($p = 0.353$)							
Test for subgroup differences: $\chi^2_1 = 0.10$, df = 1 ($p = 0.746$)							

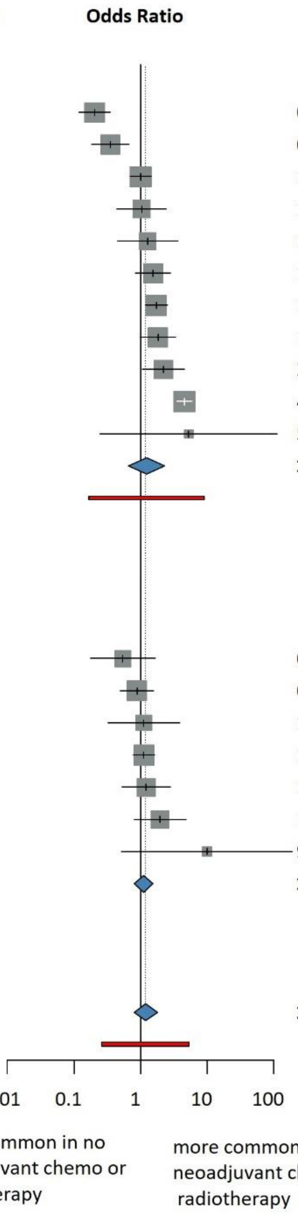

Figure S125.: Association between malnutrition-related complication risk and neoadjuvant chemo or radiotherapy in colorectal cancer (Biological composite scores)

Study	Neoadjuvant chemo or radiotherapy: Malnutrition	Neoadjuvant chemo or radiotherapy: Total	No neoadjuvant chemo or radiotherapy: Malnutrition	No neoadjuvant chemo or radiotherapy: Total	OR	95% CI	Weight
T. Amano, 2023	15	46	79	191	0.69	[0.35; 1.35]	10.17%
L. Zhang, 2023	143	454	64	185	0.87	[0.61; 1.25]	12.12%
A. Okamoto, 2023	4	40	11	105	0.95	[0.28; 3.17]	6.87%
C. K. Liao, 2021	26	71	117	322	1.01	[0.59; 1.73]	11.14%
X. Wang, 2020	20	56	62	225	1.46	[0.79; 2.71]	10.58%
M. Bailón-Cuadrado, 2019	36	67	102	293	2.17	[1.27; 3.72]	11.11%
C. Akgul, 2024	37	74	68	226	2.32	[1.36; 3.98]	11.11%
R. Tokunaga, 2015	34	47	197	509	4.14	[2.13; 8.04]	10.29%
S. Xiang, 2023	16	52	17	184	4.37	[2.02; 9.45]	9.56%
Y. Nakamura, 2023	10	14	170	587	6.13	[1.90; 19.82]	7.05%
**Random effects model**	**341**	**921**	**887**	**2827**	**1.78**	**[1.05; 3.02]**	**100.00%**
**Prediction interval**						**[0.37; 8.68]**	

Heterogeneity: I^2 = 79% [62%; 88%], τ = 0.6457 [0.3529; 1.3344]

Test for overall effect: t_9 = 2.47 (p = 0.036)

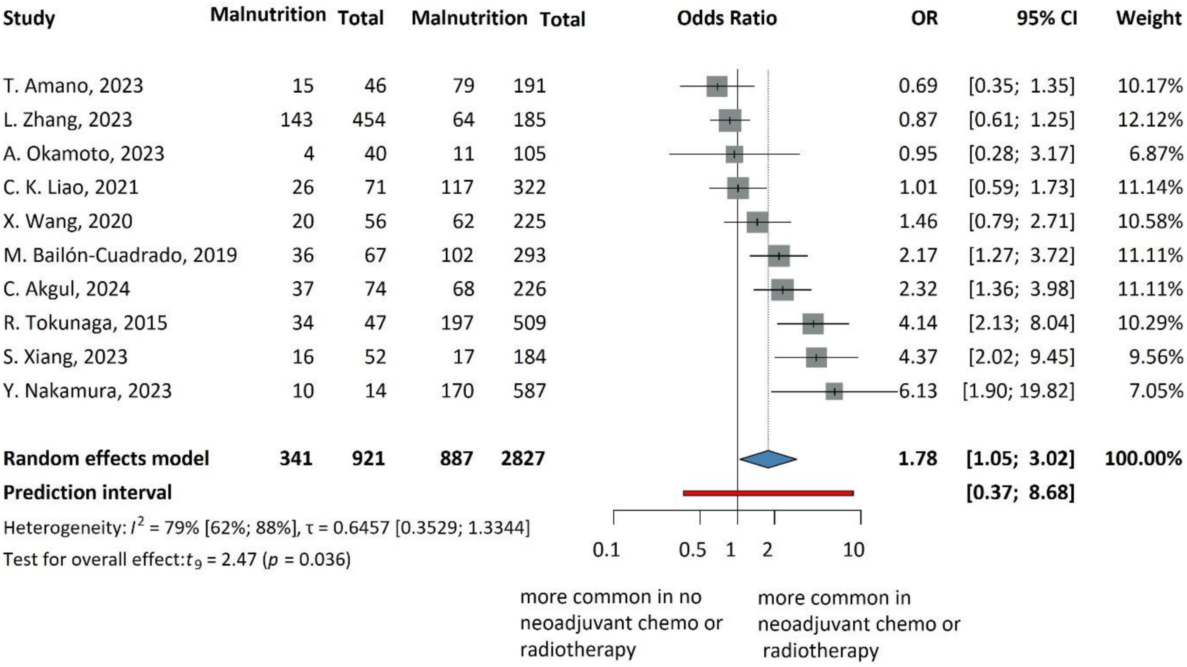

Supplementary Document S14: Moderator analysis: Figures S126-S172

For age categories, we assumed a normal distribution of age, allowing us to treat mean and median values equivalently; therefore, studies were categorized using an age cut off of <70 vs. ≥70, regardless of whether the reported value was a mean or median. However, moderator analysis based on categorized continuous variables may introduce ecological bias.

Figure S126. Cancer type moderator analysis for the association between malnutrition-related complication risk and age >65 vs. <65 in gastrointestinal cancer (Biological composite scores)

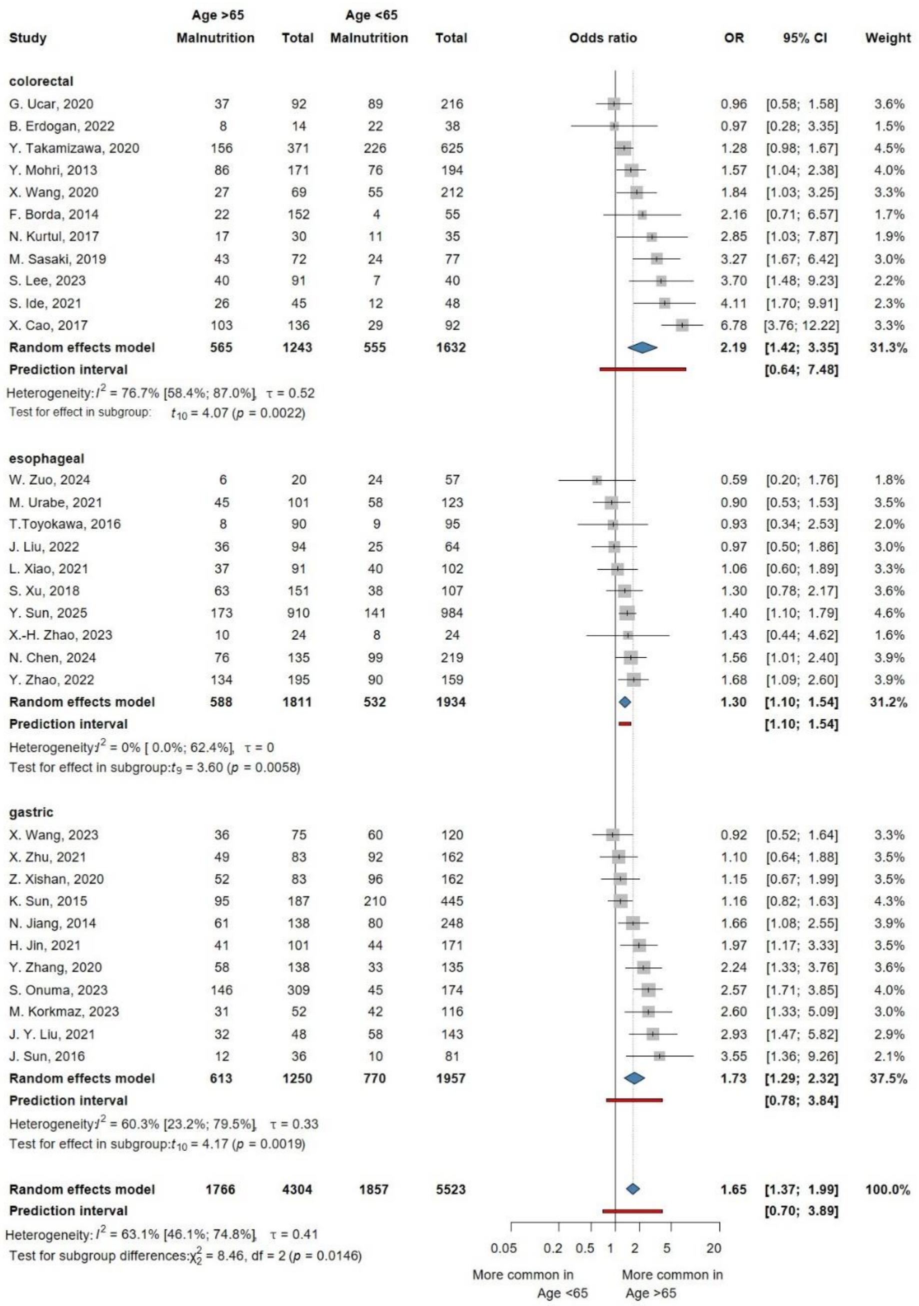

Figure S127. Resectability moderator analysis for the association between malnutrition-related complication risk and age >65 vs. <65 in gastrointestinal cancer (Biological composite scores)

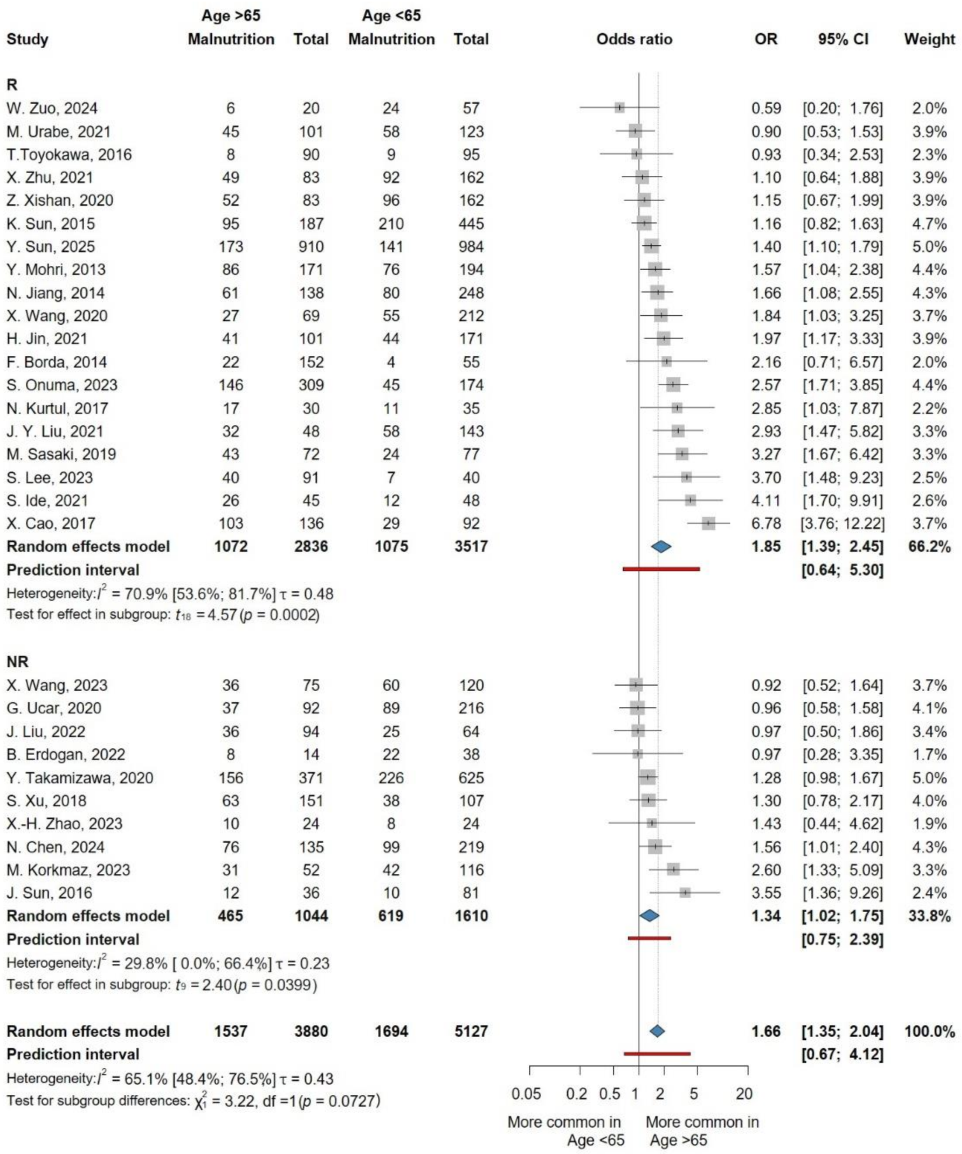

Abbreviations: R= resectable, NR= non-resectable

Figure S128. Assessment tool moderator analysis for the association between malnutrition-related complication risk and age >65 vs. <65 in gastrointestinal cancer (Biological composite scores)

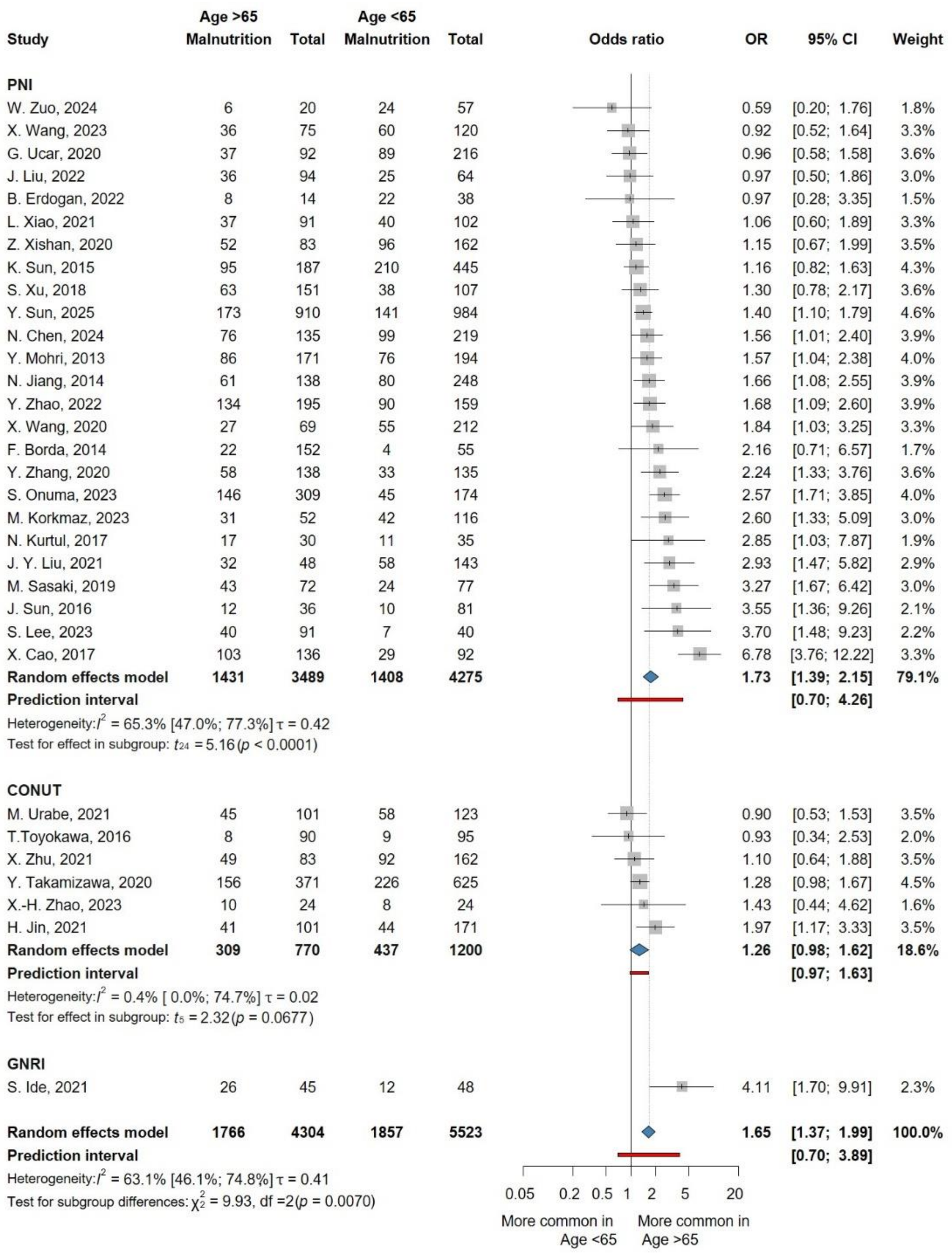

Abbreviations: PNI= Prognostic Nutritional Index, CONUT= Controlling Nutritional Status, GNRI= Geriatric Nutritional Risk Index

Figure S129. Female proportion meta regression analysis for the association between malnutrition-related complication risk and age >65 vs. <65 in gastrointestinal cancer (Biological composite scores)

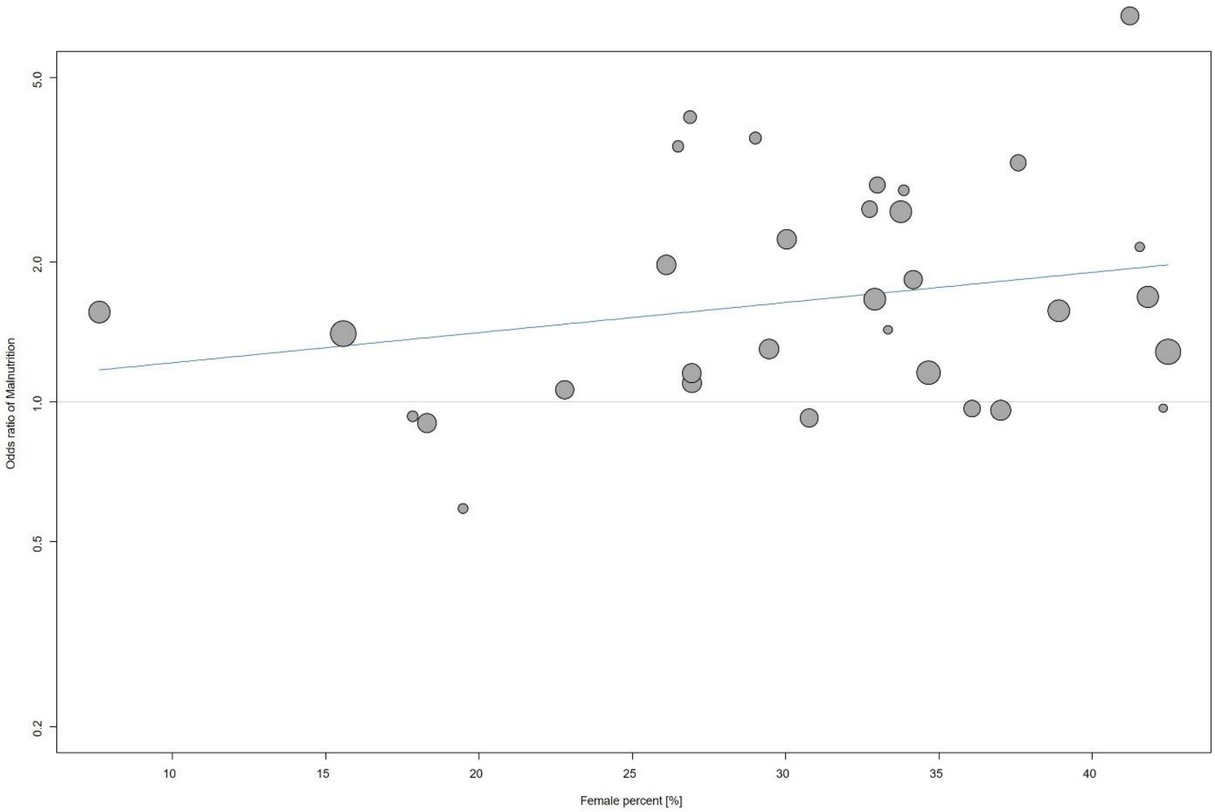

Assuming a linear relation, the estimated slope is 0.015 [95% CI: -0.0064 - 0.0363] [log(OR of Malnutrition) / female %] (p-value: 0.1624). NOTE: it is given in the log scale!

Figure S130. Female proportion meta regression analysis for the association between malnutrition risk and sex in colorectal cancer (symptom-based tools)

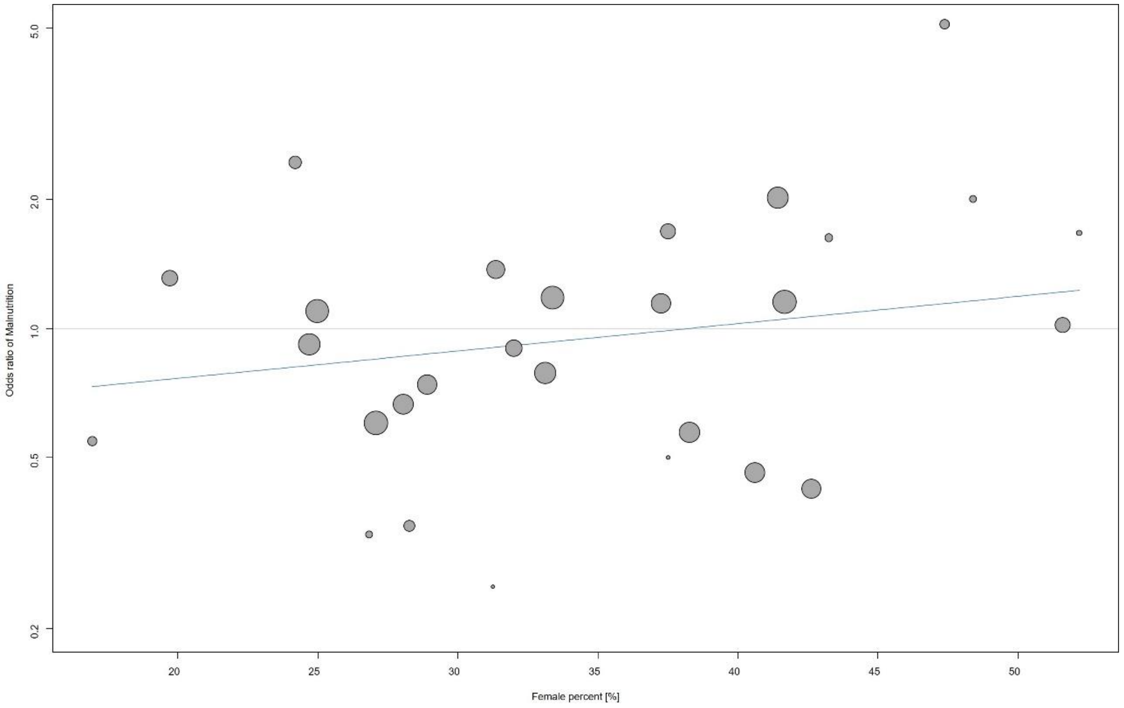

Assuming a linear relation, the estimated slope is -0.0231 [95% CI: -0.0553 - 0.0091] [log(OR of Malnutrition) / female %] (p-value: 0.1502). NOTE: it is given in the log scale!

Figure S131. Female proportion meta regression analysis for the association between malnutrition-related complication risk and sex in esophageal cancer (Biological composite scores)

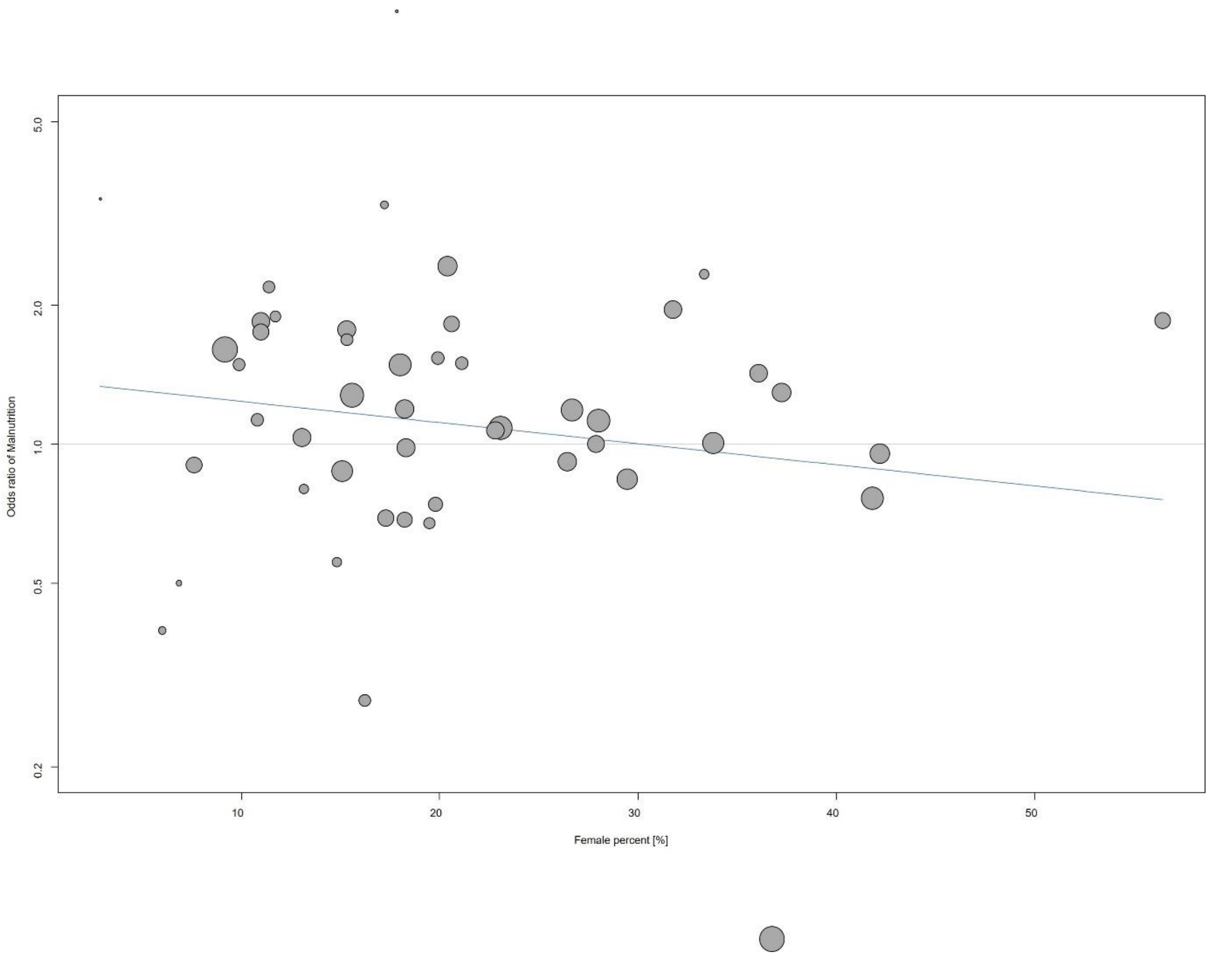

Assuming a linear relation, the estimated slope is -0.0105 [95% CI: -0.028 - 0.0069] [log(OR of Malnutrition) / female %] (p-value: 0.2305). NOTE: it is given in the log scale!

Figure S132. Female proportion meta regression analysis for the association between malnutrition-related complication risk and sex in gastric cancer (Biological composite scores)

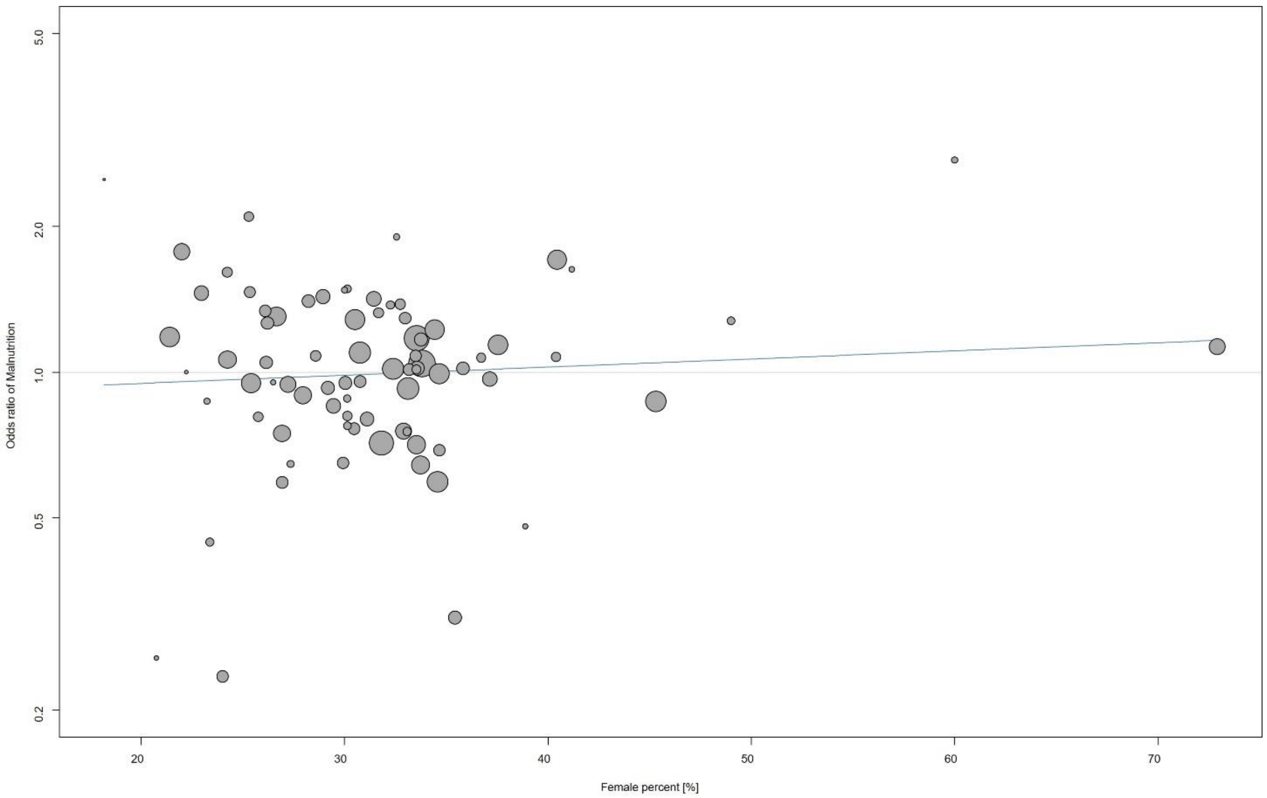

Assuming a linear relation, the estimated slope is 0.0038 [95% CI: -0.0072 - 0.0149] [log(OR of Malnutrition) / female %] (p-value: 0.4914). NOTE: it is given in the log scale!

Figure S133. Female proportion meta regression analysis for the association between malnutrition-related complication risk and sex in resectable hepatocellular carcinoma (Biological composite scores)

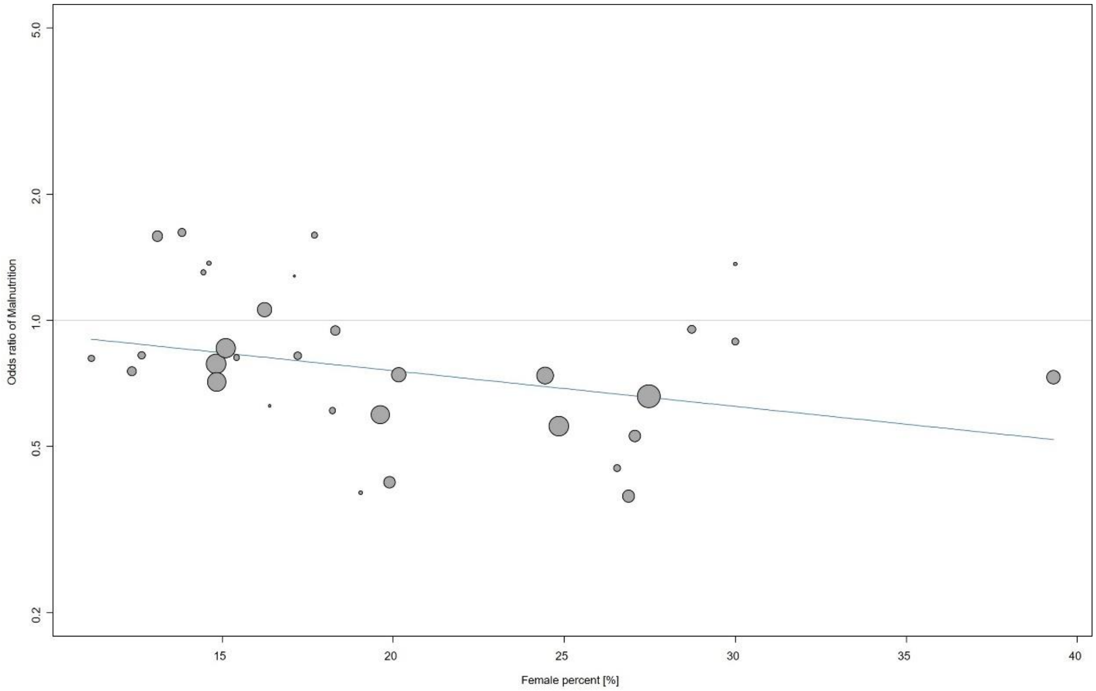

Assuming a linear relation, the estimated slope is -0.0197 [95% CI: -0.0374 - -0.0019] [log(OR of Malnutrition) / female %] (p-value: 0.0308). NOTE: it is given in the log scale!

Figure S134. Female proportion meta regression analysis for the association between cachexia and sex in gastrointestinal cancer

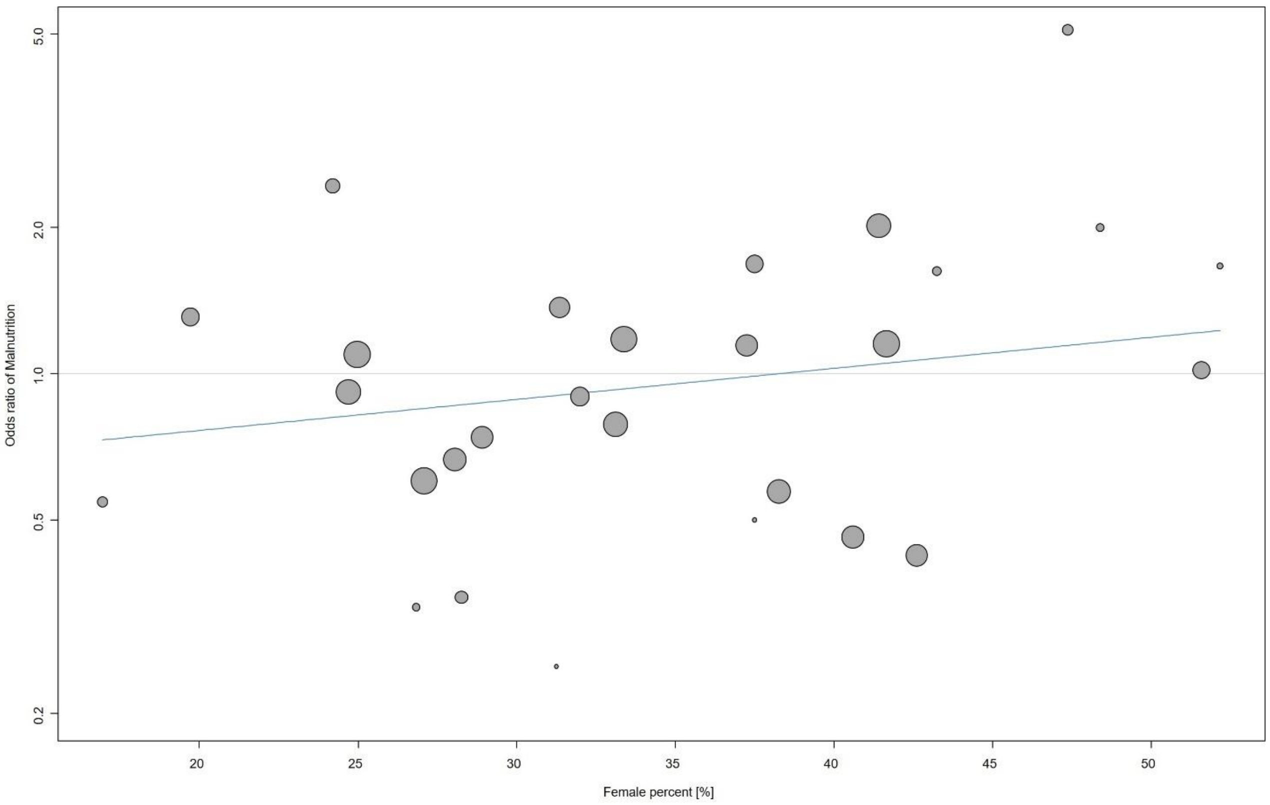

Assuming a linear relation, the estimated slope is 0.0147 [95% CI: -0.0119 - 0.0413] [log(OR of Malnutrition) / female %] (p-value: 0.2671). NOTE: it is given in the log scale!

Figure S135. Cancer type moderator analysis for the association between cachexia and sex in gastrointestinal cancer

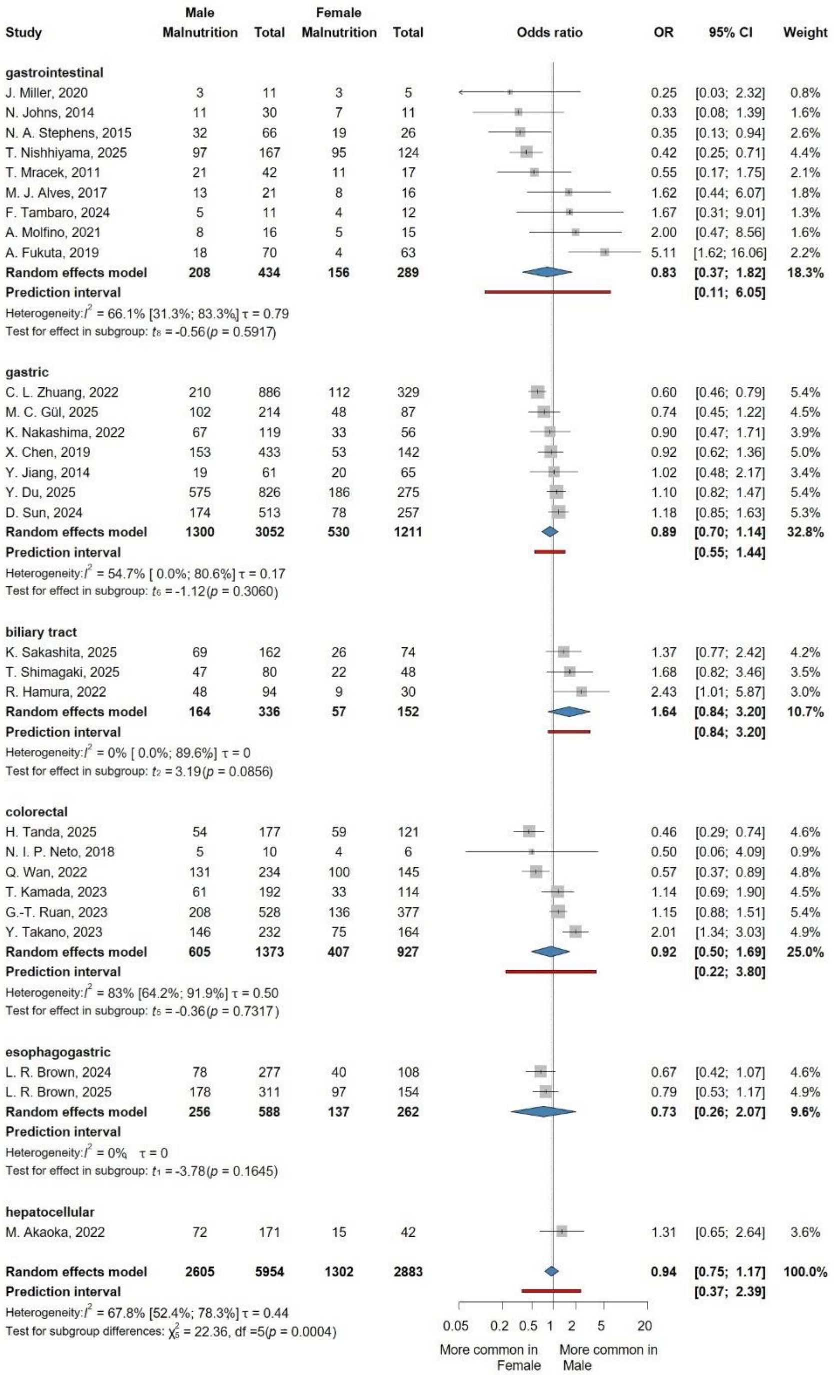

Figure S136. Assessment tool moderator analysis for the association between cachexia and sex in gastrointestinal cancer

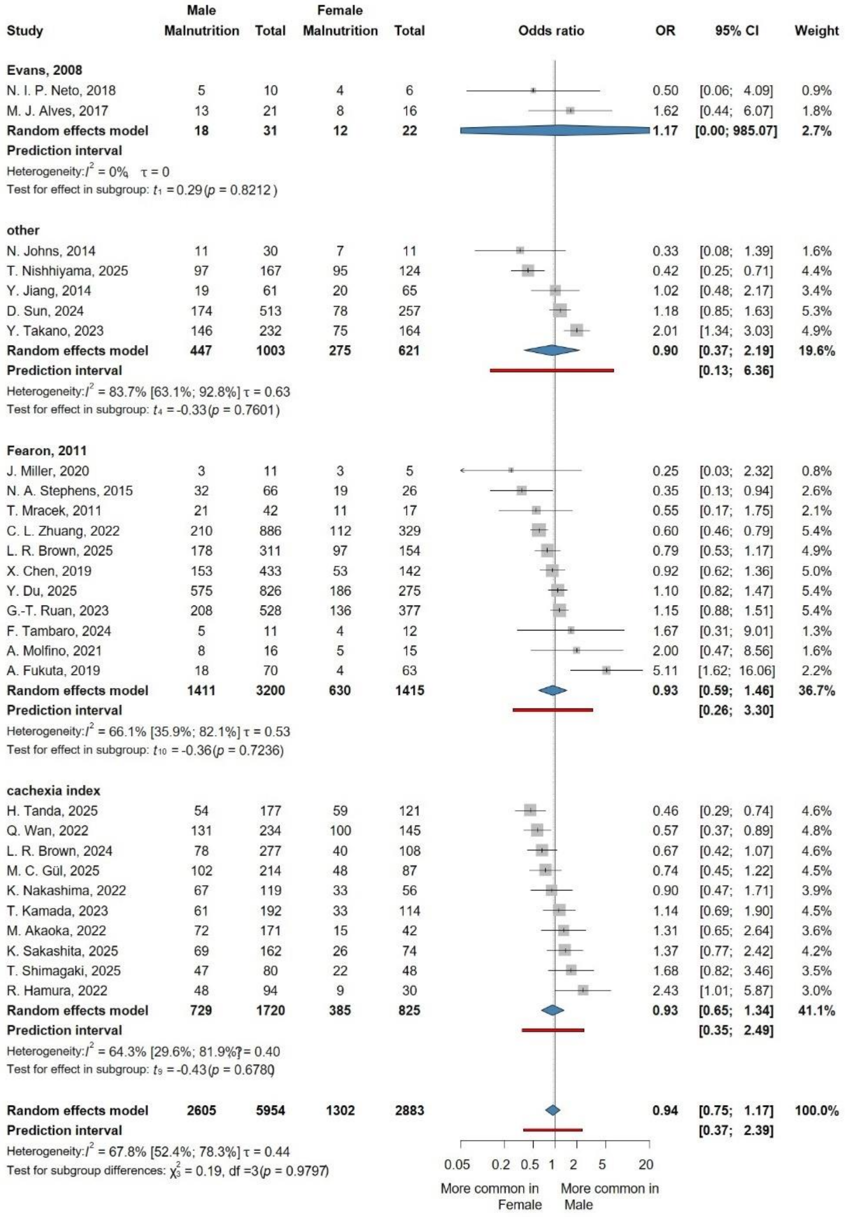

Figure S137. Resectability moderator analysis for the association between malnutrition-related complication risk and sex in esophageal cancer (Biological composite scores)

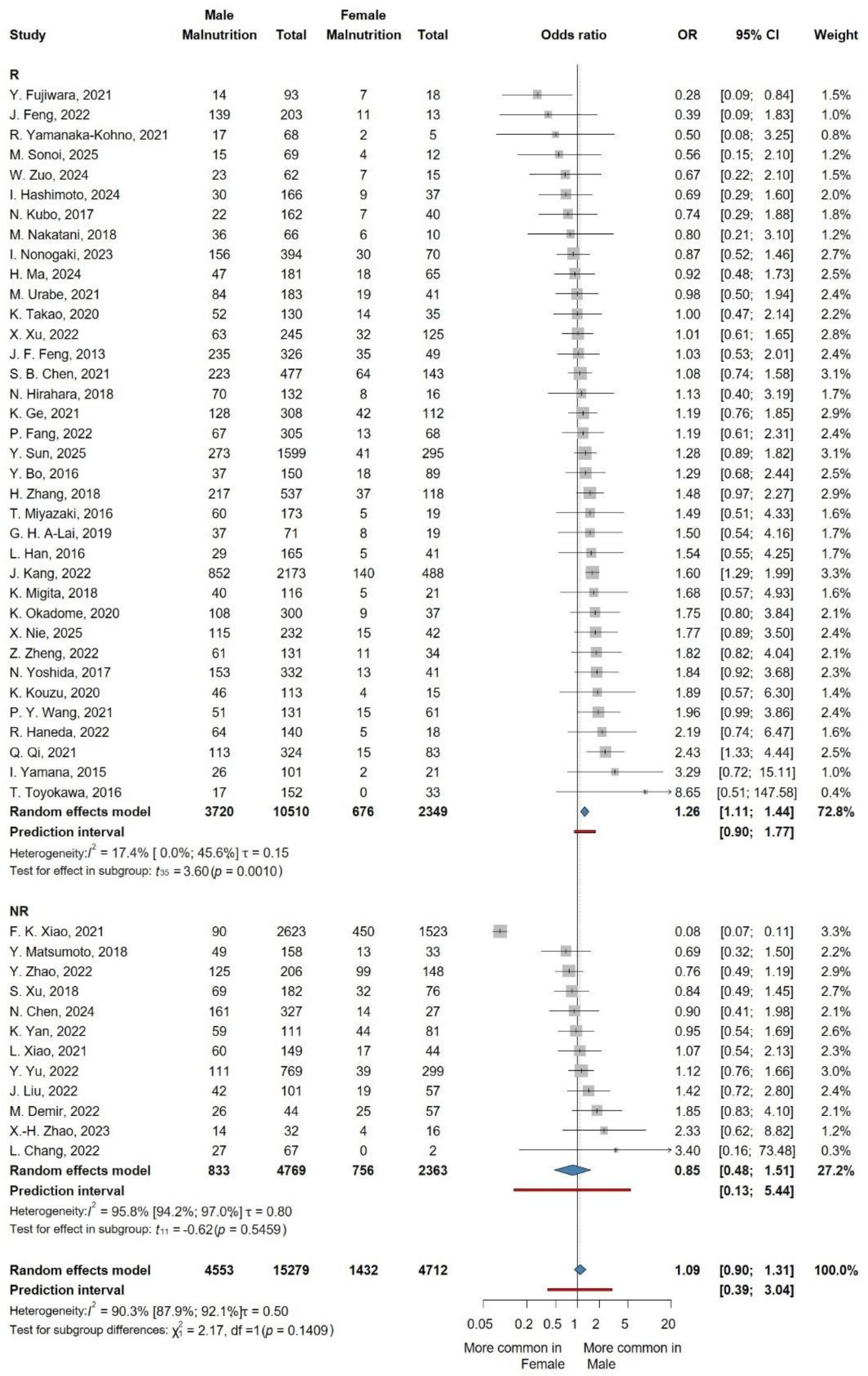

Abbreviations: R= resectable, NR= non-resectable

Figure S138. Cancer type moderator analysis for the association between malnutrition-related complication risk and sex in esophageal cancer (Biological composite scores)

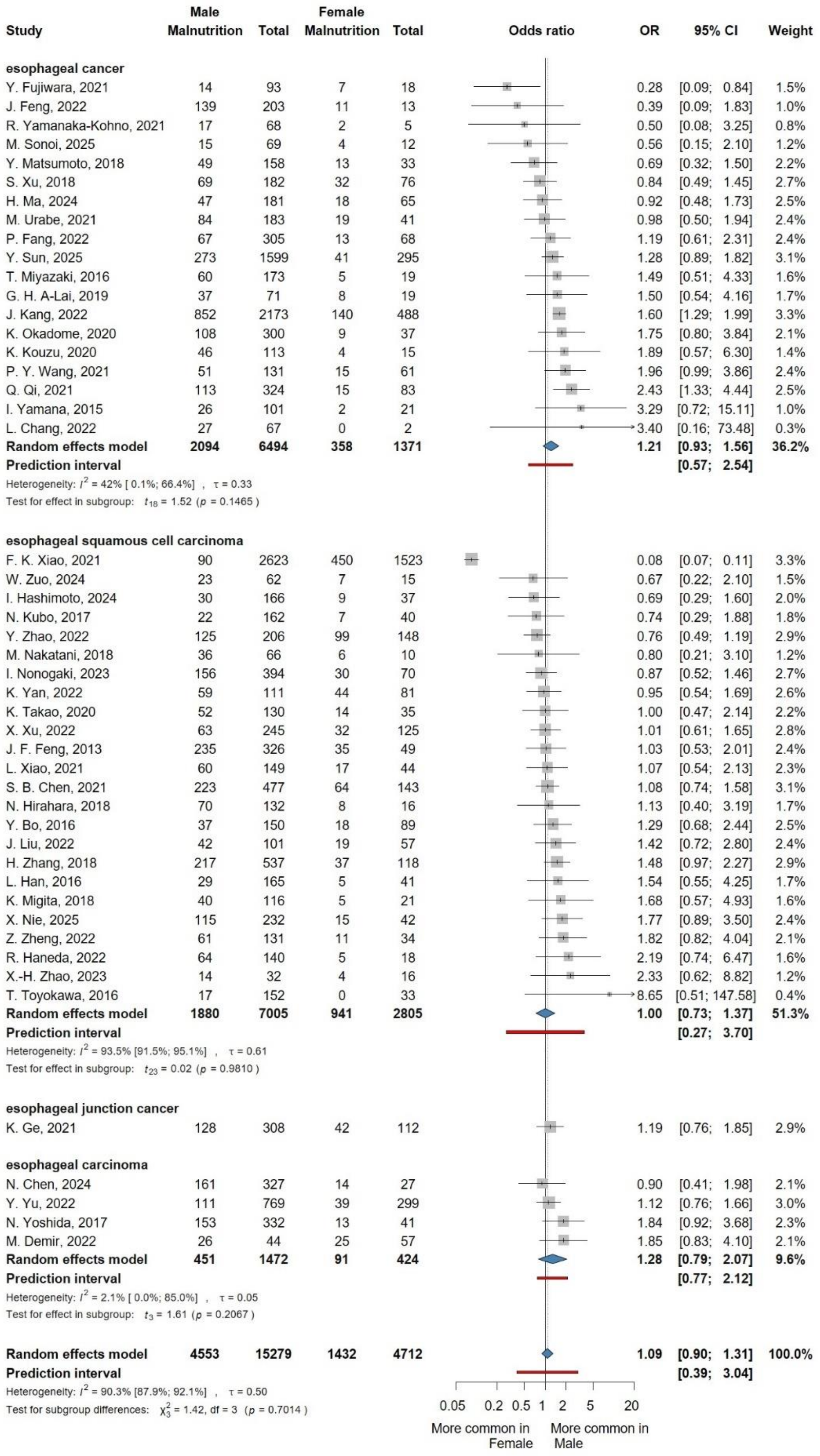

Figure S139. Assessment tool moderator analysis for the association between malnutrition-related complication risk and sex in esophageal cancer (Biological composite scores)

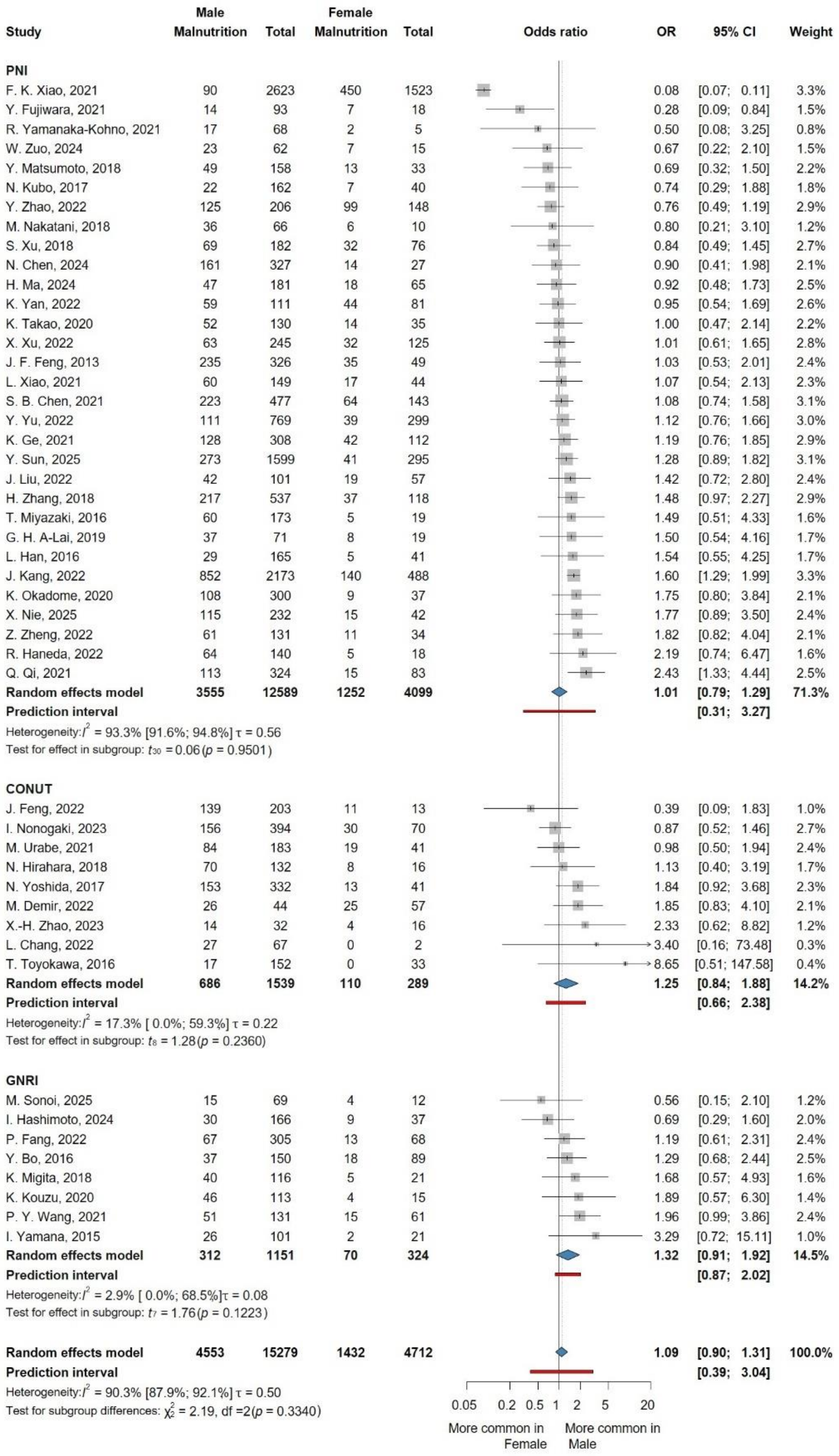

Abbreviations: PNI= Prognostic Nutritional Index, CONUT= Controlling Nutritional Status, GNRI= Geriatric Nutritional Risk Index

Figure S140. Cancer type moderator analysis for the association between malnutrition risk and sex in colorectal cancer (symptom-based tools)

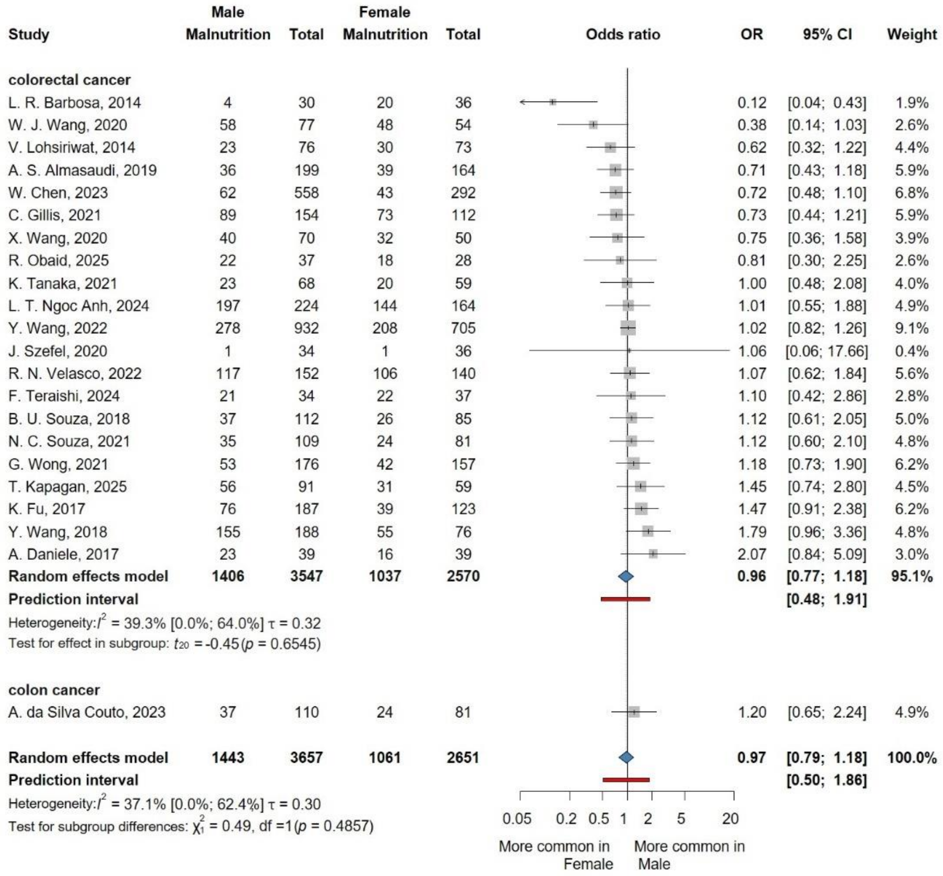

Figure S141. Resectability moderator analysis for the association between malnutrition risk and sex in colorectal cancer (symptom-based tools)

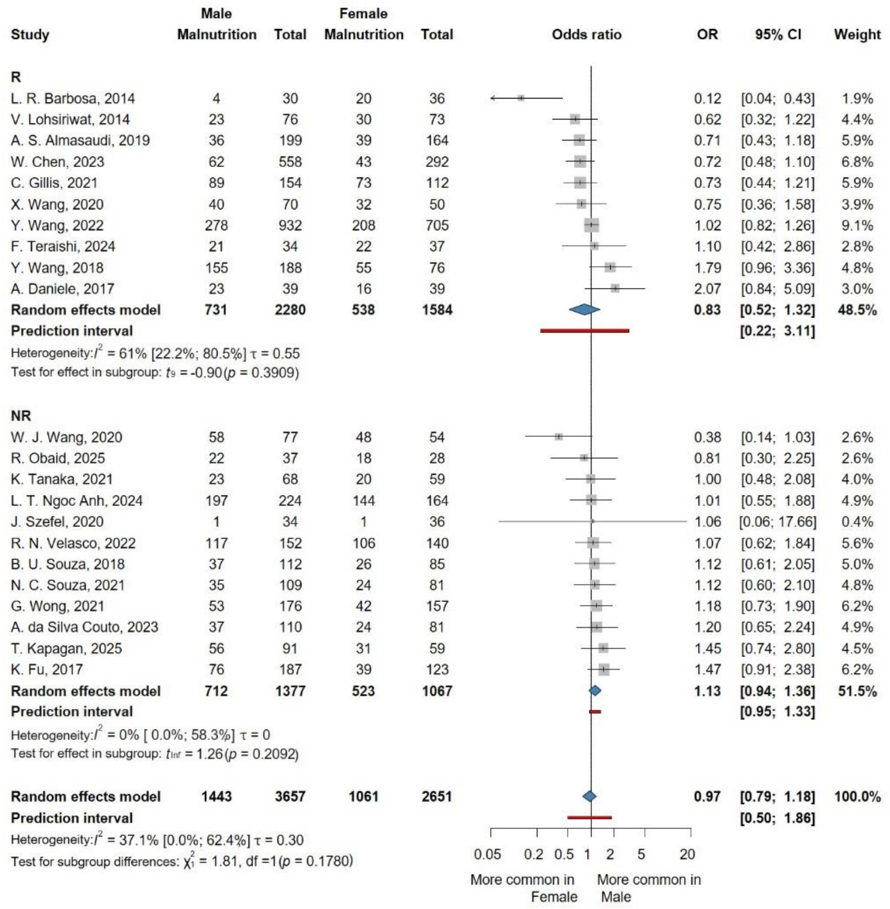

Abbreviations: R= resectable, NR= non-resectable

Figure S142. Assessment tool moderator analysis for the association between malnutrition risk and sex in colorectal cancer (symptom-based tools)

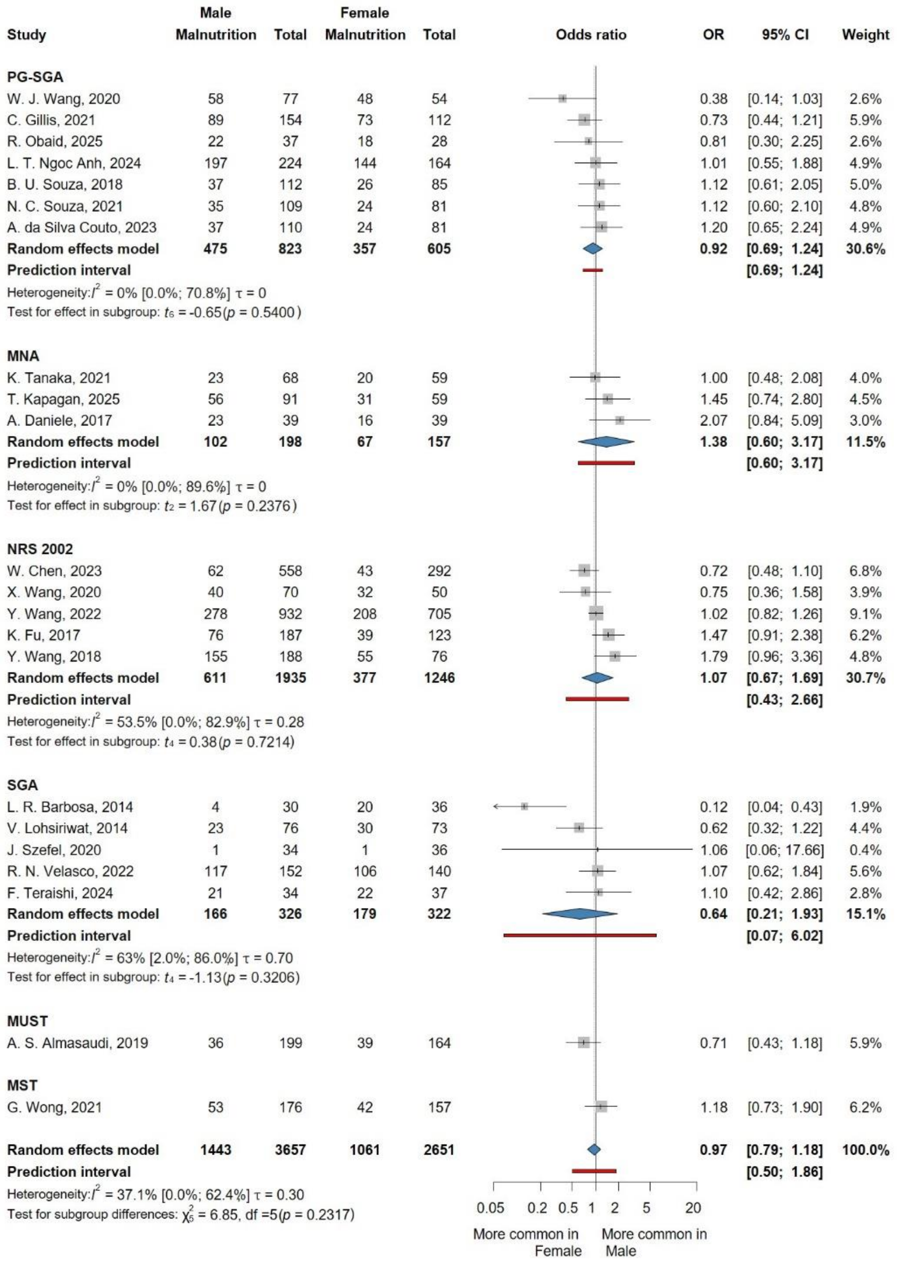

Abbreviations: PG-SGA= Patient-Generated Subjective Global Assessment, MNA= Mini Nutritional Assessment, NRS 2002= Nutrition Risk Screening 2002, SGA= Subjective Global Assessment, MUST= Malnutrition Universal Screening Tool, MST= Malnutrition Screening Tool

Figure S143. Resectability moderator analysis for the association between malnutrition-related complication risk and sex in gastric cancer (Biological composite scores)

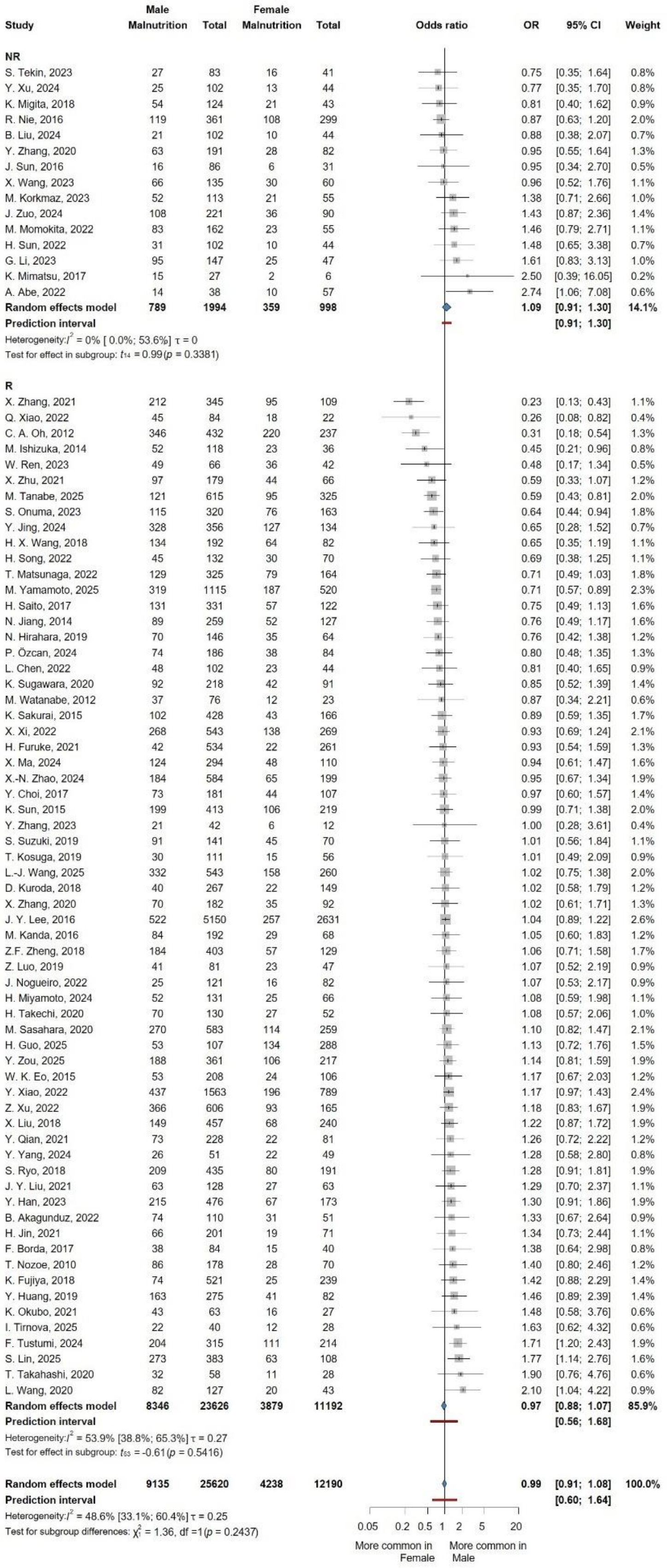

Abbreviations: R= resectable, NR= non-resectable

Figure S144. Age group moderator analysis for the association between malnutrition-related complication risk and sex in gastric cancer (Biological composite scores)

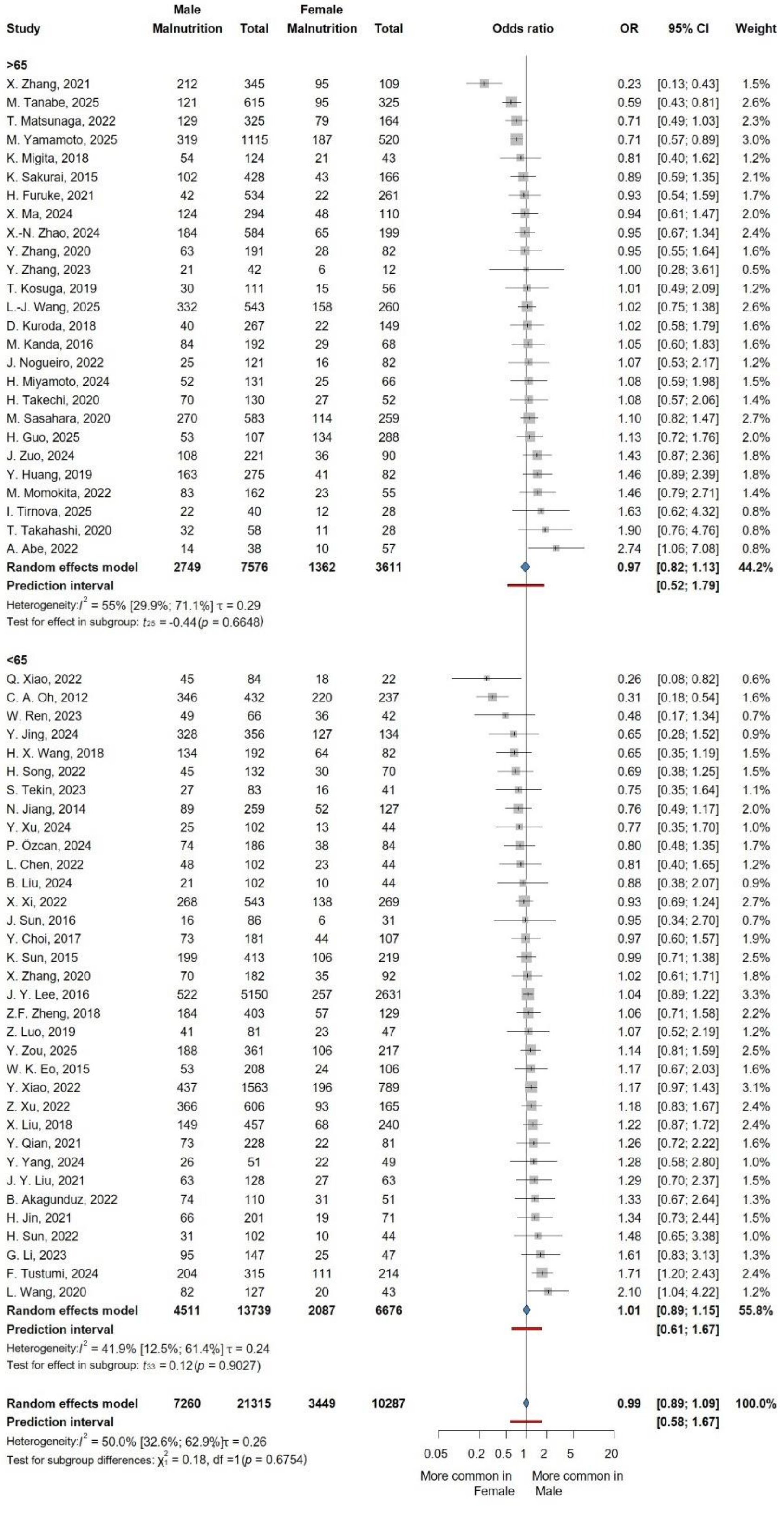

Figure S145. Assessment tool moderator analysis for the association between malnutrition-related complication risk and sex in gastric cancer (Biological composite scores)

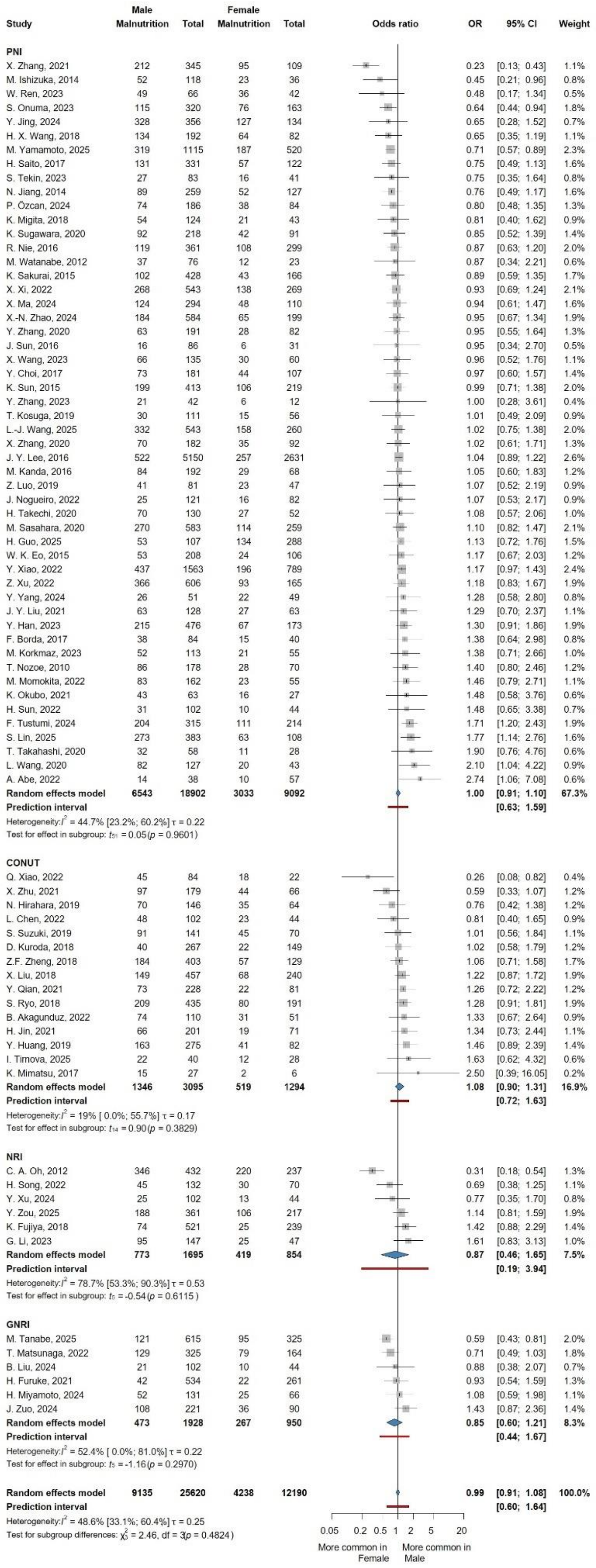

Abbreviations: PNI= Prognostic Nutritional Index, CONUT= Controlling Nutritional Status, GNRI= Geriatric Nutritional Risk Index, NRI = Nutritional Risk Index

Figure S146. Age group moderator analysis for the association between malnutrition-related complication risk and sex in resectable hepatocellular carcinoma (Biological composite scores)

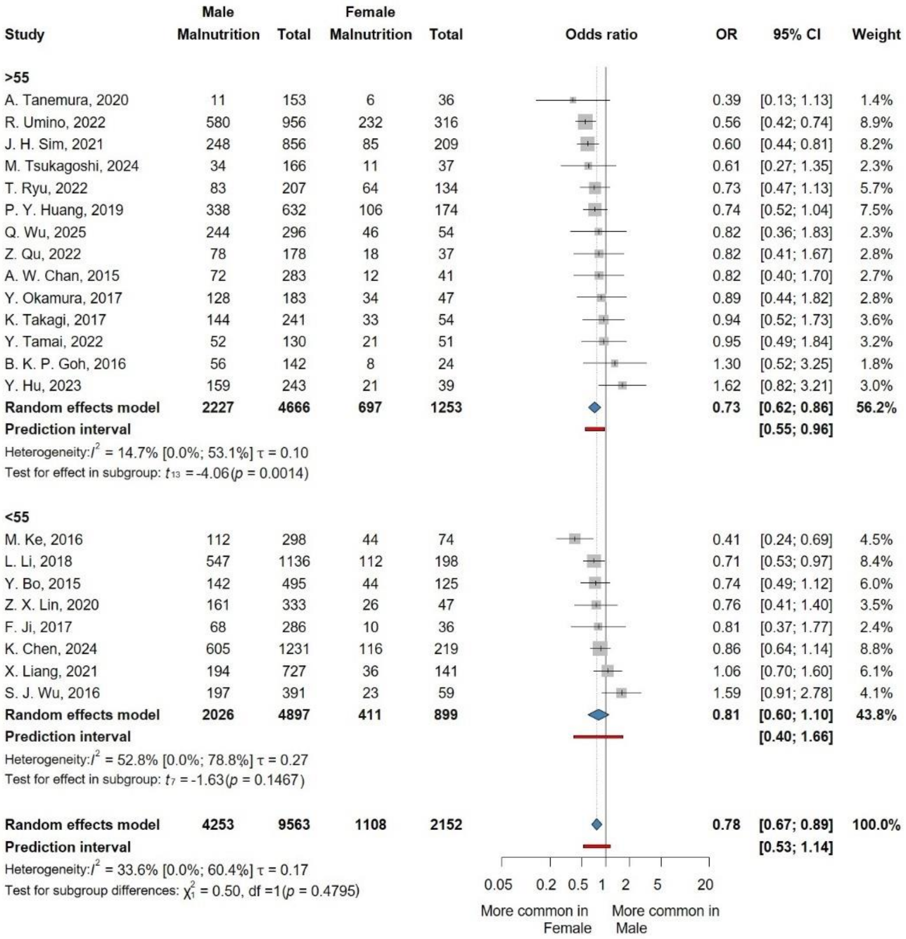

Figure S147. Assessment tool moderator analysis for the association between malnutrition-related complication risk and sex in resectable hepatocellular carcinoma (Biological composite scores)

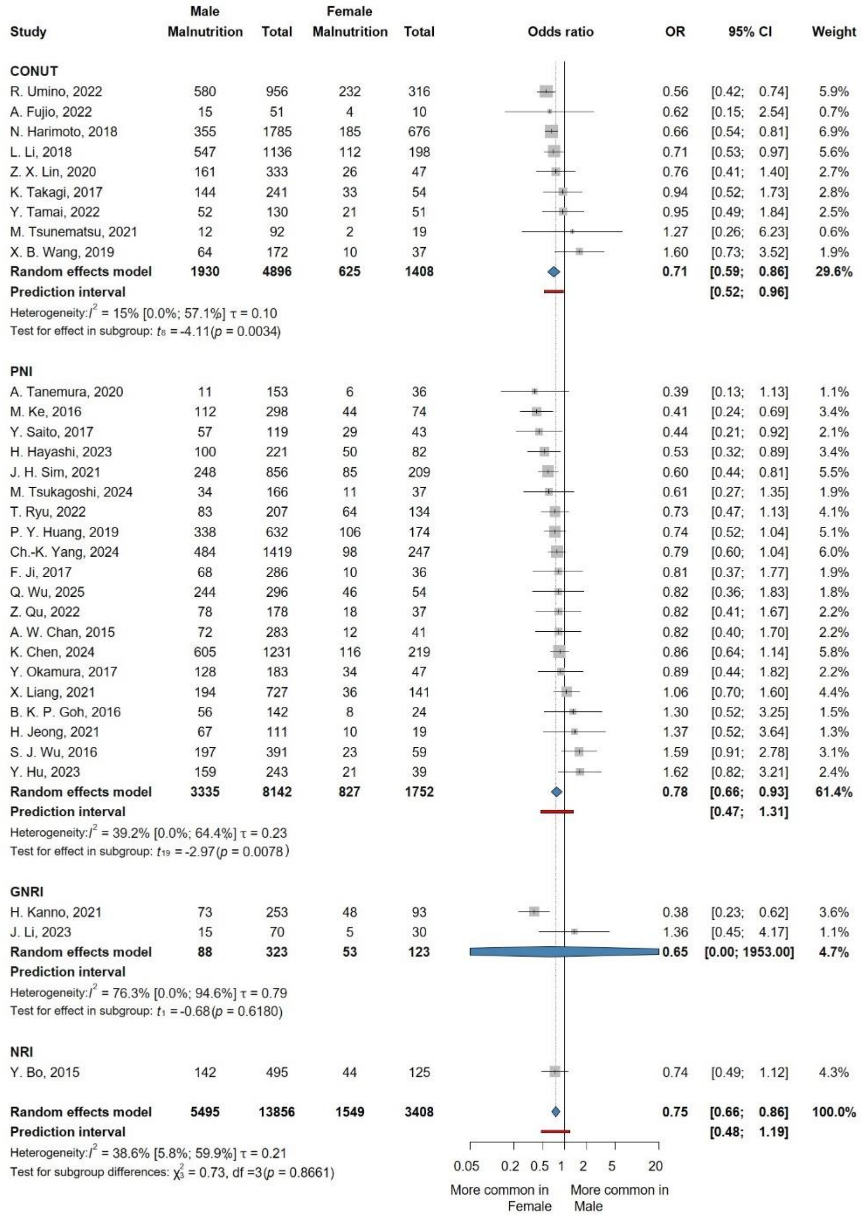

Abbreviations: PNI= Prognostic Nutritional Index, CONUT= Controlling Nutritional Status, GNRI= Geriatric Nutritional Risk Index, NRI = Nutritional Risk Index

Figure S148. Resectability moderator analysis for the association between malnutrition-related complication risk and serum CA19-9 level in colorectal cancer (Biological composite scores)

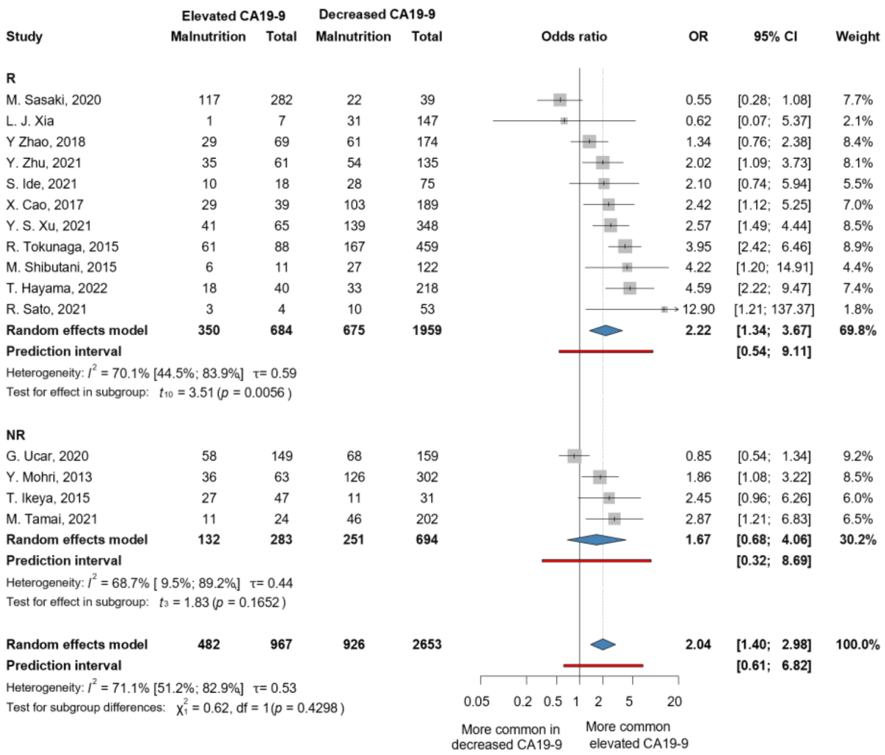

Abbreviations: R= resectable, NR= non-resectable

Figure S149. Cancer type moderator analysis for the association between malnutrition-related complication risk and serum CA19-9 level in colorectal cancer (Biological composite scores)

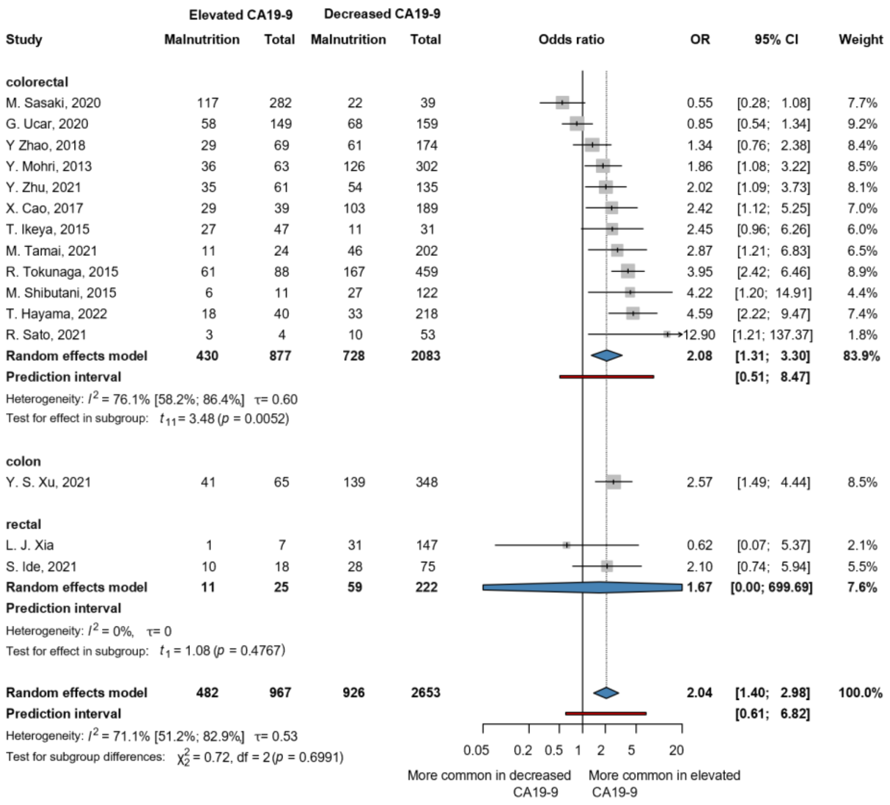

Figure S150. Assessment tool moderator analysis for the association between malnutrition-related complication risk and serum CA19-9 level in colorectal cancer (Biological composite scores)

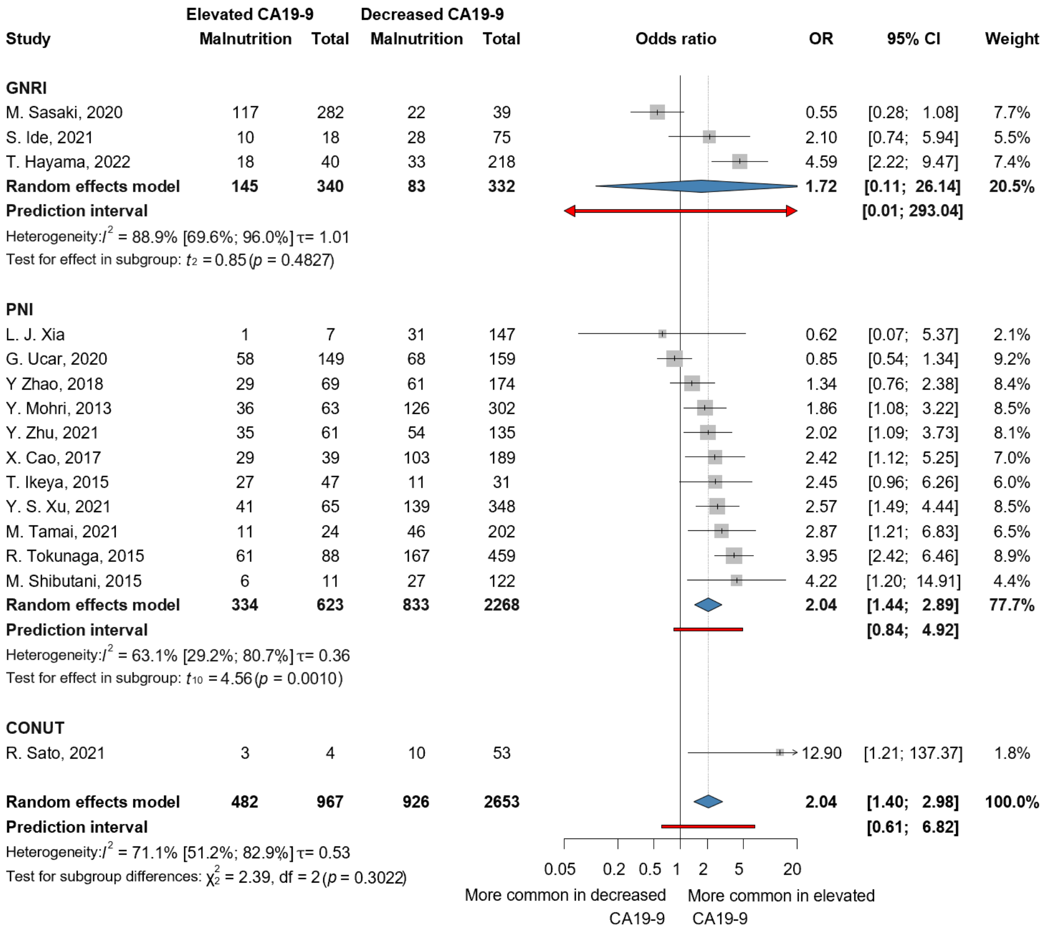

Abbreviations: PNI= Prognostic Nutritional Index, CONUT= Controlling Nutritional Status, GNRI= Geriatric Nutritional Risk Index

Figure S151. Female proportion meta regression analysis for the association between malnutrition-related complication risk and serum CA19-9 level in colorectal cancer (Biological composite scores)

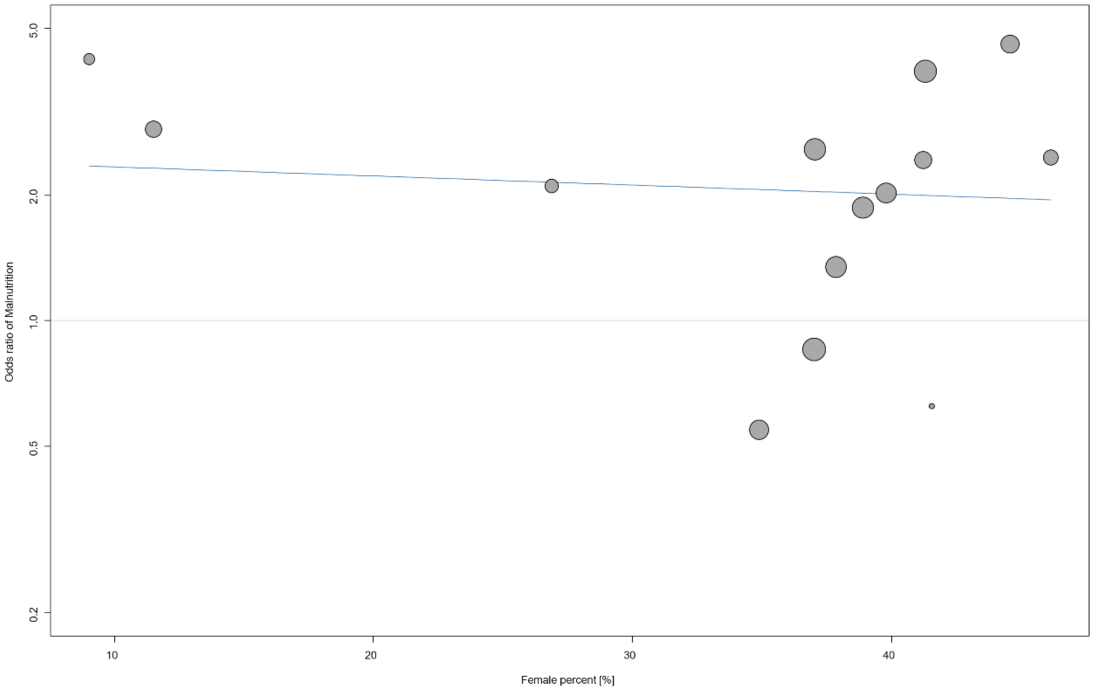

Assuming a linear relation, the estimated slope is -0.005 [95% CI: -0.0453 - 0.0352] [log(OR of Malnutrition) / female %] (p-value: 0.7907). NOTE: it is given in the log scale!

Figure S152. Resectability moderator analysis for the association between malnutrition-related complication risk and T stage in esophageal cancer (Biological composite scores)

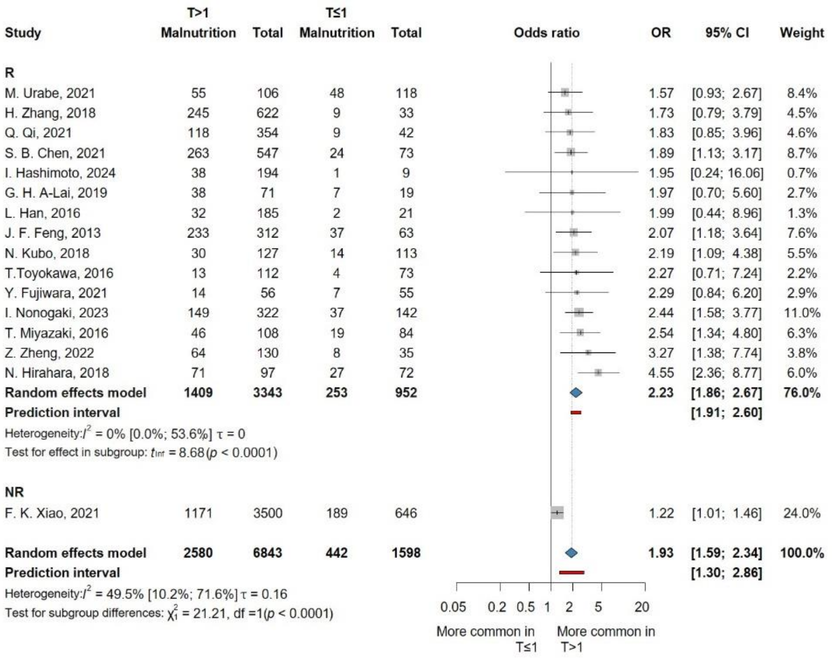

Abbreviations: R= resectable, NR= non-resectable

Figure S153. Cancer type moderator analysis for the association between malnutrition-related complication risk and T stage in esophageal cancer (Biological composite scores)

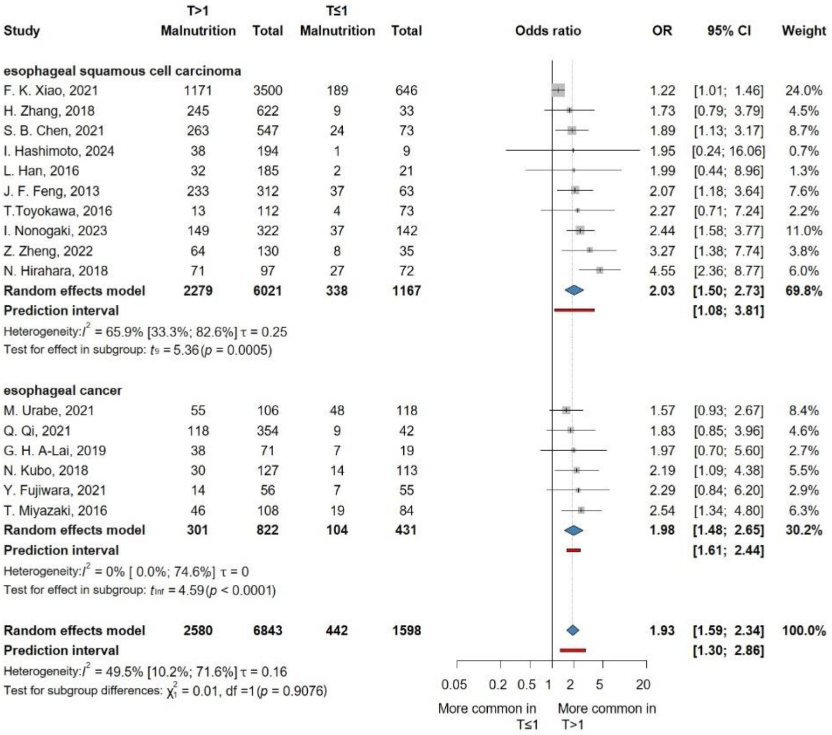

Figure S154. Assessment tool moderator analysis for the association between malnutrition-related complication risk and T stage in esophageal cancer (Biological composite scores)

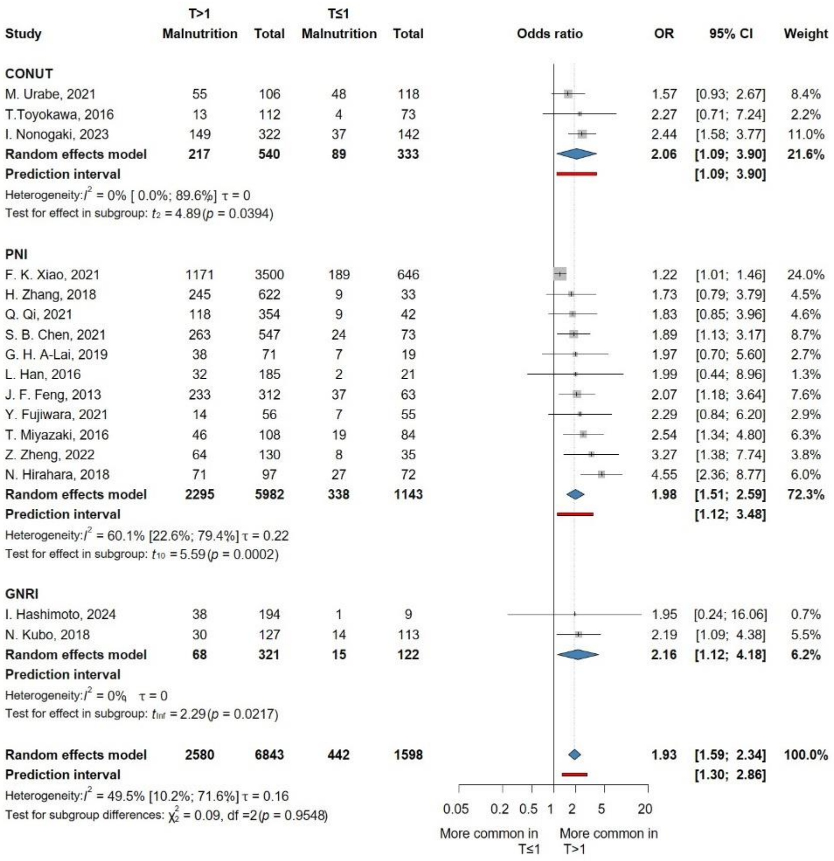

Abbreviations: PNI= Prognostic Nutritional Index, CONUT= Controlling Nutritional Status, GNRI= Geriatric Nutritional Risk Index

Figure S155. TNM staging edition moderator analysis for association between malnutrition-related complication risk and T stage in esophageal cancer (Biological composite scores)

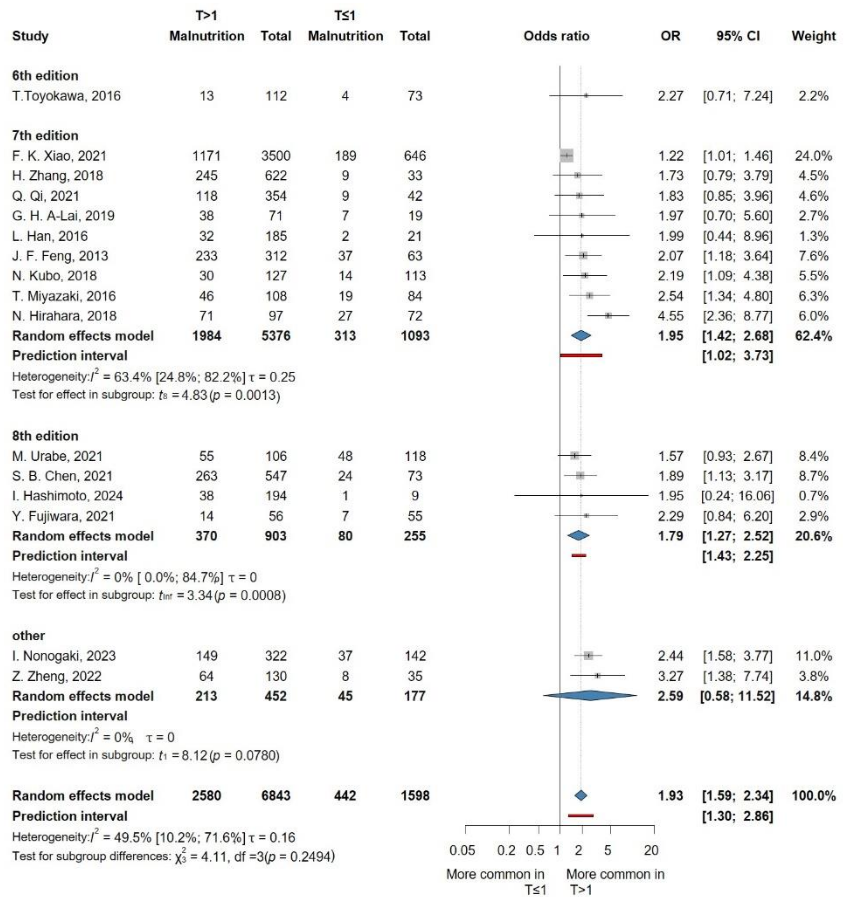

Figure S156. Female proportion meta regression analysis for the association between malnutrition-related complication risk and T stage in esophageal cancer (Biological composite scores)

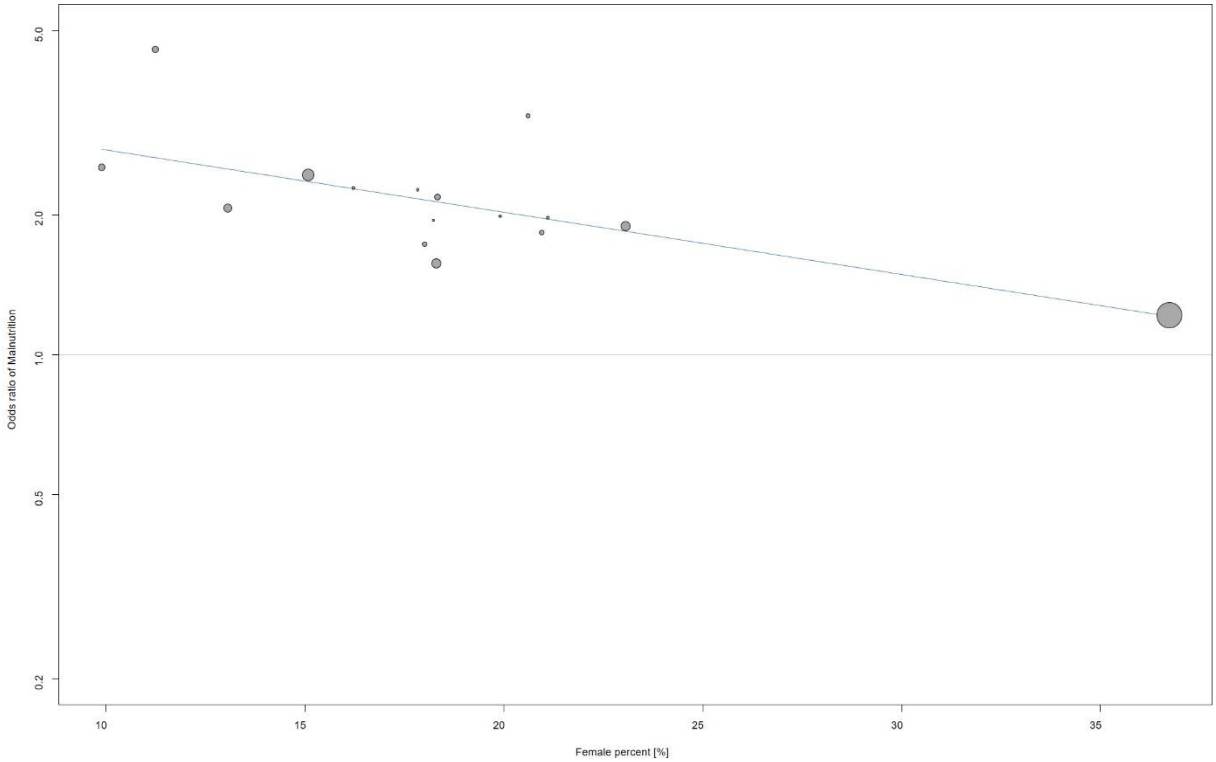

Assuming a linear relation, the estimated slope is -0.0309 [95% CI: -0.0399 - -0.0219] [log(OR of Malnutrition) / female %] (p-value: <0.0001). NOTE: it is given in the log scale!

Figure S157. Assessment tool moderator analysis for association between malnutrition-related complication risk and T stage in gastric cancer (Biological composite scores)

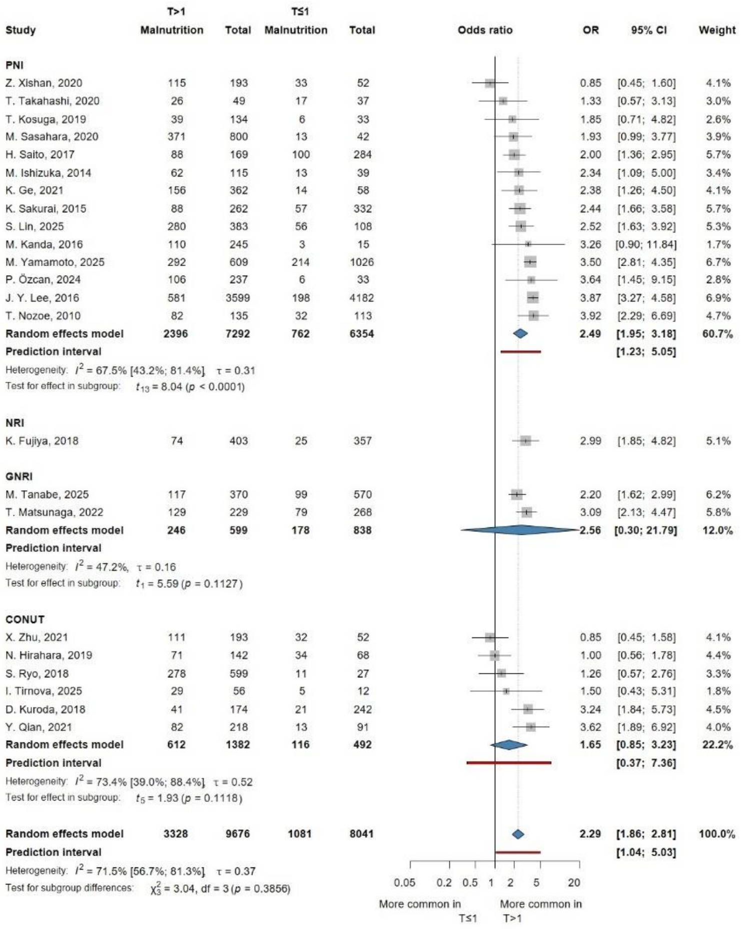

Abbreviations: PNI= Prognostic Nutritional Index, CONUT= Controlling Nutritional Status, GNRI= Geriatric Nutritional Risk Index, NRI= Nutritional Risk Index

Figure S158. TNM staging edition moderator analysis for association between malnutrition-related complication risk and T stage in gastric cancer (Biological composite scores)

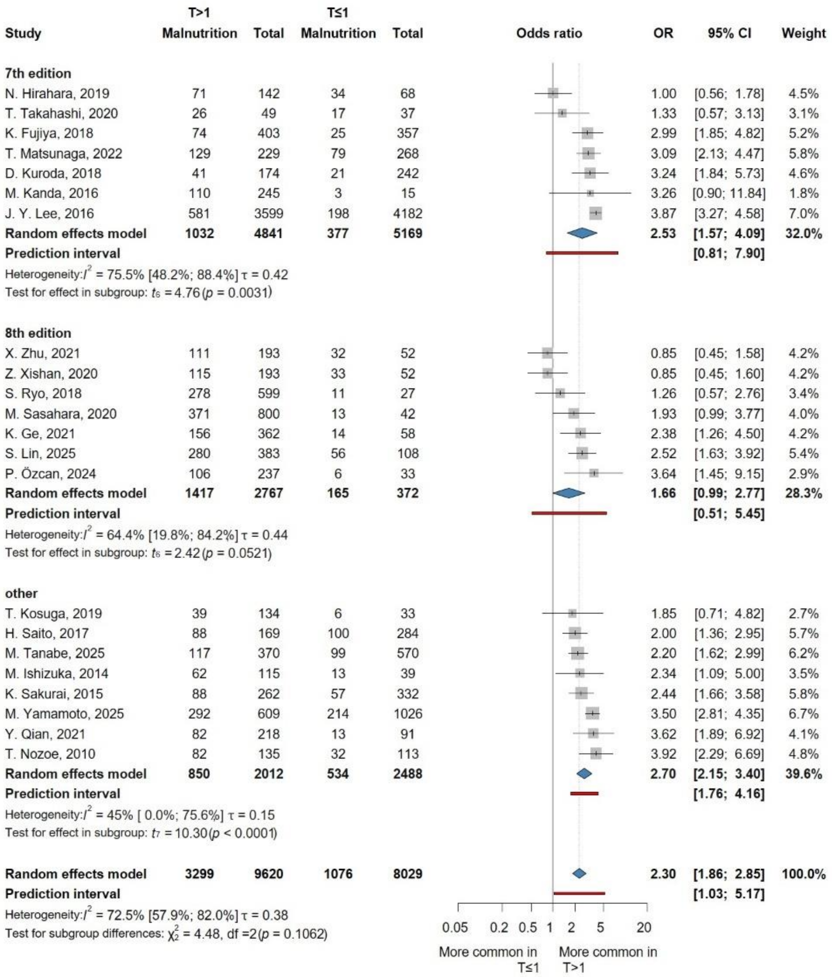

Figure S159. Female proportion meta regression analysis for association between malnutrition-related complication risk and T stage in gastric cancer (Biological composite scores)

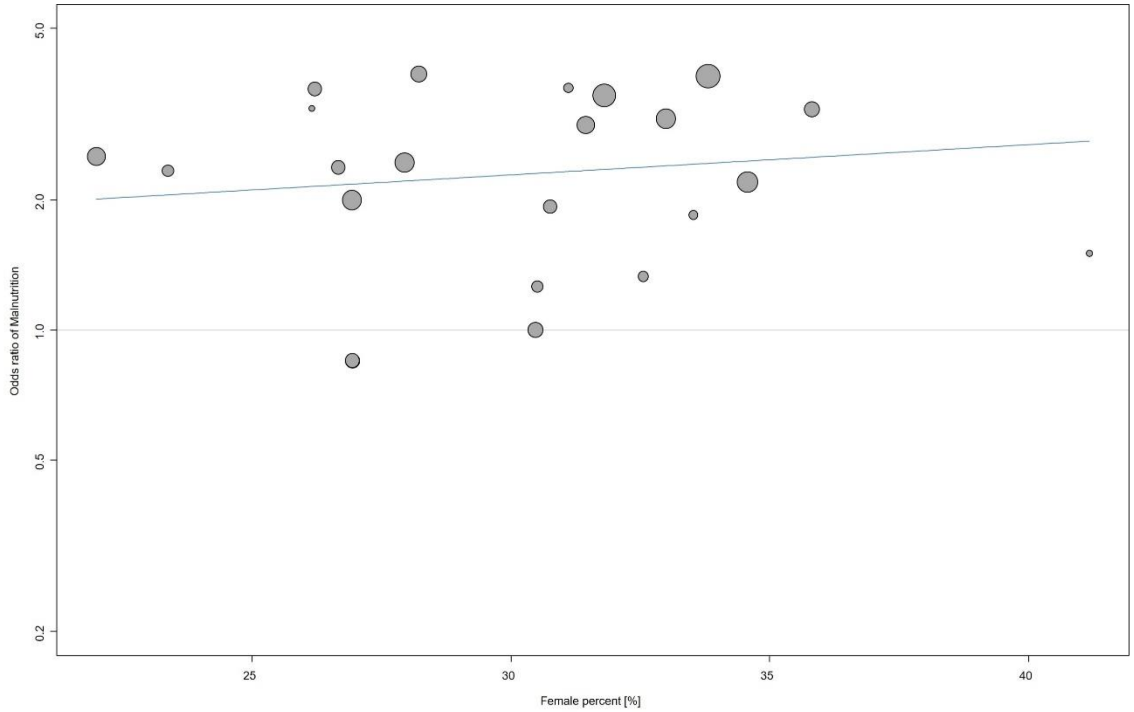

Assuming a linear relation, the estimated slope is 0.0162 [95% CI: -0.037 - 0.0694] [log(OR of Malnutrition) / female %] (p-value: 0.5335). NOTE: it is given in the log scale!

Figure S160. Assessment tool moderator analysis for association between malnutrition-related complication risk and N stage in esophageal cancer (Biological composite scores)

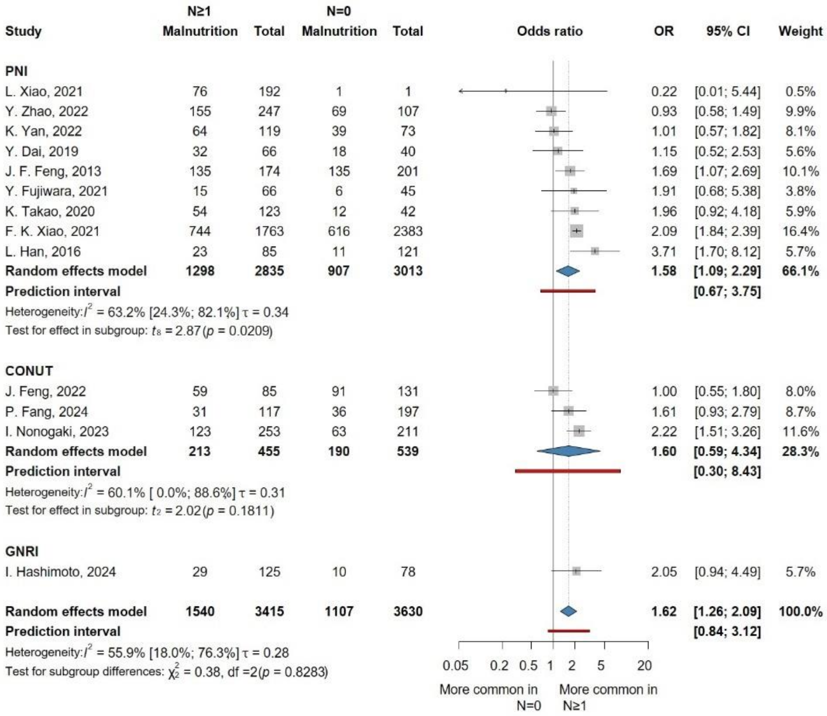

Abbreviations: PNI= Prognostic Nutritional Index, CONUT= Controlling Nutritional Status,

Figure S161. Resectability moderator analysis for association between malnutrition-related complication risk and N stage in esophageal cancer (Biological composite scores)

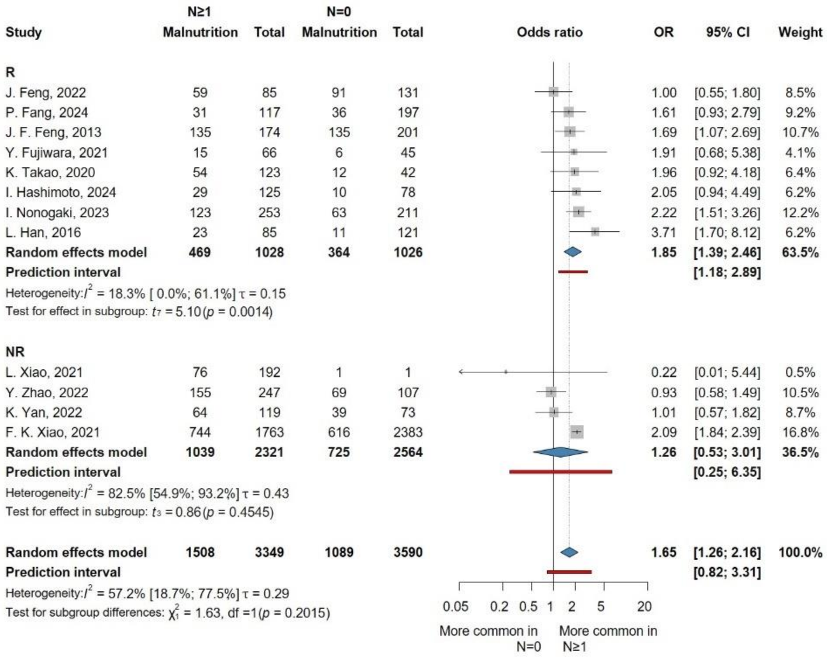

Abbreviations: R= resectable, NR= non-resectable

Figure S162. TNM staging edition moderator analysis for association between malnutrition-related complication risk and N stage in esophageal cancer (Biological composite scores)

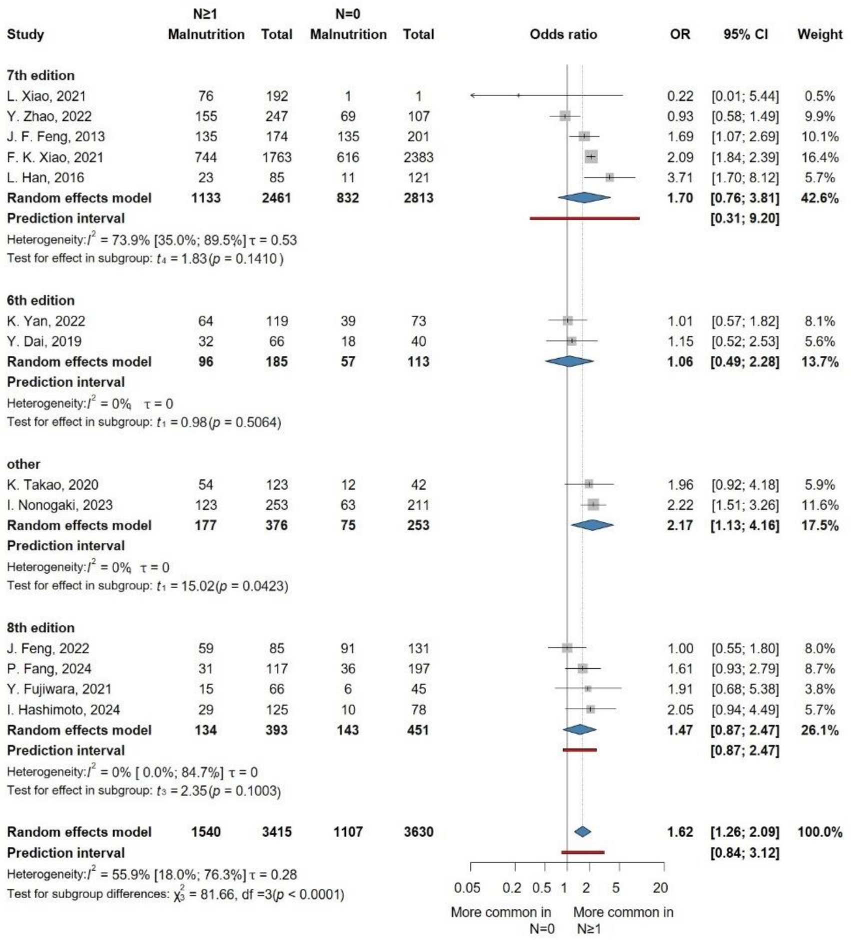

Figure S163. Female proportion meta regression analysis for association between malnutrition-related complication risk and N stage in esophageal cancer (Biological composite scores)

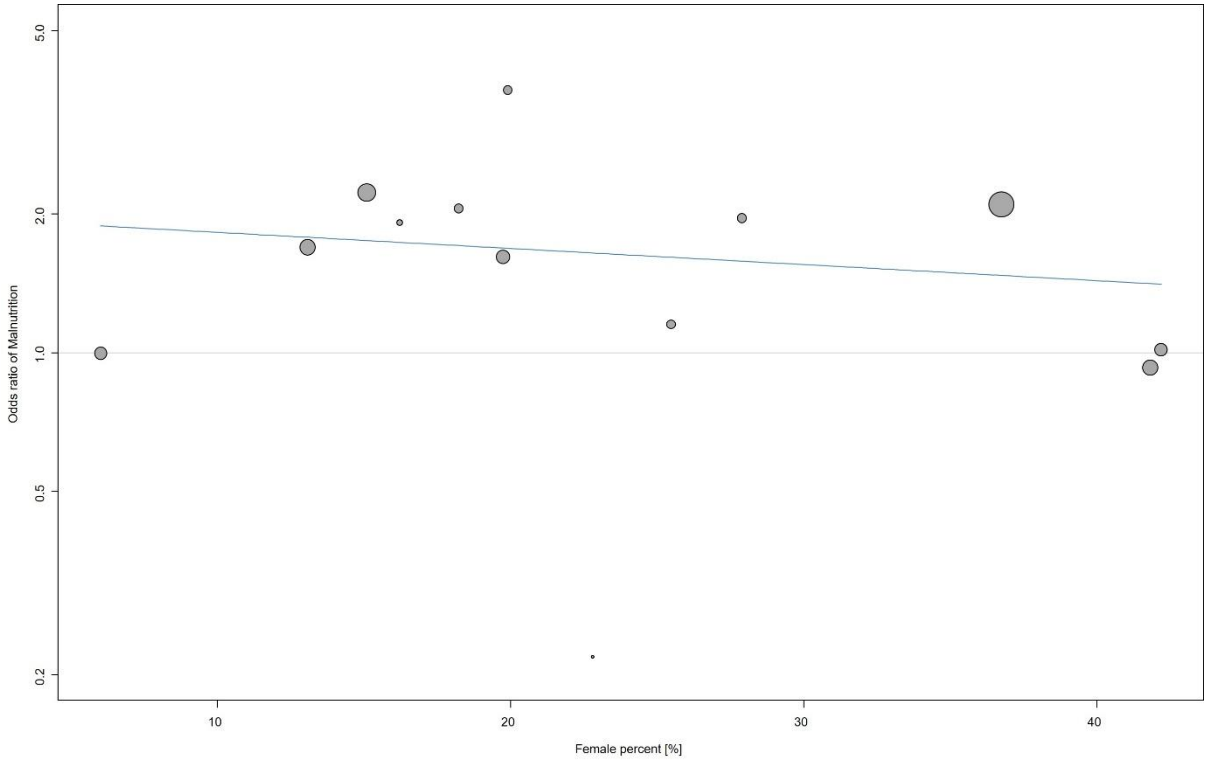

Assuming a linear relation, the estimated slope is -0.0081 [95% CI: -0.0302 - 0.014] [log(OR of Malnutrition) / female %] (p-value: 0.4374). NOTE: it is given in the log scale!

Figure S164. Assessment tool moderator analysis for association between malnutrition-related complication risk and N stage in gastric cancer (Biological composite scores)

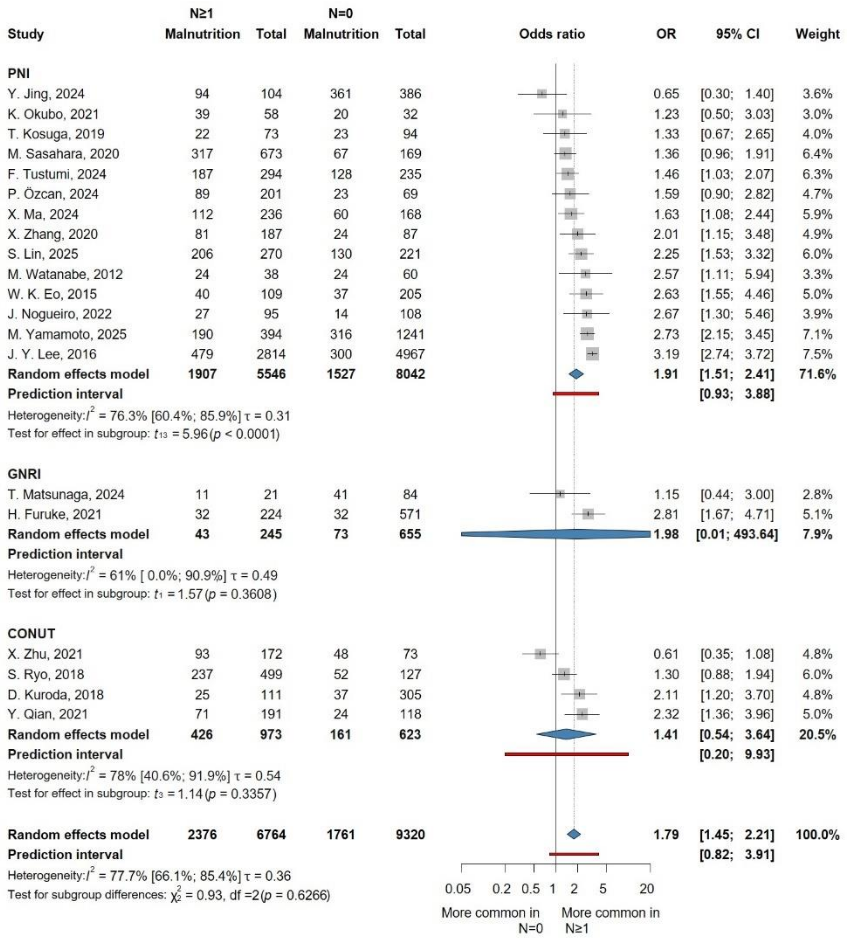

Abbreviations: PNI= Prognostic Nutritional Index, CONUT= Controlling Nutritional Status, GNRI= Geriatric Nutritional Risk Index

Figure S165. TNM staging edition moderator analysis for association between malnutrition-related complication risk and N stage in gastric cancer (Biological composite scores)

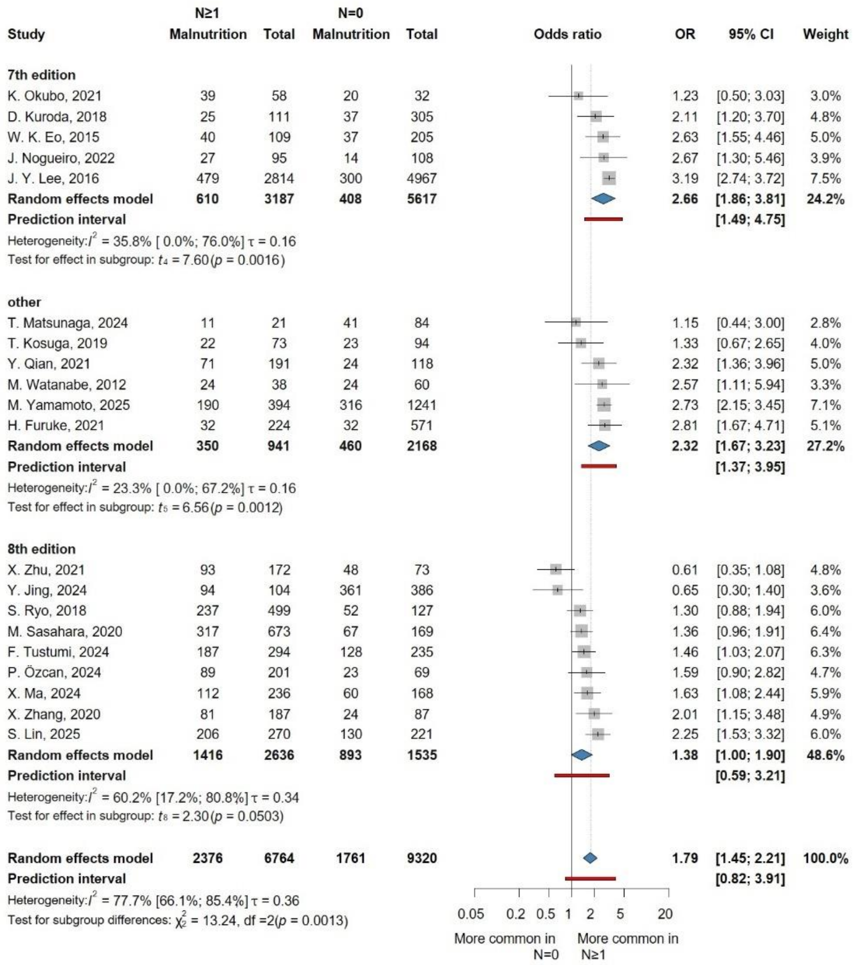

Figure S166. Female proportion meta regression analysis for association between malnutrition-related complication risk and N stage in gastric cancer (Biological composite scores)

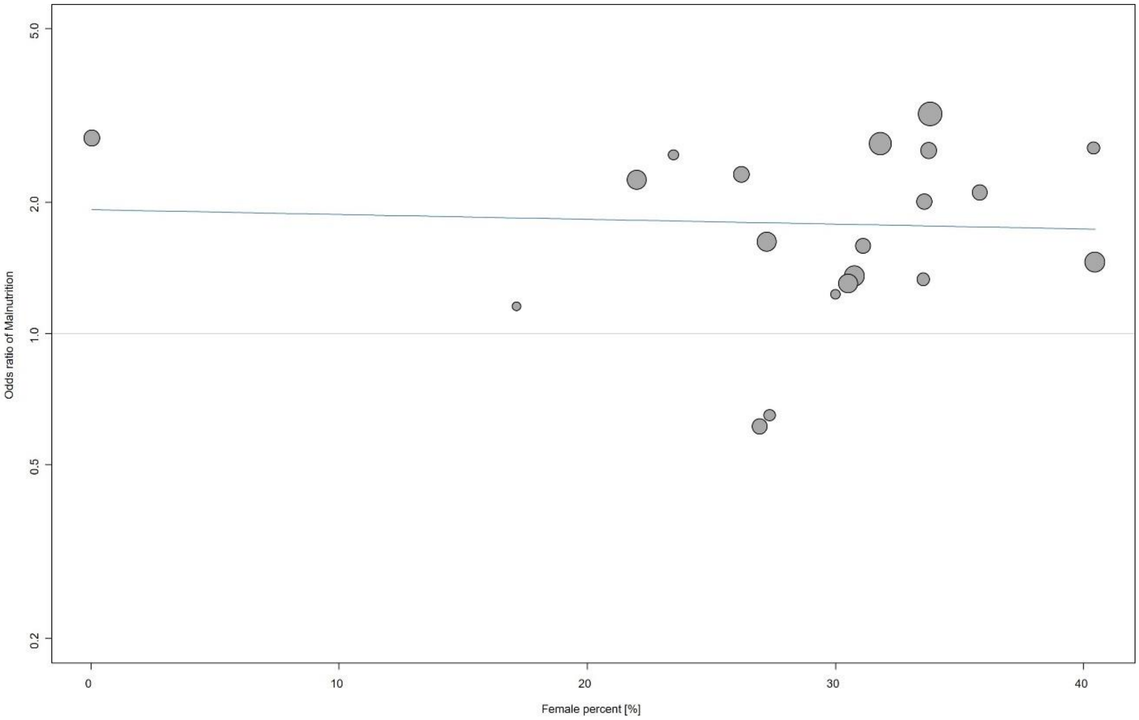

Assuming a linear relation, the estimated slope is -0.0026 [95% CI: -0.0281 - 0.0229] [log(OR of Malnutrition) / female %] (p-value: 0.8344). NOTE: it is given in the log scale!

Figure S167. Assessment tool moderator analysis for association between malnutrition-related complication risk and tumor location in colorectal cancer (Biological composite scores)

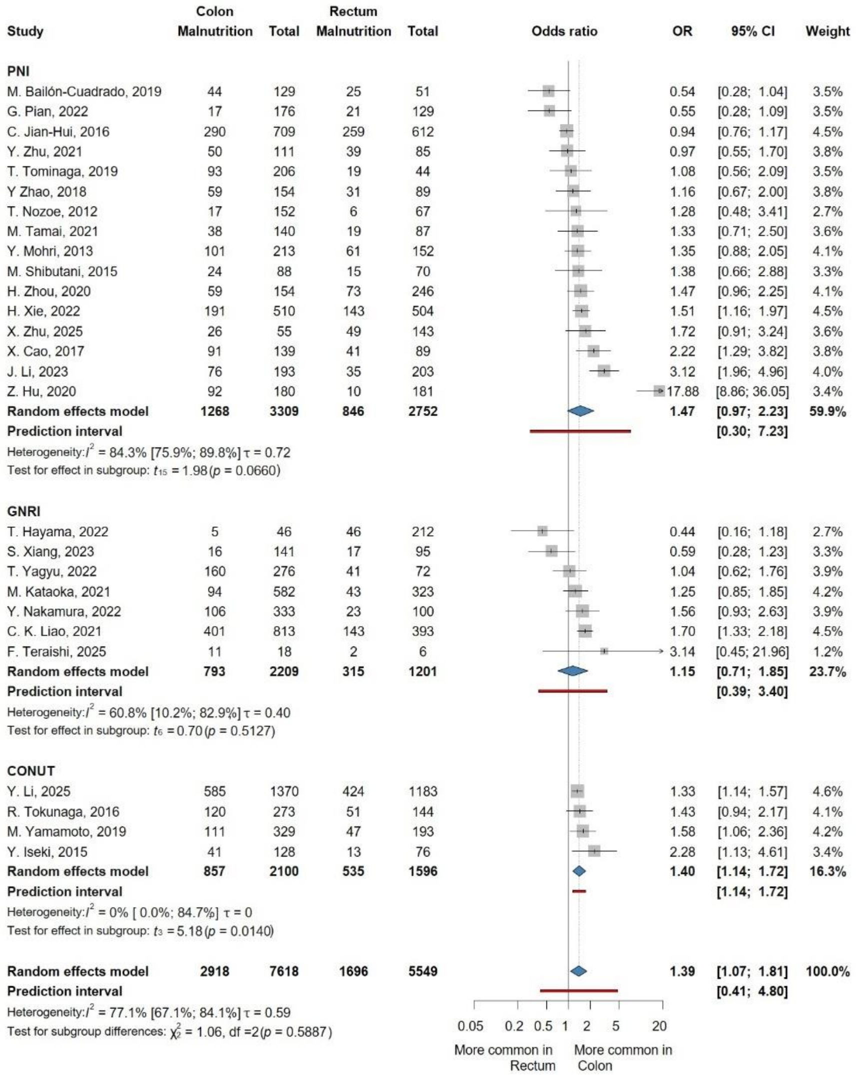

Abbreviations: PNI= Prognostic Nutritional Index, CONUT= Controlling Nutritional Status, GNRI= Geriatric Nutritional Risk Index

Figure S168. Age group moderator analysis for association between malnutrition-related complication risk and tumor location in colorectal cancer (Biological composite scores)

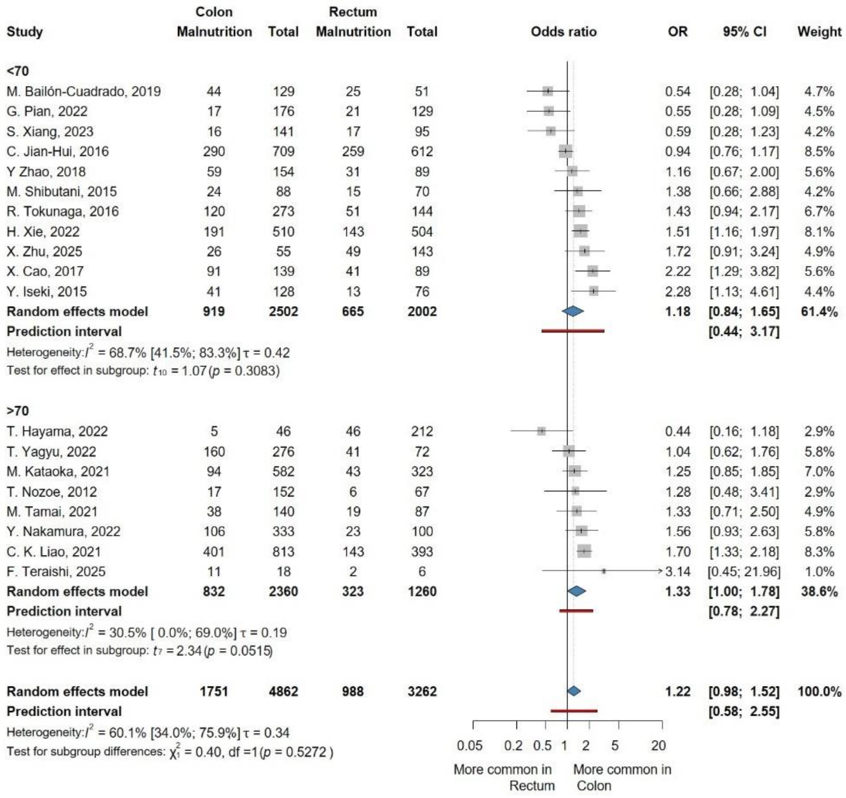

Figure S169. Female proportion meta regression analysis for association between malnutrition-related complication risk and tumor location in colorectal cancer (Biological composite scores)

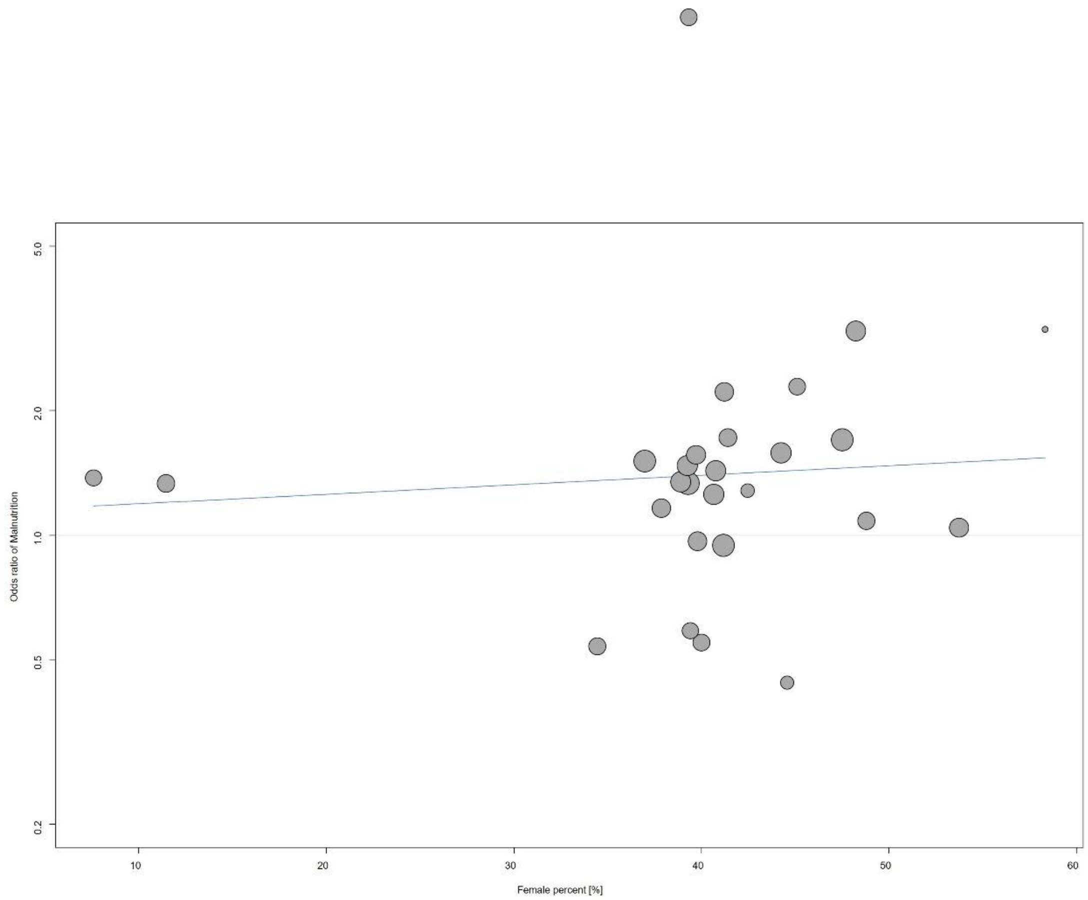

Assuming a linear relation, the estimated slope is 0.0053 [95% CI: -0.0231 - 0.0337] [log(OR of Malnutrition) / female %] (p-value: 0.704). NOTE: it is given in the log scale!

Figure S170. Assessment tool moderator analysis for association between malnutrition-related complication risk and neoadjuvant therapy in upper gastrointestinal cancer (Biological composite scores)

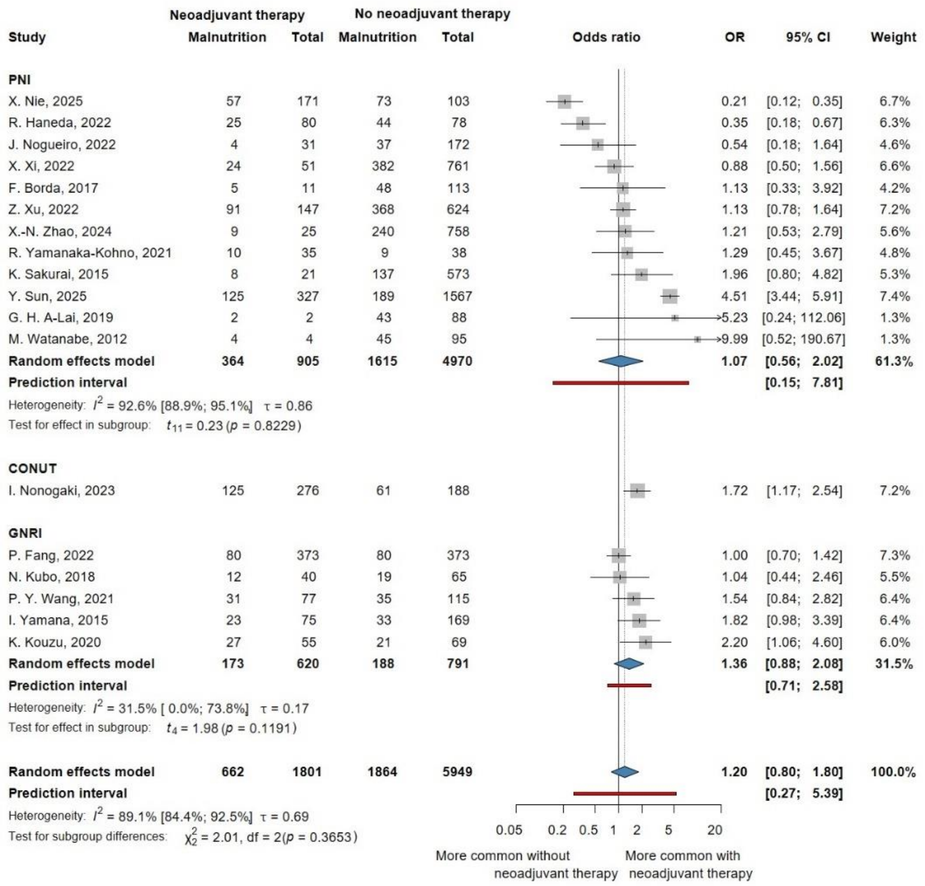

Abbreviations: PNI= Prognostic Nutritional Index, CONUT= Controlling Nutritional Status, GNRI= Geriatric Nutritional Risk Index

Figure S171. Cancer type moderator analysis for association between malnutrition-related complication risk and neoadjuvant therapy in upper gastrointestinal cancer (Biological composite scores)

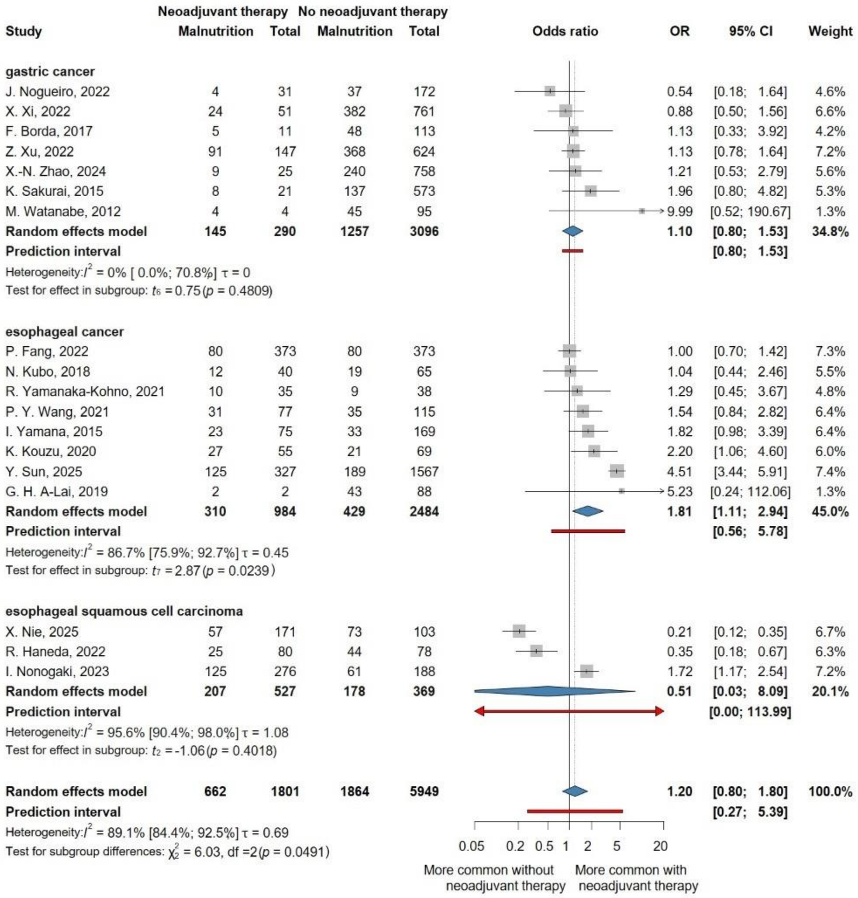

Figure S172. Female proportion meta regression analysis for association between malnutrition-related complication risk and neoadjuvant therapy in upper gastrointestinal cancer (Biological composite scores)

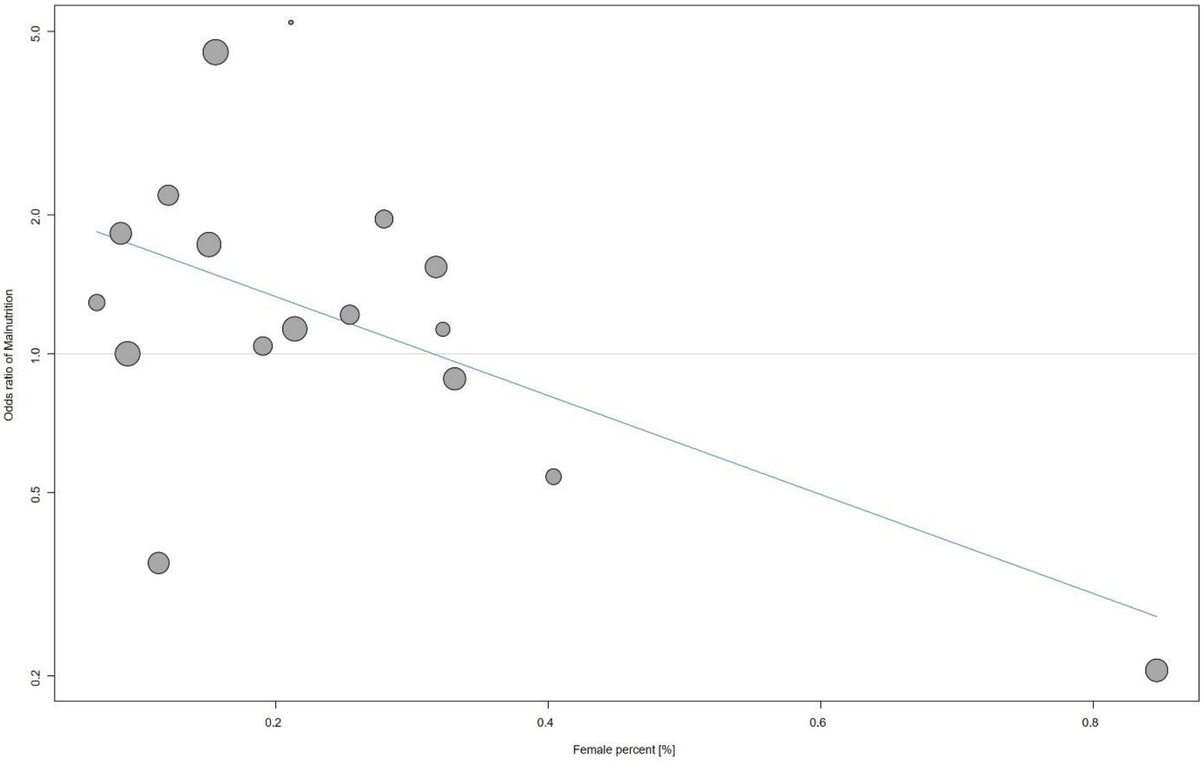

Assuming a linear relation, the estimated slope is -2.4711 [95% CI: -4.2362 - -0.706] [log(OR of Malnutrition) / female %] (p-value: 0.0091). NOTE: it is given in the log scale!

Supplementary Document S15: Publication bias

Publication bias was assessed by visual inspection of the funnel plots and Egger's test. Publication bias is judged to be meaningful if at least 10 articles are in the analysis.
The Egger's test indicated potential publication bias in the following cases (see figures in Document S10):

- **Figure 182**: Association between malnutrition-related complication risk and hypertension in gastrointestinal cancer (Biological composite scores) [Egger test: t = -2.06, df = 15, p-value = 0.0568]
- **Figure 184**: Association between malnutrition-related complication risk and N stage (N0 vs. N≥1) in gastric cancer (Biological composite scores) [Egger test: t = -2.32, df = 18, p-value = 0.0320]
- **Figure 194**: Association between malnutrition-related complication risk and T stage (T>1 vs. T≤1) in esophageal cancer (Biological composite scores) [Egger test: t = 4.44, df = 14, p-value = 0.0006]
- **Figure 196**: Association between malnutrition-related complication risk and T stage (T>1 vs. T≤1) in gastric cancer (Biological composite scores) [Egger test: t = -2.51, df = 21, p-value = 0.0204]
- **Figure 197**: Association between malnutrition-related complication risk and T stage (T4 vs. <T4) in gastric cancer (Biological composite scores) [Egger test: t = -2.07, df = 20, p-value = 0.0520]
- **Figure 206**: Association between malnutrition risk and sex in esophageal cancer (Symptom-based risk assessment tools) [Egger test: t = -2.67, df = 9, p-value = 0.0254]

In case of **Figure 182,** we see that study with a large standard error (small sample size) tend to bias towards a lower OR. If articles were published with a small standard error (large sample size), this would tend to bias towards a higher OR.

In case of **Figure 184,** we see that studies with a large standard error (small sample size) appear lower or nonsignificant effects to be underrepresented, while studies with a small standard error (large sample size) tend to bias towards a higher OR.

In the case of **Figure 194**, The analyses rather indicate that there is an article with an extreme standard error and showing a different effect. In case of Figure 22 this article is F.K. Xiao, 2022. Also, studies with a small standard error (large sample size) tend to bias towards a higher OR.

In the case of **Figure 196**, the analyses rather indicate that the effect differs between two different sets of articles (an unidentified subgroup), and this is why the p-value of the Egger test is small and not because of a possible publication bias.

In the case of **Figure 197** the suspicion of publication bias is indeed stronger, and in each instance, we see that studies with a large standard error (small sample size) tend to bias towards a higher OR. If articles were published with a small standard error (large sample size), this would tend to bias towards a lower OR.

In the case of **Figure 206,** we see that studies with a large standard error (small sample size) tend to bias towards a lower OR.

Supplementary Document S16: Funnel plots for the publication bias and leave-one-out plots: Figures S173-S227

Figure S173: Funnel plot: Association between malnutrition-related complication risk and T stage (T4 vs. <T4) in colorectal cancer (Biological composite scores)

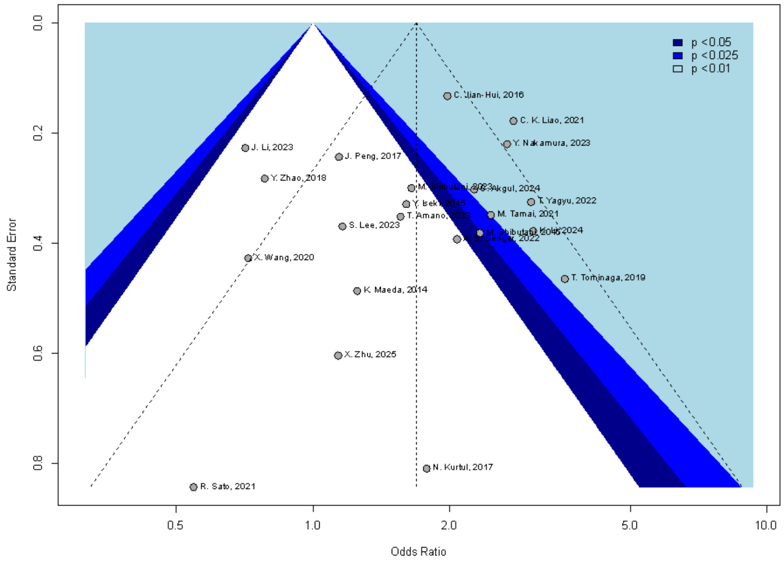

Egger test: t = -1.81, df = 20, p-value = 0.0851

Figure S173.1: Leave-one-out plot: Association between malnutrition-related complication risk and T stage (T4 vs. <T4) in colorectal cancer (Biological composite scores)

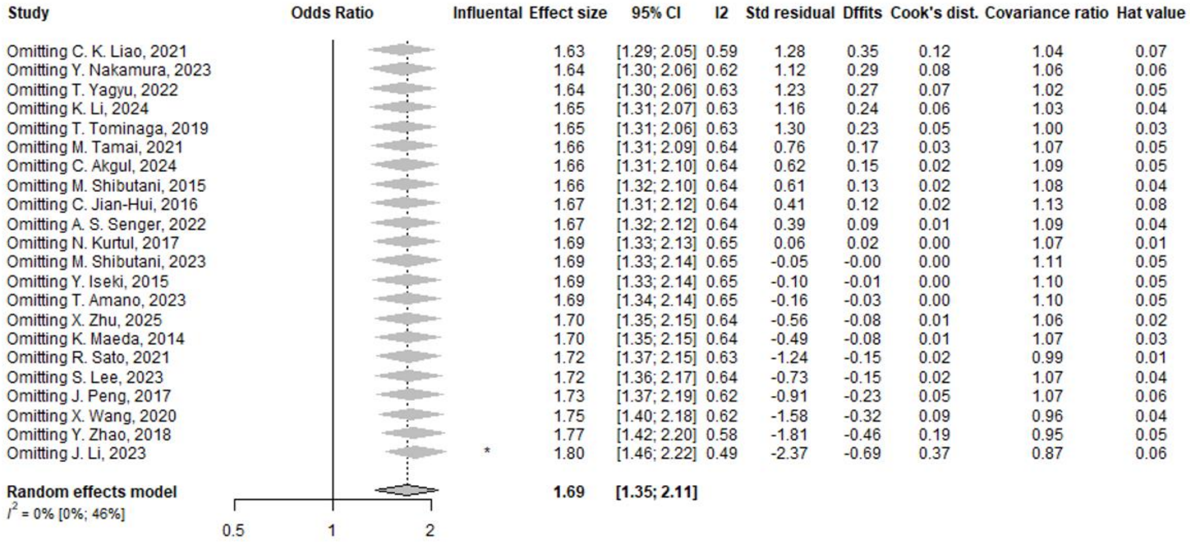

Study	Influental	Effect size	95% CI	I2	Std residual	Dffits	Cook's dist.	Covariance ratio	Hat value
Omitting C. K. Liao, 2021		1.63	[1.29; 2.05]	0.59	1.28	0.35	0.12	1.04	0.07
Omitting Y. Nakamura, 2023		1.64	[1.30; 2.06]	0.62	1.12	0.29	0.08	1.06	0.06
Omitting T. Yagyu, 2022		1.64	[1.30; 2.06]	0.63	1.23	0.27	0.07	1.02	0.05
Omitting K. Li, 2024		1.65	[1.31; 2.07]	0.63	1.16	0.24	0.06	1.03	0.04
Omitting T. Tominaga, 2019		1.65	[1.31; 2.06]	0.63	1.30	0.23	0.05	1.00	0.03
Omitting M. Tamai, 2021		1.66	[1.31; 2.09]	0.64	0.76	0.17	0.03	1.07	0.05
Omitting C. Akgul, 2024		1.66	[1.31; 2.10]	0.64	0.62	0.15	0.02	1.09	0.05
Omitting M. Shibutani, 2015		1.66	[1.32; 2.10]	0.64	0.61	0.13	0.02	1.08	0.04
Omitting C. Jian-Hui, 2016		1.67	[1.31; 2.12]	0.64	0.41	0.12	0.02	1.13	0.08
Omitting A. S. Senger, 2022		1.67	[1.32; 2.12]	0.64	0.39	0.09	0.01	1.09	0.04
Omitting N. Kurtul, 2017		1.69	[1.33; 2.13]	0.65	0.06	0.02	0.00	1.07	0.01
Omitting M. Shibutani, 2023		1.69	[1.33; 2.14]	0.65	-0.05	-0.00	0.00	1.11	0.05
Omitting Y. Iseki, 2015		1.69	[1.33; 2.14]	0.65	-0.10	-0.01	0.00	1.10	0.05
Omitting T. Amano, 2023		1.69	[1.34; 2.14]	0.65	-0.16	-0.03	0.00	1.10	0.05
Omitting X. Zhu, 2025		1.70	[1.35; 2.15]	0.64	-0.56	-0.08	0.01	1.06	0.02
Omitting K. Maeda, 2014		1.70	[1.35; 2.15]	0.64	-0.49	-0.08	0.01	1.07	0.03
Omitting R. Sato, 2021		1.72	[1.37; 2.15]	0.63	-1.24	-0.15	0.02	0.99	0.01
Omitting S. Lee, 2023		1.72	[1.36; 2.17]	0.64	-0.73	-0.15	0.02	1.07	0.04
Omitting J. Peng, 2017		1.73	[1.37; 2.19]	0.62	-0.91	-0.23	0.05	1.07	0.06
Omitting X. Wang, 2020		1.75	[1.40; 2.18]	0.62	-1.58	-0.32	0.09	0.96	0.04
Omitting Y. Zhao, 2018		1.77	[1.42; 2.20]	0.58	-1.81	-0.46	0.19	0.95	0.05
Omitting J. Li, 2023	*	1.80	[1.46; 2.22]	0.49	-2.37	-0.69	0.37	0.87	0.06
**Random effects model**		**1.69**	**[1.35; 2.11]**						

I^2 = 0% [0%; 46%]

Figure S174: Funnel plot: Association between malnutrition-related complication risk and age (cut off 65) in gastrointestinal cancer (Biological composite scores)

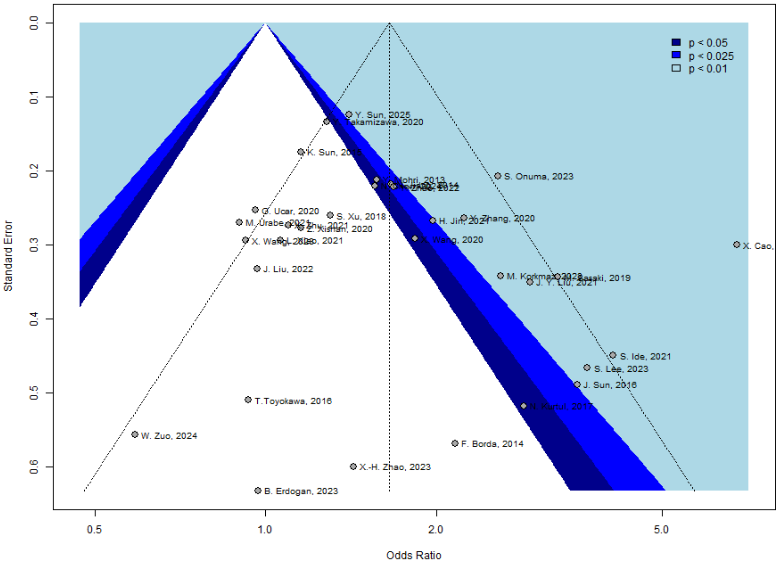

Egger test: t = 0.94, df = 30, p-value = 0.3562

Figure S174.1: Leave-one-out plot: Association between malnutrition-related complication risk and age (cut off 65) in gastrointestinal cancer (Biological composite scores)

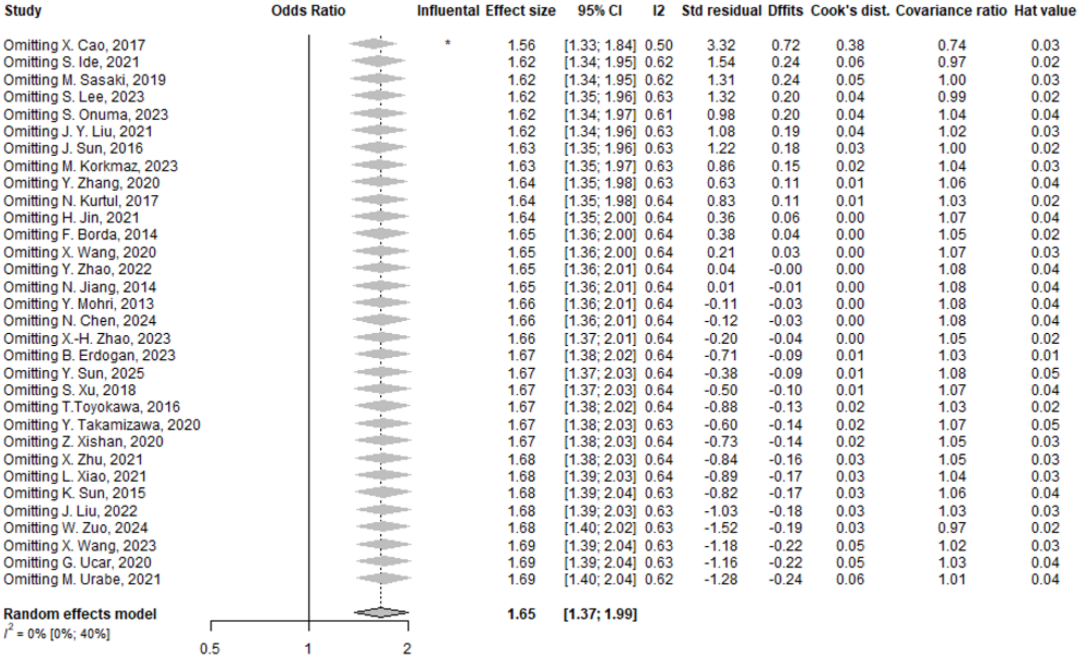

Figure S175: Funnel plot: Association between malnutrition-related complication risk and ASA score (≥3 vs. <3) in gastric cancer (Biological composite scores)

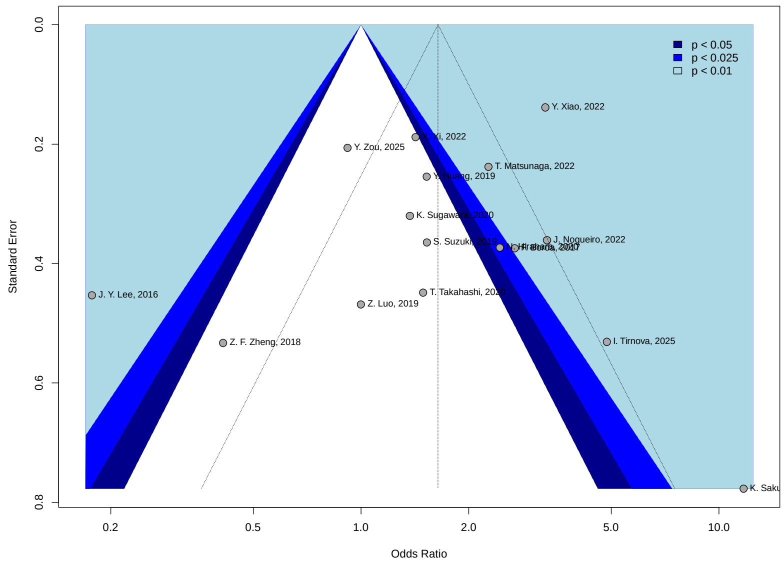

Egger test: t = 1.58, df = 14, p-value = 0.1358

Figure S175.1: Leave-one-out plot: Association between malnutrition-related complication risk and ASA score (≥3 vs. <3) in gastric cancer (Biological composite scores)

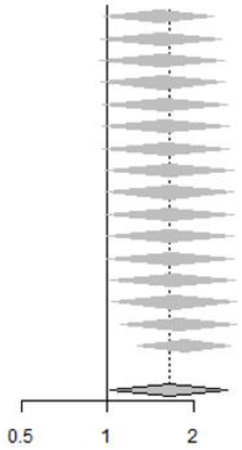

Study	Influental	Effect size	95% CI	I2	Std residual	Dffits	Cook's dist.	Covariance ratio	Hat value
Omitting K. Sakurai, 2016		1.52	[0.97; 2.37]	0.82	1.89	0.36	0.12	0.86	0.04
Omitting I. Tirnova, 2025		1.54	[0.95; 2.51]	0.82	1.17	0.28	0.08	1.03	0.05
Omitting Y. Xiao, 2022		1.55	[0.94; 2.57]	0.76	0.86	0.24	0.06	1.10	0.07
Omitting J. Nogueiro, 2022		1.57	[0.95; 2.59]	0.82	0.81	0.21	0.05	1.10	0.06
Omitting F. Borda, 2017		1.59	[0.95; 2.64]	0.83	0.56	0.14	0.02	1.13	0.06
Omitting N. Hirahara, 2020		1.60	[0.96; 2.67]	0.83	0.45	0.11	0.01	1.14	0.06
Omitting T. Matsunaga, 2022		1.60	[0.96; 2.68]	0.83	0.38	0.10	0.01	1.15	0.07
Omitting S. Suzuki, 2019		1.65	[0.99; 2.76]	0.83	-0.08	-0.02	0.00	1.16	0.06
Omitting Y. Huang, 2019		1.65	[0.98; 2.77]	0.83	-0.09	-0.03	0.00	1.16	0.07
Omitting T. Takahashi, 2020		1.65	[0.99; 2.76]	0.83	-0.10	-0.03	0.00	1.15	0.06
Omitting X. Xi, 2022		1.66	[0.99; 2.79]	0.83	-0.17	-0.05	0.00	1.16	0.07
Omitting K. Sugawara, 2020		1.66	[0.99; 2.79]	0.83	-0.21	-0.06	0.00	1.15	0.07
Omitting Z. Luo, 2019		1.69	[1.02; 2.81]	0.83	-0.53	-0.13	0.02	1.12	0.06
Omitting Y. Zou, 2025		1.72	[1.03; 2.85]	0.80	-0.71	-0.20	0.04	1.12	0.07
Omitting Z. F. Zheng, 2018		1.77	[1.11; 2.83]	0.81	-1.53	-0.35	0.11	0.96	0.05
Omitting J. Y. Lee, 2016		1.86	[1.28; 2.70]	0.75	-3.15	-0.72	0.31	0.60	0.06
**Random effects model**		**1.64**	**[1.02; 2.64]**						
I^2 = 0% [0%; 52%]									

Figure S176: Funnel plot: Association between malnutrition-related complication risk and serum CA 19-9 level in colorectal cancer (Biological composite scores)

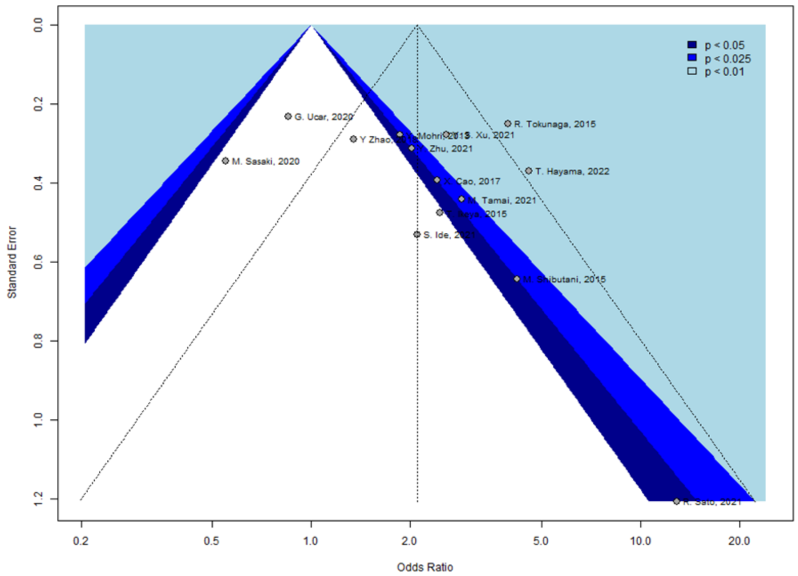

Egger test: t = 0.68, df = 12, p-value = 0.5087

Figure S176.1: Leave-one-out plot: Association between malnutrition-related complication riskand serum CA 19-9 level in colorectal cancer (Biological composite scores)

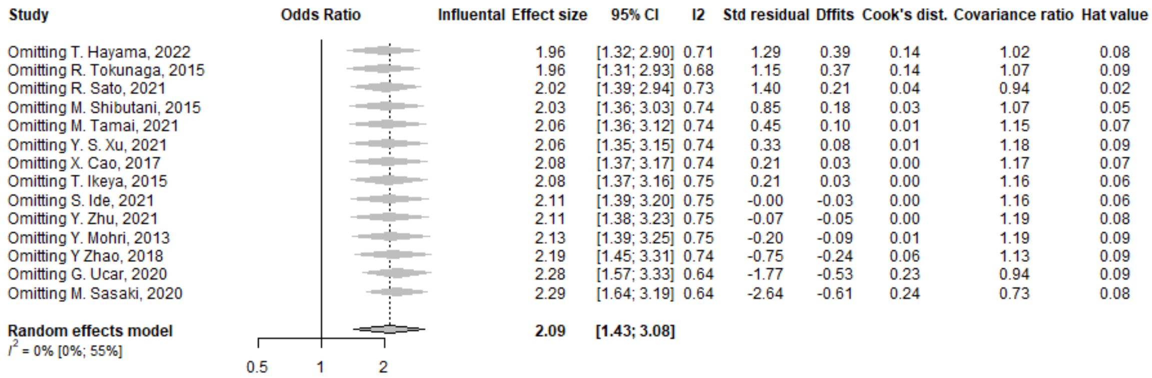

Study	Influental	Effect size	95% CI	I2	Std residual	Dffits	Cook's dist.	Covariance ratio	Hat value
Omitting T. Hayama, 2022		1.96	[1.32; 2.90]	0.71	1.29	0.39	0.14	1.02	0.08
Omitting R. Tokunaga, 2015		1.96	[1.31; 2.93]	0.68	1.15	0.37	0.14	1.07	0.09
Omitting R. Sato, 2021		2.02	[1.39; 2.94]	0.73	1.40	0.21	0.04	0.94	0.02
Omitting M. Shibutani, 2015		2.03	[1.36; 3.03]	0.74	0.85	0.18	0.03	1.07	0.05
Omitting M. Tamai, 2021		2.06	[1.36; 3.12]	0.74	0.45	0.10	0.01	1.15	0.07
Omitting Y. S. Xu, 2021		2.06	[1.35; 3.15]	0.74	0.33	0.08	0.01	1.18	0.09
Omitting X. Cao, 2017		2.08	[1.37; 3.17]	0.74	0.21	0.03	0.00	1.17	0.07
Omitting T. Ikeya, 2015		2.08	[1.37; 3.16]	0.75	0.21	0.03	0.00	1.16	0.06
Omitting S. Ide, 2021		2.11	[1.39; 3.20]	0.75	-0.00	-0.03	0.00	1.16	0.06
Omitting Y. Zhu, 2021		2.11	[1.38; 3.23]	0.75	-0.07	-0.05	0.00	1.19	0.08
Omitting Y. Mohri, 2013		2.13	[1.39; 3.25]	0.75	-0.20	-0.09	0.01	1.19	0.09
Omitting Y Zhao, 2018		2.19	[1.45; 3.31]	0.74	-0.75	-0.24	0.06	1.13	0.09
Omitting G. Ucar, 2020		2.28	[1.57; 3.33]	0.64	-1.77	-0.53	0.23	0.94	0.09
Omitting M. Sasaki, 2020		2.29	[1.64; 3.19]	0.64	-2.64	-0.61	0.24	0.73	0.08
**Random effects model**		**2.09**	**[1.43; 3.08]**						

I^2 = 0% [0%; 55%]

Figure S177: Funnel plot: Association between malnutrition-related complication risk and serum CEA level in colorectal cancer (Biological composite scores)

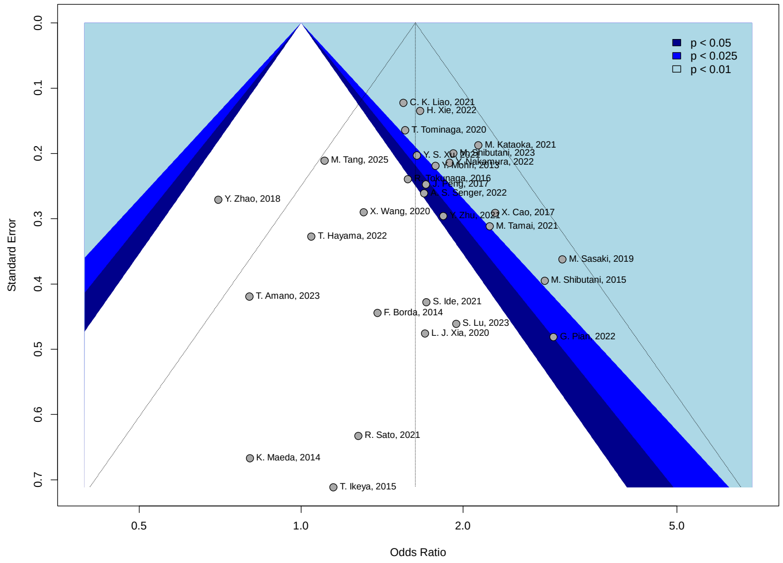

Egger test: t = -0.58, df = 27, p-value = 0.5653

Figure S177.1: Leave-one-out plot: Association between malnutrition-related complication risk and serum CEA level in colorectal cancer (Biological composite scores)

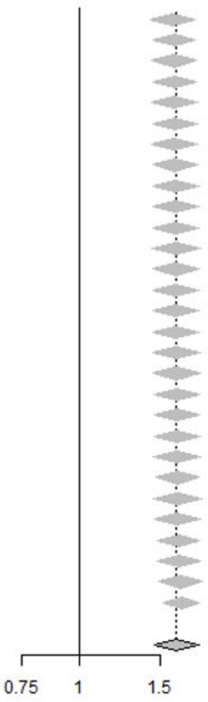

Study	Influential	Effect size	95% CI	I2	Std residual	Dffits	Cook's dist.	Covariance ratio	Hat value
Omitting M. Kataoka, 2021		1.60	[1.43; 1.79]	0.12	1.28	0.32	0.10	1.02	0.06
Omitting M. Sasaki, 2019		1.61	[1.45; 1.79]	0.10	1.70	0.23	0.05	0.90	0.02
Omitting X. Cao, 2017		1.61	[1.44; 1.81]	0.14	1.11	0.19	0.04	1.02	0.03
Omitting M. Shibutani, 2015		1.62	[1.45; 1.80]	0.13	1.36	0.17	0.03	0.96	0.02
Omitting M. Shibutani, 2023		1.62	[1.44; 1.82]	0.16	0.70	0.17	0.03	1.09	0.05
Omitting M. Tamai, 2021		1.62	[1.44; 1.81]	0.15	0.96	0.16	0.03	1.03	0.03
Omitting Y. Nakamura, 2022		1.62	[1.44; 1.82]	0.17	0.60	0.14	0.02	1.09	0.05
Omitting G. Pian, 2022		1.62	[1.45; 1.81]	0.14	1.20	0.13	0.02	0.98	0.01
Omitting Y. Mohri, 2013		1.62	[1.44; 1.83]	0.18	0.34	0.08	0.01	1.11	0.05
Omitting Y. Zhu, 2021		1.63	[1.45; 1.83]	0.18	0.37	0.07	0.00	1.09	0.03
Omitting S. Lu, 2023		1.63	[1.45; 1.83]	0.18	0.37	0.04	0.00	1.07	0.01
Omitting H. Xie, 2022		1.63	[1.44; 1.84]	0.18	0.11	0.03	0.00	1.16	0.09
Omitting J. Peng, 2017		1.63	[1.45; 1.83]	0.18	0.16	0.03	0.00	1.10	0.04
Omitting A. S. Senger, 2022		1.63	[1.45; 1.83]	0.18	0.13	0.03	0.00	1.10	0.04
Omitting S. Ide, 2021		1.63	[1.45; 1.83]	0.18	0.11	0.01	0.00	1.08	0.01
Omitting L. J. Xia, 2020		1.63	[1.45; 1.83]	0.18	0.08	0.01	0.00	1.08	0.01
Omitting Y. S. Xu, 2021		1.63	[1.45; 1.83]	0.18	0.03	0.01	0.00	1.12	0.05
Omitting R. Tokunaga, 2016		1.63	[1.45; 1.84]	0.18	-0.12	-0.02	0.00	1.11	0.04
Omitting R. Sato, 2021		1.63	[1.46; 1.83]	0.18	-0.38	-0.03	0.00	1.06	0.01
Omitting T. Ikeya, 2015		1.63	[1.46; 1.83]	0.18	-0.49	-0.04	0.00	1.05	0.01
Omitting F. Borda, 2014		1.64	[1.46; 1.83]	0.18	-0.35	-0.04	0.00	1.07	0.01
Omitting T. Tominaga, 2020		1.64	[1.45; 1.84]	0.18	-0.21	-0.06	0.00	1.14	0.07
Omitting K. Maeda, 2014		1.64	[1.47; 1.83]	0.15	-1.05	-0.08	0.01	1.00	0.01
Omitting C. K. Liao, 2021		1.64	[1.45; 1.85]	0.18	-0.30	-0.09	0.01	1.17	0.10
Omitting X. Wang, 2020		1.64	[1.46; 1.84]	0.17	-0.71	-0.13	0.02	1.06	0.03
Omitting T. Amano, 2023		1.65	[1.48; 1.83]	0.10	-1.67	-0.19	0.04	0.91	0.02
Omitting T. Hayama, 2022		1.65	[1.48; 1.84]	0.13	-1.31	-0.20	0.04	0.98	0.02
Omitting M. Tang, 2025		1.66	[1.50; 1.85]	0.08	-1.74	-0.40	0.14	0.93	0.05
Omitting Y. Zhao, 2018		1.67	[1.53; 1.83]	0.00	-3.16	-0.50	0.21	0.75	0.03
**Random effects model**		**1.63**	**[1.46; 1.82]**						

I^2 = 0% [0%; 41%]

Figure S178: Funnel plot: Association between malnutrition-related complication risk and serum CEA level in gastric cancer (Biological composite scores)

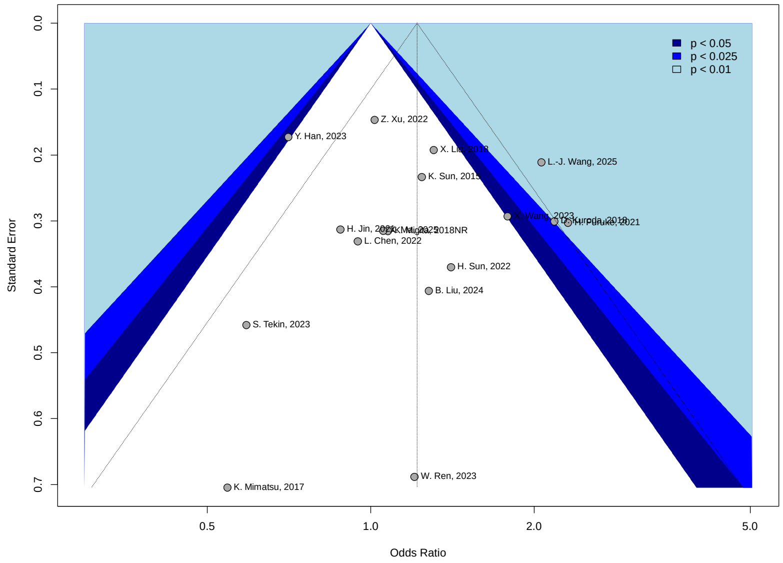

Egger test: t = -1.20, df = 15, p-value = 0.2479

Figure S178.1: Leave-one-out plot: Association between malnutrition-related complication risk and serum CEA level in gastric cancer (Biological composite scores)

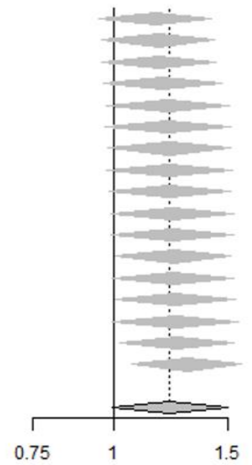

Study	Odds Ratio	Influental	Effect size	95% CI	I2	Std residual	Dffits	Cook's dist.	Covariance ratio	Hat value
Omitting L.-J. Wang, 2025			1.16	[0.95; 1.41]	0.42	1.79	0.57	0.27	0.95	0.08
Omitting H. Furuke, 2021			1.17	[0.96; 1.43]	0.47	1.75	0.43	0.17	0.93	0.06
Omitting D. Kuroda, 2018			1.17	[0.96; 1.44]	0.49	1.57	0.39	0.14	0.97	0.06
Omitting X. Wang, 2023			1.19	[0.96; 1.47]	0.52	1.01	0.26	0.07	1.06	0.06
Omitting H. Sun, 2022			1.21	[0.97; 1.50]	0.55	0.32	0.07	0.01	1.12	0.05
Omitting X. Liu, 2018			1.21	[0.97; 1.51]	0.55	0.22	0.07	0.00	1.17	0.09
Omitting B. Liu, 2024			1.21	[0.98; 1.51]	0.55	0.10	0.02	0.00	1.12	0.04
Omitting K. Sun, 2015			1.22	[0.97; 1.52]	0.55	0.06	0.02	0.00	1.16	0.08
Omitting W. Ren, 2023			1.22	[0.98; 1.51]	0.55	-0.02	-0.00	0.00	1.09	0.02
Omitting K. Migita, 2018NR			1.23	[0.99; 1.53]	0.55	-0.30	-0.08	0.01	1.13	0.06
Omitting X. Ma, 2025			1.23	[0.99; 1.53]	0.55	-0.35	-0.09	0.01	1.13	0.06
Omitting K. Mimatsu, 2017			1.23	[1.00; 1.52]	0.54	-1.08	-0.14	0.02	1.01	0.02
Omitting L. Chen, 2022			1.23	[0.99; 1.53]	0.55	-0.60	-0.14	0.02	1.10	0.05
Omitting H. Jin, 2021			1.24	[1.00; 1.54]	0.54	-0.81	-0.20	0.04	1.08	0.06
Omitting Z. Xu, 2022			1.24	[1.00; 1.55]	0.53	-0.62	-0.21	0.05	1.16	0.10
Omitting S. Tekin, 2023			1.25	[1.02; 1.53]	0.52	-1.42	-0.25	0.06	0.97	0.03
Omitting Y. Han, 2023			1.29	[1.07; 1.57]	0.35	-2.15	-0.74	0.41	0.90	0.10
**Random effects model**			**1.22**	**[0.99; 1.49]**						

I^2 = 0% [0%; 51%]

Figure S179: Funnel plot: Association between malnutrition-related complication risk and Child-Pugh class (B, C vs. A) in hepatocellular carcinoma (Biological composite scores)

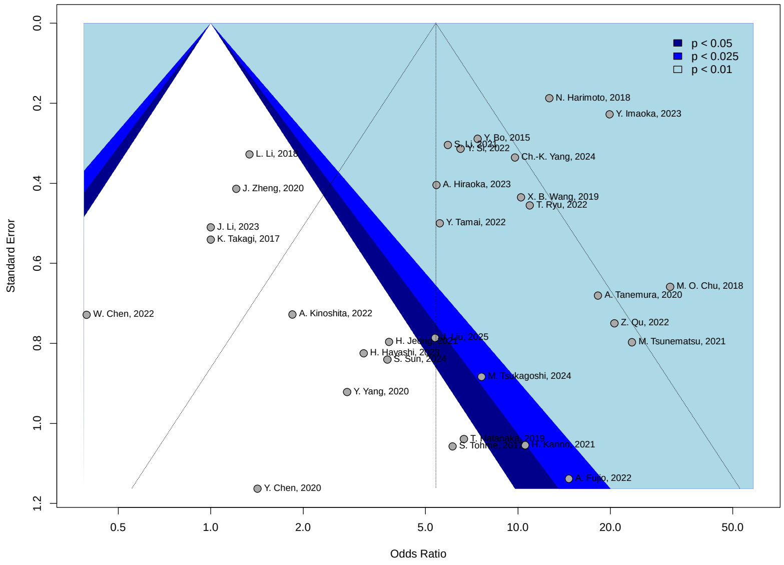

Egger test: t = -0.50, df = 29, p-value = 0.6215

Figure S179.1: Leave-one-out plot: Association between malnutrition-related complication risk and Child-Pugh class (B, C vs. A) in hepatocellular carcinoma (Biological composite scores)

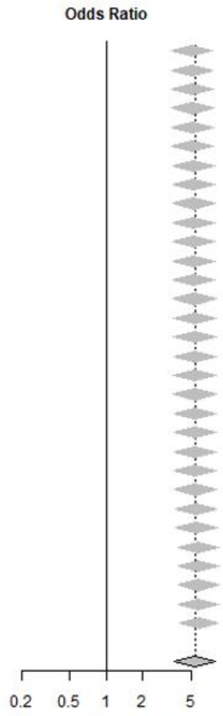

Study	Influental	Effect size	95% CI	I2	Std residual	Dffits	Cook's dist.	Covariance ratio	Hat value
Omitting Y. Imaoka, 2023		5.07	[3.42; 7.53]	0.75	1.53	0.34	0.11	1.00	0.05
Omitting M. O. Chu, 2018		5.12	[3.47; 7.54]	0.79	1.68	0.29	0.08	0.97	0.03
Omitting N. Harimoto, 2018		5.18	[3.46; 7.76]	0.77	0.98	0.22	0.05	1.05	0.05
Omitting M. Tsunematsu, 2021		5.19	[3.50; 7.71]	0.79	1.27	0.21	0.04	1.01	0.03
Omitting Z. Qu, 2022		5.20	[3.50; 7.73]	0.79	1.19	0.20	0.04	1.01	0.03
Omitting A. Tanemura, 2020		5.20	[3.49; 7.75]	0.79	1.12	0.20	0.04	1.02	0.03
Omitting T. Ryu, 2022		5.25	[3.50; 7.87]	0.79	0.73	0.15	0.02	1.06	0.04
Omitting Ch.-K. Yang, 2024		5.26	[3.50; 7.90]	0.79	0.64	0.14	0.02	1.07	0.04
Omitting X. B. Wang, 2019		5.26	[3.51; 7.90]	0.79	0.66	0.14	0.02	1.06	0.04
Omitting A. Fujio, 2022		5.30	[3.55; 7.92]	0.79	0.70	0.10	0.01	1.04	0.02
Omitting Y. Bo, 2015		5.32	[3.53; 8.01]	0.80	0.34	0.08	0.01	1.08	0.04
Omitting H. Kanno, 2021		5.33	[3.56; 7.97]	0.79	0.49	0.07	0.01	1.05	0.02
Omitting M. Tsukagoshi, 2024		5.35	[3.57; 8.03]	0.80	0.28	0.05	0.00	1.06	0.02
Omitting Y. Si, 2022		5.35	[3.55; 8.07]	0.80	0.20	0.05	0.00	1.08	0.04
Omitting T. Hatanaka, 2019		5.38	[3.59; 8.06]	0.80	0.16	0.03	0.00	1.06	0.02
Omitting S. Li, 2021		5.38	[3.57; 8.10]	0.79	0.10	0.02	0.00	1.08	0.04
Omitting S. Tohme, 2017		5.39	[3.59; 8.07]	0.80	0.09	0.02	0.00	1.06	0.02
Omitting Y. Tamai, 2022		5.39	[3.58; 8.12]	0.79	0.03	0.01	0.00	1.07	0.04
Omitting A. Hiraoka, 2023		5.40	[3.58; 8.13]	0.79	0.01	0.00	0.00	1.08	0.04
Omitting J. Liu, 2025		5.40	[3.60; 8.11]	0.80	-0.00	0.00	0.00	1.06	0.03
Omitting S. Sun, 2024		5.45	[3.63; 8.18]	0.79	-0.30	-0.05	0.00	1.06	0.03
Omitting H. Jeong, 2021		5.45	[3.63; 8.18]	0.79	-0.30	-0.05	0.00	1.06	0.03
Omitting H. Hayashi, 2023		5.48	[3.65; 8.21]	0.79	-0.45	-0.07	0.01	1.06	0.03
Omitting Y. Yang, 2020		5.49	[3.66; 8.22]	0.79	-0.53	-0.08	0.01	1.05	0.02
Omitting Y. Chen, 2020		5.53	[3.71; 8.24]	0.79	-0.93	-0.12	0.02	1.02	0.02
Omitting A. Kinoshita, 2022		5.58	[3.74; 8.33]	0.79	-0.96	-0.17	0.03	1.03	0.03
Omitting K. Takagi, 2017		5.76	[3.91; 8.49]	0.77	-1.74	-0.34	0.11	0.97	0.04
Omitting J. Zheng, 2020		5.76	[3.90; 8.52]	0.77	-1.64	-0.34	0.11	0.99	0.04
Omitting L. Li, 2018		5.77	[3.89; 8.54]	0.75	-1.59	-0.35	0.11	0.99	0.04
Omitting J. Li, 2023		5.77	[3.92; 8.50]	0.77	-1.77	-0.36	0.12	0.97	0.04
Omitting W. Chen, 2022		5.85	[4.05; 8.45]	0.77	-2.51	-0.44	0.17	0.87	0.03
**Random effects model**		**5.40**	**[3.65; 8.01]**						

I^2 = 0% [0%; 40%]

Figure S180: Funnel plot: Association between malnutrition-related complication risk and cardiovascular disease in gastric cancer (Biological composite scores)

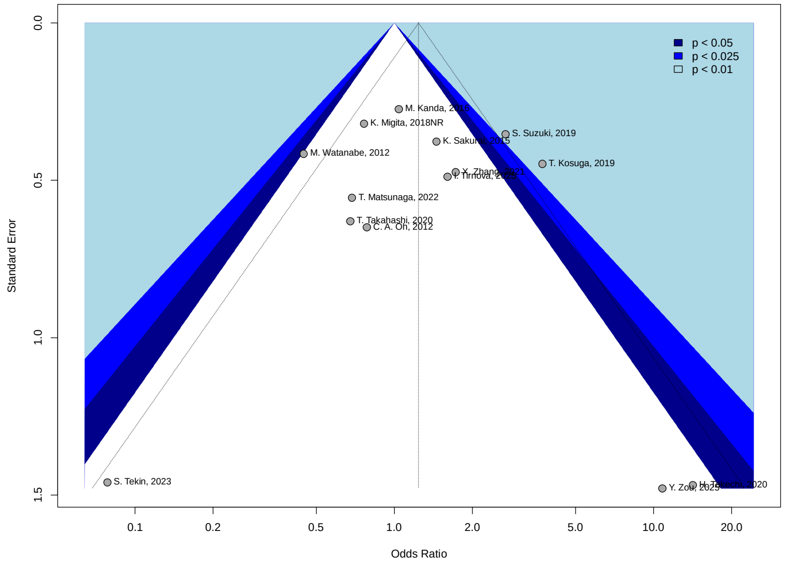

Egger test: t = -0.86, df = 12, p-value = 0.4060

Figure S180.1: Leave-one-out plot: Association between malnutrition-related complication risk and cardiovascular disease in gastric cancer (Biological composite scores)

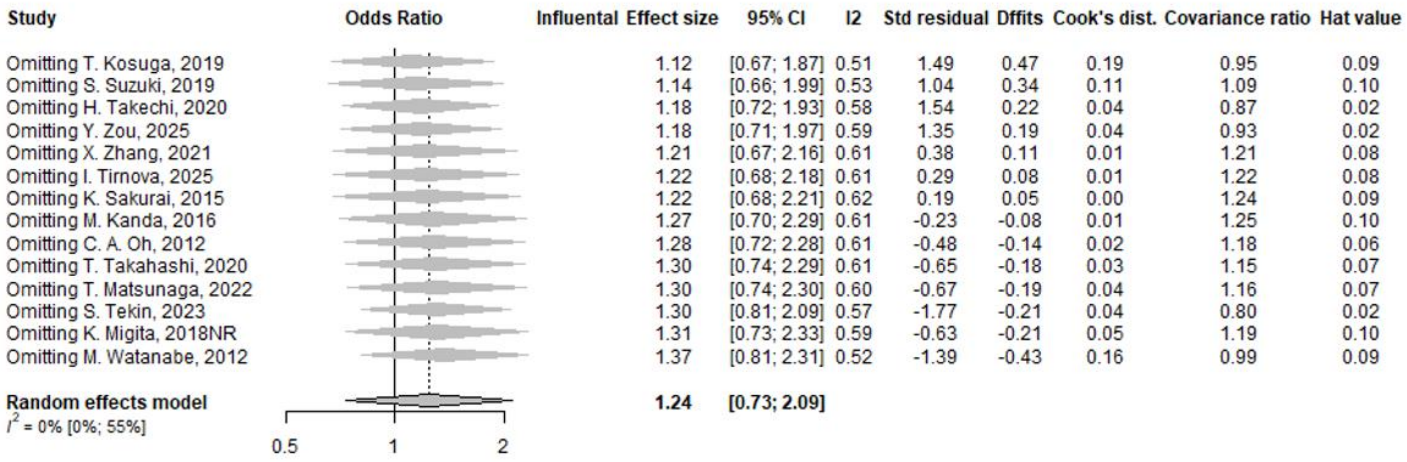

Study	Odds Ratio	Influential	Effect size	95% CI	I2	Std residual	Dffits	Cook's dist.	Covariance ratio	Hat value
Omitting T. Kosuga, 2019			1.12	[0.67; 1.87]	0.51	1.49	0.47	0.19	0.95	0.09
Omitting S. Suzuki, 2019			1.14	[0.66; 1.99]	0.53	1.04	0.34	0.11	1.09	0.10
Omitting H. Takechi, 2020			1.18	[0.72; 1.93]	0.58	1.54	0.22	0.04	0.87	0.02
Omitting Y. Zou, 2025			1.18	[0.71; 1.97]	0.59	1.35	0.19	0.04	0.93	0.02
Omitting X. Zhang, 2021			1.21	[0.67; 2.16]	0.61	0.38	0.11	0.01	1.21	0.08
Omitting I. Tirnova, 2025			1.22	[0.68; 2.18]	0.61	0.29	0.08	0.01	1.22	0.08
Omitting K. Sakurai, 2015			1.22	[0.68; 2.21]	0.62	0.19	0.05	0.00	1.24	0.09
Omitting M. Kanda, 2016			1.27	[0.70; 2.29]	0.61	-0.23	-0.08	0.01	1.25	0.10
Omitting C. A. Oh, 2012			1.28	[0.72; 2.28]	0.61	-0.48	-0.14	0.02	1.18	0.06
Omitting T. Takahashi, 2020			1.30	[0.74; 2.29]	0.61	-0.65	-0.18	0.03	1.15	0.07
Omitting T. Matsunaga, 2022			1.30	[0.74; 2.30]	0.60	-0.67	-0.19	0.04	1.16	0.07
Omitting S. Tekin, 2023			1.30	[0.81; 2.09]	0.57	-1.77	-0.21	0.04	0.80	0.02
Omitting K. Migita, 2018NR			1.31	[0.73; 2.33]	0.59	-0.63	-0.21	0.05	1.19	0.10
Omitting M. Watanabe, 2012			1.37	[0.81; 2.31]	0.52	-1.39	-0.43	0.16	0.99	0.09
**Random effects model**			**1.24**	**[0.73; 2.09]**						

I^2 = 0% [0%; 55%]

Odds Ratio axis: 0.5, 1, 2

Figure S181: Funnel plot: Association between malnutrition-related complication risk and diabetes mellitus in gastrointestinal cancer (Biological composite scores)

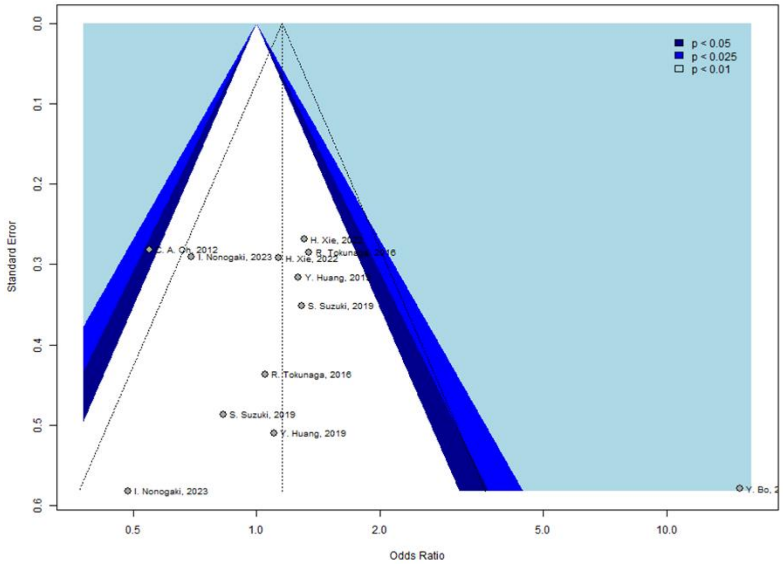

Egger test: t = 1.17, df = 10, p-value = 0.2699

Figure S181.1: Leave-one-out plot: Association between malnutrition-related complication risk and diabetes mellitus in gastrointestinal cancer (Biological composite scores)

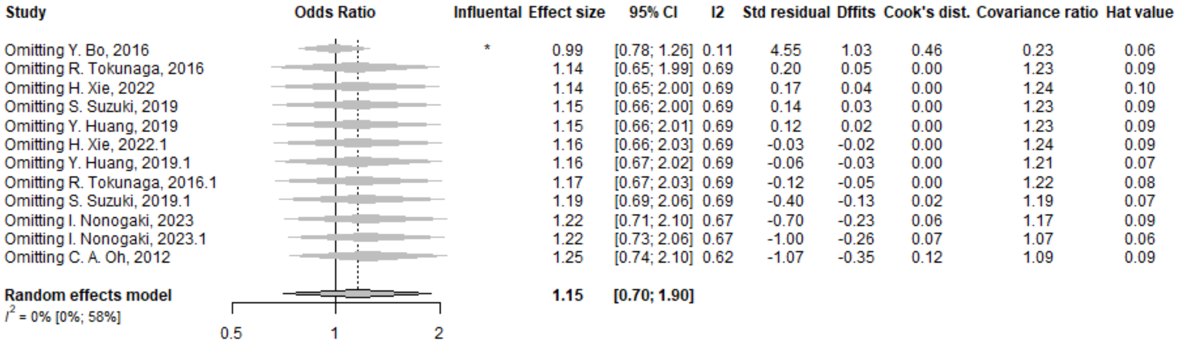

Study	Influental	Effect size	95% CI	I2	Std residual	Dffits	Cook's dist.	Covariance ratio	Hat value
Omitting Y. Bo, 2016	*	0.99	[0.78; 1.26]	0.11	4.55	1.03	0.46	0.23	0.06
Omitting R. Tokunaga, 2016		1.14	[0.65; 1.99]	0.69	0.20	0.05	0.00	1.23	0.09
Omitting H. Xie, 2022		1.14	[0.65; 2.00]	0.69	0.17	0.04	0.00	1.24	0.10
Omitting S. Suzuki, 2019		1.15	[0.66; 2.00]	0.69	0.14	0.03	0.00	1.23	0.09
Omitting Y. Huang, 2019		1.15	[0.66; 2.01]	0.69	0.12	0.02	0.00	1.23	0.09
Omitting H. Xie, 2022.1		1.16	[0.66; 2.03]	0.69	-0.03	-0.02	0.00	1.24	0.09
Omitting Y. Huang, 2019.1		1.16	[0.67; 2.02]	0.69	-0.06	-0.03	0.00	1.21	0.07
Omitting R. Tokunaga, 2016.1		1.17	[0.67; 2.03]	0.69	-0.12	-0.05	0.00	1.22	0.08
Omitting S. Suzuki, 2019.1		1.19	[0.69; 2.06]	0.69	-0.40	-0.13	0.02	1.19	0.07
Omitting I. Nonogaki, 2023		1.22	[0.71; 2.10]	0.67	-0.70	-0.23	0.06	1.17	0.09
Omitting I. Nonogaki, 2023.1		1.22	[0.73; 2.06]	0.67	-1.00	-0.26	0.07	1.07	0.06
Omitting C. A. Oh, 2012		1.25	[0.74; 2.10]	0.62	-1.07	-0.35	0.12	1.09	0.09
**Random effects model**		**1.15**	**[0.70; 1.90]**						

I^2 = 0% [0%; 58%]

Figure S182: Funnel plot: Association between malnutrition-related complication risk and hypertension in gastrointestinal cancer (Biological composite scores)

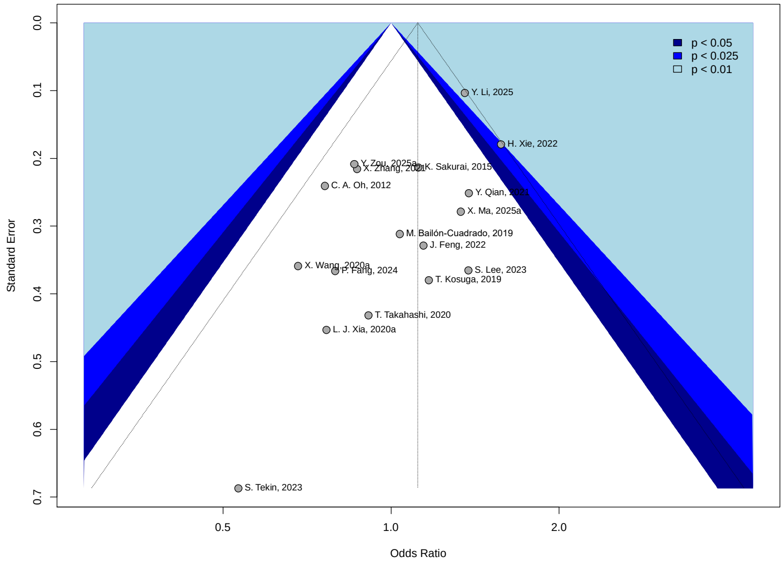

Egger test: t = -2.06, df = 15, p-value = 0.0568

Figure S182.1: Leave-one-out plot: Association between malnutrition-related complication risk and hypertension in gastrointestinal cancer (Biological composite scores)

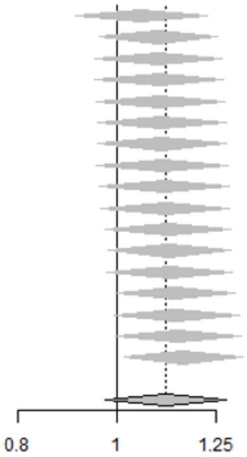

Study	Influential	Effect size	95% CI	I2	Std residual	Dffits	Cook's dist.	Covariance ratio	Hat value
Omitting Y. Li, 2025	*	1.06	[0.91; 1.23]	0.00	1.99	1.09	0.72	1.17	0.24
Omitting H. Xie, 2022		1.10	[0.96; 1.25]	0.02	1.88	0.29	0.07	0.92	0.11
Omitting Y. Qian, 2021		1.10	[0.95; 1.27]	0.18	0.82	0.24	0.06	1.09	0.06
Omitting X. Ma, 2025a		1.10	[0.95; 1.27]	0.19	0.64	0.22	0.05	1.10	0.05
Omitting S. Lee, 2023		1.10	[0.96; 1.27]	0.19	0.57	0.18	0.03	1.09	0.03
Omitting T. Kosuga, 2019		1.11	[0.96; 1.28]	0.20	0.14	0.13	0.02	1.11	0.03
Omitting J. Feng, 2022		1.11	[0.96; 1.28]	0.20	0.09	0.13	0.02	1.12	0.04
Omitting K. Sakurai, 2015		1.11	[0.95; 1.29]	0.20	0.04	0.12	0.02	1.18	0.08
Omitting M. Bailón-Cuadrado, 2019		1.11	[0.96; 1.29]	0.20	-0.21	0.06	0.00	1.12	0.04
Omitting T. Takahashi, 2020		1.12	[0.97; 1.29]	0.19	-0.45	0.02	0.00	1.09	0.02
Omitting L. J. Xia, 2020a		1.12	[0.97; 1.29]	0.17	-0.82	-0.08	0.01	1.05	0.02
Omitting S. Tekin, 2023		1.12	[0.98; 1.29]	0.15	-1.08	-0.12	0.01	1.00	0.01
Omitting P. Fang, 2024		1.13	[0.98; 1.30]	0.16	-0.92	-0.14	0.02	1.04	0.03
Omitting X. Wang, 2020a		1.14	[1.00; 1.31]	0.10	-1.39	-0.35	0.12	0.96	0.03
Omitting X. Zhang, 2021		1.14	[0.99; 1.32]	0.12	-1.15	-0.37	0.14	1.06	0.08
Omitting Y. Zou, 2025a		1.15	[1.00; 1.32]	0.11	-1.26	-0.45	0.19	1.04	0.08
Omitting C. A. Oh, 2012		1.16	[1.02; 1.33]	0.05	-1.67	-0.64	0.38	0.93	0.06
**Random effects model**		**1.12**	**[0.97; 1.28]**						

I^2 = 0% [0%; 51%]

Figure S183: Funnel plot: Association between malnutrition-related complication risk and N stage (N≥1 vs. N0) in esophageal squamous cell carcinoma (Biological composite scores)

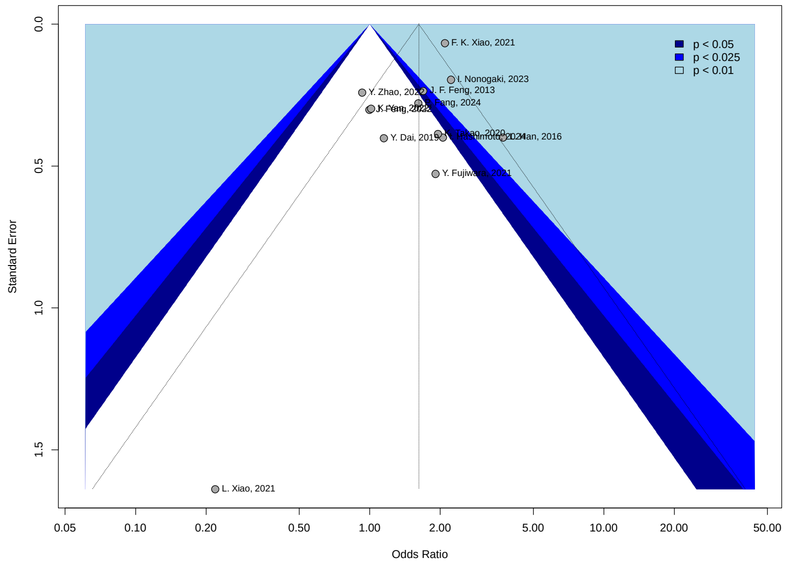

Egger test: t = -1.87, df = 11, p-value = 0.0881

Figure S183.1: Leave-one-out plot: Association between malnutrition-related complication risk and N stage (N≥1 vs. N0) in esophageal squamous cell carcinoma (Biological composite scores)

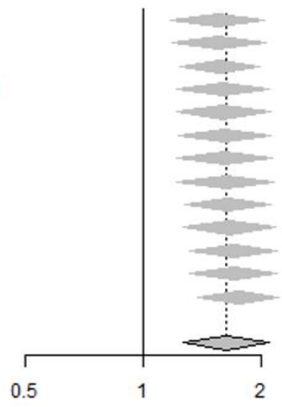

Study	Influental	Effect size	95% CI	I2	Std residual	Dffits	Cook's dist.	Covariance ratio	Hat value
Omitting F. K. Xiao, 2021		1.54	[1.16; 2.04]	0.45	0.98	0.43	0.19	1.20	0.16
Omitting I. Nonogaki, 2023		1.55	[1.18; 2.04]	0.58	0.99	0.36	0.13	1.13	0.12
Omitting L. Han, 2016		1.57	[1.24; 1.98]	0.55	1.84	0.31	0.09	0.83	0.06
Omitting I. Hashimoto, 2024		1.59	[1.21; 2.09]	0.60	0.50	0.15	0.02	1.15	0.06
Omitting K. Takao, 2020		1.60	[1.21; 2.10]	0.60	0.40	0.13	0.02	1.17	0.06
Omitting Y. Fujiwara, 2021		1.60	[1.22; 2.11]	0.60	0.28	0.08	0.01	1.15	0.04
Omitting J. F. Feng, 2013		1.61	[1.21; 2.13]	0.59	0.13	0.07	0.01	1.24	0.10
Omitting P. Fang, 2024		1.62	[1.22; 2.14]	0.59	-0.00	0.03	0.00	1.22	0.09
Omitting L. Xiao, 2021		1.64	[1.28; 2.10]	0.57	-1.21	-0.10	0.01	0.96	0.00
Omitting Y. Dai, 2019		1.65	[1.26; 2.16]	0.57	-0.71	-0.16	0.03	1.12	0.06
Omitting K. Yan, 2022		1.69	[1.30; 2.19]	0.52	-1.23	-0.38	0.14	1.03	0.08
Omitting J. Feng, 2022		1.69	[1.31; 2.19]	0.52	-1.27	-0.39	0.14	1.02	0.08
Omitting Y. Zhao, 2022		1.74	[1.37; 2.21]	0.40	-1.82	-0.70	0.39	0.88	0.10
**Random effects model**		**1.62**	**[1.26; 2.09]**						

I^2 = 0% [0%; 57%]

Figure S184: Funnel plot: Association between malnutrition-related complication risk and N stage (N≥1 vs. N0) in gastric cancer (Biological composite scores)

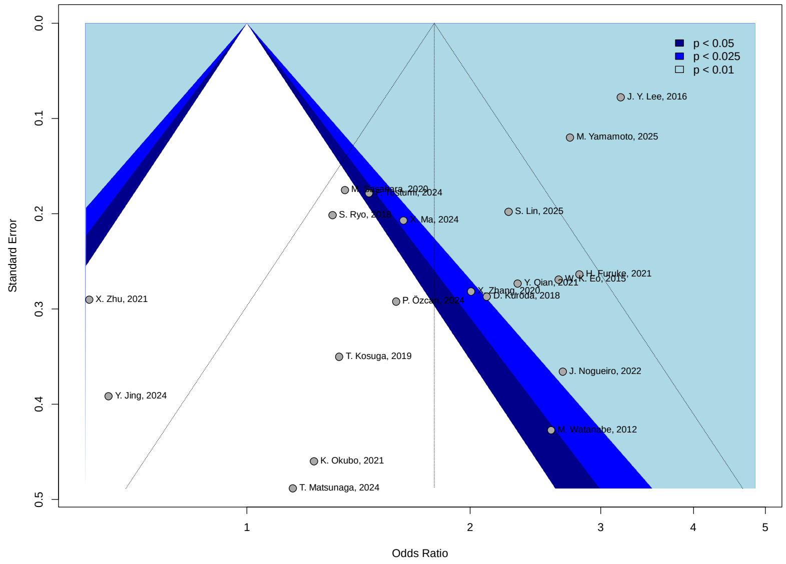

Egger test: t = -2.32, df = 18, p-value = 0.0320

Figure S184.1: Leave-one-out plot: Association between malnutrition-related complication risk and N stage (N≥1 vs. N0) in gastric cancer (Biological composite scores)

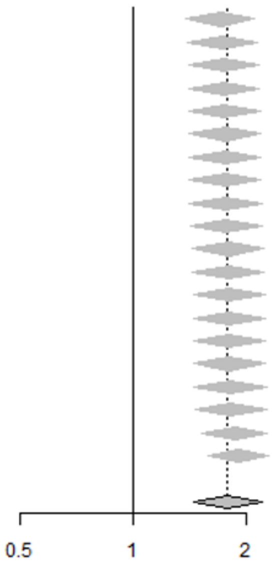

Study	Influential	Effect size	95% CI	I2	Std residual	Dffits	Cook's dist.	Covariance ratio	Hat value
Omitting J. Y. Lee, 2016		1.71	[1.39; 2.11]	0.65	1.76	0.48	0.19	0.97	0.08
Omitting M. Yamamoto, 2025		1.73	[1.39; 2.16]	0.78	1.16	0.32	0.10	1.06	0.07
Omitting H. Furuke, 2021		1.75	[1.41; 2.17]	0.79	1.04	0.24	0.06	1.05	0.05
Omitting W. K. Eo, 2015		1.75	[1.41; 2.18]	0.79	0.88	0.21	0.04	1.07	0.05
Omitting J. Nogueiro, 2022		1.76	[1.41; 2.19]	0.79	0.79	0.17	0.03	1.06	0.04
Omitting S. Lin, 2025		1.76	[1.41; 2.20]	0.79	0.57	0.16	0.03	1.11	0.06
Omitting Y. Qian, 2021		1.76	[1.41; 2.20]	0.79	0.58	0.15	0.02	1.09	0.05
Omitting M. Watanabe, 2012		1.76	[1.42; 2.20]	0.79	0.66	0.13	0.02	1.07	0.03
Omitting D. Kuroda, 2018		1.77	[1.42; 2.21]	0.79	0.36	0.10	0.01	1.10	0.05
Omitting X. Zhang, 2020		1.77	[1.42; 2.22]	0.79	0.25	0.08	0.01	1.11	0.05
Omitting X. Ma, 2024		1.80	[1.43; 2.25]	0.78	-0.23	-0.04	0.00	1.12	0.06
Omitting P. Özcan, 2024		1.80	[1.44; 2.24]	0.79	-0.25	-0.04	0.00	1.11	0.05
Omitting K. Okubo, 2021		1.81	[1.45; 2.25]	0.79	-0.64	-0.10	0.01	1.07	0.03
Omitting T. Kosuga, 2019		1.81	[1.45; 2.26]	0.78	-0.59	-0.11	0.01	1.08	0.04
Omitting T. Matsunaga, 2024		1.81	[1.46; 2.25]	0.78	-0.73	-0.11	0.01	1.06	0.03
Omitting F. Tustumi, 2024		1.81	[1.45; 2.26]	0.77	-0.50	-0.11	0.01	1.11	0.06
Omitting M. Sasahara, 2020		1.82	[1.46; 2.27]	0.77	-0.70	-0.17	0.03	1.10	0.06
Omitting S. Ryo, 2018		1.82	[1.46; 2.28]	0.77	-0.78	-0.19	0.04	1.09	0.06
Omitting Y. Jing, 2024		1.87	[1.53; 2.28]	0.76	-2.06	-0.45	0.18	0.88	0.04
Omitting X. Zhu, 2021	*	1.91	[1.59; 2.30]	0.73	-2.75	-0.75	0.43	0.76	0.05
**Random effects model**		**1.79**	**[1.45; 2.21]**						

I^2 = 0% [0%; 48%]

Figure S185: Funnel plot: Association between malnutrition-related complication risk and neoadjuvant chemo or radiotherapy in upper gastrointestinal cancer (Biological composite scores)

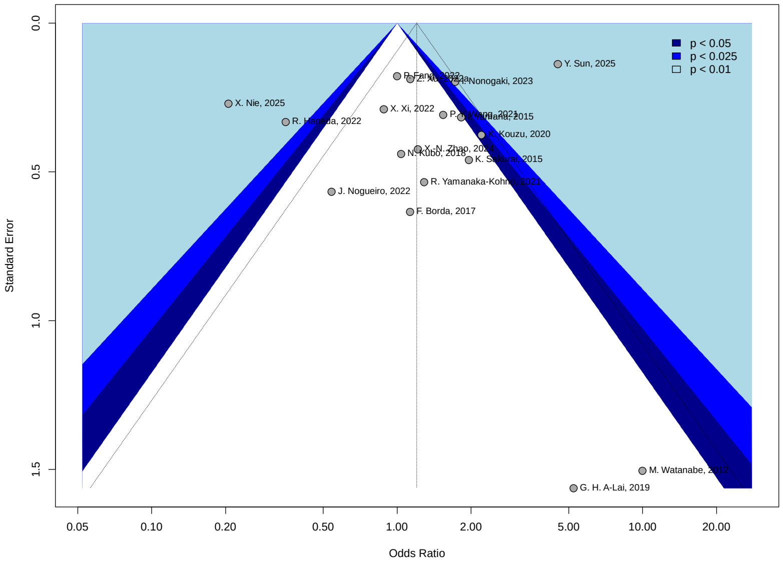

Egger test: t = -0.24, df = 16, p-value = 0.8115

Figure S185.1: Leave-one-out plot: Association between malnutrition-related complication risk and neoadjuvant chemo or radiotherapy in upper gastrointestinal cancer (Biological composite scores)

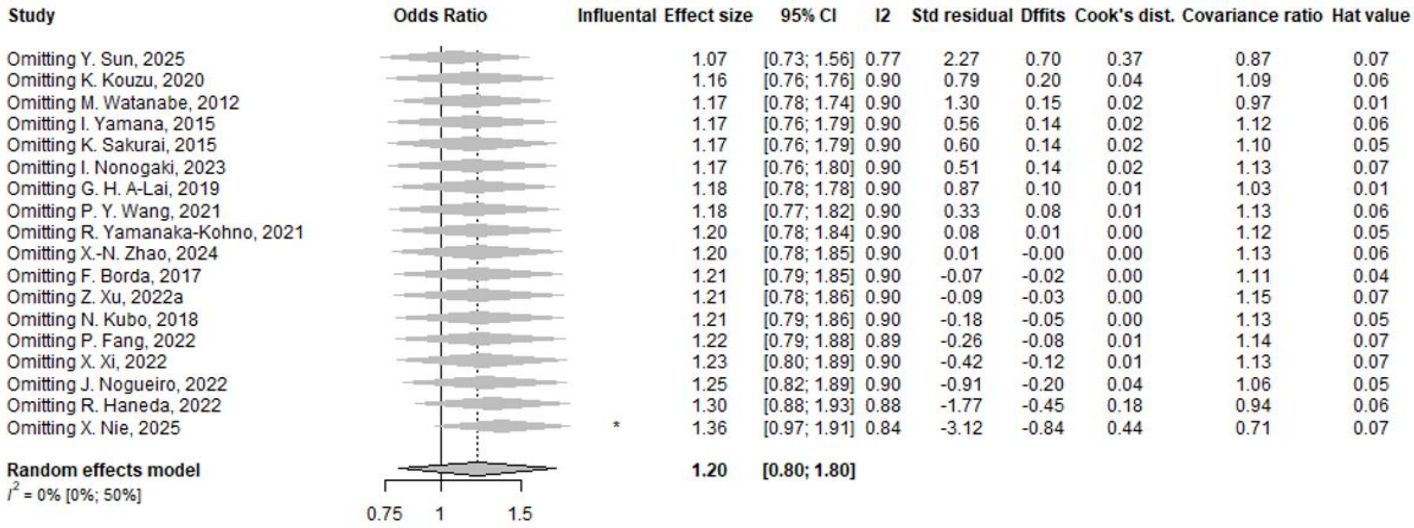

Figure S186: Funnel plot: Association between cachexia and sex in resectable gastrointestinal cancer

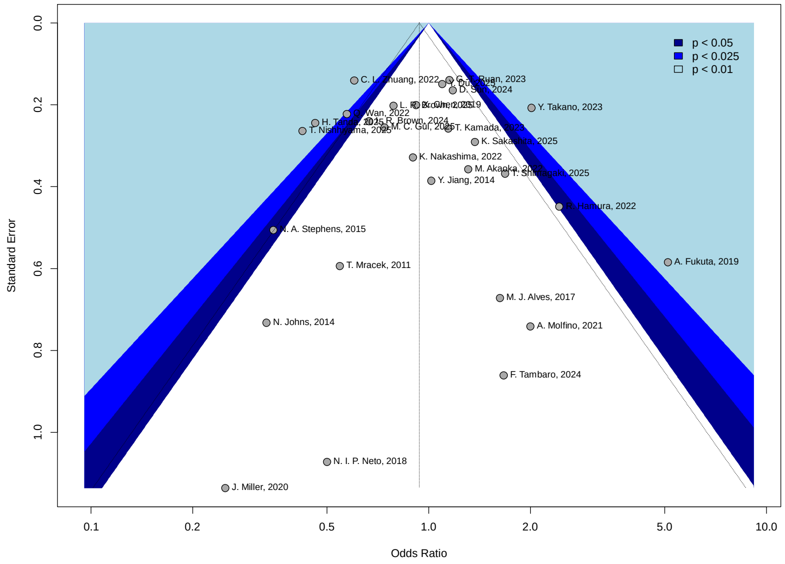

Egger test: t = -0.12, df = 26, p-value = 0.9048

Figure S186.1: Leave-one-out plot: Association between cachexia and sex in resectable gastrointestinal cancer

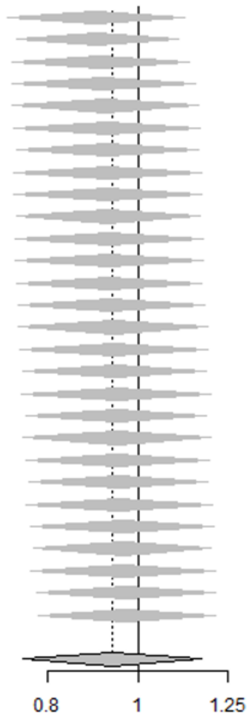

Study	Influental	Effect size	95% CI	I2	Std residual	Dffits	Cook's dist.	Covariance ratio	Hat value
Omitting Y. Takano, 2023		0.90	[0.72; 1.12]	0.62	1.69	0.40	0.15	0.96	0.05
Omitting A. Fukuta, 2019		0.90	[0.74; 1.10]	0.65	2.47	0.36	0.12	0.81	0.02
Omitting R. Hamura, 2022		0.91	[0.73; 1.13]	0.67	1.57	0.28	0.07	0.96	0.03
Omitting T. Shimagaki, 2025		0.92	[0.73; 1.15]	0.68	1.04	0.20	0.04	1.03	0.04
Omitting K. Sakashita, 2025		0.92	[0.73; 1.16]	0.68	0.72	0.15	0.02	1.07	0.04
Omitting M. Akaoka, 2022		0.93	[0.74; 1.17]	0.69	0.59	0.11	0.01	1.07	0.04
Omitting A. Molfino, 2021		0.93	[0.74; 1.16]	0.69	0.88	0.11	0.01	1.03	0.02
Omitting D. Sun, 2024		0.93	[0.73; 1.17]	0.68	0.48	0.11	0.01	1.10	0.05
Omitting G.-T. Ruan, 2023		0.93	[0.73; 1.17]	0.68	0.44	0.10	0.01	1.10	0.05
Omitting M. J. Alves, 2017		0.93	[0.74; 1.17]	0.69	0.68	0.09	0.01	1.05	0.02
Omitting T. Kamada, 2023		0.93	[0.74; 1.17]	0.69	0.38	0.08	0.01	1.09	0.04
Omitting Y. Du, 2025		0.93	[0.74; 1.18]	0.68	0.33	0.07	0.01	1.10	0.05
Omitting F. Tambaro, 2024		0.93	[0.74; 1.17]	0.69	0.59	0.06	0.00	1.05	0.01
Omitting Y. Jiang, 2014		0.94	[0.74; 1.18]	0.69	0.14	0.02	0.00	1.09	0.03
Omitting X. Chen, 2019		0.94	[0.74; 1.19]	0.69	-0.05	-0.02	0.00	1.10	0.05
Omitting K. Nakashima, 2022		0.94	[0.75; 1.19]	0.69	-0.08	-0.02	0.00	1.09	0.04
Omitting N. I. P. Neto, 2018		0.94	[0.75; 1.18]	0.69	-0.54	-0.06	0.00	1.04	0.01
Omitting L. R. Brown, 2025		0.95	[0.75; 1.20]	0.69	-0.36	-0.09	0.01	1.10	0.05
Omitting J. Miller, 2020		0.95	[0.76; 1.18]	0.69	-1.09	-0.10	0.01	1.00	0.01
Omitting M. C. Gül, 2025		0.95	[0.75; 1.20]	0.69	-0.47	-0.11	0.01	1.09	0.04
Omitting T. Mracek, 2011		0.95	[0.76; 1.19]	0.69	-0.74	-0.11	0.01	1.05	0.02
Omitting N. Johns, 2014		0.95	[0.76; 1.19]	0.68	-1.23	-0.15	0.02	0.99	0.02
Omitting L. R. Brown, 2024		0.95	[0.76; 1.20]	0.68	-0.69	-0.15	0.02	1.08	0.05
Omitting Q. Wan, 2022		0.96	[0.77; 1.21]	0.67	-1.02	-0.23	0.05	1.05	0.05
Omitting N. A. Stephens, 2015		0.96	[0.77; 1.20]	0.68	-1.53	-0.23	0.05	0.96	0.03
Omitting C. L. Zhuang, 2022		0.96	[0.77; 1.21]	0.65	-0.98	-0.24	0.06	1.06	0.05
Omitting H. Tanda, 2025		0.97	[0.78; 1.21]	0.66	-1.48	-0.32	0.10	0.99	0.05
Omitting T. Nishhiyama, 2025		0.97	[0.78; 1.21]	0.65	-1.65	-0.34	0.11	0.97	0.04
**Random effects model**		**0.94**	**[0.75; 1.17]**						

I^2 = 0% [0%; 42%]

Figure S187: Funnel plot: Association between malnutrition-related complication risk and sex in colorectal cancer (Biological composite scores)

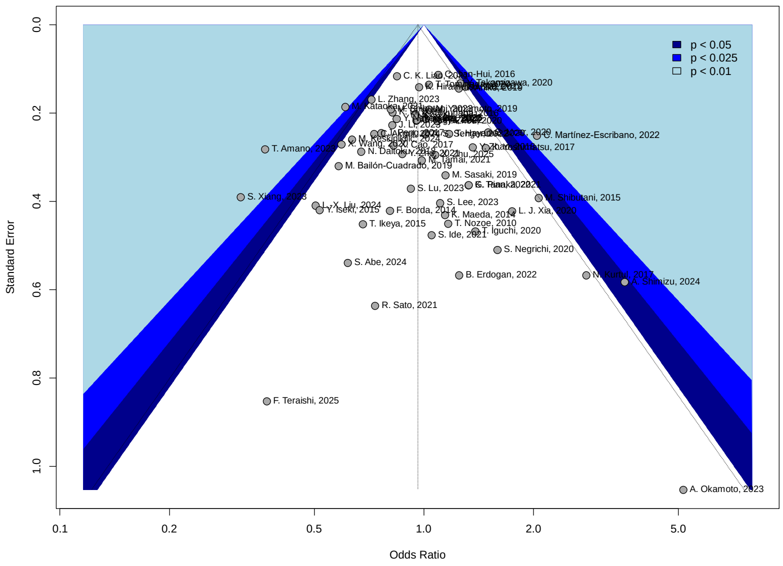

Egger test: t = 0.30, df = 60, p-value = 0.7645

Figure S187.1: Leave-one-out plot: Association between malnutrition-related complication risk and sex in colorectal cancer (Biological composite scores)

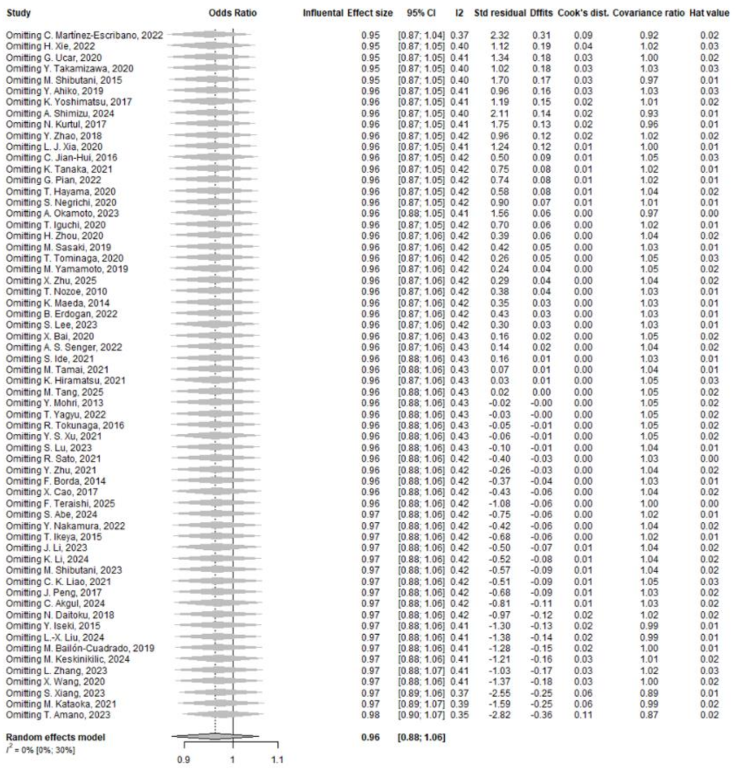

Figure S188: Funnel plot: Association between malnutrition-related complication risk and sex in esophageal cancer (Biological composite scores)

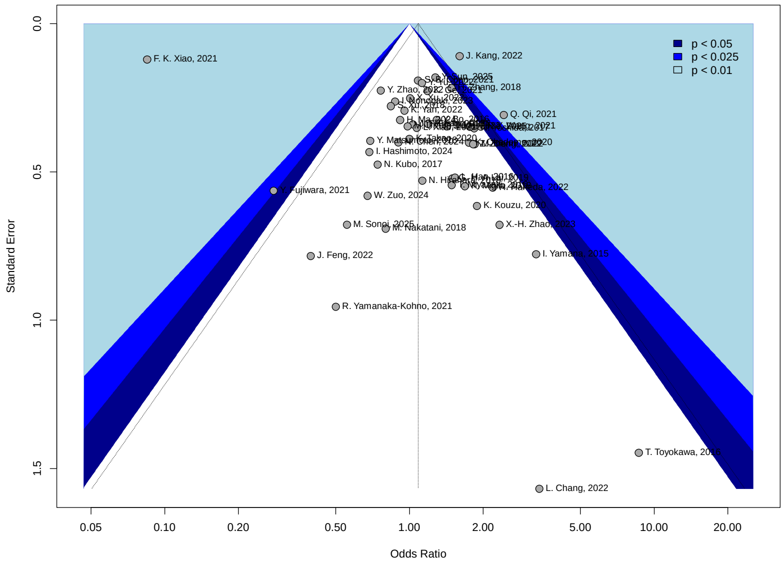

Egger test: t = 1.81, df = 46, p-value = 0.0765

Figure S188.1: Leave-one-out plot: Association between malnutrition-related complication risk and sex in esophageal cancer (Biological composite scores)

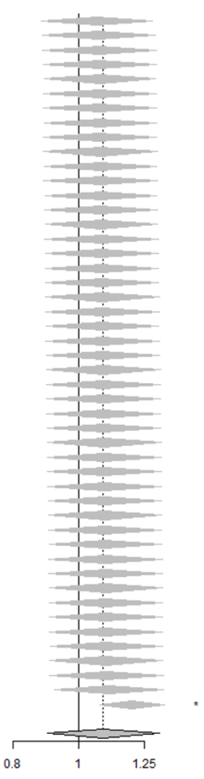

Study	Influental	Effect size	95% CI	I2	Std residual	Dffits	Cook's dist.	Covariance ratio	Hat value
Omitting Q. Qi, 2021		1.06	[0.88; 1.29]	0.90	1.40	0.23	0.05	1.01	0.03
Omitting P. Y. Wang, 2021		1.07	[0.88; 1.30]	0.90	0.97	0.15	0.02	1.03	0.02
Omitting J. Kang, 2022		1.07	[0.88; 1.30]	0.90	0.76	0.14	0.02	1.04	0.03
Omitting N. Yoshida, 2017		1.07	[0.89; 1.30]	0.90	0.87	0.13	0.02	1.03	0.02
Omitting I. Yamana, 2015		1.07	[0.89; 1.30]	0.90	1.21	0.13	0.02	1.00	0.01
Omitting X. Nie, 2025		1.07	[0.89; 1.30]	0.90	0.80	0.12	0.02	1.03	0.02
Omitting M. Demir, 2022		1.07	[0.89; 1.30]	0.90	0.83	0.12	0.01	1.03	0.02
Omitting R. Haneda, 2022		1.07	[0.89; 1.30]	0.90	0.94	0.12	0.01	1.02	0.02
Omitting Z. Zheng, 2022		1.07	[0.89; 1.30]	0.90	0.81	0.12	0.01	1.03	0.02
Omitting K. Okadome, 2020		1.08	[0.89; 1.30]	0.90	0.75	0.11	0.01	1.03	0.02
Omitting X.-H. Zhao, 2023		1.08	[0.89; 1.30]	0.90	0.91	0.10	0.01	1.02	0.01
Omitting H. Zhang, 2018		1.08	[0.89; 1.31]	0.90	0.57	0.09	0.01	1.04	0.03
Omitting T. Toyokawa, 2016		1.08	[0.89; 1.30]	0.90	1.36	0.09	0.01	0.99	0.00
Omitting K. Kouzu, 2020		1.08	[0.89; 1.31]	0.90	0.70	0.08	0.01	1.02	0.01
Omitting K. Migita, 2018		1.08	[0.89; 1.31]	0.90	0.59	0.07	0.01	1.03	0.02
Omitting J. Liu, 2022		1.08	[0.89; 1.31]	0.90	0.44	0.06	0.00	1.04	0.02
Omitting L. Han, 2016		1.08	[0.89; 1.31]	0.90	0.48	0.06	0.00	1.03	0.02
Omitting G. H. A-Lai, 2019		1.08	[0.89; 1.31]	0.90	0.44	0.05	0.00	1.03	0.02
Omitting T. Miyazaki, 2016		1.08	[0.89; 1.31]	0.90	0.42	0.05	0.00	1.03	0.02
Omitting Y. Sun, 2025		1.08	[0.89; 1.31]	0.90	0.30	0.05	0.00	1.05	0.03
Omitting Y. Bo, 2016		1.08	[0.89; 1.31]	0.90	0.29	0.04	0.00	1.04	0.02
Omitting L. Chang, 2022		1.08	[0.89; 1.31]	0.90	0.69	0.04	0.00	1.01	0.00
Omitting K. Ge, 2021		1.08	[0.89; 1.32]	0.90	0.16	0.02	0.00	1.05	0.03
Omitting P. Fang, 2022		1.08	[0.89; 1.32]	0.90	0.15	0.02	0.00	1.04	0.02
Omitting Y. Yu, 2022		1.09	[0.89; 1.32]	0.90	0.06	0.00	0.00	1.05	0.03
Omitting N. Hirahara, 2018		1.09	[0.90; 1.32]	0.90	0.05	-0.00	0.00	1.04	0.02
Omitting S. B. Chen, 2021		1.09	[0.90; 1.32]	0.90	-0.01	-0.01	0.00	1.05	0.03
Omitting L. Xiao, 2021		1.09	[0.90; 1.32]	0.90	-0.02	-0.01	0.00	1.04	0.02
Omitting J. F. Feng, 2013		1.09	[0.90; 1.32]	0.90	-0.08	-0.02	0.00	1.05	0.02
Omitting K. Takao, 2020		1.09	[0.90; 1.32]	0.90	-0.13	-0.03	0.00	1.04	0.02
Omitting X. Xu, 2022		1.09	[0.90; 1.32]	0.90	-0.14	-0.03	0.00	1.05	0.03
Omitting M. Urabe, 2021		1.09	[0.90; 1.32]	0.90	-0.17	-0.03	0.00	1.04	0.02
Omitting K. Yan, 2022		1.09	[0.90; 1.32]	0.90	-0.22	-0.04	0.00	1.05	0.03
Omitting M. Nakatani, 2018		1.09	[0.90; 1.32]	0.90	-0.36	-0.05	0.00	1.03	0.01
Omitting N. Chen, 2024		1.09	[0.90; 1.32]	0.90	-0.29	-0.05	0.00	1.04	0.02
Omitting H. Ma, 2024		1.09	[0.90; 1.32]	0.90	-0.29	-0.05	0.00	1.04	0.02
Omitting R. Yamanaka-Kohno, 2021		1.09	[0.90; 1.32]	0.90	-0.72	-0.07	0.00	1.02	0.01
Omitting I. Nonogaki, 2023		1.09	[0.90; 1.33]	0.90	-0.39	-0.07	0.01	1.05	0.03
Omitting S. Xu, 2018		1.09	[0.90; 1.33]	0.90	-0.45	-0.08	0.01	1.04	0.03
Omitting N. Kubo, 2017		1.09	[0.90; 1.33]	0.90	-0.56	-0.08	0.01	1.03	0.02
Omitting W. Zuo, 2024		1.09	[0.90; 1.33]	0.90	-0.62	-0.08	0.01	1.03	0.01
Omitting M. Sonoi, 2025		1.10	[0.91; 1.33]	0.90	-0.80	-0.09	0.01	1.02	0.01
Omitting I. Hashimoto, 2024		1.10	[0.91; 1.33]	0.90	-0.70	-0.10	0.01	1.03	0.02
Omitting J. Feng, 2022		1.10	[0.91; 1.33]	0.90	-1.09	-0.11	0.01	1.01	0.01
Omitting Y. Matsumoto, 2018		1.10	[0.91; 1.33]	0.90	-0.71	-0.11	0.01	1.03	0.02
Omitting Y. Zhao, 2022		1.10	[0.91; 1.33]	0.90	-0.64	-0.12	0.01	1.04	0.03
Omitting Y. Fujiwara, 2021		1.11	[0.92; 1.33]	0.90	-1.84	-0.21	0.04	0.97	0.02
Omitting F. K. Xiao, 2021	*	1.20	[1.07; 1.34]	0.18	-13.63	-2.91	1.10	0.34	0.03
**Random effects model**		**1.09**	**[0.90; 1.31]**						

I^2 = 0% [0%; 34%]

Figure S189: Funnel plot: Association between malnutrition-related complication risk and sex in gastric cancer (Biological composite scores)

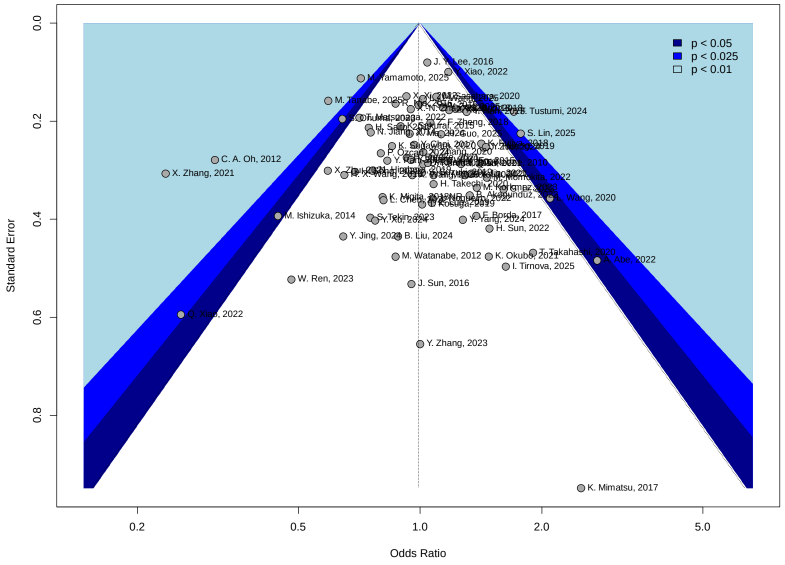

Egger test: t = -0.05, df = 78, p-value = 0.9592

Figure S190: Funnel plot: Association between malnutrition-related complication risk and sex in hepatocellular carcinoma (Biological composite scores)

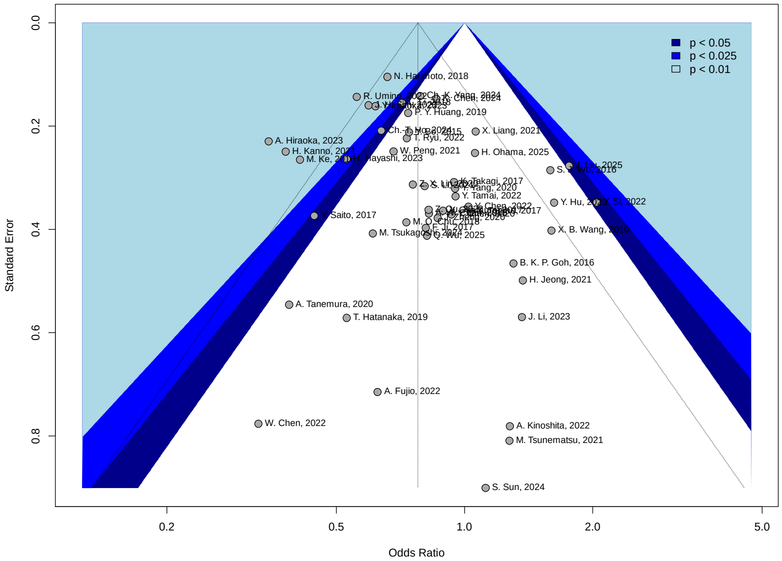

Egger test: t = 1.82, df = 48, p-value = 0.0749

Figure S190.1: Leave-one-out plot: Association between malnutrition-related complication risk and sex in hepatocellular carcinoma (Biological composite scores)

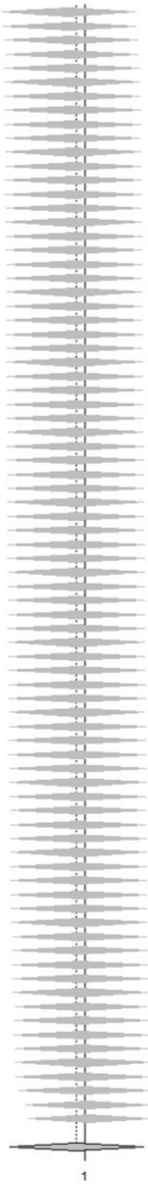

Study	Influential	Effect size	95% CI	I2	Std residual	Dffits	Cook's dist.	Covariance ratio	Hat value
Omitting F. Tustumi, 2024		0.98	[0.90; 1.06]	0.45	1.84	0.25	0.06	0.98	0.02
Omitting S. Lin, 2025		0.98	[0.90; 1.06]	0.46	1.79	0.22	0.05	0.98	0.02
Omitting L. Wang, 2020		0.98	[0.91; 1.07]	0.47	1.75	0.17	0.03	0.98	0.01
Omitting A. Abe, 2022		0.98	[0.91; 1.07]	0.47	1.90	0.14	0.02	0.97	0.01
Omitting Y. Huang, 2019		0.98	[0.91; 1.07]	0.48	1.10	0.13	0.02	1.01	0.01
Omitting K. Fujiya, 2018		0.98	[0.91; 1.07]	0.48	1.04	0.12	0.02	1.01	0.01
Omitting J. Zuo, 2024		0.98	[0.91; 1.07]	0.48	1.06	0.12	0.02	1.01	0.01
Omitting Y. Han, 2023		0.98	[0.91; 1.07]	0.48	0.91	0.12	0.02	1.02	0.02
Omitting S. Ryo, 2018		0.99	[0.91; 1.07]	0.48	0.86	0.12	0.01	1.02	0.02
Omitting G. Li, 2023		0.99	[0.91; 1.07]	0.48	1.16	0.12	0.01	1.00	0.01
Omitting T. Nozoe, 2010		0.99	[0.91; 1.07]	0.48	0.93	0.10	0.01	1.01	0.01
Omitting M. Momokita, 2022		0.99	[0.91; 1.07]	0.48	0.98	0.10	0.01	1.01	0.01
Omitting Y. Xiao, 2022		0.99	[0.91; 1.07]	0.48	0.65	0.10	0.01	1.03	0.02
Omitting X. Liu, 2018		0.99	[0.91; 1.07]	0.48	0.70	0.10	0.01	1.03	0.02
Omitting T. Takahashi, 2020		0.99	[0.91; 1.07]	0.48	1.24	0.10	0.01	1.00	0.01
Omitting H. Jin, 2021		0.99	[0.91; 1.07]	0.48	0.77	0.08	0.01	1.02	0.01
Omitting M. Korkmaz, 2023		0.99	[0.91; 1.07]	0.48	0.80	0.08	0.01	1.02	0.01
Omitting Z. Xu, 2022		0.99	[0.91; 1.07]	0.48	0.58	0.08	0.01	1.03	0.02
Omitting H. Sun, 2022		0.99	[0.91; 1.07]	0.48	0.84	0.07	0.01	1.01	0.01
Omitting Y. Qian, 2021		0.99	[0.91; 1.07]	0.48	0.65	0.07	0.01	1.02	0.01
Omitting J. Y. Liu, 2021		0.99	[0.91; 1.07]	0.48	0.67	0.07	0.01	1.02	0.01
Omitting I. Tirnova, 2025		0.99	[0.91; 1.07]	0.48	0.90	0.07	0.00	1.01	0.01
Omitting B. Akagunduz, 2022		0.99	[0.91; 1.07]	0.48	0.68	0.07	0.00	1.02	0.01
Omitting Y. Zou, 2025		0.99	[0.91; 1.07]	0.48	0.46	0.06	0.00	1.03	0.02
Omitting F. Borda, 2017		0.99	[0.91; 1.07]	0.48	0.71	0.06	0.00	1.02	0.01
Omitting K. Okubo, 2021		0.99	[0.91; 1.07]	0.48	0.75	0.06	0.00	1.01	0.01
Omitting M. Sasahara, 2020		0.99	[0.91; 1.07]	0.49	0.36	0.05	0.00	1.04	0.02
Omitting W. K. Eo, 2015		0.99	[0.91; 1.07]	0.49	0.44	0.05	0.00	1.03	0.01
Omitting H. Guo, 2025		0.99	[0.91; 1.07]	0.49	0.39	0.05	0.00	1.03	0.02
Omitting Y. Yang, 2024		0.99	[0.91; 1.07]	0.49	0.54	0.05	0.00	1.02	0.01
Omitting K. Mimatsu, 2017		0.99	[0.91; 1.07]	0.48	0.95	0.04	0.00	1.00	0.00
Omitting J. Y. Lee, 2016		0.99	[0.91; 1.08]	0.49	0.20	0.03	0.00	1.04	0.03
Omitting Z. F. Zheng, 2018		0.99	[0.91; 1.08]	0.49	0.22	0.03	0.00	1.03	0.02
Omitting H. Miyamoto, 2024		0.99	[0.91; 1.08]	0.49	0.22	0.02	0.00	1.03	0.01
Omitting H. Takechi, 2020		0.99	[0.91; 1.08]	0.49	0.21	0.02	0.00	1.03	0.01
Omitting J. Nogueiro, 2022		0.99	[0.91; 1.08]	0.49	0.19	0.02	0.00	1.02	0.01
Omitting Z. Luo, 2019		0.99	[0.91; 1.08]	0.49	0.18	0.02	0.00	1.02	0.01
Omitting M. Kanda, 2016		0.99	[0.91; 1.08]	0.49	0.15	0.02	0.00	1.03	0.01
Omitting L.-J. Wang, 2025		0.99	[0.91; 1.08]	0.49	0.09	0.01	0.00	1.04	0.02
Omitting X. Zhang, 2020		0.99	[0.91; 1.08]	0.49	0.08	0.01	0.00	1.03	0.01
Omitting D. Kuroda, 2018		0.99	[0.91; 1.08]	0.49	0.07	0.01	0.00	1.03	0.01
Omitting S. Suzuki, 2019		0.99	[0.91; 1.08]	0.49	0.05	0.01	0.00	1.03	0.01
Omitting T. Kosuga, 2019		0.99	[0.91; 1.08]	0.49	0.05	0.00	0.00	1.02	0.01
Omitting Y. Zhang, 2023		0.99	[0.91; 1.08]	0.49	0.01	0.00	0.00	1.02	0.00
Omitting K. Sun, 2015		0.99	[0.91; 1.08]	0.49	0.00	0.00	0.00	1.04	0.02
Omitting J. Sun, 2016		0.99	[0.91; 1.08]	0.49	-0.07	-0.00	0.00	1.02	0.00
Omitting Y. Choi, 2017		0.99	[0.91; 1.08]	0.49	-0.06	-0.01	0.00	1.03	0.01
Omitting X. Wang, 2023		0.99	[0.91; 1.08]	0.49	-0.09	-0.01	0.00	1.03	0.01
Omitting Y. Zhang, 2020		0.99	[0.91; 1.08]	0.49	-0.11	-0.01	0.00	1.03	0.01
Omitting X. Ma, 2024		0.99	[0.91; 1.08]	0.49	-0.15	-0.02	0.00	1.03	0.02
Omitting M. Watanabe, 2012		0.99	[0.91; 1.08]	0.49	-0.24	-0.02	0.00	1.02	0.01
Omitting B. Liu, 2024		0.99	[0.91; 1.08]	0.49	-0.23	-0.02	0.00	1.02	0.01
Omitting X.-N. Zhao, 2024		0.99	[0.91; 1.08]	0.49	-0.14	-0.02	0.00	1.03	0.02
Omitting H. Furuke, 2021		0.99	[0.91; 1.08]	0.49	-0.18	-0.02	0.00	1.03	0.01
Omitting X. Xi, 2022		0.99	[0.91; 1.08]	0.49	-0.24	-0.03	0.00	1.04	0.02
Omitting K. Sakurai, 2015		0.99	[0.91; 1.08]	0.49	-0.31	-0.04	0.00	1.03	0.02
Omitting L. Chen, 2022		0.99	[0.91; 1.08]	0.49	-0.45	-0.04	0.00	1.02	0.01
Omitting K. Migita, 2018NR		0.99	[0.91; 1.08]	0.49	-0.47	-0.05	0.00	1.02	0.01
Omitting Y. Xu, 2024		0.99	[0.91; 1.08]	0.49	-0.52	-0.05	0.00	1.02	0.01
Omitting K. Sugawara, 2020		0.99	[0.91; 1.08]	0.49	-0.43	-0.05	0.00	1.03	0.01
Omitting S. Tekin, 2023		0.99	[0.91; 1.08]	0.49	-0.59	-0.05	0.00	1.02	0.01
Omitting Y. Pan, 2023		0.99	[0.91; 1.08]	0.49	-0.47	-0.05	0.00	1.03	0.01
Omitting R. Nie, 2016		0.99	[0.91; 1.08]	0.48	-0.44	-0.06	0.00	1.03	0.02
Omitting P. Özcan, 2024		0.99	[0.91; 1.08]	0.48	-0.59	-0.07	0.00	1.02	0.01
Omitting Y. Jing, 2024		0.99	[0.91; 1.08]	0.48	-0.86	-0.07	0.01	1.01	0.01
Omitting N. Hirahara, 2019		0.99	[0.91; 1.08]	0.48	-0.67	-0.07	0.01	1.02	0.01
Omitting W. Ren, 2023		0.99	[0.92; 1.08]	0.48	-1.26	-0.09	0.01	1.00	0.01
Omitting H. Song, 2022		0.99	[0.91; 1.08]	0.48	-0.93	-0.10	0.01	1.01	0.01
Omitting N. Jiang, 2014		0.99	[0.91; 1.08]	0.48	-0.82	-0.10	0.01	1.02	0.02
Omitting H. Saito, 2017		0.99	[0.92; 1.08]	0.48	-0.87	-0.11	0.01	1.02	0.02
Omitting H. X. Wang, 2018		0.99	[0.92; 1.08]	0.48	-1.07	-0.11	0.01	1.01	0.01
Omitting Q. Xiao, 2022		1.00	[0.92; 1.08]	0.47	-2.12	-0.13	0.02	0.95	0.00
Omitting X. Zhu, 2021		1.00	[0.92; 1.08]	0.48	-1.34	-0.14	0.02	1.00	0.01
Omitting T. Matsunaga, 2022		1.00	[0.92; 1.08]	0.48	-1.08	-0.14	0.02	1.02	0.02
Omitting M. Ishizuka, 2014		1.00	[0.92; 1.08]	0.47	-1.74	-0.15	0.02	0.98	0.01
Omitting S. Onuma, 2023		1.00	[0.92; 1.08]	0.47	-1.40	-0.19	0.03	1.00	0.02
Omitting M. Yamamoto, 2025		1.00	[0.92; 1.08]	0.45	-1.23	-0.19	0.04	1.02	0.02
Omitting M. Tanabe, 2025		1.00	[0.92; 1.09]	0.45	-1.81	-0.26	0.07	0.99	0.02
Omitting C. A. Oh, 2012	*	1.00	[0.93; 1.08]	0.42	-3.33	-0.36	0.12	0.87	0.01
Omitting X. Zhang, 2021	*	1.01	[0.93; 1.08]	0.40	-3.97	-0.39	0.13	0.81	0.01
**Random effects model**		**0.99**	**[0.91; 1.08]**						

I^2 = 0% [0%; 27%]

Figure S191: Funnel plot: Association between malnutrition-related complication risk and sex in PDAC cancer (Biological composite scores)

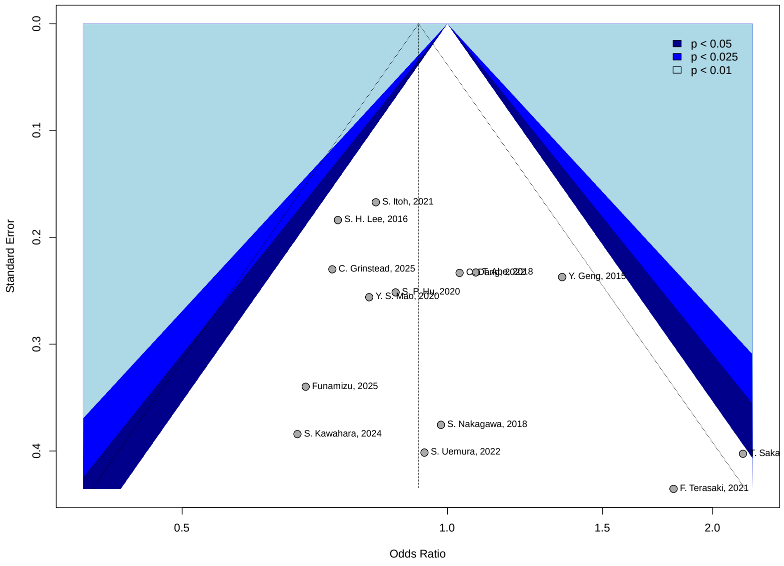

Egger test: t = 0.81, df = 12, p-value = 0.4329

Figure S191.1: Leave-one-out plot: Association between malnutrition-related complication risk and sex in PDAC cancer (Biological composite scores)

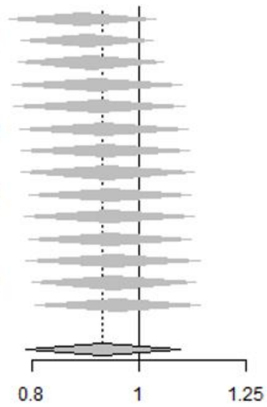

Study	Odds Ratio	Influental	Effect size	95% CI	I2	Std residual	Dffits	Cook's dist.	Covariance ratio	Hat value
Omitting Y. Geng, 2015			0.89	[0.76; 1.04]	0.00	1.68	0.61	0.32	0.93	0.09
Omitting T. Sakamoto, 2021			0.90	[0.78; 1.03]	0.00	2.15	0.45	0.19	0.88	0.03
Omitting F. Terasaki, 2021			0.91	[0.78; 1.05]	0.00	1.57	0.33	0.11	0.88	0.03
Omitting T. Abe, 2018			0.92	[0.77; 1.10]	0.14	0.59	0.14	0.02	1.19	0.09
Omitting C. Dang, 2022			0.92	[0.77; 1.10]	0.15	0.41	0.08	0.01	1.21	0.09
Omitting S. Nakagawa, 2018			0.93	[0.78; 1.11]	0.16	0.14	-0.03	0.00	1.17	0.04
Omitting S. Uemura, 2022			0.93	[0.78; 1.11]	0.17	0.02	-0.06	0.00	1.17	0.03
Omitting S. P. Hu, 2020			0.94	[0.78; 1.12]	0.16	-0.24	-0.13	0.02	1.22	0.08
Omitting S. Kawahara, 2024			0.94	[0.79; 1.11]	0.12	-0.81	-0.18	0.03	1.08	0.04
Omitting Y. S. Mao, 2020			0.94	[0.79; 1.12]	0.15	-0.49	-0.19	0.04	1.19	0.08
Omitting Funamizu, 2025			0.94	[0.80; 1.12]	0.12	-0.85	-0.21	0.04	1.08	0.04
Omitting S. Itoh, 2021			0.95	[0.79; 1.14]	0.14	-0.61	-0.28	0.09	1.26	0.15
Omitting C. Grinstead, 2025			0.95	[0.80; 1.13]	0.10	-0.95	-0.31	0.09	1.11	0.09
Omitting S. H. Lee, 2016			0.96	[0.81; 1.14]	0.07	-1.11	-0.42	0.17	1.12	0.13
**Random effects model**			**0.93**	**[0.79; 1.09]**						

I^2 = 0% [0%; 55%]

Figure S192: Funnel plot: Association between malnutrition risk and sex in colorectal cancer (Symptom-based risk assessment tools)

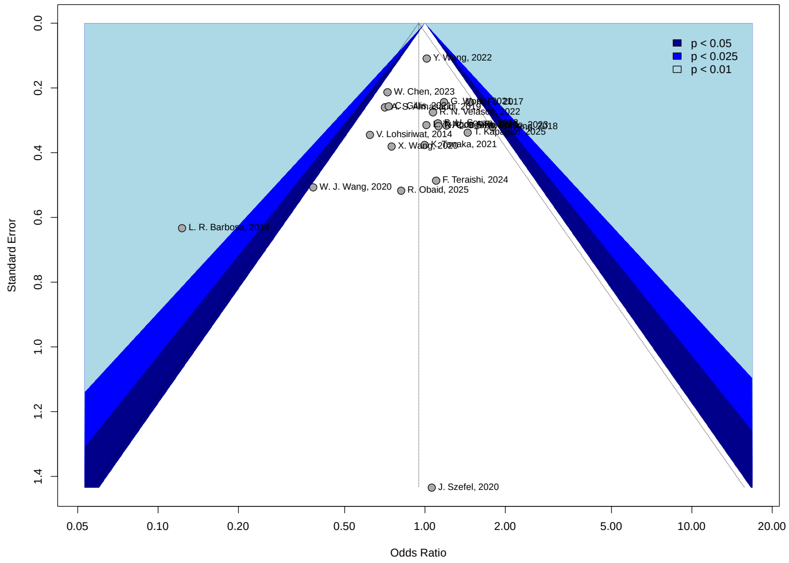

Egger test: t = -1.44, df = 19, p-value = 0.1650

Figure S192.1: Leave-one-out plot: Association between malnutrition risk and sex in colorectal cancer (Symptom-based risk assessment tools)

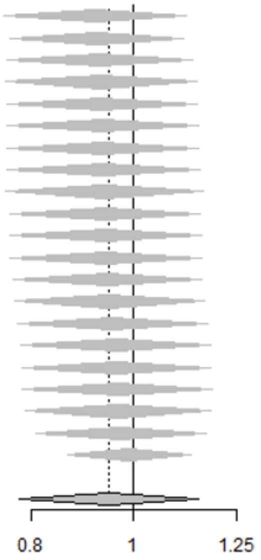

Study	Influental	Effect size	95% CI	I2	Std residual	Dffits	Cook's dist.	Covariance ratio	Hat value
Omitting K. Fu, 2017		0.92	[0.76; 1.12]	0.31	1.27	0.32	0.09	1.02	0.06
Omitting Y. Wang, 2018		0.92	[0.76; 1.11]	0.30	1.62	0.31	0.09	0.92	0.05
Omitting T. Kapagan, 2025		0.93	[0.76; 1.13]	0.35	0.99	0.22	0.05	1.05	0.05
Omitting G. Wong, 2021		0.93	[0.76; 1.15]	0.37	0.60	0.18	0.03	1.12	0.06
Omitting A. da Silva Couto, 2023		0.93	[0.76; 1.15]	0.37	0.58	0.15	0.02	1.11	0.05
Omitting B. U. Souza, 2018		0.94	[0.76; 1.15]	0.38	0.41	0.12	0.02	1.13	0.05
Omitting N. C. Souza, 2021		0.94	[0.76; 1.15]	0.38	0.41	0.12	0.01	1.13	0.05
Omitting R. N. Velasco, 2022		0.94	[0.76; 1.16]	0.38	0.32	0.11	0.01	1.14	0.06
Omitting Y. Wang, 2022		0.94	[0.76; 1.16]	0.38	0.24	0.10	0.01	1.19	0.10
Omitting F. Teraishi, 2024		0.94	[0.77; 1.16]	0.38	0.27	0.08	0.01	1.11	0.03
Omitting L. T. Ngoc Anh, 2024		0.94	[0.76; 1.16]	0.38	0.17	0.07	0.00	1.14	0.05
Omitting K. Tanaka, 2021		0.94	[0.77; 1.16]	0.38	0.11	0.05	0.00	1.13	0.04
Omitting J. Szefel, 2020		0.94	[0.77; 1.16]	0.38	0.08	0.04	0.00	1.09	0.00
Omitting R. Obaid, 2025		0.95	[0.77; 1.17]	0.38	-0.25	-0.01	0.00	1.11	0.03
Omitting X. Wang, 2020		0.95	[0.78; 1.17]	0.37	-0.49	-0.08	0.01	1.11	0.04
Omitting C. Gillis, 2021		0.96	[0.78; 1.18]	0.35	-0.69	-0.16	0.03	1.11	0.06
Omitting A. S. Almasaudi, 2019		0.96	[0.79; 1.18]	0.35	-0.78	-0.18	0.04	1.10	0.06
Omitting W. Chen, 2023		0.97	[0.79; 1.19]	0.34	-0.78	-0.20	0.04	1.11	0.07
Omitting V. Lohsiriwat, 2014		0.97	[0.79; 1.18]	0.35	-0.97	-0.21	0.04	1.05	0.04
Omitting W. J. Wang, 2020		0.97	[0.81; 1.17]	0.30	-1.65	-0.30	0.09	0.90	0.03
Omitting L. R. Barbosa, 2014		0.99	[0.87; 1.13]	0.05	-3.26	-0.52	0.23	0.47	0.02
**Random effects model**		**0.95**	**[0.78; 1.15]**						

I^2 = 0% [0%; 47%]

Figure S193: Funnel plot: Association between malnutrition-related complication risk and smoking (active or history) in esophageal cancer (Biological composite scores)

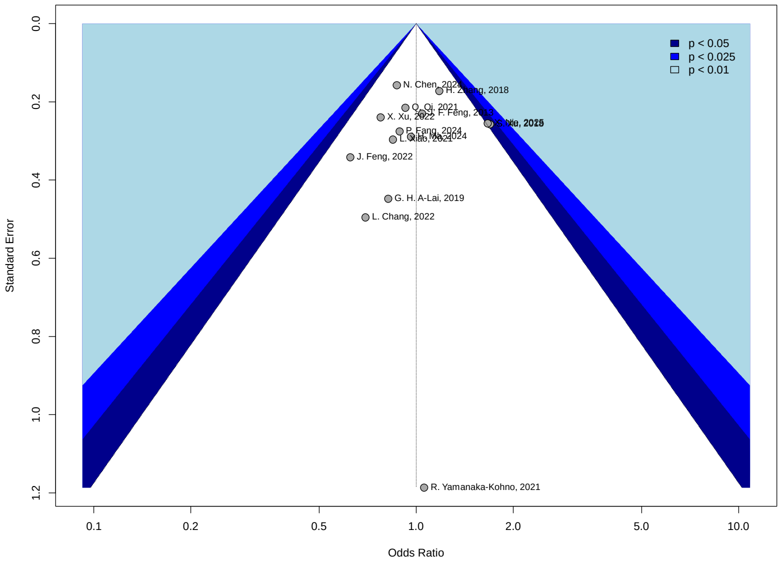

Egger test: t = -0.50, df = 12, p-value = 0.6267

Figure S193.1: Leave-one-out plot: Association between malnutrition-related complication risk and smoking (active or history) in esophageal cancer (Biological composite scores)

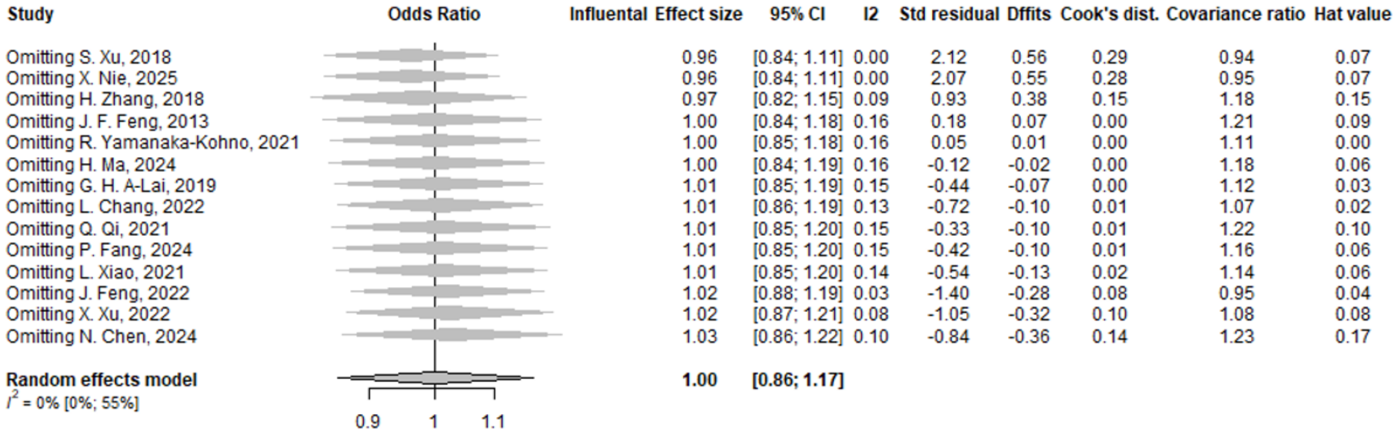

Figure S194: Funnel plot: Association between malnutrition-related complication risk and T stage (T>1 vs. T≤1) in esophageal cancer (Biological composite scores)

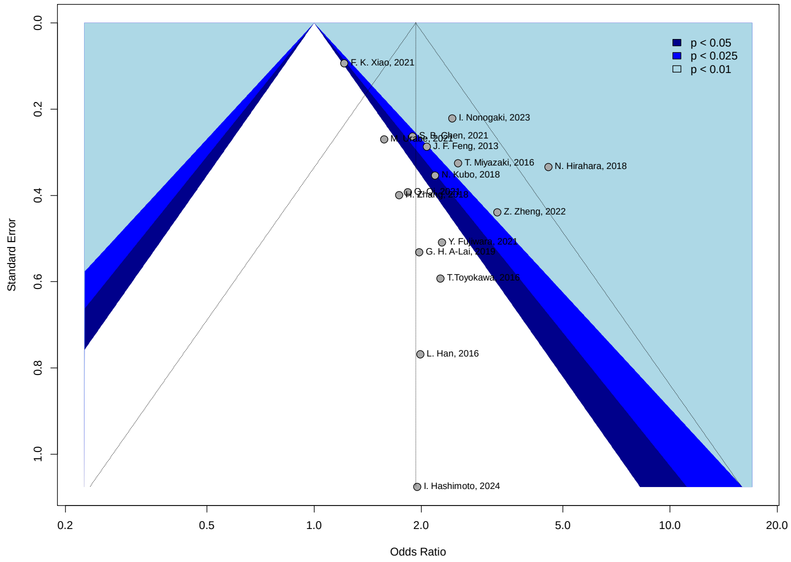

Egger test: t = 4.44, df = 14, p-value = 0.0006

Figure S194.1: Leave-one-out plot: Association between malnutrition-related complication risk and T stage (T>1 vs. T≤1) in esophageal cancer (Biological composite scores)

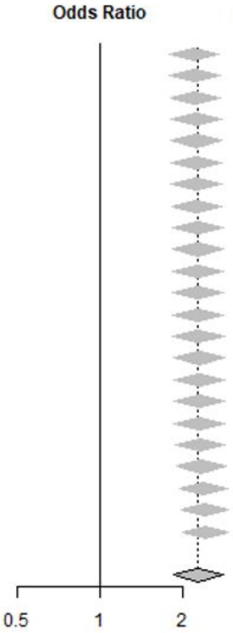

Study	Influental	Effect size	95% CI	I2	Std residual	Dffits	Cook's dist.	Covariance ratio	Hat value
Omitting J. Y. Lee, 2016		2.21	[1.79; 2.71]	0.63	1.51	0.39	0.14	1.01	0.07
Omitting M. Yamamoto, 2025		2.22	[1.80; 2.75]	0.71	1.16	0.30	0.09	1.05	0.07
Omitting T. Nozoe, 2010		2.23	[1.81; 2.75]	0.72	1.22	0.26	0.06	1.03	0.05
Omitting Y. Qian, 2021		2.24	[1.82; 2.77]	0.73	0.95	0.20	0.04	1.05	0.04
Omitting T. Matsunaga, 2022		2.24	[1.81; 2.78]	0.73	0.74	0.19	0.04	1.09	0.06
Omitting D. Kuroda, 2018		2.25	[1.82; 2.78]	0.73	0.76	0.18	0.03	1.07	0.04
Omitting K. Fujiya, 2018		2.25	[1.82; 2.79]	0.73	0.61	0.16	0.03	1.09	0.05
Omitting P. Özcan, 2024		2.26	[1.83; 2.79]	0.73	0.79	0.14	0.02	1.05	0.03
Omitting M. Kanda, 2016		2.27	[1.84; 2.81]	0.73	0.47	0.08	0.01	1.06	0.02
Omitting S. Lin, 2025		2.27	[1.83; 2.82]	0.73	0.23	0.08	0.01	1.11	0.05
Omitting K. Sakurai, 2015		2.27	[1.83; 2.83]	0.73	0.16	0.06	0.00	1.11	0.06
Omitting K. Ge, 2021		2.28	[1.84; 2.83]	0.73	0.08	0.04	0.00	1.10	0.04
Omitting M. Ishizuka, 2014		2.28	[1.84; 2.83]	0.73	0.05	0.03	0.00	1.09	0.03
Omitting M. Tanabe, 2025		2.29	[1.84; 2.85]	0.72	-0.09	0.00	0.00	1.12	0.06
Omitting T. Kosuga, 2019		2.30	[1.86; 2.84]	0.73	-0.35	-0.04	0.00	1.07	0.03
Omitting M. Sasahara, 2020		2.30	[1.86; 2.85]	0.72	-0.34	-0.05	0.00	1.09	0.04
Omitting H. Saito, 2017		2.30	[1.85; 2.86]	0.72	-0.32	-0.06	0.00	1.11	0.06
Omitting I. Tirnova, 2025		2.30	[1.86; 2.85]	0.72	-0.57	-0.06	0.00	1.05	0.02
Omitting T. Takahashi, 2020		2.33	[1.89; 2.87]	0.72	-0.97	-0.17	0.03	1.03	0.03
Omitting S. Ryo, 2018		2.34	[1.90; 2.88]	0.71	-1.13	-0.22	0.05	1.02	0.03
Omitting N. Hirahara, 2019		2.39	[1.96; 2.92]	0.68	-1.92	-0.48	0.21	0.92	0.04
Omitting Z. Xishan, 2020		2.41	[1.99; 2.92]	0.67	-2.26	-0.57	0.28	0.86	0.04
Omitting X. Zhu, 2021		2.41	[1.99; 2.92]	0.67	-2.29	-0.58	0.29	0.86	0.04
**Random effects model**		**2.29**	**[1.86; 2.81]**						

I^2 = 0% [0%; 45%]

Figure S195: Funnel plot: Association between malnutrition-related complication risk and T stage (T4 vs. <T4) in esophageal cancer (Biological composite scores)

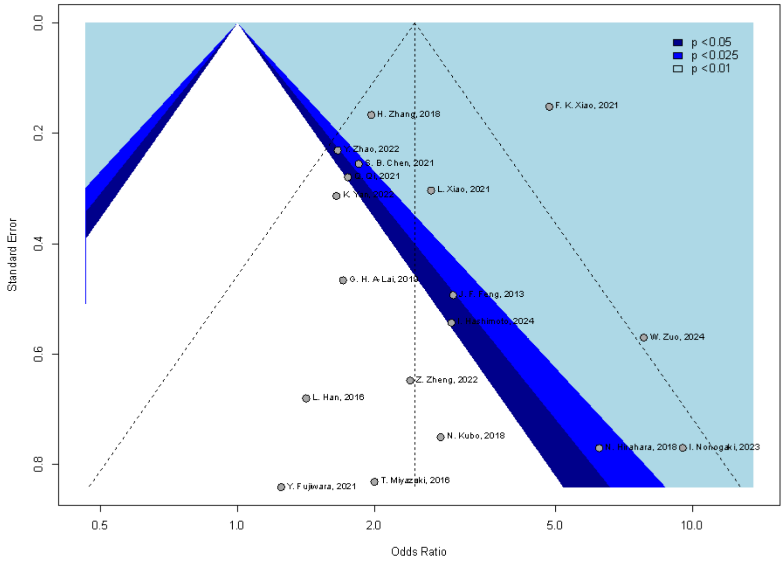

Egger test: t = -1.62, df = 16, p-value = 0.1245

Figure S195.1: Leave-one-out plot: Association between malnutrition-related complication risk and T stage (T4 vs. <T4) in esophageal cancer (Biological composite scores)

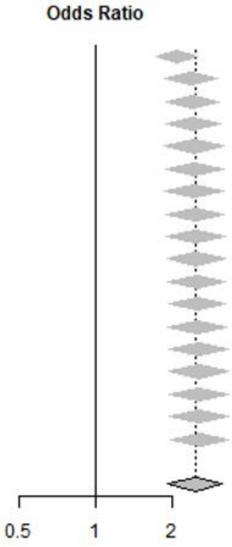

Study	Influental	Effect size	95% CI	I2	Std residual	Dffits	Cook's dist.	Covariance ratio	Hat value
Omitting F. K. Xiao, 2021	*	2.08	[1.72; 2.51]	0.01	4.58	2.84	1.92	0.57	0.13
Omitting W. Zuo, 2024		2.36	[1.87; 2.99]	0.55	1.90	0.33	0.10	0.87	0.03
Omitting I. Nonogaki, 2023		2.39	[1.88; 3.03]	0.57	1.69	0.23	0.05	0.90	0.02
Omitting N. Hirahara, 2018		2.40	[1.87; 3.09]	0.58	1.15	0.17	0.03	1.00	0.02
Omitting J. F. Feng, 2013		2.43	[1.87; 3.17]	0.60	0.34	0.06	0.00	1.12	0.04
Omitting I. Hashimoto, 2024		2.44	[1.87; 3.18]	0.60	0.30	0.05	0.00	1.11	0.04
Omitting L. Xiao, 2021		2.44	[1.86; 3.20]	0.60	0.19	0.05	0.00	1.16	0.08
Omitting N. Kubo, 2018		2.45	[1.88; 3.19]	0.60	0.16	0.01	0.00	1.10	0.02
Omitting Z. Zheng, 2022		2.46	[1.89; 3.20]	0.60	-0.03	-0.02	0.00	1.11	0.03
Omitting T. Miyazaki, 2016		2.46	[1.90; 3.21]	0.60	-0.23	-0.04	0.00	1.09	0.02
Omitting Y. Fujiwara, 2021		2.48	[1.92; 3.21]	0.59	-0.76	-0.11	0.01	1.05	0.02
Omitting L. Han, 2016		2.49	[1.92; 3.22]	0.59	-0.75	-0.13	0.02	1.06	0.03
Omitting G. H. A-Lai, 2019		2.50	[1.92; 3.25]	0.59	-0.67	-0.16	0.02	1.09	0.05
Omitting S. B. Chen, 2021		2.53	[1.93; 3.30]	0.58	-0.75	-0.24	0.06	1.14	0.09
Omitting H. Zhang, 2018		2.53	[1.92; 3.32]	0.56	-0.68	-0.25	0.07	1.18	0.12
Omitting Q. Qi, 2021		2.53	[1.94; 3.30]	0.58	-0.87	-0.27	0.07	1.11	0.09
Omitting K. Yan, 2022		2.53	[1.95; 3.29]	0.58	-0.96	-0.28	0.08	1.09	0.08
Omitting Y. Zhao, 2022		2.56	[1.97; 3.33]	0.55	-1.12	-0.37	0.14	1.09	0.10
**Random effects model**		**2.45**	**[1.91; 3.15]**						

I^2 = 0% [0%; 50%]

Figure S196: Funnel plot: Association between malnutrition-related complication risk and T stage (T>1 vs. T≤1) in gastric cancer (Biological composite scores)

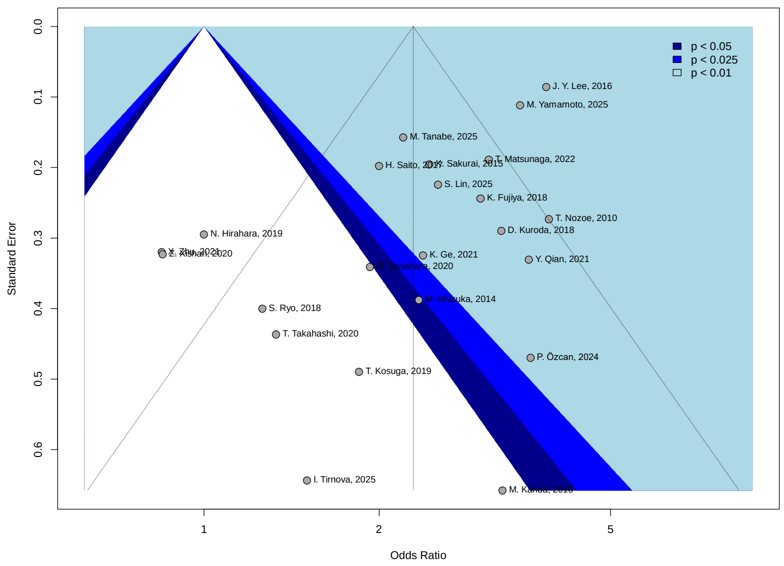

Egger test:t = -2.51, df = 21, p-value = 0.0204

Figure S196.1: Leave-one-out plot: Association between malnutrition-related complication risk and T stage (T>1 vs. T≤1) in gastric cancer (Biological composite scores)

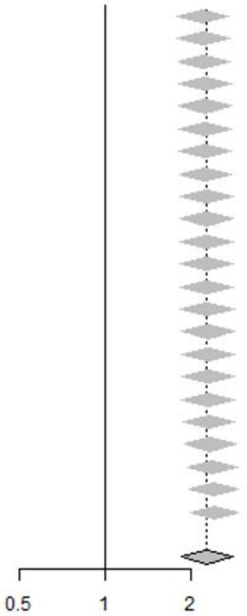

Study	Influental	Effect size	95% CI	I2	Std residual	Dffits	Cook's dist.	Covariance ratio	Hat value
Omitting J. Y. Lee, 2016		2.21	[1.79; 2.71]	0.63	1.51	0.39	0.14	1.01	0.07
Omitting M. Yamamoto, 2025		2.22	[1.80; 2.75]	0.71	1.16	0.30	0.09	1.05	0.07
Omitting T. Nozoe, 2010		2.23	[1.81; 2.75]	0.72	1.22	0.26	0.06	1.03	0.05
Omitting Y. Qian, 2021		2.24	[1.82; 2.77]	0.73	0.95	0.20	0.04	1.05	0.04
Omitting T. Matsunaga, 2022		2.24	[1.81; 2.78]	0.73	0.74	0.19	0.04	1.09	0.06
Omitting D. Kuroda, 2018		2.25	[1.82; 2.78]	0.73	0.76	0.18	0.03	1.07	0.04
Omitting K. Fujiya, 2018		2.25	[1.82; 2.79]	0.73	0.61	0.16	0.03	1.09	0.05
Omitting P. Özcan, 2024		2.26	[1.83; 2.79]	0.73	0.79	0.14	0.02	1.05	0.03
Omitting M. Kanda, 2016		2.27	[1.84; 2.81]	0.73	0.47	0.08	0.01	1.06	0.02
Omitting S. Lin, 2025		2.27	[1.83; 2.82]	0.73	0.23	0.08	0.01	1.11	0.05
Omitting K. Sakurai, 2015		2.27	[1.83; 2.83]	0.73	0.16	0.06	0.00	1.11	0.06
Omitting K. Ge, 2021		2.28	[1.84; 2.83]	0.73	0.08	0.04	0.00	1.10	0.04
Omitting M. Ishizuka, 2014		2.28	[1.84; 2.83]	0.73	0.05	0.03	0.00	1.09	0.03
Omitting M. Tanabe, 2025		2.29	[1.84; 2.85]	0.72	-0.09	0.00	0.00	1.12	0.06
Omitting T. Kosuga, 2019		2.30	[1.86; 2.84]	0.73	-0.35	-0.04	0.00	1.07	0.03
Omitting M. Sasahara, 2020		2.30	[1.86; 2.85]	0.72	-0.34	-0.05	0.00	1.09	0.04
Omitting H. Saito, 2017		2.30	[1.85; 2.86]	0.72	-0.32	-0.06	0.00	1.11	0.06
Omitting I. Tirnova, 2025		2.30	[1.86; 2.85]	0.72	-0.57	-0.06	0.00	1.05	0.02
Omitting T. Takahashi, 2020		2.33	[1.89; 2.87]	0.72	-0.97	-0.17	0.03	1.03	0.03
Omitting S. Ryo, 2018		2.34	[1.90; 2.88]	0.71	-1.13	-0.22	0.05	1.02	0.03
Omitting N. Hirahara, 2019		2.39	[1.96; 2.92]	0.68	-1.92	-0.48	0.21	0.92	0.04
Omitting Z. Xishan, 2020		2.41	[1.99; 2.92]	0.67	-2.26	-0.57	0.28	0.86	0.04
Omitting X. Zhu, 2021		2.41	[1.99; 2.92]	0.67	-2.29	-0.58	0.29	0.86	0.04
**Random effects model**		**2.29**	**[1.86; 2.81]**						

I^2 = 0% [0%; 45%]

Figure S197: Funnel plot: Association between malnutrition-related complication risk and T stage (T4 vs. <T4) in gastric cancer (Biological composite scores)

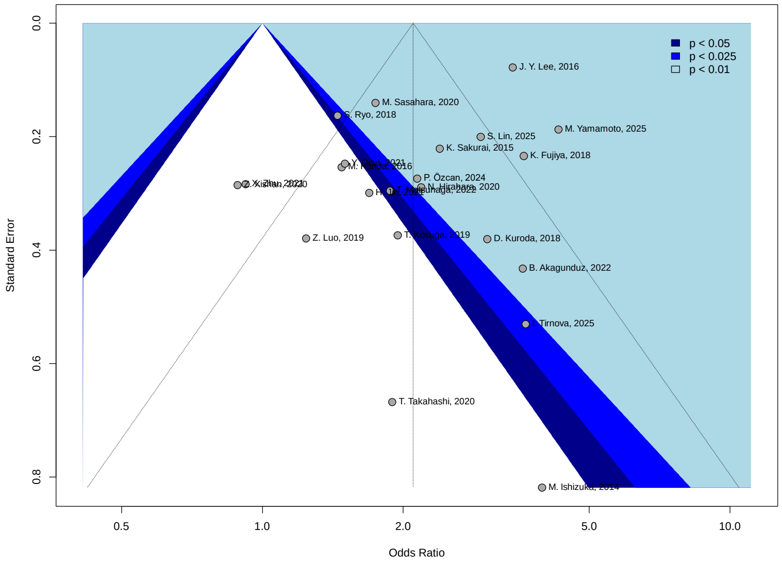

Egger test: t = -2.07, df = 20, p-value = 0.0520

Figure S197.1: Leave-one-out plot: Association between malnutrition-related complication risk and T stage (T4 vs. <T4) in gastric cancer (Biological composite scores)

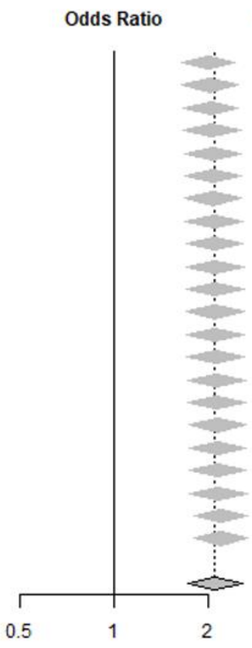

Study	Influental	Effect size	95% CI	I2	Std residual	Dffits	Cook's dist.	Covariance ratio	Hat value
Omitting M. Yamamoto, 2025		2.01	[1.65; 2.44]	0.74	2.00	0.50	0.21	0.93	0.06
Omitting J. Y. Lee, 2016		2.02	[1.64; 2.48]	0.65	1.47	0.42	0.16	1.02	0.07
Omitting K. Fujiya, 2018		2.04	[1.66; 2.50]	0.77	1.35	0.32	0.10	1.02	0.05
Omitting S. Lin, 2025		2.06	[1.67; 2.54]	0.77	0.84	0.21	0.04	1.08	0.06
Omitting B. Akagunduz, 2022		2.06	[1.68; 2.54]	0.77	0.98	0.18	0.03	1.03	0.03
Omitting D. Kuroda, 2018		2.07	[1.68; 2.56]	0.77	0.71	0.14	0.02	1.06	0.04
Omitting I. Tirnova, 2025		2.07	[1.68; 2.55]	0.77	0.88	0.14	0.02	1.04	0.02
Omitting K. Sakurai, 2015		2.08	[1.68; 2.58]	0.77	0.32	0.08	0.01	1.11	0.06
Omitting M. Ishizuka, 2014		2.08	[1.69; 2.56]	0.77	0.72	0.08	0.01	1.04	0.01
Omitting N. Hirahara, 2020		2.10	[1.69; 2.59]	0.77	0.09	0.02	0.00	1.10	0.05
Omitting P. Özcan, 2024		2.10	[1.69; 2.60]	0.77	0.05	0.02	0.00	1.10	0.05
Omitting T. Takahashi, 2020		2.10	[1.70; 2.60]	0.77	-0.14	-0.01	0.00	1.07	0.02
Omitting T. Kosuga, 2019		2.11	[1.70; 2.60]	0.77	-0.15	-0.02	0.00	1.09	0.04
Omitting T. Matsunaga, 2022		2.11	[1.71; 2.61]	0.77	-0.25	-0.05	0.00	1.10	0.05
Omitting H. Jin, 2021		2.12	[1.72; 2.62]	0.77	-0.47	-0.10	0.01	1.09	0.04
Omitting M. Sasahara, 2020		2.13	[1.72; 2.64]	0.76	-0.49	-0.13	0.02	1.11	0.07
Omitting Y. Qian, 2021		2.14	[1.73; 2.64]	0.76	-0.79	-0.18	0.03	1.07	0.05
Omitting M. Kanda, 2016		2.14	[1.73; 2.64]	0.76	-0.83	-0.19	0.04	1.07	0.05
Omitting Z. Luo, 2019		2.14	[1.74; 2.63]	0.77	-1.04	-0.20	0.04	1.03	0.04
Omitting S. Ryo, 2018		2.15	[1.75; 2.66]	0.74	-0.99	-0.26	0.07	1.07	0.06
Omitting X. Zhu, 2021		2.19	[1.80; 2.66]	0.74	-1.99	-0.46	0.18	0.92	0.05
Omitting Z. Xishan, 2020		2.19	[1.81; 2.66]	0.74	-2.10	-0.48	0.20	0.90	0.05
**Random effects model**		**2.10**	**[1.71; 2.57]**						

I^2 = 0% [0%; 46%]

Figure S198: Funnel plot: Association between malnutrition-related complication risk and colorectal tumour location (colon vs. rectum) (Biological composite scores)

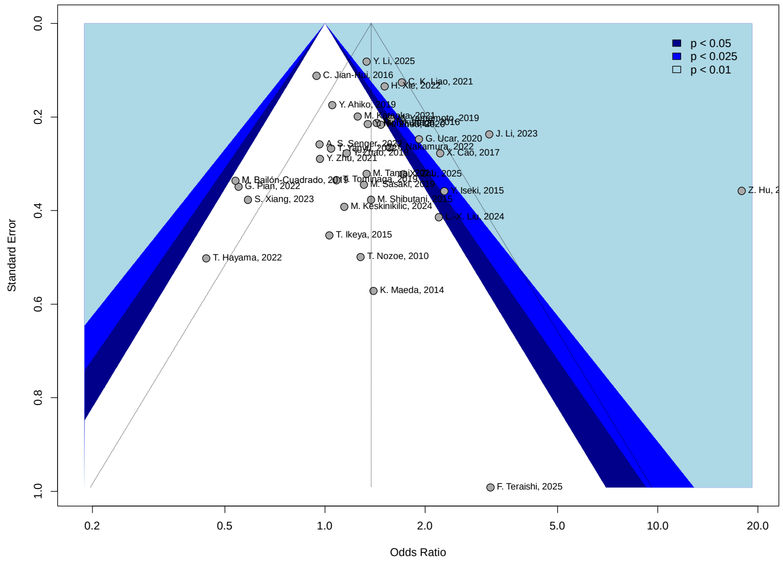

Egger test: t = 0.21, df = 33, p-value = 0.8320

Figure S198.1: Leave-one-out plot: Association between malnutrition-related complication risk and colorectal tumour location (colon vs. rectum) (Biological composite scores)

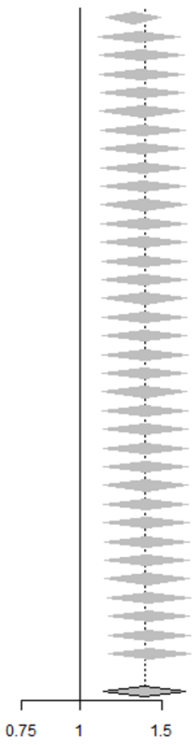

Study	Influental	Effect size	95% CI	I2	Std residual	Dffits	Cook's dist.	Covariance ratio	Hat value
Omitting Z. Hu, 2020	*	1.30	[1.14; 1.49]	0.52	5.78	0.77	0.31	0.45	0.03
Omitting J. Li, 2023		1.34	[1.09; 1.64]	0.70	1.53	0.28	0.07	0.99	0.03
Omitting X. Cao, 2017		1.36	[1.10; 1.67]	0.72	0.84	0.15	0.02	1.04	0.03
Omitting Y. Iseki, 2015		1.36	[1.10; 1.67]	0.72	0.83	0.14	0.02	1.04	0.03
Omitting L.-X. Liu, 2024		1.36	[1.11; 1.68]	0.73	0.72	0.11	0.01	1.04	0.02
Omitting G. Ucar, 2020		1.36	[1.11; 1.68]	0.72	0.59	0.11	0.01	1.06	0.03
Omitting C. K. Liao, 2021		1.37	[1.11; 1.68]	0.72	0.41	0.08	0.01	1.07	0.04
Omitting F. Teraishi, 2025		1.37	[1.11; 1.68]	0.73	0.74	0.07	0.00	1.02	0.01
Omitting X. Zhu, 2025		1.37	[1.11; 1.69]	0.73	0.37	0.06	0.00	1.06	0.03
Omitting M. Yamamoto, 2019		1.37	[1.11; 1.69]	0.73	0.26	0.05	0.00	1.07	0.03
Omitting Y. Nakamura, 2022		1.37	[1.11; 1.69]	0.73	0.22	0.04	0.00	1.07	0.03
Omitting H. Xie, 2022		1.37	[1.11; 1.69]	0.73	0.18	0.04	0.00	1.07	0.04
Omitting H. Zhou, 2020		1.37	[1.11; 1.69]	0.73	0.12	0.02	0.00	1.07	0.03
Omitting R. Tokunaga, 2016		1.37	[1.11; 1.70]	0.73	0.07	0.01	0.00	1.07	0.03
Omitting K. Maeda, 2014		1.38	[1.12; 1.70]	0.73	0.02	0.00	0.00	1.05	0.02
Omitting M. Shibutani, 2015		1.38	[1.12; 1.70]	0.73	-0.00	0.00	0.00	1.06	0.03
Omitting Y. Mohri, 2013		1.38	[1.12; 1.70]	0.73	-0.04	-0.01	0.00	1.07	0.03
Omitting M. Tamai, 2021		1.38	[1.12; 1.70]	0.73	-0.05	-0.01	0.00	1.06	0.03
Omitting Y. Li, 2025		1.38	[1.12; 1.70]	0.73	-0.06	-0.01	0.00	1.08	0.04
Omitting M. Sasaki, 2019		1.38	[1.12; 1.70]	0.73	-0.08	-0.01	0.00	1.06	0.03
Omitting T. Nozoe, 2010		1.38	[1.12; 1.70]	0.73	-0.10	-0.01	0.00	1.06	0.02
Omitting M. Kataoka, 2021		1.38	[1.12; 1.70]	0.73	-0.17	-0.03	0.00	1.07	0.03
Omitting M. Keskinkilic, 2024		1.38	[1.12; 1.70]	0.73	-0.29	-0.05	0.00	1.06	0.02
Omitting Y. Zhao, 2018		1.38	[1.12; 1.71]	0.73	-0.29	-0.05	0.00	1.06	0.03
Omitting T. Ikeya, 2015		1.39	[1.12; 1.71]	0.73	-0.43	-0.06	0.00	1.05	0.02
Omitting T. Tominaga, 2019		1.39	[1.12; 1.71]	0.73	-0.40	-0.07	0.00	1.06	0.03
Omitting T. Yagyu, 2022		1.39	[1.13; 1.71]	0.73	-0.49	-0.09	0.01	1.06	0.03
Omitting Y. Ahiko, 2019		1.39	[1.13; 1.71]	0.72	-0.51	-0.10	0.01	1.06	0.04
Omitting Y. Zhu, 2021		1.39	[1.13; 1.71]	0.73	-0.61	-0.11	0.01	1.05	0.03
Omitting A. S. Senger, 2022		1.39	[1.13; 1.72]	0.72	-0.63	-0.11	0.01	1.05	0.03
Omitting C. Jian-Hui, 2016		1.40	[1.13; 1.72]	0.70	-0.74	-0.15	0.02	1.05	0.04
Omitting S. Xiang, 2023		1.41	[1.15; 1.72]	0.72	-1.39	-0.22	0.05	0.99	0.03
Omitting T. Hayama, 2022		1.41	[1.15; 1.72]	0.72	-1.65	-0.23	0.05	0.96	0.02
Omitting G. Pian, 2022		1.41	[1.15; 1.73]	0.71	-1.55	-0.26	0.06	0.98	0.03
Omitting M. Bailón-Cuadrado, 2019		1.41	[1.16; 1.73]	0.71	-1.61	-0.27	0.07	0.98	0.03
**Random effects model**		**1.38**	**[1.12; 1.69]**						

I^2 = 0% [0%; 38%]

Figure S199: Funnel plot: Association between malnutrition-related complication risk and esophagus tumour location (upper vs. middle or lower part) (Biological composite scores)

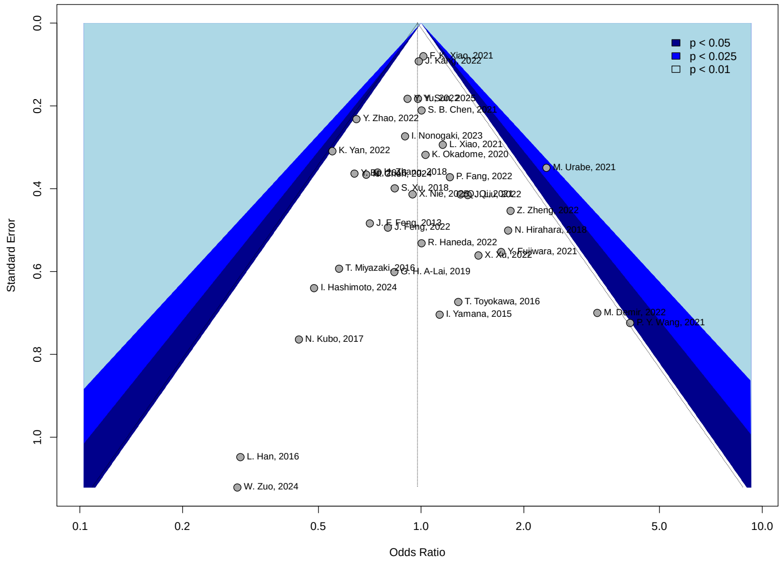

Egger test: t = 0.29, df = 34, p-value = 0.7712

Figure S199.1: Leave-one-out plot: Association between malnutrition-related complication risk and esophagus tumour location (upper vs. middle or lower part) (Biological composite scores)

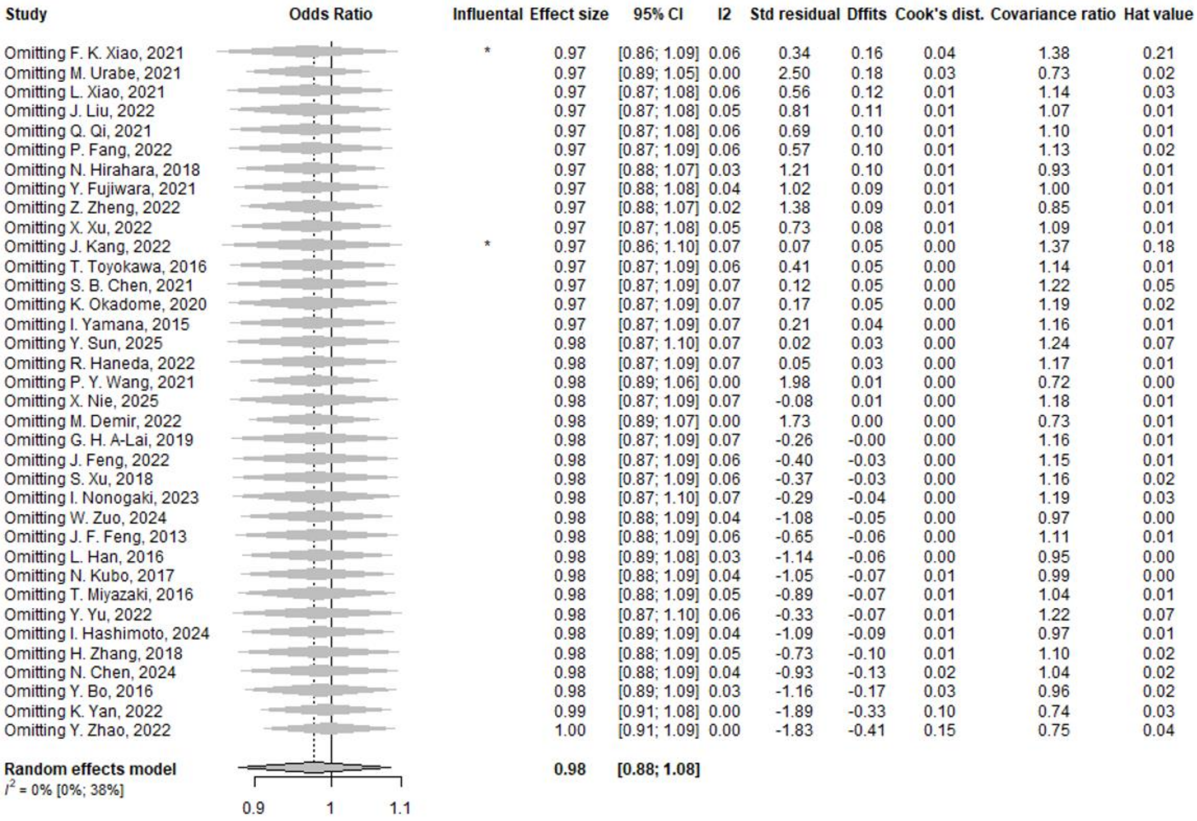

Figure S200: Funnel plot: Association between malnutrition-related complication risk and gastric tumour location (upper vs. middle or lower part) (Biological composite scores)

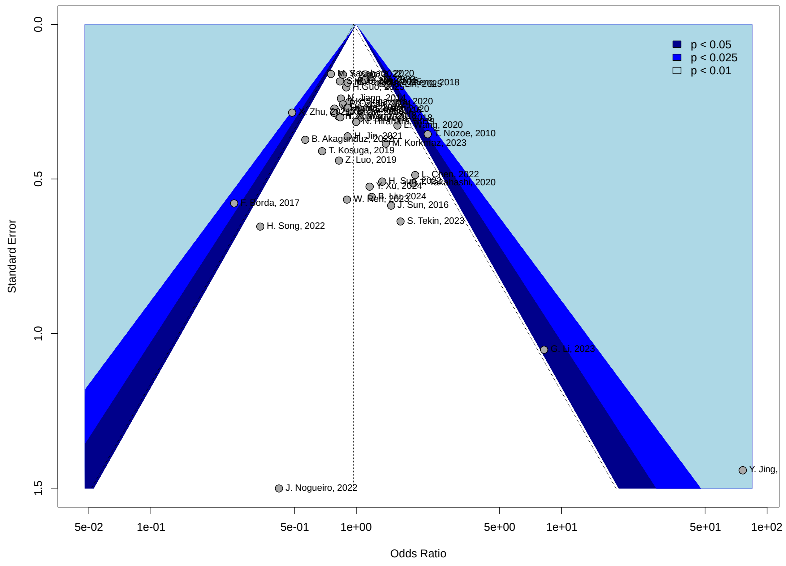

Egger test: t = -0.01, df = 40, p-value = 0.9907

Figure S200.1: Leave-one-out plot: Association between malnutrition-related complication risk and gastric tumour location (upper vs. middle or lower part) (Biological composite scores)

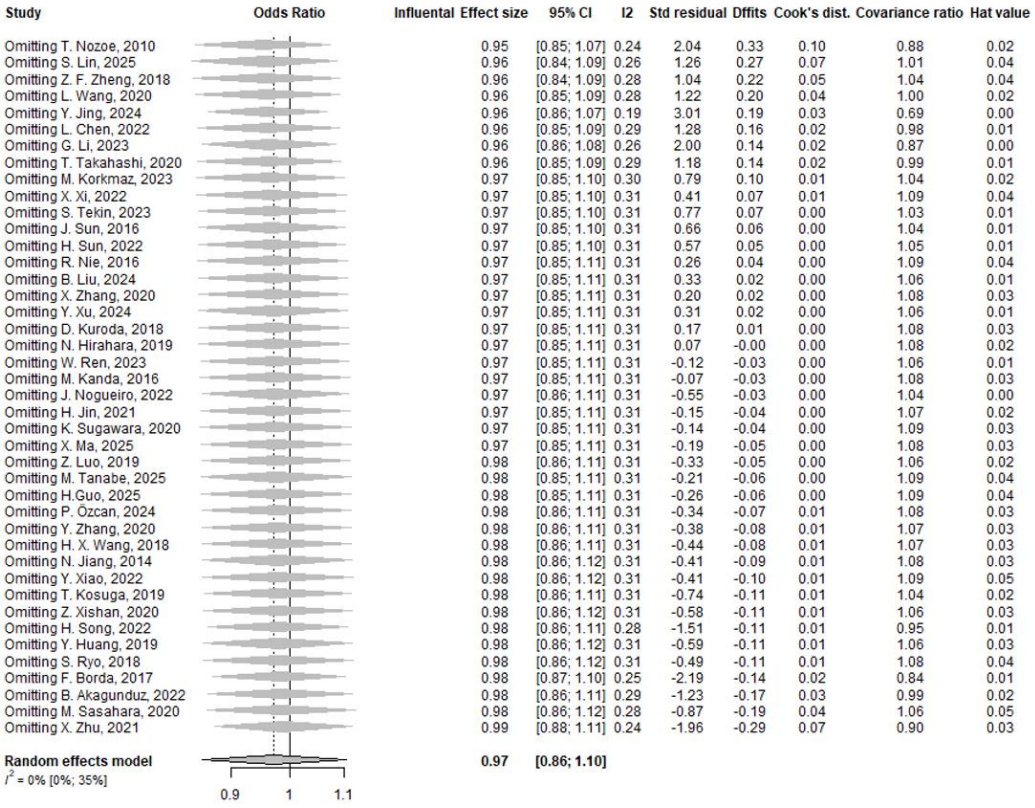

Figure S201: Funnel plot: Association between malnutrition-related complication risk and macrovascular invasion in gastric cancer (Biological composite scores)

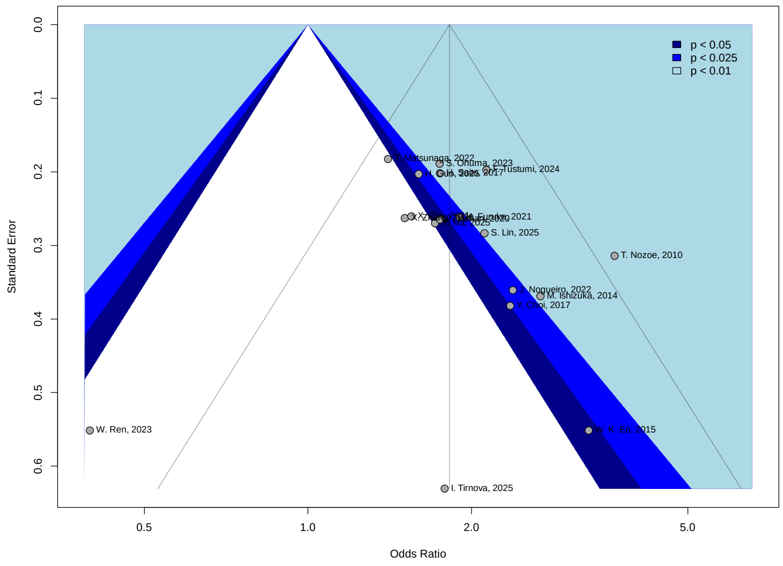

Egger test: t = -0.36, df = 16, p-value = 0.7201

Figure S201.1: Leave-one-out plot: Association between malnutrition-related complication risk and macrovascular invasion in gastric cancer (Biological composite scores)

Study	Odds Ratio	Influental	Effect size	95% CI	I2	Std residual	Dffits	Cook's dist.	Covariance ratio	Hat value
Omitting T. Nozoe, 2010			1.75	[1.54; 2.00]	0.00	2.30	0.58	0.28	0.74	0.05
Omitting M. Ishizuka, 2014			1.80	[1.53; 2.11]	0.16	0.99	0.19	0.03	1.04	0.03
Omitting F. Tustumi, 2024			1.80	[1.52; 2.13]	0.18	0.63	0.17	0.03	1.17	0.09
Omitting W. K. Eo, 2015			1.80	[1.54; 2.11]	0.16	1.04	0.14	0.02	1.01	0.02
Omitting J. Nogueiro, 2022			1.81	[1.53; 2.13]	0.19	0.69	0.12	0.01	1.10	0.04
Omitting Y. Choi, 2017			1.81	[1.53; 2.13]	0.19	0.62	0.10	0.01	1.11	0.03
Omitting S. Lin, 2025			1.81	[1.53; 2.14]	0.20	0.45	0.08	0.01	1.15	0.05
Omitting H. Furuke, 2021			1.82	[1.54; 2.16]	0.21	0.13	-0.00	0.00	1.19	0.06
Omitting I. Tirnova, 2025			1.83	[1.55; 2.16]	0.21	-0.04	-0.04	0.00	1.13	0.01
Omitting Z. Xishan, 2020			1.83	[1.54; 2.17]	0.21	-0.14	-0.07	0.01	1.19	0.06
Omitting X. Ma, 2025			1.83	[1.55; 2.17]	0.21	-0.20	-0.08	0.01	1.18	0.06
Omitting H. Saito, 2017			1.83	[1.54; 2.18]	0.21	-0.17	-0.08	0.01	1.22	0.09
Omitting S. Onuma, 2023			1.83	[1.54; 2.18]	0.21	-0.18	-0.09	0.01	1.22	0.09
Omitting W. Ren, 2023			1.84	[1.63; 2.07]	0.00	-2.76	-0.14	0.02	0.72	0.02
Omitting X. Zhu, 2021			1.84	[1.56; 2.18]	0.20	-0.54	-0.16	0.03	1.15	0.06
Omitting X. Zhang, 2020			1.85	[1.56; 2.18]	0.19	-0.64	-0.18	0.04	1.14	0.06
Omitting H. Guo, 2025			1.85	[1.56; 2.19]	0.19	-0.52	-0.18	0.04	1.18	0.09
Omitting T. Matsunaga, 2022			1.87	[1.59; 2.20]	0.12	-1.20	-0.39	0.14	1.06	0.10
**Random effects model**			**1.82**	**[1.56; 2.13]**						

I^2 = 0% [0%; 50%]

0.5 1 2

Figure S202: Funnel plot: Association between malnutrition-related complication risk and age ≥65 in gastric cancer (Biological composite scores)

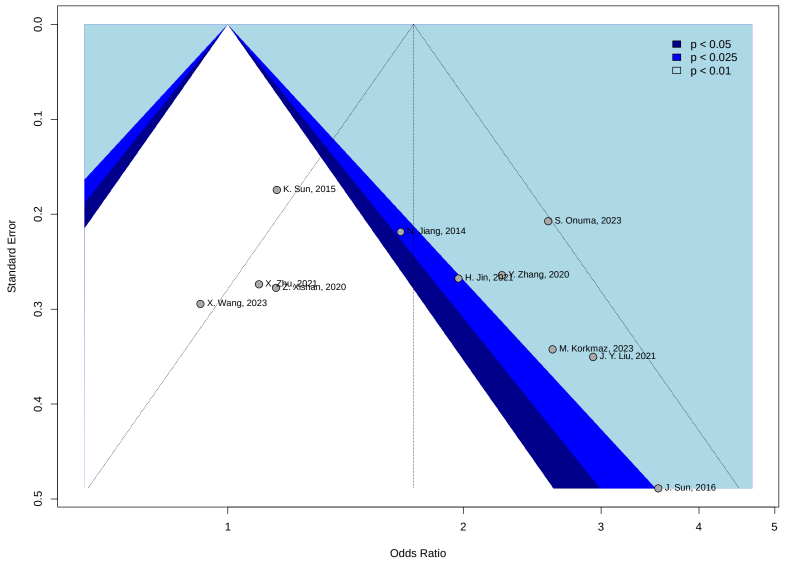

Egger test: t = 0.86, df = 9, p-value = 0.4125

Figure S202.1: Leave-one-out plot: Association between malnutrition-related complication risk and age ≥65 in gastric cancer (Biological composite scores)

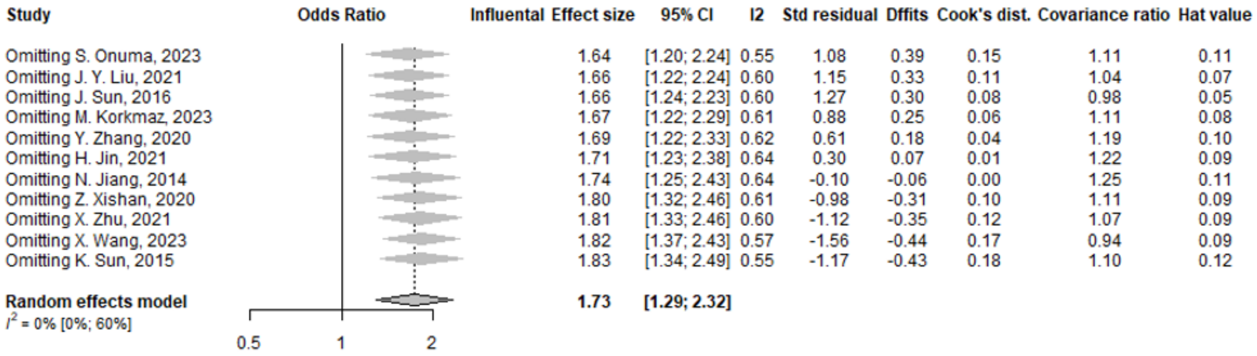

Figure S203: Funnel plot: Association between malnutrition-related complication risk and age ≥65 in esophageal cancer (Biological composite scores)

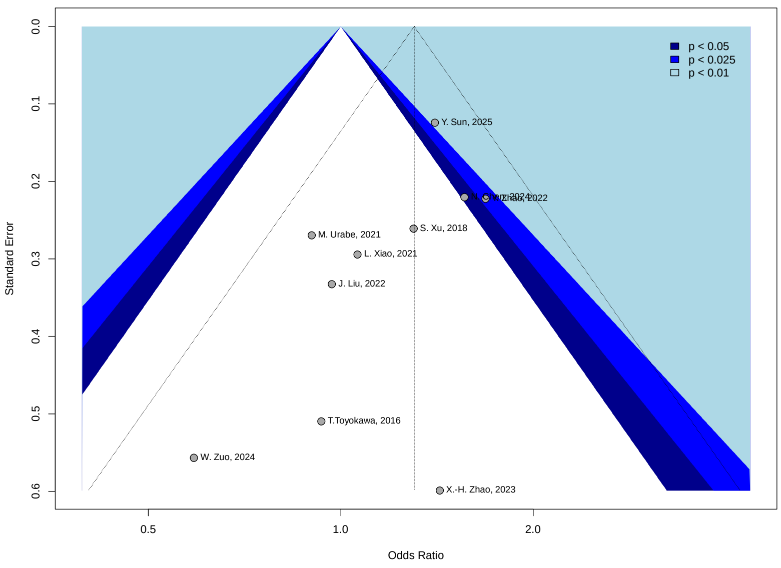

Egger test:t = -1.76, df = 8, p-value = 0.1167

Figure S203.1: Leave-one-out plot: Association between malnutrition-related complication risk and age ≥65 in esophageal cancer (Biological composite scores)

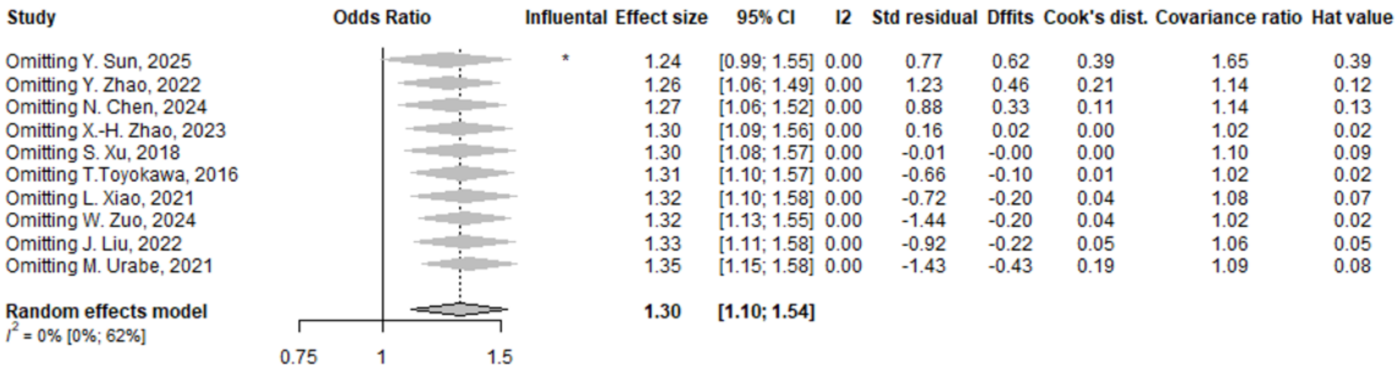

Figure S204: Funnel plot: Association between malnutrition-related complication risk and age ≥65 in colorectal cancer (Biological composite scores)

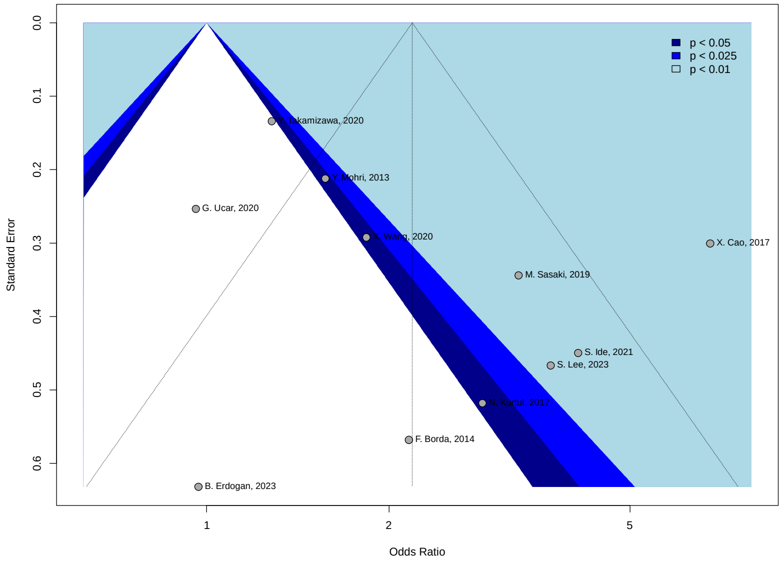

Egger test: t = 1.31, df = 9, p-value = 0.2221

Figure S204.1: Leave-one-out plot: Association between malnutrition-related complication risk and age ≥65 in colorectal cancer (Biological composite scores)

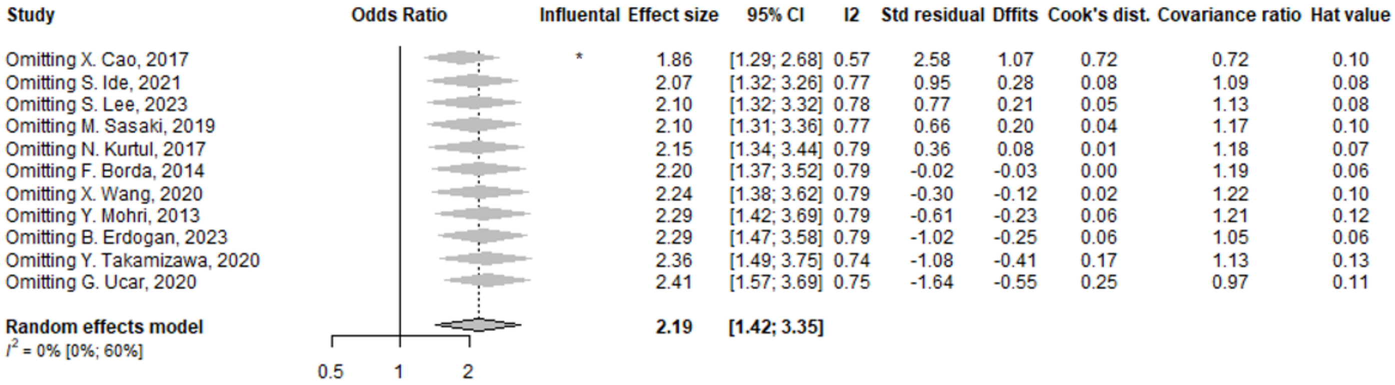

Figure S205: Funnel plot: Association between malnutrition-related complication risk and age ≥60 in gastric cancer (Biological composite scores)

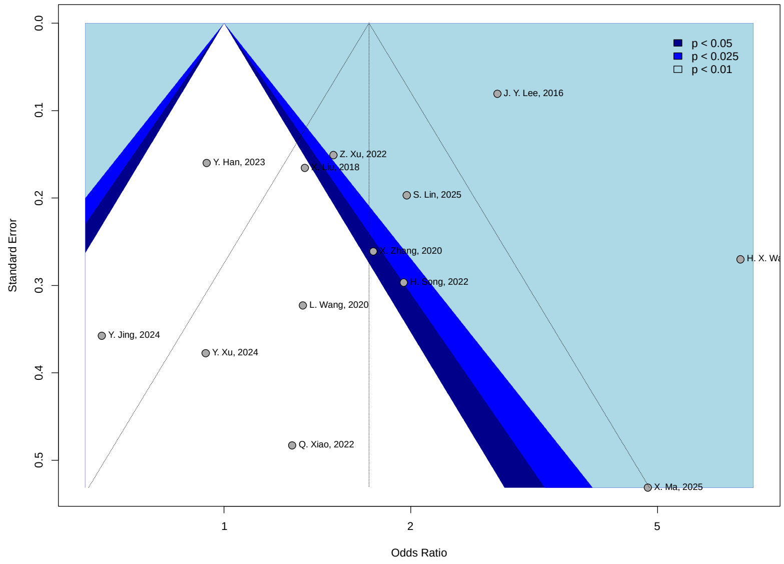

Egger test:t = -0.87, df = 11, p-value = 0.4049

Figure S205.1: Leave-one-out plot: Association between malnutrition-related complication risk and age ≥60 in gastric cancer (Biological composite scores)

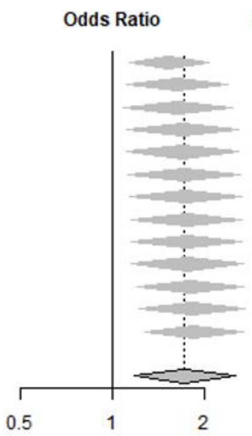

Study	Influental	Effect size	95% CI	I2	Std residual	Dffits	Cook's dist.	Covariance ratio	Hat value
Omitting H. X. Wang, 2018		1.53	[1.13; 2.07]	0.83	3.02	0.86	0.42	0.62	0.08
Omitting X. Ma, 2025		1.62	[1.11; 2.37]	0.87	1.41	0.32	0.10	0.97	0.05
Omitting J. Y. Lee, 2016		1.63	[1.08; 2.46]	0.80	0.87	0.28	0.08	1.13	0.09
Omitting S. Lin, 2025		1.69	[1.11; 2.58]	0.87	0.23	0.07	0.01	1.19	0.09
Omitting H. Song, 2022		1.69	[1.11; 2.58]	0.87	0.20	0.06	0.00	1.18	0.08
Omitting X. Zhang, 2020		1.71	[1.12; 2.61]	0.87	0.03	0.01	0.00	1.19	0.08
Omitting Z. Xu, 2022		1.74	[1.14; 2.65]	0.87	-0.23	-0.07	0.01	1.20	0.09
Omitting Q. Xiao, 2022		1.74	[1.15; 2.64]	0.87	-0.38	-0.10	0.01	1.15	0.06
Omitting L. Wang, 2020		1.75	[1.15; 2.65]	0.87	-0.38	-0.11	0.01	1.17	0.07
Omitting X. Liu, 2018		1.75	[1.15; 2.67]	0.87	-0.41	-0.13	0.02	1.18	0.09
Omitting Y. Xu, 2024		1.79	[1.20; 2.68]	0.87	-0.92	-0.25	0.06	1.09	0.07
Omitting Y. Han, 2023		1.82	[1.22; 2.72]	0.83	-1.09	-0.34	0.11	1.08	0.09
Omitting Y. Jing, 2024		1.84	[1.27; 2.67]	0.86	-1.63	-0.44	0.17	0.93	0.07
**Random effects model**		**1.71**	**[1.17; 2.51]**						

I^2 = 0% [0%; 57%]

Figure S206: Funnel plot: Association between malnutrition risk and sex in esophageal cancer (Symptom-based risk assessment tools)

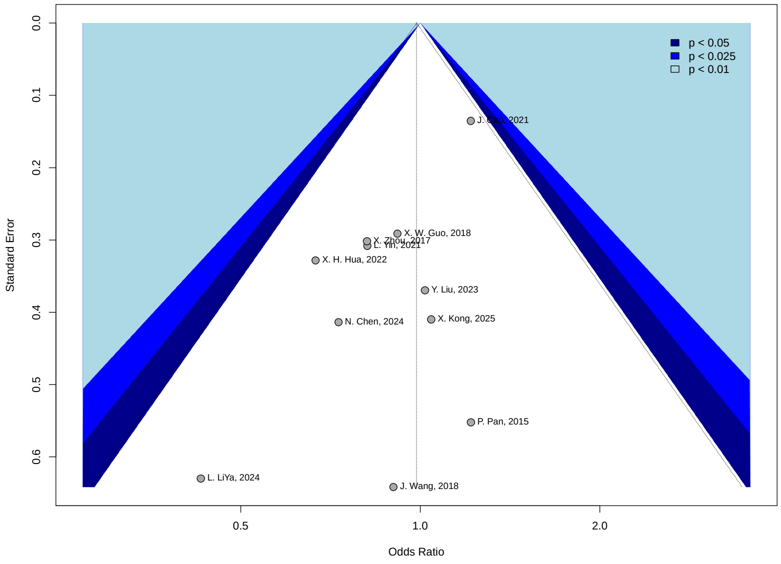

Egger test: t = -2.67, df = 9, p-value = 0.0254

Figure S207 Funnel plot: Association between malnutrition risk and sex in gastric cancer (Symptom-based risk assessment tools)

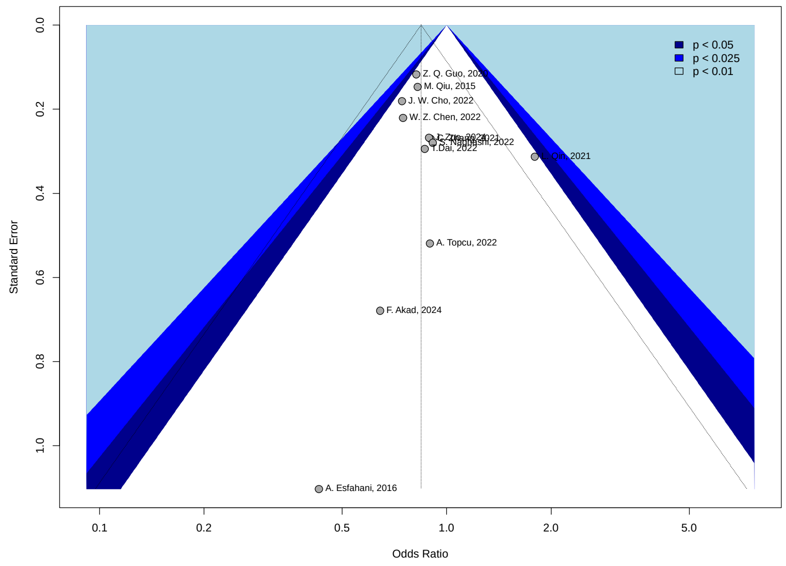

Egger test: t = 0.28, df = 10, p-value = 0.7876

Figure S207.1 Leave-one-out plot: Association between malnutrition risk and sex in gastric cancer (Symptom-based risk assessment tools)

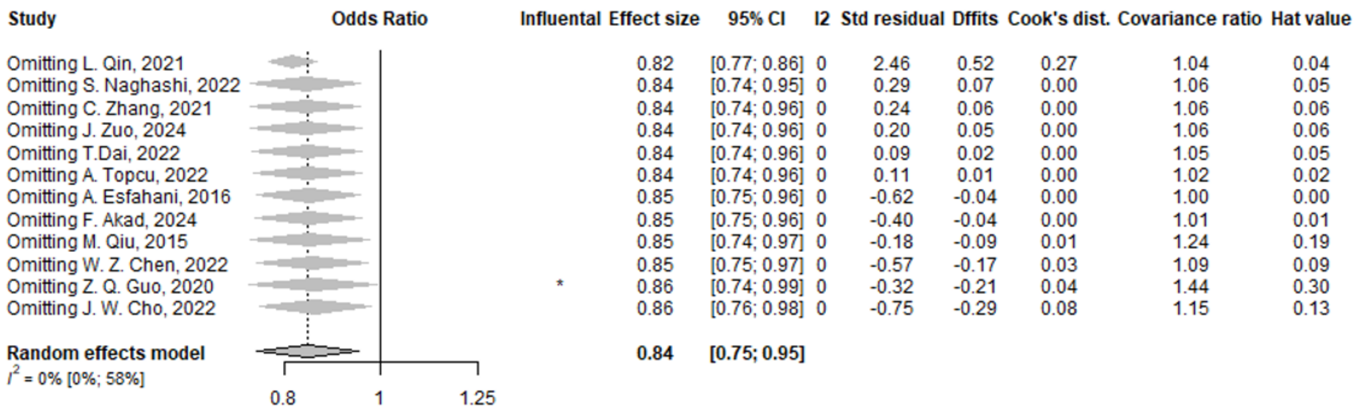

Figure S208: Funnel plot: Association between cachexia and sex in gastric cancer

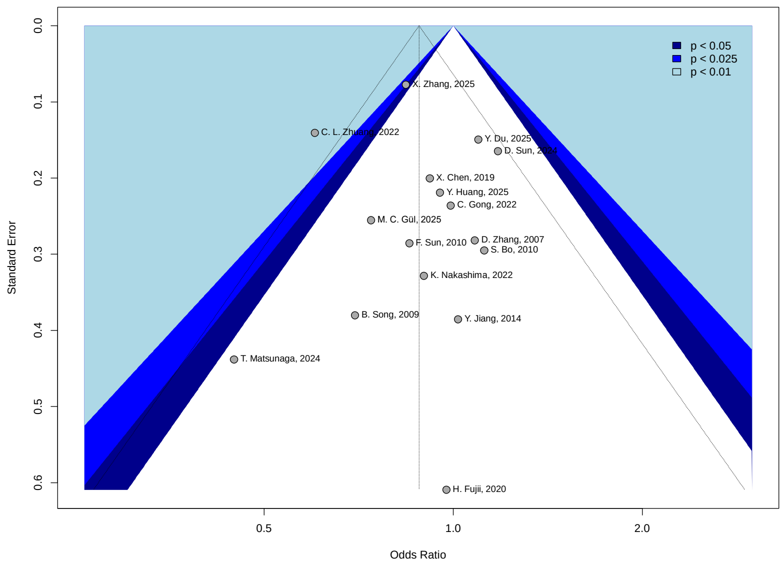

Egger test:t = 0.01, df = 14, p-value = 0.9939

Figure S208.1 Leave-one-out plot: Association between cachexia and sex in gastric cancer

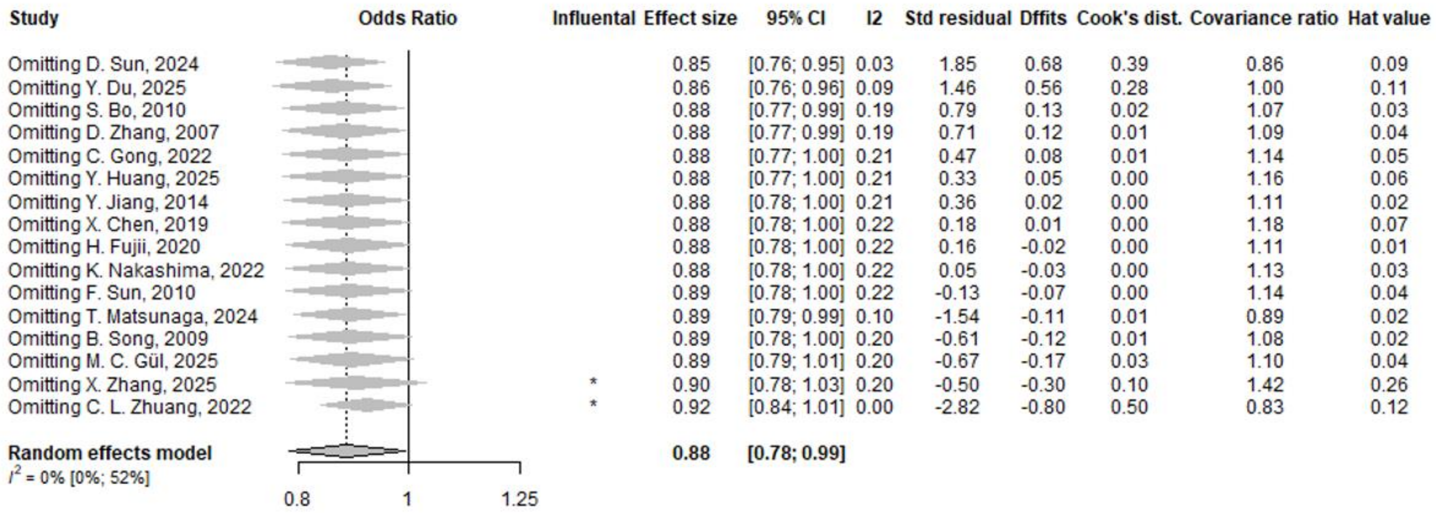

Study	Influental	Effect size	95% CI	I2	Std residual	Dffits	Cook's dist.	Covariance ratio	Hat value
Omitting D. Sun, 2024		0.85	[0.76; 0.95]	0.03	1.85	0.68	0.39	0.86	0.09
Omitting Y. Du, 2025		0.86	[0.76; 0.96]	0.09	1.46	0.56	0.28	1.00	0.11
Omitting S. Bo, 2010		0.88	[0.77; 0.99]	0.19	0.79	0.13	0.02	1.07	0.03
Omitting D. Zhang, 2007		0.88	[0.77; 0.99]	0.19	0.71	0.12	0.01	1.09	0.04
Omitting C. Gong, 2022		0.88	[0.77; 1.00]	0.21	0.47	0.08	0.01	1.14	0.05
Omitting Y. Huang, 2025		0.88	[0.77; 1.00]	0.21	0.33	0.05	0.00	1.16	0.06
Omitting Y. Jiang, 2014		0.88	[0.78; 1.00]	0.21	0.36	0.02	0.00	1.11	0.02
Omitting X. Chen, 2019		0.88	[0.78; 1.00]	0.22	0.18	0.01	0.00	1.18	0.07
Omitting H. Fujii, 2020		0.88	[0.78; 1.00]	0.22	0.16	-0.02	0.00	1.11	0.01
Omitting K. Nakashima, 2022		0.88	[0.78; 1.00]	0.22	0.05	-0.03	0.00	1.13	0.03
Omitting F. Sun, 2010		0.89	[0.78; 1.00]	0.22	-0.13	-0.07	0.00	1.14	0.04
Omitting T. Matsunaga, 2024		0.89	[0.79; 0.99]	0.10	-1.54	-0.11	0.01	0.89	0.02
Omitting B. Song, 2009		0.89	[0.78; 1.00]	0.20	-0.61	-0.12	0.01	1.08	0.02
Omitting M. C. Gül, 2025		0.89	[0.79; 1.01]	0.20	-0.67	-0.17	0.03	1.10	0.04
Omitting X. Zhang, 2025	*	0.90	[0.78; 1.03]	0.20	-0.50	-0.30	0.10	1.42	0.26
Omitting C. L. Zhuang, 2022	*	0.92	[0.84; 1.01]	0.00	-2.82	-0.80	0.50	0.83	0.12
**Random effects model**		**0.88**	**[0.78; 0.99]**						

I^2 = 0% [0%; 52%]

Figure S209. Funnel plot: Association between cachexia and sex in colorectal cancer

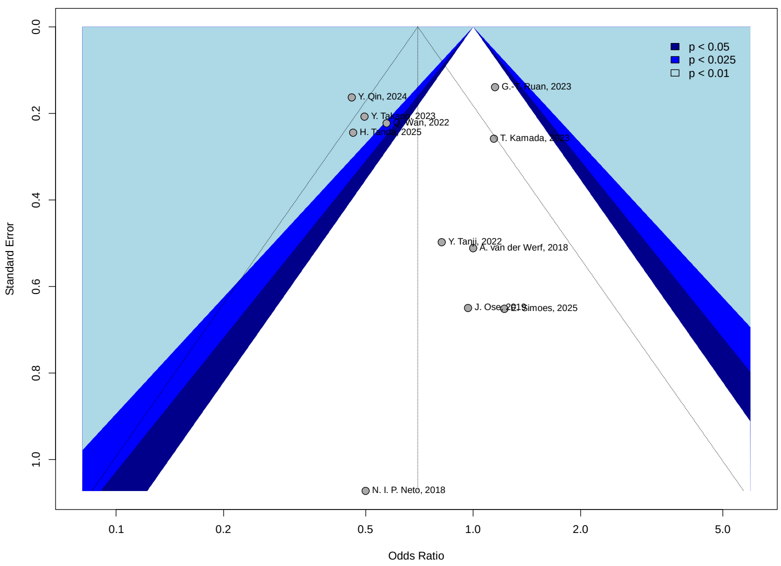

Egger test: t = 0.18, df = 9, p-value = 0.8614

Figure S209.1. Leave-one-out plot: Association between cachexia and sex in colorectal cancer

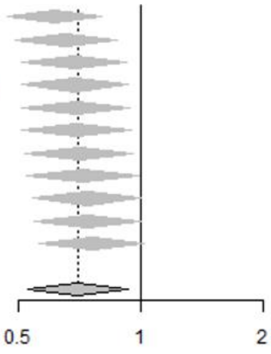

Study	Influental	Effect size	95% CI	I2	Std residual	Dffits	Cook's dist.	Covariance ratio	Hat value
Omitting G.-T. Ruan, 2023	*	0.61	[0.47; 0.80]	0.36	2.33	1.31	1.05	0.88	0.17
Omitting T. Kamada, 2023		0.66	[0.49; 0.88]	0.67	1.45	0.53	0.26	1.02	0.11
Omitting E. Simoes, 2025		0.69	[0.51; 0.93]	0.71	0.80	0.14	0.02	1.07	0.03
Omitting A. van der Werf, 2018		0.69	[0.51; 0.93]	0.71	0.62	0.13	0.02	1.12	0.05
Omitting J. Ose, 2019		0.69	[0.51; 0.94]	0.71	0.46	0.07	0.01	1.12	0.03
Omitting Y. Tanji, 2022		0.70	[0.51; 0.95]	0.71	0.27	0.04	0.00	1.16	0.05
Omitting N. I. P. Neto, 2018		0.70	[0.52; 0.95]	0.71	-0.31	-0.06	0.00	1.11	0.01
Omitting Q. Wan, 2022		0.72	[0.53; 0.99]	0.70	-0.59	-0.24	0.06	1.22	0.13
Omitting Y. Takano, 2023		0.74	[0.54; 1.00]	0.68	-1.08	-0.42	0.18	1.14	0.14
Omitting H. Tanda, 2025		0.74	[0.55; 1.00]	0.68	-1.23	-0.44	0.18	1.08	0.12
Omitting Y. Qin, 2024		0.76	[0.56; 1.02]	0.60	-1.56	-0.68	0.39	1.05	0.16
**Random effects model**		**0.70**	**[0.53; 0.93]**						

I^2 = 0% [0%; 60%]

Figure S210 Funnel plot: Association between malnutrition diagnosis and sex in resectable gastrointestinal cancer (GLIM criteria)

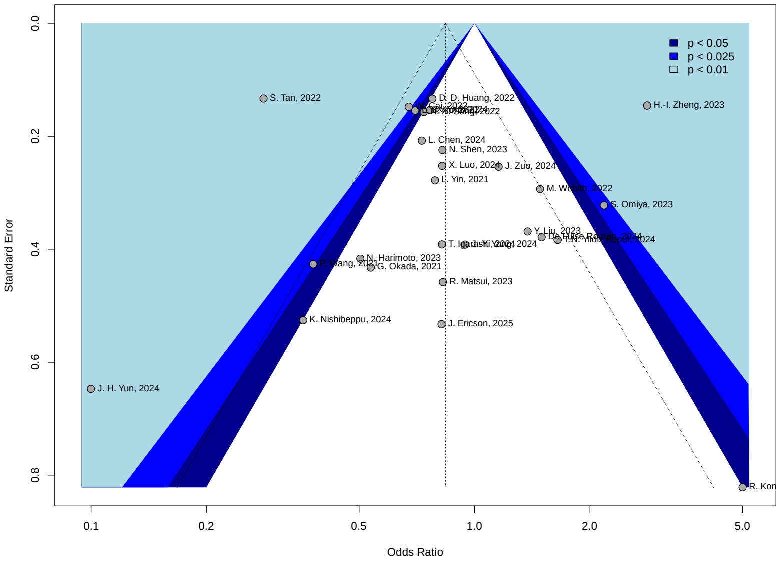

Egger test: t = 0.19, df = 25, p-value = 0.8500

Figure S210.1 Leave-one-out plot: Association between malnutrition diagnosis and sex in resectable gastrointestinal cancer (GLIM criteria)

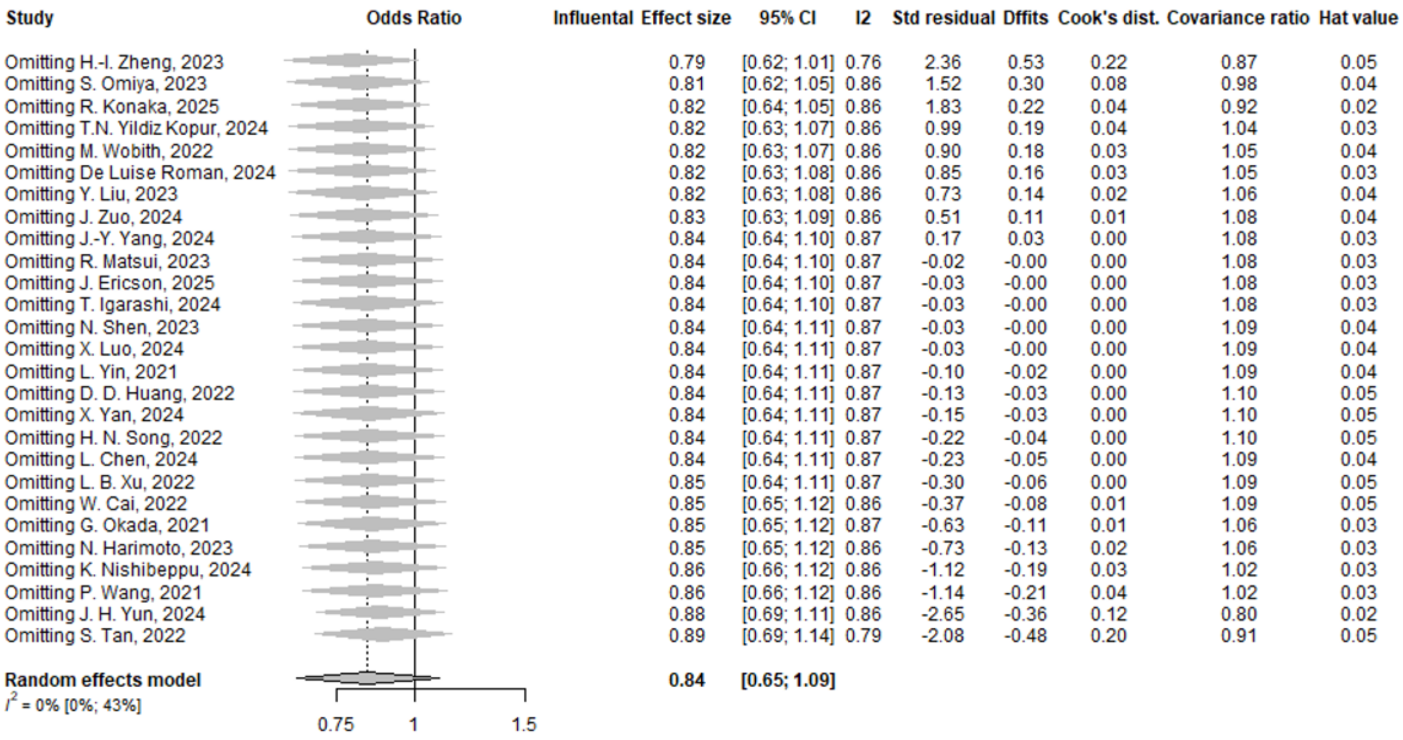

Figure S211 Funnel plot: Association between malnutrition diagnosis and sex in gastric cancer (GLIM criteria)

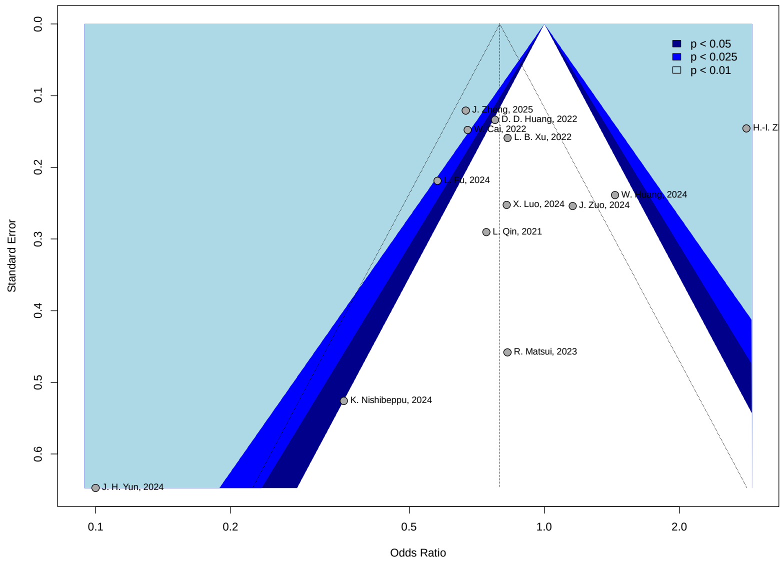

Egger test: t = -1.21, df = 11, p-value = 0.2529

Figure S211.1 Leave-one-out plot: Association between malnutrition diagnosis and sex in gastric cancer (GLIM criteria)

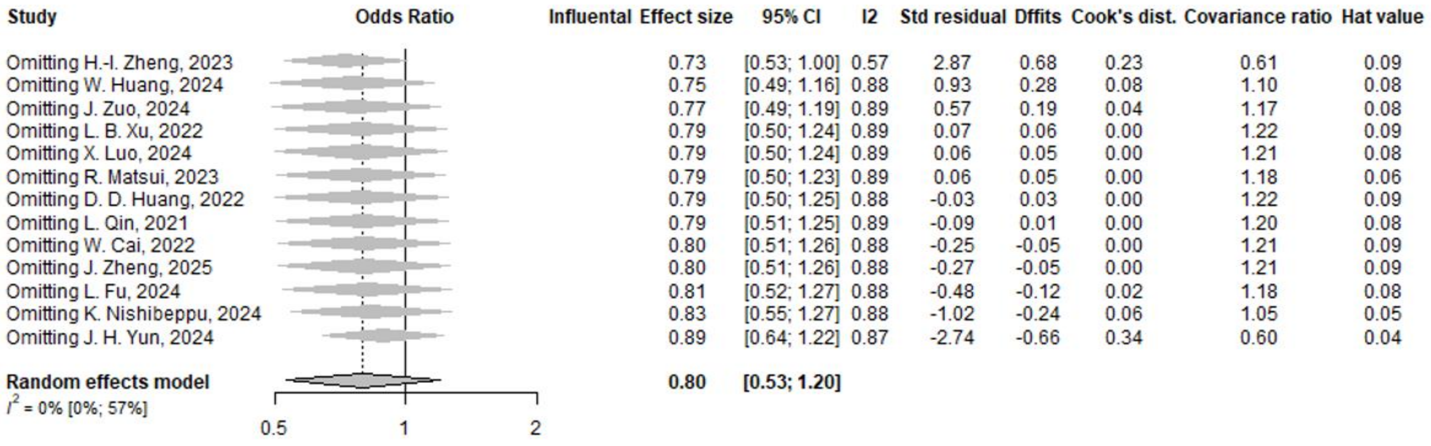

Study	Influental	Effect size	95% CI	I2	Std residual	Dffits	Cook's dist.	Covariance ratio	Hat value
Omitting H.-I. Zheng, 2023		0.73	[0.53; 1.00]	0.57	2.87	0.68	0.23	0.61	0.09
Omitting W. Huang, 2024		0.75	[0.49; 1.16]	0.88	0.93	0.28	0.08	1.10	0.08
Omitting J. Zuo, 2024		0.77	[0.49; 1.19]	0.89	0.57	0.19	0.04	1.17	0.08
Omitting L. B. Xu, 2022		0.79	[0.50; 1.24]	0.89	0.07	0.06	0.00	1.22	0.09
Omitting X. Luo, 2024		0.79	[0.50; 1.24]	0.89	0.06	0.05	0.00	1.21	0.08
Omitting R. Matsui, 2023		0.79	[0.50; 1.23]	0.89	0.06	0.05	0.00	1.18	0.06
Omitting D. D. Huang, 2022		0.79	[0.50; 1.25]	0.88	-0.03	0.03	0.00	1.22	0.09
Omitting L. Qin, 2021		0.79	[0.51; 1.25]	0.89	-0.09	0.01	0.00	1.20	0.08
Omitting W. Cai, 2022		0.80	[0.51; 1.26]	0.88	-0.25	-0.05	0.00	1.21	0.09
Omitting J. Zheng, 2025		0.80	[0.51; 1.26]	0.88	-0.27	-0.05	0.00	1.21	0.09
Omitting L. Fu, 2024		0.81	[0.52; 1.27]	0.88	-0.48	-0.12	0.02	1.18	0.08
Omitting K. Nishibeppu, 2024		0.83	[0.55; 1.27]	0.88	-1.02	-0.24	0.06	1.05	0.05
Omitting J. H. Yun, 2024		0.89	[0.64; 1.22]	0.87	-2.74	-0.66	0.34	0.60	0.04
**Random effects model**		**0.80**	**[0.53; 1.20]**						

I^2 = 0% [0%; 57%]

Figure S212. Funnel plot: Association between malnutrition-related complication risk and smoking (active or history) in gastric cancer (Biological composite scores)

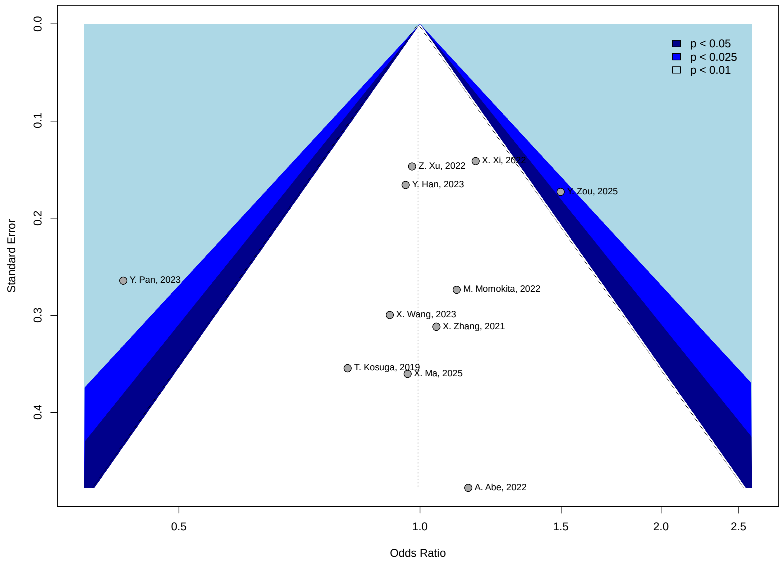

Egger test: t = -0.98, df = 9, p-value = 0.3513

Figure S212.1 Leave-one-out plot: Association between malnutrition-related complication risk and smoking (active or history) in gastric cancer (Biological composite scores)

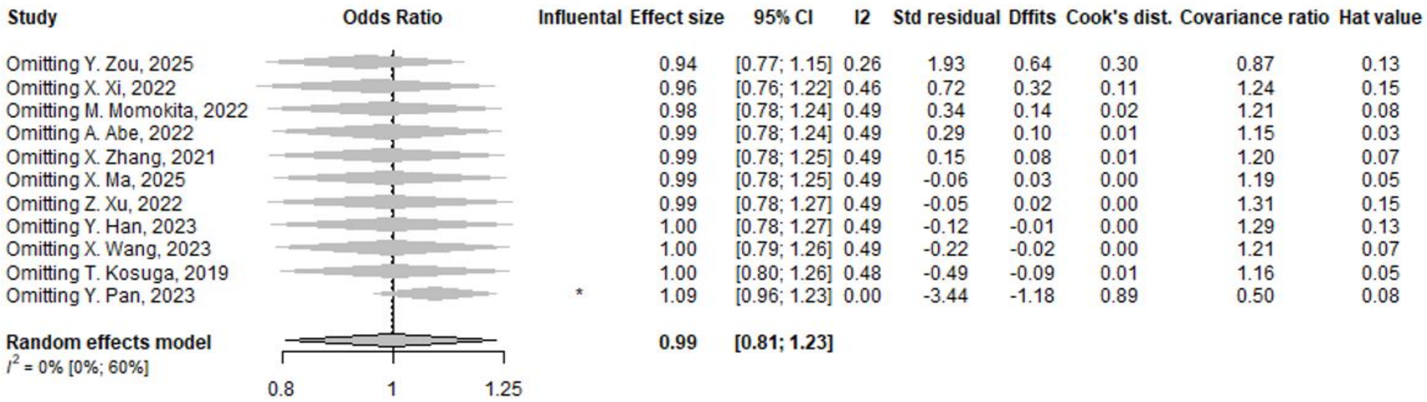

Study	Influential	Effect size	95% CI	I2	Std residual	Dffits	Cook's dist.	Covariance ratio	Hat value
Omitting Y. Zou, 2025		0.94	[0.77; 1.15]	0.26	1.93	0.64	0.30	0.87	0.13
Omitting X. Xi, 2022		0.96	[0.76; 1.22]	0.46	0.72	0.32	0.11	1.24	0.15
Omitting M. Momokita, 2022		0.98	[0.78; 1.24]	0.49	0.34	0.14	0.02	1.21	0.08
Omitting A. Abe, 2022		0.99	[0.78; 1.24]	0.49	0.29	0.10	0.01	1.15	0.03
Omitting X. Zhang, 2021		0.99	[0.78; 1.25]	0.49	0.15	0.08	0.01	1.20	0.07
Omitting X. Ma, 2025		0.99	[0.78; 1.25]	0.49	-0.06	0.03	0.00	1.19	0.05
Omitting Z. Xu, 2022		0.99	[0.78; 1.27]	0.49	-0.05	0.02	0.00	1.31	0.15
Omitting Y. Han, 2023		1.00	[0.78; 1.27]	0.49	-0.12	-0.01	0.00	1.29	0.13
Omitting X. Wang, 2023		1.00	[0.79; 1.26]	0.49	-0.22	-0.02	0.00	1.21	0.07
Omitting T. Kosuga, 2019		1.00	[0.80; 1.26]	0.48	-0.49	-0.09	0.01	1.16	0.05
Omitting Y. Pan, 2023	*	1.09	[0.96; 1.23]	0.00	-3.44	-1.18	0.89	0.50	0.08
**Random effects model**		**0.99**	**[0.81; 1.23]**						

I^2 = 0% [0%; 60%]

Figure S213: Funnel plot: Association between malnutrition-related complication risk and active alcohol consumption in gastrointestinal cancer (Biological composite scores)

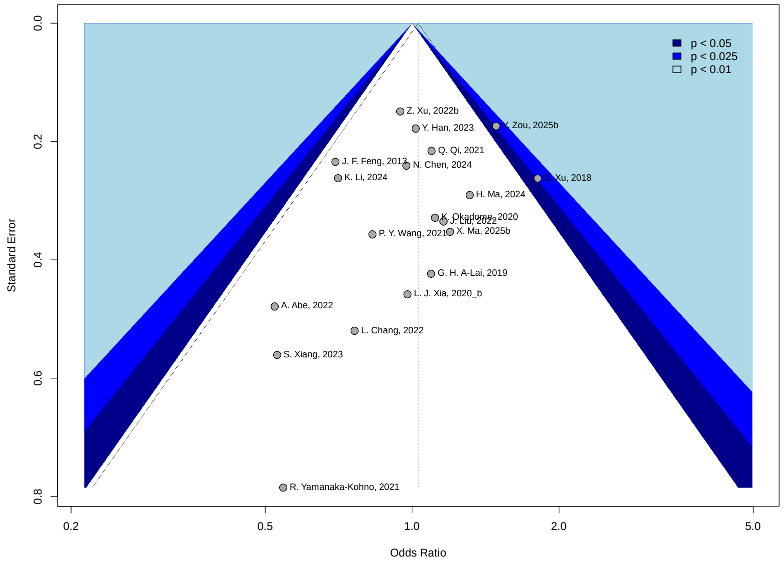

Egger test: t = -1.00, df = 17, p-value = 0.3302

Figure S213.1 Leave-one-out plot: Association between malnutrition-related complication risk and active alcohol consumption in gastrointestinal cancer (Biological composite scores)

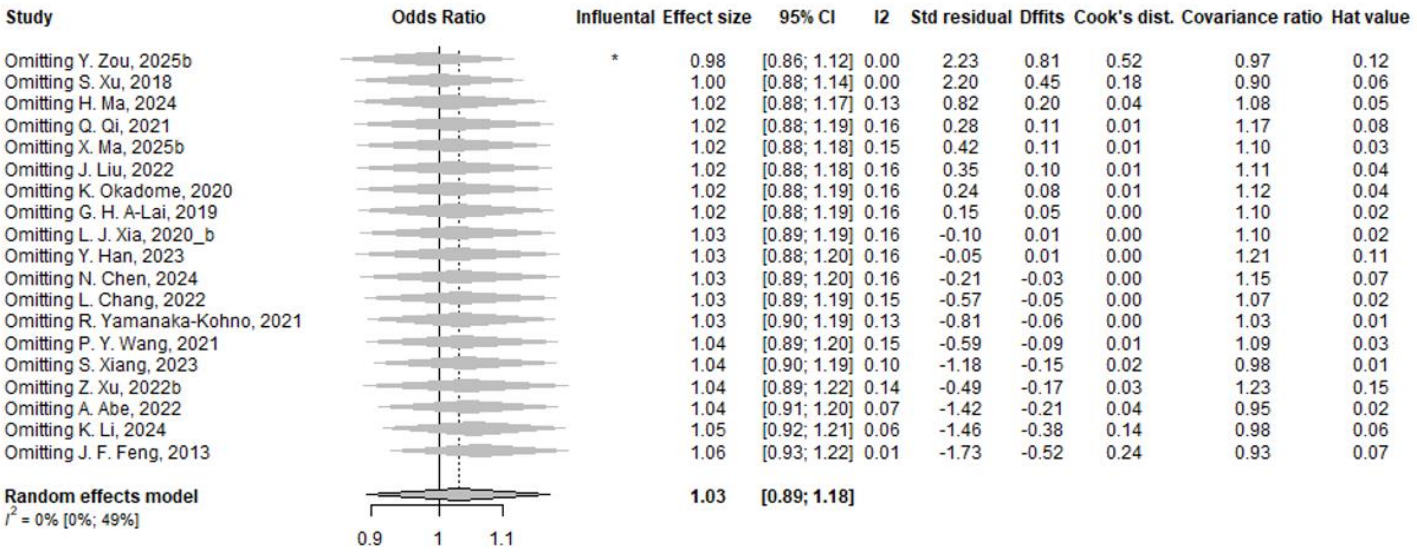

Study	Influental	Effect size	95% CI	I2	Std residual	Dffits	Cook's dist.	Covariance ratio	Hat value
Omitting Y. Zou, 2025b	*	0.98	[0.86; 1.12]	0.00	2.23	0.81	0.52	0.97	0.12
Omitting S. Xu, 2018		1.00	[0.88; 1.14]	0.00	2.20	0.45	0.18	0.90	0.06
Omitting H. Ma, 2024		1.02	[0.88; 1.17]	0.13	0.82	0.20	0.04	1.08	0.05
Omitting Q. Qi, 2021		1.02	[0.88; 1.19]	0.16	0.28	0.11	0.01	1.17	0.08
Omitting X. Ma, 2025b		1.02	[0.88; 1.18]	0.15	0.42	0.11	0.01	1.10	0.03
Omitting J. Liu, 2022		1.02	[0.88; 1.18]	0.16	0.35	0.10	0.01	1.11	0.04
Omitting K. Okadome, 2020		1.02	[0.88; 1.19]	0.16	0.24	0.08	0.01	1.12	0.04
Omitting G. H. A-Lai, 2019		1.02	[0.88; 1.19]	0.16	0.15	0.05	0.00	1.10	0.02
Omitting L. J. Xia, 2020_b		1.03	[0.89; 1.19]	0.16	-0.10	0.01	0.00	1.10	0.02
Omitting Y. Han, 2023		1.03	[0.88; 1.20]	0.16	-0.05	0.01	0.00	1.21	0.11
Omitting N. Chen, 2024		1.03	[0.89; 1.20]	0.16	-0.21	-0.03	0.00	1.15	0.07
Omitting L. Chang, 2022		1.03	[0.89; 1.19]	0.15	-0.57	-0.05	0.00	1.07	0.02
Omitting R. Yamanaka-Kohno, 2021		1.03	[0.90; 1.19]	0.13	-0.81	-0.06	0.00	1.03	0.01
Omitting P. Y. Wang, 2021		1.04	[0.89; 1.20]	0.15	-0.59	-0.09	0.01	1.09	0.03
Omitting S. Xiang, 2023		1.04	[0.90; 1.19]	0.10	-1.18	-0.15	0.02	0.98	0.01
Omitting Z. Xu, 2022b		1.04	[0.89; 1.22]	0.14	-0.49	-0.17	0.03	1.23	0.15
Omitting A. Abe, 2022		1.04	[0.91; 1.20]	0.07	-1.42	-0.21	0.04	0.95	0.02
Omitting K. Li, 2024		1.05	[0.92; 1.21]	0.06	-1.46	-0.38	0.14	0.98	0.06
Omitting J. F. Feng, 2013		1.06	[0.93; 1.22]	0.01	-1.73	-0.52	0.24	0.93	0.07
**Random effects model**		**1.03**	**[0.89; 1.18]**						
I^2 = 0% [0%; 49%]									

Figure S214 Funnel plot: Association between malnutrition-related complication risk and comorbidities in upper gastrointestinal cancer (Biological composite scores)

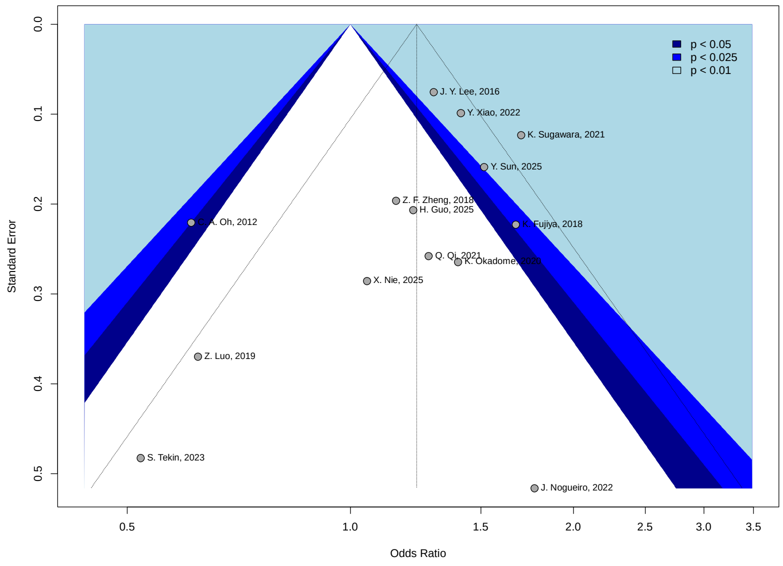

Egger test: t = -2.04, df = 12, p-value = 0.0637

Figure S214.1 Leave-one-out plot Association between malnutrition-related complication risk and comorbidities in upper gastrointestinal cancer (Biological composite scores)

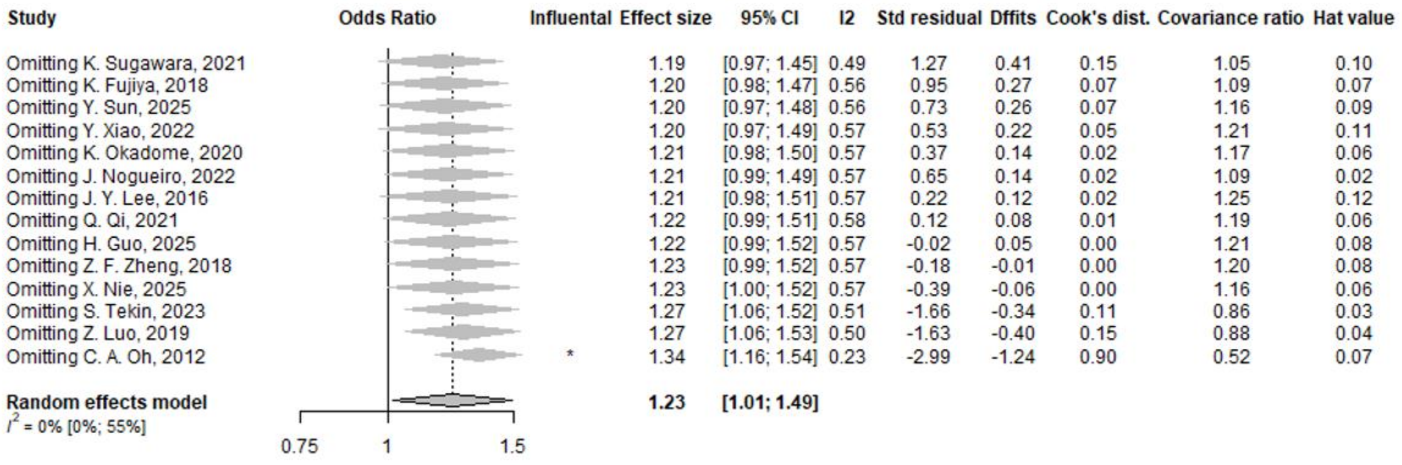

Study	Influental	Effect size	95% CI	I2	Std residual	Dffits	Cook's dist.	Covariance ratio	Hat value
Omitting K. Sugawara, 2021		1.19	[0.97; 1.45]	0.49	1.27	0.41	0.15	1.05	0.10
Omitting K. Fujiya, 2018		1.20	[0.98; 1.47]	0.56	0.95	0.27	0.07	1.09	0.07
Omitting Y. Sun, 2025		1.20	[0.97; 1.48]	0.56	0.73	0.26	0.07	1.16	0.09
Omitting Y. Xiao, 2022		1.20	[0.97; 1.49]	0.57	0.53	0.22	0.05	1.21	0.11
Omitting K. Okadome, 2020		1.21	[0.98; 1.50]	0.57	0.37	0.14	0.02	1.17	0.06
Omitting J. Nogueiro, 2022		1.21	[0.99; 1.49]	0.57	0.65	0.14	0.02	1.09	0.02
Omitting J. Y. Lee, 2016		1.21	[0.98; 1.51]	0.57	0.22	0.12	0.02	1.25	0.12
Omitting Q. Qi, 2021		1.22	[0.99; 1.51]	0.58	0.12	0.08	0.01	1.19	0.06
Omitting H. Guo, 2025		1.22	[0.99; 1.52]	0.57	-0.02	0.05	0.00	1.21	0.08
Omitting Z. F. Zheng, 2018		1.23	[0.99; 1.52]	0.57	-0.18	-0.01	0.00	1.20	0.08
Omitting X. Nie, 2025		1.23	[1.00; 1.52]	0.57	-0.39	-0.06	0.00	1.16	0.06
Omitting S. Tekin, 2023		1.27	[1.06; 1.52]	0.51	-1.66	-0.34	0.11	0.86	0.03
Omitting Z. Luo, 2019		1.27	[1.06; 1.53]	0.50	-1.63	-0.40	0.15	0.88	0.04
Omitting C. A. Oh, 2012	*	1.34	[1.16; 1.54]	0.23	-2.99	-1.24	0.90	0.52	0.07
**Random effects model**		**1.23**	**[1.01; 1.49]**						

I^2 = 0% [0%; 55%]

Figure S215 Funnel plot: Association between malnutrition diagnosis and ASA score (American Society of Anesthesiologists Physical Status Classification) in gastrointestinal cancer (GLIM criteria)

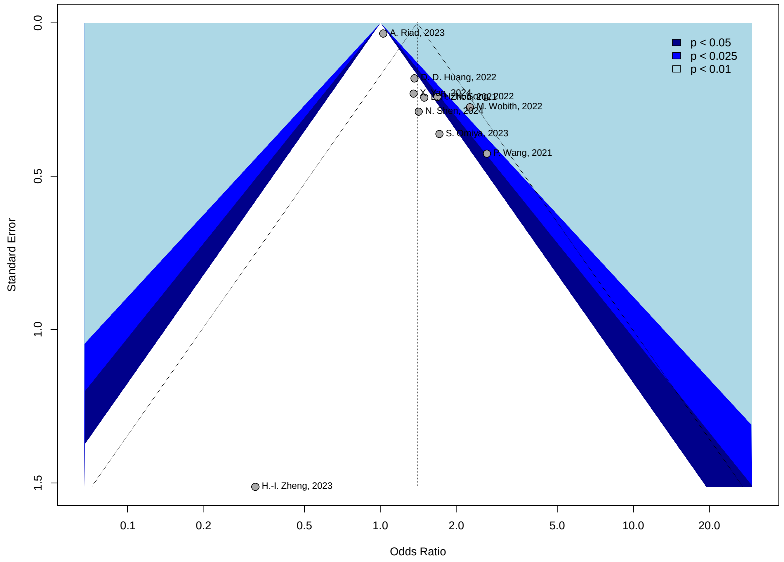

Egger test: t = 1.20, df = 8, p-value = 0.2658

Figure S215.1 Leave-one-out plot: Association between malnutrition diagnosis and ASA score (American Society of Anesthesiologists Physical Status Classification) in gastrointestinal cancer (GLIM criteria)

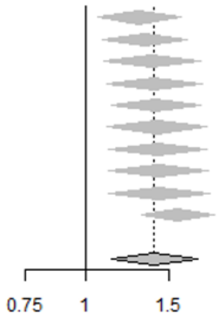

Study	Influential	Effect size	95% CI	I2	Std residual	Dffits	Cook's dist.	Covariance ratio	Hat value
Omitting M. Wobith, 2022	*	1.30	[1.06; 1.58]	0.54	1.74	0.83	0.61	0.86	0.08
Omitting P. Wang, 2021		1.34	[1.09; 1.64]	0.60	1.48	0.48	0.22	0.91	0.04
Omitting H. N. Song, 2022		1.38	[1.09; 1.75]	0.62	0.61	0.11	0.01	1.21	0.10
Omitting S. Omiya, 2023		1.40	[1.11; 1.76]	0.65	0.48	-0.00	0.00	1.17	0.05
Omitting H.-I. Zheng, 2023		1.41	[1.13; 1.74]	0.66	-0.97	-0.07	0.00	1.01	0.00
Omitting L. P. Zhou, 2021		1.41	[1.11; 1.79]	0.65	0.17	-0.10	0.01	1.26	0.10
Omitting N. Shen, 2024		1.42	[1.12; 1.80]	0.66	-0.01	-0.16	0.03	1.24	0.08
Omitting X. Yan, 2024		1.43	[1.12; 1.82]	0.66	-0.17	-0.22	0.05	1.27	0.11
Omitting D. D. Huang, 2022		1.43	[1.11; 1.83]	0.65	-0.17	-0.22	0.05	1.32	0.14
Omitting A. Riad, 2023	*	1.56	[1.31; 1.86]	0.00	-4.34	-5.98	1.46	0.94	0.29
**Random effects model**		**1.40**	**[1.13; 1.72]**						

I^2 = 0% [0%; 62%]

Figure S216 Funnel plot: Association between malnutrition-related complication risk and ASA score (American Society of Anesthesiologists Physical Status Classification) in colorectal cancer (Biological composite scores)

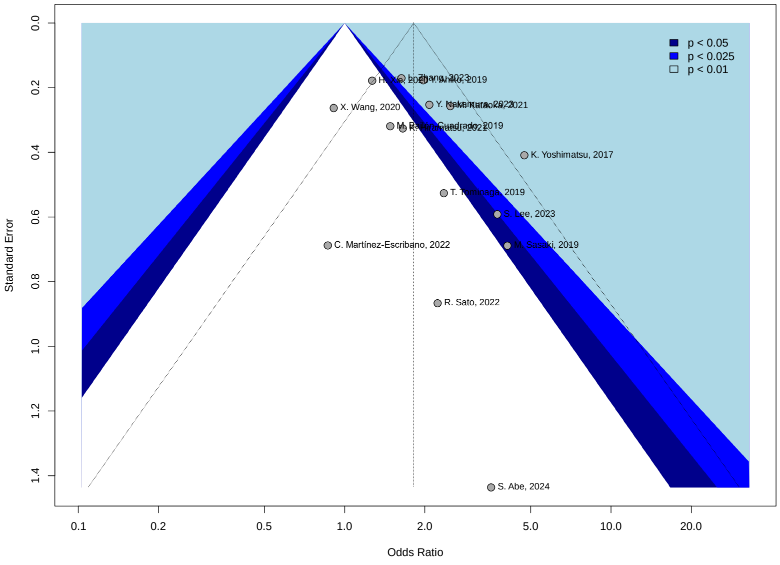

Egger test: t = 0.91, df = 13, p-value = 0.3800

Figure S216.1 Leave-one-out plot: Association between malnutrition-related complication risk and ASA score (American Society of Anesthesiologists Physical Status Classification) in colorectal cancer (Biological composite scores)

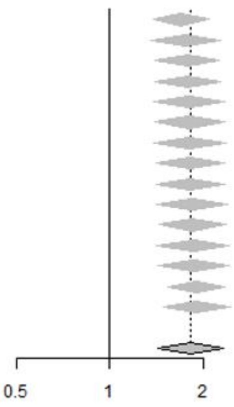

Study	Influental	Effect size	95% CI	I2	Std residual	Dffits	Cook's dist.	Covariance ratio	Hat value
Omitting K. Yoshimatsu, 2017		1.70	[1.38; 2.08]	0.25	2.28	0.68	0.38	0.71	0.05
Omitting M. Kataoka, 2021		1.76	[1.36; 2.28]	0.39	0.90	0.28	0.08	1.13	0.09
Omitting S. Lee, 2023		1.77	[1.39; 2.26]	0.40	1.14	0.21	0.05	1.00	0.03
Omitting M. Sasaki, 2019		1.78	[1.40; 2.27]	0.41	1.12	0.18	0.03	1.00	0.02
Omitting Y. Nakamura, 2023		1.80	[1.38; 2.35]	0.43	0.36	0.07	0.01	1.21	0.10
Omitting T. Tominaga, 2019		1.81	[1.40; 2.34]	0.44	0.44	0.04	0.00	1.13	0.04
Omitting Y. Ahiko, 2019		1.81	[1.38; 2.37]	0.43	0.25	0.04	0.00	1.26	0.13
Omitting S. Abe, 2024		1.82	[1.41; 2.34]	0.44	0.46	-0.01	0.00	1.09	0.01
Omitting R. Sato, 2022		1.82	[1.41; 2.35]	0.44	0.22	-0.02	0.00	1.12	0.02
Omitting K. Hiramatsu, 2021		1.84	[1.41; 2.40]	0.45	-0.24	-0.12	0.02	1.19	0.07
Omitting C. Martínez-Escribano, 2022		1.85	[1.45; 2.36]	0.42	-1.02	-0.15	0.02	1.02	0.02
Omitting L. Zhang, 2023		1.86	[1.42; 2.43]	0.44	-0.36	-0.18	0.04	1.25	0.13
Omitting M. Bailón-Cuadrado, 2019		1.86	[1.43; 2.41]	0.44	-0.51	-0.19	0.04	1.17	0.07
Omitting X. Wang, 2020		1.90	[1.54; 2.34]	0.23	-2.26	-0.45	0.14	0.75	0.09
Omitting H. Xie, 2020		1.91	[1.48; 2.46]	0.34	-1.26	-0.46	0.19	1.08	0.13
**Random effects model**		**1.82**	**[1.43; 2.31]**						

I^2 = 0% [0%; 54%]

Figure S217 Funnel plot: Association between malnutrition-related complication risk and CA19-9 level in upper gastrointestinal cancer (Biological composite scores)

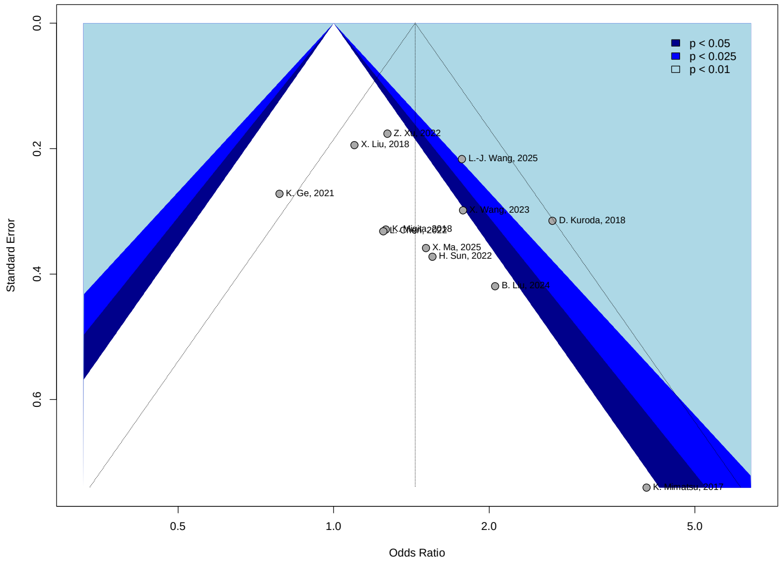

Egger test: t = 1.07, df = 10, p-value = 0.3118

Figure S217.1 Leave-one-out plot: Association between malnutrition-related complication risk and CA19-9 level in upper gastrointestinal cancer (Biological composite scores)

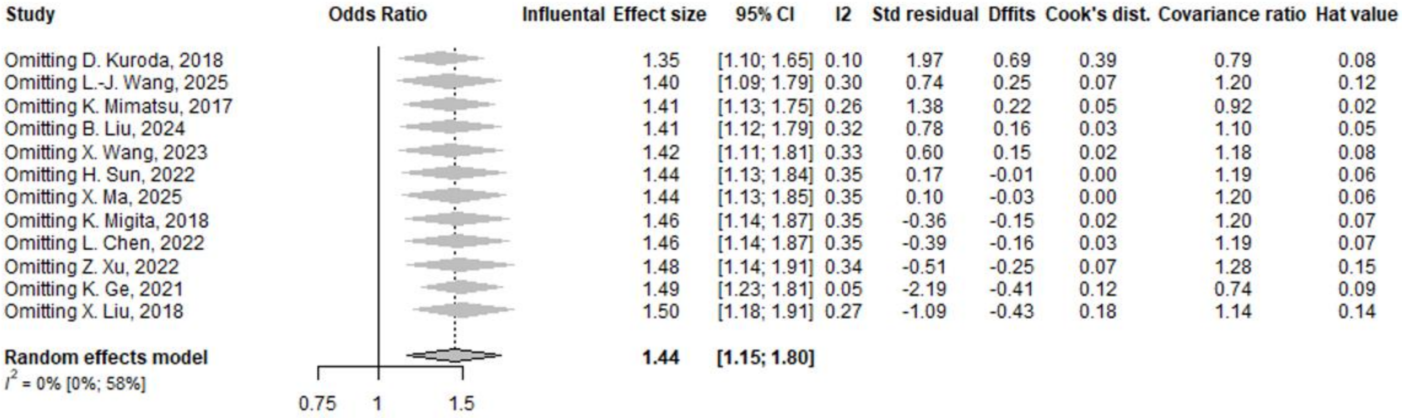

Study	Odds Ratio	Influental	Effect size	95% CI	I2	Std residual	Dffits	Cook's dist.	Covariance ratio	Hat value
Omitting D. Kuroda, 2018			1.35	[1.10; 1.65]	0.10	1.97	0.69	0.39	0.79	0.08
Omitting L.-J. Wang, 2025			1.40	[1.09; 1.79]	0.30	0.74	0.25	0.07	1.20	0.12
Omitting K. Mimatsu, 2017			1.41	[1.13; 1.75]	0.26	1.38	0.22	0.05	0.92	0.02
Omitting B. Liu, 2024			1.41	[1.12; 1.79]	0.32	0.78	0.16	0.03	1.10	0.05
Omitting X. Wang, 2023			1.42	[1.11; 1.81]	0.33	0.60	0.15	0.02	1.18	0.08
Omitting H. Sun, 2022			1.44	[1.13; 1.84]	0.35	0.17	-0.01	0.00	1.19	0.06
Omitting X. Ma, 2025			1.44	[1.13; 1.85]	0.35	0.10	-0.03	0.00	1.20	0.06
Omitting K. Migita, 2018			1.46	[1.14; 1.87]	0.35	-0.36	-0.15	0.02	1.20	0.07
Omitting L. Chen, 2022			1.46	[1.14; 1.87]	0.35	-0.39	-0.16	0.03	1.19	0.07
Omitting Z. Xu, 2022			1.48	[1.14; 1.91]	0.34	-0.51	-0.25	0.07	1.28	0.15
Omitting K. Ge, 2021			1.49	[1.23; 1.81]	0.05	-2.19	-0.41	0.12	0.74	0.09
Omitting X. Liu, 2018			1.50	[1.18; 1.91]	0.27	-1.09	-0.43	0.18	1.14	0.14
**Random effects model**			**1.44**	**[1.15; 1.80]**						
I^2 = 0% [0%; 58%]										

Figure S218 Funnel plot: Association between malnutrition-related complication risk and CA19-9 level in hepato-biliopancreatic cancer (Biological composite scores)

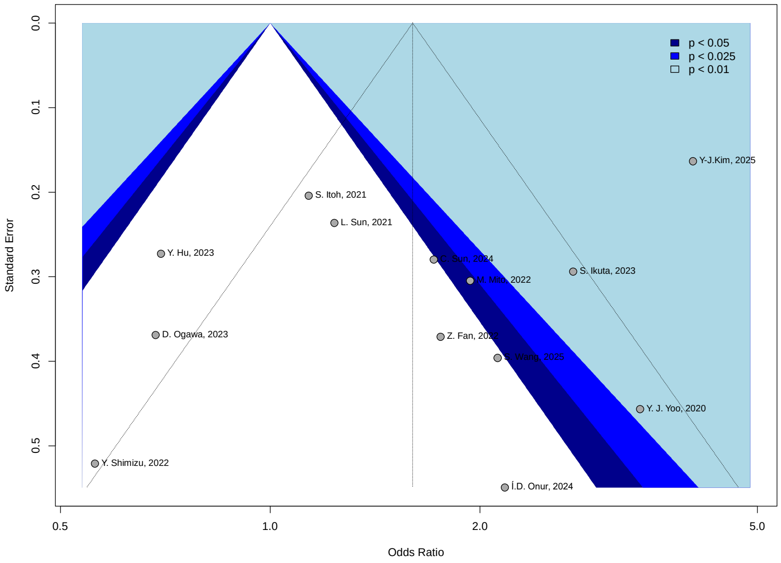

Egger test: t = -0.89, df = 11, p-value = 0.3905

Figure S218.1 Leave-one-out plot: Association between malnutrition-related complication risk and CA19-9 level in hepato-biliopancreatic cancer (Biological composite scores)

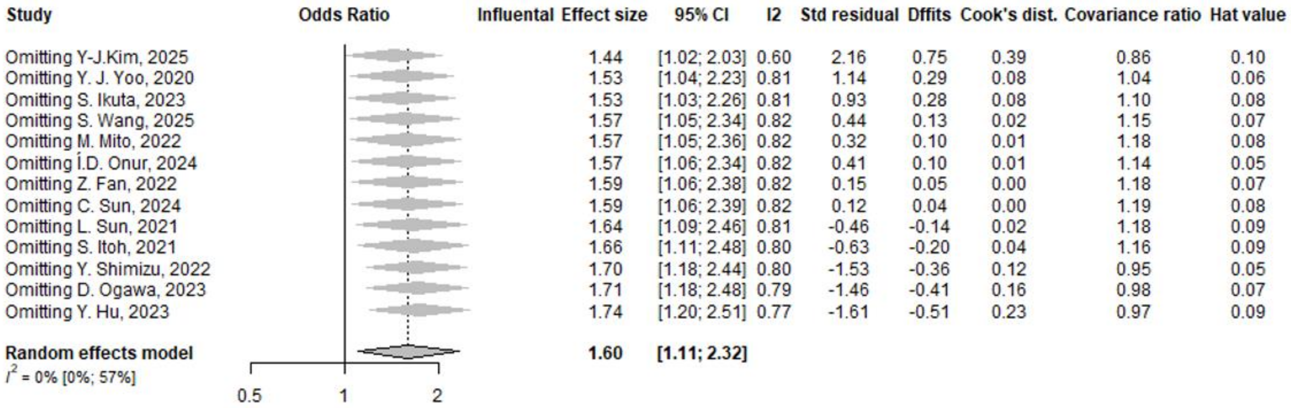

Figure S219 Funnel plot: Association between malnutrition-related complication risk and anaemia in gastrointestinal cancer (Biological composite scores)

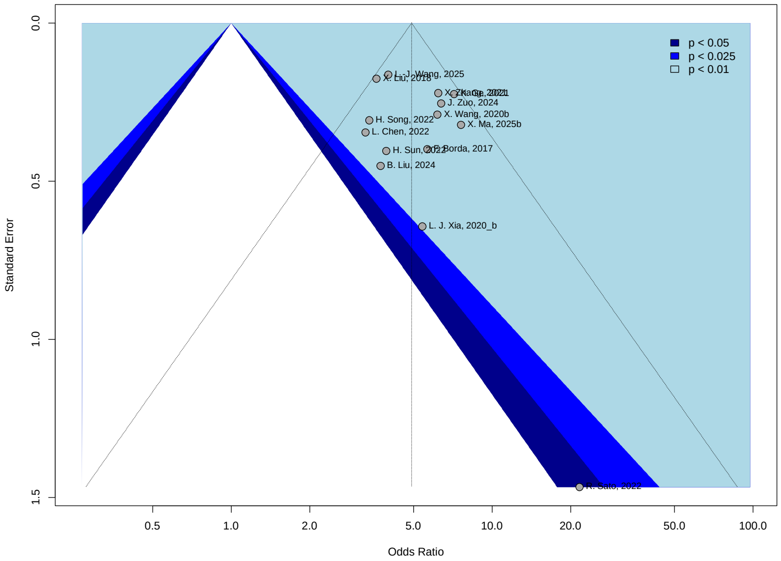

Egger test: t = 1.11, df = 12, p-value = 0.2873

Figure S219.1 Leave-one-out plot: Association between malnutrition-related complication risk and anaemia in gastrointestinal cancer (Biological composite scores)

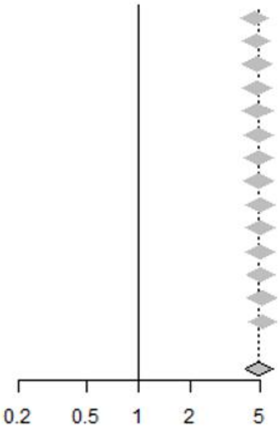

Study	Influental	Effect size	95% CI	I2	Std residual	Dffits	Cook's dist.	Covariance ratio	Hat value
Omitting K. Ge, 2021		4.66	[3.89; 5.59]	0.10	1.68	0.67	0.38	0.96	0.11
Omitting X. Ma, 2025b		4.77	[3.96; 5.74]	0.18	1.33	0.36	0.12	0.99	0.06
Omitting X. Zhang, 2021		4.77	[3.92; 5.81]	0.22	0.96	0.33	0.11	1.13	0.11
Omitting J. Zuo, 2024		4.79	[3.94; 5.82]	0.23	0.95	0.29	0.09	1.11	0.09
Omitting X. Wang, 2020b		4.83	[3.97; 5.88]	0.25	0.73	0.19	0.04	1.12	0.07
Omitting R. Sato, 2022		4.89	[4.06; 5.88]	0.24	1.01	0.06	0.00	1.00	0.00
Omitting F. Borda, 2017		4.90	[4.02; 5.96]	0.28	0.33	0.04	0.00	1.13	0.04
Omitting L. J. Xia, 2020_b		4.92	[4.05; 5.98]	0.28	0.14	-0.02	0.00	1.11	0.02
Omitting B. Liu, 2024		4.97	[4.09; 6.03]	0.27	-0.59	-0.14	0.02	1.10	0.03
Omitting H. Sun, 2022		4.97	[4.09; 6.04]	0.27	-0.53	-0.14	0.02	1.11	0.04
Omitting L. Chen, 2022		5.02	[4.16; 6.05]	0.22	-1.13	-0.25	0.06	1.03	0.05
Omitting H. Song, 2022		5.03	[4.17; 6.08]	0.22	-1.16	-0.29	0.08	1.04	0.06
Omitting L.-J. Wang, 2025		5.11	[4.18; 6.25]	0.20	-1.08	-0.48	0.22	1.18	0.16
Omitting X. Liu, 2018		5.17	[4.29; 6.24]	0.10	-1.69	-0.68	0.37	1.02	0.15
**Random effects model**		**4.91**	**[4.09; 5.90]**						

I^2 = 0% [0%; 55%]

Figure S220 Funnel plot: Association between malnutrition-related complication risk and alpha-fetoprotein level in hepatocellular carcinoma (Biological composite scores)

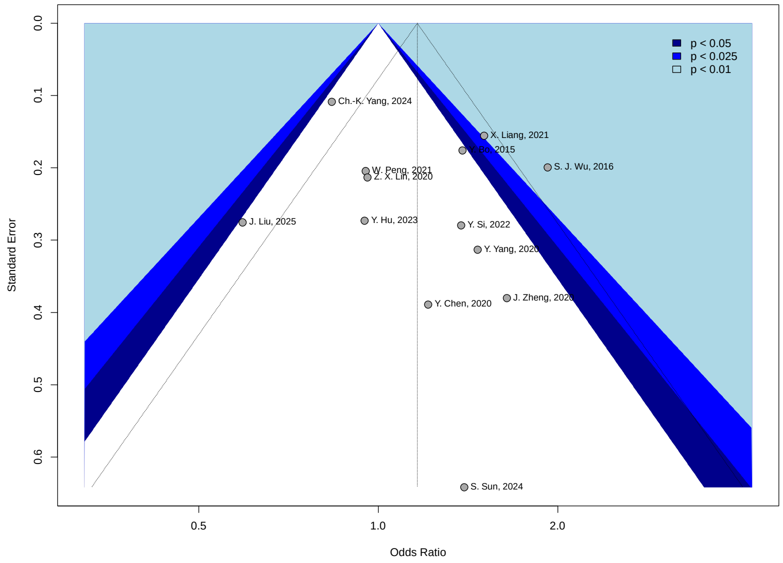

Egger test: t = 0.60, df = 11, p-value = 0.5634

Figure S220.1 Leave-one-out plot: Association between malnutrition-related complication risk and alpha-fetoprotein level in hepatocellular carcinoma (Biological composite scores)

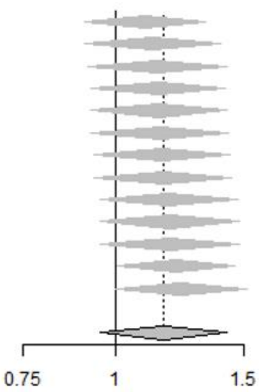

Study	Odds Ratio	Influental	Effect size	95% CI	I2	Std residual	Dffits	Cook's dist.	Covariance ratio	Hat value
Omitting S. J. Wu, 2016			1.10	[0.91; 1.33]	0.47	2.02	0.71	0.40	0.88	0.10
Omitting X. Liang, 2021			1.12	[0.91; 1.39]	0.56	1.01	0.37	0.13	1.13	0.12
Omitting Y. Bo, 2015			1.14	[0.92; 1.42]	0.60	0.63	0.21	0.05	1.18	0.11
Omitting J. Zheng, 2020			1.14	[0.93; 1.41]	0.61	0.80	0.17	0.03	1.08	0.04
Omitting Y. Yang, 2020			1.15	[0.93; 1.42]	0.61	0.61	0.15	0.02	1.12	0.06
Omitting Y. Si, 2022			1.15	[0.93; 1.43]	0.61	0.48	0.12	0.01	1.15	0.07
Omitting S. Sun, 2024			1.16	[0.94; 1.43]	0.62	0.27	0.03	0.00	1.11	0.02
Omitting Y. Chen, 2020			1.16	[0.94; 1.44]	0.62	0.09	0.01	0.00	1.14	0.04
Omitting Y. Hu, 2023			1.18	[0.95; 1.47]	0.62	-0.59	-0.17	0.03	1.14	0.07
Omitting Z. X. Lin, 2020			1.19	[0.95; 1.47]	0.61	-0.64	-0.21	0.05	1.16	0.09
Omitting W. Peng, 2021			1.19	[0.96; 1.48]	0.61	-0.68	-0.22	0.05	1.15	0.09
Omitting J. Liu, 2025			1.21	[1.01; 1.45]	0.53	-2.14	-0.47	0.18	0.82	0.07
Omitting Ch.-K. Yang, 2024			1.23	[1.00; 1.51]	0.42	-1.58	-0.66	0.36	1.04	0.14
**Random effects model**			**1.16**	**[0.95; 1.42]**						
I^2 = 0% [0%; 57%]										

Figure S221: Funnel plot: Association between malnutrition-related complication risk and T stage (T>1 vs. T≤1) in colorectal cancer (Biological composite scores)

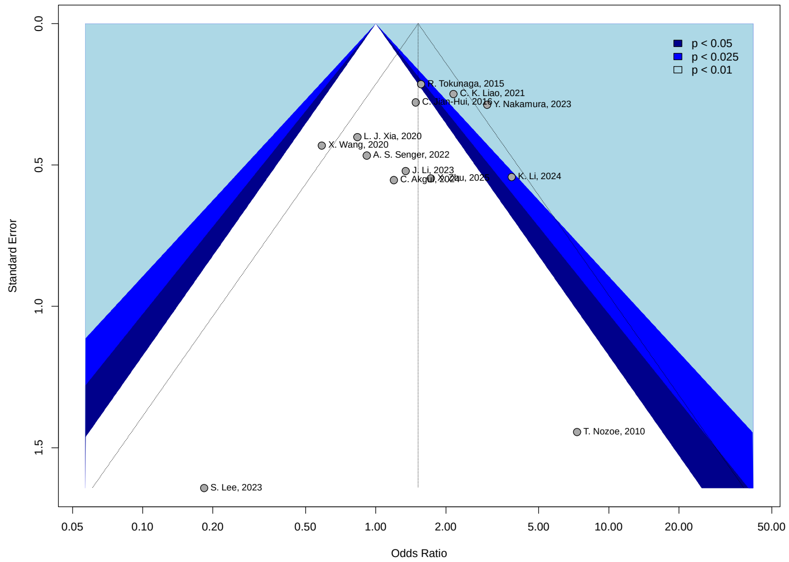

Egger test: t = -1.91, df = 11, p-value = 0.0819

Figure S221.1: Leave-one-out plot: Association between malnutrition-related complication risk and T stage (T>1 vs. T≤1) in colorectal cancer (Biological composite scores)

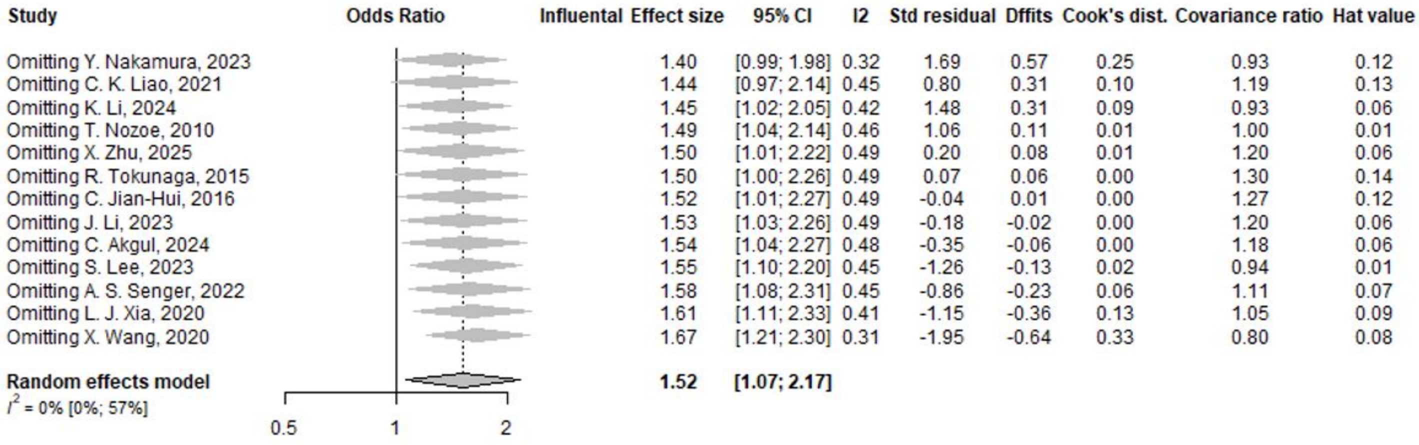

Figure S222: Funnel plot: Association between malnutrition-related complication risk and BCLC stage (C vs. B) in hepatocellular carcinoma (Biological composite scores)

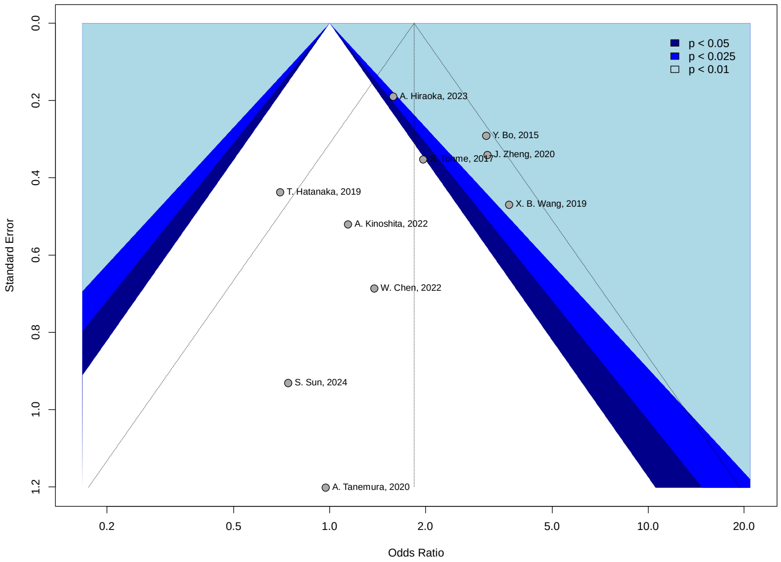

Egger test: t = -0.36, df = 8, p-value = 0.7261

Figure S222.1: Leave-one-out plot: Association between malnutrition-related complication risk and BCLC stage (C vs. B) in hepatocellular carcinoma (Biological composite scores)

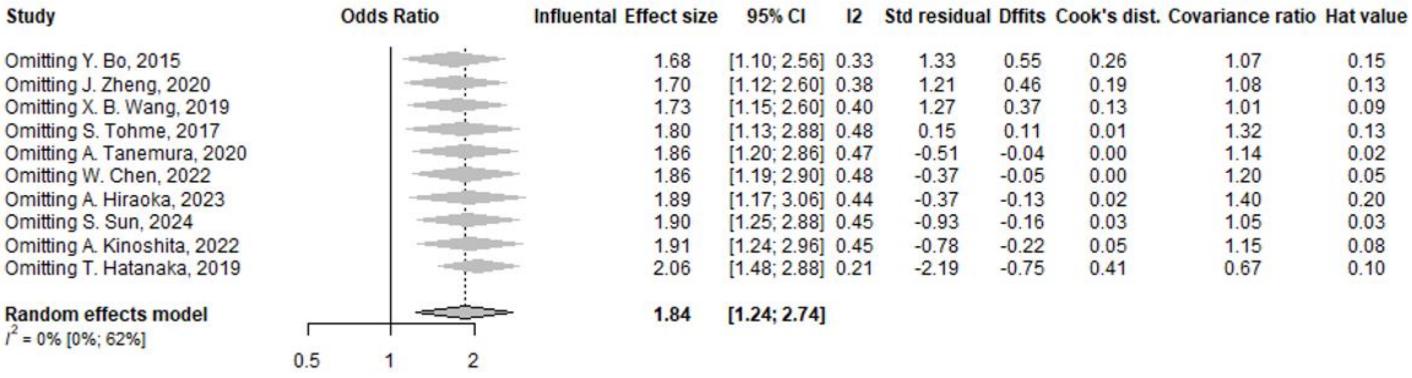

Study	Odds Ratio	Influental	Effect size	95% CI	I2	Std residual	Dffits	Cook's dist.	Covariance ratio	Hat value
Omitting Y. Bo, 2015			1.68	[1.10; 2.56]	0.33	1.33	0.55	0.26	1.07	0.15
Omitting J. Zheng, 2020			1.70	[1.12; 2.60]	0.38	1.21	0.46	0.19	1.08	0.13
Omitting X. B. Wang, 2019			1.73	[1.15; 2.60]	0.40	1.27	0.37	0.13	1.01	0.09
Omitting S. Tohme, 2017			1.80	[1.13; 2.88]	0.48	0.15	0.11	0.01	1.32	0.13
Omitting A. Tanemura, 2020			1.86	[1.20; 2.86]	0.47	-0.51	-0.04	0.00	1.14	0.02
Omitting W. Chen, 2022			1.86	[1.19; 2.90]	0.48	-0.37	-0.05	0.00	1.20	0.05
Omitting A. Hiraoka, 2023			1.89	[1.17; 3.06]	0.44	-0.37	-0.13	0.02	1.40	0.20
Omitting S. Sun, 2024			1.90	[1.25; 2.88]	0.45	-0.93	-0.16	0.03	1.05	0.03
Omitting A. Kinoshita, 2022			1.91	[1.24; 2.96]	0.45	-0.78	-0.22	0.05	1.15	0.08
Omitting T. Hatanaka, 2019			2.06	[1.48; 2.88]	0.21	-2.19	-0.75	0.41	0.67	0.10
**Random effects model**			**1.84**	**[1.24; 2.74]**						

I^2 = 0% [0%; 62%]

Figure S223: Funnel plot: Association between malnutrition-related complication risk and etiology (viral vs. non-viral) in hepatocellular carcinoma (Biological composite scores)

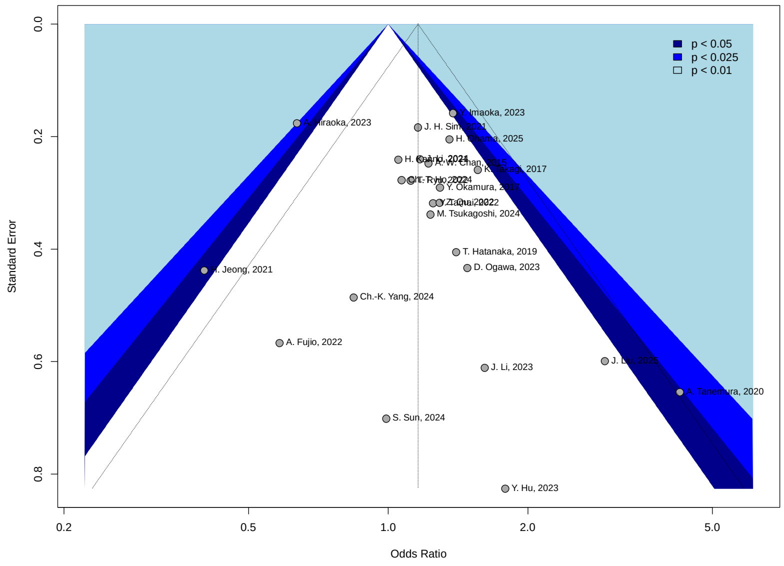

Egger test: t = 0.59, df = 22, p-value = 0.5631

Figure S223.1: Leave-one-out plot: Association between malnutrition-related complication risk and etiology (viral vs. non-viral) in hepatocellular carcinoma (Biological composite scores)

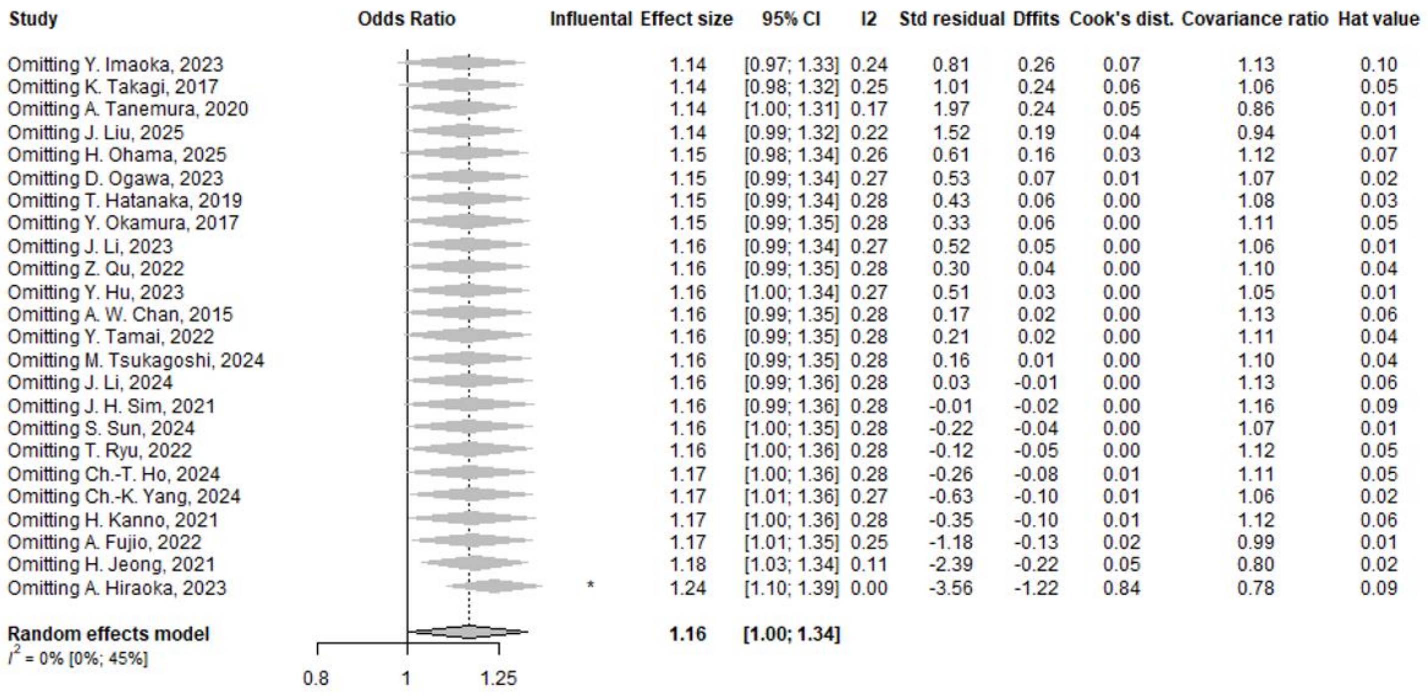

Study	Influental	Effect size	95% CI	I2	Std residual	Dffits	Cook's dist.	Covariance ratio	Hat value
Omitting Y. Imaoka, 2023		1.14	[0.97; 1.33]	0.24	0.81	0.26	0.07	1.13	0.10
Omitting K. Takagi, 2017		1.14	[0.98; 1.32]	0.25	1.01	0.24	0.06	1.06	0.05
Omitting A. Tanemura, 2020		1.14	[1.00; 1.31]	0.17	1.97	0.24	0.05	0.86	0.01
Omitting J. Liu, 2025		1.14	[0.99; 1.32]	0.22	1.52	0.19	0.04	0.94	0.01
Omitting H. Ohama, 2025		1.15	[0.98; 1.34]	0.26	0.61	0.16	0.03	1.12	0.07
Omitting D. Ogawa, 2023		1.15	[0.99; 1.34]	0.27	0.53	0.07	0.01	1.07	0.02
Omitting T. Hatanaka, 2019		1.15	[0.99; 1.34]	0.28	0.43	0.06	0.00	1.08	0.03
Omitting Y. Okamura, 2017		1.15	[0.99; 1.35]	0.28	0.33	0.06	0.00	1.11	0.05
Omitting J. Li, 2023		1.16	[0.99; 1.34]	0.27	0.52	0.05	0.00	1.06	0.01
Omitting Z. Qu, 2022		1.16	[0.99; 1.35]	0.28	0.30	0.04	0.00	1.10	0.04
Omitting Y. Hu, 2023		1.16	[1.00; 1.34]	0.27	0.51	0.03	0.00	1.05	0.01
Omitting A. W. Chan, 2015		1.16	[0.99; 1.35]	0.28	0.17	0.02	0.00	1.13	0.06
Omitting Y. Tamai, 2022		1.16	[0.99; 1.35]	0.28	0.21	0.02	0.00	1.11	0.04
Omitting M. Tsukagoshi, 2024		1.16	[0.99; 1.35]	0.28	0.16	0.01	0.00	1.10	0.04
Omitting J. Li, 2024		1.16	[0.99; 1.36]	0.28	0.03	-0.01	0.00	1.13	0.06
Omitting J. H. Sim, 2021		1.16	[0.99; 1.36]	0.28	-0.01	-0.02	0.00	1.16	0.09
Omitting S. Sun, 2024		1.16	[1.00; 1.35]	0.28	-0.22	-0.04	0.00	1.07	0.01
Omitting T. Ryu, 2022		1.16	[1.00; 1.36]	0.28	-0.12	-0.05	0.00	1.12	0.05
Omitting Ch.-T. Ho, 2024		1.17	[1.00; 1.36]	0.28	-0.26	-0.08	0.01	1.11	0.05
Omitting Ch.-K. Yang, 2024		1.17	[1.01; 1.36]	0.27	-0.63	-0.10	0.01	1.06	0.02
Omitting H. Kanno, 2021		1.17	[1.00; 1.36]	0.28	-0.35	-0.10	0.01	1.12	0.06
Omitting A. Fujio, 2022		1.17	[1.01; 1.35]	0.25	-1.18	-0.13	0.02	0.99	0.01
Omitting H. Jeong, 2021		1.18	[1.03; 1.34]	0.11	-2.39	-0.22	0.05	0.80	0.02
Omitting A. Hiraoka, 2023	*	1.24	[1.10; 1.39]	0.00	-3.56	-1.22	0.84	0.78	0.09
**Random effects model**		**1.16**	**[1.00; 1.34]**						

I^2 = 0% [0%; 45%]

Figure S224: Funnel plot: Association between malnutrition-related complication risk and N stage (N≥1 vs. N0) in colorectal cancer (Biological composite scores)

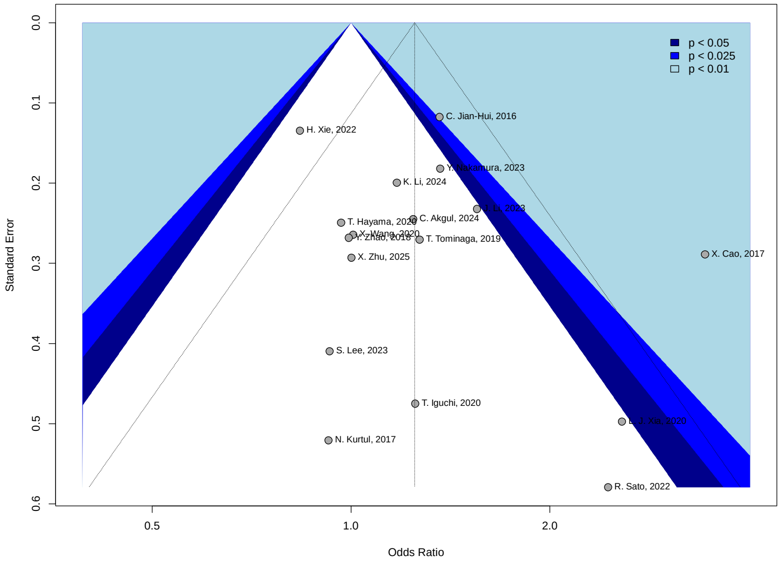

Egger test: t = 0.68, df = 15, p-value = 0.5084

Figure S224.1: Leave-one-out plot: Association between malnutrition-related complication risk and N stage (N≥1 vs. N0) in colorectal cancer (Biological composite scores)

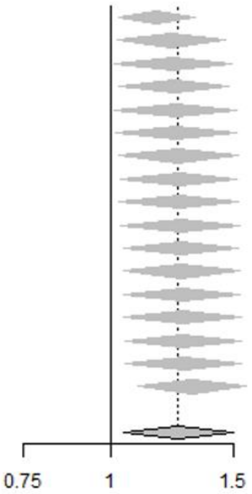

Study	Influential	Effect size	95% CI	I2	Std residual	Dffits	Cook's dist.	Covariance ratio	Hat value
Omitting X. Cao, 2017	*	1.17	[1.03; 1.32]	0.09	3.60	1.00	0.64	0.49	0.05
Omitting L. J. Xia, 2020		1.22	[1.02; 1.46]	0.46	1.36	0.23	0.05	0.96	0.02
Omitting J. Li, 2023		1.23	[1.01; 1.49]	0.48	0.68	0.17	0.03	1.12	0.07
Omitting R. Sato, 2022		1.23	[1.03; 1.48]	0.47	1.10	0.16	0.02	1.00	0.02
Omitting C. Jian-Hui, 2016		1.24	[1.01; 1.51]	0.48	0.33	0.09	0.01	1.20	0.11
Omitting Y. Nakamura, 2023		1.24	[1.02; 1.51]	0.49	0.30	0.07	0.00	1.18	0.09
Omitting T. Tominaga, 2019		1.25	[1.03; 1.52]	0.50	0.04	-0.02	0.00	1.15	0.06
Omitting T. Iguchi, 2020		1.25	[1.03; 1.52]	0.50	-0.00	-0.03	0.00	1.12	0.03
Omitting C. Akgul, 2024		1.25	[1.03; 1.52]	0.50	-0.02	-0.03	0.00	1.16	0.07
Omitting K. Li, 2024		1.26	[1.03; 1.53]	0.50	-0.21	-0.09	0.01	1.17	0.08
Omitting N. Kurtul, 2017		1.26	[1.04; 1.52]	0.49	-0.53	-0.10	0.01	1.08	0.02
Omitting S. Lee, 2023		1.26	[1.04; 1.53]	0.49	-0.64	-0.14	0.02	1.09	0.03
Omitting X. Zhu, 2025		1.27	[1.04; 1.53]	0.49	-0.61	-0.16	0.03	1.11	0.05
Omitting X. Wang, 2020		1.27	[1.05; 1.54]	0.49	-0.63	-0.18	0.03	1.12	0.06
Omitting Y. Zhao, 2018		1.27	[1.05; 1.54]	0.49	-0.67	-0.18	0.04	1.11	0.06
Omitting T. Hayama, 2020		1.27	[1.05; 1.54]	0.48	-0.78	-0.22	0.05	1.10	0.06
Omitting H. Xie, 2022		1.31	[1.09; 1.56]	0.28	-1.82	-0.62	0.29	0.95	0.11
**Random effects model**		**1.25**	**[1.04; 1.50]**						

I^2 = 0% [0%; 51%]

Figure S225: Funnel plot: Association between malnutrition-related complication risk and macrovascular involvement in colorectal cancer (Biological composite scores)

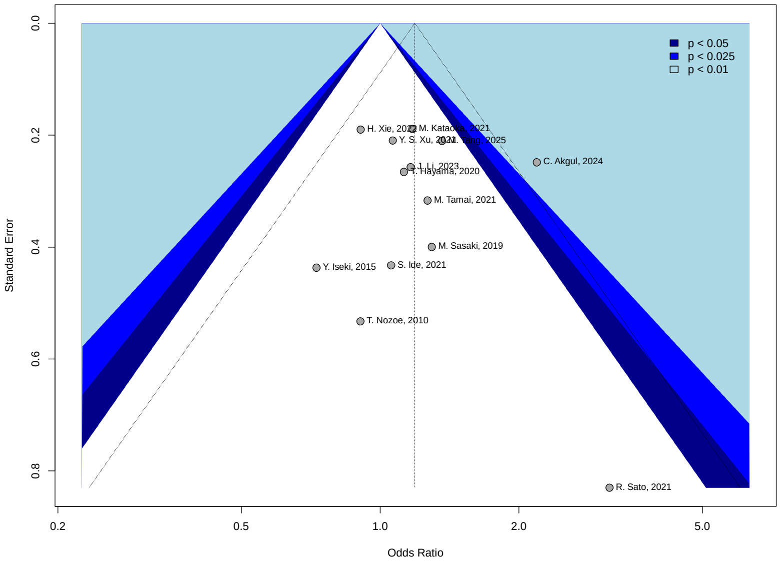

Egger test: t = 1.32, df = 11, p-value = 0.2137

Figure S225.1: Leave-one-out plot: Association between malnutrition-related complication risk and macrovascular involvement in colorectal cancer (Biological composite scores)

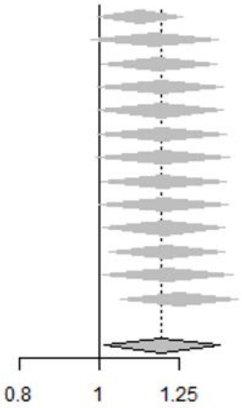

Study	Odds Ratio	Influental	Effect size	95% CI	I2	Std residual	Dffits	Cook's dist.	Covariance ratio	Hat value
Omitting C. Akgul, 2024		*	1.12	[0.99; 1.26]	0.00	2.57	0.81	0.66	1.10	0.09
Omitting M. Tang, 2025			1.17	[0.97; 1.40]	0.03	0.67	0.24	0.06	1.19	0.13
Omitting R. Sato, 2021			1.18	[1.00; 1.39]	0.00	1.18	0.11	0.01	1.01	0.01
Omitting M. Tamai, 2021			1.19	[0.99; 1.42]	0.07	0.20	0.03	0.00	1.16	0.06
Omitting M. Sasaki, 2019			1.19	[1.00; 1.41]	0.07	0.21	0.02	0.00	1.14	0.04
Omitting J. Li, 2023			1.19	[1.00; 1.43]	0.07	-0.09	-0.05	0.00	1.20	0.08
Omitting M. Kataoka, 2021			1.19	[0.99; 1.44]	0.07	-0.07	-0.05	0.00	1.29	0.16
Omitting S. Ide, 2021			1.19	[1.00; 1.42]	0.07	-0.28	-0.07	0.01	1.13	0.03
Omitting T. Hayama, 2020			1.20	[1.00; 1.43]	0.07	-0.21	-0.08	0.01	1.19	0.08
Omitting T. Nozoe, 2010			1.20	[1.01; 1.42]	0.05	-0.51	-0.09	0.01	1.09	0.02
Omitting Y. Iseki, 2015			1.21	[1.02; 1.42]	0.00	-1.14	-0.20	0.04	1.03	0.03
Omitting Y. S. Xu, 2021			1.21	[1.01; 1.45]	0.05	-0.55	-0.22	0.05	1.22	0.13
Omitting H. Xie, 2022			1.25	[1.06; 1.47]	0.00	-1.55	-0.66	0.44	1.18	0.16
**Random effects model**			**1.19**	**[1.01; 1.40]**						
I^2 = 0% [0%; 57%]										

Figure S226: Funnel plot: Association between malnutrition-related complication risk and tumor location (right-sided vs. left-sided) in colorectal cancer (Biological composite scores)

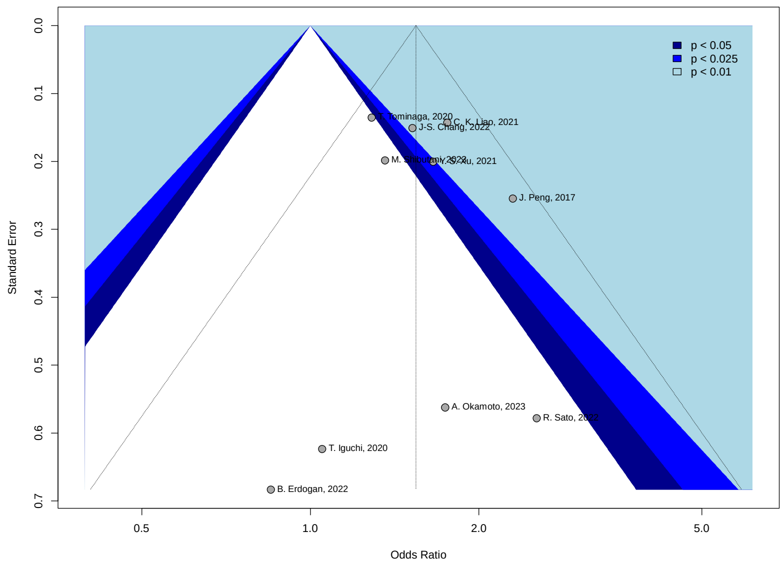

Egger test: t = -0.17, df = 8, p-value = 0.8657

Figure S226.1: Leave-one-out plot: Association between malnutrition-related complication risk and tumor location (right-sided vs. left-sided) in colorectal cancer (Biological composite scores)

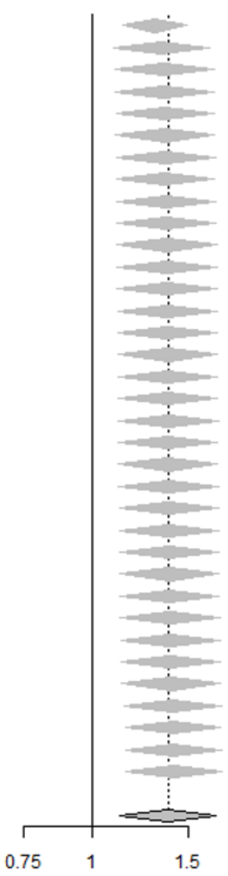

Study	Influental	Effect size	95% CI	I2	Std residual	Dffits	Cook's dist.	Covariance ratio	Hat value
Omitting Z. Hu, 2020	*	1.30	[1.14; 1.49]	0.52	5.78	0.77	0.31	0.45	0.03
Omitting J. Li, 2023		1.34	[1.09; 1.64]	0.70	1.53	0.28	0.07	0.99	0.03
Omitting X. Cao, 2017		1.36	[1.10; 1.67]	0.72	0.84	0.15	0.02	1.04	0.03
Omitting Y. Iseki, 2015		1.36	[1.10; 1.67]	0.72	0.83	0.14	0.02	1.04	0.03
Omitting L.-X. Liu, 2024		1.36	[1.11; 1.68]	0.73	0.72	0.11	0.01	1.04	0.02
Omitting G. Ucar, 2020		1.36	[1.11; 1.68]	0.72	0.59	0.11	0.01	1.06	0.03
Omitting C. K. Liao, 2021		1.37	[1.11; 1.68]	0.72	0.41	0.08	0.01	1.07	0.04
Omitting F. Teraishi, 2025		1.37	[1.11; 1.68]	0.73	0.74	0.07	0.00	1.02	0.01
Omitting X. Zhu, 2025		1.37	[1.11; 1.69]	0.73	0.37	0.06	0.00	1.06	0.03
Omitting M. Yamamoto, 2019		1.37	[1.11; 1.69]	0.73	0.26	0.05	0.00	1.07	0.03
Omitting Y. Nakamura, 2022		1.37	[1.11; 1.69]	0.73	0.22	0.04	0.00	1.07	0.03
Omitting H. Xie, 2022		1.37	[1.11; 1.69]	0.73	0.18	0.04	0.00	1.07	0.04
Omitting H. Zhou, 2020		1.37	[1.11; 1.69]	0.73	0.12	0.02	0.00	1.07	0.03
Omitting R. Tokunaga, 2016		1.37	[1.11; 1.70]	0.73	0.07	0.01	0.00	1.07	0.03
Omitting K. Maeda, 2014		1.38	[1.12; 1.70]	0.73	0.02	0.00	0.00	1.05	0.02
Omitting M. Shibutani, 2015		1.38	[1.12; 1.70]	0.73	-0.00	0.00	0.00	1.06	0.03
Omitting Y. Mohri, 2013		1.38	[1.12; 1.70]	0.73	-0.04	-0.01	0.00	1.07	0.03
Omitting M. Tamai, 2021		1.38	[1.12; 1.70]	0.73	-0.05	-0.01	0.00	1.06	0.03
Omitting Y. Li, 2025		1.38	[1.12; 1.70]	0.73	-0.06	-0.01	0.00	1.08	0.04
Omitting M. Sasaki, 2019		1.38	[1.12; 1.70]	0.73	-0.08	-0.01	0.00	1.06	0.03
Omitting T. Nozoe, 2010		1.38	[1.12; 1.70]	0.73	-0.10	-0.01	0.00	1.06	0.02
Omitting M. Kataoka, 2021		1.38	[1.12; 1.70]	0.73	-0.17	-0.03	0.00	1.07	0.03
Omitting M. Keskinikilic, 2024		1.38	[1.12; 1.70]	0.73	-0.29	-0.05	0.00	1.06	0.02
Omitting Y. Zhao, 2018		1.38	[1.12; 1.71]	0.73	-0.29	-0.05	0.00	1.06	0.03
Omitting T. Ikeya, 2015		1.39	[1.12; 1.71]	0.73	-0.43	-0.06	0.00	1.05	0.02
Omitting T. Tominaga, 2019		1.39	[1.12; 1.71]	0.73	-0.40	-0.07	0.00	1.06	0.03
Omitting T. Yagyu, 2022		1.39	[1.13; 1.71]	0.73	-0.49	-0.09	0.01	1.06	0.03
Omitting Y. Ahiko, 2019		1.39	[1.13; 1.71]	0.72	-0.51	-0.10	0.01	1.06	0.04
Omitting Y. Zhu, 2021		1.39	[1.13; 1.71]	0.73	-0.61	-0.11	0.01	1.05	0.03
Omitting A. S. Senger, 2022		1.39	[1.13; 1.72]	0.72	-0.63	-0.11	0.01	1.05	0.03
Omitting C. Jian-Hui, 2016		1.40	[1.13; 1.72]	0.70	-0.74	-0.15	0.02	1.05	0.04
Omitting S. Xiang, 2023		1.41	[1.15; 1.72]	0.72	-1.39	-0.22	0.05	0.99	0.03
Omitting T. Hayama, 2022		1.41	[1.15; 1.72]	0.72	-1.65	-0.23	0.05	0.96	0.02
Omitting G. Pian, 2022		1.41	[1.15; 1.73]	0.71	-1.55	-0.26	0.06	0.98	0.03
Omitting M. Bailón-Cuadrado, 2019		1.41	[1.16; 1.73]	0.71	-1.61	-0.27	0.07	0.98	0.03
**Random effects model**		**1.38**	**[1.12; 1.69]**						

I^2 = 0% [0%; 38%]

Figure S227: Funnel plot: Association between malnutrition-related complication risk and neoadjuvant chemo-, or radiotherapy in colorectal cancer (Biological composite scores)

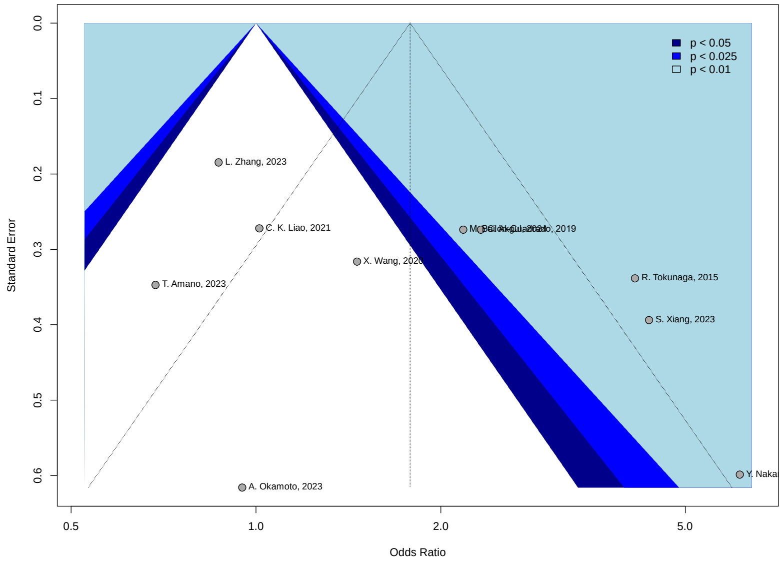

Egger test: t = -0.91, df = 8, p-value = 0.3885

Figure S227.1: Leave-one-out plot: Association between malnutrition-related complication risk and neoadjuvant chemo-, or radiotherapy in colorectal cancer (Biological composite scores)

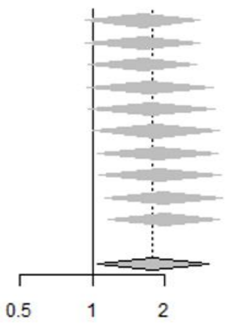

Study	Influential	Effect size	95% CI	I2	Std residual	Dffits	Cook's dist.	Covariance ratio	Hat value
Omitting R. Tokunaga, 2015		1.61	[0.93; 2.79]	0.76	1.27	0.44	0.18	1.04	0.10
Omitting S. Xiang, 2023		1.62	[0.94; 2.79]	0.77	1.30	0.43	0.17	1.02	0.10
Omitting Y. Nakamura, 2023		1.62	[0.97; 2.72]	0.78	1.54	0.43	0.16	0.92	0.07
Omitting C. Akgul, 2024		1.73	[0.95; 3.16]	0.80	0.37	0.12	0.02	1.25	0.11
Omitting M. Bailón-Cuadrado, 2019		1.75	[0.95; 3.20]	0.80	0.28	0.08	0.01	1.26	0.11
Omitting X. Wang, 2020		1.83	[1.00; 3.35]	0.81	-0.28	-0.11	0.01	1.25	0.11
Omitting A. Okamoto, 2023		1.87	[1.05; 3.33]	0.81	-0.72	-0.21	0.04	1.14	0.07
Omitting C. K. Liao, 2021		1.91	[1.07; 3.43]	0.80	-0.84	-0.30	0.09	1.17	0.11
Omitting L. Zhang, 2023		1.97	[1.12; 3.45]	0.74	-1.17	-0.43	0.18	1.09	0.12
Omitting T. Amano, 2023		1.97	[1.16; 3.37]	0.78	-1.45	-0.47	0.19	0.98	0.10
**Random effects model**		**1.78**	**[1.05; 3.02]**						

I^2 = 0% [0%; 62%]

Supplementary Table S1.: PRISMA 2020 Checklist and PRISMA 2020 for Abstract Checklist

PRISMA 2020 Checklist

Section and Topic	Item #	Checklist item	Location where the item is reported
**TITLE**			
Title	1	Identify the report as a systematic review.	1
**ABSTRACT**			
Abstract	2	See the PRISMA 2020 for Abstracts checklist.	
**INTRODUCTION**			
Rationale	3	Describe the rationale for the review in the context of existing knowledge.	3
Objectives	4	Provide an explicit statement of the objective(s) or question(s) the review addresses.	3
**METHODS**			
Eligibility criteria	5	Specify the inclusion and exclusion criteria for the review and how studies were grouped for the syntheses.	4
Information sources	6	Specify all databases, registers, websites, organisations, reference lists and other sources searched or consulted to identify studies. Specify the date when each source was last searched or consulted.	3-4
Search strategy	7	Present the full search strategies for all databases, registers and websites, including any filters and limits used.	3-4, Supplementary Document 2
Selection process	8	Specify the methods used to decide whether a study met the inclusion criteria of the review, including how many reviewers screened each record and each report retrieved, whether they worked independently, and if applicable, details of automation tools used in the process.	4, Supplementary Document 4
Data collection process	9	Specify the methods used to collect data from reports, including how many reviewers collected data from each report, whether they worked independently, any processes for obtaining or confirming data from study investigators, and if applicable, details of automation tools used in the process.	4, Supplementary Document 5
Data items	10a	List and define all outcomes for which data were sought. Specify whether all results that were compatible with each outcome domain in each study were sought (e.g. for all measures, time points, analyses), and if not, the methods used to decide which results to collect.	4
	10b	List and define all other variables for which data were sought (e.g. participant and intervention characteristics, funding sources). Describe any assumptions made about any missing or unclear information.	4
Study risk of bias assessment	11	Specify the methods used to assess risk of bias in the included studies, including details of the tool(s) used, how many reviewers assessed each study and whether they worked independently, and if applicable, details of automation tools used in the process.	5
Effect measures	12	Specify for each outcome the effect measure(s) (e.g. risk ratio, mean difference) used in the synthesis or presentation of results.	5
Synthesis	13a	Describe the processes used to decide which studies were eligible for each synthesis (e.g. tabulating the study intervention characteristics	5

Section and Topic	Item #	Checklist item	Location where the item is reported
methods		and comparing against the planned groups for each synthesis (item #5)).	
	13b	Describe any methods required to prepare the data for presentation or synthesis, such as handling of missing summary statistics, or data conversions.	5
	13c	Describe any methods used to tabulate or visually display results of individual studies and syntheses.	5
	13d	Describe any methods used to synthesize results and provide a rationale for the choice(s). If meta-analysis was performed, describe the model(s), method(s) to identify the presence and extent of statistical heterogeneity, and software package(s) used.	5
	13e	Describe any methods used to explore possible causes of heterogeneity among study results (e.g. subgroup analysis, meta-regression).	5
	13f	Describe any sensitivity analyses conducted to assess robustness of the synthesized results.	5
Reporting bias assessment	14	Describe any methods used to assess risk of bias due to missing results in a synthesis (arising from reporting biases).	4-5
Certainty assessment	15	Describe any methods used to assess certainty (or confidence) in the body of evidence for an outcome.	NA
**RESULTS**			
Study selection	16a	Describe the results of the search and selection process, from the number of records identified in the search to the number of studies included in the review, ideally using a flow diagram.	6
	16b	Cite studies that might appear to meet the inclusion criteria, but which were excluded, and explain why they were excluded.	6
Study characteristics	17	Cite each included study and present its characteristics.	6, Supplementary Document 12<br>Supplementary Table 2
Risk of bias in studies	18	Present assessments of risk of bias for each included study.	18, Supplementary Table 3
Results of individual studies	19	For all outcomes, present, for each study: (a) summary statistics for each group (where appropriate) and (b) an effect estimate and its precision (e.g. confidence/credible interval), ideally using structured tables or plots.	6-18<br>Supplementary Figures 1-124
Results of syntheses	20a	For each synthesis, briefly summarise the characteristics and risk of bias among contributing studies.	18
	20b	Present results of all statistical syntheses conducted. If meta-analysis was done, present for each the summary estimate and its precision (e.g. confidence/credible interval) and measures of statistical heterogeneity. If comparing groups, describe the direction of the effect.	6-18
	20c	Present results of all investigations of possible causes of heterogeneity among study results.	18, Supplementary Document 11
	20d	Present results of all sensitivity analyses conducted to assess the robustness of the synthesized results.	16, Supplementary

Section and Topic	Item #	Checklist item	Location where the item is reported
			Document 6
Reporting biases	21	Present assessments of risk of bias due to missing results (arising from reporting biases) for each synthesis assessed.	16, Supplementary Document 9
Certainty of evidence	22	Present assessments of certainty (or confidence) in the body of evidence for each outcome assessed.	NA
**DISCUSSION**			
Discussion	23a	Provide a general interpretation of the results in the context of other evidence.	19-22
	23b	Discuss any limitations of the evidence included in the review.	19-22
	23c	Discuss any limitations of the review processes used.	19-22
	23d	Discuss implications of the results for practice, policy, and future research.	19-22
**OTHER INFORMATION**			
Registration and protocol	24a	Provide registration information for the review, including register name and registration number, or state that the review was not registered.	3
	24b	Indicate where the review protocol can be accessed, or state that a protocol was not prepared.	3
	24c	Describe and explain any amendments to information provided at registration or in the protocol.	3
Support	25	Describe sources of financial or non-financial support for the review, and the role of the funders or sponsors in the review.	23
Competing interests	26	Declare any competing interests of review authors.	23
Availability of data, code and other materials	27	Report which of the following are publicly available and where they can be found: template data collection forms; data extracted from included studies; data used for all analyses; analytic code; any other materials used in the review.	23

NA= not applicable

From: Page MJ, McKenzie JE, Bossuyt PM, Boutron I, Hoffmann TC, Mulrow CD, et al. The PRISMA 2020 statement: an updated guideline for reporting systematic reviews. BMJ 2021;372:n71. doi: 10.1136/bmj.n71.
This work is licensed under CC BY 4.0. To view a copy of this license, visit https://creativecommons.org/licenses/by/4.0/

PRISMA 2020 for Abstract Checklist

Section and Topic	Item #	Checklist item	Reported (Yes/No)
**TITLE**			
Title	1	Identify the report as a systematic review.	Yes
**BACKGROUND**			
Objectives	2	Provide an explicit statement of the main objective(s) or question(s) the review addresses.	Yes
**METHODS**			
Eligibility criteria	3	Specify the inclusion and exclusion criteria for the review.	Yes
Information sources	4	Specify the information sources (e.g. databases, registers) used to identify studies and the date when each was last searched.	Yes
Risk of bias	5	Specify the methods used to assess risk of bias in the included studies.	No
Synthesis of results	6	Specify the methods used to present and synthesise results.	Yes
**RESULTS**			
Included studies	7	Give the total number of included studies and participants and summarise relevant characteristics of studies.	Yes
Synthesis of results	8	Present results for main outcomes, preferably indicating the number of included studies and participants for each. If meta-analysis was done, report the summary estimate and confidence/credible interval. If comparing groups, indicate the direction of the effect (i.e. which group is favoured).	Yes
**DISCUSSION**			
Limitations of evidence	9	Provide a brief summary of the limitations of the evidence included in the review (e.g. study risk of bias, inconsistency and imprecision).	Yes
Interpretation	10	Provide a general interpretation of the results and important implications.	Yes
**OTHER**			
Funding	11	Specify the primary source of funding for the review.	No
Registration	12	Provide the register name and registration number.	No

From: Page MJ, McKenzie JE, Bossuyt PM, Boutron I, Hoffmann TC, Mulrow CD, et al. The PRISMA 2020 statement: an updated guideline for reporting systematic reviews. BMJ 2021;372:n71. doi: 10.1136/bmj.n71. This work is licensed under CC BY 4.0. To view a copy of this license, visit https://creativecommons.org/licenses/by/4.0/

Supplementary Table S2.: Study characteristics table

First Author	Year of publication	Study Design	Country	Centers (N0)	total number of patients	age (mean±SD or median+ range*)	gender (No. (%) of females)	cancer type
A. Abe	2022	cross-sectional	Japan	1	145	72.2±8.5; **(50-89)	29 (20)	gastric cancer
A. Abe	2022	cross-sectional	Japan	1	95	67.2±13.2; **(37-89)	57 (60)	gastric cancer
F. Akad	2024	cross-sectional	Romania	1	51	66.91±12.68	17 (33.3)	gastric cancer
S. Abe	2024	prospective cohort	Japan	1	93	62 (52-67)	24 (25.8)	rectal cancer
T. Abe	2018	retrospective cohort	Japan	1	329	67 ††(61-74)	131 (39.8)	PDAC
Y. Ahiko	2019	retrospective cohort	Japan	1	830	78 (75-94)	360 (43.4)	colorectal cancer
B. Akagunduz	2022	retrospective cohort	Turkey	1	161	58.7 (32-80)	51 (31.7)	gastric cancer
M. Akaoka	2022	retropective cohort	Japan	1	213	68 (61-74)	42 (19.7)	hepatocellular carcinoma
C. Akgul	2024	cross-sectional	Turkey	1	300	NA	114 (38)	colorectal cancer
O. Akgül	2018	retrospective cohort	NA	NA	637	NA	NA	hepatobiliary cancer
O. Akgül	2018	retrospective cohort	NA	NA	637	NA	NA	hepatobiliary cancer

G. H. A-Lai	2019	retrospective cohort	China	1	90	64.34±NA	19 (21.1)	esophageal cancer
Ş. B. Alkan	2018	cross-sectional	Turkey	1	52	NA	21 (40.4)	gastrointestinal cancer
A. S. Almasaudi	2019	prospective cohort	United Kingdom	1	363	66±12	199 (54.8)	colorectal cancer
A. S. Almasaudi	2020	retrospective cohort	United Kingdom	1	795	NA	437 (55)	colorectal cancer
M. J. Alves	2017	cross-sectional	Brazil	1	38	NA	16 (42.1)	gastrointestinal cancer
T. Amano	2023	retropective cohort	Japan	1	237	67 (64-71)	81 (34.2)	rectal cancer
A. Attar	2012	prospective cohort	France	11	313	¶ 63 **(21-93)	33 (10.5)	gastrointestinal cancer
J. Bachmann	2013	retrospective cohort	Germany	1	242	65 (57-70)	106 (43.8)	PDAC
J. Bachmann	2008	prospective cohort	Germany	1	227	NA	98 (43.2)	PDAC
J. Bachmann	2009	prospective cohort	Germany	1	198	64 (57-79)	82 (41.4)	PDAC
X. Bai	2020	retrospective cohort	China	1	355	58 (20-88); 58.17±13.39	161 (45.4)	colorectal cancer
M. Bailón-Cuadrado	2019	prospective cohort	Spain	1	180	68.67±11.18	62 (34.4)	colorectal cancer
L. R. Barbosa	2014	cross-sectional	Brazil	1	66	61±NA	36 (54.5)	colorectal cancer
M. Bardakci	2021	retrospective cohort	Turkey	1	126	61 (36-88)	51 (40.5)	papilla vateri tumor
M. Barett	2011	prospective cohort	France	8	114	65 (22-92)	41 (36)	colorectal cancer

M. L. Batista	2013	cross-sectional	Brazil	1	43	NA	16 (37.2)	gastrointestinal cancer
M. L. Batista	2016	cross-sectional	Brazil	1	19	NA	8 (42.1)	gastrointestinal cancer
D. H. Bicakli	2019	prospective cohort	Turkey	1	96	61 (28-78)	37 (38.5)	PDAC
S. Bo	2010	cross-sectional	China	1	208	NA	70 (33.7)	gastric cancer
Y. Bo	2015	prospective cohort	China	1	620	53.8±10.6; **(28-80)	125 (20.2)	hepatocellular carcinoma
Y. Bo	2016	retrospective cohort	China	1	239	67.9±5.9; **(60-88)	89 (37.2)	esophageal squamous cell carcinoma
F. Borda	2014	retrospective cohort	Spain	1	207	NA	86 (41.5)	colorectal cancer
F. Borda	2017	retrospective cohort	Spain	1	124	NA	40 (32.3)	gastric cancer
D. R. Brown	2001	cross-sectional	USA	1	64	64±10.7	25 (39.1)	PDAC
L. R. Brown	2024	prospective cohort	United Kingdom	2	385	§66	108 (28.1)	esophageal, gastric cancer
L. Brown	2024	prospective cohort	United Kingdom	2	385	§66	108 (28.1)	esophageal, gastric cancer
L. R. Brown	2025	retropective cohort	United Kingdom	4	465	NA	154 (33.1)	esophageal, gastric cancer
W. Cai	2022	retropective cohort	China	1	1007	NA	267 (26.5)	gastric cancer
S. B. G. Campos	2022	cross-sectional	Brazil	1	31	58.97±14.96	16 (51.6)	colorectal cancer
J. Cao	2021	cross-sectional	China	72	1482	62.68±10.91	273 (18.4)	esophageal cancer

X. Cao	2017	retrospective cohort	China	1	228	69 (27-92); 66.11±12.79	94 (41.2)	colorectal cancer
V. W. Chai	2021	retrospective cohort	Australia	1	228	§ 69	89 (39)	colorectal cancer
V. Casalone	2025	retropective cohort	Italy	1	391	65±NA	159 (40.7)	gastrointestinal
A. W. Chan	2015	retrospective cohort	Hong Kong	1	324	56.8±10.9	41 (12.7)	hepatocellular carcinoma
L. Chang	2022	retrospective cohort	China	1	69	60 (44-78)	2 (2.9)	esophageal cancer
J-S. Chang	2022	retropective cohort	Taiwan	1	768	NA	273 (35.5)	colorectal cancer
L. Chang	2023	retropective cohort	China	1	62	§61**(42-85)	0 (0)	esophageal cancer
F. F. Chen	2016	prospective cohort	China	1	158	66.9±8.7	32 (20.3)	gastric cancer
L. Chen	2022	retrospective cohort	China	1	146	59 (34-82)	44 (30.1)	gastric cancer
M. F. Chen	2021	prospective cohort	Taiwan	1	340	NA	NA	esophageal squamous cell carcinoma
S. B. Chen	2021	retrospective cohort	Taiwan	1	620	61 (38-84)	143 (23.1)	esophageal squamous cell carcinoma
W. Chen	2022	retrospective cohort	China	1	83	57.31±11.8; **(27-82)	12 (14.5)	hepatocellular carcinoma
W. Z. Chen	2022	prospective cohort	China	1	742	72 (65-91)	173 (22.9)	gastric cancer
X. Chen	2019	prospective cohort	China	1	575	64.41±10.6	142 (24.7)	gastric cancer

X. Chen	2022	retrospective cohort	China	1	359	62 (55-68)	84 (23.4)	hepatocellular carcinoma
X. Y. Chen	2019	prospective cohort	China	1	313	§62 ††(50-74)	105 (33.5)	gastric cancer
Y. Chen	2020	retrospective cohort	China	2	325	NA	55 (16.9)	hepatocellular carcinoma
Y. Chen	2022	retrospective cohort	China	1	228	64.3±9.6	20 (8.8)	hepatocellular carcinoma
K. Chen	2024	retropective cohort	China	1	1450	53 (46-62)	219 (15.1)	hepatocellular carcinoma
L.Chen	2023	prospective cohort	china	1	78	NA	78 (100)	gastrointestinal
L. Chen	2024	retropective cohort	China	2	426	NA	139 (32.6)	gastric, colorectal
N. Chen	2024	prospective cohort	China	1	354	§62**(41-83)	27 (7.6)	esophageal cancer
W. Chen	2023	retropective cohort	China	2	850	§64 (50-78)	292 (34.4)	colorectal cancer
J. W. Cho	2022	retrospective cohort	Korea	1	1940	55.3±11.5; **(24-82)	33 (17.0)	gastric cancer
Y. Choi	2017	retrospective cohort	Korea	1	288	62 (28-87)	107 (37.2)	gastric cancer
M. O. Chu	2018	retrospective cohort	Taiwam	1	118	69±10; **(32-90)	44 (37.3)	hepatocellular carcinoma
A. da Silva Couto	2023	retrospective cohort	Brazil	1	191	60.5±11.3	81 (42.4)	colon cancer
Y. Dai	2019	retrospective cohort	China	1	106	58 (41-79)	27 (25.5)	cervical esophageal squamous cell carcinoma
T.Dai	2022	case-control	China	1	242	NA	63 (26)	gastric cancer

N. Daitoku	2018	retrospective cohort	Japan	1	211	63 (34-86)	85 (40.3)	colorectal cancer
C. A. D'Almeida	2013	prospective cohort	Brazil	1	72	57.3±12.8	41 (56.9)	gastrointestinal cancer
L. E. Daly	2018	prospective cohort	Ireland	1	225	65.6 (35.8-83.4)	75 (33.3)	gastrointestinal cancer
C. Dang	2022	prospective cohort	China	1	382	57.5 (28-78)	161 (42.1)	PDAC
C. Dang	2022	retrospective cohort	China	1	792	NA	322 (40.7)	PDAC
A. Daniele	2017	cross-sectional	Italy	1	78	78 (44-87)	39 (50)	colorectal cancer
De Luise Roman	2024	prospective cohort	Spain	1	137	§69.8**(55-89)	49 (35.8)	colorectal cancer
G. S. de Castro	2021	cross-sectional	Italy	1	94	NA (30-90)	40 (42.5)	gastrointestinal cancer
M. Demir	2022	retropective cohort	Turkey	1	101	§59**(35-84)	57 (56.4)	esophageal cancer
D. Diakowska	2010	cross-sectional	Poland	1	135	NA	27 (20)	esophageal cancer
W. P. M. Dijksterhuis	2021	prospective cohort	Netherlands	multicenter	406	§66 ††(59-71)	96 (23.6)	gastrointestinal cancer
T. G. Dolin	2022	cross-sectional	Denmark	1	64	79.6±6.4	38 (59)	colorectal cancer
L. Dou	2020	prospective cohort	China	1	201	64.682±10.483	51 (25.4)	gastrointestinal cancer
Y. Du	2025	retropective cohort	China	1	1101	NA	275 (25)	gastric cancer
H. Duan	2022	cross-sectional	China	1	152	NA	24 (15.8)	hepatocellular carcinoma

W. K. Eo	2015	retrospective cohort	Republic of Korea	1	314	59±NA	106 (33.8)	gastric cancer
B. Erdogan	2022	retropective cohort	Turkey	1	52	NA	22 (42.3)	colorectal cancer
J. Ericson	2025	randomized control trial	Sweden/Norway/Germany	multicenter	117	64±NA	22 (18.8)	esophageal or gastroesophageal junction carcinoma
A. Esfahani	2016	prospective cohort	Iran	1	71	62.13±14.39	15 (21.1)	gastric cancer
Z. Fan	2022	prospective cohort	China	NA	279	51 (19-81)	NA	hepatocellular carcinoma
P. Fang	2022	retrospective cohort	China	1	373	NA	68 (18.2)	esophageal cancer
P. Fang	2024	retropective cohort	China	1	314	NA	62 (19.7)	esophageal squamous cell carcinoma
J. Feng	2013	retrospective cohort	China	1	375	59.1±7.8	49 (13.1)	esophageal cancer
J. Feng	2022	retrospective cohort	China	NA	216	63.2±6.6; **(47-75)	13 (6)	esophageal cancer
J. Feng	2024	retropective cohort	China	1	224	NA	17 (7.6)	esophageal squamous cell carcinoma
M. Findlay	2020	retrospective audit	Australia	1	37	64 (41-82)	6 (16.2)	esophageal cancer
A. V. G. Fruchtenicht	2018	prospective cohort	Brazil	1	41	59±12	20 (48.8)	gastrointestinal cancer
K. Fu	2017	prospective cohort	China	1	310	56.3 (41-71)	123 (39.7)	colorectal cancer
L. Fu	2024	cross sectional	China	1	418	NA	124 (29.7)	gastric cancer

Y. Fugane	2025	retropective cohort	China	1	332	NA	104 (31.3)	biliary and pancreatic cancer
H. Fujii	2020	retrospective cohort	Japan	1	44	NA	21 (47.7)	gastric cancer
A. Fujio	2022	retrospective cohort	Japan	1	61	NA	10 (16.4)	hepatocellular carcinoma
Y. Fujiwara	2014	prospective cohort	Japan	1	21	NA	5 (23.8)	PDAC
Y. Fujiwara	2021	retrospective cohort	Japan	1	111	NA	18 (16.2)	esophageal cancer
Y. Fujiwara	2022	retropective cohort	Japan	1	118	66.16±8.08	18 (15.3)	esophageal squamous cell carcinoma
K. Fujiya	2018	prospective cohort	Japan	1	760	NA	239 (31.4)	gastric cancer
Y. Fukuda	2015	retrospective cohort	Japan	1	800	NA	237 (29.6)	gastric cancer
A. Fukuta	2019	prospective cohort	Japan	1	98	NA	63 (64.3)	gastrointestinal cancer
N. Funamizu	2025	retropective cohort	Japan	1	141	68 (43-84)	64 (45.4)	PDAC
N. Funamizu	2022	retropective cohort	Japan	1	139	NA	85 (61.2)	PDAC
H. Furuke	2021	retrospective cohort	Japan	1	795	68 (29-89)	262 (33)	gastric cancer
D. K. Gabrielson	2021	prospective cohort	Canada	1	41	58.5±11.3	18 (43.9)	gastrointestinal cancer
J. M. Gallardo-Valverde	2005	cross-sectional	Spain	1	43	¶ 67 **(46-84)	10 (23.3	colorectal cancer

J. Gao	2019	retrospective cohort	China	1	374	56.8±10.8	75 (20.1)	gastric cancer
A. K. Garth	2010	retrospective cohort	Australia	1	95	66.3±13; **(18-NA)	33 (34.7)	gastrointestinal cancer
C. Gavazzi	2011	prospective cohort	Italy	NA	100	64±13.5	40 (40)	gastric cancer
K. Ge	2021	retrospective cohort	China	1	420	66 (43-86)	112 (26.7)	esophageal junction cancer
Y. Geng	2015	retrospective cohort	China	1	211	NA	77 (36.5)	PDAC
C. Gillis	2021	cross-sectional	Canada	1	266	71±11	112 (42.1)	colorectal cancer
L. A. Gilmore	2022	retrospective cohort	USA	1	879	66.4±12	384 (43.7)	gastrointestinal cancer
B. K. P. Goh	2016	retrospective cohort	Singapore	1	166	66 (21-85)	24 (14.5)	hepatocellular carcinoma
M. J. Goh	2022	retrospective cohort	South Korea	1	116	60 (52-67)	18 (15.5)	hepatocellular carcinoma
M. B. Gómez Sánchez	2010	prospective randomized controlled trial	Spain	1	82	NA	48 (58.5)	gastrointestinal cancer
C. Gong	2022	retrospective cohort	China	1	324	NA	107 (33)	gastric cancer
C. Grinstead	2025	retropective cohort	USA	1	314	70.5±NA; §71**(30-98)	140 (44.6)	PDAC
X. W. Guo	2018	retrospective cohort	China	1	277	62 (40-82)	62 (22.4)	esophageal cancer
Z. Q. Guo	2020	cross-sectional	China	several tertiary public	2322	¶ 62 **(18-90)	694 (29.9)	gastric cancer

				hospitals in China				
H. Guo	2025	retropective cohort	China	1	395	68 (63-74)	208 (52.7)	gastric cancer
M.C. Gül	2025	retropective cohort	Turkey	1	301	NA	87 (28.9)	gastric cancer
R. Hamura	2022	retrospective cohort	Japan	1	124	§70 ††(61-74)	30 (24.2)	biliary tract cancer
J. Han	2021	cross-sectional	China	1	1627	NA	558 (34.3)	gastrointestinal cancer
L. Han	2016	retrospective cohort	China	1	206	60 (32-84)	41 (19.9)	esophageal squamous cell carcinoma
Y. Han	2023	retropective cohort	China	2	650	NA	173 (26.6)	gastric cancer
R. Haneda	2022	retrospective cohort	Japan	1	158	67 (40-82)	18 (11.4)	esophageal squamous cell carcinoma
N. Harimoto	2017	retrospective cohort	Japan	1	357	NA	87 (24.4)	hepatocellular carcinoma
N. Harimoto	2018	retrospective cohort	Japan	13	2461	NA	676 (27.5)	hepatocellular carcinoma
N. Harimoto	2023	retropective cohort	Japan	1	174	NA	31 (17.8)	hepatocellular carcinoma
I. Hashimoto	2024	retropective cohort	Japan	1	203	NA	37 (18.2)	esophageal squamous cell carcinoma
T. Hatada	2000	prospective cohort	Japan	1	100	NA	38 (38)	colorectal cancer

T. Hatanaka	2019	retrospective cohort	Japan	1	178	NA	35 (19.7)	hepatocellular carcinoma
H. Hauner	2020	cross-sectional	Germany	17	192	NA	100 (52.1)	gastrointestinal cancer
T. Hayama	2022	retrospective cohort	Japan	1	259	74.2 (65-93)	115 (44.4)	colorectal cancer
T. Hayama	2020	retrospective cohort	Japan	1	301	67.74 (22-93)	121 (40.2)	colorectal cancer
H. Hayashi	2023	retrospective cohort	Japan	1	303	NA	82 (27.1)	hepatocellular carcinoma
Y. He	2023	retropective cohort	China	1	365	§73	80 (21.9)	esophageal cancer
Y. He	2023	retropective cohort	China	1	148	**(28-78)	60 (40.5)	distal cholangiocarcinoma
A. E. Hendifar	2018	retrospective cohort	USA	1	977	NA	472 (48.3)	PDAC
A. Hiraoka	2023	retropective cohort	Japan	1	525	74 (68-80)	105 (20)	hepatocellular carcinoma
N. Hirahara	2018	retrospective cohort	Japan	1	148	NA	16 (10.8)	esophageal squamous cell carcinoma
N. Hirahara	2018	retrospective cohort	Japan	1	169	NA	19 (11.2)	esophageal squamous cell carcinoma
N. Hirahara	2019	retrospective cohort	Japan	1	210	NA	64 (30.5)	gastric cancer
N. Hirahara	2020	retrospective cohort	Japan	1	297	NA	92 (31	gastric adenocarcinoma

N. Hirahara	2020	retrospective cohort	Japan	1	191	NA	22 (11.5)	esophageal squamous cell carcinoma
K. Hiramatsu	2021	retrospective cohort	Japan	1	821	65 (27-98)	360 (43.8)	colorectal cancer
Ch.-T. Ho	2024	retropective cohort	Taiwan	1	446	65 (58-74)	173 (38.8)	hepatocellular carcinoma
T. H. Hoc	2021	cross-sectional	Vietnam	1	76	56.7±13.2; **(27-79)	23 (30.3)	esophageal, gastric cancer
Z. Hu	2020	retropective cohort	China	1	361	NA	142 (39.3)	colorectal cancer
S. P. Hu	2020	retrospective cohort	China	1	282	58.7±13.5	117 (41.5)	PDAC
Y. Hu	2023	retropective cohort	China	1	282		39 (13.8)	hepatocellular carcinoma
X. H. Hua	2022	prospective cohort	China	1	219	§66 ††(60-70)	73 (33.3)	esophageal squamous cell carcinoma
D. Huang	2021	prospective cohort	China	1	597	72 (65-NA)	134 (22.5)	gastric cancer
D. D. Huang	2022	prospective cohort	China	1	1359	§ 66	359 (26.4)	gastric cancer
D. D. Huang	2021	prospective cohort	China	1	587	§65	155 (26.4)	gastric cancer
P. Y. Huang	2019	retrospective cohort	Taiwan	1	891	58.54±11.6	197 (22.1)	hepatocellular carcinoma
S. Huang	2021	cross-sectional	China	1	60	NA	32 (53.3)	hepatocellular carcinoma
T. H. Huang	2019	retrospective cohort	Taiwan	1	287	§63 ††(56-70)	72 (25.1)	hepatocellular carcinoma

Y. Huang	2019	prospective cohort	China	1	357	73.29±5.24	82 (23)	gastric cancer
W.Huang	2024	retropective cohort	China	1	312	54.1±12.8	113 (36.2)	gastric cancer
Y. Huang	2025	cross sectional	China	1	431	§68**(62-72)	122 (28.3)	gastric cancer
S. Ide	2021	retrospective cohort	Japan	1	93	63 (32-83)	25 (26.9)	rectal cancer
T. Igarashi	2024	retropective cohort	Japan	1	114	NA	43 (37.7)	biliary tract cancer
T. Iguchi	2020	retropective cohort	Japan	1	80	63.6±NA; **(30-86)	36 (45)	colorectal cancer
T. Ikeya	2015	retrospective cohort	Japan	1	80	63 (36-80)	36 (45)	colorectal cancer
S. Ikuta	2023	retropective cohort	Japan	1	213	§72**(39-88)	95 (44.6)	biliary tract cancer
Y. Imaoka	2023	retropective cohort	Japan	multicenter (381 hospital)	1494	NA	332 (22.2)	hepatocellular carcinoma
Y. Iseki	2015	retrospective cohort	Japan	1	204	NA	92 (45.1)	colorectal cancer
M. Ishizuka	2014	retrospective cohort	Japan	1	154	NA	36 (23.4)	gastric cancer
S. Itoh	2021	retropective cohort	Japan	multicenter	589	71 (63-77)	263 (44.7)	PDAC
C. H. Jeon	2020	retrospective cohort	Republic of Korea	1	1307	NA	445 (34)	gastric cancer
H. Jeong	2021	retrospective cohort	Korea	1	130	NA	19 (14.6)	hepatocellular carcinoma
F. Ji	2017	prospective cohort	China	1	322	¶ 51 **(21-79)	36 (11.2)	hepatocellular carcinoma

P.Jia	2024	prospective cohort	China	multicenter	2177	66 (58-73)	789 (36.1)	gastric, colorectal
N. Jiang	2014	retrospective cohort	China	1	386	60 (20-80)	127 (33)	gastric cancer
Y. Jiang	2014	retrospective cohort	China	1	126	NA	65 (51.6)	gastric cancer
C. Jian-Hui	2016	retrospective cohort	China	1	1321	57.5 (18-91)	544 (41.2)	colorectal cancer
H. Jin	2021	retrospective cohort	China	1	272	61 (32-80)	71 (26.1)	gastric adenocarcinoma
J. Jin	2021	retrospective cohort	China	1	511	§58 ††(49-66)	213 (41.7)	ampulla of Vater cancer
Y. Jing	2024	retropective cohort	China	1	490	58.9±NA	134 (27.3)	gastric cancer
N. Johns	2014	cross-sectional	UK	1	41	65±2	11 (26.8)	gastrointestinal cancer
T. Kamada	2023	retrospective cohort	Japan	1	306	71.5 (39-96)	114 (37.3)	colorectal cancer
M. Kanda	2016	retrospective cohort	Japan	1	260	66 (20-96)	68 (26.2)	gastric cancer
G. Kaner	2018	cross-sectional	Turkey	1	119	NA	50 (42)	gastrointestinal cancer
J. Kang	2022	retrospective cohort	China	1	1332	62 (34-90)	244 (18.3)	esophageal cancer
H. Kanno	2021	retrospective cohort	Japan	1	528	NA	93 (17.6)	hepatocellular carcinoma
T. Kapagan	2025	prospective cohort	Finland	1	150	NA	59 (39.3)	colorectal cancer
M. Kataoka	2021	retropective cohort	Japan	1	904	NA	368 (40.7)	colorectal cancer

Y. Kato	2018	retrospective cohort	Japan	1	344	64.8±9.9	137 (39.8)	PDAC
S. Kawahara	2024	retropective cohort	Japan	1	123	NA	58 (47.2)	PDAC
A. Ş. Kaya	2020	cross-sectional	Turkey	1	110	54±8.5	76 (41.8)	gastrointestinal cancer
E. Kazlow	2025	cross sectional	Israel	1	136	66.4±11.7	53 (39)	pancreatic head adenocarcinoma
M. Ke	2016	retrospective cohort	China	1	372	51.74±10.87	74 (19.9)	hepatocellular carcinoma
M. Keskinkilic	2024	cross sectional	Turkey	1	253	61**(27.1-88.6)	97 (38.3)	colorectal cancer
Y.Kim	2024	cross sectional	Republic of Korea	1	162	64.7±8.5	32 (19.8)	hepatocellular carcinoma
Y-J.Kim	2025	retropective cohort	Republic of Korea	1	718	NA	429 (59.7)	Pancreatic, periampullary
E. Kim	2018	prospective cohort	South Korea	1	355	61.8±11.2	145 (40.8)	periampullary neoplasms
E. Kim	2019	prospective cohort	South Korea	1	154	71±4.5	74 (48.1)	periampullary neoplasms
J. R. Kim	2017	conference abstract	Korea	1	117	62.8±NA	NA	PDAC
A. Kinoshita	2022	retrospective cohort	Japan	1	61	NA	8 (13.1)	hepatocellular carcinoma
R. Konaka	2025	retropective cohort	Japan	1	58	NA	22 (37.9)	biliary tract cancer
X. Kong	2025	cross sectional	China	1	160	NA	43 (26.9)	esophageal cancer
M. Korkmaz	2023	prospective cohort	Turkey	1	168	NA	55 (32.7)	gastric cancer

T. Kosuga	2019	retropective cohort	Japan	1	167	67.7±11.6; §69**(29-87)	56 (33.5)	gastric cancer
K. Kouzu	2020	cross-sectional	Japan	1	128	73.2±5.5	15 (11.7)	esophageal cancer
N. Kubo	2017	retrospective cohort	Japan	1	202	63.73±7.93	40 (19.8)	esophageal squamous cell carcinoma
N. Kubo	2018	cross-sectional	Japan	1	240	63.4±7.8; **(66-113)	44 (18.3)	esophageal cancer
D. Kuroda	2018	retrospective cohort	Japan	1	416	¶ 67.2 **(25-94)	149 (35.8)	gastric cancer
N. Kurtul	2017	retrospective cohort	Turkey	1	65	63 (28-81)	22 (33.8)	rectal cancer
A. E. J. Latenstein	2020	prospective cohort	Netherlands	18	94	§70 ††(62-74)	22 (44.7)	PDAC
S. H. Lee	2016	retrospective cohort	Korea	1	499	62.1±10.2	210 (42.1)	PDAC
J. Y. Lee	2016	retrospective cohort	Korea	1	7781	57.1±11.9	2631 (33.8)	gastric adenocarcinoma
S. Y. Lee	2018	retrospective cohort	South Korea	1	1063	NA	357 (33.6)	colon cancer
S. Y. Lee	2019	conference abstract	NA	NA	404	NA	NA	colon cancer
S. Y. Lee	2020	retrospective cohort	South Korea	1	404	NA	188 (46.5)	colon cancer
S. Lee	2023	retropective cohort	Korea	1	131	70.1±10.7	38 (29.0)	rectal cancer
G. Li	2023	prospective cohort	China	1	415	60.83±10.42	47 (11.32)	gastric cancer
H. Li	2024	retropective cohort	China	1	278	§60**(34-80)	33 (11.9)	esophageal cancer

J. Li	2024	retropective cohort	China	1	360	60.54±9.82	90 (25)	hepatocellular carcinoma
J. Li	2023	retropective cohort	China	1	100	NA	30 (30)	hepatocellular carcinoma
J. Li	2023	retropective cohort	China	2	511	NA	154 (30.1)	colorectal cancer
K. Li	2024	retropective cohort	China	1	470	NA	191 (40.6)	colorectal cancer
X.-X. Li	2024	retropective cohort	China	2	228	NA	42 (18.4)	hepatocellular carcinoma
Y. Li	2025	retropective cohort	China	1	2553	NA	1003 (39.3)	colorectal cancer
L. Li	2018	retrospective cohort	China	1	1334	§50 ††(41-60)	198 (14.8)	hepatocellular carcinoma
S. Li	2021	retrospective cohort	China	1	285	59 (31-80)	53 (18.6)	hepatocellular carcinoma
X. Liang	2021	prospective cohort	China	1	868	50.53±12.04; **(12-81)	141 (16.2)	hepatocellular carcinoma
C. K. Liao	2021	retrospective cohort	Taiwan	1	1206	80.45±4.42	573 (47.5)	colorectal cancer
W.-Ch. Liao	2020	prospective cohort	Taiwan	1	176	NA	74 (42.0)	PDAC
Z. X. Lin	2020	retrospective cohort	China	1	380	50 (19-80)	47 (12.4)	hepatocellular carcinoma
Shuxiang Lin	2025	retropective cohort	China	1	491	NA	108 (22)	gastric cancer
Bao Liu	2024	retropective cohort	China	1	146	58.05±9.84	44 (30.1)	gastric cancer
Jianwei Liu	2025	retropective cohort	China	1	234	62.0 (54-68)	85 (35.5)	hepatocellular carcinoma

Jiang Liu	2022	retropective cohort	China	1	158	§67**(52-87)	57 (36.1)	esophageal squamous cell carcinoma
Jiawei Liu	2024	retropective cohort	China	1	1281	§57**(25-88)	533 (41.6)	gastric cancer
Li-Xiang Liu	2024	retropective cohort	China	2	217	58.4±12.7	92 (42.4)	colorectal cancer
Yali Liu	2023	prospective cohort	China	1	182	NA	38 (20.9)	esophageal squamous cell carcinoma
J. Y. Liu	2021	retrospective cohort	China	1	191	56 (26-79)	63 (33)	gastric adenocarcinoma
X. Liu	2018	retrospective cohort	China	1	697	57 (21-86); ††(41-89)	240 (34.4)	gastric cancer
Li LiYa	2024	cross sectional	China	1	80	62.1±7.56	16 (20)	esophageal cancer
K. W. Loh	2012	cross-sectional	Netherlands	1	104	64.7±10.8	41 (39.4)	gastrointestinal cancer
V. Lohsiriwat	2014	prospective cohort	Thailand	1	149	NA	73 (49)	colorectal cancer
S. Lu	2023	retrospective cohort	China	1	172	67 (63-73)	59 (34.3)	rectal cancer
Y. Lu	2019	cross-sectional	China	1	182	NA	87 (47.8)	colorectal cancer
Z. Luo	2019	retrospective cohort	China	multicenter	230	54±12.3	47 (20.4)	gastric adenocarcinoma
Xi Luo	2024	retropective cohort	China	1	301	NA	90 (30)	gastric cancer
Hui Ma	2024	retropective cohort	China	1	246	65.5±7.4	65 (26.4)	esophageal cancer
Huaixing Ma	2025	retropective cohort	China	1	140	NA	38 (27.1)	gastrointestinal

X. Ma	2025	retropective cohort	China	1	404	68 (63-74)	110 (27.2)	gastric cancer
Xinghao Ma	2024	retropective cohort	China	1	404	68 (63-74)	110 (27.2)	gastric cancer
K. Maeda	2014	retrospective cohort	Japan	1	100	60.4±10.6; **(39-87)	45 (45)	colorectal cancer
Y. S. Mao	2020	retrospective cohort	China	1	144	NA	75 (52.1)	PDAC
C. Martínez-Escribano	2022	cross-sectional	Spain	1	325	NA	150 (46.2)	colorectal cancer
T. Matsuda	2021	retropective cohort	Japan	17	316	71 (63-76)	129 (40.8)	Intrahepatic cholangiocarcinoma
R. Matsui	2022	retrospective cohort	Japan	1	512	67.93±11.1	NA	gastric cancer
R. Matsui	2022	retrospective cohort	Japan	1	512	NA	NA	gastric cancer
R. Matsui	2023	retrospective cohort	Japan	1	281	65.03±10.17	87 (31)	gastric cancer
R. Matsui	2023	retrospective cohort	Japan	1	457	67.88±11	156 (34.1)	gastric cancer
Y. Matsumoto	2018	retrospective cohort	Japan	1	191	66 (39-82)	33 (17.3)	esophageal cancer
T. Matsunaga	2022	retrospective cohort	Japan	14	497	80.6±NA	164 (33)	gastric cancer
T. Matsunaga	2024	retropective cohort	Japan	10	105	NA	18 (17.1)	gastric cancer
T. Matsunaga	2024	retropective cohort	Japan	1	102	65.9±12.2	31 (30.4)	gastric cancer

S. F. Maurício	2013	cross-sectional	Brazil	1	70	60.4±14.3	39 (55.7)	colorectal cancer
K. Migita	2013	retrospective cohort	Japan	1	548	67 (24-89)	146 (26.6)	gastric cancer
K. Migita	2018	retrospective cohort	Japan	1	137	NA	21 (15.3)	esophageal squamous cell carcinoma
K. Migita	2018	retrospective cohort	Japan	1	167	67.6±11.3	43 (25.7)	gastric cancer
J. Miller	2020	cross-sectional	UK	1	16	NA	5 (31.3)	gastrointestinal cancer
K. Mimatsu	2017	retrospective cohort	Japan	1	33	NA	6 (18.2)	gastric cancer
M. Mito	2022	retrospective cohort	Japan	1	224	72 (31-90)	72 (32.1)	hepatobiliary cancer
H. Miyamoto	2024	retropective cohort	Japan	1	197	NA	66 (33.5)	gastric cancer
T. Miyazaki	2016	retrospective cohort	Japan	1	192	¶ 65.8 **(42-86)	19 (9.9)	esophageal cancer
Y. Mohri	2013	retrospective cohort	Japan	1	365	NA	142 (38.9)	colorectal cancer
A. Molfino	2021	case-control	Italy	1	31	71±12	15 (48.4)	gastrointestinal cancer
M. Momokita	2022	cross-sectional	Japan	1	217	70.7±10.4 §72	55 (25.3)	gastric cancer
T. Mracek	2011	cross-sectional	UK	1	25	NA	10 (40)	gastrointestinal cancer
F. Mülküt	2025	retropective cohort	Turkey	1	220	60.63±1056	59 (26.8)	gastric cancer

S. Nagata	2021	retrospective cohort	Japan	1	510	NA	124 (24.3)	hepatocellular carcinoma
S. Naghashi	2022	cross-sectional	Iran	1	299	67.39±12.04	91 (30.4)	gastric cancer
S. Nakagawa	2018	retrospective cohort	Japan	1	151	§70 ††(61-75)	72 (47.7)	PDAC
T. Nakagoe	2003	retrospective cohort	Japan	1	62	67 (29-90)	26 (41.9)	colorectal cancer
Y. Nakamura	2022	retrospective cohort	Japan	1	433	§72 ††(65-78)	172 (39.7)	colorectal cancer
Y. Nakamura	2023	retropective cohort	Japan	1	601	71 (64-78)	240 (40)	colorectal cancer
Y. Nakamura	2025	retropective cohort	Japan	1	105	74 (70-80)	51 (48.6)	colorectal cancer
M. Nakatani	2018	retrospective cohort	Japan	1	76	64.2±7.1	10 (13.2)	esophageal squamous cell carcinoma
K. Nakashima1	2022	retropective cohort	Japan	1	175	70 (38-92)	56 (32)	gastric cancer
A. Narasimhan	2018	cross-sectional	Canada	1	42	NA	23 (54.8)	PDAC
S. Negrichi	2020	cross-sectional	Algeria	1	90	NA	37 (41.1)	colorectal cancer
N. I. P. Neto	2018	cross-sectional	Brazil	1	16	NA	6 (37.5)	colorectal cancer
L. T. Ngoc Anh	2024	cross sectional	Vietnam	1	388	60 (51-66)	164 (42.3)	colorectal cancer
X. Nie	2025	retropective cohort	China	1	274	§83**(41-80)	42 (15.3)	eophageal squamous cell carcinoma
R. Nie	2016	retrospective cohort	China	1	660	NA	299 (45.3)	gastric adenocarcinoma

K. Nishibeppu	2024	retropective cohort	Japan	1	69	NA	33 (47.8)	gastric cancer
T. Nishiyama	2025	retropective cohort	Japan	1	291	NA	124 (42.6)	gastrointestinal
J. Nogueiro	2022	retrospective cohort	Portugal	1	203	66.09±12.45; **(54-78)	82 (40.4)	gastric cancer
I. Nonogaki	2023	retrospective cohort	Japan	1	464	62.2±8.2	70 (15.1)	esophageal squamous cell carcinoma
T. Nozoe	2010	retrospective cohort	Japan	1	219	NA (24-90)	93 (42.5)	colorectal cancer
R. Obaid	2025	cross sectional	Kuwait	1	65	NA	28 (43.1)	colorectal cancer
T. Nozoe	2010	cross-sectional	Japan	1	248	NA (27-89)	70 (28.2)	gastric adenocarcinoma
T. Ogata	2022	retrospective cohort	Japan	1	346	70.5 (29-103)	147 (42.5)	colorectal cancer
D. Ogawa	2023	retropective cohort	Japan	1	123	§70	82 (66.6)	Intrahepatic cholangiocarcinoma
C. A. Oh	2012	cross-sectional	South Korea	1	669	56.77±11.44	237 (35.4)	gastric adenocarcinoma
S. E. Oh	2022	cross-sectional	Korea	1	601	67 (30-91)	252 (42)	hepatobiliary cancer
H. Ohama	2025	retropective cohort	Japan	multicenter	484	73 (67-78)	100 (20.6)	hepatocellular carcinoma
T. Ohara	2025	retropective cohort	Japan	1	54	72 (45-96)	34 (63)	gastrointestinal
G. Okada	2021	retrospective randomized controlled trial	Japan	1	117	NA	29 (24.8)	esophageal cancer

A. Okamoto	2023	retropective cohort	Japan	1	145	65 (58-72)	36 (24.8)	colorectal cancer
K. Okadome	2020	retrospective cohort	Japan	1	337	65.9±9.2	37 (11)	esophageal cancer
Y. Okamura	2017	retrospective cohort	Japan	1	375	70 (30-87)	69 (18.4)	hepatocellular carcinoma
K. Okubo	2021	retrospective cohort	Japan	1	90	NA	27 (30)	gastric adenocarcinoma
S. Olaechea	2021	retrospective cohort	USA	1	126	63 (38-86)	26 (20.6)	gastrointestinal cancer
S. Olaechea	2023	retropective cohort	USA	1	882	61 (53-70)	334 (37.9)	colorectal, gastric, esophageal
Satoshi Omiya	2023	retropective cohort	Japan	1	293	70±NA	48 (16.4)	hepatocellular carcinoma
Shizune Onuma	2023	retropective cohort	Japan	1	483	NA	163 (33.7)	gastric cancer
S. Onishi	2020	retrospective cohort	Japan	1	207	68.9±5.4; **(60-81)	38 (18.4)	esophageal squamous cell carcinoma
Í.D. Onur	2024	retropective cohort	Turkey	1	68	NA	41 (60.3)	Gallbladder and cholangiocarcinoma
J. Ose	2019	prospective cohort	Germany	1	52	NA	16 (30.8)	colorectal cancer
Pırıltı Özcan	2024	retropective cohort	Turkey	2	270	61.45±12.27; §63**(23-90)	84 (31.1)	gastric cancer
E. Paillaud	2014	prospective cohort survey - cross sectional analysis	France	2	248	80.5±5.7	122 (49.2)	gastrointestinal cancer

P. Pan	2015	prospective cohort	China	1	154	NA (18-70)	27 (17.5)	esophageal cancer
Yuting Pan	2023	retropective cohort	China	1	268	§59**(18-86)	69 (25.7)	gastric cancer
J. H. Park	2021	retrospective cohort	Korea	1	1281	NA	487 (38)	gastric adenocarcinoma
J. S. Park	2019	retrospective cohort	Korea	1	412	NA	209 (50.7)	PDAC
S. H. Park	2019	retrospective cohort	Republic of Korea	1	1868	57.8±12.5	614 (32.9)	gastric cancer
P. S. Patil	2017	prospective cohort	India	1	600	NA	NA	gastric adenocarcinoma
P. S. Patil	2017	prospective cohort	India	1	600	NA	NA	gastric cancer
J. Peng	2017	retrospective cohort	China	1	274	55 (22-85)	118 (43.1)	colon cancer
W. Peng	2021	retrospective cohort	China	1	266	NA	44 (16.5)	hepatocellular carcinoma
E. Pérez-Cruz	2017	retrospective cohort	Mexico	1	57	57.8±14.5	26 (46)	gastrointestinal cancer
Jennifer B. Permuth	2024	prospective cohort	United States	1	309	NA	156 (50.5)	PDAC
Mara Persano	2023	retropective cohort	Italy	1	773	NA	151 (19.5)	hepatocellular carcinoma
G. Pian	2022	retrospective cohort	China	1	305	¶ 63 **(25-87)	122 (40)	colorectal cancer
M. Piciucchi	2013	retrospective cohort	Italy	NA	291	NA	NA	PDAC
M. Piciucchi	2013	retrospective cohort	Italy	NA	237	NA	NA	PDAC

M. Piciucchi	2013	retrospective cohort	Italy	NA	291	NA	NA	PDAC
Q. Qi	2021	cross-sectional	China	1	407	§ 65	83 (20.4)	esophageal cancer
Y. Qian	2021	retrospective cohort	China	1	309	63.4±0.6	81 (26.2)	gastric cancer
L. Qin	2021	cross-sectional	China	1	217	§60 ††(50-67)	93 (42.9)	gastric cancer
Y. Qin	2024	prospective cohort	China	1	656	61 (53-66)	261 (39.8)	colon cancer
M. Qiu	2015	prospective cohort	China	1	830	59±NA	282 (34)	gastric cancer
Z. Qu	2022	retropective cohort	China	1	215	59±10.49	37 (17.2)	hepatocellular carcinoma
H. Ræder	2018	cross-sectional	Norway	1	97	NA (50-80)	46 (47.4)	colorectal cancer
Wenzhen Ren	2023	retropective cohort	China	1	108	54.83±19.93; §55**(32-76)	42 (38.9)	gastric cancer
A. Riad	2023	retropective cohort	75 countries	multicenter (381 hospital)	5709	64.8±13.5	2432 (42.6)	colorectal, gastric
N. E. Rich	2022	retrospective cohort	US	1	604	§ 60.9	168 (27.8)	hepatocellular carcinoma
M. Rivelsrud	2021	prevalence survey	Norway	1	149	66.6±12.1	70 (47)	PDAC
Guo-Tian Ruan	2023	prospective cohort	China	1	905	59.3±11.5	377 (41.7)	colorectal cancer
S. Ryo	2018	retrospective cohort	Japan	9	626	NA	191 (30.5)	gastric cancer
T. Ryu	2022	retrospective cohort	Japan	1	341	70 (38-91)	134 (39.3)	hepatocellular carcinoma

M. Sachlova	2014	prospective cohort	Czech Republic	1	91	NA	42 (46.2)	gastric cancer
H. Saito	2017	retrospective cohort	Japan	1	453	NA	122 (26.9)	gastric adenocarcinoma
Y. Saito	2017	retrospective cohort	Japan	1	162	NA	43 (26.5)	hepatocellular carcinoma
Y. Saito	2021	retrospective cohort	Japan	1	162	NA	43 (26.5)	hepatocellular carcinoma
T. Sakamoto	2021	retrospective cohort	Japan	1	105	NA	46 (43.8)	PDAC
K. Sakanaka	2019	retrospective cohort	Japan	1	44	66 (55-84)	7 (15.9)	esophageal cancer
Katsuya Sakashita	2025	retropective cohort	Japan	1	236	NA	74 (31.4)	perihilar cholangiocarcinoma
K. Sakurai	2015	retrospective cohort	Japan	1	594	65.9±11	166 (27.9)	gastric cancer
K. Sakurai	2016	retrospective cohort	Japan	1	147	79±3.4	52 (35.4)	gastric cancer
C. A. Santos	2021	cross-sectional	Portugal	1	155	NA	NA	gastric cancer
I. Santos	2021	retrospective cohort	Portugal	1	41	69±11; **(40-87)	18 (43.9)	PDAC
M. Sasahara	2020	retrospective cohort	Japan	9	842	68.6±10.6	259 (30.8)	gastric cancer
M. Sasaki	2019	retrospective cohort	Japan	1	149	64 (26-87)	56 (37.6)	colorectal cancer
M. Sasaki	2020	retrospective cohort	Japan	1	313	73 (65-94)	112 (35.8)	colorectal cancer
R. Sato	2020	retrospective cohort	Japan	1	72	71±11.4; **(37-90)	31 (43.1)	colorectal cancer

R. Sato	2021	retrospective cohort	Japan	1	75	72.2±11.8; **(37-90)	23 (30.6)	colorectal cancer
Ryuichiro Sato	2022	retropective cohort	Japan	1	61	§75**(65-93)	26 (42.6)	colorectal cancer
K. Schütte	2015	cross-sectional	Germany	1	51	66.18±9.82	7 (13.7)	hepatocellular carcinoma
A. S. Senger	2022	retrospective cohort	Turkey	1	314	NA	89 (28.3)	colorectal cancer
M. G. Serna Thomé	2020	prospective cohort	Mexico	1	354	NA	NA	gastrointestinal cancer
Ningzhe Shen	2023	prospective cohort	China	1	385	73±11	154 (40)	colorectal cancer
M. Shibutani	2023	retropective cohort	Japan	1	529	§71**(21-100)	216 (40.8)	colorectal cancer
M. Shibutani	2015	retrospective cohort	Japan	1	32	69 (42-86)	12 (37.5)	colorectal cancer
H. Shim	2013	prospective cohort	Korea	1	435	58.5±11.9; ** (20-80)	192 (44.1)	gastrointestinal cancer
T. Shimagaki	2025	retrospective analyzed from propectively maintained database	Japan	1	128	70.3±0.9	48 (37.5)	biliary tract cancer
A. Shimizu	2024	retropective cohort	Japan	1	59	NA	19 (32.2)	colorectal cancer
Y. Shimizu	2022	retrospective cohort	Japan	1	91	70 (40-84)	43 (47.3)	ampulla of Vater cancer
Y. Si	2022	retrospective cohort	China	1	235	NA	45 (19.1)	hepatocellular carcinoma

J. H. Sim	2021	retropective cohort	Korea	1	1065	NA	209 (19.6)	hepatocellular carcinoma
E. Simoes	2025	case-control	Brazil	2	38	NA	18 (47.4)	colorectal cancer
J. D. P. Soares	2020	cross-sectional	Brazil	1	106	NA (29-83)	21 (19.8)	gastrointestinal cancer
B. Song	2009	cross-sectional	China	1	125	NA	42 (33.6)	gastric cancer
H. Song	2022	retrospective cohort	China	1	202	61 (28-83); ††(52-66)	70 (34.7)	gastric cancer
H. N. Song	2022	prospective cohort	China	1	918	§ 66	363 (39.5)	colorectal cancer
M. Sonoi	2025	retropective cohort	Japan	1	81	68.5±5.3	12 (14.8)	esophageal cancer
V. Soria-Utrilla	2024	retropective cohort	Spain	3	586	68.4±10.2	221 (37.7)	colorectal cancer
B. U. Souza	2018	cross-sectional	Brazil	1	197	60±11.4	85 (43)	colorectal cancer
N. C. Souza	2020	cross-sectional	Brazil	1	188	61±11.4	80 (42.6)	colorectal cancer
N. C. Souza	2021	cross-sectional	Brazil	1	190	60.5±11.3	81 (42.6)	colorectal cancer
N. A. Stephens	2015	prospective cohort	UK	1	92	65±10	26 (28.3)	gastrointestinal cancer
K. Sugawara	2020	retrospective cohort	Japan	1	309	NA	91 (29.4)	gastric cancer
K. Sugawara	2021	retrospective cohort	Japan	1	1166	NA	350 (30)	gastric cancer
F. Sun	2010	cross-sectional	China	1	223	NA	73 (32.7)	gastric cancer
F. Sun	2010	cross-sectional	China	1	190	NA	57 (30)	gastric cancer
F. Sun	2021	retrospective cohort	China	1	1479	60.4±17.3	396 (26.8)	gastric cancer

H. Sun	2022	retrospective cohort	China	1	146	§ 59	44 (30.1)	gastric cancer
J. Sun	2016	retrospective cohort	China	1	117	59 (34-78)	31 (26.5)	gastric cancer
K. Sun	2015	retrospective cohort	China	1	632	¶ 57 **(19-89)	219 (34.7)	gastric cancer
L. Sun	2021	retrospective cohort	China	1	371	61.5±10.4; **(23-88)	172 (46.4)	biliary tract cancer
Y. Sun	2014	cross-sectional	China	1	5118	60±11; **(23-80)	1453 (28.4)	gastrointestinal cancer
C. Sun	2024	retropective cohort	China	1	233	57 (14-78)	100 (42.9)	ampullary adenocarcinoma
D. Sun	2024	prospective cohort	China	1	770	NA	257 (33.4)	gastric cancer
S. Sun	2024	retropective cohort	China	1	49	57 (37–77)	7 (14.3)	hepatocellular carcinoma
Y. Sun	2025	cross-sectional	China	1	1894	63.77±7.86; **(38-87)	295 (15.6)	esophageal cancer
H. Suzuki	2021	retrospective cohort	Japan	1	99	66.2 (39-85)	38 (38.4)	PDAC
S. Suzuki	2019	retrospective cohort	Japan	1	211	NA	70 (33.2)	gastric cancer
J. Szefel	2020	prospective cohort	Poland	1	70	NA	36 (51.4)	colorectal cancer
K. Takagi	2017	retrospective cohort	Japan	1	295	65.8±10.4	54 (18.3)	hepatocellular carcinoma
T. Takahashi	2020	retrospective cohort	Japan	1	86	84±2.7	28 (32.6)	gastric cancer
T. Takahashi	2024	retropective cohort	Japan	1	80	¶83	26 (32.5)	gastric cancer

Y. Takamizawa	2020	retrospective cohort	Japan	1	996	61 (20-91)	423 (42.5)	colorectal cancer
K. Takao	2020	retrospective cohort	Japan	1	263	NA	46 (17.5)	esophageal squamous cell carcinoma
Y. Takano	2023	retropective cohort	Japan	2	396	**(23-93)	1164 (41.4)	colorectal cancer
H. Takechi	2020	retrospective cohort	Japan	1	182	70 (38-90)	52 (28.6)	gastric cancer
T. Takeda	2021	retrospective cohort	Japan	1	80	77 (75-87)	NA	PDAC
E. E. Talbert	2018	prospective cohort	USA	1	70	NA	37 (52.9)	PDAC
M. Tamai	2021	retrospective cohort	Japan	1	227	NA	26 (11.5)	colorectal cancer
Y. Tamai	2022	retrospective cohort	Japan	1	181	71.4±7.9	52 (28.7)	hepatocellular carcinoma
F. Tambaro	2024	prospective cohort	Italy	1	23	72.1±11.6	12 (52.2)	gastrointestinal
S. Tan	2024	cross-sectional	China	1	207	56.6±11.3	37 (17.9)	hepatocellular carcinoma
S. Tan	2022	prospective cohort	China	1	1115	62.6±10.8	370 (33.2)	gastrointestinal cancer
M. Tanabe	2025	retropective cohort	Japan	1	940	§69**(27-90)	325 (34.6)	gastric cancer
K. Tanaka	2021	cross-sectional	Japan	1	127	§71 ††(63-76)	59 (46.5)	colorectal cancer
H. Tanda	2025	retropective cohort	Japan	1	298	§71**(27-100)	121 (40.6)	colorectal cancer
A. Tanemura	2020	retropective cohort	Japan	1	189	§70**(41-85)	36 (19.0)	hepatocellular carcinoma

M. Tang	2025	retropective cohort	China	1	389	NA	157 (40.4)	rectal cancer
J. Tang	2021	retrospective cohort	USA	1	506	58.85±12.98; § 59	242 (47.8)	colorectal cancer
M. Tang	2021	prospective cohort	China	4	527	59.56±11.58	129 (24.5)	gastrointestinal cancer
Y. Tanji	2022	retropective cohort	Japan	1	118	§66**(60-75)	37 (31.6)	colorectal cancer
Z. Tao	2024	cross-sectional	China	1	98	62±13	36 (36.7)	gastrointestinal
S. Tekin	2023	retropective cohort	Turkey	1	124	58.16±11.04	41 (33.1)	gastric cancer
F. Teraishi	2025	prospective cohort	Japan	1	24	89 (86-91)	14 (58.3)	colorectal cancer
F. Teraishi	2024	prospective cohort	Japan	1	71	78 (75-92)	37 (52.1)	colorectal cancer
F. Terasaki	2020	retrospective cohort	Japan	1	149	NA	34 (22.8)	distal cholangiocarcinoma
F. Terasaki	2021	retrospective cohort	Japan	1	307	NA	125 (40.7)	PDAC
I. Tirnova	2025	retropective cohort	Turkey	1	68	66.1±13.3	28 (41.2)	gastric cancer
S. Tohme	2017	editor letter	USA	1	176	NA	53 (30.1)	hepatocellular carcinoma
R. Tokunaga	2015	retrospective cohort	Japan	1	556	68 (19-93)	226 (40.6)	colorectal cancer
R. Tokunaga	2016	retrospective cohort	Japan	1	417	68 (19-93)	170 (40.8)	colorectal cancer
T. Tominaga	2019	retrospective cohort	Japan	1	250	NA	122 (48.8)	colorectal cancer

T. Tominaga	2020	retrospective cohort	Japan	1	896	65 (28-93)	492 (55)	colon cancer
A. Topçu	2022	retrospective cohort	Turkey	1	77	58.7±13.6	24 (31.2)	gastric cancer
T. Toyokawa	2016	retrospective cohort	Japan	1	185	§64 ††(59-70)	33 (17.8)	esophageal squamous cell carcinoma
I. Trestini	2020	retrospective cohort	Italy	1	73	65±11	32 (43.8)	PDAC
M. Tsunematsu	2021	retrospective cohort	Japan	1	111	NA	19 (17.1)	hepatocellular carcinoma
M. Tsukagoshi	2024	retropective cohort	Japan	1	203	NA	37 (18.2)	hepatocellular carcinoma
M. Y. Tu	2012	prospective cohort	China Taiwan	1	45	62±11.5	20 (44.4)	colorectal cancer
F. Tustumi	2024	retropective cohort	Brazil	1	529	62.8±NA; **(22-94)	214 (40.5)	gastric cancer
G. Ucar	2020	retrospective cohort	Turkey	1	308	57.5 (25-83)	114 (37)	colorectal cancer
S. Uemura	2022	retrospective cohort	Japan	1	110	66 (38-84)	40 (44)	PDAC
R. Umino	2022	retrospective cohort	Japan	1	1272	66 (28-89)	316 (24.8)	hepatocellular carcinoma
S. Unome	2025	retropective cohort	Japan	1	200	73 (66-79)	40 (20)	hepatocellular carcinoma
M. Urabe	2021	retrospective cohort	Japan	1	224	NA	41 (18.3)	esophageal cancer
G. van der Kroft	2018	prospective cohort	the Netherlands	1	63	69±10.5	23 (36)	colorectal cancer

A. van der Werf	2018	randomized controlled trial	the Netherlands	1	69	65±11	23 (33)	colorectal cancer
P. Vashi	2017	cross-sectional	US	1	84	54.5 (31-73) ¶ 53.4	36 (42.9)	gastrointestinal cancer
P. G. Vashi	2019	retrospective cohort	USA	1	112	53.3 (18-NA)	49 (43.8)	colorectal cancer
R. N. Velasco	2022	cross-sectional	Philippines	1	292	57±11.8	140 (47.9)	colorectal cancer
E. Vieira Maroun	2024	cross-sectional	Spain	1	35	62.8±8.8	9 (25.7)	esophageal, gastric cancer
Q. Wan	2022	retrospective cohort	China	1	379	60.42±11.06	145 (38.3)	colorectal cancer
C. Wang	2019	retrospective cohort	China	1	95	NA	7 (7.4)	esophageal cancer
P. Wang	2021	prospective cohort	China	1	189	65.1±7.2	59 (31.2)	esophageal cancer
P. Y. Wang	2021	cross-sectional	China	1	192	65.1±7.2	61 (31.8)	esophageal cancer
H. X. Wang	2018	retrospective cohort	China	1	274	61±3.2	82 (29.9)	gastric cancer
J. Wang	2018	prospective cohort	China	1	97	NA	14 (14.4)	esophageal cancer
J. Wang	2021	retrospective cohort	China	1	430	62.5±10.9	229 (53.3)	biliary tract cancer
J. Wang	2023	prospective cohort	China	1	1513	63±10.9	509 (33.6)	gastrointestinal cancer
L. Wang	2020	retrospective cohort	China	1	170	61.14±11.47	43 (25.4)	gastric cancer
W. J. Wang	2020	retrospective cohort	China	1	131	NA	54 (41.2)	colorectal cancer

X. Wang	2020	retrospective cohort	China	1	281	55.9±12.1	96 (34.2)	rectal cancer
X. Wang	2020	retrospective cohort	China	1	120	NA	50 (41.7)	colorectal cancer
X. B. Wang	2019	retrospective cohort	China	1	209	NA (18-75)	37 (17.7)	hepatocellular carcinoma
Y. Wang	2018	prospective cohort	China	1	264	NA	76 (28.8)	colorectal cancer
Y. Wang	2020	retrospective cohort	China	2	52	74 (70-83)	18 (34.6)	esophageal squamous cell carcinoma
L.-J. Wang	2025	retropective cohort	China	1	803	**(22-87)	260 (32.4)	gastric cancer
S. Wang	2025	retropective cohort	China	2	157	70.1±4.2	83 (52.9)	Intrahepatic cholangiocarcinoma
X. Wang	2023	retropective cohort	China	1	195	NA	60 (30.8)	gastric cancer
X. Wang	2024	retropective cohort	China	1	391	64.7±11.72	150 (38.4)	colorectal cancer
A. Wang	2021	retropective cohort	China	1	94	54.5±8.6	31 (33)	hilar cholangiocarcinoma
Y. Wang	2022	prospective cohort	China	2	1637	57.7±10.6; **(18-85)	705 (43.1)	colorectal cancer
H. Watanabe	2021	retrospective cohort	Japan	1	110	NA	31 (282)	gastrointestinal cancer
M. Watanabe	2012	retrospective cohort	Japan	1	99	NA (75-79)	23 (23.2)	gastric cancer
A. G. Wijma	2024	prospective cohort	The Netherlands	multicenter	30	NA	17 (56.6)	PDAC

M. Wobith	2022	cross-sectional	Germany	1	260	70.15 (37-92)	113 (43.5)	gastrointestinal cancer
J. H. Wolf	2020	retrospective cohort	US and Canada	700	9289	NA	3595 (38.7)	rectal cancer
G. Wong	2021	prospective cohort	US	1	333	NA	157 (47.1)	colorectal cancer
S. J. Wu	2016	retrospective cohort	China	1	450	¶ 49.63 **(17-81)	59 (13.1)	hepatocellular carcinoma
T. Wu	2022	prospective cohort	China	multicenter	3621	64.09±12.45	1439 (39,7)	colorectal cancer
Q. Wu	2025	retropective cohort	China	multicenter	350	69.6±NA	54 (15.4)	hepatocellular carcinoma
T. Wu	2024	retropective cohort	China	multicenter	4487	64.18±12.42	1799 (40.1)	colorectal cancer
X. Xi	2022	retrospective cohort	China	1	812	63.6±9.2	269 (33.1)	gastric cancer
L. J. Xia	2020	retrospective cohort	China	1	154	¶ 63.7 **(32-90)	64 (41.6)	rectal cancer
S. Xiang	2023	retropective cohort	China	1	236	45 (39-48)	93 (39.4)	colorectal cancer
A. T. Xiao	2020	retrospective cohort	China	1	202	52.6±10.8	76 (37.6)	gastric cancer
Y. Xiao	2022	retropective cohort	China	1	2352	NA	789 (33,5)	gastric cancer
F. K. Xiao	2021	retrospective cohort	China	1	4146	61±8.9	1523 (36.7)	esophageal squamous cell carcinoma
L. Xiao	2021	retrospective cohort	China	1	193	64 (34-86)	44 (22.8)	esophageal squamous cell carcinoma

Q. Xiao	2022	cross-sectional	China	1	106	67 (43-85); ¶ 64	22 (20.8)	gastric cancer
H. Xie	2020	retrospective cohort	China	1	512	58.48±13.22	188 (36.7)	colorectal cancer
H. Xie	2022	retrospective cohort	China	1	1014	57.33±13.34	375 (37)	colorectal cancer
H. Xie	2022	retrospective cohort	China	1	1014	57.33±13.34	375 (37)	colorectal cancer
Z. Xishan	2020	retrospective cohort	China	1	245	NA	66 (26.9)	gastric cancer
L. B. Xu	2022	retrospective cohort	China	1	895	NA	233 (26)	gastric cancer
L. B. Xu	2022	retrospective cohort	China	1	1188	§66 ††(58-73)	319 (26.9)	gastric cancer
S. Xu	2018	cross-sectional	China	1	258	NA (46-80)	76 (29.5)	esophageal cancer
X. Xu	2022	retrospective cohort	China	1	370	61 (40-81)	125 (33.8)	esophageal squamous cell carcinoma
Y. S. Xu	2021	retrospective cohort	China	1	413	NA	153 (37)	colon cancer
Z. Xu	2022	retrospective cohort	China	1	771	§ 58.2	165 (21.4)	gastric mucosal adenocarcinoma
S. Xu	2023	retropective cohort	China	1	263	43.63±4.62; **(33-72)	65 (24.7)	Gastroesophageal Junction Adenocarcinoma
Y. Xu	2024	retropective cohort	China	1	146	NA	44 (30.1)	gastric cancer
T. Yagyu	2022	retrospective cohort	Japan	11	348	NA	187 (53.7)	colorectal cancer

M. Yamamoto	2019	retrospective cohort	Japan	1	522	NA	231 (44.3)	colorectal cancer
M. Yamamoto	2025	retropective cohort	Japan	1	1635	§67**(25-93)	520 (31.8)	gastric cancer
I. Yamana	2015	retrospective cohort	Japan	1	122	63.9±9.1; **(43-83)	21 (17.2)	esophageal cancer
R. Yamanaka-Kohno	2021	retrospective case-control	Japan	1	73	65 (60-71.5)	5 (6.8)	esophageal cancer
K. Yan	2022	retrospective cohort	China	1	192	73 (65-88)	81 (42.2)	esophageal squamous cell carcinoma
X. Yan	2024	prospective cohort	China	1	983	NA	358 (36.4)	colorectal cancer
Ch.-K. Yang	2024	retropective cohort	China	1	1666	NA	247 (14.8)	hepatocellular carcinoma
J.-Y- Yang	2024	retropective cohort	China	1	121	69.07±5.51	77 (63.6)	gastrointestinal
Y. Yang	2024	prospective cohort	China	1	100	NA	49 (49)	gastric cancer
Y. Yang	2020	retrospective cohort	China	1	403	NA	74 (18.4)	hepatocellular carcinoma
S. Yasui-Yamada	2020	retrospective cohort	Japan	1	501	§70 ††(63-76)	185 (36.9)	gastrointestinal cancer
X. J. Ye	2018	cross-sectional	China	2	255	76.5±4.8	95 (37.3)	gastrointestinal cancer
I. Yildirim	2021	retrospective cohort	Turkey	1	219	66.6±11.7	80 (36.5)	PDAC
T.N. Yildiz Kopur	2024	prospective cohort	Turkey	1	121	62.3±12.08	45 (37.2)	colorectal cancer

L. Yin	2021	prospective cohort	China	1	360	64.08±7.74	69 (19.2)	esophageal cancer
Y. J. Yoo	2020	retrospective cohort	South Korea	1	118	61.1±10.2	54 (45.8)	ampulla of Vater cancer
N. Yoshida	2017	retropective cohort	Japan	1	373	NA	41 (11)	esophageal cancer
K. Yoshimatsu	2017	retrospective cohort	Japan	1	351	68 (29-92)	145 (41.3)	colorectal cancer
Y. Yu	2022	retropective cohort	China	1	1068	NA	299 (28)	esophageal cancer
J. H. Yun	2024	retropective cohort	Republic of Korea	1	116	59.35±10.98	28 (24.1)	gastric cancer
J. Zhang	2024	cross-sectional	China	1	226	60.16±10.47; **(34-87)	35 (15.5)	hepatocellular carcinoma
L. Zhang	2023	prospective cohort	China	1	955	NA	310 (32.5)	rectal cancer
X. Zhang	2025	prospective cohort	China	multicenter	3158	67 (58-74)	980 (31.0)	gastric cancer
Y. Zhang	2023	retropective cohort	China	2	54	§70.5**(39-87)	12 (22.2)	gastric cancer
C. Zhang	2016	retrospective cohort	China	1	173	NA	66 (38.2)	hepatobiliary cancer
C. Zhang	2021	cross-sectional	China	1	249	70.29±3.92; **(60-79)	135 (54.2)	gastric cancer
D. Zhang	2007	prospective cohort	China	1	214	NA	87 (40.7)	gastric cancer
D. Zhang	2008	prospective cohort	China	1	126	NA	53 (42.1)	PDAC
F. M. Zhang	2021	prospective cohort	China	1	1056	NA	278 (26.3)	gastric adenocarcinoma

H. Zhang	2018	retrospective cohort	China	1	655	61 (27-88)	118 (1)	esophageal squamous cell carcinoma
Q. Zhang	2022	cross-sectional	China	1	265	70 (66-74)	7 (27.2)	gastrointestinal cancer
X. Zhang	2020	retrospective cohort	China	1	274	62.2 (27-83)	92 (33.6)	gastric cancer
X. Zhang	2021	retrospective cohort	China	1	454	§ 71	109 (24)	gastric cancer
Y. Zhang	2020	prospective cohort	China	1	273	69 (26-87)	82 (30)	gastric cancer
Y. X. Zhang	2022	retrospective cohort	The United States	more than 1000	12785	NA	5979 (46.8)	PDAC
X. Zhao	2022	retrospective cohort	China	1	783	70 (65-86); ††(67-74)	199 (25.4)	gastric cancer
Y. Zhao	2018	retrospective cohort	China	1	243	56 (25-82)	92 (37.9)	colorectal cancer
Y. Zhao	2022	retrospective cohort	China	1	354	67 (41-90)	148 (41.8)	esophageal squamous cell carcinoma
X.-H. Zhao	2023	retropective cohort	China	1	48	§65	16 (33.3)	esophageal squamous cell carcinoma
X.-N. Zhao	2024	prospective collected, retrospectively analyzed	China	1	783	§70**(65-86)	199 (25.4)	gastric cancer
H.-I. Zheng	2023	prospective collected, retrospectively analyzed	China	1	1121	NA	267 (23.8)	gastric cancer

J. Zheng	2025	cross-sectional	China	multicenter (25 hospitals)	1406	59 (51-65)	398 (28.3)	gastric cancer
J. Zheng	2020	retrospective cohort	China	1	154	71 (67-74)	37 (24)	hepatocellular carcinoma
Z. Zheng	2022	retrospective cohort	China	1	165	62.67±7.95; **(44-85)	34 (20.6)	esophageal squamous cell carcinoma
Z. F. Zheng	2018	retrospective cohort	China	1	532	61.1±11.5	129 (24.2)	gastric cancer
C. J. Zhou	2020	retrospective cohort	China	1	381	65±16	147 (38.6)	rectal cancer
J. Zhou	2022	retrospective cohort	China	1	69	NA	22 (31.9)	colorectal cancer
L. P. Zhou	2021	prospective cohort	China	1	768	NA	269 (35)	gastrointestinal cancer
H. Zhou	2020	retropective cohort	China	1	400	NA	157 (39.3)	colorectal cancer
X. Zhou	2017	retrospective cohort	China	1	187	61 (38-78)	72 (38.5)	esophageal squamous cell carcinoma
C. Zhu	2020	retrospective cohort	China	1	243	NA	9 (20.2)	gastric cancer
X. Zhu	2021	retrospective cohort	China	1	245	NA	66 (26.9)	gastric cancer
Y. Zhu	2021	retrospective cohort	China	1	196	NA	78 (39.8)	colorectal cancer
X. Zhu	2025	retropective cohort	China	1	212	66 (57-74)	82 (38.7)	colorectal cancer

C. L. Zhuang	2022	prospective cohort	China	1	1215	§ 65	329 (27.1)	gastric adenocarcinoma
Y. Zou	2025	retropective cohort	China	1	578	60 (52-64)	217 (37.5)	gastric cancer
W. Zou	2024	retropective cohort	China	1	77	§62**(41-79)	15 (19.5)	esophageal squamous cell carcinoma
J. Zuo	2024	cross-sectional	China	1	302	68 (62-73)	89 (29.5)	gastric cancer
J. Zuo	2024	cross-sectional	China	1	311	66.1±8.9	90 (29)	gastric cancer
J. Zuo	2024	cross-sectional	China	1	316	68 (62-72,25)	92 (29.1)	gastric cancer
J. Zuo	2023	prospective cohort	China	1	248	68 (61-72)	71 (28.6)	gastric cancer

PDAC= Pancreatic ductal adenocarcinoma; NA= not available; §= median; ¶= mean; **=range; ††=interquartile range

Data reported as mean with standard deviation or median with interquartile range, unless otherwise specified.

Supplementary Table S3.: Summary plot of the risk of bias assessment

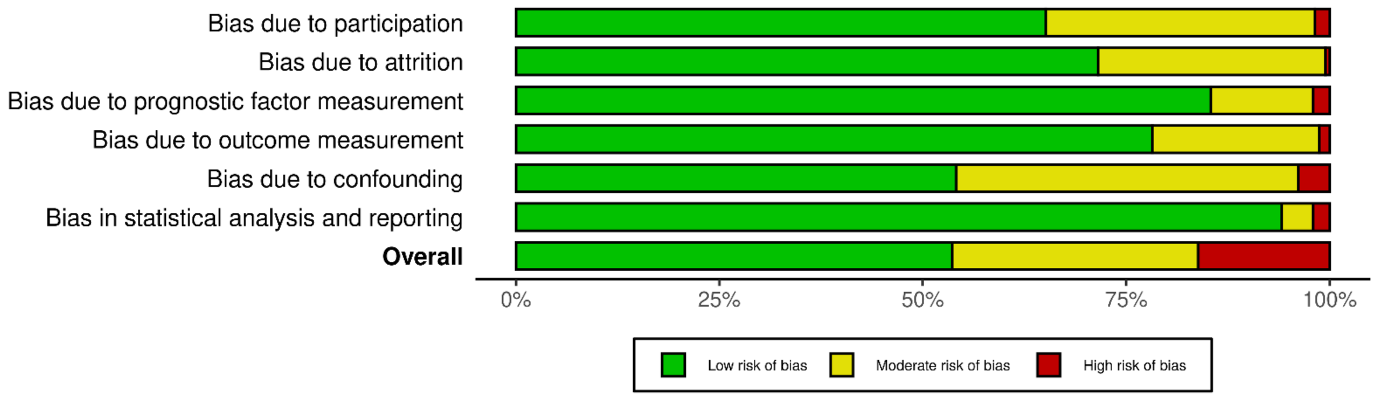

McGuinness, LA, Higgins, JPT. Risk-of-bias VISualization (robvis): An R package and Shiny web app for visualizing risk-of-bias assessments. Res Syn Meth. 2020; 1- 7. https://doi.org/10.1002/jrsm.1411

The risk of bias for study participation, attrition, prognostic factor measurement, outcome measurement, study confounding, and statistical analysis reporting was low.
